# Environmental Viral Genomes Shed New Light on Virus-Host Interactions in the Ocean

**DOI:** 10.1128/mSphere.00359-16

**Published:** 2017-03-01

**Authors:** Yosuke Nishimura, Hiroyasu Watai, Takashi Honda, Tomoko Mihara, Kimiho Omae, Simon Roux, Romain Blanc-Mathieu, Keigo Yamamoto, Pascal Hingamp, Yoshihiko Sako, Matthew B. Sullivan, Susumu Goto, Hiroyuki Ogata, Takashi Yoshida

**Affiliations:** a Institute for Chemical Research, Kyoto University, Uji, Kyoto, Japan; b Graduate School of Agriculture, Kyoto University, Kyoto, Japan; c Department of Microbiology, the Ohio State University, Columbus, Ohio, USA; d Research Institute of Environment, Agriculture and Fisheries, Osaka Prefecture, Osaka, Japan; e CNRS, IGS UMR 7256, Aix Marseille Université, Marseille, France; f Department of Civil, Environmental and Geodetic Engineering, the Ohio State University, Columbus, Ohio, USA; National Institute of Advanced Industrial Science and Technology

**Keywords:** genome, marine ecosystem, metabolism, metagenomics, virus

## Abstract

Metagenomics has revealed the existence of numerous uncharacterized viral lineages, which are referred to as viral “dark matter.” However, our knowledge regarding viral genomes is biased toward culturable viruses. In this study, we analyzed 1,600 (1,352 nonredundant) complete double-stranded DNA viral genomes (10 to 211 kb) assembled from 52 marine viromes. Together with 244 previously reported uncultured viral genomes, a genome-wide comparison delineated 617 genus-level operational taxonomic units (OTUs) for these environmental viral genomes (EVGs). Of these, 600 OTUs contained no representatives from known viruses, thus putatively corresponding to novel viral genera. Predicted hosts of the EVGs included major groups of marine prokaryotes, such as marine group II *Euryarchaeota* and SAR86, from which no viruses have been isolated to date, as well as *Flavobacteriaceae* and SAR116. Our analysis indicates that marine cyanophages are already well represented in genome databases and that one of the EVGs likely represents a new cyanophage lineage. Several EVGs encode many enzymes that appear to function for an efficient utilization of iron-sulfur clusters or to enhance host survival. This suggests that there is a selection pressure on these marine viruses to accumulate genes for specific viral propagation strategies. Finally, we revealed that EVGs contribute to a 4-fold increase in the recruitment of photic-zone viromes compared with the use of current reference viral genomes.

**IMPORTANCE** Viruses are diverse and play significant ecological roles in marine ecosystems. However, our knowledge of genome-level diversity in viruses is biased toward those isolated from few culturable hosts. Here, we determined 1,352 nonredundant complete viral genomes from marine environments. Lifting the uncertainty that clouds short incomplete sequences, whole-genome-wide analysis suggests that these environmental genomes represent hundreds of putative novel viral genera. Predicted hosts include dominant groups of marine bacteria and archaea with no isolated viruses to date. Some of the viral genomes encode many functionally related enzymes, suggesting a strong selection pressure on these marine viruses to control cellular metabolisms by accumulating genes.

## Abstract (precis)

Viruses are diverse and play significant ecological roles in marine ecosystems. However, our knowledge of genome-level diversity in viruses is biased toward those isolated from few culturable hosts. Here, we determined 1,352 nonredundant complete viral genomes from marine environments. Lifting the uncertainty that clouds short incomplete sequences, whole-genome-wide analysis suggests that these environmental genomes represent hundreds of putative novel viral genera. Predicted hosts include dominant groups of marine bacteria and archaea with no isolated viruses to date. Some of the viral genomes encode many functionally related enzymes, suggesting a strong selection pressure on these marine viruses to control cellular metabolisms by accumulating genes.

## INTRODUCTION

Viruses outnumber microbes such as bacteria in the oceans (1), and the destructive lytic infections caused by viruses are thought to have crucial effects on energy and nutrient cycles driven by marine microorganisms (2, 3). Genomics-based research has been a powerful approach used to clarify the biology of viruses, including their infection strategies as well as their ecological significance (4–7). However, the diversity of viral genomes is still underrepresented in publically available genome databases (8, 9). For example, SAR11 (*Pelagibacterales*) and SAR116 are major marine prokaryotic components, but only four and one phage genomes have been sequenced for these bacteria, respectively (10, 11). Cyanophages, for which about 100 genomes have already been characterized, are the sole exception.

To address the issue of the paucity of viral genomic data, Roux et al. analyzed publicly available prokaryotic genome sequence data to mine marine and nonmarine viral genomes that have been sequenced along with the genomes of their hosts (12). They identified 12,498 viral DNA sequences (either long fragments or whole circular genomes) representing 264 predicted new genera.

Culture-independent viral metagenomics is also an effective research option for analyzing viral genomes in complex marine microbial communities (9, 13–16). A decisive advantage of viral metagenomics stems from the small genomes of viruses. Viral genomes have so far been assembled from the metagenomes of the following viral types: RNA viruses (17, 18), single-stranded DNA (ssDNA) viruses (19–26), and double-stranded DNA (dsDNA) viruses (27–29). Among these viruses, the genomes of dsDNA viruses have been the most difficult to assemble from metagenomes because of their relatively large genomes. However, recent advances in the construction of libraries (30), sequencing technologies, and bioinformatics software have resulted in the generation of larger assemblies. For example, 7 complete dsDNA viral genomes have been reported for a hypersaline lake (27), 18 for the deep-sea hydrothermal vent plumes (28), and 54 for glacial cryoconite holes (29). An interesting approach involved the construction of metagenomic fosmid libraries from virus-infected prokaryotes, which revealed 1 (31) and 42 (32) complete viral DNA genomes for solar salterns and 208 marine tailed-phage genomes (33). These studies indicated that marine viral metagenomics investigations have advanced from focusing on environmental genetics (i.e., collections of genes) to analyzing environmental genomics (i.e., collections of complete genomes), helping to unveil the evolutionary histories, life cycles, and metabolic strategies of individual viruses. In this study, we analyzed nine novel marine viral metagenomes (i.e., viromes) generated using a benchtop Illumina/MiSeq sequencer as well as previously published large-scale viromes (9). We identified 1,352 nonredundant complete viral genomes, the vast majority of which corresponded to previously unidentified viral lineages.

## RESULTS AND DISCUSSION

### Choice of assemblers.

We generated nine viromes (Osaka Bay viromes [OBVs]; 8.5 M read pairs; 2.4 Gbp) from water samples collected over a 24-h period in Osaka Bay, Japan (see Materials and Methods). We first compared four assemblers (SPAdes [34], metaSPAdes, IDBA-UD [35], and Ray Meta [36]) regarding their ability to assemble viromes. SPAdes, metaSPAdes, and IDBA-UD clearly outperformed Ray Meta in terms of the total size of >10-kb contigs (Table 1). Of the first three assemblers, SPAdes (11.9 Mb) produced the largest assemblies (i.e., metaSPAdes, 6.8 Mb; IDBA-UD, 5.3 Mb). Regarding assembly error rates assessed by REAPR (37), SPAdes (8.48 regions/kb), metaSPAdes (8.73), and IDBA-UD (8.80) had similar error rates, which were slightly higher than that of Ray Meta (6.42). Most (99.97%) of these errors were short insertion/deletions (REAPR type 1 and type 3 errors), while there were very few (0 to 0.00662 regions/kb) scaffolding errors (type 2 and type 4 errors) (Table 1). On the basis of these results, we chose SPAdes as the best assembler for the following analyses.

**TABLE 1:** Comparison of four assemblers

Parameter	Value			
	SPAdes	metaSPAdes	IDBA	Ray
Assembly size (for contigs >10 kb)	11,869,699	6,818,200	5,264,822	471,387
				
REAPR error types^a^				
FCD error (type 1)	0.01490	0.01045	0.01083	0.00470
FCD error over a gap (type 2)	0.00000	0.00000	0.00000	0.00000
Low-coverage error (type 3)	8.46559	8.71562	8.78596	6.41814
Low-coverage error over a gap (type 4)	0.00414	0.00662	0.00000	0.00000
Total no. of errors	8.48463	8.73268	8.79678	6.42284

a Error values are presented as the number of times the error occurs per 1 kb for contigs longer than 1 kb. Type 1 and 3 errors were associated with short insertion/deletions. Type 2 and 4 errors were associated with scaffolding errors (e.g., chimeric assemblies). FCD, fragment coverage distribution.

### Forty-six genomes assembled from the Osaka Bay viromes.

Given that the nine samples were collected at the same location over a short period and that the reads were relatively long (i.e., 2 × 150 or 2 × 300 bp), a coassembly consisting of the pooled nine samples was also prepared. The coassembly resulted in 879 contigs (>10 kb) that likely originated from dsDNA viruses (see Materials and Methods). Of these, 46 (28.5 to 192 kb; average, 54.2 kb) were assembled in a circular form (see Fig. S1 in the supplemental material). Thus, we refer to these 46 contigs as environmental viral genomes (EVGs).

10.1128/mSphere.00359-16.2 FIG S1  Length and read coverage distributions of OBV contigs. Lengths and read coverages of 46 EVGs (i.e., circular contigs; red) and 833 noncircular contigs (blue) are presented. Boxes represent first quartile, median, and third quartile. (A) Lengths. (B) Coverages. Download FIG S1, PDF file, 1.7 MB. Copyright © 2017 Nishimura et al. 2017 Nishimura et al. This content is distributed under the terms of the Creative Commons Attribution 4.0 International license.

The EVGs did not contain any scaffolding errors (REAPR type 2 and type 4 errors), indicating high structural integrity for the contigs. To further assess the integrity of these EVGs, we mapped the contigs assembled from individual viromes on the EVGs. Of the 46 EVGs, 16 were totally covered by the contigs from individual assemblies, thus decreasing the possibility of artefactual chimeras due to coassembly for these 16 EVGs. The remaining 30 EVGs contained 1 to 24 regions (229 in total) that were supported only by coassembly and were not observed in the individually assembled contigs. We randomly selected 21 such weakly supported regions and tested the coassemblies by PCR assays (using the environmental DNA samples as a template) and sequencing. The results verified all of the tested regions of the coassembled contigs (Fig. S2A). Furthermore, 18 of the 46 EVGs exhibited complete or nearly complete genomic colinearity with closely related reference genomes (Fig. S2B; see Materials and Methods for the definition of genomic colinearity) or with the other independently determined EVGs described below (Fig. S2C). These results further corroborated the accuracy of the overall structure of the EVG assemblies.

10.1128/mSphere.00359-16.3 FIG S2  Assessment of the integrity of OBV contigs. (A) PCR validation of 21 weakly supported regions of OBV_N00005, OBV_N00020, OBV_N00021, and OBV_N00023. All PCR products for the 21 regions were the expected sizes, and their sequences confirmed the OBV coassemblies. Ladder: 2-log DNA ladder (0.1 to 10.0 kb; New England Biolabs, Ipswich, MA). (B) Dot plots of 46 OBV-EVGs and their most closely related reference genomes. The sequences in the comparison were selected on the basis of the best *S*_G_ score for the RVG data set (i.e., prokaryotic dsDNA viruses). (C) Dot plots of 46 OBV-EVGs and their most closely related viral genomes. The sequences in the comparison were selected on the basis of the best *S*_G_ score for the EVGs and RVGs (dsDNA viruses). Genome sequence IDs beginning with “TARA_” represent TOV-EVGs obtained in this study, and IDs with “AP” represent other previously described EVGs (33). In panels B and C, *B*_g_ represents the percentage of OBV-EVG genes that had orthologous relationships (bidirectional best hits of BLASTp; E value, <1e−5) with the genome in the comparison. *C*_g_ corresponds to the percentage of genes in each OBV-EVG that are present in colinearity regions as determined by MCScanX with default settings. These dot plots are sorted by *B*_g_ (from upper left to bottom right on three pages). All tBLASTx alignments are shown. The sequences in the dot plots are circularly permuted and/or reversed for clarity. Grid lines are drawn for every 10 kb, and bold grid lines indicate 50-kb intervals. The color scale represents tBLASTx percent identity. Download FIG S2, PDF file, 1.5 MB. Copyright © 2017 Nishimura et al. 2017 Nishimura et al. This content is distributed under the terms of the Creative Commons Attribution 4.0 International license.

### SNPs and nucleotide diversity.

Each of the individual EVGs likely corresponds to genomes of closely related viruses because the sequence assemblies were obtained from environmental viral populations. To assess the genetic diversity of each EVG, we analyzed single nucleotide polymorphisms (SNPs) and calculated the nucleotide diversity of each EVG. Nucleotide sites containing SNPs that were supported by at least one read were present in genomes at a rate of 0.558 to 7.897% (median, 2.473%) (see Table S1A in the supplemental material). The nucleotide diversity of EVGs was 0.073 to 1.734% (median, 0.423%). These results are within the ranges for genomes from the same viral species (38). We conclude that each of the EVGs represents a consensus genome of a viral species.

**FIG 1:**
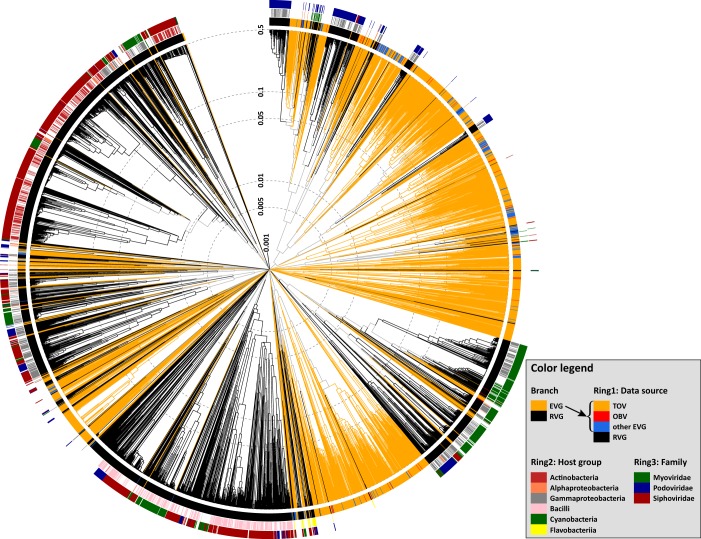
Proteomic tree. The dendrogram represents proteome-wide similarity relationships among 4,240 prokaryotic dsDNA virus genomes. Branches are colored orange (EVG, environmental viral genome) or black (RVG, reference viral genome), and branch lengths are indicated using a logarithmic scale. TOV, *Tara* Oceans viromes; OBV, Osaka Bay viromes. The tree is midpoint rooted. Rings outside the dendrogram represent, from inside to outside, sources of genome data, taxonomic groups of known hosts, and viral family classifications.

10.1128/mSphere.00359-16.10 TABLE S1  (A) SNP and nucleotide diversity of 46 OBV-EVGs. (B) List of 4,240 prokaryotic dsDNA virus genomes in the viral proteomic tree (sorted in the order used for Fig. 1; clockwise) and the corresponding genus-level gOTUs. (C) Host predictions of 29 EVGs by genome-wide sequence similarity (*S*_G_). (D) Predicted virion structural and packaging proteins encoded among 58 putative archaeal EVGs. (E) Predicted Fe-S cluster assembly genes and Fe-S-related genes in nine T4-like EVGs. (F) Photosynthetic genes detected in a set of EVGs/RVGs. (G) Gene annotation table of *aceBA* encoding TOV-EVG (TARA_ERS478052_N000008). (H) PCR primer pairs for validating OBV-EVG assemblies. (I) Seed sequences of photosynthetic genes for PSI-BLAST analysis. Download TABLE S1, PDF file, 1.2 MB. Copyright © 2017 Nishimura et al. 2017 Nishimura et al. This content is distributed under the terms of the Creative Commons Attribution 4.0 International license.

### One thousand five hundred genomes assembled from the *Tara* Oceans viromes.

Prompted by the detection of 46 OBV-EVGs in a modest sequencing effort, we applied our genome assembly and complete genome identification protocol to the *Tara* Oceans viromes (TOV), which consist of 43 viromes representing 26 oceanic locations (9). Given the wide geographic areas and seasons covered by these samples and the large volume of sequence data for individual TOV samples (i.e., average, 50 M reads; 2 × 100 bp), we assembled these 43 viromes individually. We obtained 1,554 TOV-EVGs (i.e., circular complete contigs, 10 to 211 kb) with a predicted viral origin. Only 64 were detected as complete in the previously reported original TOV assemblies (9), and 85.6% of the remaining EVGs (i.e., 1,275 EVGs) were detected in the original assemblies as smaller contigs with less than half the size of the contigs in these new assemblies. Clustering on the basis of the nucleotide sequence identity among the OBV-/TOV-EVGs resulted in 1,352 nonredundant complete genomes (i.e., 46 OBV-EVGs and 1,306 TOV-EVGs).

After discarding possible eukaryotic virus genomes, we obtained 1,567 complete genomes that were likely of prokaryotic dsDNA viral origin (45 OBV-EVGs and 1,522 TOV-EVGs; see Materials and Methods). Of these genomes, 1,404 (89.6%) were predicted to encode homologs of tailed-virus hallmark proteins (i.e., terminase large subunits [89.5%], major capsid proteins [34.4%], or portal proteins [60.2%]), suggesting that the genomes were derived from tailed viruses. Of the remaining 163 EVGs, 72 were predicted to encode integrase homologs.

### Diversity of environmental viral genomes.

To investigate the global novelty offered by culture-independent viral genome sequencing efforts, we compiled a set of 1,811 EVGs (>10 kb) composed of the 45 OBV-EVGs, the 1,522 TOV-EVGs, and 244 EVGs from other studies (29, 33, 39). We also compiled a set of 2,429 prokaryotic dsDNA viral genomes (>10 kb) from cultured viruses, which are referred to here as reference viral genomes (RVGs) (Fig. S3; Table S1B).

10.1128/mSphere.00359-16.4 FIG S3  Length and percent G+C content of EVGs and RVGs. Lengths (top panel) and percent G+C content (bottom panel) of 1,811 EVGs and 2,429 RVGs are presented. The EVGs (i.e., 45 OBV-EVGs, 1,522 TOV-EVGs, and 244 other EVGs) were categorized on the basis of data sources. The other EVGs include viral genomes from uvMED (33), cryoconite (29), and SAG (39). Boxes represent the first quartile, median, and third quartile. Dot colors represent the following data sources: OBV (red), TOV (blue), other EVG (green), and RVG (gray). Download FIG S3, PDF file, 0.1 MB. Copyright © 2017 Nishimura et al. 2017 Nishimura et al. This content is distributed under the terms of the Creative Commons Attribution 4.0 International license.

**FIG 2:**
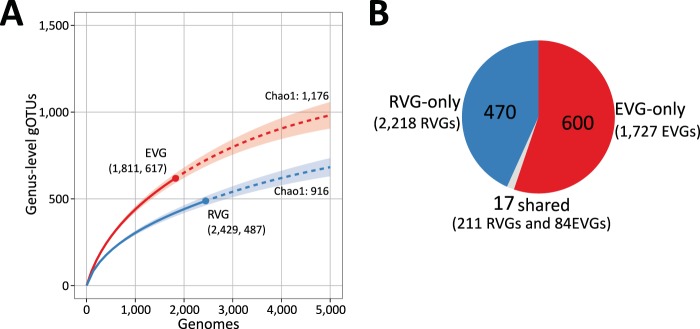
Genus-level genomic OTU (gOTU) richness. The genome-wide similarity score (*S*_G_) cutoff for clustering was set to 0.15 (i.e., viral-genus-level cutoff). The EVGs and RVGs were clustered together, and subsets of the EVGs and RVGs were then constructed by extracting each member. (A) Rarefaction curves for the number of gOTUs. Rarefaction curves are presented with shading representing 95% confidence intervals obtained from 100 bootstrap replicates using the R package iNEXT (107). Dashed curves represent extrapolations to 5,000 genome sequences. Numbers in parentheses represent the number of genomes and gOTUs. Chao1 richness estimates for the EVGs and RVGs are indicated. (B) Proportions of genus-level gOTU clusters. Colors represent the following cluster categories: EVG-only clusters (red), RVG-only clusters (blue), and shared clusters (gray).

We first generated a viral proteomic tree (40) on the basis of genomic similarity scores (denoted by *S*_G_) derived from tBLASTx scores. The *S*_G_ value is 1 when two genomes in a comparison are identical and decreases to 0 when a tBLASTx search fails to detect any sequence similarities. The viral proteomic tree revealed a clear separation between EVG and RVG clades, with most of the EVGs grouped with other EVGs and not with the RVGs (Fig. 1). We also used average linkage clustering of the EVGs/RVGs to delineate operational taxonomic units (i.e., genomic OTUs [gOTUs]) on the basis of the *S*_G_ value, with six different clustering cutoff values (Fig. 2 for cutoff *S*_G_ = 0.15 and Fig. S4 for all cutoff values from 0.1 to 0.9). The EVG-containing gOTUs outnumbered the RVG-containing gOTUs at five of six tested *S*_G_ cutoff values. For example, we observed a 1.6-fold EVG-to-RVG gOTU overrepresentation ratio at *S*_G_ = 0.3 (Fig. S4A). The proteomic tree and comparative genome maps are available at http://www.genome.jp/viptree/EVG2017.

10.1128/mSphere.00359-16.5 FIG S4  Genomic operational taxonomic unit (gOTU) richness with six different thresholds. The EVGs and RVGs were clustered together, and the gOTU subsets of the EVGs and RVGs were then constructed by extracting each member. (A) Rarefaction curves for the number of gOTU clusters. The cutoffs of the genome-wide proteomic similarity score (i.e., *S*_G_) for clustering are indicated in each plot. Numbers in parentheses represent the actual number of genomes and gOTUs. Rarefaction curves are presented with shading representing 95% confidence intervals obtained from 100 bootstrap replicates using the R package iNEXT (107). Dashed curves represent extrapolations to 5,000 genome sequences. Chao1 richness estimates for the EVGs and RVGs are indicated. (B) Proportions of three types of clusters, EVG-only clusters (red), RVG-only clusters (blue), and shared clusters (gray). Proportions determined by the number of gOTU clusters (left) and by the number of genomes (right) are presented. Download FIG S4, PDF file, 0.3 MB. Copyright © 2017 Nishimura et al. 2017 Nishimura et al. This content is distributed under the terms of the Creative Commons Attribution 4.0 International license.

### Genus-level operational taxonomic units.

We analyzed the viral taxonomic classification of the RVGs and evaluated the correspondence between viral genera and gOTUs using different *S*_G_ cutoff values. The *S*_G_ values between 0.07 and 0.2 were associated with relatively high adjusted Rand index values (i.e., > 0.79), and *S*_G_ = 0.15 (adjusted Rand index = 0.847) was determined to be the most accurate cutoff value for a genus-level classification (Fig. S5). With this cutoff value, we obtained 1,087 gOTUs for the EVGs/RVGs. The 2,429 RVGs were distributed across 487 gOTUs, whereas the 1,811 EVGs were distributed across 617 gOTUs (i.e., 1.27-fold-higher richness), with only 1.4% of the total gOTUs containing both EVGs and RVGs (Fig. 2B). Therefore, the EVGs potentially represent 600 new viral genera. Of the 600 gOTUs, 497 were composed exclusively of OBV-/TOV-EVGs. To complement this analysis, we added 11,779 mined viral genomes (MVGs; genome sizes, >10 kb) (12). We observed only a limited overlap of gOTUs among the EVGs, RVGs, and MVGs (i.e., only two gOTUs with sequences from all three sets), and 590 genus-level gOTUs remained specific to the EVGs.

10.1128/mSphere.00359-16.6 FIG S5  Evaluation of *S*_G_ cutoff levels for the delineation of genus-level gOTUs. The correspondence between the genus-level classification of known viruses and their grouping into gOTUs was evaluated using the adjusted Rand index for various *S*_G_ cutoff values (see Materials and Methods). Numbers in parentheses represent the *S*_G_ value (0.15) where the adjusted Rand index reached the highest value (0.847). Download FIG S5, PDF file, 0.1 MB. Copyright © 2017 Nishimura et al. 2017 Nishimura et al. This content is distributed under the terms of the Creative Commons Attribution 4.0 International license.

### Virus-host interactions. (i) Host prediction on the basis of genomic similarity.

Because of the dissimilarity between EVGs and RVGs, host predictions on the basis of similarities to known viral genomes (i.e., RVGs) were difficult to make. Using information regarding RVG hosts, we calculated an optimal *S*_G_ threshold that separated viruses into those that infect similar hosts and those that do not. The threshold was a *S*_G_ value of >0.2937 (>90% precision) for the prediction of pairs of viruses infecting host organisms that are evolutionarily related at the genus level (Fig. S6). With this cutoff, we predicted host groups for only 29 of 1,811 EVGs (2 OBV-EVGs, 13 TOV-EVGs, and 14 other EVGs; Table S1C). Of the 29 EVGs, 18, 10, and 1 were predicted to be cyanophages, *Pelagibacter* phages, and *Pseudoalteromonas* phages, respectively. Two additional host prediction methods based on tRNA genes and clustered regularly interspaced short palindromic repeat (CRISPR) spacer sequences (41) failed to predict possible hosts for the EVGs. However, the physical linkage of genes on the EVGs provided additional clues about their hosts and biology. In the following sections, we describe virus-host interactions inferred from the genomic contexts of EVGs.

10.1128/mSphere.00359-16.7 FIG S6  Evaluation of host group predictions. A precision curve for predictions of host taxonomic groups (mainly at the genus level, except for *Cyanobacteria* and *Enterobacteriaceae*; see Materials and Methods) was generated using the genome-wide proteomic similarity score (i.e., *S*_G_). We estimated the precision of host group predictions for a subset of the RVGs used in this study (i.e., 2,429 prokaryotic dsDNA viruses), each of which was uniquely assigned to the host taxonomic group. For each RVG, the best *S*_G_ values for the members of the same host taxonomic group (i.e., mostly the same genus), and for the different host taxonomic groups, were recorded (i.e., 2,570 *S*_G_ scores). The precision curve was generated using sliding *S*_G_ cutoff values. Download FIG S6, PDF file, 0.1 MB. Copyright © 2017 Nishimura et al. 2017 Nishimura et al. This content is distributed under the terms of the Creative Commons Attribution 4.0 International license.

### (ii) MGII viruses.

**FIG 3:**
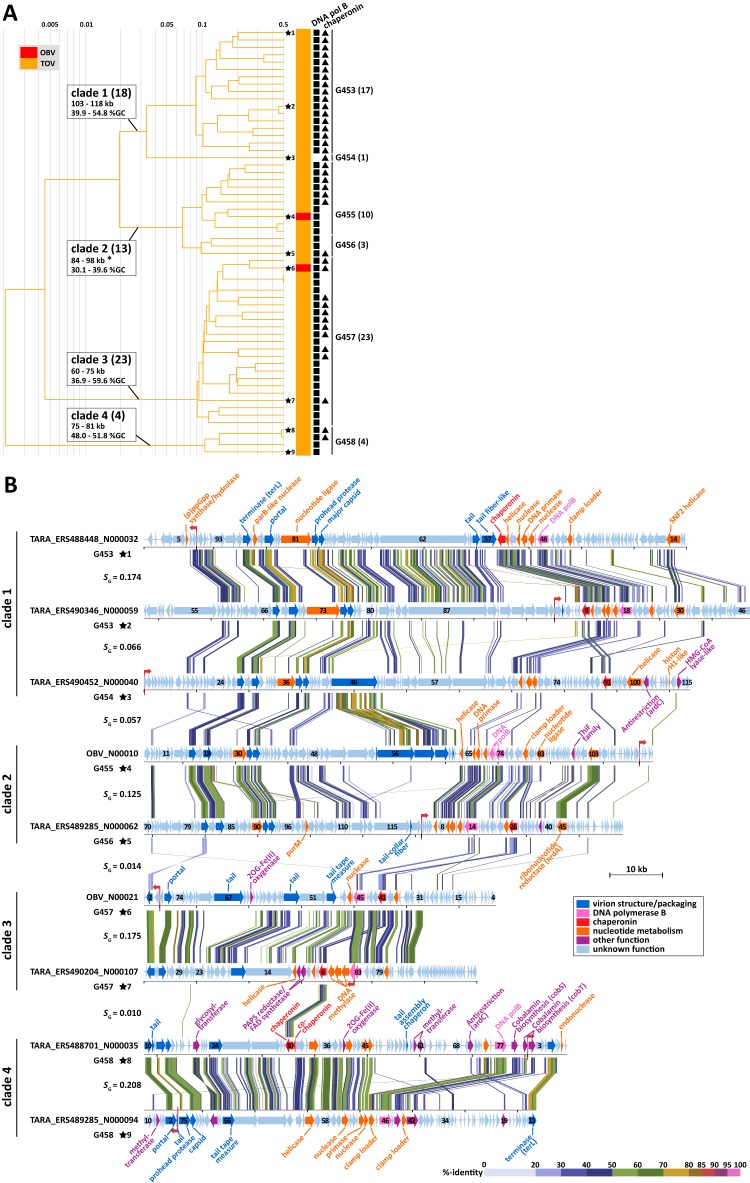
Fifty-eight putative archaeal virus genomes. (A) Part of the proteomic tree with 2 OBV-EVGs (red) and 56 TOV-EVGs (orange), predicted to be derived from euryarchaeal tailed viruses infecting marine group II (MGII) species. Genomes with genes encoding DNA polymerase B (squares) and chaperonin (triangles) are indicated. Clade names and genus-level gOTUs are indicated. Numbers in parentheses represent the number of genomes of each clade or gOTU. The ranges of genome sizes and percent G+C contents for each clade are presented, with the exception that clade 2 includes a long contig (121 kb; asterisk). Branch lengths are logarithmically scaled from the root of the entire proteomic tree in Fig. 1. (B) Genome map of nine archaeal viral genomes that are indicated by stars in panel A. The sequences are circularly permuted and/or reversed. Red arrows indicate the original start position of the sequences. Putative gene functions are indicated. All tBLASTx alignments are represented by colored lines between the two genomes. The color scale represents tBLASTx percent identity.

**FIG 4:**
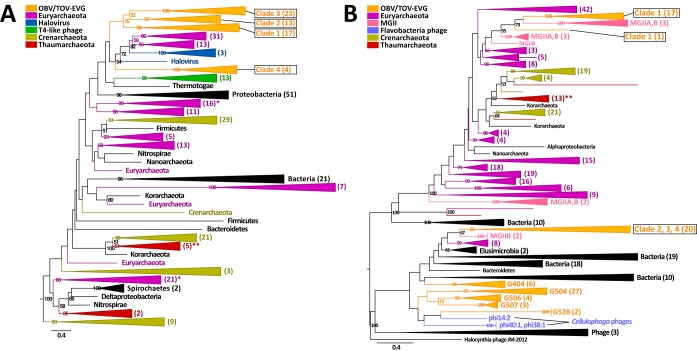
Gene phylogenetic trees of DNA polymerase B and chaperonin. (A) Maximum likelihood tree of DNA polymerase B. The tree is rooted by four distant bacterial sequences (not shown) and includes 348 sequences. (B) Maximum likelihood tree of chaperonin. The tree is midpoint rooted and includes 381 sequences. In panels A and B, numbers in parentheses represent the number of sequences in each collapsed node. Colors represent taxonomies. Asterisks indicate collapsed nodes that include MGII (*) and MGI (**) sequences. The scale bar refers to the estimated number of amino acid substitutions per site. Numbers near the nodes represent bootstrap percentages of >50%. MGIIA and MGIIB indicate sequences from reported genomes (45 and 46, respectively).

Four previously undescribed lineages that likely infect unculturable marine group II (MGII) *Euryarchaeota* species were revealed in the proteomic tree. These four clades were exclusively composed of OBV/TOV-EVGs, with 18, 13, 23, and 4 EVGs in clades 1, 2, 3, and 4, respectively (Fig. 3). Phylogenetic analyses of the DNA polymerases encoded in those EVGs strongly support the existence of the four clades identified in the proteomic tree (Fig. 4A). These clades were grouped with homologs from haloviruses and euryarchaea. Identifications of archaeal hosts for the 58 EVGs were also supported by their gene content. Of the genes in the EVGs with homologs in cellular organisms, an average of 36.1% (14.3 to 60.0%) were most closely matched to archaeal proteins. Additionally, one to five tailed-virus structural protein homologs were detected in each of the EVGs (Table S1D). Archaeal tailed viruses have been detected only in *Euryarchaeota* species (42), with the exception of a provirus of *Nitrososphaera* *viennensis* (*Thaumarchaeota*) isolated from soil (43).

We observed that the EVGs contained chaperonin genes (Fig. 3A). Thirty-eight of the 58 EVGs encode chaperonin homologs, even though chaperonin genes have rarely been identified in sequenced viral genomes (i.e., only 7 of the 2,429 RVGs encode chaperonins). In some viruses, chaperonins, which are usually provided by the hosts, are responsible for the correct assembly of viral particles (44). All 18 EVGs in clade 1 encode archaeon-type chaperonin homologs (i.e., thermosome; group II chaperonin), while 20 EVGs in clades 2 to 4 encode bacterium-type chaperonin homologs (i.e., GroEL; group I chaperonin). We detected both groups of chaperonin genes in the MGII genomes (45, 46). The group I and group II chaperonin sequences from the EVGs were grouped with these MGII chaperonins (Fig. 4B), suggesting that MGII species serve as hosts for these environmental viruses.

The following three archaeal taxa are abundant in the marine water column: marine group I *Thaumarchaeota* (MGI), MGII, and marine group III *Euryarchaeota* (MGIII) (47). Of these, currently cultivated representatives exist only in MGI (48). The members of MGII are abundant in particle-rich surface waters (49, 50), while those of MGIII have been observed almost exclusively in deep seas (47). A recent study revealed that MGII members can temporarily become the most abundant (up to 40%) prokaryotic components in the days following a spring bloom (51). The 58 EVGs were derived from surface or deep chlorophyll maximum viromes, suggesting their photic-zone habitat. These observations and the genomic context described above suggest that the 58 EVGs represent genomes of tailed viruses infecting MGII *Euryarchaeota* species.

### (iii) A SAR86 phage encoding IscU.

**FIG 5:**
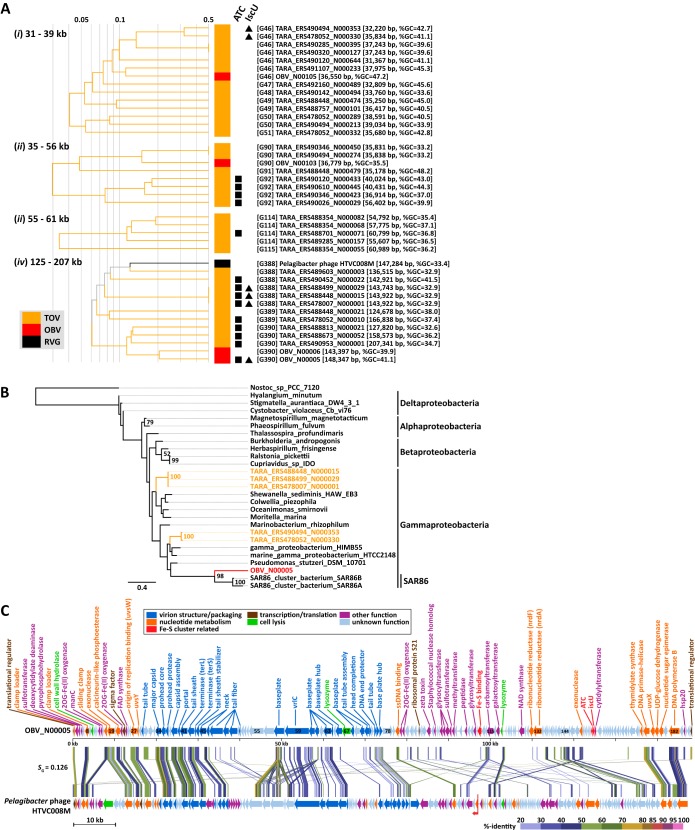
Genomes with Fe-S cluster assembly-related genes. (A) Four parts of the proteomic tree with genomes carrying Fe-S cluster assembly genes (i.e., ATC [■] and IscU [▲] genes). Branch lengths are logarithmically scaled as described for Fig. 3A. Genus-level gOTUs and genome identifiers (IDs), lengths, and percent G+C compositions are indicated. (B) Maximum likelihood tree of IscU genes. The tree contains protein sequences encoded in OBV_N00005 (red), five TOV-EVGs (orange), and 21 *Proteobacteria* and cyanobacterial genomes (black). The scale bar refers to the estimated number of amino acid substitutions per site. Numbers close to the nodes represent bootstrap percentages of >50%. The tree is rooted by the cyanobacterial *Nostoc* species sequence. (C) Genome map of OBV_N00085 and *Pelagibacter* phage HTVC008M. The HTVC008M sequence is circularly permuted at 97,000 bp and reversed. A red arrow indicates the original start position of the HTVC008M sequence. Putative gene functions of OBV_N00005 and HTVC008M (described in reference 10) are indicated. All tBLASTx alignments are represented by colored lines between the two genomes. The color scale represents tBLASTx percent identity. FAD, flavin adenine dinucleotide; NAD, nicotinamide adenine dinucleotide.

Iron-sulfur (Fe-S) cluster proteins are involved in a variety of biological processes, including gene regulation, electron transfer, catalytic reactions, and oxygen-iron sensing (52). In a previous study, Fe-S cluster assembly protein genes (e.g., *sufA* and *iscU*) were identified as auxiliary metabolic genes (AMGs) of photic-zone viromes (15, 53). However, the lack of complete genome data hindered further characterizations of the viruses carrying these genes. We identified 16 OBV/TOV-EVGs with Fe-S cluster assembly protein genes, including 14 EVGs containing an Fe-S cluster A-type carrier (ATC) gene (54) and 6 EVGs carrying the IscU gene (Fig. 5A). These genomes are scattered across four groups of viruses in the proteomic tree, and many of their close relatives (i.e., other EVGs and *Pelagibacter* phage HTVC008M in Fig. 5A) do not contain these genes. The ATC and IscU proteins function as scaffolds in which Fe and S atoms are assembled into Fe-S clusters (55, 56). Phylogenetic trees of IscU (Fig. 5B) and ATC (Fig. S7) revealed that all six EVG-encoded IscU genes form a clade with gammaproteobacterial homologs. Of these, an IscU gene from OBV_N00005 was phylogenetically closely related to homologs from SAR86 (57), suggesting that SAR86 members represent potential hosts for OBV_N00005. The prevalence of these viral genes in photic-zone viromes (15) appears to be linked to the wide distribution of these bacteria.

In addition to the Fe-S scaffolding proteins, some of the EVGs encode several Fe-S cluster proteins that use Fe-S clusters as prosthetic groups, such as radical S-adenosylmethionine (SAM) superfamily enzymes (58) and CRISPR-associated Cas4 exonucleases (59, 60). The EVGs also encode proteins involved in the metabolism of Fe-S cluster proteins, such as glutaredoxins (Grx), the phenylacetyl-coenzyme A oxygenase component PaaD (61, 62), and ClpP, which is a serine protease targeting Fe-S cluster proteins (15). A notable example is the T4-like TARA_ERS488813_N000010 (183 kb; group *iv* in Fig. 5A), which includes an ATC gene, 12 genes for radical SAM superfamily enzymes, and *cas4*, *grx*, and *paaD* (16 genes in total; Table S1E). Other T4-like EVGs encoding ATC and/or IscU proteins contain two to seven additional Fe-S-related genes. Of these genes, *paaD* has not been previously associated with a virally encoded protein and thus represents a novel AMG. These observations suggest that Fe-S cluster assembly proteins encoded in these viral genomes function as a part of Fe-S cluster-related metabolic processes involving not only host proteins but also many virally encoded proteins.

10.1128/mSphere.00359-16.8 FIG S7  Maximum likelihood tree of ATC genes. The tree includes OBV_N00005 (red), 13 TOV-EVGs (orange), and 58 published reference sequences (54). The reference sequences are colored as follows: alphaproteobacteria and mitochondria (brown), betaproteobacteria (cyan), gammaproteobacteria (blue), and others (black). The group numbers (i.e., *ii*, *iii*, and *iv*) correspond to the groups of EVGs described in the Fig. 5A legend. Subfamilies of ATCs (i.e., ATC-I, ATC-II, and ATC-III) correspond to those described in reference 54. *Escherichia coli* ErpA, IscA, and SufA are indicated. The tree is midpoint rooted. The scale bar refers to the estimated number of amino acid substitutions per site. Numbers close to the nodes represent bootstrap percentages of >50%. Download FIG S7, PDF file, 0.3 MB. Copyright © 2017 Nishimura et al. 2017 Nishimura et al. This content is distributed under the terms of the Creative Commons Attribution 4.0 International license.

### (iv) A novel cyanophage lineage.

The RVG set included 114 cyanophage genomes, which were grouped into 17 viral-genus-level gOTUs. There were no other RVGs classified into these gOTUs. Of these 17 gOTUs, 5 included 34 EVGs (i.e., 3 OBV-EVGs, 16 TOV-EVGs, and 15 previously described EVGs [33]), which are likely to have been derived from cyanophages or their relatives. Screening all EVGs with 11 photosynthesis-related AMGs (see Materials and Methods) led to the identification of 11 predicted cyanophage EVGs, of which 10 were included in the gOTUs mentioned above (Table S1F). The remaining EVG (i.e., TARA_ERS489084_N000023; gOTU G241), which carries *psbA* and *hli*, formed a singleton gOTU and represents a new cyanophage group. To characterize the approximate abundances of these 18 cyanophage gOTUs (149 genomes; Table 2), we mapped the TOV and OBV reads on these putative cyanophage genomes. The following five most abundant gOTUs represented >98% of the total cyanophage content: (i) G386, including T4-like myoviruses (35.1%); (ii) G14, including podoviruses (33.7%); (iii) G234, including a siphovirus and dwarf myoviruses (23.4%); (iv) G238, including *Synechococcus* phage S-EIV1 (63) (3.3%); and (v) G15, including *Prochlorococcus* phage P-RSP2 (3.2%) (Tables 2 and S1B for the list of genomes). Thus, marine cyanophage genomes are well represented in the current databases.

**TABLE 2:** Photosynthetic genes and abundance of cyanophage genomes

Genus-level gOTU	No. of EVGs	No. of RVGs	Photosynthetic gene(s) in EVG	Photosynthetic gene(s) in RVG	FPKM^a^	% abundance^b^	Most abundant RVG
G14	7	21	*hli*, *psbA*	*hli*, *psbA*	3,484.7	**33.7**	*Prochlorococcus* phage P-GSP1
G15	1	1	*hli*		334.6	**3.2**	*Prochlorococcus* phage P-RSP2
G234	16	3			2,419.5	**23.4**	Cyanophage MED4-117
G237	1	1			35	0.3	*Synechococcus* phage S-CBS4
G238	6	1	*hli*, *ptoX*		340.7	**3.3**	*Synechococcus* phage S-EIV1
G241	1	0	*hli*, *psbA*		48.6	0.5	
G242	1	1	*hli*, *psbA*	*hli*	10.5	0.1	*Synechococcus* phage S-CBS2
G243	0	1			3.3	0	Cyanophage P-SS2
G277	0	1		*nblA*	0	0	*Planktothrix* phage PaV-LD
G278	0	2		*nblA*	1.8	0	*Microcystis aeruginosa* phage Ma-LMM01
G386	2	72	*cpeT*, *hli*, *petE*, *psbA*, *psbD*, *ptoX*	*cpeT*, *hli*, *ho1*, *pcyA*, *pebS*, *petE*, *petF*, *psbA*, *psbD*, *ptoX*	3,622.7	**35.1**	*Synechococcus* phage S-SM2
G387	0	1		*hli*, *psbA*	0.1	0	*Synechococcus* phage S-CRM01
G402	0	1		*hli*, *petE*, *psbA*, *psbD*, *ptoX*	21.8	0.2	Cyanophage S-TIM5
G769	0	2			0	0	Cyanophage PP
G770	0	1			0	0	*Anabaena* phage A-4L
G771	0	1			0	0	*Phormidium* phage Pf-WMP4
G818	0	2		*nblA*	0	0	*Phormidium* phage MIS-PhV1B
G1074	0	2			2.1	0	*Synechococcus* phage S-CBS1

a The FPKM for each gOTU was calculated as the average of the sum of FPKMs of the genomes in the gOTU across different samples. In calculating the average, the nine OBV samples were treated as a single sample to avoid any bias toward a local region.

b Abundance represents a normalized FPKM (the sum is equal to 100), and values of >3% are indicated in bold.

### (v) Diverse marine *Bacteroidetes* phages.

**FIG 6:**
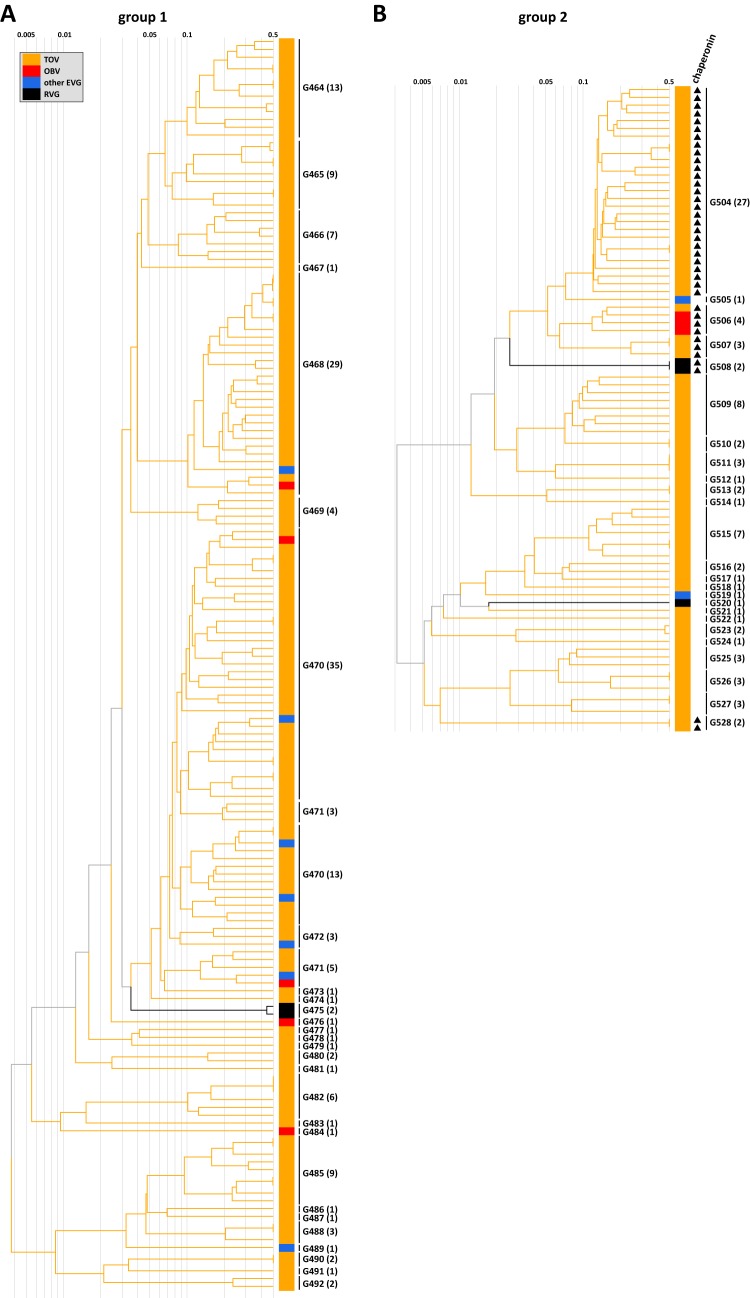
Two parts of the proteomic tree with EVGs of putative *Flavobacteriaceae* phages. Branch lengths are logarithmically scaled as described for Fig. 3A. Genus-level gOTUs are indicated. Numbers in parentheses represent the number of genomes in each gOTU. (A) Group 1 distributed in 29 gOTUs, including two *Persicivirga* phages (black), 5 OBV-EVGs (red), 147 TOV-EVGs (orange), and 7 other EVGs (blue). (B) Group 2 distributed in 25 genus-level gOTUs, including two *Cellulophaga* phages (phi40:1 and phi38:1; black; G508), IAS virus (black; G520), 3 OBV-EVGs (red), 75 TOV-EVGs (orange), and 2 other EVGs (blue). Genomes encoding chaperonins are indicated by a triangle.

*Bacteroidetes* is one of the most abundant bacterial phyla in the oceans (e.g., 30% of the bacterioplankton during phytoplankton blooms) (64). Members of this phylum are involved in the decomposition and remineralization of phytoplankton biomass (65). A recent study revealed that an algal bloom is followed by the presence of a rapid succession of diverse *Flavobacteriaceae* bacteria (64). To the best of our knowledge, the genomes of the following 38 phages infecting marine *Bacteroidetes* (*Flavobacteriaceae*) have been described: psychrophilic* Flavobacterium* phage 11b (66), *Croceibacter* phage P2559S (67), 2* Persicivirga* phages (68), 31 *Cellulophaga* phages (69), *Flavobacterium* phage 1/32 (70), and 2 *Polaribacter* phages (71). *Polaribacter* was reported to be abundant following a spring phytoplankton bloom (64), while *Cellulophaga* phages (31 of 38) likely represent a “rare biosphere” rather than abundant marine phages (69). We detected two groups (i.e., groups 1 and 2) of putative *Flavobacteriaceae* phage genomes (i.e., 5 RVGs, 8 OBV-EVGs, 222 TOV-EVGs, and 9 EVGs from another study; Fig. 6). Group 1 and group 2 consisted of 29 and 25 gOTUs, respectively. Of these, 23 and 21 gOTUs were exclusively composed of OBV/TOV-EVGs. Of the genes in the OBV/TOV-EVGs having homologs in cellular organisms, 64.4% (15.8% to 92.3%) on average for the members of group 1 and 32.4% (10.5% to 59.1%) on average for the members of group 2 were most similar to *Bacteroidetes* genes. For example, the gene20 sequence of OBV_N00025 (group 2, G506; Fig. 6B) was most similar to the RNA polymerase sigma-70 factor sequence of a *Flavobacteria* strain from marine surface water (WP_009781949; *Leeuwenhoekiella blandensis*; E value = 1e-30) (72, 73). Genomes of these groups also encode conserved virion structural or morphogenetic proteins. For the members of group 1, we detected putative portal gene homologs in 148 EVGs (93.7%) and prohead protease homologs in 145 EVGs (91.8%). For the members of group 2, we detected homologs of two to six structural proteins of *Cellulophaga* phage phi38:1 (i.e., a member of group 2) in 78 EVGs (100%). Additionally, we detected GroEL homologs in 36 EVGs of the members of group 2 (Fig. 6B) which were phylogenetically related to the homologs in *Cellulophaga* phages (Fig. 4B). Therefore, these EVGs probably correspond to viruses of *Flavobacteriaceae* species and may prove to be useful genetic markers for studying viruses affecting bacterial decomposer communities.

### (vi) A virus potentially enhancing the adaptation of its host.

Isocitrate lyase (AceA) and malate synthase (AceB) catalyze two reactions in the glyoxylate shunt, which bypasses the CO_2_-generating steps of the tricarboxylic acid cycle and enables the net assimilation of carbon from acetyl-coenzyme A (acetyl-CoA), leading to gluconeogenesis (i.e., generation of glucose) and cell growth (74, 75). We identified an *aceBA* operon in a TOV-EVG (TARA_ERS478052_N000008; 179 kb; see Table S1G for gene description) that included homologs of three structural genes from T4-like phages. Our genomic similarity and gene composition analysis did not provide any clue about the host of this virus. A previous study detected *aceA* and *aceB* in ocean viromes (14), but this is the first time, to our knowledge, that an *aceBA* operon has been observed in a complete viral genome. The genome also encoded six enzymes (i.e., Gmd, WcaG, ManC, NeuA, KdsA, and WaaG) for the biosynthesis of lipopolysaccharides (LPS) and capsular polysaccharides, important components of bacterial cell wall and capsule (76, 77). Previous studies identified LPS synthesis genes in temperate and lytic phages and proposed that these genes function to modify cell surface compositions to prevent other viruses from attaching to the cell during the lysogenic or pseudolysogenic phase, in the latter of which a lytic process is halted due to suboptimal host cell growth (78, 79). Following this “lock out” hypothesis, the *aceBA*-carrying virus (i.e., TARA_ERS478052_N000008) should have a provirus phase, and AceA and AceB may function to promote the growth of host cells. gene40 of the TOV-EVG was predicted to encode a homolog of zeta toxin proteins (Table S1G) thought to be involved in a toxin-antitoxin system. Toxin-antitoxin systems enhance the stability of plasmids and prophages by postsegregational killing (80). This corroborates the existence of a lysogenic phase of this virus, though there was no other evidence for lysogeny in the viral genome. It should be further noted that the function of LPS is not limited to protection of the cell from viral infection but that LPS on bacterial outer membrane confers tolerance of temperature and oxidative stresses as well as resistance to antibiotics (81). Therefore, *aceBA* and the cell wall biogenesis genes in the TOV-EVG may contribute to a host’s survival and environmental adaptation by altering carbon metabolism and cell surface compositions during the lysogenic phase.

### (vii) Temperate phages of SAR116.

**FIG 7:**
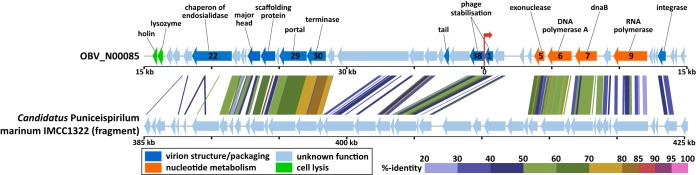
Genomic alignment between the whole sequence of OBV_N00085 and a genomic region (385,000 to 425,000 bp) of “*Candidatus* Puniceispirillum marinum” IMCC1322. The OBV_N00085 sequence is circularly permuted at 15,000 bp for clarity, and a red arrow indicates the original start position of the sequence. Putative gene functions and function categories of OBV_N00085 are indicated by texts and colors. All tBLASTx alignments are presented. The color scale represents tBLASTx percent identity.

Our analysis also unveiled phage genomes likely infecting members of the SAR116 clade, which is one of the most abundant marine bacterial lineages (11). OBV_N00085 (40 kb) and three closely related TOV-EVGs (40 to 41 kb; *S*_G_ for OBV_N00085 = 0.25 to 0.26) exhibited clear collinearity with an approximately 40-kb genomic segment from “*Candidatus* Puniceispirillum marinum” IMCC1322 of the SAR116 clade (class: *Alphaproteobacteria*) (Fig. 7 for OBV_N00085) (82). This suggests that these EVGs are derived from temperate phages infecting SAR116 or related bacteria. These genomes consistently encode integrases.

### (viii) Phages related to SAR11 phages.

Seven EVGs (OBV_N00073, three TOV-EVGs, and three other EVGs; 39 to 42 kb) exhibited high genome-wide sequence similarities to *Pelagibacter* podovirus HTVC019P (10) (*S*_G_ = 0.34 to 0.44; 42 kb; a dot plot comparing OBV_N00073 and HTVC019P is presented in Fig. S2B). On the basis of the *S*_G_ values (i.e., >0.2937; estimated precision, >90%), we predict that these EVGs infect host species in the genus *Pelagibacter* (Table S1C). Another *Pelagibacter* podovirus (i.e., HTVC010P), which is believed to be a member of the most abundant virus subfamily in the biosphere (10), was classified in a different group of the proteomic tree together with 102 EVGs (OBV_N00107, 77 TOV-EVGs, and 24 other EVGs; 31 to 73 kb; Fig. S8). These 102 genomes carry homologs of HTVC010P structural protein genes. The G+C content of the HTVC010P genome is 32% (10), while the EVGs of this group contain higher levels of G+C content (i.e., 31 to 57%). Low levels of G+C content (i.e., 28.6 to 32.3%) are a common genomic feature of the SAR11 clade members (83). Since high levels of correlation between the G+C content of prokaryotic viruses and that of their hosts were previously reported (84, 85), the variation in the levels of their G+C content suggests that the viruses in this group infect a wide range of host species.

10.1128/mSphere.00359-16.9 FIG S8  Part of the proteomic tree with *Pelagibacter* podovirus HTVC019P (black), OBV_N00107 (red), 83 TOV-EVGs (orange), and 25 other EVGs (blue). Branch lengths are logarithmically scaled as described for Fig. 3A. Genus-level gOTUs are indicated. Numbers in parentheses represent the number of genomes in each gOTU. Download FIG S8, PDF file, 0.3 MB. Copyright © 2017 Nishimura et al. 2017 Nishimura et al. This content is distributed under the terms of the Creative Commons Attribution 4.0 International license.

### Environmental viral genomes as a reference during marine virome analyses.

**FIG 8:**
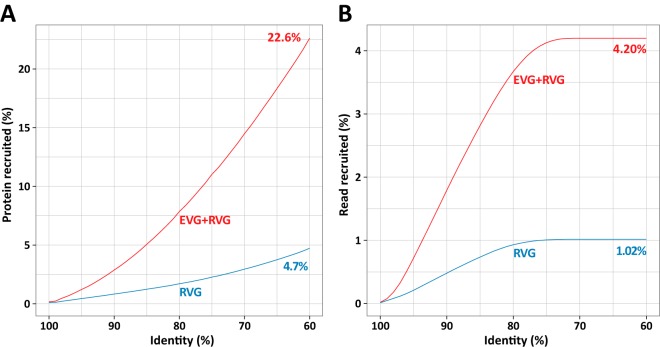
Recruitment of photic POV sequences to RVGs (blue) and to a pool of EVGs and RVGs (red). Mappings were performed with tBLASTn (proteins) and BLASTn (reads). In both mappings, the initial filtering of hits involved an E value of <1e−3, and an additional filtering was based on ≥60% identity and ≥80% alignment length of the query sequence. (A) Recruitment of proteins. (B) Recruitment of reads.

We mapped protein sequences and raw reads from independently generated photic virome data (i.e., the Pacific Ocean viromes [POV]) (86) on the RVGs and EVGs. The RVG set recruited 4.70% of the POV proteins, while the EVG/RVG union set recruited 22.6% of the proteins (i.e., a 4.8-fold increase; Fig. 8A). At the nucleotide sequence level, the RVG set recruited 1.02% of the POV reads, while the EVG/RVG union set recruited 4.20% of the reads (i.e., a 4.1-fold increase; Fig. 8B). Thus, the EVGs serve as an effective additional reference viral genome data set for exploring viromes from photic oceans.

### Conclusion.

From the assemblies of 52 marine viromes, we obtained 1,567 circular complete genomes that are most likely of prokaryotic dsDNA viral origin. The acquisition of the complete genome sequences helped classify the viral lineages and provided important clues about their hosts and metabolisms. The genome-based clustering of the metagenome-derived viral genomes together with previously reported ones suggests that 600 of the 617 gOTUs represent new genera of prokaryotic viruses. Additionally, they contain greater genome richness than the reference genomes of cultured prokaryotic viruses that have so far been sequenced. Our analyses also predicted the relationships among the EVGs and the major groups of marine prokaryotes, for which no viruses have been isolated (i.e., MGII and SAR86). Given the lack of isolation of viruses, the physiological features of the sequenced EVGs are unclear. However, some of the newly identified EVGs carried functionally related AMGs, such as those encoding proteins related to Fe-S clusters (16 genes) and to carbon assimilation/cell wall biogenesis enzymes (8 genes). These AMGs may function to coordinate the supply/recycling of Fe-S clusters and to enhance host adaptation during the lysogenic cycle. Previous studies also revealed that cyanophages carry multiple functionally linked photosynthesis and lipopolysaccharide synthesis genes for their efficient replication (79, 87, 88). Therefore, viral survival strategies in marine viruses involving many functionally related AMGs appear to target not only the biosynthesis of molecular building blocks (e.g., nucleotides) but also diverse metabolic and cellular processes.

## MATERIALS AND METHODS

### Sample preparation and sequencing.

Seawater samples (9 × 4 liters) were collected at a 5-m depth at the entrance of Osaka Bay (34°19′28″N, 135°7′15″E), Japan, every 3 h for 24 h on 25 and 26 August 2014. Seawater was filtered through a 142-mm-diameter (3.0-μm-pore-size) polycarbonate membrane (Millipore, Billerica, MA) and then through a 142-mm-diameter (0.22-μm-pore-size) Durapore polyvinylidene fluoride membrane (Millipore). The filtrates were stored at 4°C prior to treatments. The viruses in the filtrate were concentrated by FeCl_3_ precipitation (89) and purified using DNase and a CsCl density centrifugation step (90). The DNA was then extracted as previously described (91). Libraries were prepared using a Nextera XT DNA sample preparation kit (Illumina, San Diego, CA) according to the manufacturer’s protocol, except that we used 0.25 ng viral DNA. Samples were sequenced with a MiSeq sequencing system and MiSeq V2 (2 × 150 bp; five samples) or V3 (2 × 300 bp; four samples) reagent kits (Illumina, San Diego, CA).

### Genome assembly and error estimation.

Nine OBVs were individually assembled using the following four assemblers: SPAdes, metaSPAdes (http://bioinf.spbau.ru/spades), IDBA-UD, and Ray Meta. SPAdes 3.1.1 was used with default k-mer lengths as well as the accompanying BayesHammer (92) and MismatchCorrector. The metaSPAdes 3.7.0 program was used with default k-mer lengths and BayesHammer. The IDBA-UD 1.1.1 program was used with fixed multiple k-mer lengths (24 to 124, increased by 10 for 2 × 300 bp reads; 24 to 84, increased by 10 for 2 × 150 bp reads) and the option of a preread correction with a minimum contig length of 300 bp. Ray Meta 2.3.1 was used with a fixed k-mer length (*k* = 41). Additionally, we used scaffolds of these assemblies, which we called contigs for simplicity. The REAPR 1.0.18 program was used to assess the quality of the assemblies. This program reports four types of errors categorized as short insertion/deletion errors (i.e., types 1 and 3) or scaffolding errors (i.e., types 2 and 4).

Nine sets of OBV reads were also coassembled by SPAdes with the same settings as described above. We determined that a contig was circular (i.e., complete) if its 5′ and 3′ terminal regions were nearly identical (i.e., >94% and ≥50 bp). We identified 40 circular contigs (>10 kb) satisfying this condition. A coassembly involving the merged paired-end reads generated by FLASH was also prepared (93). We included the merged and remaining unmerged reads for the assembly. With this second coassembly, we detected 34 circular contigs (>10 kb), of which 6 were not detected in the first coassembly. We incorporated these 6 contigs in our data set, and we ultimately obtained 934 OBV contigs (>10 kb), including 46 circular ones. Forty-three TOV samples were similarly analyzed, except that the sequence assemblies were prepared sample by sample and only with raw reads (i.e., not from merged paired-end reads). Code for circular contig detection is downloadable at ftp://ftp.genome.jp/pub/db/community/EVG2017.

### Gene prediction and annotation.

Gene predictions were completed using MetaGeneMark (94). Homology searches were conducted using BLASTp against the NCBI-nr database (E value, <1e−5), RPS-BLAST against the COG database (as of April 2015; E value, <1e−4), and HMMER against the Pfam (as of May 2015; E value, <1e−4) and TIGRFAMs (release 15; E value, <1e−4) databases. For predictions of tailed-virus hallmark genes and integrase genes, we used HHsearch (E value, <1e−9) against the Pfam database after constructing query hidden Markov models (HMMs) using jackhmmer (part of the HMMER package) with default settings (95, 96). We also used PSI-BLAST to identify homologs of specific genes.

### Discrimination of viral and prokaryotic contigs and PCR assays.

We used a newly developed method (see Text S1 in the supplemental material) and VirSorter (97) to distinguish between viral and prokaryotic contigs. We discarded all contigs predicted to be of prokaryotic origin by either or both methods. Finally, 879 of the 934 OBV contigs (including 46 circular ones) and 1,554 of the 1,618 TOV circular contigs were considered to originate from viruses.

10.1128/mSphere.00359-16.1 TEXT S1  New method for discriminating between viral and prokaryotic contigs. Download TEXT S1, DOCX file, 0.05 MB. Copyright © 2017 Nishimura et al. 2017 Nishimura et al. This content is distributed under the terms of the Creative Commons Attribution 4.0 International license.

We conducted PCR assays for 21 weakly supported regions in four randomly selected OBV circular contigs (i.e., OBV_N00005, OBV_N00020, OBV_N00021, and OBV_N00023; see Fig. S2A in the supplemental material). Primer sequences are provided in Table S1H in the supplemental material.

### Genomic colinearity.

Colinearity was evaluated on the basis of the percentage of OBV-EVG genes that had orthologous relationships with the most closely related genome (i.e., *B*_g_ in Fig. S2B). If ≥60% of the OBV-EVG genes had orthologs in the closest relative, we considered the OBV-EVG to exhibit nearly complete genomic colinearity. Eighteen OBV-EVGs (39%) were observed to exhibit complete or nearly complete colinearity with other viral genomes. Additionally, we identified colinear genomic regions using MCScanX (98) and calculated the percentage of OBV-EVG genes in these regions (i.e., *C*_g_ in Fig. S2B).

### Quality control of reads.

We used raw reads for the above assemblies, but the reads underwent a quality-control screening before being back-mapped to contigs with the following procedure: (i) duplicated reads were removed using FastUniq (99); (ii) paired-end reads were merged with FLASH, and the merged and unmerged reads were kept; (iii) reads were removed if the percentage of high-quality nucleotide positions (i.e., quality score >30) was <80%; and (iv) reads were removed if the sum of the lengths of ambiguous nucleotide positions and low-complexity regions detected by DUST was >40% of the total length. If one of the paired-end reads was removed in step iii or step iv, the mate was retained as a single read.

### Detection of single nucleotide polymorphisms and calculation of nucleotide diversity.

To detect SNPs and assess nucleotide diversity, we mapped quality-controlled reads on contigs using the Bowtie 2 program. To minimize the inclusion of sequencing errors among the mapped nucleotides, we considered only high-quality nucleotides (i.e., quality score, >30). Nucleotide diversity was defined as previously described (100) and was calculated using equation 1 of a published method (101). The SNPs were detected for positions with ≥5× sequence coverage using the following six criteria: (i) at least one read, (ii) at least two reads, (iii) more than 10% coverage, (iv) more than 20% coverage, (v) more than 10% coverage or at least two reads, and (vi) more than 10% coverage and at least two reads. These criteria were applied to the second-most-frequent nucleotide at each position.

### Redundancy of obtained environmental viral genomes.

To detect redundancies among TOV-EVGs and OBV-EVGs, an all-against-all BLASTn search was conducted. We merged high-scoring segment pairs (HSPs) for each resulting pair, and if the merged HSPs covered ≥80% of the shorter EVG, with ≥95% average identity, the EVGs were considered redundant. Nonredundant EVGs were obtained by single-linkage clustering of these redundant pairs.

### Viral genomes.

We first compiled 46 OBV-EVGs, 1,554 TOV-EVGs, and 247 EVGs from three projects, including 192 complete contigs (33), 54 circular consensus genomes (29), and a complete viral genome obtained from samples from single amplified genomes (SAG) (39). The RVGs were retrieved from RefSeq (release 75; March 2016), EBI Genomes Pages (May 2015), and CAMERA. We selected dsDNA viral genomes that were larger than 10 kb. We then removed the genomes of eukaryotic viruses identified using the GenomeNet Virus–Host Database (85). Thirty-six EVGs (i.e., 1 OBV, 32 TOVs, and 3 others) were most similar to eukaryotic viral genomes among RVGs and were removed from the proteomic tree and gOTU analyses, which were used to compare the levels of diversity of the RVGs and EVGs of prokaryotic viruses.

### Proteomic tree.

We constructed a proteomic tree as previously described (102). Briefly, the all-against-all distance matrix of the EVG/RVG data set was calculated on the basis of the normalized bit score of tBLASTx (*S*_G_), and the proteomic tree was built with BIONJ using the distance matrix. The proteomic tree, gene annotations, and genome alignment views are accessible at http://www.genome.ad.jp/viptree/EVG2017.

### Genus-level operational taxonomic units.

The genus-level threshold value for gOTU clustering was estimated from a subset of the RVGs used in this study (i.e., 345 prokaryotic dsDNA viruses), each of which was assigned to a viral genus (i.e., 82 genera in total). We constructed gOTUs with different *S*_G_ cutoffs (intervals of 0.01) and evaluated how closely the resulting gOTUs corresponded to the genus-level viral classifications using the adjusted Rand index (103).

### Host predictions according to proteomic similarities.

We attempted to predict host taxonomic groups for EVGs on the basis of viral genomic similarities measured with *S*_G_. We estimated the precision of our prediction method on the basis of RVGs (i.e., 1,285 prokaryotic dsDNA viruses), each of which was linked to a uniquely assigned host taxonomic group according to the Virus-Host Database. Regarding host taxonomic groups, *Cyanobacteria* (phylum) and *Enterobacteriaceae* (family) were regarded as individual host taxonomic groups because closely related viruses are known to infect hosts of different genera belonging to these host groups. The remaining viral hosts were grouped at the genus level. For each RVG, the best *S*_G_ values for the members of the same host group, and for the members outside the host group, were recorded (i.e., 2,570 *S*_G_ scores in total). A precision curve was generated using sliding *S*_G_ cutoff values (Fig. S6). When the *S*_G_ cutoff value was >0.3889 or >0.2937, the viral pairs were predicted to infect hosts in the same group at >95% or >90% precision, respectively.

### Photosynthetic gene identification.

To detect photosynthetic genes in the EVG/RVG data set, we used PSI-BLAST (E value, 1e−6; inclusion_ethresh, 1e−6; num_iterations, 3) and the query sequences listed in Table S1I.

### Phylogenetic trees.

Multiple sequences were aligned using the MAFFT program (version 7.245) (104), with the FFT-NS-2 mode and a maximum of 1,000 iterations (--retree 2, --maxiterate 1000). Conserved positions in the alignments were selected with the trimAl program (version 1.3) (105). Maximum likelihood trees with 100 bootstrap replicates were calculated with RAxML (version 8.2.4) (106) using the fast bootstrapping mode, and models were selected by the use of ProteinModelSelection.pl (i.e., LGF for DNA polymerase B and LG for chaperonins, IscU, and ATC).

### Recruitment of Pacific Ocean virome sequences.

Reads (3.68 M sequences) and proteins (2.78 M sequences) of 16 photic POV samples were downloaded from iMicrobe (http://data.imicrobe.us). These sequences were mapped on EVGs and RVGs using BLASTn (for reads; E value, <1e−3) and tBLASTn (for proteins; E value, <1e−3) if the alignment revealed ≥60% identity and covered ≥80% of the query sequence.

### Accession number(s).

Read and assembled sequences obtained from OBV were deposited at DNA Data Bank of Japan (DDBJ) under accession numbers DRR053207 to DRR053215 and SAMD00045684 to SAMD00045692. The sequence data for the OBV project are accessible under DDBJ BioProject accession number PRJDB4437. Sequences and additional data are available at ftp://ftp.genome.jp/pub/db/community/EVG2017.
