# Supplementary figures and images for: Environmental Viral Genomes Shed New Light on Virus-Host Interactions in the Ocean
Source: mSphere. 2017 Mar 1;2(2):e00359-16. doi: 10.1128/mSphere.00359-16 (PMC5332604; doi:10.1128/mSphere.00359-16)

**A**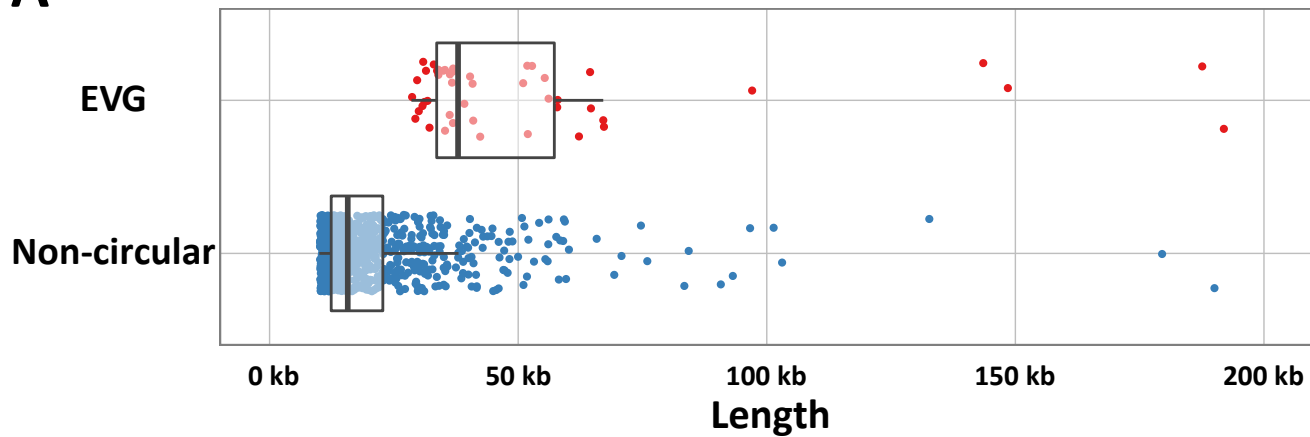**B**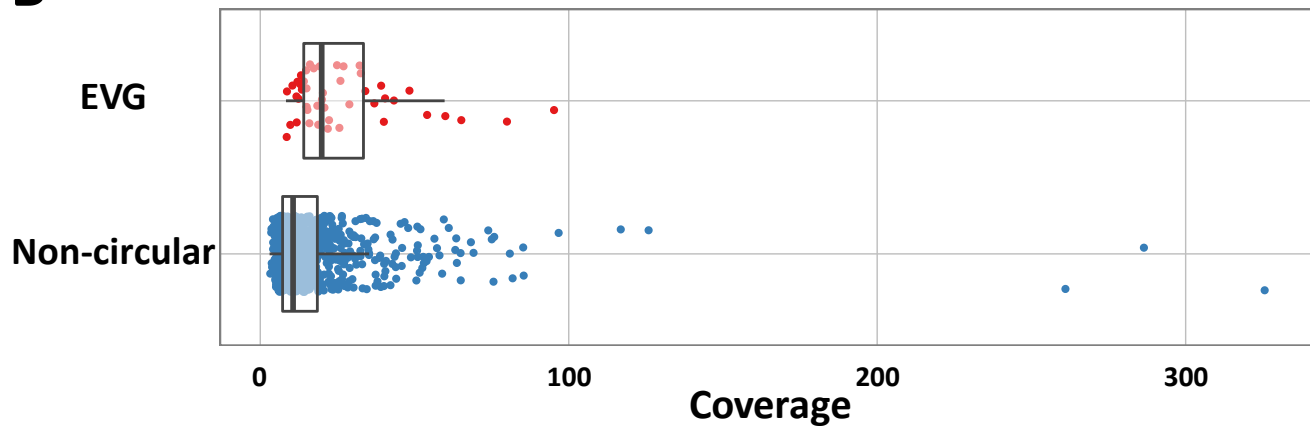

Supplement: FIG S1 [file sph002172244sf1.pdf]

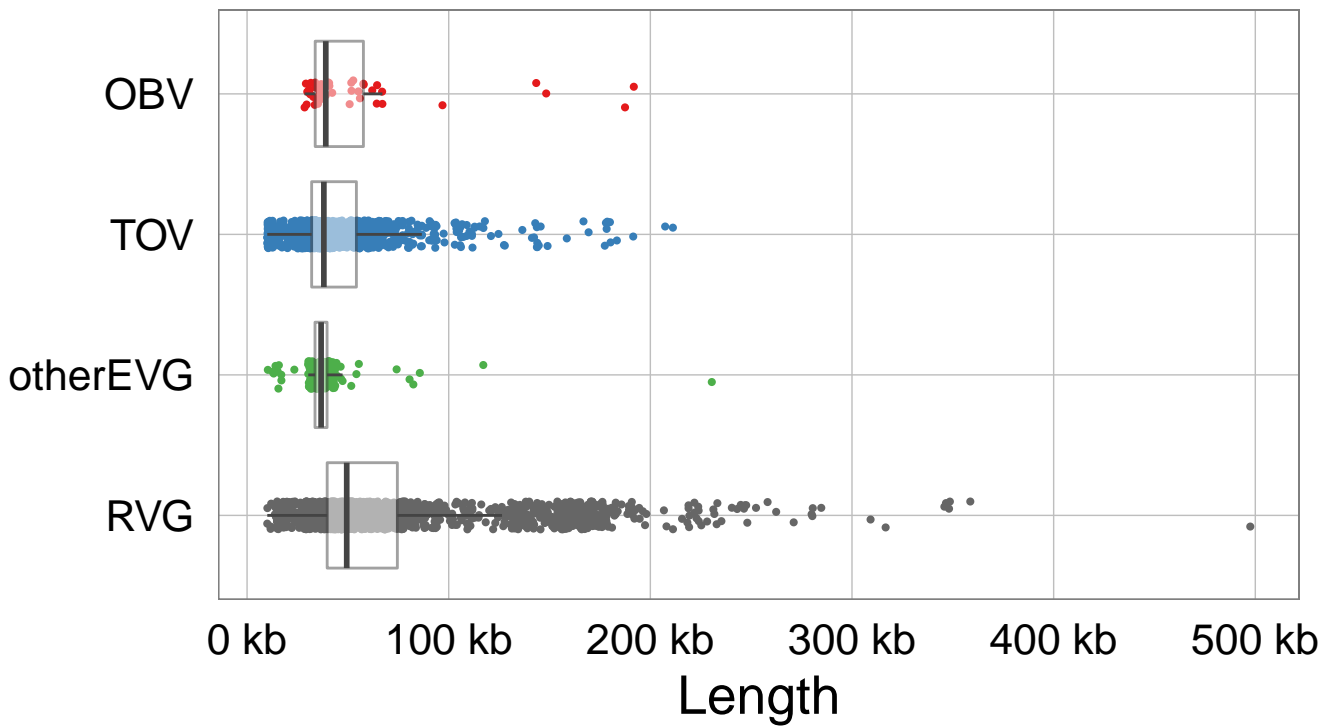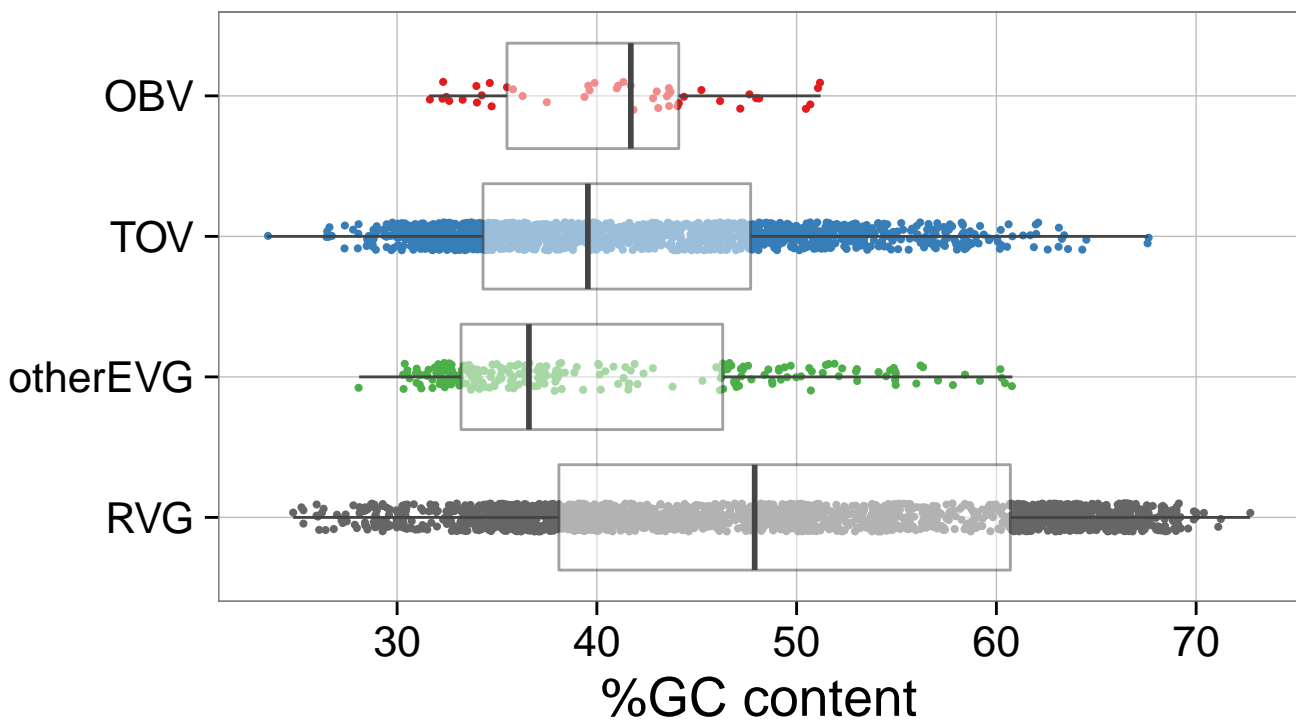

Supplement: FIG S3 [file sph002172244sf3.pdf]

**A**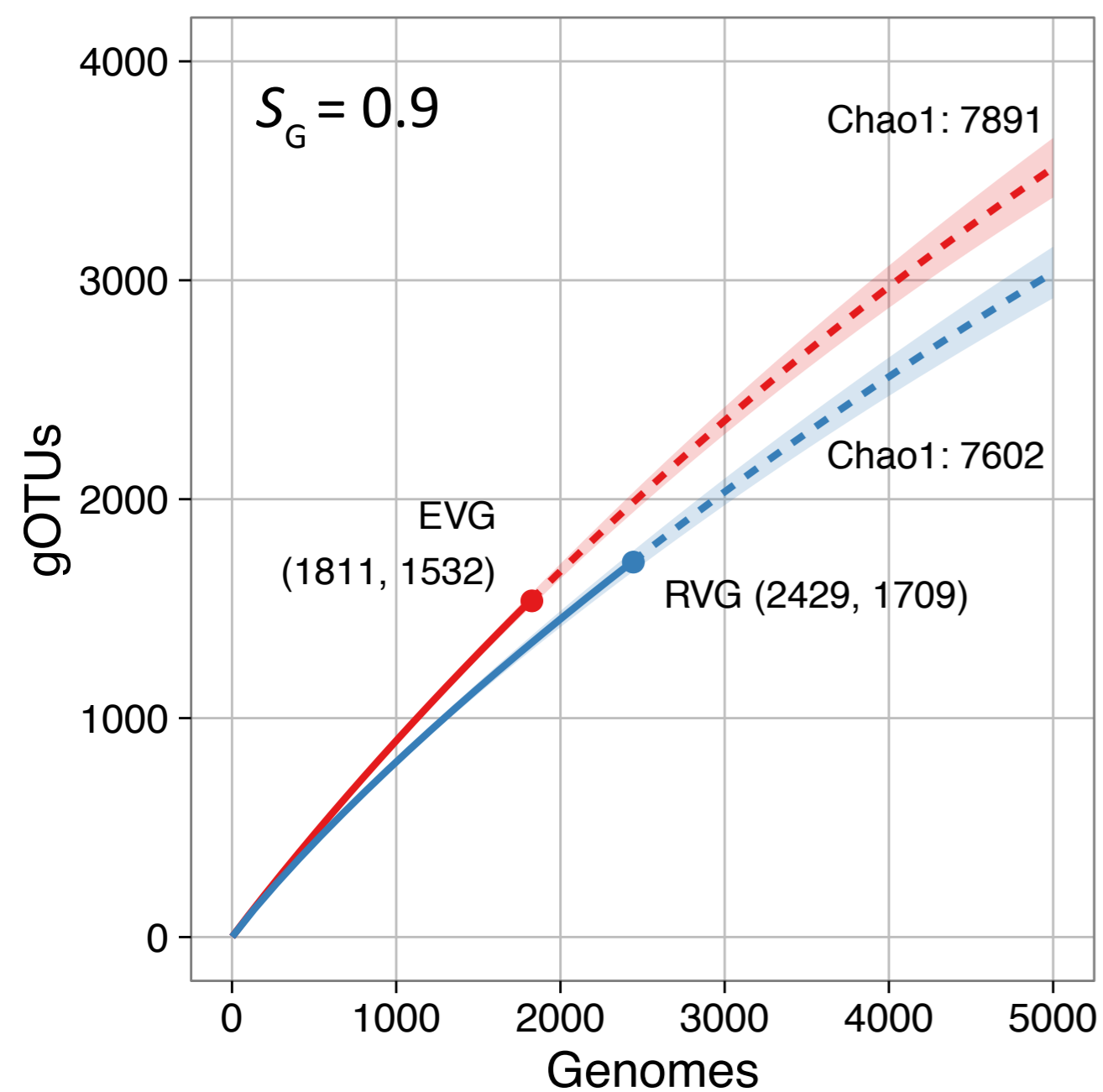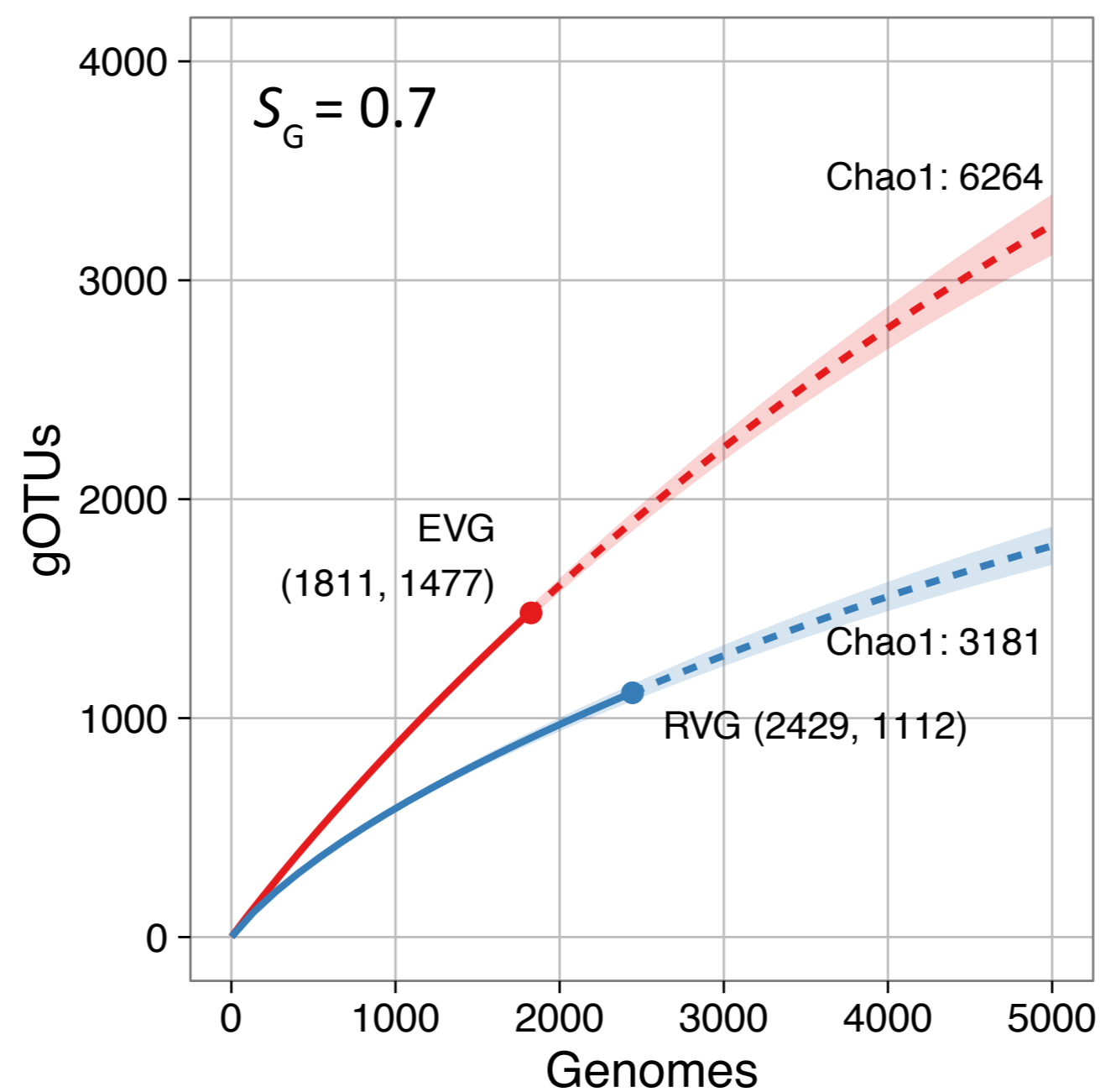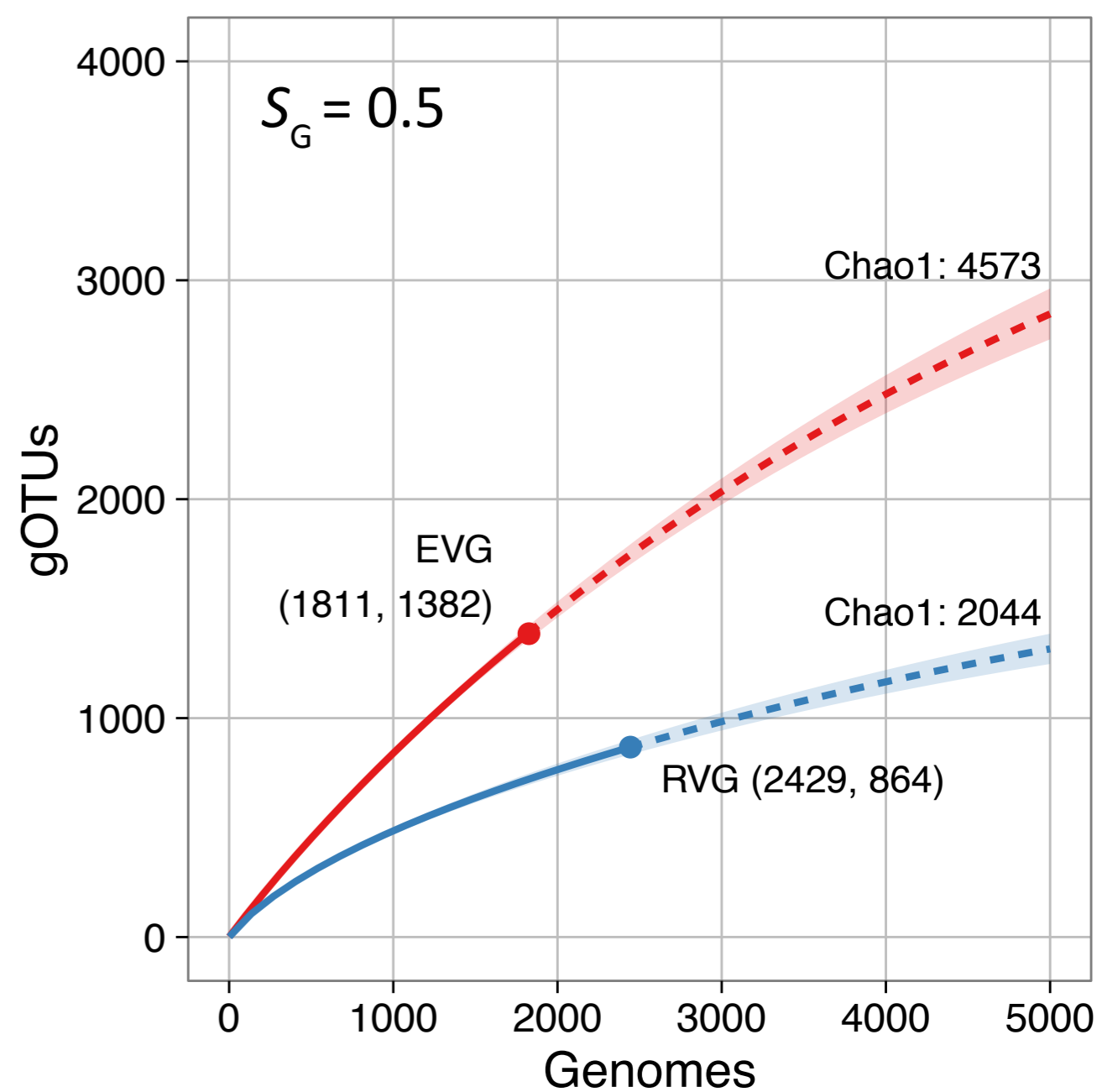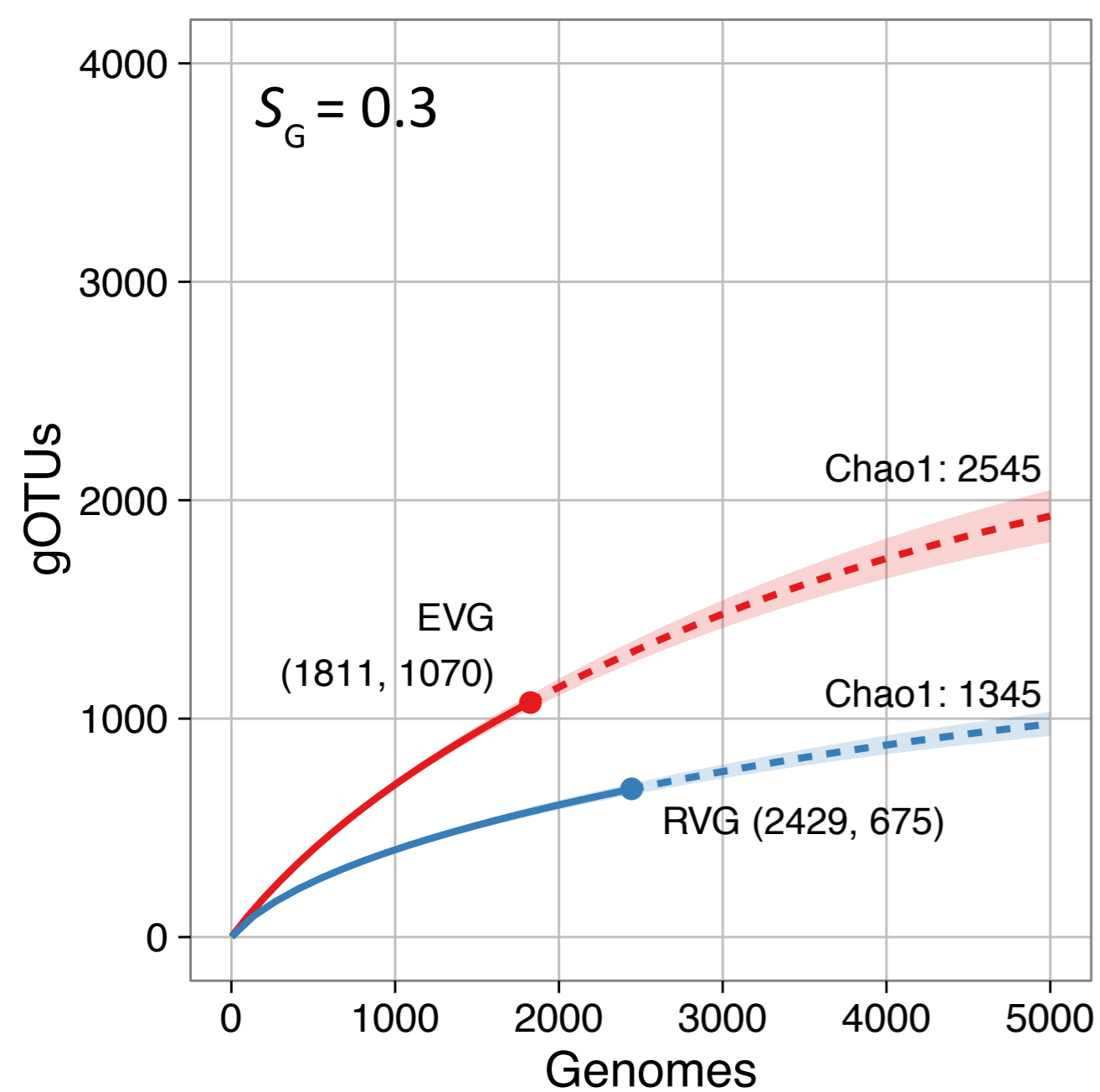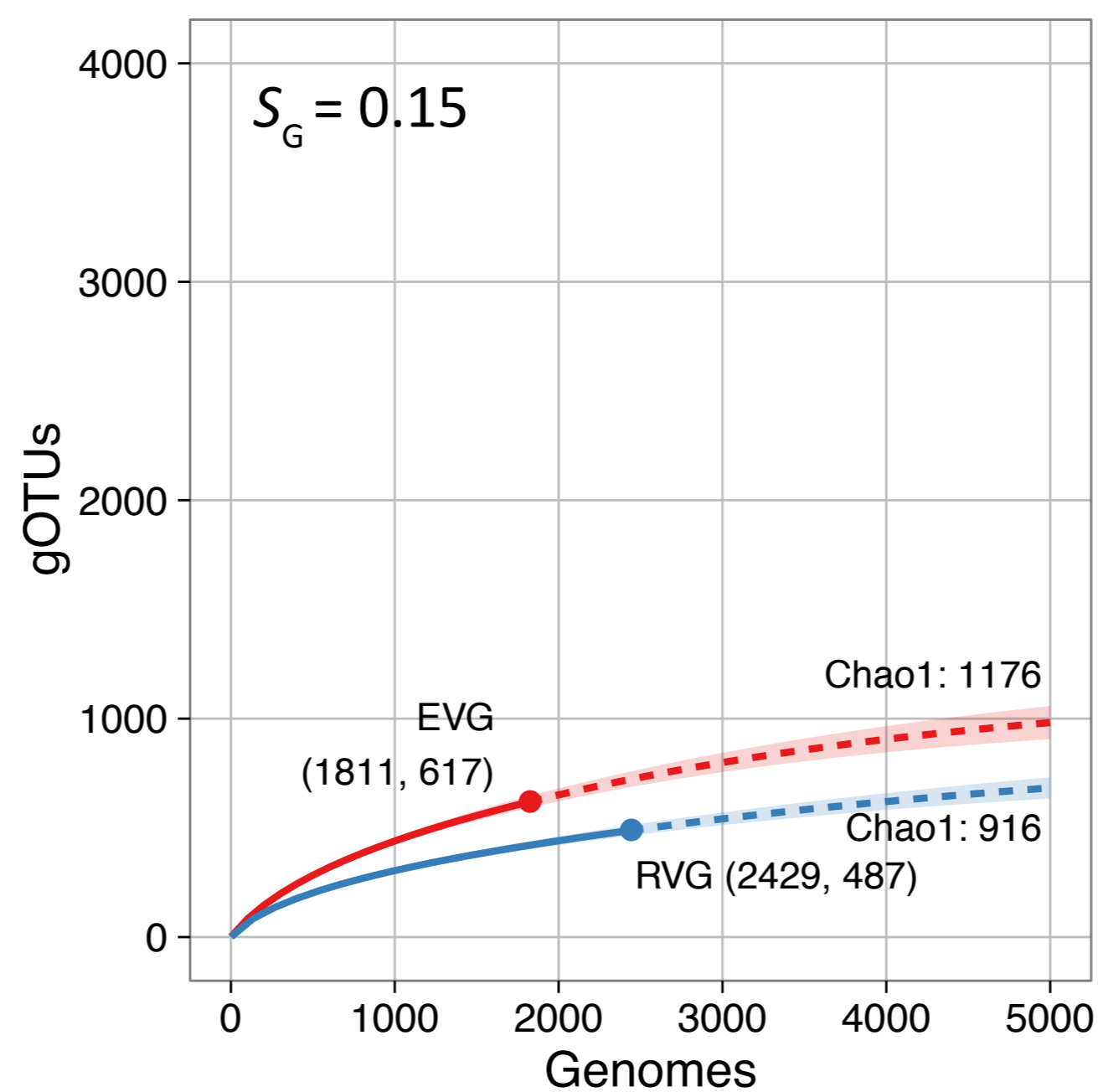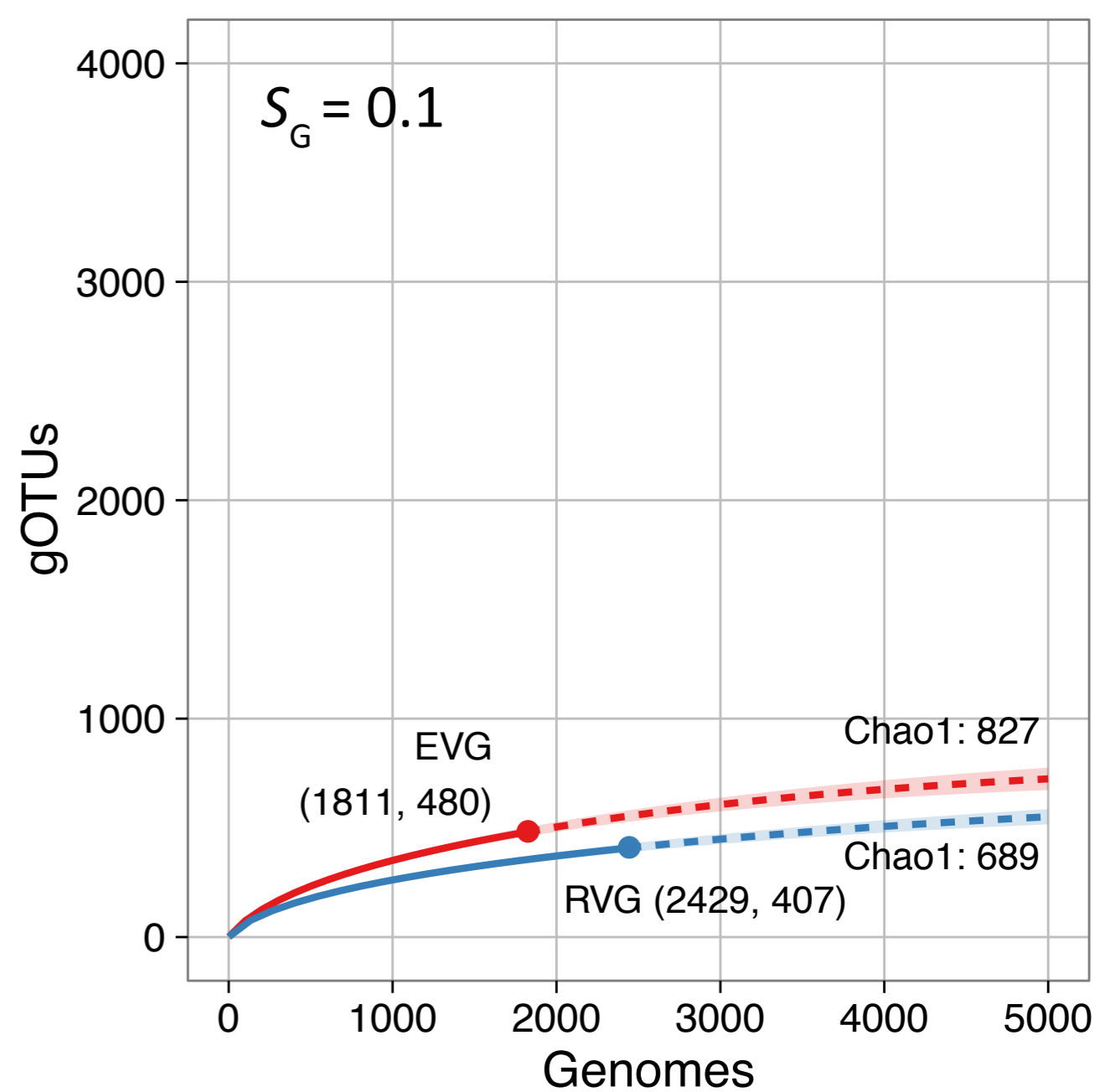**B**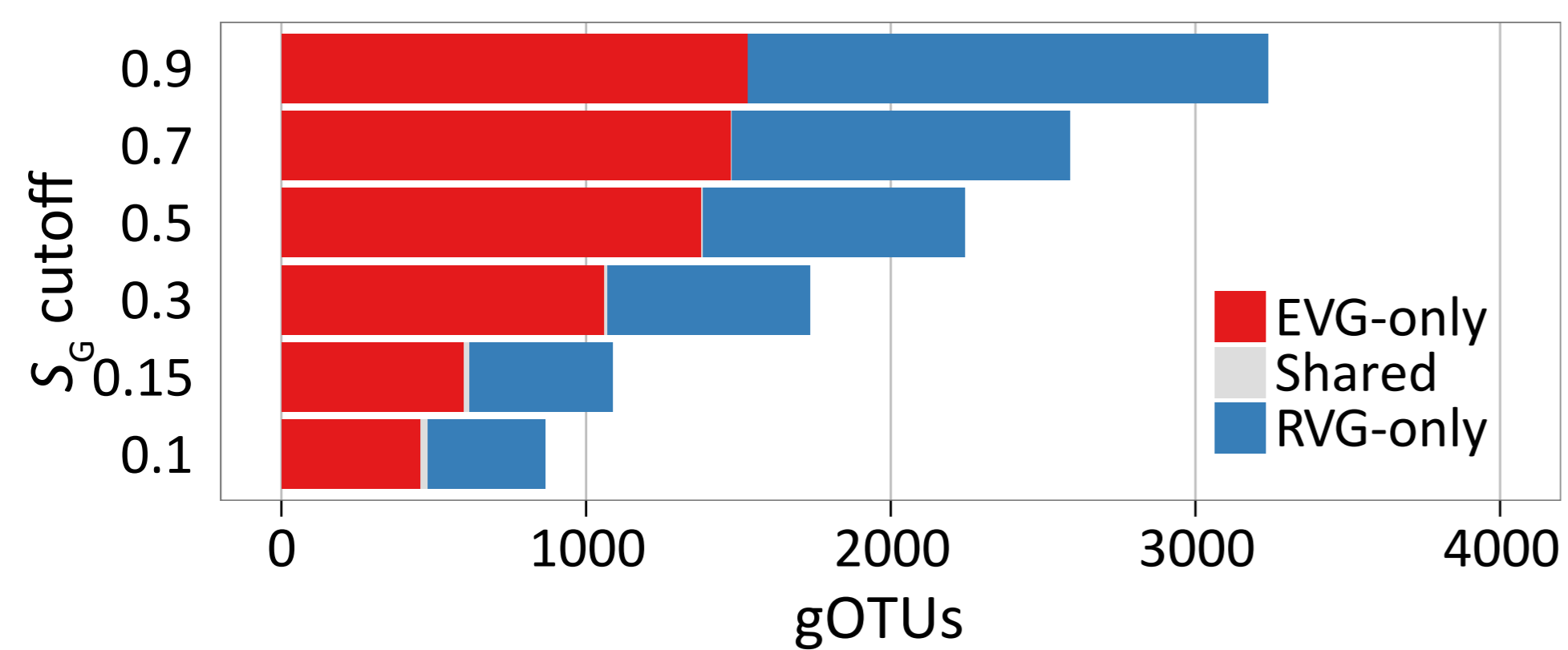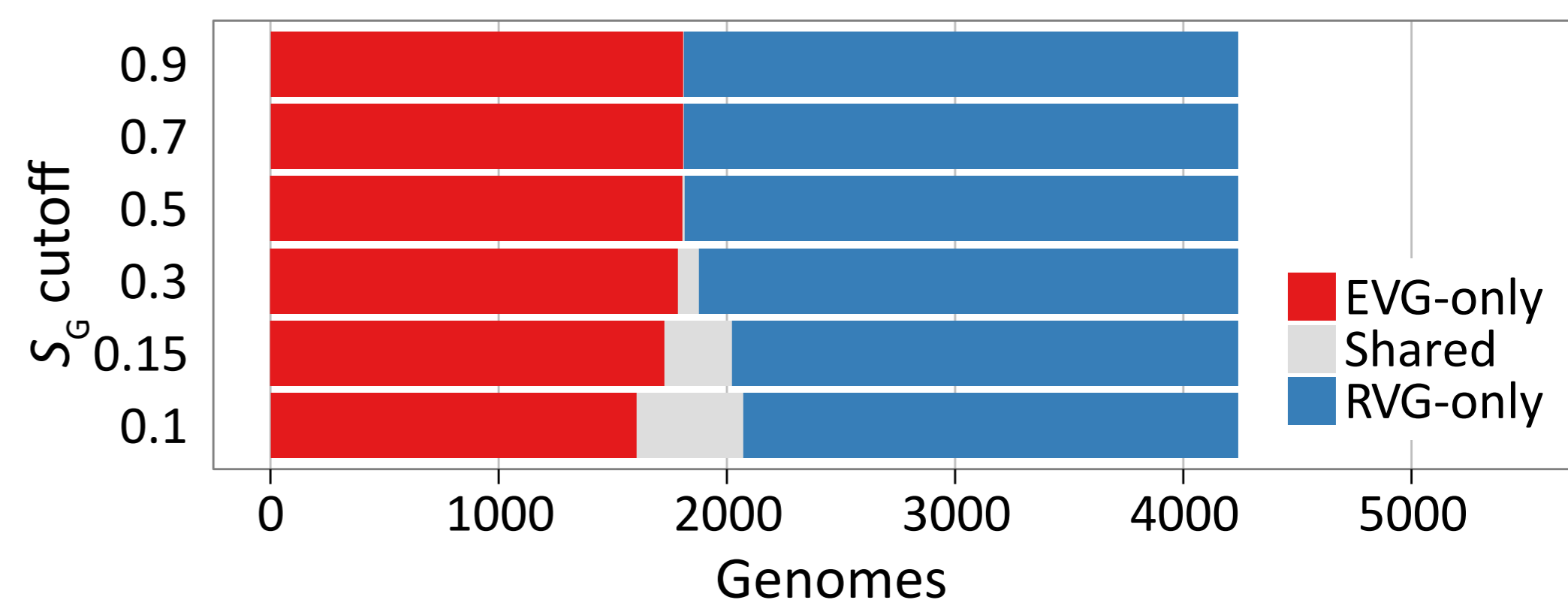

Supplement: FIG S4 [file sph002172244sf4.pdf]

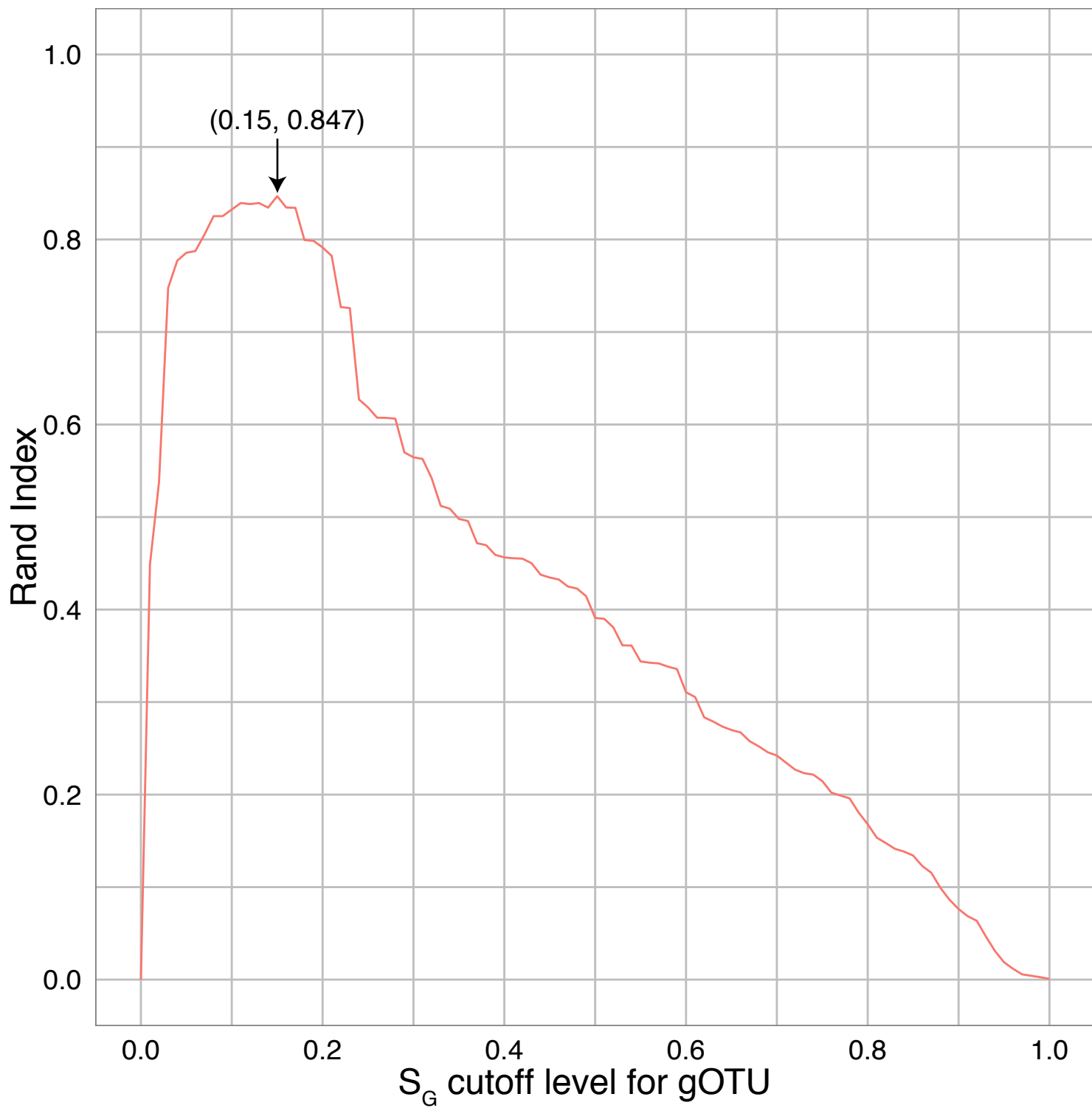

Supplement: FIG S5 [file sph002172244sf5.pdf]

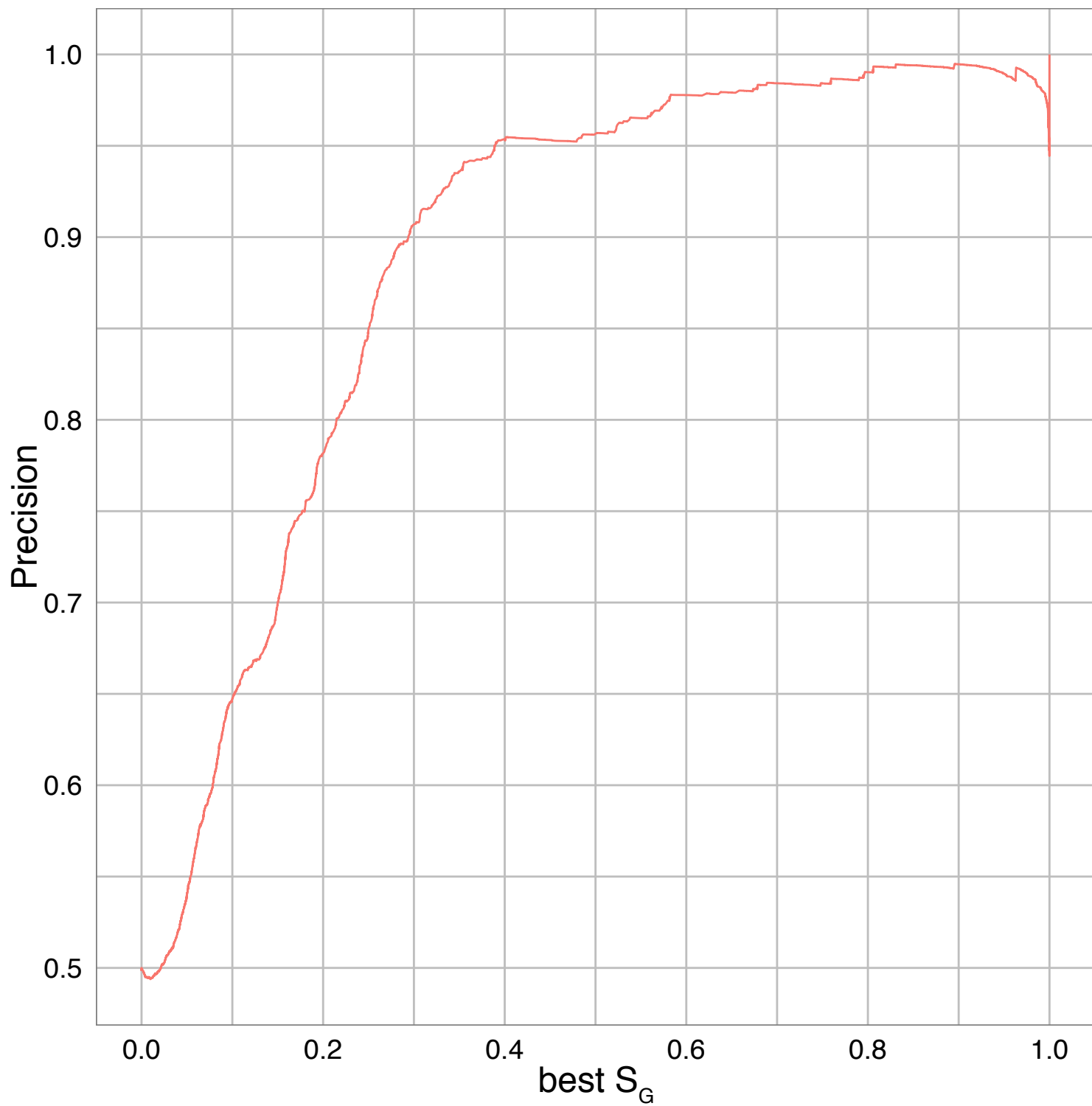

Supplement: FIG S6 [file sph002172244sf6.pdf]

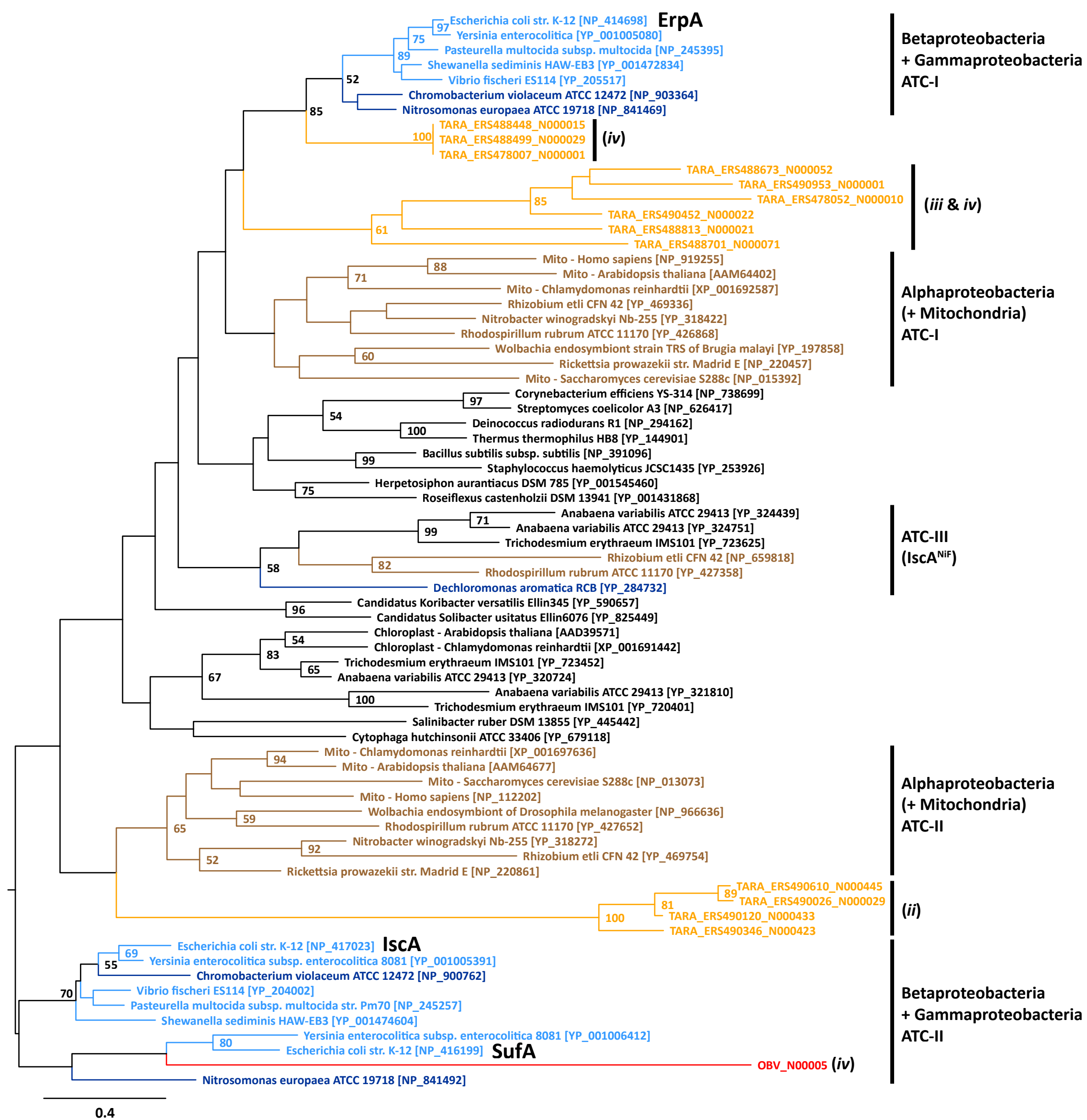

Supplement: FIG S7 [file sph002172244sf7.pdf]

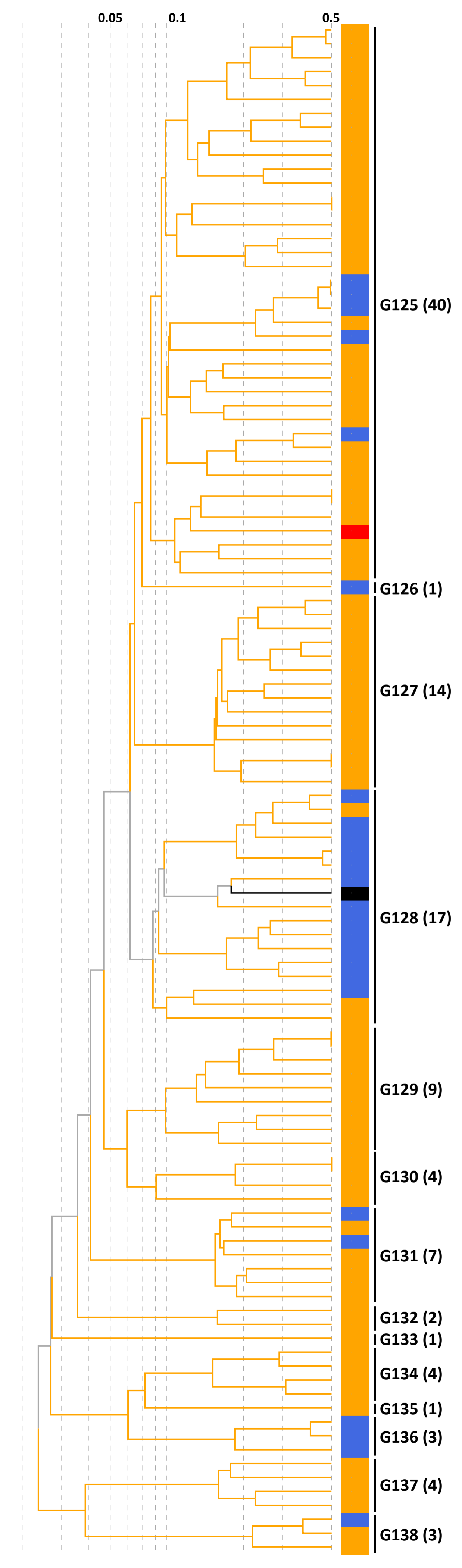

Supplement: FIG S8 [file sph002172244sf8.pdf]
