# Supplementary material for: Environmental Viral Genomes Shed New Light on Virus-Host Interactions in the Ocean
Source: mSphere. 2017 Mar 1;2(2):e00359-16. doi: 10.1128/mSphere.00359-16 (PMC5332604; doi:10.1128/mSphere.00359-16)
Supplement: FIG S2 [file sph002172244sf2.pdf]

A

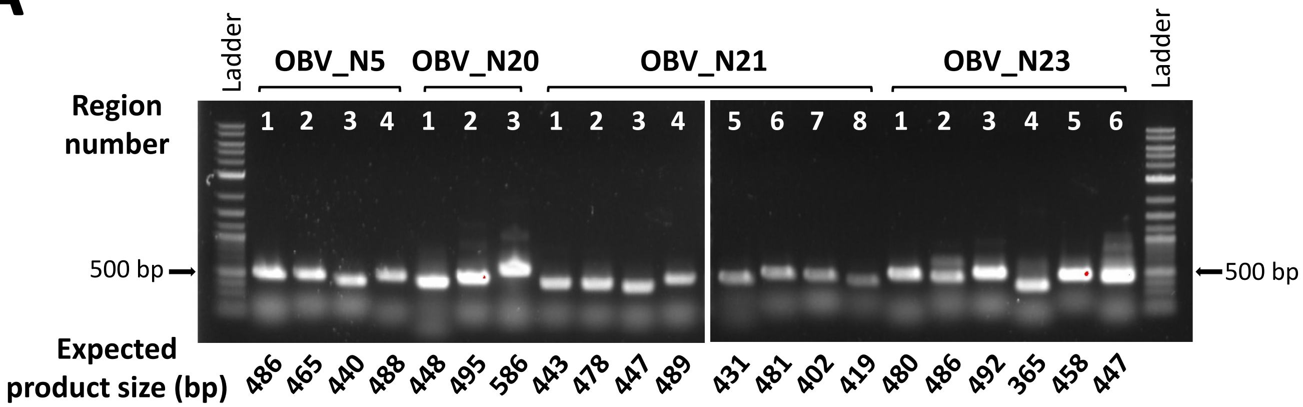
Ladder
OBV_N5
OBV_N20
OBV_N21
OBV_N23
Ladder
Region number
1 2 3 4 1 2 3 1 2 3 4 5 6 7 8 1 2 3 4 5 6
500 bp
500 bp
Expected product size (bp)
486 465 440 488 448 495 586 443 478 447 489 431 481 402 419 480 486 492 365 458 447

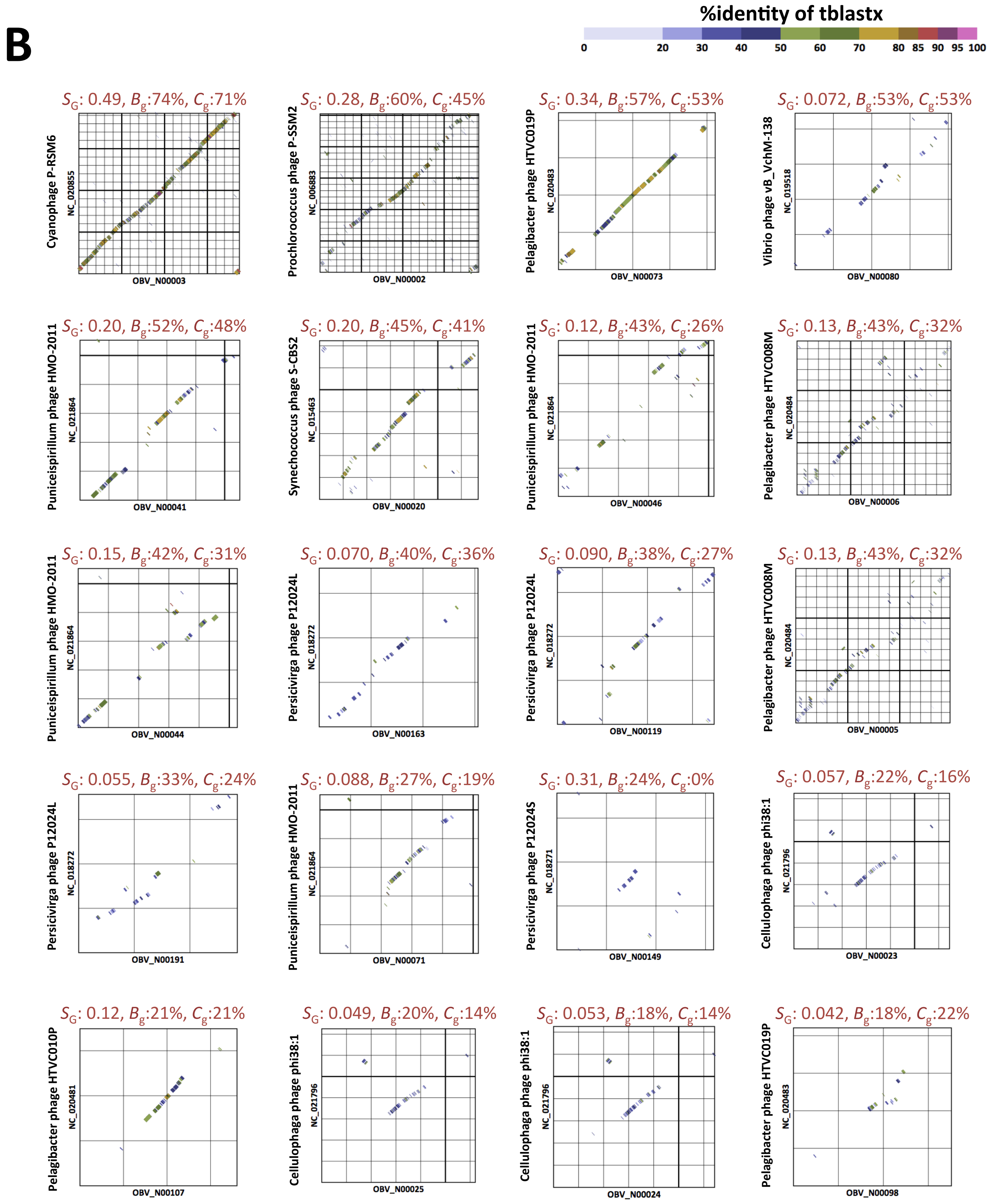
B
%identity of tblastx
0
20
30
40
50
60
70
80
85
90
95
100
S_G: 0.49, B_g:74%, C_g:71%
Cyanophage P-RSM6
NC_020855
OBV_N00003
S_G: 0.28, B_g:60%, C_g:45%
Prochlorococcus phage P-SSM2
NC_006883
OBV_N00002
S_G: 0.34, B_g:57%, C_g:53%
Pelagibacter phage HTVC019P
NC_020483
OBV_N00073
S_G: 0.072, B_g:53%, C_g:53%
Vibrio phage vB_VchM-138
NC_019518
OBV_N00080
S_G: 0.20, B_g:52%, C_g:48%
Puniceispirillum phage HMO-2011
NC_021864
OBV_N00041
S_G: 0.20, B_g:45%, C_g:41%
Synechococcus phage S-CBS2
NC_015463
OBV_N00020
S_G: 0.12, B_g:43%, C_g:26%
Puniceispirillum phage HMO-2011
NC_021864
OBV_N00046
S_G: 0.13, B_g:43%, C_g:32%
Pelagibacter phage HTVC008M
NC_020484
OBV_N00006
S_G: 0.15, B_g:42%, C_g:31%
Puniceispirillum phage HMO-2011
NC_021864
OBV_N00044
S_G: 0.070, B_g:40%, C_g:36%
Persicivirga phage P12024L
NC_018272
OBV_N00163
S_G: 0.090, B_g:38%, C_g:27%
Persicivirga phage P12024L
NC_018272
OBV_N00119
S_G: 0.13, B_g:43%, C_g:32%
Pelagibacter phage HTVC008M
NC_020484
OBV_N00005
S_G: 0.055, B_g:33%, C_g:24%
Persicivirga phage P12024L
NC_018272
OBV_N00191
S_G: 0.088, B_g:27%, C_g:19%
Puniceispirillum phage HMO-2011
NC_021864
OBV_N00071
S_G: 0.31, B_g:24%, C_g:0%
Persicivirga phage P12024S
NC_018271
OBV_N00149
S_G: 0.057, B_g:22%, C_g:16%
Cellulophaga phage phi38:1
NC_021796
OBV_N00023
S_G: 0.12, B_g:21%, C_g:21%
Pelagibacter phage HTVC010P
NC_020481
OBV_N00107
S_G: 0.049, B_g:20%, C_g:14%
Cellulophaga phage phi38:1
NC_021796
OBV_N00025
S_G: 0.053, B_g:18%, C_g:14%
Cellulophaga phage phi38:1
NC_021796
OBV_N00024
S_G: 0.042, B_g:18%, C_g:22%
Pelagibacter phage HTVC019P
NC_020483
OBV_N00098

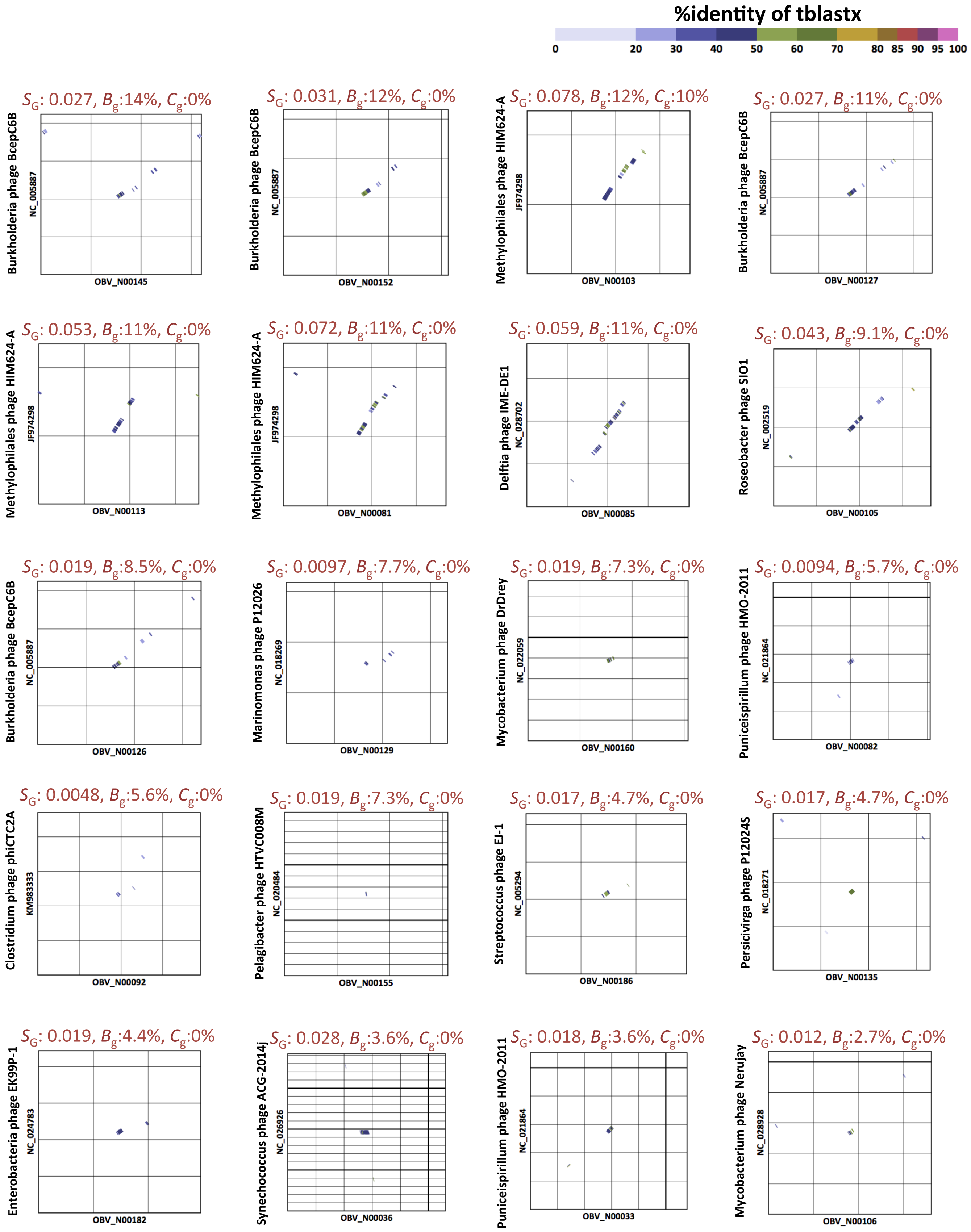

%identity of tblastx
0 20 30 40 50 60 70 80 85 90 95 100
S_G: 0.027, B_g:14%, C_g:0%
Burkholderia phage BcepC6B
NC_005887
OBV_N00145
S_G: 0.031, B_g:12%, C_g:0%
Burkholderia phage BcepC6B
NC_005887
OBV_N00152
S_G: 0.078, B_g:12%, C_g:10%
Methylophilales phage HIM624-A
JF974298
OBV_N00103
S_G: 0.027, B_g:11%, C_g:0%
Burkholderia phage BcepC6B
NC_005887
OBV_N00127
S_G: 0.053, B_g:11%, C_g:0%
Methylophilales phage HIM624-A
JF974298
OBV_N00113
S_G: 0.072, B_g:11%, C_g:0%
Methylophilales phage HIM624-A
JF974298
OBV_N00081
S_G: 0.059, B_g:11%, C_g:0%
Delftia phage IME-DE1
NC_028702
OBV_N00085
S_G: 0.043, B_g:9.1%, C_g:0%
Roseobacter phage SIO1
NC_002519
OBV_N00105
S_G: 0.019, B_g:8.5%, C_g:0%
Burkholderia phage BcepC6B
NC_005887
OBV_N00126
S_G: 0.0097, B_g:7.7%, C_g:0%
Marinomonas phage P12026
NC_018269
OBV_N00129
S_G: 0.019, B_g:7.3%, C_g:0%
Mycobacterium phage DrDrey
NC_022059
OBV_N00160
S_G: 0.0094, B_g:5.7%, C_g:0%
Puniceispirillum phage HMO-2011
NC_021864
OBV_N00082
S_G: 0.0048, B_g:5.6%, C_g:0%
Clostridium phage phiCTC2A
KM983333
OBV_N00092
S_G: 0.019, B_g:7.3%, C_g:0%
Pelagibacter phage HTVC008M
NC_020484
OBV_N00155
S_G: 0.017, B_g:4.7%, C_g:0%
Streptococcus phage EJ-1
NC_005294
OBV_N00186
S_G: 0.017, B_g:4.7%, C_g:0%
Persicivirga phage P12024S
NC_018271
OBV_N00135
S_G: 0.019, B_g:4.4%, C_g:0%
Enterobacteria phage EK99P-1
NC_024783
OBV_N00182
S_G: 0.028, B_g:3.6%, C_g:0%
Synechococcus phage ACG-2014j
NC_026926
OBV_N00036
S_G: 0.018, B_g:3.6%, C_g:0%
Puniceispirillum phage HMO-2011
NC_021864
OBV_N00033
S_G: 0.012, B_g:2.7%, C_g:0%
Mycobacterium phage Nerujay
NC_028928
OBV_N00106

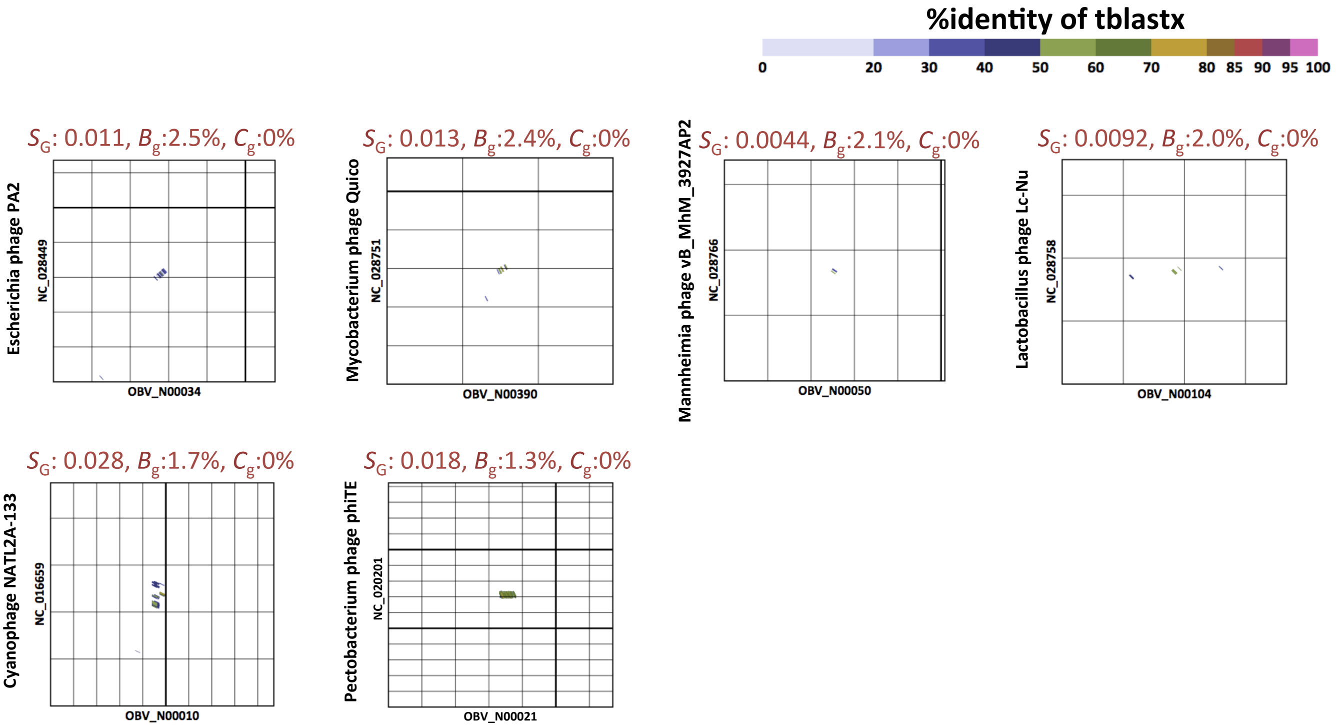
%identity of tblastx
0
20
30
40
50
60
70
80
85
90
95
100
SG: 0.011, Bg:2.5%, Cg:0%
Escherichia phage PA2
NC_028449
OBV_N00034
SG: 0.013, Bg:2.4%, Cg:0%
Mycobacterium phage Quico
NC_028751
OBV_N00390
SG: 0.0044, Bg:2.1%, Cg:0%
Mannheimia phage vB_MhM_3927AP2
NC_028766
OBV_N00050
SG: 0.0092, Bg:2.0%, Cg:0%
Lactobacillus phage Lc-Nu
NC_028758
OBV_N00104
SG: 0.028, Bg:1.7%, Cg:0%
Cyanophage NATL2A-133
NC_016659
OBV_N00010
SG: 0.018, Bg:1.3%, Cg:0%
Pectobacterium phage phiTE
NC_020201
OBV_N00021

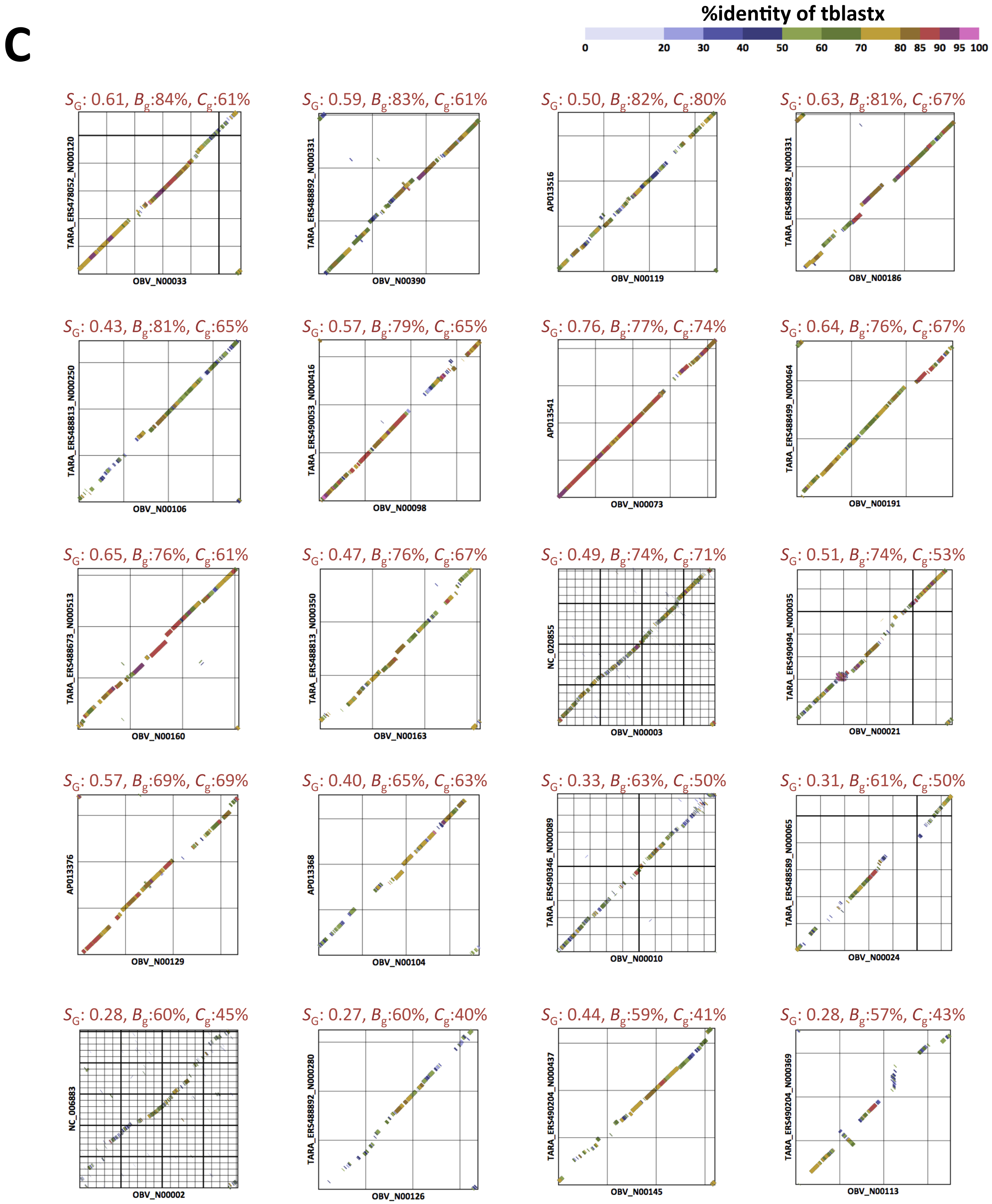
C
%identity of tblastx
0 20 30 40 50 60 70 80 85 90 95 100
S_G: 0.61, B_g:84%, C_g:61%
TARA_ERS478052_N000120
OBV_N00033
S_G: 0.59, B_g:83%, C_g:61%
TARA_ERS488892_N000331
OBV_N00390
S_G: 0.50, B_g:82%, C_g:80%
AP013516
OBV_N00119
S_G: 0.63, B_g:81%, C_g:67%
TARA_ERS488892_N000331
OBV_N00186
S_G: 0.43, B_g:81%, C_g:65%
TARA_ERS488813_N000250
OBV_N00106
S_G: 0.57, B_g:79%, C_g:65%
TARA_ERS490053_N000416
OBV_N00098
S_G: 0.76, B_g:77%, C_g:74%
AP013541
OBV_N00073
S_G: 0.64, B_g:76%, C_g:67%
TARA_ERS488499_N000464
OBV_N00191
S_G: 0.65, B_g:76%, C_g:61%
TARA_ERS488673_N000513
OBV_N00160
S_G: 0.47, B_g:76%, C_g:67%
TARA_ERS488813_N000350
OBV_N00163
S_G: 0.49, B_g:74%, C_g:71%
NC_020855
OBV_N00003
S_G: 0.51, B_g:74%, C_g:53%
TARA_ERS490494_N000035
OBV_N00021
S_G: 0.57, B_g:69%, C_g:69%
AP013376
OBV_N00129
S_G: 0.40, B_g:65%, C_g:63%
AP013368
OBV_N00104
S_G: 0.33, B_g:63%, C_g:50%
TARA_ERS490346_N000089
OBV_N00010
S_G: 0.31, B_g:61%, C_g:50%
TARA_ERS488589_N000065
OBV_N00024
S_G: 0.28, B_g:60%, C_g:45%
NC_006883
OBV_N00002
S_G: 0.27, B_g:60%, C_g:40%
TARA_ERS488892_N000280
OBV_N00126
S_G: 0.44, B_g:59%, C_g:41%
TARA_ERS490204_N000437
OBV_N00145
S_G: 0.28, B_g:57%, C_g:43%
TARA_ERS490204_N000369
OBV_N00113

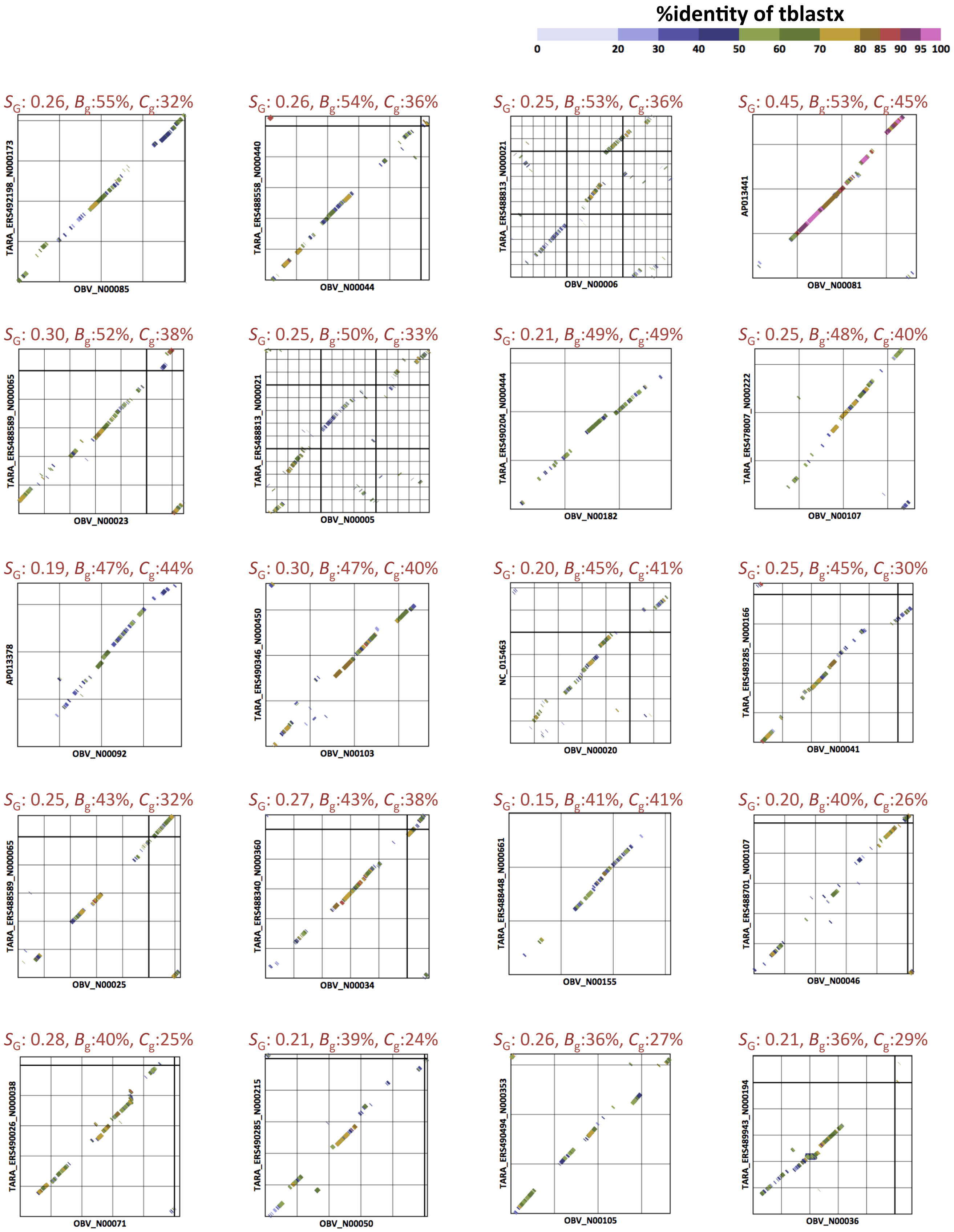
%identity of tblastx
0
20
30
40
50
60
70
80
85
90
95
100
S_G: 0.26, B_g:55%, C_g:32%
TARA_ERS492198_N000173
OBV_N00085
S_G: 0.26, B_g:54%, C_g:36%
TARA_ERS488558_N000440
OBV_N00044
S_G: 0.25, B_g:53%, C_g:36%
TARA_ERS488813_N000021
OBV_N00006
S_G: 0.45, B_g:53%, C_g:45%
AP013441
OBV_N00081
S_G: 0.30, B_g:52%, C_g:38%
TARA_ERS488589_N000065
OBV_N00023
S_G: 0.25, B_g:50%, C_g:33%
TARA_ERS488813_N000021
OBV_N00005
S_G: 0.21, B_g:49%, C_g:49%
TARA_ERS490204_N000444
OBV_N00182
S_G: 0.25, B_g:48%, C_g:40%
TARA_ERS478007_N000222
OBV_N00107
S_G: 0.19, B_g:47%, C_g:44%
AP013378
OBV_N00092
S_G: 0.30, B_g:47%, C_g:40%
TARA_ERS490346_N000450
OBV_N00103
S_G: 0.20, B_g:45%, C_g:41%
NC_015463
OBV_N00020
S_G: 0.25, B_g:45%, C_g:30%
TARA_ERS489285_N000166
OBV_N00041
S_G: 0.25, B_g:43%, C_g:32%
TARA_ERS488589_N000065
OBV_N00025
S_G: 0.27, B_g:43%, C_g:38%
TARA_ERS488340_N000360
OBV_N00034
S_G: 0.15, B_g:41%, C_g:41%
TARA_ERS488448_N000661
OBV_N00155
S_G: 0.20, B_g:40%, C_g:26%
TARA_ERS488701_N000107
OBV_N00046
S_G: 0.28, B_g:40%, C_g:25%
TARA_ERS490026_N000038
OBV_N00071
S_G: 0.21, B_g:39%, C_g:24%
TARA_ERS490285_N000215
OBV_N00050
S_G: 0.26, B_g:36%, C_g:27%
TARA_ERS490494_N000353
OBV_N00105
S_G: 0.21, B_g:36%, C_g:29%
TARA_ERS489943_N000194
OBV_N00036

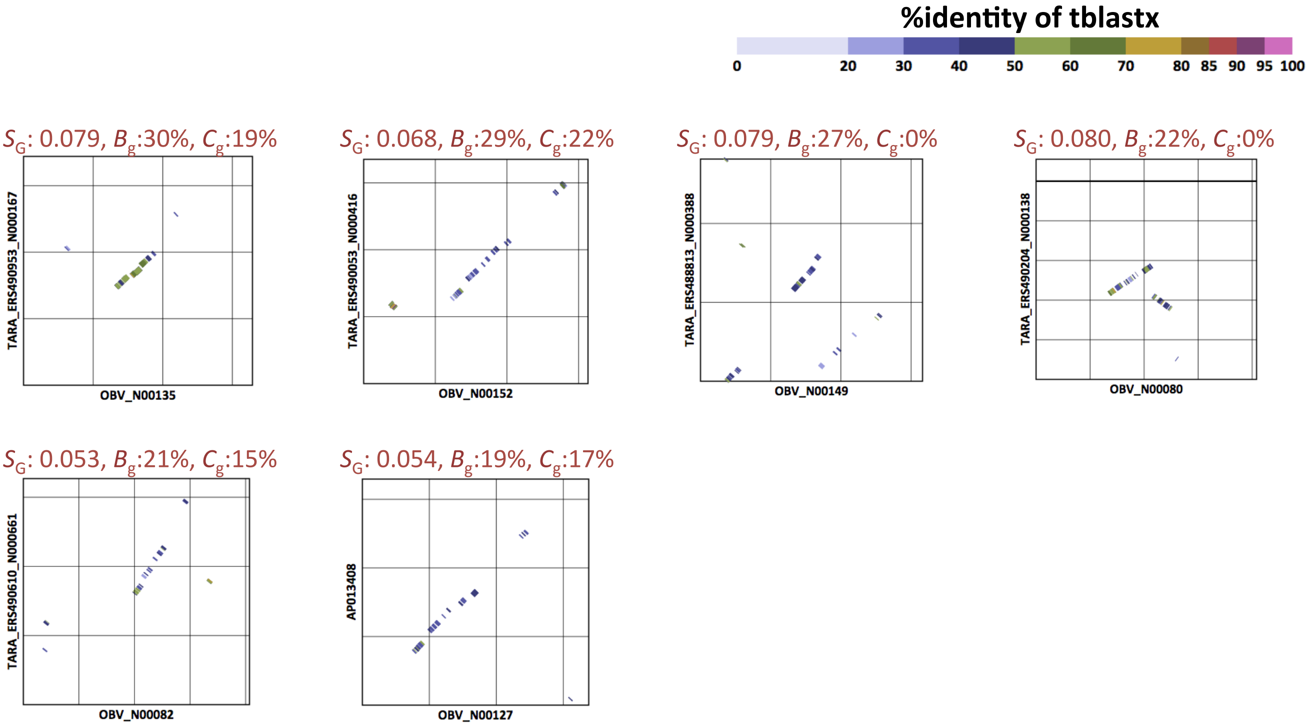

%identity of tblastx
0
20
30
40
50
60
70
80
85
90
95
100
SG: 0.079, Bg:30%, Cg:19%
TARA_ERS490953_N000167
OBV_N00135
SG: 0.068, Bg:29%, Cg:22%
TARA_ERS490053_N000416
OBV_N00152
SG: 0.079, Bg:27%, Cg:0%
TARA_ERS488813_N000388
OBV_N00149
SG: 0.080, Bg:22%, Cg:0%
TARA_ERS490204_N000138
OBV_N00080
SG: 0.053, Bg:21%, Cg:15%
TARA_ERS490610_N000661
OBV_N00082
SG: 0.054, Bg:19%, Cg:17%
AP013408
OBV_N00127
