# Supplementary material for: Environmental Viral Genomes Shed New Light on Virus-Host Interactions in the Ocean
Source: mSphere. 2017 Mar 1;2(2):e00359-16. doi: 10.1128/mSphere.00359-16 (PMC5332604; doi:10.1128/mSphere.00359-16)
Supplement: TABLE S1 [file sph002172244st10.pdf]

Table S1A. SNP and nucleotide diversity of 46 OBV-EVGs.

OBV-EVG	length	position of ≥5 coverage	%position of ≥5 coverage	coverage	SNP (*i*) (≥1 reads)	SNP (*ii*) (≥2 reads)	SNP (*iii*) (>10%)	SNP (*iv*) (>20%)	SNP (*v*) (>10% or ≥2 reads)	SNP (*vi*) (>10% and ≥2 reads)	nucleotide diversity
OBV_N00002	191,793	191,663	99.93%	32.0	3.457%	1.369%	0.688%	0.316%	1.372%	0.685%	0.460%
OBV_N00003	187,437	187,415	99.99%	53.9	3.017%	1.005%	0.457%	0.242%	1.005%	0.457%	0.298%
OBV_N00005	148,347	148,016	99.78%	15.9	0.949%	0.261%	0.284%	0.126%	0.299%	0.245%	0.184%
OBV_N00006	143,397	143,368	99.98%	59.8	1.975%	0.354%	0.164%	0.094%	0.354%	0.164%	0.132%
OBV_N00010	96,913	95,243	98.28%	14.8	0.953%	0.252%	0.298%	0.075%	0.363%	0.187%	0.177%
OBV_N00020	67,112	66,752	99.46%	14.8	1.709%	0.328%	0.409%	0.058%	0.470%	0.267%	0.282%
OBV_N00021	66,977	66,416	99.16%	14.6	1.926%	0.768%	0.810%	0.385%	0.896%	0.682%	0.444%
OBV_N00023	64,495	64,187	99.52%	28.7	1.923%	0.598%	0.263%	0.070%	0.615%	0.246%	0.220%
OBV_N00024	64,322	64,295	99.96%	64.9	4.682%	1.041%	0.098%	0.019%	1.045%	0.093%	0.217%
OBV_N00025	62,085	61,398	98.89%	15.1	2.218%	0.697%	0.652%	0.143%	0.798%	0.551%	0.402%
OBV_N00033	57,810	57,698	99.81%	19.0	1.088%	0.269%	0.213%	0.021%	0.283%	0.199%	0.158%
OBV_N00034	57,704	57,625	99.86%	25.4	2.317%	1.118%	0.946%	0.545%	1.126%	0.937%	0.493%
OBV_N00036	55,971	55,569	99.28%	18.6	1.731%	0.378%	0.229%	0.025%	0.398%	0.209%	0.233%
OBV_N00041	55,201	55,137	99.88%	24.7	1.589%	0.426%	0.227%	0.118%	0.426%	0.227%	0.208%
OBV_N00044	52,651	52,596	99.90%	25.8	4.181%	2.053%	1.578%	0.808%	2.059%	1.572%	0.809%
OBV_N00046	51,792	50,044	96.62%	12.2	1.443%	0.701%	0.907%	0.456%	0.917%	0.691%	0.424%
OBV_N00050	50,862	50,674	99.63%	20.6	2.214%	0.926%	0.685%	0.253%	0.969%	0.641%	0.388%
OBV_N00071	51,687	51,677	99.98%	32.3	6.798%	3.303%	1.337%	0.327%	3.307%	1.333%	0.862%
OBV_N00073	42,216	40,139	95.08%	13.3	3.226%	1.602%	1.918%	0.989%	1.956%	1.565%	0.917%
OBV_N00080	40,816	39,612	97.05%	11.6	0.558%	0.099%	0.182%	0.023%	0.194%	0.086%	0.107%
OBV_N00081	36,738	36,030	98.07%	26.8	4.871%	2.406%	1.443%	0.508%	2.431%	1.418%	0.793%
OBV_N00082	40,689	40,677	99.97%	48.1	2.761%	1.219%	0.713%	0.524%	1.219%	0.713%	0.411%
OBV_N00085	40,177	38,550	95.95%	10.2	0.913%	0.231%	0.464%	0.138%	0.467%	0.228%	0.232%
OBV_N00092	39,038	39,003	99.91%	43.1	4.261%	1.587%	0.351%	0.033%	1.587%	0.351%	0.359%
OBV_N00098	35,134	35,008	99.64%	18.3	1.700%	0.686%	0.648%	0.283%	0.737%	0.597%	0.344%
OBV_N00103	36,779	35,617	96.84%	12.8	2.417%	1.148%	1.362%	0.708%	1.379%	1.132%	0.667%
OBV_N00104	36,709	36,165	98.52%	21.7	4.410%	2.483%	2.165%	1.380%	2.547%	2.102%	1.097%
OBV_N00105	36,550	36,507	99.88%	40.2	5.057%	3.383%	2.408%	1.441%	3.388%	2.402%	1.109%
OBV_N00106	36,131	36,119	99.97%	95.0	4.458%	1.185%	0.022%	0.011%	1.191%	0.017%	0.151%
OBV_N00107	36,077	35,943	99.63%	20.1	2.165%	0.910%	0.785%	0.317%	0.929%	0.765%	0.423%
OBV_N00113	35,147	34,768	98.92%	13.9	2.528%	1.159%	1.222%	0.489%	1.340%	1.041%	0.598%
OBV_N00119	34,805	34,671	99.61%	39.0	2.247%	0.805%	0.583%	0.415%	0.819%	0.568%	0.332%
OBV_N00126	33,814	30,601	90.50%	8.4	1.098%	0.173%	0.572%	0.095%	0.575%	0.170%	0.264%
OBV_N00127	33,791	33,544	99.27%	33.8	1.959%	0.769%	0.346%	0.107%	0.781%	0.334%	0.237%
OBV_N00129	33,553	33,413	99.58%	39.8	5.932%	3.556%	2.541%	1.410%	3.711%	2.385%	1.215%
OBV_N00135	32,884	31,546	95.93%	11.8	3.620%	2.168%	2.603%	1.474%	2.622%	2.149%	1.181%
OBV_N00145	32,025	28,975	90.48%	9.5	2.913%	1.670%	2.202%	1.401%	2.209%	1.664%	1.065%
OBV_N00149	31,630	30,661	96.94%	13.0	4.165%	2.374%	2.841%	1.539%	2.896%	2.319%	1.310%
OBV_N00152	31,315	31,315	100.00%	79.7	7.897%	5.167%	2.746%	1.597%	5.167%	2.746%	1.368%
OBV_N00155	31,018	31,004	99.95%	36.8	5.016%	2.532%	1.090%	0.203%	2.538%	1.084%	0.643%
OBV_N00160	30,759	30,679	99.74%	17.1	0.606%	0.007%	0.026%	0.000%	0.026%	0.007%	0.073%
OBV_N00163	30,613	30,129	98.42%	19.8	4.368%	2.791%	2.433%	1.394%	2.891%	2.333%	1.145%
OBV_N00182	29,541	29,470	99.76%	22.1	2.928%	1.727%	1.459%	0.960%	1.744%	1.442%	0.740%
OBV_N00186	29,180	28,226	96.73%	11.5	2.012%	1.081%	1.382%	0.850%	1.385%	1.077%	0.663%
OBV_N00191	28,525	27,982	98.10%	15.7	4.128%	2.437%	2.498%	1.476%	2.695%	2.241%	1.185%
OBV_N00390	29,867	23,156	77.53%	8.3	4.327%	2.820%	3.744%	2.414%	3.779%	2.786%	1.734%
**Median**	**37,909**	**37,529**	**99.55%**	**19.9**	**2.473%**	**1.099%**	**0.749%**	**0.322%**	**1.158%**	**0.688%**	**0.423%**

Table S1B. List of 4,240 prokaryotic dsDNA virus genomes in the viral proteomic tree (sorted in order of Fig. 1; clockwise) and corresponding genus-level gOTUs.

genome ID	source	type	genus-level gOTU	length	%G+C	name	taxonomy	taxid	host-lineage
KP791807	EBI	RVG	G1	39,142	50.5	Enterobacter phage E-4	Viruses; dsDNA viruses, no RNA stage; Caudovirales; Podoviridae.	1636315	-
NC_029102	RefSeq	RVG	G1	36,051	50.6	Enterobacter phage E-2	Viruses; dsDNA viruses, no RNA stage; Caudovirales; Podoviridae.	1636313	-
NC_028795	RefSeq	RVG	G1	31,522	50.8	Enterobacter phage E-3	Viruses; dsDNA viruses, no RNA stage; Caudovirales; Podoviridae.	1636314	-
NC_001271	RefSeq	RVG	G1	39,600	50.6	Yersinia phage phiYeO3-12	Viruses; dsDNA viruses, no RNA stage; Caudovirales; Podoviridae; Autographivirinae; T7likevirus.	110457	Bacteria; Proteobacteria; Gammaproteobacteria; Enterobacteriales; Enterobacteriaceae; Yersinia
NC_025451	RefSeq	RVG	G1	38,646	50.7	Yersinia phage vB_YenP_AP5	Viruses; dsDNA viruses, no RNA stage; Caudovirales; Podoviridae; Autographivirinae; T7likevirus.	1536611	Bacteria; Proteobacteria; Gammaproteobacteria; Enterobacteriales; Enterobacteriaceae; Yersinia
NC_010807	RefSeq	RVG	G1	38,815	50.9	Salmonella phage phiSG-JL2	Viruses; dsDNA viruses, no RNA stage; Caudovirales; Podoviridae; Autographivirinae; T7likevirus.	529929	Bacteria; Proteobacteria; Gammaproteobacteria; Enterobacteriales; Enterobacteriaceae; Salmonella; Salmonella enterica
NC_028880	RefSeq	RVG	G1	38,625	50.3	Citrobacter phage phiCFP-1	Viruses; dsDNA viruses, no RNA stage; Caudovirales; Podoviridae.	1610508	-
JX421753	EBI	RVG	G1	38,202	49.9	Enterobacteria phage T7M	Viruses; dsDNA viruses, no RNA stage; Caudovirales; Podoviridae; Autographivirinae; T7likevirus.	1075774	-
NC_003298	RefSeq	RVG	G1	38,208	49.9	Enterobacteria phage T3	Viruses; dsDNA viruses, no RNA stage; Caudovirales; Podoviridae; Autographivirinae; T7likevirus.	10759	Bacteria; Proteobacteria; Gammaproteobacteria; Enterobacteriales; Enterobacteriaceae; Escherichia
JX000007	EBI	RVG	G1	37,260	48.4	Yersinia phage R	Viruses; dsDNA viruses, no RNA stage; Caudovirales; Podoviridae; Autographivirinae; T7likevirus.	1195074	-
NC_004777	RefSeq	RVG	G1	37,555	48.3	Yersinia phage phiA1122	Viruses; dsDNA viruses, no RNA stage; Caudovirales; Podoviridae; Autographivirinae; T7likevirus.	227720	Bacteria; Proteobacteria; Gammaproteobacteria; Enterobacteriales; Enterobacteriaceae; Yersinia; Yersinia pseudotuberculosis complex
JQ957925	EBI	RVG	G1	37,432	48.3	Yersinia phage Y	Viruses; dsDNA viruses, no RNA stage; Caudovirales; Podoviridae; Autographivirinae; T7likevirus.	1176434	-
NC_001604	RefSeq	RVG	G1	39,937	48.4	Enterobacteria phage T7	Viruses; dsDNA viruses, no RNA stage; Caudovirales; Podoviridae; Autographivirinae; T7likevirus.	10760	Bacteria; Proteobacteria; Gammaproteobacteria; Enterobacteriales; Enterobacteriaceae; Escherichia
NC_011045	RefSeq	RVG	G1	38,841	48.4	Enterobacteria phage 13a	Viruses; dsDNA viruses, no RNA stage; Caudovirales; Podoviridae; Autographivirinae; T7likevirus.	532076	Bacteria; Proteobacteria; Gammaproteobacteria; Enterobacteriales; Enterobacteriaceae; Escherichia
NC_027387	RefSeq	RVG	G1	38,810	48.8	Escherichia phage CICC 80001	Viruses; dsDNA viruses, no RNA stage; Caudovirales; Podoviridae; Autographivirinae; T7likevirus.	1527506	Bacteria; Proteobacteria; Gammaproteobacteria; Enterobacteriales; Enterobacteriaceae; Escherichia
NC_015271	RefSeq	RVG	G1	38,368	48.9	Salmonella phage Vi06	Viruses; dsDNA viruses, no RNA stage; Caudovirales; Podoviridae; Autographivirinae; T7likevirus.	866889	Bacteria; Proteobacteria; Gammaproteobacteria; Enterobacteriales; Enterobacteriaceae; Salmonella; Salmonella enterica
NC_019416	RefSeq	RVG	G1	38,513	53.7	Stenotrophomonas phage IME15	Viruses; dsDNA viruses, no RNA stage; Caudovirales; Podoviridae; Autographivirinae; T7likevirus.	1239382	Bacteria; Proteobacteria; Gammaproteobacteria; Xanthomonadales; Xanthomonadaceae; Stenotrophomonas
NC_015249	RefSeq	RVG	G1	39,270	48.7	Enterobacteria phage 285P	Viruses; dsDNA viruses, no RNA stage; Caudovirales; Podoviridae; Autographivirinae; T7likevirus.	669008	Bacteria; Proteobacteria; Gammaproteobacteria; Enterobacteriales; Enterobacteriaceae; Escherichia
NC_011040	RefSeq	RVG	G1	39,816	48.8	Enterobacteria phage BA14	Viruses; dsDNA viruses, no RNA stage; Caudovirales; Podoviridae; Autographivirinae; T7likevirus.	532074	Bacteria; Proteobacteria; Gammaproteobacteria; Enterobacteriales; Enterobacteriaceae; Escherichia
NC_022744	RefSeq	RVG	G1	39,860	48.6	Erwinia phage FE44	Viruses; dsDNA viruses, no RNA stage; Caudovirales; Podoviridae; Autographivirinae; T7likevirus.	1407608	Bacteria; Proteobacteria; Gammaproteobacteria; Enterobacteriales; Enterobacteriaceae; Erwinia
NC_028822	RefSeq	RVG	G1	40,829	48.4	Escherichia phage P483	Viruses; dsDNA viruses, no RNA stage; Caudovirales; Podoviridae.	1572753	-
NC_011534	RefSeq	RVG	G1	39,472	48.6	Kluyvera phage Kvp1	Viruses; dsDNA viruses, no RNA stage; Caudovirales; Podoviridae; Autographivirinae; T7likevirus.	47049	Bacteria; Proteobacteria; Gammaproteobacteria; Enterobacteriales; Enterobacteriaceae; Kluyvera
NC_028863	RefSeq	RVG	G1	40,477	48.4	Escherichia phage P694	Viruses; dsDNA viruses, no RNA stage; Caudovirales; Podoviridae.	1572754	-
NC_008694	RefSeq	RVG	G1	38,564	47.2	Yersinia phage Berlin	Viruses; dsDNA viruses, no RNA stage; Caudovirales; Podoviridae; Autographivirinae; T7likevirus.	369257	Bacteria; Proteobacteria; Gammaproteobacteria; Enterobacteriales; Enterobacteriaceae; Yersinia; Yersinia pseudotuberculosis complex
NC_023715	RefSeq	RVG	G1	38,616	47.1	Yersinia phage Yep-phi	Viruses; dsDNA viruses, no RNA stage; Caudovirales; Podoviridae; Autographivirinae; T7likevirus.	928293	Bacteria; Proteobacteria; Gammaproteobacteria; Enterobacteriales; Enterobacteriaceae; Yersinia; Yersinia pseudotuberculosis complex
NC_011038	RefSeq	RVG	G1	38,677	47.3	Yersinia phage Yepe2	Viruses; dsDNA viruses, no RNA stage; Caudovirales; Podoviridae; Autographivirinae; T7likevirus.	532078	Bacteria; Proteobacteria; Gammaproteobacteria; Enterobacteriales; Enterobacteriaceae; Yersinia; Yersinia pseudotuberculosis complex
NC_011043	RefSeq	RVG	G1	41,181	53.2	Klebsiella phage K11	Viruses; dsDNA viruses, no RNA stage; Caudovirales; Podoviridae; Autographivirinae; T7likevirus.	532077	Bacteria; Proteobacteria; Gammaproteobacteria; Enterobacteriales; Enterobacteriaceae; Klebsiella
NC_028688	RefSeq	RVG	G1	40,114	53.3	Klebsiella phage vB_Kp1	Viruses; dsDNA viruses, no RNA stage; Caudovirales; Podoviridae; Autographivirinae; unclassified Autographivirinae.	1701804	-
NC_013647	RefSeq	RVG	G1	41,119	52.4	Klebsiella phage KP32	Viruses; dsDNA viruses, no RNA stage; Caudovirales; Podoviridae; Autographivirinae; T7likevirus.	674082	Bacteria; Proteobacteria; Gammaproteobacteria; Enterobacteriales; Enterobacteriaceae; Klebsiella
NC_028800	RefSeq	RVG	G1	41,698	52.5	Klebsiella phage K5	Viruses; dsDNA viruses, no RNA stage; Caudovirales; Podoviridae.	1647374	-
NC_015719	RefSeq	RVG	G1	40,940	51.4	Enterobacteria phage K30	Viruses; dsDNA viruses, no RNA stage; Caudovirales; Podoviridae; Autographivirinae; T7likevirus.	1041524	Bacteria; Proteobacteria; Gammaproteobacteria
NC_028977	RefSeq	RVG	G1	41,054	52.6	Klebsiella phage vB_KpnP_KpV289	Viruses; dsDNA viruses, no RNA stage; Caudovirales; Podoviridae.	1671396	-
NC_028655	RefSeq	RVG	G1	39,235	51.8	Yersinia phage vB_YenP_AP10	Viruses; dsDNA viruses, no RNA stage; Caudovirales; Podoviridae.	1735591	-
NC_028772	RefSeq	RVG	G1	39,133	51.7	Enterobacter phage phiEap-1	Viruses; dsDNA viruses, no RNA stage; Caudovirales; Podoviridae; Autographivirinae; T7likevirus.	1587520	-
NC_019510	RefSeq	RVG	G1	39,282	51.9	Erwinia phage vB_EamP-L1	Viruses; dsDNA viruses, no RNA stage; Caudovirales; Podoviridae; Autographivirinae; T7likevirus.	1051673	Bacteria; Proteobacteria; Gammaproteobacteria; Enterobacteriales; Enterobacteriaceae; Erwinia
NC_011085	RefSeq	RVG	G1	38,457	46.5	Morganella phage MmP1	Viruses; dsDNA viruses, no RNA stage; Caudovirales; Podoviridae; Autographivirinae; T7likevirus.	526118	Bacteria; Proteobacteria; Gammaproteobacteria; Enterobacteriales; Enterobacteriaceae; Morganella
NC_007456	RefSeq	RVG	G1	39,704	49.8	Enterobacteria phage K1F	Viruses; dsDNA viruses, no RNA stage; Caudovirales; Podoviridae; Autographivirinae; T7likevirus.	344021	Bacteria; Proteobacteria; Gammaproteobacteria; Enterobacteriales; Enterobacteriaceae; Escherichia
NC_024379	RefSeq	RVG	G1	39,093	49.9	Escherichia phage PE3-1	Viruses; dsDNA viruses, no RNA stage; Caudovirales; Podoviridae; Autographivirinae; unclassified Autographivirinae.	1498170	Bacteria; Proteobacteria; Gammaproteobacteria; Enterobacteriales; Enterobacteriaceae; Escherichia
NC_011042	RefSeq	RVG	G1	39,252	49.9	Enterobacteria phage EcoDS1	Viruses; dsDNA viruses, no RNA stage; Caudovirales; Podoviridae; Autographivirinae; T7likevirus.	532075	Bacteria; Proteobacteria; Gammaproteobacteria; Enterobacteriales; Enterobacteriaceae; Escherichia

NC_023576	RefSeq	RVG	G1	39,207	50.5	Citrobacter phage CR44b	Viruses; dsDNA viruses, no RNA stage; Caudovirales; Podoviridae; Autographivirinae; unclassified Autographivirinae.	1455075	Bacteria; Proteobacteria; Gammaproteobacteria; Enterobacteriales; Enterobacteriaceae; Citrobacter
NC_023558	RefSeq	RVG	G1	38,966	52.6	Cronobacter phage Dev2	Viruses; dsDNA viruses, no RNA stage; Caudovirales; Podoviridae; Autographivirinae; unclassified Autographivirinae.	1410331	Bacteria; Proteobacteria; Gammaproteobacteria; Enterobacteriales; Enterobacteriaceae; Siccibacter
NC_023548	RefSeq	RVG	G1	39,651	49.7	Citrobacter phage CR8	Viruses; dsDNA viruses, no RNA stage; Caudovirales; Podoviridae; Autographivirinae; unclassified Autographivirinae.	1455076	Bacteria; Proteobacteria; Gammaproteobacteria; Enterobacteriales; Enterobacteriaceae; Citrobacter
NC_021062	RefSeq	RVG	G1	40,192	56.2	Pseudomonas phage Phi-S1	Viruses; dsDNA viruses, no RNA stage; Caudovirales; Podoviridae; Autographivirinae; T7likevirus.	1204538	Bacteria; Proteobacteria; Gammaproteobacteria; Pseudomonadales; Pseudomonadaceae; Pseudomonas; Pseudomonas fluorescens group
NC_027292	RefSeq	RVG	G1	39,167	56.5	Pseudomonas phage Pf-10	Viruses; dsDNA viruses, no RNA stage; Caudovirales; Podoviridae; Autographivirinae; T7likevirus.	1562076	Bacteria; Proteobacteria; Gammaproteobacteria; Pseudomonadales; Pseudomonadaceae; Pseudomonas; Pseudomonas putida group
NC_015264	RefSeq	RVG	G1	40,973	56.3	Pseudomonas phage phiIBB-PF7A	Viruses; dsDNA viruses, no RNA stage; Caudovirales; Podoviridae; Autographivirinae; T7likevirus.	942165	Bacteria; Proteobacteria; Gammaproteobacteria; Pseudomonadales; Pseudomonadaceae; Pseudomonas; Pseudomonas fluorescens group
NC_015208	RefSeq	RVG	G1	39,562	58.2	Pseudomonas phage phi15	Viruses; dsDNA viruses, no RNA stage; Caudovirales; Podoviridae; Autographivirinae; T7likevirus.	988656	Bacteria; Proteobacteria; Gammaproteobacteria; Pseudomonadales; Pseudomonadaceae; Pseudomonas; Pseudomonas putida group
NC_023005	RefSeq	RVG	G1	41,386	56.8	Pseudomonas phage PPpW-4	Viruses; dsDNA viruses, no RNA stage; Caudovirales; Podoviridae.	1279083	Bacteria; Proteobacteria; Gammaproteobacteria; Pseudomonadales; Pseudomonadaceae; Pseudomonas; Pseudomonas putida group
NC_004665	RefSeq	RVG	G1	37,359	57.4	Pseudomonad phage gh-1	Viruses; dsDNA viruses, no RNA stage; Caudovirales; Podoviridae; Autographivirinae; T7likevirus.	197783	Bacteria; Proteobacteria; Gammaproteobacteria; Pseudomonadales; Pseudomonadaceae; Pseudomonas; Pseudomonas putida group
NC_024362	RefSeq	RVG	G1	40,472	57.4	Pseudomonas phage phiPSA2	Viruses; dsDNA viruses, no RNA stage; Caudovirales; Podoviridae; Autographivirinae; T7likevirus.	1500756	Bacteria; Proteobacteria; Gammaproteobacteria; Pseudomonadales; Pseudomonadaceae; Pseudomonas; Pseudomonas syringae group; Pseudomonas syringae group genomosp. 1
NC_028661	RefSeq	RVG	G1	41,149	57	Pseudomonas phage PPPL-1	Viruses; dsDNA viruses, no RNA stage; Caudovirales; Podoviridae.	1755692	-
HQ641343	EBI	RVG	G1	39,349	42.8	Vibrio phage ICP3_2008_A	Viruses; dsDNA viruses, no RNA stage; Caudovirales; Podoviridae; Autographivirinae; T7likevirus.	979537	-
NC_015159	RefSeq	RVG	G1	39,162	42.9	Vibrio phage ICP3	Viruses; dsDNA viruses, no RNA stage; Caudovirales; Podoviridae; Autographivirinae; T7likevirus.	979535	Bacteria; Proteobacteria; Gammaproteobacteria; Vibrionales; Vibrionaceae; Vibrio
HQ641344	EBI	RVG	G1	39,088	42.9	Vibrio phage ICP3_2007_A	Viruses; dsDNA viruses, no RNA stage; Caudovirales; Podoviridae; Autographivirinae; T7likevirus.	979536	-
HQ641341	EBI	RVG	G1	39,042	42.8	Vibrio phage ICP3_2009_B	Viruses; dsDNA viruses, no RNA stage; Caudovirales; Podoviridae; Autographivirinae; T7likevirus.	979539	-
NC_013651	RefSeq	RVG	G1	38,497	42.8	Vibrio phage N4	Viruses; dsDNA viruses, no RNA stage; Caudovirales; Podoviridae; Autographivirinae; T7likevirus.	644563	Bacteria; Proteobacteria; Gammaproteobacteria; Vibrionales; Vibrionaceae; Vibrio
JQ780163	EBI	RVG	G1	39,481	42.6	Vibrio phage VP3	Viruses; dsDNA viruses, no RNA stage; Caudovirales; Podoviridae; Autographivirinae; T7likevirus.	588068	-
NC_007149	RefSeq	RVG	G1	39,503	42.6	Vibriophage VP4	Viruses; dsDNA viruses, no RNA stage; Caudovirales; Podoviridae; Autographivirinae; T7likevirus.	329886	Bacteria; Proteobacteria; Gammaproteobacteria; Vibrionales; Vibrionaceae
NC_028702	RefSeq	RVG	G1	38,084	60.4	Delftia phage IME-DE1	Viruses; dsDNA viruses, no RNA stage; Caudovirales; Podoviridae.	1647385	-
NC_023736	RefSeq	RVG	G1	40,411	61.8	Ralstonia phage RSB2	Viruses; dsDNA viruses, no RNA stage; Caudovirales; Podoviridae; Autographivirinae; unclassified Autographivirinae.	913183	Bacteria; Proteobacteria; Betaproteobacteria; Burkholderiales; Burkholderiaceae; Ralstonia
TARA_ERS488673_N000385	TOV	EVG	G2	38,508	42.7	-	-	-	-
TARA_ERS490346_N000381	TOV	EVG	G2	39,064	47.1	-	-	-	-
TARA_ERS492160_N000364	TOV	EVG	G2	39,387	50.6	-	-	-	-
TARA_ERS492198_N000207	TOV	EVG	G2	38,655	54.4	-	-	-	-
TARA_ERS490494_N000198	TOV	EVG	G2	39,972	48.6	-	-	-	-
TARA_ERS489603_N000141	TOV	EVG	G2	39,641	39.9	-	-	-	-
TARA_ERS489285_N000318	TOV	EVG	G2	38,591	50.3	-	-	-	-
TARA_ERS492198_N000225	TOV	EVG	G2	37,465	49.2	-	-	-	-
TARA_ERS490346_N000398	TOV	EVG	G2	38,102	43.8	-	-	-	-
TARA_ERS478052_N000312	TOV	EVG	G2	36,909	39.6	-	-	-	-
TARA_ERS489084_N000223	TOV	EVG	G2	37,489	38.4	-	-	-	-
TARA_ERS489943_N000472	TOV	EVG	G2	35,091	51.8	-	-	-	-
TARA_ERS489084_N000191	TOV	EVG	G2	39,094	56	-	-	-	-
TARA_ERS490494_N000231	TOV	EVG	G2	38,262	37.7	-	-	-	-
TARA_ERS488673_N000369	TOV	EVG	G2	39,704	37.7	-	-	-	-
TARA_ERS489148_N000238	TOV	EVG	G2	38,340	40.2	-	-	-	-
TARA_ERS490120_N000408	TOV	EVG	G3	41,007	48.6	-	-	-	-
TARA_ERS490053_N000259	TOV	EVG	G3	41,315	48.5	-	-	-	-
TARA_ERS490026_N000130	TOV	EVG	G3	38,299	47.5	-	-	-	-
TARA_ERS488929_N000155	TOV	EVG	G3	42,207	44.3	-	-	-	-
AP013544	uvMED	EVG	G3	40,564	47.1	uvMED-CGR-U-MedDCM-OCT-S45-C18 (G8)	-	-	-
TARA_ERS490610_N000517	TOV	EVG	G3	37,978	39.7	-	-	-	-
TARA_ERS488892_N000165	TOV	EVG	G3	40,378	39	-	-	-	-
TARA_ERS488518_N000422	TOV	EVG	G3	39,795	41.4	-	-	-	-
TARA_ERS478007_N000119	TOV	EVG	G3	39,795	41.5	-	-	-	-
TARA_ERS490053_N000213	TOV	EVG	G4	45,868	44.8	-	-	-	-
TARA_ERS490120_N000351	TOV	EVG	G4	45,868	44.7	-	-	-	-
TARA_ERS489148_N000139	TOV	EVG	G4	46,796	46.5	-	-	-	-
TARA_ERS490053_N000219	TOV	EVG	G4	44,884	44	-	-	-	-
TARA_ERS490120_N000356	TOV	EVG	G4	44,884	44	-	-	-	-
TARA_ERS488448_N000369	TOV	EVG	G5	39,372	37.7	-	-	-	-
TARA_ERS478052_N000257	TOV	EVG	G5	40,639	37.7	-	-	-	-
TARA_ERS488518_N000405	TOV	EVG	G5	40,690	37.8	-	-	-	-
AP013542	uvMED	EVG	G5	42,564	33.8	uvMED-CGR-U-MedDCM-OCT-S35-C6 (G8)	-	-	-

NC_020483	RefSeq	RVG	G5	42,084	34	Pelagibacter phage HTVC019P	Viruses; dsDNA viruses, no RNA stage; Caudovirales; Podoviridae.	1283079	Bacteria; Proteobacteria; Alphaproteobacteria; Pelagibacterales; Pelagibacteraceae; Candidatus Pelagibacter
AP013541	uvMED	EVG	G5	42,399	33.9	uvMED-CGR-C97-MedDCM-OCT-S42-C7 (G8)	-	-	-
OBV_N00073	OBV	EVG	G5	42,216	34	-	-	-	-
AP013545	uvMED	EVG	G5	41,803	33.8	uvMED-CGR-U-MedDCM-OCT-S46-C10 (G8)	-	-	-
AP013540	uvMED	EVG	G5	40,866	32.7	uvMED-CGR-C62A-MedDCM-OCT-S28-C10 (G8)	-	-	-
AP013543	uvMED	EVG	G5	37,553	32.4	uvMED-CGR-U-MedDCM-OCT-S30-C28 (G8)	-	-	-
NC_020482	RefSeq	RVG	G5	39,921	32	Pelagibacter phage HTVC011P	Viruses; dsDNA viruses, no RNA stage; Caudovirales; Podoviridae.	1283078	Bacteria; Proteobacteria; Alphaproteobacteria; Pelagibacterales; Pelagibacteraceae; Candidatus Pelagibacter
TARA_ERS492198_N000194	TOV	EVG	G5	39,359	42.2	-	-	-	-
TARA_ERS488448_N000342	TOV	EVG	G6	40,823	43.8	-	-	-	-
TARA_ERS488499_N000203	TOV	EVG	G6	40,853	43.8	-	-	-	-
TARA_ERS488499_N000221	TOV	EVG	G6	39,734	46.2	-	-	-	-
TARA_ERS488757_N000078	TOV	EVG	G6	39,716	42.8	-	-	-	-
TARA_ERS488929_N000162	TOV	EVG	G6	41,420	35.7	-	-	-	-
TARA_ERS478052_N000285	TOV	EVG	G6	38,966	35.5	-	-	-	-
JX483873	EBI	RVG	G7	43,444	59.2	Rhizobium phage RHEph01	Viruses; dsDNA viruses, no RNA stage; Caudovirales; Podoviridae.	1220601	-
NC_025431	RefSeq	RVG	G7	45,718	61.1	Mesorhizobium phagevB_MloP_Lo5R7ANS	Viruses; dsDNA viruses, no RNA stage; Caudovirales; Podoviridae.	1527771	Bacteria; Proteobacteria; Alphaproteobacteria; Rhizobiales; Phyllobacteriaceae; Mesorhizobium
TARA_ERS478007_N000135	TOV	EVG	G8	38,450	36.3	-	-	-	-
TARA_ERS490026_N000126	TOV	EVG	G9	38,796	56	-	-	-	-
TARA_ERS488448_N000279	TOV	EVG	G10	44,255	58	-	-	-	-
TARA_ERS490557_N000318	TOV	EVG	G11	38,767	32.5	-	-	-	-
TARA_ERS490452_N000261	TOV	EVG	G11	39,160	32.5	-	-	-	-
TARA_ERS488673_N000377	TOV	EVG	G11	38,954	33.1	-	-	-	-
TARA_ERS489113_N000219	TOV	EVG	G12	39,850	51.4	-	-	-	-
TARA_ERS490120_N000420	TOV	EVG	G13	40,618	63.1	-	-	-	-
TARA_ERS490053_N000266	TOV	EVG	G13	40,638	63.1	-	-	-	-
NC_020865	RefSeq	RVG	G14	45,730	47.4	Cyanophage KBS-P-1A	Viruses; dsDNA viruses, no RNA stage; unclassified dsDNA phages.	889951	Bacteria; Cyanobacteria; Oscillatoriophycideae; Chroococcales; Synechococcus
NC_020838	RefSeq	RVG	G14	45,728	47.3	Synechococcus phage S-RIP2	Viruses; dsDNA viruses, no RNA stage; Caudovirales; Podoviridae.	754040	Bacteria; Cyanobacteria; Oscillatoriophycideae; Chroococcales; Synechococcus
AP013538	uvMED	EVG	G14	44,705	45.3	uvMED-CGR-U-MedDCM-OCT-S45-C4 (G7)	-	-	-
NC_020867	RefSeq	RVG	G14	44,892	42.9	Synechococcus phage S-RIP1	Viruses; dsDNA viruses, no RNA stage; Caudovirales; Podoviridae.	754041	Bacteria; Cyanobacteria; Oscillatoriophycideae; Chroococcales; Synechococcus
AP013539	uvMED	EVG	G14	43,444	46.7	uvMED-CGR-U-MedDCM-OCT-S28-C3 (G7)	-	-	-
TARA_ERS490026_N000066	TOV	EVG	G14	46,040	44.1	-	-	-	-
TARA_ERS489943_N000335	TOV	EVG	G14	43,667	42.9	-	-	-	-
NC_025464	RefSeq	RVG	G14	44,147	44.4	Synechococcus phage S-CBP4	Viruses; dsDNA viruses, no RNA stage; Caudovirales; Podoviridae.	754059	Bacteria; Cyanobacteria; Oscillatoriophycideae; Chroococcales; Synechococcus
NC_025461	RefSeq	RVG	G14	45,871	46.9	Synechococcus phage S-CBP3	Viruses; dsDNA viruses, no RNA stage; unclassified dsDNA phages.	756276	Bacteria; Cyanobacteria; Oscillatoriophycideae; Chroococcales; Synechococcus
NC_025456	RefSeq	RVG	G14	46,547	47.6	Synechococcus phage S-CBP1	Viruses; dsDNA viruses, no RNA stage; Caudovirales; Podoviridae; Autographivirinae; unclassified Autographivirinae.	1273711	Bacteria; Cyanobacteria; Oscillatoriophycideae; Chroococcales; Synechococcus
TARA_ERS478007_N000062	TOV	EVG	G14	47,397	45	-	-	-	-
HQ634152	EBI	RVG	G14	47,039	39.2	Prochlorococcus phage P-SSP6	Viruses; dsDNA viruses, no RNA stage; Caudovirales; Podoviridae; Autographivirinae; unclassified Autographivirinae.	382275	-
NC_016657	RefSeq	RVG	G14	47,055	39.2	Cyanophage 9515-10a	Viruses; dsDNA viruses, no RNA stage; Caudovirales; Podoviridae; Autographivirinae; unclassified Autographivirinae.	444875	Bacteria; Cyanobacteria; Prochlorales; Prochlorococcaceae; Prochlorococcus
NC_020835	RefSeq	RVG	G14	47,325	39.2	Prochlorococcus phage P-SSP10	Viruses; dsDNA viruses, no RNA stage; Caudovirales; Podoviridae.	885867	Bacteria; Cyanobacteria; Prochlorales; Prochlorococcaceae; Prochlorococcus
NC_016659	RefSeq	RVG	G14	47,536	39.9	Cyanophage NATL2A-133	Viruses; dsDNA viruses, no RNA stage; Caudovirales; Podoviridae; Autographivirinae; unclassified Autographivirinae.	445692	Bacteria; Cyanobacteria; Prochlorales; Prochlorococcaceae; Prochlorococcus
JF974297	CAMERA	RVG	G14	32,402	42.9	Cyanophage KBS-S-1A	Viruses; dsDNA viruses, no RNA stage; unclassified dsDNA phages.	889952	-
NC_020874	RefSeq	RVG	G14	46,198	37.9	Prochlorococcus phage P-SSP3	Viruses; dsDNA viruses, no RNA stage; unclassified dsDNA phages.	382273	Bacteria; Cyanobacteria; Prochlorales; Prochlorococcaceae; Prochlorococcus
NC_016656	RefSeq	RVG	G14	45,890	37.9	Cyanophage P-SSP2	Viruses; dsDNA viruses, no RNA stage; Caudovirales.	444876	Bacteria; Cyanobacteria; Oscillatoriophycideae; Chroococcales; Synechococcus
NC_006882	RefSeq	RVG	G14	45,176	38.8	Prochlorococcus phage P-SSP7	Viruses; dsDNA viruses, no RNA stage; Caudovirales; Podoviridae; Autographivirinae; unassigned Autographivirinae.	268748	Bacteria; Cyanobacteria; Prochlorales; Prochlorococcaceae
NC_020878	RefSeq	RVG	G14	44,945	39.6	Prochlorococcus phage P-GSP1	Viruses; dsDNA viruses, no RNA stage; unclassified dsDNA phages.	382262	Bacteria; Cyanobacteria; Prochlorales; Prochlorococcaceae; Prochlorococcus; Prochlorococcus marinus
NC_016658	RefSeq	RVG	G14	47,741	38.7	Cyanophage NATL1A-7	Viruses; dsDNA viruses, no RNA stage; Caudovirales; Podoviridae; Autographivirinae; unclassified Autographivirinae.	445693	Bacteria; Cyanobacteria; Prochlorales; Prochlorococcaceae; Prochlorococcus
NC_020872	RefSeq	RVG	G14	46,997	40.5	Cyanophage SS120-1	Viruses; dsDNA viruses, no RNA stage; Caudovirales.	616674	Bacteria; Cyanobacteria; Prochlorales; Prochlorococcaceae; Prochlorococcus; Prochlorococcus marinus
NC_009531	RefSeq	RVG	G14	46,214	55	Synechococcus phage Syn5	Viruses; dsDNA viruses, no RNA stage; Caudovirales; Podoviridae; Autographivirinae; unassigned Autographivirinae.	438482	Bacteria; Cyanobacteria; Oscillatoriophycideae; Chroococcales; Synechococcus
TARA_ERS490142_N000309	TOV	EVG	G14	45,651	45.6	-	-	-	-
TARA_ERS490120_N000335	TOV	EVG	G14	47,070	44.7	-	-	-	-
NC_029031	RefSeq	RVG	G14	45,218	54.6	Synechococcus phage S-CBP42	Viruses; dsDNA viruses, no RNA stage; Caudovirales; Podoviridae.	461711	-
NC_003390	RefSeq	RVG	G14	46,675	53.3	Synechococcus phage P60	Viruses; dsDNA viruses, no RNA stage; Caudovirales; Podoviridae; Autographivirinae; unassigned Autographivirinae.	151528	Bacteria; Cyanobacteria; Oscillatoriophycideae; Chroococcales; Synechococcus
NC_025455	RefSeq	RVG	G14	46,237	55	Synechococcus phage S-CBP2	Viruses; dsDNA viruses, no RNA stage; unclassified dsDNA phages.	756277	Bacteria; Cyanobacteria; Oscillatoriophycideae; Chroococcales; Synechococcus
HQ332139	CAMERA	RVG	G15	42,257	34	Prochlorococcus phage P-RSP2	Viruses; dsDNA viruses, no RNA stage; unclassified dsDNA phages.	756283	-
TARA_ERS488558_N000636	TOV	EVG	G15	41,574	34.1	-	-	-	-
NC_024329	RefSeq	RVG	G16	39,425	34.7	Podovirus Lau218	Viruses; dsDNA viruses, no RNA stage; Caudovirales; Podoviridae.	1465639	-
AP013546	uvMED	EVG	G17	42,127	54.7	uvMED-CGR-C79-MedDCM-OCT-S37-C6 (G9)	-	-	-
TARA_ERS488737_N000220	TOV	EVG	G17	40,574	55.2	-	-	-	-
TARA_ERS489084_N000181	TOV	EVG	G17	39,711	37.4	-	-	-	-
AP013547	uvMED	EVG	G17	43,552	56.2	uvMED-CGR-U-MedDCM-OCT-S31-C1 (G9)	-	-	-
AP013548	uvMED	EVG	G17	43,698	57.1	uvMED-CGR-U-MedDCM-OCT-S38-C3 (G9)	-	-	-

AP013549	uvMED	EVG	G18	41,987	51.6	uvMED-CGR-U-MedDCM-OCT-S39-C11 (G9)	-	-	-
TARA_ERS489084_N000236	TOV	EVG	G19	36,587	57.3	-	-	-	-
TARA_ERS478052_N000106	TOV	EVG	G20	62,883	34.7	-	-	-	-
TARA_ERS488448_N000097	TOV	EVG	G20	66,644	38.8	-	-	-	-
TARA_ERS489603_N000030	TOV	EVG	G20	64,959	42.6	-	-	-	-
TARA_ERS490346_N000151	TOV	EVG	G20	65,228	39.8	-	-	-	-
TARA_ERS489148_N000041	TOV	EVG	G20	66,637	45	-	-	-	-
TARA_ERS490346_N000139	TOV	EVG	G20	67,106	41.8	-	-	-	-
TARA_ERS490142_N000165	TOV	EVG	G21	69,080	40.2	-	-	-	-
TARA_ERS490180_N000144	TOV	EVG	G21	69,173	40.2	-	-	-	-
TARA_ERS478007_N000080	TOV	EVG	G22	44,233	52.7	-	-	-	-
TARA_ERS490953_N000111	TOV	EVG	G23	40,645	49.8	-	-	-	-
TARA_ERS492198_N000173	TOV	EVG	G23	41,493	45.8	-	-	-	-
OBV_N00085	OBV	EVG	G23	40,177	44	-	-	-	-
TARA_ERS478007_N000114	TOV	EVG	G23	40,297	48.7	-	-	-	-
TARA_ERS488448_N000293	TOV	EVG	G23	43,594	44.1	-	-	-	-
TARA_ERS491107_N000112	TOV	EVG	G24	55,900	41	-	-	-	-
TARA_ERS490494_N000221	TOV	EVG	G25	38,672	39.1	-	-	-	-
TARA_ERS489084_N000144	TOV	EVG	G25	42,621	59.7	-	-	-	-
TARA_ERS489084_N000147	TOV	EVG	G25	42,370	44.5	-	-	-	-
NC_013649	RefSeq	RVG	G26	43,809	54.1	Klebsiella phage KP34	Viruses; dsDNA viruses, no RNA stage; Caudovirales; Podoviridae.	674081	Bacteria; Proteobacteria; Gammaproteobacteria; Enterobacteriales; Enterobacteriaceae; Klebsiella
NC_028816	RefSeq	RVG	G26	43,809	53.7	Klebsiella phage vB_KpnP_SU503	Viruses; dsDNA viruses, no RNA stage; Caudovirales; Podoviridae; Autographivirinae; unclassified Autographivirinae.	1610834	-
NC_028670	RefSeq	RVG	G26	44,203	54	Klebsiella phage vB_KpnP_KpV41	Viruses; dsDNA viruses, no RNA stage; Caudovirales; Podoviridae.	1747282	-
NC_025418	RefSeq	RVG	G26	43,871	54.2	Klebsiella phage NTUH-K2044-K1-1	Viruses; dsDNA viruses, no RNA stage; Caudovirales; Podoviridae.	1194091	Bacteria; Proteobacteria; Gammaproteobacteria; Enterobacteriales; Enterobacteriaceae; Klebsiella; Klebsiella pneumoniae
NC_028870	RefSeq	RVG	G26	43,595	54.2	Klebsiella phage vB_KpnP_SU552A	Viruses; dsDNA viruses, no RNA stage; Caudovirales; Podoviridae; Autographivirinae; unclassified Autographivirinae.	1610835	-
NC_028664	RefSeq	RVG	G26	43,963	53.8	Klebsiella phage vB_Kp2	Viruses; dsDNA viruses, no RNA stage; Caudovirales; Podoviridae; Autographivirinae; unclassified Autographivirinae.	1701805	-
NC_023567	RefSeq	RVG	G26	43,766	53.8	Klebsiella phage F19	Viruses; dsDNA viruses, no RNA stage; Caudovirales; Podoviridae.	1416011	Bacteria; Proteobacteria; Gammaproteobacteria; Enterobacteriales; Enterobacteriaceae; Klebsiella
JQ267518	EBI	RVG	G26	43,804	51.7	Enterobacter phage phiKDA1	Viruses; dsDNA viruses, no RNA stage; Caudovirales; Podoviridae; Autographivirinae; unclassified phiKMV-like phages.	1147139	-
NC_027342	RefSeq	RVG	G26	41,268	41.4	Proteus phage PM16	Viruses; dsDNA viruses, no RNA stage; Caudovirales; Podoviridae.	1357704	Bacteria; Proteobacteria; Gammaproteobacteria; Enterobacteriales; Enterobacteriaceae; Proteus
NC_027363	RefSeq	RVG	G26	41,480	41.6	Proteus phage PM 75	Viruses; dsDNA viruses, no RNA stage; Caudovirales; Podoviridae.	1560282	Bacteria; Proteobacteria; Gammaproteobacteria; Enterobacteriales; Enterobacteriaceae; Proteus
NC_019454	RefSeq	RVG	G26	44,546	54	Pantoea phage LIMElight	Viruses; dsDNA viruses, no RNA stage; Caudovirales; Podoviridae; Autographivirinae.	881915	Bacteria; Proteobacteria; Gammaproteobacteria; Enterobacteriales; Enterobacteriaceae; Pantoea; Pantoea agglomerans group
NC_012662	RefSeq	RVG	G26	43,931	49.1	Vibrio phage VP93	Viruses; dsDNA viruses, no RNA stage; Caudovirales; Podoviridae; Autographivirinae; unclassified phiKMV-like phages.	641832	Bacteria; Proteobacteria; Gammaproteobacteria; Vibrionales; Vibrionaceae; Vibrio; Vibrio harveyi group
NC_029081	RefSeq	RVG	G26	45,633	48.9	Pectobacterium phage Peat1	Viruses; dsDNA viruses, no RNA stage; Caudovirales; Podoviridae.	1654601	-
NC_021316	RefSeq	RVG	G27	42,185	39.2	Acinetobacter phage Abp1	Viruses; dsDNA viruses, no RNA stage; Caudovirales; Podoviridae.	1235824	Bacteria; Proteobacteria; Gammaproteobacteria; Pseudomonadales; Moraxellaceae; Acinetobacter; Acinetobacter calcoaceticus/baumannii complex
NC_028675	RefSeq	RVG	G27	41,526	39.1	Acinetobacter phage phiAB1	Viruses; dsDNA viruses, no RNA stage; Caudovirales; Podoviridae.	691318	-
NC_028684	RefSeq	RVG	G27	41,563	39.5	Acinetobacter phage vB_AbaP_PD-6A3	Viruses; dsDNA viruses, no RNA stage; Caudovirales; Podoviridae.	1701807	-
NC_028679	RefSeq	RVG	G27	40,938	39.3	Acinetobacter phage vB_AbaP_PD-AB9	Viruses; dsDNA viruses, no RNA stage; Caudovirales; Podoviridae.	1701808	-
NC_028848	RefSeq	RVG	G27	41,805	39.3	Acinetobacter phage Fri1	Viruses; dsDNA viruses, no RNA stage; Caudovirales; Podoviridae.	1647373	-
NC_021337	RefSeq	RVG	G27	31,185	39.2	Acinetobacter phage AB3	Viruses; dsDNA viruses, no RNA stage; Caudovirales; Podoviridae.	1273713	Bacteria; Proteobacteria; Gammaproteobacteria; Pseudomonadales; Moraxellaceae; Acinetobacter; Acinetobacter calcoaceticus/baumannii complex
NC_028987	RefSeq	RVG	G27	41,243	39.3	Acinetobacter phage IME-200	Viruses; dsDNA viruses, no RNA stage; Caudovirales; Podoviridae.	1735582	-
NC_025457	RefSeq	RVG	G27	42,654	41.2	Acinetobacter phage vB_AbaP_Acibel007	Viruses; dsDNA viruses, no RNA stage; Caudovirales; Podoviridae.	1481187	Bacteria; Proteobacteria; Gammaproteobacteria; Pseudomonadales; Moraxellaceae; Acinetobacter; Acinetobacter calcoaceticus/baumannii complex
NC_023570	RefSeq	RVG	G27	40,739	42.2	Acinetobacter phage Petty	Viruses; dsDNA viruses, no RNA stage; Caudovirales; Podoviridae; Autographivirinae; unclassified phiKMV-like phages.	1406779	Bacteria; Proteobacteria; Gammaproteobacteria; Pseudomonadales; Moraxellaceae; Acinetobacter; Acinetobacter calcoaceticus/baumannii complex
EU056923	EBI	RVG	G28	42,954	62.3	Pseudomonas phage PT5	Viruses; dsDNA viruses, no RNA stage; Caudovirales; Podoviridae; Autographivirinae; unclassified phiKMV-like phages.	476523	-
NC_017865	RefSeq	RVG	G28	42,966	62.3	Pseudomonas phage vB_Pae-TbilisiM32	Viruses; dsDNA viruses, no RNA stage; Caudovirales; Podoviridae; Autographivirinae; unclassified phiKMV-like phages.	1141525	Bacteria; Proteobacteria; Gammaproteobacteria; Pseudomonadales; Pseudomonadaceae; Pseudomonas; Pseudomonas aeruginosa group
LN610580	EBI	RVG	G28	42,750	62.2	Pseudomonas phage vB_PaeP_PAO1_1-15pyo	Viruses; dsDNA viruses, no RNA stage; Caudovirales; Podoviridae; Autographivirinae; unclassified phiKMV-like phages.	1548908	-
KC969441	EBI	RVG	G28	42,519	62.1	Pseudomonas phage MBL	Viruses; dsDNA viruses, no RNA stage; Caudovirales; Podoviridae; Autographivirinae; T7likevirus.	1327768	-
NC_011107	RefSeq	RVG	G28	42,961	62.2	Pseudomonas phage PT2	Viruses; dsDNA viruses, no RNA stage; Caudovirales; Podoviridae; Autographivirinae; T7likevirus.	496396	Bacteria; Proteobacteria; Gammaproteobacteria; Pseudomonadales; Pseudomonadaceae; Pseudomonas; Pseudomonas aeruginosa group
NC_005045	RefSeq	RVG	G28	42,519	62.3	Pseudomonas phage phiKMV	Viruses; dsDNA viruses, no RNA stage; Caudovirales; Podoviridae; Autographivirinae.	204270	Bacteria; Proteobacteria; Gammaproteobacteria; Pseudomonadales; Pseudomonadaceae; Pseudomonas; Pseudomonas aeruginosa group
NC_010326	RefSeq	RVG	G28	43,548	62.3	Pseudomonas phage LUZ19	Viruses; dsDNA viruses, no RNA stage; Caudovirales; Podoviridae; Autographivirinae; unclassified phiKMV-like phages.	484896	Bacteria; Proteobacteria; Gammaproteobacteria; Pseudomonadales; Pseudomonadaceae; Pseudomonas; Pseudomonas aeruginosa group
NC_022746	RefSeq	RVG	G28	42,957	62.3	Pseudomonas phage MPK6	Viruses; dsDNA viruses, no RNA stage; Caudovirales; Podoviridae; Autographivirinae; unclassified phiKMV-like phages.	1262514	Bacteria; Proteobacteria; Gammaproteobacteria; Pseudomonadales; Pseudomonadaceae; Pseudomonas; Pseudomonas aeruginosa group
NC_028836	RefSeq	RVG	G28	42,508	62.2	Pseudomonas phage DL62	Viruses; dsDNA viruses, no RNA stage; Caudovirales; Podoviridae.	1640972	-

NC_027375	RefSeq	RVG	G28	43,227	62.4	Pseudomonas phage vB_PaeP_PPA-ABTNL	Viruses; dsDNA viruses, no RNA stage; Caudovirales; Podoviridae; Autographivirinae; unclassified phiKMV-like phages.	1527525	Bacteria; Proteobacteria; Gammaproteobacteria; Pseudomonadales; Pseudomonadaceae; Pseudomonas; Pseudomonas aeruginosa group
NC_022091	RefSeq	RVG	G28	42,874	62.1	Pseudomonas phage MPK7	Viruses; dsDNA viruses, no RNA stage; Caudovirales; Podoviridae; Autographivirinae.	1225790	Bacteria; Proteobacteria; Gammaproteobacteria; Pseudomonadales; Pseudomonadaceae; Pseudomonas; Pseudomonas aeruginosa group
NC_026602	RefSeq	RVG	G28	43,639	62.3	Pseudomonas phage vB_PaeP_PAO1_Ab05	Viruses; dsDNA viruses, no RNA stage; Caudovirales; Podoviridae.	1548902	Bacteria; Proteobacteria; Gammaproteobacteria; Pseudomonadales; Pseudomonadaceae; Pseudomonas; Pseudomonas aeruginosa group
NC_012418	RefSeq	RVG	G28	43,152	62.9	Pseudomonas phage phikF77	Viruses; dsDNA viruses, no RNA stage; Caudovirales; Podoviridae; Autographivirinae; unclassified phiKMV-like phages.	627480	Bacteria; Proteobacteria; Gammaproteobacteria; Pseudomonadales; Pseudomonadaceae; Pseudomonas; Pseudomonas aeruginosa group
NC_009935	RefSeq	RVG	G28	43,200	62.3	Pseudomonas phage LKD16	Viruses; dsDNA viruses, no RNA stage; Caudovirales; Podoviridae; Autographivirinae; unclassified phiKMV-like phages.	386792	Bacteria; Proteobacteria; Gammaproteobacteria; Pseudomonadales; Pseudomonadaceae; Pseudomonas; Pseudomonas aeruginosa group
NC_013638	RefSeq	RVG	G28	43,144	58.9	Pseudomonas phage phi-2	Viruses; dsDNA viruses, no RNA stage; Caudovirales; Podoviridae; Autographivirinae; unclassified phiKMV-like phages.	693582	Bacteria; Proteobacteria; Gammaproteobacteria; Pseudomonadales; Pseudomonadaceae; Pseudomonas; Pseudomonas fluorescens group
NC_009936	RefSeq	RVG	G28	41,593	60.9	Pseudomonas phage LKA1	Viruses; dsDNA viruses, no RNA stage; Caudovirales; Podoviridae; Autographivirinae.	386793	Bacteria; Proteobacteria; Gammaproteobacteria; Pseudomonadales; Pseudomonadaceae; Pseudomonas; Pseudomonas aeruginosa group
NC_022917	RefSeq	RVG	G29	44,578	61	Ralstonia phage RSB3	Viruses; dsDNA viruses, no RNA stage; Caudovirales; Podoviridae; Autographivirinae; unclassified phiKMV-like phages.	1402875	Bacteria; Proteobacteria; Betaproteobacteria; Burkholderiales; Burkholderiaceae; Ralstonia
NC_015585	RefSeq	RVG	G30	43,032	55.4	Pantoea phage LIMEzero	Viruses; dsDNA viruses, no RNA stage; Caudovirales; Podoviridae; Autographivirinae.	943335	Bacteria; Proteobacteria; Gammaproteobacteria; Enterobacteriales; Enterobacteriaceae; Pantoea; Pantoea agglomerans group
NC_025445	RefSeq	RVG	G30	40,981	55.7	Enterobacteria phage J8-65	Viruses; dsDNA viruses, no RNA stage; Caudovirales; Podoviridae.	1536597	Bacteria; Proteobacteria; Gammaproteobacteria; Enterobacteriales; Enterobacteriaceae; Escherichia
HG796221	EBI	RVG	G31	42,112	61.8	Burkholderia phage Bp-AMP4	Viruses; dsDNA viruses, no RNA stage; Caudovirales; Podoviridae; Autographivirinae; unclassified Autographivirinae.	1437329	-
HG796220	EBI	RVG	G31	41,882	61.8	Burkholderia phage Bp-AMP3	Viruses; dsDNA viruses, no RNA stage; Caudovirales; Podoviridae; Autographivirinae; unclassified Autographivirinae.	1673729	-
HG796219	EBI	RVG	G31	42,492	61.8	Burkholderia phage Bp-AMP2	Viruses; dsDNA viruses, no RNA stage; Caudovirales; Podoviridae; Autographivirinae; unclassified Autographivirinae.	1437328	-
HG793132	EBI	RVG	G31	42,409	61.7	Burkholderia phage Bp-AMP1	Viruses; dsDNA viruses, no RNA stage; Caudovirales; Podoviridae; Autographivirinae; unclassified Autographivirinae.	1432428	-
NC_011201	RefSeq	RVG	G31	43,079	61.7	Ralstonia phage RSB1	Viruses; dsDNA viruses, no RNA stage; Caudovirales; Podoviridae; Autographivirinae; unclassified phiKMV-like phages.	551790	Bacteria; Proteobacteria; Betaproteobacteria; Burkholderiales; Burkholderiaceae; Ralstonia
NC_029007	RefSeq	RVG	G31	43,745	60.7	Ralstonia phage RSJ5	Viruses; dsDNA viruses, no RNA stage; Caudovirales; Podoviridae.	1538364	-
NC_028988	RefSeq	RVG	G31	44,360	60.9	Ralstonia phage RSJ2	Viruses; dsDNA viruses, no RNA stage; Caudovirales; Podoviridae.	1481785	-
NC_022916	RefSeq	RVG	G31	41,604	60.7	Burkholderia phage JG068	Viruses; dsDNA viruses, no RNA stage; Caudovirales; Podoviridae; Autographivirinae; unclassified phiKMV-like phages.	1401297	Bacteria; Proteobacteria; Betaproteobacteria; Burkholderiales; Burkholderiaceae; Burkholderia; Burkholderia cepacia complex
NC_016764	RefSeq	RVG	G31	40,058	58.4	Pseudomonas phage Bf7	Viruses; dsDNA viruses, no RNA stage; Caudovirales; Podoviridae.	1100790	Bacteria; Proteobacteria; Gammaproteobacteria; Pseudomonadales; Pseudomonadaceae; Pseudomonas; Pseudomonas fluorescens group
NC_029092	RefSeq	RVG	G31	44,974	60.9	Caulobacter phage Percy	Viruses; dsDNA viruses, no RNA stage; Caudovirales; Podoviridae.	1701809	-
NC_022987	RefSeq	RVG	G31	43,940	63	Xylella phage Prado	Viruses; dsDNA viruses, no RNA stage; Caudovirales; Podoviridae; Autographivirinae; unclassified phiKMV-like phages.	1415146	Bacteria; Proteobacteria; Gammaproteobacteria; Xanthomonadales; Xanthomonadaceae; Xylella
NC_022982	RefSeq	RVG	G31	43,869	60.2	Xylella phage Paz	Viruses; dsDNA viruses, no RNA stage; Caudovirales; Podoviridae; Autographivirinae; unclassified phiKMV-like phages.	1415145	Bacteria; Proteobacteria; Gammaproteobacteria; Xanthomonadales; Xanthomonadaceae; Xylella
NC_019520	RefSeq	RVG	G32	42,608	51.6	Escherichia phage phiKT	Viruses; dsDNA viruses, no RNA stage; Caudovirales; Podoviridae; Autographivirinae; unclassified phiKMV-like phages.	1141519	Bacteria; Proteobacteria; Gammaproteobacteria; Enterobacteriales; Enterobacteriaceae
HE956707	EBI	RVG	G33	41,700	51.8	Yersinia phage phiR8-01	Viruses; dsDNA viruses, no RNA stage; Caudovirales; Podoviridae; Autographivirinae; unclassified Autographivirinae.	1206556	-
NC_028850	RefSeq	RVG	G33	41,449	53.8	Yersinia phage vB_YenP_ISAO8	Viruses; dsDNA viruses, no RNA stage; Caudovirales; Podoviridae.	1675027	-
NC_019528	RefSeq	RVG	G33	41,572	56.9	Aeromonas phage phiAS7	Viruses; dsDNA viruses, no RNA stage; Caudovirales; Podoviridae; Autographivirinae; unassigned Autographivirinae.	1141132	Bacteria; Proteobacteria; Gammaproteobacteria; Aeromonadales; Aeromonadaceae; Aeromonas; Aeromonas salmonicida
NC_020078	RefSeq	RVG	G33	41,796	55.7	Cronobacter phage vB_CskP_GAP227	Viruses; dsDNA viruses, no RNA stage; Caudovirales; Podoviridae; Autographivirinae; unclassified phiKMV-like phages.	1264737	Bacteria; Proteobacteria; Gammaproteobacteria; Enterobacteriales; Enterobacteriaceae; Cronobacter
NC_029070	RefSeq	RVG	G33	41,620	53.8	Cronobacter phage Dev-CD-23823	Viruses; dsDNA viruses, no RNA stage; Caudovirales; Podoviridae.	1712539	-
NC_019911	RefSeq	RVG	G33	42,081	47.6	Yersinia phage phi80-18	Viruses; dsDNA viruses, no RNA stage; Caudovirales; Podoviridae; Autographivirinae; unclassified Autographivirinae.	1206559	Bacteria; Proteobacteria; Gammaproteobacteria; Enterobacteriales; Enterobacteriaceae; Yersinia
JX483880	EBI	RVG	G34	45,962	49.3	Rhizobium phage RHEph09	Viruses; dsDNA viruses, no RNA stage; Caudovirales; Podoviridae.	1220716	-
JX483879	EBI	RVG	G34	43,619	49.4	Rhizobium phage RHEph08	Viruses; dsDNA viruses, no RNA stage; Caudovirales; Podoviridae.	1220715	-
JX483875	EBI	RVG	G34	45,912	49.3	Rhizobium phage RHEph03	Viruses; dsDNA viruses, no RNA stage; Caudovirales; Podoviridae.	1220603	-
JX483874	EBI	RVG	G34	46,486	49.4	Rhizobium phage RHEph02	Viruses; dsDNA viruses, no RNA stage; Caudovirales; Podoviridae.	1220602	-
TARA_ERS488499_N000151	TOV	EVG	G35	47,724	42.6	-	-	-	-
TARA_ERS490953_N000074	TOV	EVG	G35	48,271	41.7	-	-	-	-
NC_009543	RefSeq	RVG	G36	44,520	51.9	Xanthomonas phage Xop411	Viruses; dsDNA viruses, no RNA stage; Caudovirales; Siphoviridae; Xp10likevirus.	470314	Bacteria; Proteobacteria; Gammaproteobacteria; Xanthomonadales; Xanthomonadaceae; Xanthomonas
NC_004902	RefSeq	RVG	G36	44,373	52	Xanthomonas phage Xp10	Viruses; dsDNA viruses, no RNA stage; Caudovirales; Siphoviridae; Xp10likevirus.	232237	Bacteria; Proteobacteria; Gammaproteobacteria; Xanthomonadales; Xanthomonadaceae; Xanthomonas
NC_007709	RefSeq	RVG	G36	43,785	51.1	Xanthomonas phage OP1	Viruses; dsDNA viruses, no RNA stage; Caudovirales; Siphoviridae; Xp10likevirus.	329254	Bacteria; Proteobacteria; Gammaproteobacteria; Xanthomonadales; Xanthomonadaceae; Xanthomonas
NC_012742	RefSeq	RVG	G36	44,080	55.6	Xanthomonas phage phiL7	Viruses; dsDNA viruses, no RNA stage; Caudovirales; Siphoviridae; Xp10likevirus.	538979	Bacteria; Proteobacteria; Gammaproteobacteria; Xanthomonadales; Xanthomonadaceae; Xanthomonas
NC_019933	RefSeq	RVG	G36	43,870	53.3	Xanthomonas phage CP1	Viruses; dsDNA viruses, no RNA stage; Caudovirales; Siphoviridae; Xp10likevirus.	1188794	Bacteria; Proteobacteria; Gammaproteobacteria; Xanthomonadales; Xanthomonadaceae; Xanthomonas; Xanthomonas citri group
NC_019403	RefSeq	RVG	G37	43,731	45.3	Enterobacteria phage vB_EcoP_ACG-C91	Viruses; dsDNA viruses, no RNA stage; Caudovirales; Podoviridae; Autographivirinae; unclassified Sp6likevirus.	1141139	Bacteria; Proteobacteria; Gammaproteobacteria; Enterobacteriales; Enterobacteriaceae; Escherichia
NC_007637	RefSeq	RVG	G37	45,251	45.1	Enterobacteria phage K1E	Viruses; dsDNA viruses, no RNA stage; Caudovirales; Podoviridae; Autographivirinae.	344022	Bacteria; Proteobacteria; Gammaproteobacteria; Enterobacteriales; Enterobacteriaceae; Escherichia

NC_008152	RefSeq	RVG	G37	44,385	45.2	Enterobacteria phage K1-5	Viruses; dsDNA viruses, no RNA stage; Caudovirales; Podoviridae; Autographivirinae.	187764	Bacteria; Proteobacteria; Gammaproteobacteria; Enterobacteriales; Enterobacteriaceae; Escherichia
NC_020414	RefSeq	RVG	G37	43,931	47.4	Enterobacteria phage UAB_Phi78	Viruses; dsDNA viruses, no RNA stage; Caudovirales; Podoviridae; Autographivirinae; unclassified Sp6likevirus.	979726	Bacteria; Proteobacteria; Gammaproteobacteria; Enterobacteriales; Enterobacteriaceae; Salmonella; Salmonella enterica
NC_004831	RefSeq	RVG	G37	43,769	47.2	Enterobacteria phage SP6	Viruses; dsDNA viruses, no RNA stage; Caudovirales; Podoviridae; Autographivirinae.	194966	Bacteria; Proteobacteria; Gammaproteobacteria; Enterobacteriales; Enterobacteriaceae; Salmonella; Salmonella enterica
NC_027390	RefSeq	RVG	G37	45,169	39.4	Proteus phage PM 93	Viruses; dsDNA viruses, no RNA stage; Caudovirales; Podoviridae.	1560284	Bacteria; Proteobacteria; Gammaproteobacteria; Enterobacteriales; Enterobacteriaceae; Proteus
NC_028916	RefSeq	RVG	G37	44,573	39.6	Proteus phage vB_PmiP_Pm5460	Viruses; dsDNA viruses, no RNA stage; Caudovirales; Podoviridae; Autographivirinae; unclassified Sp6likevirus.	1636249	-
NC_027379	RefSeq	RVG	G37	43,642	39.3	Proteus phage PM 85	Viruses; dsDNA viruses, no RNA stage; Caudovirales; Podoviridae.	1560283	Bacteria; Proteobacteria; Gammaproteobacteria; Enterobacteriales; Enterobacteriaceae; Proteus
NC_019542	RefSeq	RVG	G37	44,400	49.7	Pectobacterium phage PP1	Viruses; dsDNA viruses, no RNA stage; Caudovirales; Podoviridae; Autographivirinae; unclassified Sp6likevirus.	1217810	Bacteria; Proteobacteria; Gammaproteobacteria; Enterobacteriales; Enterobacteriaceae; Pectobacterium; Pectobacterium carotovorum
NC_025450	RefSeq	RVG	G37	44,366	51	Lelliottia phage phD2B	Viruses; dsDNA viruses, no RNA stage; Caudovirales; Podoviridae; Autographivirinae; unclassified Sp6likevirus.	1542498	Bacteria; Proteobacteria; Gammaproteobacteria; Enterobacteriales; Enterobacteriaceae; Lelliottia
FQ482084	EBI	RVG	G37	45,522	49.7	Erwinia phage phiEa1H	Viruses; dsDNA viruses, no RNA stage; Caudovirales; Podoviridae; Autographivirinae; unclassified Autographivirinae.	925985	-
NC_019926	RefSeq	RVG	G37	45,554	49.7	Erwinia phage phiEa100	Viruses; dsDNA viruses, no RNA stage; Caudovirales; Podoviridae; Autographivirinae; unclassified Autographivirinae.	925983	Bacteria; Proteobacteria; Gammaproteobacteria; Enterobacteriales; Enterobacteriaceae; Erwinia
NC_009014	RefSeq	RVG	G37	45,445	49.8	Erwinia amylovora phage Era103	Viruses; dsDNA viruses, no RNA stage; Caudovirales; Podoviridae; Autographivirinae.	418443	Bacteria; Proteobacteria; Gammaproteobacteria; Enterobacteriales; Enterobacteriaceae; Erwinia
KF800937	EBI	RVG	G37	42,542	43.5	Vibrio phage AS51	Viruses; dsDNA viruses, no RNA stage; Caudovirales; Podoviridae.	1434127	-
NC_025822	RefSeq	RVG	G37	42,544	43.5	Vibrio phage phi-A318	Viruses; dsDNA viruses, no RNA stage; Caudovirales; Podoviridae.	1151014	Bacteria; Proteobacteria; Gammaproteobacteria; Vibrionales; Vibrionaceae; Vibrio; Vibrio harveyi group
KJ502657	EBI	RVG	G37	44,541	44.2	Vibrio phage Vc1	Viruses; dsDNA viruses, no RNA stage; Caudovirales; Podoviridae.	1480731	-
NC_027330	RefSeq	RVG	G37	45,403	45.9	Escherichia phage ECBP5	Viruses; dsDNA viruses, no RNA stage; Caudovirales; Podoviridae.	1498172	Bacteria; Proteobacteria; Gammaproteobacteria; Enterobacteriales; Enterobacteriaceae; Escherichia
TARA_ERS488558_N000440	TOV	EVG	G38	53,168	45	-	-	-	-
OBV_N00044	OBV	EVG	G38	52,651	43.6	-	-	-	-
TARA_ERS490026_N000037	TOV	EVG	G38	53,003	42.5	-	-	-	-
TARA_ERS488589_N000103	TOV	EVG	G38	51,790	38.9	-	-	-	-
TARA_ERS488354_N000095	TOV	EVG	G38	52,102	36.3	-	-	-	-
TARA_ERS488701_N000107	TOV	EVG	G39	52,675	35.1	-	-	-	-
TARA_ERS490494_N000090	TOV	EVG	G39	52,208	34.5	-	-	-	-
TARA_ERS488354_N000088	TOV	EVG	G39	54,062	39.2	-	-	-	-
OBV_N00046	OBV	EVG	G39	51,792	41.7	-	-	-	-
TARA_ERS488892_N000100	TOV	EVG	G40	48,268	32.1	-	-	-	-
TARA_ERS488892_N000093	TOV	EVG	G40	49,365	38.6	-	-	-	-
TARA_ERS490026_N000038	TOV	EVG	G40	52,774	35.6	-	-	-	-
OBV_N00071	OBV	EVG	G40	51,687	34.2	-	-	-	-
TARA_ERS489285_N000166	TOV	EVG	G41	53,779	48.3	-	-	-	-
TARA_ERS488448_N000160	TOV	EVG	G41	54,675	39.9	-	-	-	-
OBV_N00041	OBV	EVG	G41	55,201	42.8	-	-	-	-
NC_021864	RefSeq	RVG	G41	55,282	43.1	Puniceispirillum phage HMO-2011	Viruses; dsDNA viruses, no RNA stage; Caudovirales; Podoviridae.	948071	Bacteria; Proteobacteria; Alphaproteobacteria; Candidatus Puniceispirillum
TARA_ERS489603_N000049	TOV	EVG	G41	58,186	36.7	-	-	-	-
TARA_ERS478052_N000151	TOV	EVG	G41	54,218	35.1	-	-	-	-
TARA_ERS488354_N000071	TOV	EVG	G41	57,492	41.1	-	-	-	-
TARA_ERS488929_N000069	TOV	EVG	G41	57,123	41.4	-	-	-	-
TARA_ERS488589_N000077	TOV	EVG	G42	54,414	38.5	-	-	-	-
TARA_ERS490953_N000055	TOV	EVG	G42	54,959	44.7	-	-	-	-
TARA_ERS489603_N000066	TOV	EVG	G43	53,644	46.3	-	-	-	-
TARA_ERS492198_N000087	TOV	EVG	G44	56,072	35.9	-	-	-	-
NC_023863	RefSeq	RVG	G45	66,316	43.7	Vibrio phage CHOED	Viruses; dsDNA viruses, no RNA stage; Caudovirales; Podoviridae.	1458716	Bacteria; Proteobacteria; Gammaproteobacteria; Vibrionales; Vibrionaceae; Vibrio
TARA_ERS490494_N000353	TOV	EVG	G46	32,220	42.7	-	-	-	-
TARA_ERS478052_N000330	TOV	EVG	G46	35,834	41.1	-	-	-	-
TARA_ERS489285_N000395	TOV	EVG	G46	37,243	39.6	-	-	-	-
TARA_ERS490320_N000127	TOV	EVG	G46	37,243	39.6	-	-	-	-
TARA_ERS490120_N000644	TOV	EVG	G46	31,367	41.1	-	-	-	-
TARA_ERS491107_N000233	TOV	EVG	G46	37,975	45.3	-	-	-	-
OBV_N00105	OBV	EVG	G46	36,550	47.2	-	-	-	-
TARA_ERS492160_N000489	TOV	EVG	G47	32,809	45.6	-	-	-	-
TARA_ERS490142_N000494	TOV	EVG	G48	33,760	33.6	-	-	-	-
TARA_ERS488448_N000474	TOV	EVG	G49	35,250	45	-	-	-	-
TARA_ERS488757_N000101	TOV	EVG	G49	36,417	40.5	-	-	-	-
TARA_ERS478052_N000289	TOV	EVG	G50	38,591	40.5	-	-	-	-
TARA_ERS490494_N000213	TOV	EVG	G50	39,034	33.9	-	-	-	-
TARA_ERS478052_N000332	TOV	EVG	G51	35,680	42.8	-	-	-	-
TARA_ERS491107_N000165	TOV	EVG	G52	45,985	44.5	-	-	-	-
TARA_ERS490953_N000083	TOV	EVG	G52	46,217	44.1	-	-	-	-
LDNO01000003	cryoconite	EVG	G53	46,526	47.3	-	-	-	-
NC_029098	RefSeq	RVG	G54	133,531	49.5	Streptomyces phage Jay2Jay	Viruses; dsDNA viruses, no RNA stage; Caudovirales; Siphoviridae.	1556290	-
NC_002519	RefSeq	RVG	G55	39,898	46.2	Roseobacter phage SIO1	Viruses; dsDNA viruses, no RNA stage; Caudovirales; Podoviridae.	136084	Bacteria; Proteobacteria; Alphaproteobacteria; Rhodobacterales; Rhodobacteraceae

NC_018280	RefSeq	RVG	G55	38,889	46.1	Celeribacter phage P12053L	Viruses; dsDNA viruses, no RNA stage; Caudovirales.	1197951	Bacteria; Proteobacteria; Alphaproteobacteria; Rhodobacterales; Rhodobacteraceae; Celeribacter
TARA_ERS488836_N000097	TOV	EVG	G55	45,563	48	-	-	-	-
TARA_ERS488813_N000141	TOV	EVG	G55	45,567	48	-	-	-	-
TARA_ERS490285_N000309	TOV	EVG	G55	42,503	39.1	-	-	-	-
TARA_ERS489148_N000158	TOV	EVG	G55	44,272	38.8	-	-	-	-
KM224878	EBI	RVG	G56	50,250	42.5	Vibrio phage ICP2_2011_A	Viruses; dsDNA viruses, no RNA stage; Caudovirales; Podoviridae.	1529057	-
HQ641346	EBI	RVG	G56	48,626	42.5	Vibrio phage ICP2_2006_A	Viruses; dsDNA viruses, no RNA stage; Caudovirales; Podoviridae.	979534	-
NC_015158	RefSeq	RVG	G56	49,675	42.7	Vibrio phage ICP2	Viruses; dsDNA viruses, no RNA stage; Caudovirales; Podoviridae.	979533	Bacteria; Proteobacteria; Gammaproteobacteria; Vibrionales; Vibrionaceae; Vibrio
NC_024791	RefSeq	RVG	G56	50,440	42.8	Vibrio phage ICP2_2013_A_Haiti	Viruses; dsDNA viruses, no RNA stage; Caudovirales; Podoviridae.	1529058	Bacteria; Proteobacteria; Gammaproteobacteria; Vibrionales; Vibrionaceae; Vibrio
KF302037	EBI	RVG	G57	45,035	44.7	Pseudoalteromonas phage HP1	Viruses; dsDNA viruses, no RNA stage; Caudovirales; Myoviridae.	1357706	-
NC_021300	RefSeq	RVG	G57	43,882	44.7	Pseudoalteromonas phage RIO-1	Viruses; dsDNA viruses, no RNA stage; Caudovirales; Podoviridae.	1316739	Bacteria; Proteobacteria; Gammaproteobacteria; Alteromonadales; Pseudoalteromonadaceae; Pseudoalteromonas
TARA_ERS490953_N000102	TOV	EVG	G58	43,192	42	-	-	-	-
NC_007808	RefSeq	RVG	G59	49,639	44.8	Pseudomonas phage PA11	Viruses; dsDNA viruses, no RNA stage; unclassified dsDNA phages.	347327	Bacteria; Proteobacteria; Gammaproteobacteria; Pseudomonadales; Pseudomonadaceae; Pseudomonas; Pseudomonas aeruginosa group
NC_027988	RefSeq	RVG	G59	47,636	41.6	Citrobacter phage CVT22	Viruses; unclassified phages.	1622234	Bacteria; Proteobacteria; Gammaproteobacteria; Enterobacteriales; Enterobacteriaceae; Citrobacter
NC_019540	RefSeq	RVG	G59	49,390	47.7	Salinivibrio phage CW02	Viruses; dsDNA viruses, no RNA stage; Caudovirales; Podoviridae.	1161935	Bacteria; Proteobacteria; Gammaproteobacteria; Vibrionales; Vibrionaceae; Salinivibrio
NC_003907	RefSeq	RVG	G60	46,012	49.5	Vibrio phage VpV262	Viruses; dsDNA viruses, no RNA stage; Caudovirales; Podoviridae.	194802	Bacteria; Proteobacteria; Gammaproteobacteria; Vibrionales; Vibrionaceae; Vibrio; Vibrio harveyi group
AP013441	uvMED	EVG	G61	36,710	31.6	uvMED-CGR-C22-MedDCM-OCT-S33-C34 (G17)	-	-	-
OBV_N00081	OBV	EVG	G61	36,738	31.6	-	-	-	-
AP013437	uvMED	EVG	G61	38,257	33.3	uvMED-CGR-C100-MedDCM-OCT-S33-C20 (G17)	-	-	-
AP013436	uvMED	EVG	G61	38,286	32.6	uvMED-CGR-C100-MedDCM-OCT-S26-C29 (G17)	-	-	-
AP013454	uvMED	EVG	G61	36,627	31.9	uvMED-CGR-U-MedDCM-OCT-S33-C36 (G17)	-	-	-
AP013444	uvMED	EVG	G61	37,952	32.1	uvMED-CGR-C32A-MedDCM-OCT-S40-C23 (G17)	-	-	-
TARA_ERS490285_N000464	TOV	EVG	G61	34,178	32.1	-	-	-	-
TARA_ERS490320_N000162	TOV	EVG	G61	34,178	32.1	-	-	-	-
AP013453	uvMED	EVG	G61	36,835	32.4	uvMED-CGR-U-MedDCM-OCT-S28-C28 (G17)	-	-	-
AP013455	uvMED	EVG	G61	36,284	32.7	uvMED-CGR-U-MedDCM-OCT-S38-C40 (G17)	-	-	-
AP013445	uvMED	EVG	G61	38,622	30.9	uvMED-CGR-C40-MedDCM-OCT-S28-C16 (G17)	-	-	-
AP013443	uvMED	EVG	G61	39,541	32.7	uvMED-CGR-C22C-MedDCM-OCT-S23-C7 (G17)	-	-	-
AP013442	uvMED	EVG	G61	39,860	32.4	uvMED-CGR-C22-MedDCM-OCT-S36-C18 (G17)	-	-	-
AP013456	uvMED	EVG	G62	35,468	34.8	uvMED-CGR-U-MedDCM-OCT-S23-C23 (G17)	-	-	-
TARA_ERS488836_N000164	TOV	EVG	G62	36,147	34.6	-	-	-	-
AP013458	uvMED	EVG	G62	36,898	34.2	uvMED-CGR-U-MedDCM-OCT-S32-C35 (G17)	-	-	-
AP013439	uvMED	EVG	G62	35,581	35.6	uvMED-CGR-C18A-MedDCM-OCT-S41-C53 (G17)	-	-	-
AP013438	uvMED	EVG	G62	39,689	33.9	uvMED-CGR-C113A-MedDCM-OCT-S35-C17 (G17)	-	-	-
AP013459	uvMED	EVG	G63	38,825	32.2	uvMED-CGR-U-MedDCM-OCT-S39-C21 (G17)	-	-	-
TARA_ERS478052_N000281	TOV	EVG	G63	39,108	32.3	-	-	-	-
AP013440	uvMED	EVG	G63	34,607	33.6	uvMED-CGR-C19-MedDCM-OCT-S26-C54 (G17)	-	-	-
AP013460	uvMED	EVG	G63	39,004	33.6	uvMED-CGR-U-MedDCM-OCT-S40-C18 (G17)	-	-	-
AP013457	uvMED	EVG	G63	38,351	34.3	uvMED-CGR-U-MedDCM-OCT-S28-C20 (G17)	-	-	-
TARA_ERS490285_N000332	TOV	EVG	G63	40,880	32	-	-	-	-
TARA_ERS492160_N000434	TOV	EVG	G63	35,277	35.7	-	-	-	-
AP013447	uvMED	EVG	G64	32,109	38	uvMED-CGR-C66A-MedDCM-OCT-S28-C59 (G17)	-	-	-
AP013446	uvMED	EVG	G64	32,224	38.1	uvMED-CGR-C66A-MedDCM-OCT-S24-C64 (G17)	-	-	-
AP013448	uvMED	EVG	G64	32,115	38	uvMED-CGR-C66A-MedDCM-OCT-S29-C69 (G17)	-	-	-
AP013452	uvMED	EVG	G64	32,153	38.1	uvMED-CGR-C66A-MedDCM-OCT-S44-C68 (G17)	-	-	-
AP013451	uvMED	EVG	G64	32,174	37.5	uvMED-CGR-C66A-MedDCM-OCT-S40-C72 (G17)	-	-	-
AP013449	uvMED	EVG	G64	31,277	38.1	uvMED-CGR-C66A-MedDCM-OCT-S35-C82 (G17)	-	-	-
AP013450	uvMED	EVG	G64	31,599	38.4	uvMED-CGR-C66A-MedDCM-OCT-S36-C74 (G17)	-	-	-
TARA_ERS490610_N000722	TOV	EVG	G64	31,399	38.8	-	-	-	-
TARA_ERS490494_N000246	TOV	EVG	G64	37,436	47.3	-	-	-	-
TARA_ERS490610_N000468	TOV	EVG	G65	39,341	53.6	-	-	-	-
TARA_ERS489603_N000140	TOV	EVG	G65	40,171	53.3	-	-	-	-
TARA_ERS490120_N000546	TOV	EVG	G66	34,539	47.9	-	-	-	-
TARA_ERS492198_N000289	TOV	EVG	G67	33,784	48.8	-	-	-	-
TARA_ERS492160_N000451	TOV	EVG	G67	34,178	49.7	-	-	-	-
TARA_ERS488340_N000800	TOV	EVG	G67	32,247	36.4	-	-	-	-
AP013473	uvMED	EVG	G67	34,195	53	uvMED-CGR-C82-MedDCM-OCT-S28-C40 (G18)	-	-	-
AP013472	uvMED	EVG	G67	34,001	53	uvMED-CGR-C82-MedDCM-OCT-S25-C54 (G18)	-	-	-
TARA_ERS490142_N000434	TOV	EVG	G67	37,262	49.3	-	-	-	-
AP013476	uvMED	EVG	G68	32,720	51.3	uvMED-CGR-U-MedDCM-OCT-S44-C63 (G18)	-	-	-
TARA_ERS488929_N000240	TOV	EVG	G68	35,156	49.6	-	-	-	-
TARA_ERS488813_N000244	TOV	EVG	G68	35,110	36.5	-	-	-	-
TARA_ERS490204_N000363	TOV	EVG	G68	35,566	52.7	-	-	-	-
TARA_ERS490053_N000463	TOV	EVG	G69	31,644	41.6	-	-	-	-
TARA_ERS490120_N000631	TOV	EVG	G69	31,644	41.6	-	-	-	-
TARA_ERS490026_N000220	TOV	EVG	G69	31,644	41.6	-	-	-	-
TARA_ERS489943_N000556	TOV	EVG	G69	31,644	41.6	-	-	-	-
JF974298	CAMERA	RVG	G70	23,493	37.8	Methylophilales phage HIM624-A	Viruses; dsDNA viruses, no RNA stage; unclassified dsDNA phages.	889949	-
TARA_ERS492160_N000450	TOV	EVG	G71	34,187	39.5	-	-	-	-

TARA_ERS490452_N000286	TOV	EVG	G72	36,833	33.9	-	-	-	-
TARA_ERS490120_N000490	TOV	EVG	G73	37,375	47.6	-	-	-	-
TARA_ERS490053_N000314	TOV	EVG	G73	37,683	47.6	-	-	-	-
TARA_ERS490204_N000330	TOV	EVG	G73	37,247	47.4	-	-	-	-
AP013468	uvMED	EVG	G74	35,241	54.9	uvMED-CGR-C51A-MedDCM-OCT-S42-C66 (G18)	-	-	-
AP013467	uvMED	EVG	G74	35,242	54.9	uvMED-CGR-C51A-MedDCM-OCT-S39-C54 (G18)	-	-	-
AP013466	uvMED	EVG	G74	34,951	55	uvMED-CGR-C51A-MedDCM-OCT-S38-C51 (G18)	-	-	-
AP013465	uvMED	EVG	G74	34,958	54.9	uvMED-CGR-C51-MedDCM-OCT-S32-C58 (G18)	-	-	-
AP013464	uvMED	EVG	G74	35,852	54.5	uvMED-CGR-C51-MedDCM-OCT-S31-C32 (G18)	-	-	-
TARA_ERS490346_N000424	TOV	EVG	G74	36,894	46.7	-	-	-	-
TARA_ERS489285_N000390	TOV	EVG	G74	34,473	50.1	-	-	-	-
TARA_ERS488813_N000196	TOV	EVG	G75	37,525	34.2	-	-	-	-
AP013475	uvMED	EVG	G76	34,695	36.4	uvMED-CGR-C83-MedDCM-OCT-S45-C54 (G18)	-	-	-
AP013474	uvMED	EVG	G76	35,480	36.4	uvMED-CGR-C83-MedDCM-OCT-S23-C22 (G18)	-	-	-
TARA_ERS478007_N000218	TOV	EVG	G76	33,622	36.5	-	-	-	-
TARA_ERS489084_N000262	TOV	EVG	G76	35,538	37	-	-	-	-
TARA_ERS490452_N000315	TOV	EVG	G76	34,832	40.2	-	-	-	-
TARA_ERS489603_N000163	TOV	EVG	G76	37,347	38.3	-	-	-	-
TARA_ERS488673_N000435	TOV	EVG	G76	35,206	38.3	-	-	-	-
AP013477	uvMED	EVG	G77	39,948	42.8	uvMED-CGR-U-MedDCM-OCT-S25-C14 (G18)	-	-	-
TARA_ERS489084_N000199	TOV	EVG	G77	38,879	42.4	-	-	-	-
AP013479	uvMED	EVG	G78	42,029	37.9	uvMED-CGR-U-MedDCM-OCT-S35-C8 (G18)	-	-	-
AP013463	uvMED	EVG	G79	37,449	40.1	uvMED-CGR-C29A-MedDCM-OCT-S42-C45 (G18)	-	-	-
AP013461	uvMED	EVG	G79	37,755	40.1	uvMED-CGR-C29A-MedDCM-OCT-S24-C21 (G18)	-	-	-
AP013462	uvMED	EVG	G79	37,729	40.1	uvMED-CGR-C29A-MedDCM-OCT-S33-C24 (G18)	-	-	-
TARA_ERS488813_N000230	TOV	EVG	G79	35,815	49.3	-	-	-	-
AP013470	uvMED	EVG	G80	34,812	41.6	uvMED-CGR-C56A-MedDCM-OCT-S32-C59 (G18)	-	-	-
AP013469	uvMED	EVG	G80	34,856	41.7	uvMED-CGR-C56-MedDCM-OCT-S38-C52 (G18)	-	-	-
AP013471	uvMED	EVG	G80	35,133	38.2	uvMED-CGR-C63A-MedDCM-OCT-S41-C57 (G18)	-	-	-
TARA_ERS488813_N000199	TOV	EVG	G81	37,425	42.9	-	-	-	-
TARA_ERS492160_N000379	TOV	EVG	G81	38,128	44.8	-	-	-	-
TARA_ERS488340_N000798	TOV	EVG	G82	32,272	47.8	-	-	-	-
TARA_ERS490204_N000369	TOV	EVG	G82	35,309	47.6	-	-	-	-
OBV_N00113	OBV	EVG	G82	35,147	43	-	-	-	-
TARA_ERS488558_N000797	TOV	EVG	G82	35,364	50.8	-	-	-	-
TARA_ERS489285_N000353	TOV	EVG	G82	36,259	53.1	-	-	-	-
TARA_ERS490346_N000493	TOV	EVG	G82	34,233	48.7	-	-	-	-
TARA_ERS488737_N000271	TOV	EVG	G83	35,170	53.4	-	-	-	-
TARA_ERS488813_N000237	TOV	EVG	G83	35,544	53.4	-	-	-	-
TARA_ERS490494_N000207	TOV	EVG	G84	39,314	48.2	-	-	-	-
TARA_ERS488354_N000229	TOV	EVG	G85	35,216	35.7	-	-	-	-
HE983844	EBI	RVG	G86	45,469	52	Pseudomonas phage vB_PaeP_C1-14_Or	Viruses; dsDNA viruses, no RNA stage; Caudovirales; Podoviridae; unclassified Luz24likevirus.	1229675	-
NC_019813	RefSeq	RVG	G86	44,030	52	Pseudomonas phage vB_PaeP_p2-10_Or1	Viruses; dsDNA viruses, no RNA stage; Caudovirales; Podoviridae; unclassified Luz24likevirus.	1234701	Bacteria; Proteobacteria; Gammaproteobacteria; Pseudomonadales; Pseudomonadaceae; Pseudomonas; Pseudomonas aeruginosa group
JN254801	EBI	RVG	G86	44,789	52	Pseudomonas phage MR299-2	Viruses; dsDNA viruses, no RNA stage; Caudovirales; Podoviridae; unclassified Luz24likevirus.	1158721	-
NC_004466	RefSeq	RVG	G86	45,503	52.2	Pseudomonas phage PaP3	Viruses; dsDNA viruses, no RNA stage; Caudovirales; Podoviridae.	188350	Bacteria; Proteobacteria; Gammaproteobacteria; Pseudomonadales; Pseudomonadaceae; Pseudomonas; Pseudomonas aeruginosa group
NC_028933	RefSeq	RVG	G86	45,626	52	Pseudomonas phage PhiCHU	Viruses; dsDNA viruses, no RNA stage; Caudovirales; Podoviridae.	1589273	-
NC_010325	RefSeq	RVG	G86	45,625	52.2	Pseudomonas phage LUZ24	Viruses; dsDNA viruses, no RNA stage; Caudovirales; Podoviridae.	484895	Bacteria; Proteobacteria; Gammaproteobacteria; Pseudomonadales; Pseudomonadaceae; Pseudomonas; Pseudomonas aeruginosa group
NC_023583	RefSeq	RVG	G86	45,696	52.4	Pseudomonas phage TL	Viruses; dsDNA viruses, no RNA stage; Caudovirales; Podoviridae.	1406974	Bacteria; Proteobacteria; Gammaproteobacteria; Pseudomonadales; Pseudomonadaceae; Pseudomonas; Pseudomonas aeruginosa group
NC_026599	RefSeq	RVG	G86	45,808	52.4	Pseudomonas phage vB_PaeP_C2-10_Ab22	Viruses; dsDNA viruses, no RNA stage; Caudovirales; Podoviridae.	1548906	Bacteria; Proteobacteria; Gammaproteobacteria; Pseudomonadales; Pseudomonadaceae; Pseudomonas; Pseudomonas aeruginosa group
NC_028919	RefSeq	RVG	G86	45,673	52.4	Pseudomonas phage DL54	Viruses; dsDNA viruses, no RNA stage; Caudovirales; Podoviridae.	1640969	-
NC_022971	RefSeq	RVG	G86	45,344	52.3	Pseudomonas phage phiIBB-PAA2	Viruses; dsDNA viruses, no RNA stage; Caudovirales; Podoviridae; unclassified Luz24likevirus.	1429758	Bacteria; Proteobacteria; Gammaproteobacteria; Pseudomonadales; Pseudomonadaceae; Pseudomonas; Pseudomonas aeruginosa group
NC_018850	RefSeq	RVG	G86	45,517	51.5	Pseudomonas phage UFV-P2	Viruses; dsDNA viruses, no RNA stage; Caudovirales; Podoviridae.	1235661	Bacteria; Proteobacteria; Gammaproteobacteria; Pseudomonadales; Pseudomonadaceae; Pseudomonas; Pseudomonas fluorescens group
NC_017971	RefSeq	RVG	G86	46,271	53.2	Pseudomonas phage tf	Viruses; dsDNA viruses, no RNA stage; Caudovirales; Podoviridae.	1114179	Bacteria; Proteobacteria; Gammaproteobacteria; Pseudomonadales; Pseudomonadaceae; Pseudomonas; Pseudomonas putida group
KJ936628	EBI	RVG	G87	50,431	41.3	Vibrio phage VPp1	Viruses; unclassified phages.	1524880	-
NC_018835	RefSeq	RVG	G88	77,448	42	Enterobacteria phage NJ01	Viruses; dsDNA viruses, no RNA stage; Caudovirales; Podoviridae.	1237159	Bacteria; Proteobacteria; Gammaproteobacteria; Enterobacteriales; Enterobacteriaceae; Escherichia
NC_028903	RefSeq	RVG	G88	77,266	42	Escherichia phage 172-1	Viruses; dsDNA viruses, no RNA stage; Caudovirales; Podoviridae.	1598146	-
NC_010324	RefSeq	RVG	G88	77,554	42.3	Enterobacteria phage Phieco32	Viruses; dsDNA viruses, no RNA stage; Caudovirales; Podoviridae; Phieco32likevirus.	490103	Bacteria; Proteobacteria; Gammaproteobacteria; Enterobacteriales; Enterobacteriaceae; Escherichia
NC_027395	RefSeq	RVG	G88	77,327	42.1	Phage vB_EcoP_SU10	Viruses; dsDNA viruses, no RNA stage; Caudovirales; Podoviridae.	1519788	Bacteria; Proteobacteria; Gammaproteobacteria; Enterobacteriales; Enterobacteriaceae; Escherichia
NC_023593	RefSeq	RVG	G88	76,184	42.4	Escherichia phage KBNP1711	Viruses; dsDNA viruses, no RNA stage; Caudovirales; Podoviridae.	1436889	Bacteria; Proteobacteria; Gammaproteobacteria; Enterobacteriales; Enterobacteriaceae; Escherichia

NC_018859	RefSeq	RVG	G88	77,315	42.4	Escherichia phage ECBP2	Viruses; dsDNA viruses, no RNA stage; Caudovirales; Podoviridae.	1604355	Bacteria; Proteobacteria; Gammaproteobacteria; Enterobacteriales; Enterobacteriaceae; Escherichia
NC_019402	RefSeq	RVG	G89	76,631	44.2	Cronobacter phage vB_CsaP_GAP52	Viruses; dsDNA viruses, no RNA stage; Caudovirales; Podoviridae; Phieco32likevirus; unclassified Phieco32likevirus.	1141137	Bacteria; Proteobacteria; Gammaproteobacteria; Enterobacteriales; Enterobacteriaceae; Cronobacter
NC_015938	RefSeq	RVG	G89	89,916	44.1	Salmonella phage 7-11	Viruses; dsDNA viruses, no RNA stage; Caudovirales; Podoviridae.	1054968	Bacteria; Proteobacteria; Gammaproteobacteria; Enterobacteriales; Enterobacteriaceae; Salmonella; Salmonella enterica
TARA_ERS490346_N000450	TOV	EVG	G90	35,831	33.2	-	-	-	-
TARA_ERS490494_N000274	TOV	EVG	G90	35,838	33.2	-	-	-	-
OBV_N00103	OBV	EVG	G90	36,779	35.5	-	-	-	-
TARA_ERS488448_N000479	TOV	EVG	G91	35,178	48.2	-	-	-	-
TARA_ERS490120_N000433	TOV	EVG	G92	40,024	43	-	-	-	-
TARA_ERS490610_N000445	TOV	EVG	G92	40,431	44.3	-	-	-	-
TARA_ERS490346_N000423	TOV	EVG	G92	36,914	37	-	-	-	-
TARA_ERS490026_N000029	TOV	EVG	G92	56,402	39.9	-	-	-	-
TARA_ERS490053_N000307	TOV	EVG	G93	38,131	36	-	-	-	-
TARA_ERS490120_N000474	TOV	EVG	G93	38,131	36	-	-	-	-
TARA_ERS490053_N000303	TOV	EVG	G94	38,505	35	-	-	-	-
AP013369	uvMED	EVG	G95	41,524	56	uvMED-CGR-U-MedDCM-OCT-S43-C17 (G12)	-	-	-
KM058087	EBI	RVG	G96	42,205	46.1	Cronobacter phage vB_CsaP_Ss1	Viruses; dsDNA viruses, no RNA stage; Caudovirales; Podoviridae.	1498011	-
TARA_ERS490557_N000354	TOV	EVG	G97	36,978	43.4	-	-	-	-
TARA_ERS488448_N000365	TOV	EVG	G98	39,639	58.3	-	-	-	-
TARA_ERS488448_N000360	TOV	EVG	G99	39,934	52.4	-	-	-	-
TARA_ERS490494_N000201	TOV	EVG	G100	39,704	35.3	-	-	-	-
TARA_ERS488589_N000233	TOV	EVG	G101	37,424	48.3	-	-	-	-
TARA_ERS489113_N000134	TOV	EVG	G102	57,144	39.7	-	-	-	-
TARA_ERS490204_N000120	TOV	EVG	G102	59,393	39.9	-	-	-	-
TARA_ERS490610_N000232	TOV	EVG	G102	58,104	37.7	-	-	-	-
TARA_ERS488589_N000061	TOV	EVG	G102	58,289	37.6	-	-	-	-
TARA_ERS490142_N000203	TOV	EVG	G102	60,110	34.5	-	-	-	-
TARA_ERS488673_N000213	TOV	EVG	G102	58,365	35.3	-	-	-	-
TARA_ERS490452_N000123	TOV	EVG	G102	59,561	36.1	-	-	-	-
TARA_ERS488558_N000630	TOV	EVG	G102	41,711	49	-	-	-	-
TARA_ERS488701_N000073	TOV	EVG	G102	60,381	45.7	-	-	-	-
TARA_ERS478052_N000133	TOV	EVG	G102	56,461	42.4	-	-	-	-
TARA_ERS490142_N000258	TOV	EVG	G102	52,657	42.5	-	-	-	-
TARA_ERS490610_N000273	TOV	EVG	G102	53,737	42.6	-	-	-	-
TARA_ERS490346_N000192	TOV	EVG	G102	58,542	49.9	-	-	-	-
TARA_ERS490142_N000234	TOV	EVG	G102	56,385	44.5	-	-	-	-
TARA_ERS490610_N000249	TOV	EVG	G102	56,119	46.8	-	-	-	-
TARA_ERS478052_N000117	TOV	EVG	G102	59,012	46.6	-	-	-	-
TARA_ERS490610_N000241	TOV	EVG	G102	57,352	50.4	-	-	-	-
TARA_ERS490320_N000040	TOV	EVG	G102	53,899	42.2	-	-	-	-
TARA_ERS490452_N000119	TOV	EVG	G102	60,562	45.6	-	-	-	-
TARA_ERS490142_N000263	TOV	EVG	G102	52,087	47.3	-	-	-	-
TARA_ERS488929_N000062	TOV	EVG	G102	60,005	45.6	-	-	-	-
TARA_ERS492198_N000081	TOV	EVG	G102	56,885	49.4	-	-	-	-
TARA_ERS489113_N000135	TOV	EVG	G102	57,106	44.7	-	-	-	-
TARA_ERS490180_N000200	TOV	EVG	G102	56,796	47.8	-	-	-	-
TARA_ERS488589_N000062	TOV	EVG	G102	57,921	44.8	-	-	-	-
TARA_ERS490204_N000139	TOV	EVG	G103	54,751	36.4	-	-	-	-
TARA_ERS490204_N000121	TOV	EVG	G103	59,131	38.8	-	-	-	-
TARA_ERS490610_N000234	TOV	EVG	G104	58,045	32.1	-	-	-	-
TARA_ERS490452_N000132	TOV	EVG	G104	58,049	32.2	-	-	-	-
TARA_ERS489603_N000043	TOV	EVG	G104	59,997	36.9	-	-	-	-
TARA_ERS490346_N000173	TOV	EVG	G104	61,167	36.7	-	-	-	-
TARA_ERS489285_N000170	TOV	EVG	G105	53,517	40.5	-	-	-	-
TARA_ERS489148_N000095	TOV	EVG	G105	53,538	40.5	-	-	-	-
TARA_ERS490953_N000052	TOV	EVG	G105	56,740	37.5	-	-	-	-
TARA_ERS490610_N000265	TOV	EVG	G105	54,263	47.4	-	-	-	-
TARA_ERS490452_N000134	TOV	EVG	G105	56,481	43	-	-	-	-
TARA_ERS488340_N000366	TOV	EVG	G105	54,560	40.1	-	-	-	-
TARA_ERS488589_N000078	TOV	EVG	G105	54,360	37.6	-	-	-	-
TARA_ERS488340_N000386	TOV	EVG	G105	52,227	36.2	-	-	-	-
TARA_ERS490346_N000221	TOV	EVG	G106	55,065	37.6	-	-	-	-
TARA_ERS488340_N000360	TOV	EVG	G107	54,883	49.7	-	-	-	-
TARA_ERS488340_N000334	TOV	EVG	G107	57,681	40.4	-	-	-	-
TARA_ERS490494_N000075	TOV	EVG	G107	55,465	52.4	-	-	-	-
TARA_ERS489113_N000132	TOV	EVG	G107	57,387	53.2	-	-	-	-
OBV_N00034	OBV	EVG	G107	57,704	51.2	-	-	-	-
TARA_ERS492160_N000199	TOV	EVG	G107	58,036	48.9	-	-	-	-
TARA_ERS490346_N000190	TOV	EVG	G107	58,691	37.5	-	-	-	-
TARA_ERS489943_N000204	TOV	EVG	G107	58,706	38.1	-	-	-	-
TARA_ERS489943_N000194	TOV	EVG	G107	60,245	37	-	-	-	-
TARA_ERS488354_N000072	TOV	EVG	G107	57,449	39.1	-	-	-	-

TARA_ERS488892_N000058	TOV	EVG	G107	60,867	37	-	-	-	-
OBV_N00036	OBV	EVG	G107	55,971	39.6	-	-	-	-
TARA_ERS490320_N000034	TOV	EVG	G108	58,162	46.4	-	-	-	-
TARA_ERS488701_N000083	TOV	EVG	G108	58,431	48	-	-	-	-
TARA_ERS489084_N000070	TOV	EVG	G108	54,054	41.1	-	-	-	-
TARA_ERS490610_N000237	TOV	EVG	G108	57,553	46.8	-	-	-	-
TARA_ERS488448_N000131	TOV	EVG	G108	59,376	50.4	-	-	-	-
TARA_ERS490452_N000116	TOV	EVG	G108	60,997	48.3	-	-	-	-
TARA_ERS490142_N000204	TOV	EVG	G108	59,622	50.7	-	-	-	-
TARA_ERS490285_N000168	TOV	EVG	G108	59,649	50.8	-	-	-	-
TARA_ERS490494_N000068	TOV	EVG	G108	57,024	41.2	-	-	-	-
TARA_ERS489113_N000125	TOV	EVG	G108	59,131	44.6	-	-	-	-
TARA_ERS490610_N000245	TOV	EVG	G109	56,964	49.1	-	-	-	-
TARA_ERS489603_N000047	TOV	EVG	G109	59,079	49.3	-	-	-	-
TARA_ERS490494_N000048	TOV	EVG	G109	61,520	50	-	-	-	-
TARA_ERS490494_N000069	TOV	EVG	G110	56,324	47.8	-	-	-	-
TARA_ERS490610_N000228	TOV	EVG	G110	58,461	50.4	-	-	-	-
TARA_ERS488448_N000155	TOV	EVG	G111	55,615	49.9	-	-	-	-
TARA_ERS490204_N000123	TOV	EVG	G112	58,857	42.1	-	-	-	-
TARA_ERS488518_N000176	TOV	EVG	G112	64,356	38.8	-	-	-	-
TARA_ERS488518_N000203	TOV	EVG	G112	59,366	46.4	-	-	-	-
TARA_ERS488499_N000101	TOV	EVG	G112	59,666	46.3	-	-	-	-
TARA_ERS490346_N000179	TOV	EVG	G112	60,170	36.9	-	-	-	-
TARA_ERS490026_N000016	TOV	EVG	G112	66,021	37.7	-	-	-	-
TARA_ERS488499_N000139	TOV	EVG	G113	50,732	35.6	-	-	-	-
TARA_ERS478052_N000150	TOV	EVG	G113	54,607	37.5	-	-	-	-
TARA_ERS488354_N000082	TOV	EVG	G114	54,792	35.4	-	-	-	-
TARA_ERS488354_N000068	TOV	EVG	G114	57,775	37.1	-	-	-	-
TARA_ERS488701_N000071	TOV	EVG	G114	60,799	36.8	-	-	-	-
TARA_ERS489285_N000157	TOV	EVG	G114	55,607	36.5	-	-	-	-
TARA_ERS488354_N000055	TOV	EVG	G115	60,989	36.2	-	-	-	-
TARA_ERS490346_N000216	TOV	EVG	G116	55,963	34.4	-	-	-	-
TARA_ERS490142_N000228	TOV	EVG	G116	56,684	34.9	-	-	-	-
TARA_ERS490346_N000227	TOV	EVG	G116	54,139	43.3	-	-	-	-
TARA_ERS488589_N000064	TOV	EVG	G117	57,641	40	-	-	-	-
TARA_ERS488558_N000388	TOV	EVG	G117	57,677	40	-	-	-	-
TARA_ERS488813_N000070	TOV	EVG	G117	62,502	40.9	-	-	-	-
TARA_ERS488673_N000223	TOV	EVG	G117	56,419	39.5	-	-	-	-
TARA_ERS488836_N000048	TOV	EVG	G117	60,206	41.1	-	-	-	-
TARA_ERS490053_N000138	TOV	EVG	G117	55,797	41.5	-	-	-	-
TARA_ERS490120_N000249	TOV	EVG	G117	55,797	41.5	-	-	-	-
TARA_ERS490026_N000030	TOV	EVG	G117	56,282	42.8	-	-	-	-
TARA_ERS488354_N000074	TOV	EVG	G117	56,426	46.2	-	-	-	-
TARA_ERS488558_N000320	TOV	EVG	G117	66,102	56	-	-	-	-
NC_024123	RefSeq	RVG	G118	64,113	60.3	Pseudomonas phage KPP25	Viruses; dsDNA viruses, no RNA stage; Caudovirales; Podoviridae.	1462608	Bacteria; Proteobacteria; Gammaproteobacteria; Pseudomonadales; Pseudomonadaceae; Pseudomonas; Pseudomonas aeruginosa group
TARA_ERS490120_N000256	TOV	EVG	G119	54,867	38.1	-	-	-	-
TARA_ERS488892_N000081	TOV	EVG	G120	53,591	45.5	-	-	-	-
TARA_ERS488929_N000087	TOV	EVG	G120	53,591	45.6	-	-	-	-
TARA_ERS490204_N000151	TOV	EVG	G120	53,229	39.6	-	-	-	-
TARA_ERS478052_N000141	TOV	EVG	G120	55,453	35.9	-	-	-	-
TARA_ERS490494_N000074	TOV	EVG	G120	55,565	37.4	-	-	-	-
LDNN01000015	cryoconite	EVG	G121	38,334	58.4	-	-	-	-
TARA_ERS492160_N000209	TOV	EVG	G122	55,840	43.8	-	-	-	-
TARA_ERS490494_N000061	TOV	EVG	G122	57,664	39.2	-	-	-	-
TARA_ERS489148_N000065	TOV	EVG	G122	60,251	41.3	-	-	-	-
TARA_ERS490204_N000126	TOV	EVG	G123	57,877	53.6	-	-	-	-
TARA_ERS490953_N000048	TOV	EVG	G123	58,154	53.5	-	-	-	-
TARA_ERS490320_N000030	TOV	EVG	G124	59,471	46.8	-	-	-	-
TARA_ERS488354_N000062	TOV	EVG	G124	59,711	49.1	-	-	-	-
TARA_ERS478052_N000357	TOV	EVG	G125	34,902	57.4	-	-	-	-
TARA_ERS488354_N000216	TOV	EVG	G125	35,733	57.4	-	-	-	-
TARA_ERS492160_N000403	TOV	EVG	G125	36,852	57.4	-	-	-	-
TARA_ERS489113_N000314	TOV	EVG	G125	33,005	54.3	-	-	-	-
TARA_ERS490120_N000518	TOV	EVG	G125	35,489	54.5	-	-	-	-
TARA_ERS490320_N000165	TOV	EVG	G125	33,984	45.2	-	-	-	-
TARA_ERS478052_N000299	TOV	EVG	G125	37,808	50.4	-	-	-	-
TARA_ERS490494_N000028	TOV	EVG	G125	72,661	52.5	-	-	-	-
TARA_ERS490557_N000401	TOV	EVG	G125	34,773	54.9	-	-	-	-
TARA_ERS478052_N000310	TOV	EVG	G125	37,078	41.6	-	-	-	-
TARA_ERS490320_N000124	TOV	EVG	G125	37,382	42.9	-	-	-	-
TARA_ERS490320_N000119	TOV	EVG	G125	37,782	46.1	-	-	-	-
TARA_ERS488589_N000260	TOV	EVG	G125	35,687	45.1	-	-	-	-
TARA_ERS488701_N000254	TOV	EVG	G125	35,699	45.1	-	-	-	-
TARA_ERS490494_N000257	TOV	EVG	G125	36,856	44.2	-	-	-	-

TARA_ERS490320_N000137	TOV	EVG	G125	36,522	51.6	-	-	-	-
TARA_ERS488892_N000202	TOV	EVG	G125	36,596	54.4	-	-	-	-
TARA_ERS490610_N000548	TOV	EVG	G125	36,704	52.2	-	-	-	-
AP013392	uvMED	EVG	G125	34,445	36.4	uvMED-CGR-C6A-MedDCM-OCT-S43-C64 (G15)	-	-	-
AP013391	uvMED	EVG	G125	34,434	36.4	uvMED-CGR-C6A-MedDCM-OCT-S33-C54 (G15)	-	-	-
AP013390	uvMED	EVG	G125	33,823	35.9	uvMED-CGR-C6-MedDCM-OCT-S27-C41 (G15)	-	-	-
TARA_ERS478007_N000219	TOV	EVG	G125	33,606	36.5	-	-	-	-
AP013405	uvMED	EVG	G125	35,701	35.6	uvMED-CGR-U-MedDCM-OCT-S42-C63 (G15)	-	-	-
TARA_ERS490026_N000153	TOV	EVG	G125	36,338	48.1	-	-	-	-
TARA_ERS490557_N000442	TOV	EVG	G125	33,226	52.7	-	-	-	-
TARA_ERS488589_N000301	TOV	EVG	G125	33,581	37	-	-	-	-
TARA_ERS489113_N000285	TOV	EVG	G125	34,796	56.5	-	-	-	-
TARA_ERS488340_N000731	TOV	EVG	G125	34,041	57	-	-	-	-
TARA_ERS488929_N000226	TOV	EVG	G125	36,173	47.1	-	-	-	-
AP013404	uvMED	EVG	G125	32,527	46	uvMED-CGR-U-MedDCM-OCT-S41-C82 (G15)	-	-	-
TARA_ERS488589_N000312	TOV	EVG	G125	33,148	45.9	-	-	-	-
TARA_ERS492198_N000333	TOV	EVG	G125	31,720	48	-	-	-	-
TARA_ERS488448_N000523	TOV	EVG	G125	33,698	47.8	-	-	-	-
TARA_ERS488354_N000235	TOV	EVG	G125	34,836	42	-	-	-	-
TARA_ERS488340_N000703	TOV	EVG	G125	34,867	42	-	-	-	-
TARA_ERS478007_N000222	TOV	EVG	G125	33,258	43.7	-	-	-	-
OBV_N00107	OBV	EVG	G125	36,077	47.6	-	-	-	-
TARA_ERS489113_N000288	TOV	EVG	G125	34,716	37.1	-	-	-	-
TARA_ERS490142_N000476	TOV	EVG	G125	34,849	40.7	-	-	-	-
TARA_ERS488589_N000256	TOV	EVG	G125	35,988	44.2	-	-	-	-
AP013384	uvMED	EVG	G126	35,473	52.1	uvMED-CGR-C111A-MedDCM-OCT-S34-C44 (G15)	-	-	-
TARA_ERS489943_N000514	TOV	EVG	G127	33,014	46.5	-	-	-	-
TARA_ERS492198_N000303	TOV	EVG	G127	33,175	46.6	-	-	-	-
TARA_ERS488558_N000914	TOV	EVG	G127	31,642	45.8	-	-	-	-
TARA_ERS488589_N000306	TOV	EVG	G127	33,344	51.5	-	-	-	-
TARA_ERS488757_N000124	TOV	EVG	G127	33,674	50.5	-	-	-	-
TARA_ERS490120_N000592	TOV	EVG	G127	33,023	49.3	-	-	-	-
TARA_ERS489603_N000208	TOV	EVG	G127	34,589	42.5	-	-	-	-
TARA_ERS490494_N000181	TOV	EVG	G127	41,182	42.3	-	-	-	-
TARA_ERS478052_N000399	TOV	EVG	G127	33,133	46.2	-	-	-	-
TARA_ERS490346_N000548	TOV	EVG	G127	32,390	51	-	-	-	-
TARA_ERS488589_N000302	TOV	EVG	G127	33,547	38.2	-	-	-	-
TARA_ERS489603_N000238	TOV	EVG	G127	32,829	34.7	-	-	-	-
TARA_ERS490026_N000202	TOV	EVG	G127	32,829	34.7	-	-	-	-
TARA_ERS492198_N000294	TOV	EVG	G127	33,387	35	-	-	-	-
AP013400	uvMED	EVG	G128	36,021	34.4	uvMED-CGR-U-MedDCM-OCT-S30-C37 (G15)	-	-	-
TARA_ERS488836_N000142	TOV	EVG	G128	37,983	34.7	-	-	-	-
AP013406	uvMED	EVG	G128	37,654	34.7	uvMED-CGR-U-MedDCM-OCT-S46-C34 (G15)	-	-	-
AP013396	uvMED	EVG	G128	36,966	33.7	uvMED-CGR-C99-MedDCM-OCT-S44-C25 (G15)	-	-	-
AP013389	uvMED	EVG	G128	36,730	34.7	uvMED-CGR-C54A-MedDCM-OCT-S39-C35 (G15)	-	-	-
AP013388	uvMED	EVG	G128	36,799	34.6	uvMED-CGR-C54-MedDCM-OCT-S36-C37 (G15)	-	-	-
AP013387	uvMED	EVG	G128	34,473	33	uvMED-CGR-C42-MedDCM-OCT-S29-C52 (G15)	-	-	-
NC_020481	RefSeq	RVG	G128	34,892	32	Pelagibacter phage HTVC010P	Viruses; dsDNA viruses, no RNA stage; Caudovirales; Podoviridae.	1283077	Bacteria; Proteobacteria; Alphaproteobacteria; Pelagibacterales; Pelagibacteraceae; Candidatus Pelagibacter
AP013386	uvMED	EVG	G128	34,952	32.7	uvMED-CGR-C23-MedDCM-OCT-S37-C51 (G15)	-	-	-
AP013399	uvMED	EVG	G128	35,327	32.4	uvMED-CGR-U-MedDCM-OCT-S40-C42 (G15)	-	-	-
AP013385	uvMED	EVG	G128	36,025	32.4	uvMED-CGR-C16-MedDCM-OCT-S39-C44 (G15)	-	-	-
AP013398	uvMED	EVG	G128	35,951	32.5	uvMED-CGR-U-MedDCM-OCT-S30-C38 (G15)	-	-	-
AP013401	uvMED	EVG	G128	35,059	33.3	uvMED-CGR-U-MedDCM-OCT-S37-C49 (G15)	-	-	-
AP013397	uvMED	EVG	G128	34,966	33.1	uvMED-CGR-U-MedDCM-OCT-S25-C46 (G15)	-	-	-
AP013383	uvMED	EVG	G128	39,751	34	uvMED-CGR-C110A-MedDCM-OCT-S24-C13 (G15)	-	-	-
TARA_ERS488589_N000277	TOV	EVG	G128	34,845	35	-	-	-	-
TARA_ERS489113_N000295	TOV	EVG	G128	34,237	37.1	-	-	-	-
TARA_ERS490053_N000319	TOV	EVG	G129	37,388	50.5	-	-	-	-
TARA_ERS490120_N000485	TOV	EVG	G129	37,671	50.4	-	-	-	-
TARA_ERS490610_N000582	TOV	EVG	G129	35,468	48.1	-	-	-	-
TARA_ERS490494_N000272	TOV	EVG	G129	36,012	40.3	-	-	-	-
TARA_ERS491107_N000237	TOV	EVG	G129	37,466	44.4	-	-	-	-
TARA_ERS488518_N000516	TOV	EVG	G129	35,608	52.7	-	-	-	-
TARA_ERS490452_N000310	TOV	EVG	G129	35,192	53.5	-	-	-	-
TARA_ERS490320_N000136	TOV	EVG	G129	36,542	47.8	-	-	-	-
TARA_ERS490494_N000218	TOV	EVG	G129	38,757	51.8	-	-	-	-
TARA_ERS478007_N000146	TOV	EVG	G130	38,026	37.1	-	-	-	-
TARA_ERS488518_N000458	TOV	EVG	G130	38,030	37.1	-	-	-	-
TARA_ERS489943_N000432	TOV	EVG	G130	37,700	42.2	-	-	-	-
TARA_ERS490494_N000260	TOV	EVG	G130	36,782	41.6	-	-	-	-
AP013402	uvMED	EVG	G131	38,437	31.5	uvMED-CGR-U-MedDCM-OCT-S38-C27 (G15)	-	-	-
TARA_ERS489603_N000274	TOV	EVG	G131	31,023	32.7	-	-	-	-
AP013393	uvMED	EVG	G131	39,693	31	uvMED-CGR-C72-MedDCM-OCT-S43-C21 (G15)	-	-	-
TARA_ERS488558_N000775	TOV	EVG	G131	36,119	36.9	-	-	-	-

TARA_ERS489148_N000260	TOV	EVG	G131	37,041	34.3	-	-	-	-
TARA_ERS489943_N000439	TOV	EVG	G131	37,209	35.4	-	-	-	-
TARA_ERS478052_N000272	TOV	EVG	G131	39,579	32.1	-	-	-	-
TARA_ERS489148_N000275	TOV	EVG	G132	36,459	36	-	-	-	-
TARA_ERS489603_N000168	TOV	EVG	G132	36,906	39	-	-	-	-
TARA_ERS490026_N000136	TOV	EVG	G133	38,068	47.4	-	-	-	-
TARA_ERS488589_N000287	TOV	EVG	G134	34,471	51.9	-	-	-	-
TARA_ERS490204_N000345	TOV	EVG	G134	36,255	50.7	-	-	-	-
TARA_ERS489113_N000270	TOV	EVG	G134	35,648	46.2	-	-	-	-
TARA_ERS490142_N000449	TOV	EVG	G134	36,301	44.6	-	-	-	-
TARA_ERS492198_N000233	TOV	EVG	G135	36,924	47.7	-	-	-	-
AP013395	uvMED	EVG	G136	41,059	41.9	uvMED-CGR-C85-MedDCM-OCT-S43-C18 (G15)	-	-	-
AP013394	uvMED	EVG	G136	41,207	41.6	uvMED-CGR-C85-MedDCM-OCT-S24-C7 (G15)	-	-	-
AP013403	uvMED	EVG	G136	42,204	41.3	uvMED-CGR-U-MedDCM-OCT-S41-C7 (G15)	-	-	-
TARA_ERS490204_N000270	TOV	EVG	G137	40,579	41.3	-	-	-	-
TARA_ERS490494_N000182	TOV	EVG	G137	41,075	37.9	-	-	-	-
TARA_ERS489603_N000130	TOV	EVG	G137	41,391	36.1	-	-	-	-
TARA_ERS488589_N000179	TOV	EVG	G137	42,632	35.4	-	-	-	-
AP013510	uvMED	EVG	G138	42,335	40.4	uvMED-CGR-C101A-MedDCM-OCT-S30-C6 (G20)	-	-	-
TARA_ERS489059_N000114	TOV	EVG	G138	40,970	41.3	-	-	-	-
TARA_ERS490204_N000257	TOV	EVG	G138	41,490	44.9	-	-	-	-
TARA_ERS488448_N000399	TOV	EVG	G139	37,839	52.8	-	-	-	-
TARA_ERS490494_N000195	TOV	EVG	G140	40,032	43.5	-	-	-	-
TARA_ERS490204_N000403	TOV	EVG	G141	34,117	50.2	-	-	-	-
TARA_ERS488813_N000222	TOV	EVG	G142	36,192	33.1	-	-	-	-
TARA_ERS488354_N000196	TOV	EVG	G142	36,816	30.6	-	-	-	-
TARA_ERS492160_N000380	TOV	EVG	G143	38,112	32.1	-	-	-	-
TARA_ERS489113_N000221	TOV	EVG	G143	39,466	31.9	-	-	-	-
KM612265	EBI	RVG	G144	39,782	50.5	Vibrio phage J3	Viruses; dsDNA viruses, no RNA stage; Caudovirales; Podoviridae.	1558468	-
KM612261	EBI	RVG	G144	39,530	50.5	Vibrio phage H1	Viruses; dsDNA viruses, no RNA stage; Caudovirales; Podoviridae.	1558464	-
KM612263	EBI	RVG	G144	39,530	50.5	Vibrio phage H3	Viruses; dsDNA viruses, no RNA stage; Caudovirales; Podoviridae.	1558466	-
NC_027393	RefSeq	RVG	G144	39,530	50.6	Vibrio phage J2	Viruses; dsDNA viruses, no RNA stage; Caudovirales; Podoviridae.	1558467	Bacteria; Proteobacteria; Gammaproteobacteria; Vibrionales; Vibrionaceae; Vibrio
KM612262	EBI	RVG	G144	39,530	50.5	Vibrio phage H2	Viruses; dsDNA viruses, no RNA stage; Caudovirales; Podoviridae.	1558465	-
KM612260	EBI	RVG	G144	39,542	50.6	Vibrio phage CJY	Viruses; dsDNA viruses, no RNA stage; Caudovirales; Podoviridae.	1558463	-
NC_005879	RefSeq	RVG	G144	39,853	50.6	Vibrio phage VP2	Viruses; dsDNA viruses, no RNA stage; Caudovirales; Podoviridae.	260372	Bacteria; Proteobacteria; Gammaproteobacteria; Vibrionales; Vibrionaceae; Vibrio
NC_027397	RefSeq	RVG	G144	39,725	50.5	Vibrio phage QH	Viruses; dsDNA viruses, no RNA stage; Caudovirales; Podoviridae.	1558469	Bacteria; Proteobacteria; Gammaproteobacteria; Vibrionales; Vibrionaceae; Vibrio
NC_005891	RefSeq	RVG	G144	39,786	50.5	Vibrio phage VP5	Viruses; dsDNA viruses, no RNA stage; Caudovirales; Podoviridae.	260827	Bacteria; Proteobacteria; Gammaproteobacteria; Vibrionales; Vibrionaceae; Vibrio
NC_027118	RefSeq	RVG	G144	39,422	50.8	Vibrio phage phiVC8	Viruses; dsDNA viruses, no RNA stage; Caudovirales; Podoviridae.	1076759	Bacteria; Proteobacteria; Gammaproteobacteria; Vibrionales; Vibrionaceae; Vibrio
JQ691610	EBI	RVG	G145	38,689	50.2	Salmonella phage SPN9TCW	Viruses; dsDNA viruses, no RNA stage; Caudovirales; Podoviridae.	1162289	-
NC_016761	RefSeq	RVG	G145	38,684	50.2	Salmonella phage SPN1S	Viruses; dsDNA viruses, no RNA stage; Caudovirales; Podoviridae.	1125653	Bacteria; Proteobacteria; Gammaproteobacteria; Enterobacteriales; Enterobacteriaceae; Salmonella; Salmonella enterica
KC911857	EBI	RVG	G145	38,689	50.2	Salmonella phage SPC32N	Viruses; dsDNA viruses, no RNA stage; Caudovirales; Podoviridae.	1327942	-
KC911856	EBI	RVG	G145	38,689	50.2	Salmonella phage SPC32H	Viruses; dsDNA viruses, no RNA stage; Caudovirales; Podoviridae.	1327941	-
NC_004775	RefSeq	RVG	G145	39,671	50.8	Salmonella phage epsilon15	Viruses; dsDNA viruses, no RNA stage; Caudovirales; Podoviridae.	215158	Bacteria; Proteobacteria; Gammaproteobacteria; Enterobacteriales; Enterobacteriaceae; Salmonella
KP869108	EBI	RVG	G145	39,234	48.9	Escherichia coli O157 typing phage 10	Viruses; dsDNA viruses, no RNA stage; Caudovirales; Podoviridae.	1508672	-
NC_007804	RefSeq	RVG	G145	39,104	49	Escherichia phage phiV10	Viruses; dsDNA viruses, no RNA stage; Caudovirales; Podoviridae.	343516	Bacteria; Proteobacteria; Gammaproteobacteria; Enterobacteriales; Enterobacteriaceae; Escherichia
NC_019445	RefSeq	RVG	G145	44,784	47.1	Escherichia phage TL-2011b	Viruses; dsDNA viruses, no RNA stage; Caudovirales; Podoviridae; unclassified Epsilon15likevirus.	1124654	Bacteria; Proteobacteria; Gammaproteobacteria; Enterobacteriales; Enterobacteriaceae; Escherichia
NC_019923	RefSeq	RVG	G146	42,689	58.4	Pseudomonas phage AF	Viruses; dsDNA viruses, no RNA stage; Caudovirales; Podoviridae.	1235689	Bacteria; Proteobacteria; Gammaproteobacteria; Pseudomonadales; Pseudomonadaceae; Pseudomonas; Pseudomonas putida group
TARA_ERS489285_N000400	TOV	EVG	G147	34,006	33.6	-	-	-	-
TARA_ERS492198_N000165	TOV	EVG	G148	42,781	45.1	-	-	-	-
TARA_ERS490204_N000235	TOV	EVG	G148	43,366	44.6	-	-	-	-
TARA_ERS490053_N000217	TOV	EVG	G148	45,052	51.8	-	-	-	-
TARA_ERS490026_N000073	TOV	EVG	G148	45,052	51.8	-	-	-	-
TARA_ERS488354_N000121	TOV	EVG	G149	46,629	52.5	-	-	-	-
TARA_ERS492160_N000353	TOV	EVG	G150	40,283	54.4	-	-	-	-
TARA_ERS490204_N000240	TOV	EVG	G150	42,780	50.6	-	-	-	-
AP013535	uvMED	EVG	G151	40,289	35.8	uvMED-CGR-U-MedDCM-OCT-S30-C10 (G5)	-	-	-
TARA_ERS492198_N000164	TOV	EVG	G152	42,796	59.2	-	-	-	-
TARA_ERS488340_N000517	TOV	EVG	G153	42,996	61.7	-	-	-	-
TARA_ERS490204_N000157	TOV	EVG	G154	52,290	42.9	-	-	-	-
TARA_ERS490494_N000255	TOV	EVG	G155	36,996	42.1	-	-	-	-
TARA_ERS488892_N000280	TOV	EVG	G156	32,497	44.7	-	-	-	-
TARA_ERS488929_N000304	TOV	EVG	G156	32,504	46.2	-	-	-	-
TARA_ERS490204_N000448	TOV	EVG	G156	32,906	41.8	-	-	-	-
TARA_ERS490285_N000512	TOV	EVG	G156	32,607	35.3	-	-	-	-
TARA_ERS490320_N000179	TOV	EVG	G156	33,062	34.4	-	-	-	-
TARA_ERS478052_N000420	TOV	EVG	G156	32,300	33.3	-	-	-	-
TARA_ERS488589_N000328	TOV	EVG	G156	32,542	34.6	-	-	-	-
TARA_ERS489285_N000424	TOV	EVG	G156	32,607	37.7	-	-	-	-
TARA_ERS488929_N000277	TOV	EVG	G156	33,582	34.3	-	-	-	-

OBV_N00126	OBV	EVG	G156	33,814	44.4	-	-	-	-
TARA_ERS490494_N000381	TOV	EVG	G156	31,122	41.2	-	-	-	-
TARA_ERS488354_N000276	TOV	EVG	G156	32,406	41.3	-	-	-	-
TARA_ERS492160_N000458	TOV	EVG	G156	34,012	42.3	-	-	-	-
TARA_ERS489603_N000254	TOV	EVG	G156	32,264	40	-	-	-	-
AP013435	uvMED	EVG	G156	32,562	35.7	uvMED-CGR-U-MedDCM-OCT-S37-C71 (G16)	-	-	-
TARA_ERS489113_N000333	TOV	EVG	G156	32,061	33.2	-	-	-	-
AP013431	uvMED	EVG	G156	33,433	35.3	uvMED-CGR-U-MedDCM-OCT-S25-C59 (G16)	-	-	-
TARA_ERS490346_N000561	TOV	EVG	G156	31,984	35.8	-	-	-	-
TARA_ERS492198_N000299	TOV	EVG	G156	33,252	35.6	-	-	-	-
TARA_ERS490494_N000294	TOV	EVG	G156	34,955	36.5	-	-	-	-
TARA_ERS490610_N000653	TOV	EVG	G157	33,069	36.9	-	-	-	-
TARA_ERS490346_N000360	TOV	EVG	G157	40,443	34.5	-	-	-	-
AP013430	uvMED	EVG	G157	33,980	32.4	uvMED-CGR-U-MedDCM-OCT-S25-C55 (G16)	-	-	-
AP013433	uvMED	EVG	G157	33,438	33.9	uvMED-CGR-U-MedDCM-OCT-S27-C44 (G16)	-	-	-
TARA_ERS490120_N000572	TOV	EVG	G157	33,825	36	-	-	-	-
AP013425	uvMED	EVG	G157	32,324	35.3	uvMED-CGR-C55A-MedDCM-OCT-S46-C84 (G16)	-	-	-
AP013424	uvMED	EVG	G157	33,380	35.2	uvMED-CGR-C55-MedDCM-OCT-S33-C61 (G16)	-	-	-
AP013410	uvMED	EVG	G157	33,517	34.4	uvMED-CGR-C107A-MedDCM-OCT-S27-C42 (G16)	-	-	-
AP013423	uvMED	EVG	G157	33,719	32.7	uvMED-CGR-C53A-MedDCM-OCT-S34-C54 (G16)	-	-	-
AP013422	uvMED	EVG	G157	33,654	32.7	uvMED-CGR-C53A-MedDCM-OCT-S26-C61 (G16)	-	-	-
TARA_ERS488892_N000221	TOV	EVG	G157	35,219	33.5	-	-	-	-
AP013428	uvMED	EVG	G158	37,260	32.4	uvMED-CGR-C57A-MedDCM-OCT-S39-C32 (G16)	-	-	-
AP013427	uvMED	EVG	G158	37,523	32.4	uvMED-CGR-C57A-MedDCM-OCT-S25-C28 (G16)	-	-	-
AP013426	uvMED	EVG	G158	37,925	32.3	uvMED-CGR-C57-MedDCM-OCT-S27-C24 (G16)	-	-	-
AP013429	uvMED	EVG	G158	36,215	32.2	uvMED-CGR-C58A-MedDCM-OCT-S43-C47 (G16)	-	-	-
TARA_ERS490285_N000430	TOV	EVG	G158	35,723	31.6	-	-	-	-
AP013409	uvMED	EVG	G158	33,014	33.9	uvMED-CGR-C105A-MedDCM-OCT-S28-C49 (G16)	-	-	-
AP013408	uvMED	EVG	G158	32,954	33.9	uvMED-CGR-C105A-MedDCM-OCT-S27-C54 (G16)	-	-	-
TARA_ERS490053_N000416	TOV	EVG	G158	33,511	33.4	-	-	-	-
OBV_N00098	OBV	EVG	G158	35,134	32.6	-	-	-	-
TARA_ERS490610_N000541	TOV	EVG	G158	37,114	31.8	-	-	-	-
AP013411	uvMED	EVG	G159	35,248	34.3	uvMED-CGR-C108A-MedDCM-OCT-S29-C43 (G16)	-	-	-
TARA_ERS478007_N000189	TOV	EVG	G159	35,235	34.5	-	-	-	-
AP013419	uvMED	EVG	G160	39,021	37.2	uvMED-CGR-C48B-MedDCM-OCT-S41-C26 (G16)	-	-	-
AP013417	uvMED	EVG	G160	39,029	37.2	uvMED-CGR-C48B-MedDCM-OCT-S26-C25 (G16)	-	-	-
AP013416	uvMED	EVG	G160	39,033	37.2	uvMED-CGR-C48B-MedDCM-OCT-S24-C17 (G16)	-	-	-
AP013420	uvMED	EVG	G160	39,355	37	uvMED-CGR-C48B-MedDCM-OCT-S42-C26 (G16)	-	-	-
AP013418	uvMED	EVG	G160	39,355	37	uvMED-CGR-C48B-MedDCM-OCT-S30-C14 (G16)	-	-	-
AP013414	uvMED	EVG	G160	38,744	37.1	uvMED-CGR-C48A-MedDCM-OCT-S29-C21 (G16)	-	-	-
AP013413	uvMED	EVG	G160	38,738	37.1	uvMED-CGR-C48A-MedDCM-OCT-S28-C15 (G16)	-	-	-
AP013415	uvMED	EVG	G160	38,732	37.1	uvMED-CGR-C48A-MedDCM-OCT-S37-C22 (G16)	-	-	-
AP013421	uvMED	EVG	G160	33,800	46.3	uvMED-CGR-C49A-MedDCM-OCT-S31-C44 (G16)	-	-	-
TARA_ERS488892_N000226	TOV	EVG	G160	35,018	41.5	-	-	-	-
TARA_ERS492198_N000272	TOV	EVG	G160	34,860	40	-	-	-	-
TARA_ERS492160_N000435	TOV	EVG	G160	35,191	43.1	-	-	-	-
TARA_ERS490494_N000249	TOV	EVG	G160	37,291	32.2	-	-	-	-
TARA_ERS488340_N000631	TOV	EVG	G161	38,239	47.8	-	-	-	-
TARA_ERS488354_N000183	TOV	EVG	G161	38,255	47.8	-	-	-	-
TARA_ERS488757_N000130	TOV	EVG	G162	33,122	34.4	-	-	-	-
TARA_ERS488737_N000294	TOV	EVG	G162	33,122	34.4	-	-	-	-
AP013434	uvMED	EVG	G162	33,848	34	uvMED-CGR-U-MedDCM-OCT-S32-C65 (G16)	-	-	-
AP013412	uvMED	EVG	G162	34,630	33.2	uvMED-CGR-C10A-MedDCM-OCT-S46-C61 (G16)	-	-	-
AP013407	uvMED	EVG	G162	33,778	32.9	uvMED-CGR-C10-MedDCM-OCT-S30-C55 (G16)	-	-	-
TARA_ERS488558_N000882	TOV	EVG	G162	32,610	35.3	-	-	-	-
TARA_ERS488589_N000322	TOV	EVG	G162	32,614	35.3	-	-	-	-
TARA_ERS492198_N000295	TOV	EVG	G162	33,355	38	-	-	-	-
TARA_ERS489285_N000442	TOV	EVG	G162	31,511	36.3	-	-	-	-
TARA_ERS488589_N000338	TOV	EVG	G162	32,056	35.9	-	-	-	-
AP013432	uvMED	EVG	G162	32,358	37.1	uvMED-CGR-U-MedDCM-OCT-S25-C65 (G16)	-	-	-
TARA_ERS488448_N000581	TOV	EVG	G162	31,861	36.8	-	-	-	-
TARA_ERS492198_N000319	TOV	EVG	G162	32,378	36.4	-	-	-	-
TARA_ERS488892_N000250	TOV	EVG	G162	33,938	54.8	-	-	-	-
TARA_ERS489084_N000276	TOV	EVG	G163	35,032	53	-	-	-	-
TARA_ERS488836_N000181	TOV	EVG	G163	35,032	53	-	-	-	-
TARA_ERS490557_N000378	TOV	EVG	G163	35,524	51.4	-	-	-	-
TARA_ERS490610_N000578	TOV	EVG	G163	35,524	51.4	-	-	-	-
TARA_ERS488929_N000245	TOV	EVG	G163	34,970	54.8	-	-	-	-
TARA_ERS490026_N000152	TOV	EVG	G163	36,355	53.3	-	-	-	-
TARA_ERS478052_N000367	TOV	EVG	G164	34,375	48.8	-	-	-	-
TARA_ERS492198_N000359	TOV	EVG	G165	30,745	53	-	-	-	-
TARA_ERS490026_N000183	TOV	EVG	G165	33,775	36	-	-	-	-
TARA_ERS490026_N000154	TOV	EVG	G165	36,298	56.8	-	-	-	-
OBV_N00152	OBV	EVG	G166	31,315	41.3	-	-	-	-
OBV_N00127	OBV	EVG	G166	33,791	43.1	-	-	-	-

TARA_ERS488448_N000783	TOV	EVG	G167	27,461	35.1	-	-	-	-
TARA_ERS478007_N000365	TOV	EVG	G167	27,461	35.2	-	-	-	-
TARA_ERS478052_N000550	TOV	EVG	G167	27,563	35.1	-	-	-	-
TARA_ERS488589_N000430	TOV	EVG	G167	27,662	34.8	-	-	-	-
TARA_ERS490320_N000206	TOV	EVG	G167	31,715	41.2	-	-	-	-
TARA_ERS492198_N000323	TOV	EVG	G167	32,164	36.1	-	-	-	-
TARA_ERS488673_N000233	TOV	EVG	G168	53,627	30.3	-	-	-	-
TARA_ERS488558_N000433	TOV	EVG	G168	53,706	30.3	-	-	-	-
TARA_ERS489113_N000150	TOV	EVG	G168	53,696	30.2	-	-	-	-
TARA_ERS488929_N000084	TOV	EVG	G168	54,702	31.8	-	-	-	-
NC_029047	RefSeq	RVG	G169	32,894	51	Verrucomicrobia phage P8625	Viruses; dsDNA viruses, no RNA stage; Caudovirales; Siphoviridae.	1636271	-
TARA_ERS488673_N000378	TOV	EVG	G170	38,942	52.9	-	-	-	-
TARA_ERS478052_N000275	TOV	EVG	G170	39,283	52.4	-	-	-	-
TARA_ERS490204_N000267	TOV	EVG	G170	40,641	54.9	-	-	-	-
TARA_ERS488673_N000402	TOV	EVG	G171	36,945	47.8	-	-	-	-
TARA_ERS488673_N000339	TOV	EVG	G172	41,212	43.7	-	-	-	-
TARA_ERS490610_N000440	TOV	EVG	G173	40,625	34.1	-	-	-	-
TARA_ERS492160_N000308	TOV	EVG	G174	43,994	33.3	-	-	-	-
TARA_ERS492198_N000156	TOV	EVG	G174	44,156	33.4	-	-	-	-
TARA_ERS490346_N000320	TOV	EVG	G174	44,379	31.8	-	-	-	-
TARA_ERS489113_N000196	TOV	EVG	G174	44,161	33.5	-	-	-	-
TARA_ERS490452_N000242	TOV	EVG	G174	41,276	30	-	-	-	-
TARA_ERS489943_N000372	TOV	EVG	G174	41,375	29.9	-	-	-	-
TARA_ERS478052_N000256	TOV	EVG	G174	40,764	30.2	-	-	-	-
TARA_ERS490053_N000178	TOV	EVG	G174	50,107	34.5	-	-	-	-
TARA_ERS490120_N000299	TOV	EVG	G174	50,108	34.5	-	-	-	-
TARA_ERS490285_N000257	TOV	EVG	G174	47,213	32.3	-	-	-	-
TARA_ERS492198_N000122	TOV	EVG	G175	48,695	32.8	-	-	-	-
TARA_ERS490346_N000266	TOV	EVG	G175	49,558	31.8	-	-	-	-
TARA_ERS490053_N000157	TOV	EVG	G176	53,122	34.6	-	-	-	-
TARA_ERS490120_N000271	TOV	EVG	G176	53,140	34.6	-	-	-	-
TARA_ERS488589_N000128	TOV	EVG	G177	48,109	31.9	-	-	-	-
TARA_ERS490285_N000215	TOV	EVG	G177	51,406	33.3	-	-	-	-
TARA_ERS490494_N000093	TOV	EVG	G177	51,832	33.3	-	-	-	-
OBV_N00050	OBV	EVG	G177	50,862	32.3	-	-	-	-
TARA_ERS489148_N000101	TOV	EVG	G177	52,395	33.1	-	-	-	-
TARA_ERS490053_N000145	TOV	EVG	G177	54,947	34.7	-	-	-	-
TARA_ERS488518_N000266	TOV	EVG	G177	51,034	31.9	-	-	-	-
TARA_ERS478052_N000162	TOV	EVG	G177	52,094	37.2	-	-	-	-
TARA_ERS489148_N000093	TOV	EVG	G177	53,783	37.6	-	-	-	-
TARA_ERS490320_N000100	TOV	EVG	G178	40,754	32.6	-	-	-	-
TARA_ERS490026_N000096	TOV	EVG	G179	42,059	33.5	-	-	-	-
TARA_ERS490320_N000087	TOV	EVG	G179	42,872	33.7	-	-	-	-
TARA_ERS488757_N000071	TOV	EVG	G179	41,276	34.9	-	-	-	-
TARA_ERS490610_N000363	TOV	EVG	G179	45,222	38.7	-	-	-	-
TARA_ERS488701_N000109	TOV	EVG	G180	52,552	35.7	-	-	-	-
AP013382	uvMED	EVG	G181	41,230	32	uvMED-CGR-U-MedDCM-OCT-S45-C13 (G14)	-	-	-
TARA_ERS490494_N000146	TOV	EVG	G182	44,767	33.8	-	-	-	-
TARA_ERS489943_N000285	TOV	EVG	G182	48,199	34.8	-	-	-	-
TARA_ERS488589_N000148	TOV	EVG	G183	45,577	34.6	-	-	-	-
TARA_ERS488929_N000157	TOV	EVG	G184	42,136	31.3	-	-	-	-
TARA_ERS488892_N000143	TOV	EVG	G184	42,797	31.3	-	-	-	-
TARA_ERS488589_N000147	TOV	EVG	G184	45,697	33.3	-	-	-	-
TARA_ERS478052_N000209	TOV	EVG	G184	45,782	34.4	-	-	-	-
TARA_ERS489603_N000121	TOV	EVG	G184	42,319	34.3	-	-	-	-
TARA_ERS490346_N000314	TOV	EVG	G184	44,748	34.3	-	-	-	-
TARA_ERS490494_N000112	TOV	EVG	G184	48,729	37	-	-	-	-
TARA_ERS490346_N000234	TOV	EVG	G185	53,189	31.5	-	-	-	-
TARA_ERS489943_N000229	TOV	EVG	G185	54,453	30.6	-	-	-	-
TARA_ERS490494_N000064	TOV	EVG	G185	57,474	31.9	-	-	-	-
TARA_ERS490610_N000268	TOV	EVG	G185	54,133	32.6	-	-	-	-
TARA_ERS489603_N000079	TOV	EVG	G186	50,185	34.1	-	-	-	-
TARA_ERS489943_N000247	TOV	EVG	G186	52,479	33	-	-	-	-
TARA_ERS492198_N000129	TOV	EVG	G187	47,422	30.8	-	-	-	-
TARA_ERS488673_N000271	TOV	EVG	G187	48,207	30.4	-	-	-	-
TARA_ERS490320_N000060	TOV	EVG	G187	49,233	31.2	-	-	-	-
TARA_ERS489603_N000061	TOV	EVG	G188	54,229	30.6	-	-	-	-
TARA_ERS489603_N000054	TOV	EVG	G188	56,012	31.9	-	-	-	-
TARA_ERS489943_N000282	TOV	EVG	G188	48,568	30.3	-	-	-	-
TARA_ERS489084_N000057	TOV	EVG	G188	57,820	31.7	-	-	-	-
TARA_ERS492198_N000103	TOV	EVG	G188	52,276	33.9	-	-	-	-
TARA_ERS489943_N000212	TOV	EVG	G189	57,625	35	-	-	-	-
TARA_ERS490026_N000027	TOV	EVG	G189	57,625	35	-	-	-	-
TARA_ERS490494_N000050	TOV	EVG	G189	61,057	36.2	-	-	-	-
TARA_ERS488354_N000106	TOV	EVG	G190	49,485	31.2	-	-	-	-

TARA_ERS492198_N000032	TOV	EVG	G190	77,154	32.4	-	-	-	-
TARA_ERS492198_N000107	TOV	EVG	G190	51,267	33.5	-	-	-	-
TARA_ERS489148_N000084	TOV	EVG	G190	54,940	31.5	-	-	-	-
TARA_ERS488892_N000091	TOV	EVG	G191	50,348	32.5	-	-	-	-
TARA_ERS490320_N000097	TOV	EVG	G192	40,988	34.1	-	-	-	-
TARA_ERS492198_N000146	TOV	EVG	G193	45,165	34.2	-	-	-	-
TARA_ERS489603_N000085	TOV	EVG	G194	49,227	34.9	-	-	-	-
TARA_ERS490346_N000520	TOV	EVG	G195	33,313	34.2	-	-	-	-
TARA_ERS490494_N000282	TOV	EVG	G195	35,405	32.8	-	-	-	-
TARA_ERS490180_N000356	TOV	EVG	G195	38,339	30.2	-	-	-	-
TARA_ERS490053_N000350	TOV	EVG	G196	35,981	29.6	-	-	-	-
AP013380	uvMED	EVG	G197	34,478	33.8	uvMED-CGR-U-MedDCM-OCT-S31-C41 (G14)	-	-	-
TARA_ERS490053_N000397	TOV	EVG	G197	34,242	33.7	-	-	-	-
AP013379	uvMED	EVG	G197	33,989	33.5	uvMED-CGR-U-MedDCM-OCT-S28-C43 (G14)	-	-	-
TARA_ERS488892_N000236	TOV	EVG	G198	34,606	32.5	-	-	-	-
TARA_ERS489084_N000286	TOV	EVG	G198	34,606	32.5	-	-	-	-
TARA_ERS478052_N000334	TOV	EVG	G199	35,622	35.3	-	-	-	-
TARA_ERS490026_N000147	TOV	EVG	G199	36,867	36.2	-	-	-	-
TARA_ERS490053_N000361	TOV	EVG	G200	35,562	31.6	-	-	-	-
TARA_ERS488673_N000400	TOV	EVG	G200	37,215	36.1	-	-	-	-
TARA_ERS478052_N000506	TOV	EVG	G201	29,010	30.4	-	-	-	-
TARA_ERS489148_N000314	TOV	EVG	G201	34,445	30.1	-	-	-	-
TARA_ERS489084_N000412	TOV	EVG	G201	29,928	33.3	-	-	-	-
TARA_ERS478007_N000217	TOV	EVG	G201	33,771	31	-	-	-	-
TARA_ERS490053_N000438	TOV	EVG	G202	32,400	32.6	-	-	-	-
TARA_ERS490557_N000492	TOV	EVG	G203	31,614	29.5	-	-	-	-
TARA_ERS490610_N000712	TOV	EVG	G203	31,614	29.5	-	-	-	-
TARA_ERS490204_N000468	TOV	EVG	G203	32,105	29.3	-	-	-	-
TARA_ERS488813_N000346	TOV	EVG	G203	29,978	31.8	-	-	-	-
TARA_ERS490346_N000614	TOV	EVG	G203	30,555	33.6	-	-	-	-
AP013478	uvMED	EVG	G204	33,517	31.8	uvMED-CGR-U-MedDCM-OCT-S26-C62 (G18)	-	-	-
TARA_ERS490452_N000360	TOV	EVG	G204	32,036	33	-	-	-	-
TARA_ERS489603_N000265	TOV	EVG	G204	31,916	32.3	-	-	-	-
TARA_ERS490053_N000592	TOV	EVG	G205	27,411	34.7	-	-	-	-
TARA_ERS490320_N000261	TOV	EVG	G205	27,814	32.9	-	-	-	-
TARA_ERS478007_N000350	TOV	EVG	G206	27,886	36.4	-	-	-	-
TARA_ERS488518_N000759	TOV	EVG	G206	27,888	36.5	-	-	-	-
TARA_ERS490452_N000471	TOV	EVG	G206	26,894	35.8	-	-	-	-
TARA_ERS491107_N000372	TOV	EVG	G206	28,021	34.5	-	-	-	-
TARA_ERS488589_N000385	TOV	EVG	G207	29,412	32.3	-	-	-	-
TARA_ERS488589_N000327	TOV	EVG	G207	32,561	33.8	-	-	-	-
TARA_ERS488589_N000355	TOV	EVG	G207	31,065	33.6	-	-	-	-
TARA_ERS490346_N000599	TOV	EVG	G208	31,059	32.9	-	-	-	-
TARA_ERS490053_N000420	TOV	EVG	G209	33,270	36.8	-	-	-	-
TARA_ERS488589_N000264	TOV	EVG	G210	35,366	38.4	-	-	-	-
TARA_ERS489603_N000183	TOV	EVG	G210	35,788	38.7	-	-	-	-
AP013482	uvMED	EVG	G210	36,059	38.9	uvMED-CGR-U-MedDCM-OCT-S45-C47 (G18)	-	-	-
TARA_ERS488354_N000240	TOV	EVG	G210	34,610	39.3	-	-	-	-
AP013481	uvMED	EVG	G210	36,315	37.4	uvMED-CGR-U-MedDCM-OCT-S44-C29 (G18)	-	-	-
AP013480	uvMED	EVG	G210	34,967	37.2	uvMED-CGR-U-MedDCM-OCT-S37-C50 (G18)	-	-	-
TARA_ERS478052_N000414	TOV	EVG	G210	32,431	37.8	-	-	-	-
TARA_ERS488354_N000260	TOV	EVG	G210	33,283	37.9	-	-	-	-
TARA_ERS490053_N000405	TOV	EVG	G211	34,002	34.9	-	-	-	-
TARA_ERS490053_N000327	TOV	EVG	G212	36,956	35.7	-	-	-	-
TARA_ERS490120_N000498	TOV	EVG	G212	36,956	35.7	-	-	-	-
TARA_ERS490180_N000375	TOV	EVG	G212	37,476	36.3	-	-	-	-
TARA_ERS490320_N000122	TOV	EVG	G212	37,552	36.2	-	-	-	-
TARA_ERS490610_N000661	TOV	EVG	G213	32,705	34.6	-	-	-	-
TARA_ERS478052_N000317	TOV	EVG	G213	36,573	35.1	-	-	-	-
TARA_ERS489084_N000217	TOV	EVG	G212	38,005	35	-	-	-	-
TARA_ERS489084_N000190	TOV	EVG	G214	39,178	34.8	-	-	-	-
TARA_ERS489084_N000221	TOV	EVG	G215	37,618	32.9	-	-	-	-
TARA_ERS490610_N000671	TOV	EVG	G216	32,473	32.3	-	-	-	-
TARA_ERS490610_N000478	TOV	EVG	G217	38,919	32.5	-	-	-	-
OBV_N00082	OBV	EVG	G218	40,689	37.5	-	-	-	-
TARA_ERS492160_N000372	TOV	EVG	G219	38,485	37.4	-	-	-	-
TARA_ERS492198_N000203	TOV	EVG	G219	38,986	37.4	-	-	-	-
TARA_ERS489603_N000157	TOV	EVG	G219	37,910	36.9	-	-	-	-
AP013378	uvMED	EVG	G219	34,514	37.5	uvMED-CGR-U-MedDCM-OCT-S23-C32 (G14)	-	-	-
OBV_N00092	OBV	EVG	G219	39,038	34.6	-	-	-	-
AP013381	uvMED	EVG	G220	33,690	35.2	uvMED-CGR-U-MedDCM-OCT-S36-C63 (G14)	-	-	-
TARA_ERS488518_N000513	TOV	EVG	G221	35,766	33.2	-	-	-	-
TARA_ERS478007_N000104	TOV	EVG	G222	41,191	38.3	-	-	-	-
TARA_ERS489084_N000155	TOV	EVG	G222	41,645	35.1	-	-	-	-
AP013499	uvMED	EVG	G223	31,698	32.1	uvMED-CGR-C68A-MedDCM-OCT-S39-C84 (G19)	-	-	-

AP013496	uvMED	EVG	G223	31,698	32.1	uvMED-CGR-C68A-MedDCM-OCT-S28-C61 (G19)	-	-	-
AP013498	uvMED	EVG	G223	31,700	32.2	uvMED-CGR-C68A-MedDCM-OCT-S38-C76 (G19)	-	-	-
AP013497	uvMED	EVG	G223	31,699	32.2	uvMED-CGR-C68A-MedDCM-OCT-S35-C79 (G19)	-	-	-
TARA_ERS490494_N000392	TOV	EVG	G223	30,700	32.7	-	-	-	-
TARA_ERS488354_N000356	TOV	EVG	G223	28,092	37.4	-	-	-	-
AP013484	uvMED	EVG	G223	30,688	34.1	uvMED-CGR-C20A-MedDCM-OCT-S36-C79 (G19)	-	-	-
AP013483	uvMED	EVG	G223	30,688	34.1	uvMED-CGR-C20A-MedDCM-OCT-S30-C71 (G19)	-	-	-
AP013501	uvMED	EVG	G223	32,364	32.2	uvMED-CGR-U-MedDCM-OCT-S24-C60 (G19)	-	-	-
TARA_ERS491107_N000321	TOV	EVG	G223	30,909	29.5	-	-	-	-
AP013500	uvMED	EVG	G223	32,406	33.7	uvMED-CGR-C94-MedDCM-OCT-S26-C69 (G19)	-	-	-
TARA_ERS488757_N000129	TOV	EVG	G223	33,163	33.1	-	-	-	-
AP013494	uvMED	EVG	G223	31,247	32.2	uvMED-CGR-C37-MedDCM-OCT-S44-C73 (G19)	-	-	-
AP013491	uvMED	EVG	G223	31,229	32.2	uvMED-CGR-C37-MedDCM-OCT-S26-C78 (G19)	-	-	-
AP013493	uvMED	EVG	G223	31,051	32.2	uvMED-CGR-C37-MedDCM-OCT-S32-C82 (G19)	-	-	-
AP013492	uvMED	EVG	G223	31,008	32.1	uvMED-CGR-C37-MedDCM-OCT-S29-C71 (G19)	-	-	-
AP013495	uvMED	EVG	G223	32,122	31.4	uvMED-CGR-C39-MedDCM-OCT-S27-C61 (G19)	-	-	-
AP013489	uvMED	EVG	G223	30,420	30.6	uvMED-CGR-C30A-MedDCM-OCT-S45-C81 (G19)	-	-	-
AP013487	uvMED	EVG	G223	30,420	30.5	uvMED-CGR-C30A-MedDCM-OCT-S33-C83 (G19)	-	-	-
AP013488	uvMED	EVG	G223	30,646	30.4	uvMED-CGR-C30A-MedDCM-OCT-S38-C82 (G19)	-	-	-
AP013486	uvMED	EVG	G223	30,646	30.5	uvMED-CGR-C30A-MedDCM-OCT-S29-C73 (G19)	-	-	-
AP013490	uvMED	EVG	G223	32,503	31.1	uvMED-CGR-C30B-MedDCM-OCT-S46-C80 (G19)	-	-	-
AP013485	uvMED	EVG	G223	32,761	31.8	uvMED-CGR-C27-MedDCM-OCT-S28-C53 (G19)	-	-	-
TARA_ERS492198_N000331	TOV	EVG	G224	31,747	50.5	-	-	-	-
TARA_ERS490204_N000444	TOV	EVG	G224	32,997	48.9	-	-	-	-
OBV_N00182	OBV	EVG	G224	29,541	43.6	-	-	-	-
TARA_ERS488518_N000609	TOV	EVG	G225	32,171	39.3	-	-	-	-
TARA_ERS478007_N000240	TOV	EVG	G225	32,171	39.4	-	-	-	-
TARA_ERS489603_N000241	TOV	EVG	G225	32,772	40	-	-	-	-
TARA_ERS488354_N000281	TOV	EVG	G225	32,331	40.1	-	-	-	-
TARA_ERS488518_N000643	TOV	EVG	G225	31,217	37.5	-	-	-	-
TARA_ERS478007_N000258	TOV	EVG	G225	31,218	37.6	-	-	-	-
TARA_ERS488448_N000592	TOV	EVG	G225	31,551	37.7	-	-	-	-
TARA_ERS478007_N000266	TOV	EVG	G225	30,858	39.1	-	-	-	-
TARA_ERS488589_N000408	TOV	EVG	G226	28,702	42.4	-	-	-	-
TARA_ERS488354_N000346	TOV	EVG	G226	28,857	40.2	-	-	-	-
TARA_ERS478052_N000618	TOV	EVG	G227	25,609	38.1	-	-	-	-
TARA_ERS488448_N000918	TOV	EVG	G228	24,527	36.2	-	-	-	-
TARA_ERS478007_N000464	TOV	EVG	G228	24,688	36.2	-	-	-	-
TARA_ERS488499_N000539	TOV	EVG	G228	24,832	36.1	-	-	-	-
TARA_ERS478007_N000496	TOV	EVG	G228	24,113	36.2	-	-	-	-
TARA_ERS488737_N000163	TOV	EVG	G229	47,601	37.3	-	-	-	-
TARA_ERS492160_N000651	TOV	EVG	G230	27,335	38	-	-	-	-
TARA_ERS492198_N000457	TOV	EVG	G230	27,445	38	-	-	-	-
TARA_ERS490204_N000595	TOV	EVG	G230	28,540	38.2	-	-	-	-
TARA_ERS478007_N000407	TOV	EVG	G231	26,099	35.1	-	-	-	-
TARA_ERS492160_N000586	TOV	EVG	G231	29,148	38.8	-	-	-	-
NC_009804	RefSeq	RVG	G232	83,319	57.8	Thermus phage P7426	Viruses; dsDNA viruses, no RNA stage; Caudovirales; Siphoviridae; P23likevirus.	466052	Bacteria; Deinococcus-Thermus; Deinococci; Thermales; Thermaceae; Thermus
NC_009803	RefSeq	RVG	G232	84,201	57.8	Thermus phage P2345	Viruses; dsDNA viruses, no RNA stage; Caudovirales; Siphoviridae; P23likevirus.	466051	Bacteria; Deinococcus-Thermus; Deinococci; Thermales; Thermaceae; Thermus
TARA_ERS490494_N000589	TOV	EVG	G233	23,594	40.3	-	-	-	-
AP013531	uvMED	EVG	G234	42,230	48.8	uvMED-CGR-C17C-MedDCM-OCT-S44-C5 (G4)	-	-	-
AP013530	uvMED	EVG	G234	42,200	48.8	uvMED-CGR-C17C-MedDCM-OCT-S23-C4 (G4)	-	-	-
AP013534	uvMED	EVG	G234	41,065	47.2	uvMED-CGR-U-MedDCM-OCT-S40-C11 (G4)	-	-	-
TARA_ERS490557_N000308	TOV	EVG	G234	39,640	42.3	-	-	-	-
TARA_ERS489285_N000262	TOV	EVG	G234	43,161	50.3	-	-	-	-
AP013533	uvMED	EVG	G234	37,887	49.7	uvMED-CGR-U-MedDCM-OCT-S38-C34 (G4)	-	-	-
AP013529	uvMED	EVG	G234	40,572	50.6	uvMED-CGR-U-MedDCM-OCT-S40-C13 (G3)	-	-	-
AP013532	uvMED	EVG	G234	38,241	51.5	uvMED-CGR-U-MedDCM-OCT-S28-C22 (G4)	-	-	-
TARA_ERS488929_N000265	TOV	EVG	G234	34,121	46.8	-	-	-	-
AP013506	uvMED	EVG	G234	38,411	38	uvMED-CGR-C14A-MedDCM-OCT-S42-C35 (G2)	-	-	-
AP013503	uvMED	EVG	G234	38,569	37.9	uvMED-CGR-C14A-MedDCM-OCT-S29-C22 (G2)	-	-	-
AP013505	uvMED	EVG	G234	38,511	37.9	uvMED-CGR-C14A-MedDCM-OCT-S38-C26 (G2)	-	-	-
AP013504	uvMED	EVG	G234	38,514	38	uvMED-CGR-C14A-MedDCM-OCT-S34-C29 (G2)	-	-	-
AP013502	uvMED	EVG	G234	38,514	37.9	uvMED-CGR-C14A-MedDCM-OCT-S23-C10 (G2)	-	-	-
NC_020857	RefSeq	RVG	G234	38,834	37.2	Cyanophage MED4-117	Viruses; dsDNA viruses, no RNA stage; unclassified dsDNA phages.	889954	Bacteria; Cyanobacteria; Prochlorales; Prochlorococcaceae
NC_020847	RefSeq	RVG	G234	38,327	37.2	Prochlorococcus phage MED4-184	Viruses; dsDNA viruses, no RNA stage; Caudovirales; Myoviridae.	889955	Bacteria; Cyanobacteria; Prochlorales; Prochlorococcaceae
AP013508	uvMED	EVG	G234	34,694	36.6	uvMED-CGR-C15J-MedDCM-OCT-S37-C55 (G2)	-	-	-
AP013509	uvMED	EVG	G234	38,614	36.1	uvMED-CGR-U-MedDCM-OCT-S35-C26 (G2)	-	-	-
NC_020854	RefSeq	RVG	G234	40,658	49.1	Cyanophage KBS-S-2A	Viruses; dsDNA viruses, no RNA stage; unclassified dsDNA phages.	889953	Bacteria; Cyanobacteria; Oscillatoriophycideae; Chroococcales; Synechococcus
AP013526	uvMED	EVG	G235	42,608	47	uvMED-CGR-C7A-MedDCM-OCT-S33-C3 (G3)	-	-	-
AP013525	uvMED	EVG	G235	42,608	47	uvMED-CGR-C7A-MedDCM-OCT-S30-C5 (G3)	-	-	-
AP013527	uvMED	EVG	G235	42,638	47.2	uvMED-CGR-C7A-MedDCM-OCT-S38-C7 (G3)	-	-	-
AP013523	uvMED	EVG	G235	41,982	47.7	uvMED-CGR-C7-MedDCM-OCT-S37-C8 (G3)	-	-	-
AP013522	uvMED	EVG	G235	41,754	50.8	uvMED-CGR-C5-MedDCM-OCT-S26-C7 (G3)	-	-	-

TARA_ERS490557_N000292	TOV	EVG	G235	41,049	47.7	-	-	-	-
TARA_ERS489943_N000332	TOV	EVG	G235	43,978	48.3	-	-	-	-
AP013528	uvMED	EVG	G235	39,371	49.5	uvMED-CGR-C7A-MedDCM-OCT-S41-C23 (G3)	-	-	-
AP013524	uvMED	EVG	G235	39,717	49.4	uvMED-CGR-C7-MedDCM-OCT-S39-C15 (G3)	-	-	-
TARA_ERS488757_N000056	TOV	EVG	G235	47,013	51	-	-	-	-
AP013507	uvMED	EVG	G235	37,334	34.4	uvMED-CGR-C15A-MedDCM-OCT-S31-C20 (G2)	-	-	-
TARA_ERS490494_N000116	TOV	EVG	G236	48,246	38.4	-	-	-	-
NC_016766	RefSeq	RVG	G237	69,420	50.8	Synechococcus phage S-CBS4	Viruses; dsDNA viruses, no RNA stage; Caudovirales; Siphoviridae.	756275	Bacteria; Cyanobacteria; Oscillatoriophycideae; Chroococcales; Synechococcus
TARA_ERS490053_N000074	TOV	EVG	G237	71,674	50.3	-	-	-	-
TARA_ERS488929_N000031	TOV	EVG	G238	79,216	40.6	-	-	-	-
TARA_ERS489943_N000131	TOV	EVG	G238	80,215	39.5	-	-	-	-
TARA_ERS490610_N000155	TOV	EVG	G238	74,738	38.6	-	-	-	-
TARA_ERS490452_N000077	TOV	EVG	G238	78,068	38.8	-	-	-	-
TARA_ERS488737_N000081	TOV	EVG	G238	73,663	40.5	-	-	-	-
TARA_ERS488929_N000037	TOV	EVG	G238	73,931	40.5	-	-	-	-
KJ410740	EBI	RVG	G238	79,178	45.9	Synechococcus phage S-EIVl	Viruses; unclassified phages.	1468179	-
TARA_ERS488518_N000139	TOV	EVG	G239	77,117	46.9	-	-	-	-
TARA_ERS490320_N000009	TOV	EVG	G240	81,535	46.4	-	-	-	-
TARA_ERS490285_N000102	TOV	EVG	G240	82,106	46.4	-	-	-	-
TARA_ERS490120_N000131	TOV	EVG	G240	79,807	45.8	-	-	-	-
TARA_ERS489084_N000023	TOV	EVG	G241	84,534	41.8	-	-	-	-
NC_015463	RefSeq	RVG	G242	72,332	54.5	Synechococcus phage S-CBS2	Viruses; dsDNA viruses, no RNA stage; Caudovirales; Siphoviridae.	753084	Bacteria; Cyanobacteria; Oscillatoriophycideae; Chroococcales; Synechococcus
OBV_N00020	OBV	EVG	G242	67,112	45.2	-	-	-	-
NC_013021	RefSeq	RVG	G243	107,530	52.3	Cyanophage PSS2	Viruses; dsDNA viruses, no RNA stage; Caudovirales; Siphoviridae.	658401	Bacteria; Cyanobacteria; Prochlorales; Prochlorococcaceae; Prochlorococcus
AP013372	uvMED	EVG	G244	32,431	35.7	uvMED-CGR-C70A-MedDCM-OCT-S35-C74 (G13)	-	-	-
AP013371	uvMED	EVG	G244	32,391	35.8	uvMED-CGR-C70A-MedDCM-OCT-S25-C64 (G13)	-	-	-
AP013373	uvMED	EVG	G244	32,222	35.8	uvMED-CGR-C70A-MedDCM-OCT-S39-C80 (G13)	-	-	-
TARA_ERS490346_N000625	TOV	EVG	G244	30,272	35.9	-	-	-	-
TARA_ERS490053_N000417	TOV	EVG	G244	33,453	35	-	-	-	-
TARA_ERS490120_N000580	TOV	EVG	G244	33,453	35	-	-	-	-
TARA_ERS492160_N000483	TOV	EVG	G244	33,095	34.6	-	-	-	-
AP013370	uvMED	EVG	G244	30,903	35	uvMED-CGR-C115A-MedDCM-OCT-S39-C87 (G13)	-	-	-
AP013376	uvMED	EVG	G244	34,433	35.1	uvMED-CGR-U-MedDCM-OCT-S34-C52 (G13)	-	-	-
OBV_N00129	OBV	EVG	G244	33,553	35.8	-	-	-	-
TARA_ERS488892_N000275	TOV	EVG	G244	32,925	34.4	-	-	-	-
TARA_ERS488929_N000293	TOV	EVG	G244	33,019	34.4	-	-	-	-
TARA_ERS489285_N000457	TOV	EVG	G244	31,004	34.1	-	-	-	-
TARA_ERS478007_N000157	TOV	EVG	G245	37,052	34.7	-	-	-	-
AP013374	uvMED	EVG	G246	41,867	34.9	uvMED-CGR-C78-MedDCM-OCT-S42-C9 (G13)	-	-	-
TARA_ERS488813_N000160	TOV	EVG	G246	42,770	33.7	-	-	-	-
TARA_ERS488354_N000165	TOV	EVG	G246	40,545	35.1	-	-	-	-
TARA_ERS488757_N000068	TOV	EVG	G246	42,299	34.4	-	-	-	-
AP013377	uvMED	EVG	G247	40,138	34.1	uvMED-CGR-U-MedDCM-OCT-S36-C15 (G13)	-	-	-
AP013375	uvMED	EVG	G248	42,168	36.1	uvMED-CGR-U-MedDCM-OCT-S29-C4 (G13)	-	-	-
TARA_ERS490452_N000237	TOV	EVG	G248	41,608	33.8	-	-	-	-
TARA_ERS490142_N000400	TOV	EVG	G249	38,874	51.2	-	-	-	-
TARA_ERS488701_N000070	TOV	EVG	G250	60,890	47.3	-	-	-	-
TARA_ERS488589_N000268	TOV	EVG	G251	35,237	40	-	-	-	-
TARA_ERS490120_N000496	TOV	EVG	G251	37,062	41.4	-	-	-	-
TARA_ERS490204_N000357	TOV	EVG	G251	35,893	44.7	-	-	-	-
TARA_ERS488340_N000654	TOV	EVG	G251	37,256	38.1	-	-	-	-
TARA_ERS478052_N000276	TOV	EVG	G251	39,280	36.8	-	-	-	-
TARA_ERS489285_N000341	TOV	EVG	G251	37,164	42.3	-	-	-	-
TARA_ERS478007_N000140	TOV	EVG	G251	38,230	38	-	-	-	-
TARA_ERS488929_N000189	TOV	EVG	G251	38,977	37.2	-	-	-	-
TARA_ERS489059_N000150	TOV	EVG	G251	36,380	45.3	-	-	-	-
TARA_ERS489113_N000245	TOV	EVG	G252	37,197	42.6	-	-	-	-
TARA_ERS490610_N000485	TOV	EVG	G253	38,674	54.7	-	-	-	-
AP013368	uvMED	EVG	G254	35,263	41.6	uvMED-CGR-U-MedDCM-OCT-S25-C42 (G11)	-	-	-
OBV_N00104	OBV	EVG	G254	36,709	48.1	-	-	-	-
TARA_ERS489285_N000359	TOV	EVG	G254	35,849	46.8	-	-	-	-
AP013367	uvMED	EVG	G255	39,425	43.8	uvMED-CGR-U-MedDCM-OCT-S31-C11 (G11)	-	-	-
TARA_ERS488813_N000208	TOV	EVG	G256	36,897	46	-	-	-	-
AP013365	uvMED	EVG	G257	39,568	46.3	uvMED-CGR-C52A-MedDCM-OCT-S38-C20 (G11)	-	-	-
AP013363	uvMED	EVG	G257	39,568	46.3	uvMED-CGR-C52A-MedDCM-OCT-S34-C21 (G11)	-	-	-
AP013366	uvMED	EVG	G257	39,473	46.2	uvMED-CGR-C52A-MedDCM-OCT-S42-C25 (G11)	-	-	-
AP013364	uvMED	EVG	G257	39,662	46.3	uvMED-CGR-C52A-MedDCM-OCT-S37-C18 (G11)	-	-	-
AP013362	uvMED	EVG	G257	39,365	46.2	uvMED-CGR-C52A-MedDCM-OCT-S24-C15 (G11)	-	-	-
TARA_ERS488499_N000187	TOV	EVG	G258	42,888	54.8	-	-	-	-
TARA_ERS489603_N000113	TOV	EVG	G259	43,480	47.2	-	-	-	-
TARA_ERS488448_N000289	TOV	EVG	G260	43,719	45.7	-	-	-	-
TARA_ERS490610_N000162	TOV	EVG	G261	72,660	46.3	-	-	-	-
TARA_ERS488813_N000052	TOV	EVG	G262	82,121	43.2	-	-	-	-
TARA_ERS490494_N000034	TOV	EVG	G263	69,874	43.9	-	-	-	-
TARA_ERS488892_N000299	TOV	EVG	G264	31,700	35.2	-	-	-	-

TARA_ERS488813_N000217	TOV	EVG	G265	36,326	50.1	-	-	-	-
TARA_ERS488836_N000163	TOV	EVG	G265	36,326	50.1	-	-	-	-
TARA_ERS489148_N000198	TOV	EVG	G266	41,482	49.8	-	-	-	-
TARA_ERS488892_N000227	TOV	EVG	G267	34,989	50.8	-	-	-	-
TARA_ERS490204_N000361	TOV	EVG	G267	35,714	52.4	-	-	-	-
TARA_ERS490494_N000138	TOV	EVG	G268	45,619	49	-	-	-	-
TARA_ERS490120_N000340	TOV	EVG	G268	46,675	49.3	-	-	-	-
TARA_ERS490494_N000128	TOV	EVG	G268	46,604	46.1	-	-	-	-
TARA_ERS490346_N000305	TOV	EVG	G268	45,583	43.6	-	-	-	-
TARA_ERS489148_N000071	TOV	EVG	G269	58,674	49.5	-	-	-	-
TARA_ERS488836_N000040	TOV	EVG	G269	62,532	49.8	-	-	-	-
TARA_ERS489943_N000186	TOV	EVG	G269	61,512	51.6	-	-	-	-
TARA_ERS492198_N000079	TOV	EVG	G269	57,947	45.8	-	-	-	-
TARA_ERS492160_N000196	TOV	EVG	G269	58,381	45.5	-	-	-	-
TARA_ERS488929_N000056	TOV	EVG	G269	62,593	45.8	-	-	-	-
TARA_ERS492198_N000076	TOV	EVG	G270	58,205	48	-	-	-	-
TARA_ERS490026_N000014	TOV	EVG	G271	66,206	49.5	-	-	-	-
TARA_ERS489148_N000036	TOV	EVG	G272	69,009	49.1	-	-	-	-
TARA_ERS490120_N000337	TOV	EVG	G273	46,837	53.2	-	-	-	-
TARA_ERS490053_N000205	TOV	EVG	G273	46,851	53.2	-	-	-	-
TARA_ERS489943_N000304	TOV	EVG	G273	46,736	53.2	-	-	-	-
TARA_ERS490346_N000255	TOV	EVG	G273	50,688	49.6	-	-	-	-
TARA_ERS489084_N000108	TOV	EVG	G273	46,817	53	-	-	-	-
TARA_ERS488836_N000080	TOV	EVG	G274	48,590	50.4	-	-	-	-
TARA_ERS478007_N000057	TOV	EVG	G275	48,186	36.2	-	-	-	-
TARA_ERS488892_N002322	TOV	EVG	G276	10,441	31.7	-	-	-	-
TARA_ERS488929_N002216	TOV	EVG	G276	10,441	31.7	-	-	-	-
TARA_ERS488518_N003166	TOV	EVG	G276	11,307	36.4	-	-	-	-
NC_016564	RefSeq	RVG	G277	95,299	41.4	Planktothrix phage PaV-LD	Viruses; dsDNA viruses, no RNA stage; Caudovirales; Podoviridae.	994601	Bacteria; Cyanobacteria; Oscillatoriophycideae; Oscillatoriales; Planktothrix
NC_008562	RefSeq	RVG	G278	162,109	46	Microcystis aeruginosa phage Ma-LMM01	Viruses; dsDNA viruses, no RNA stage; Caudovirales; Myoviridae.	340435	Bacteria; Cyanobacteria; Oscillatoriophycideae; Chroococcales; Microcystis
NC_029002	RefSeq	RVG	G278	169,223	46	Microcystis phage MaMV-DC	Viruses; dsDNA viruses, no RNA stage; Caudovirales; Myoviridae.	1357715	-
TARA_ERS489084_N000249	TOV	EVG	G279	36,177	49.9	-	-	-	-
TARA_ERS488929_N000203	TOV	EVG	G280	37,801	53	-	-	-	-
TARA_ERS490204_N000265	TOV	EVG	G281	41,014	52.5	-	-	-	-
TARA_ERS490494_N000122	TOV	EVG	G282	47,795	45.5	-	-	-	-
TARA_ERS488354_N000179	TOV	EVG	G283	38,767	62.6	-	-	-	-
TARA_ERS489603_N000124	TOV	EVG	G284	41,817	56.6	-	-	-	-
TARA_ERS489084_N000339	TOV	EVG	G285	32,334	50.9	-	-	-	-
TARA_ERS488836_N000223	TOV	EVG	G285	32,334	50.9	-	-	-	-
TARA_ERS488813_N000293	TOV	EVG	G285	32,334	51	-	-	-	-
TARA_ERS489059_N000190	TOV	EVG	G285	32,402	48.2	-	-	-	-
TARA_ERS489084_N000270	TOV	EVG	G285	35,228	56.2	-	-	-	-
TARA_ERS489084_N000361	TOV	EVG	G285	31,450	51.7	-	-	-	-
TARA_ERS490494_N000455	TOV	EVG	G286	28,042	57.6	-	-	-	-
TARA_ERS488929_N000345	TOV	EVG	G286	30,090	56.2	-	-	-	-
TARA_ERS489084_N000385	TOV	EVG	G286	30,667	61.3	-	-	-	-
TARA_ERS490320_N000167	TOV	EVG	G287	33,881	58.7	-	-	-	-
TARA_ERS490285_N000472	TOV	EVG	G287	33,899	58.7	-	-	-	-
TARA_ERS488701_N000336	TOV	EVG	G287	31,545	58.8	-	-	-	-
TARA_ERS488892_N000331	TOV	EVG	G288	30,314	47.7	-	-	-	-
OBV_N00186	OBV	EVG	G288	29,180	43.5	-	-	-	-
OBV_N00390	OBV	EVG	G288	29,867	48	-	-	-	-
TARA_ERS490346_N000628	TOV	EVG	G288	30,178	44	-	-	-	-
TARA_ERS488673_N000513	TOV	EVG	G288	31,318	44.7	-	-	-	-
OBV_N00160	OBV	EVG	G288	30,759	44.1	-	-	-	-
TARA_ERS488737_N000343	TOV	EVG	G288	30,054	43.5	-	-	-	-
TARA_ERS488892_N000335	TOV	EVG	G288	30,230	36.8	-	-	-	-
TARA_ERS490610_N000745	TOV	EVG	G288	30,810	38.4	-	-	-	-
TARA_ERS488813_N000072	TOV	EVG	G289	62,128	38.3	-	-	-	-
TARA_ERS488892_N000402	TOV	EVG	G290	27,452	50.9	-	-	-	-
TARA_ERS488929_N000403	TOV	EVG	G290	27,452	50.9	-	-	-	-
TARA_ERS489084_N000485	TOV	EVG	G290	27,452	51	-	-	-	-
TARA_ERS492198_N000432	TOV	EVG	G290	28,262	56.9	-	-	-	-
TARA_ERS489148_N000399	TOV	EVG	G290	30,780	53.3	-	-	-	-
TARA_ERS489943_N000690	TOV	EVG	G290	27,369	56.3	-	-	-	-
TARA_ERS490053_N000221	TOV	EVG	G291	44,708	60.6	-	-	-	-
TARA_ERS490204_N000597	TOV	EVG	G292	28,479	45.5	-	-	-	-
TARA_ERS489285_N000096	TOV	EVG	G293	74,025	41.1	-	-	-	-
TARA_ERS488892_N000075	TOV	EVG	G294	54,621	55.1	-	-	-	-
TARA_ERS488929_N000077	TOV	EVG	G294	55,827	55.2	-	-	-	-
TARA_ERS492198_N000090	TOV	EVG	G294	54,246	55.1	-	-	-	-
TARA_ERS488929_N000092	TOV	EVG	G294	52,225	52.3	-	-	-	-
TARA_ERS490494_N000078	TOV	EVG	G294	55,035	54.8	-	-	-	-
TARA_ERS490026_N000026	TOV	EVG	G294	58,372	53.8	-	-	-	-
TARA_ERS488836_N000066	TOV	EVG	G294	51,907	53.7	-	-	-	-

TARA_ERS490026_N000033	TOV	EVG	G294	55,077	57	-	-	-	-
TARA_ERS492160_N000229	TOV	EVG	G294	52,230	48.1	-	-	-	-
TARA_ERS488836_N000059	TOV	EVG	G295	54,993	49.7	-	-	-	-
TARA_ERS488929_N000079	TOV	EVG	G295	54,994	49.7	-	-	-	-
TARA_ERS488892_N000073	TOV	EVG	G295	54,994	49.7	-	-	-	-
TARA_ERS488701_N000097	TOV	EVG	G295	55,830	47.5	-	-	-	-
TARA_ERS489943_N000266	TOV	EVG	G295	50,973	52.2	-	-	-	-
TARA_ERS490494_N000056	TOV	EVG	G295	58,693	51.7	-	-	-	-
TARA_ERS488589_N000094	TOV	EVG	G295	53,043	51.4	-	-	-	-
TARA_ERS478052_N000120	TOV	EVG	G296	58,534	49.9	-	-	-	-
OBV_N00033	OBV	EVG	G296	57,810	50.5	-	-	-	-
TARA_ERS488448_N000158	TOV	EVG	G296	55,153	47	-	-	-	-
TARA_ERS489084_N000034	TOV	EVG	G297	67,754	54.5	-	-	-	-
TARA_ERS488929_N000042	TOV	EVG	G297	68,253	51.9	-	-	-	-
TARA_ERS490452_N000094	TOV	EVG	G297	71,241	49	-	-	-	-
TARA_ERS488558_N000259	TOV	EVG	G297	74,393	47.5	-	-	-	-
TARA_ERS489059_N000048	TOV	EVG	G297	62,932	54.4	-	-	-	-
TARA_ERS489148_N000002	TOV	EVG	G298	169,374	49.1	-	-	-	-
TARA_ERS490494_N000095	TOV	EVG	G299	51,580	41.8	-	-	-	-
TARA_ERS490204_N000158	TOV	EVG	G300	51,988	55.8	-	-	-	-
TARA_ERS478052_N000180	TOV	EVG	G301	49,708	40.3	-	-	-	-
TARA_ERS488836_N000075	TOV	EVG	G302	49,521	56.8	-	-	-	-
TARA_ERS488929_N000299	TOV	EVG	G303	32,880	41.2	-	-	-	-
TARA_ERS488673_N000474	TOV	EVG	G303	33,104	41.4	-	-	-	-
TARA_ERS488813_N000266	TOV	EVG	G303	33,657	40.6	-	-	-	-
TARA_ERS488836_N000220	TOV	EVG	G303	32,634	39.1	-	-	-	-
TARA_ERS488673_N000529	TOV	EVG	G304	30,488	36.8	-	-	-	-
TARA_ERS490204_N000508	TOV	EVG	G304	30,894	38.8	-	-	-	-
TARA_ERS488836_N000287	TOV	EVG	G305	28,859	57.9	-	-	-	-
TARA_ERS492198_N000408	TOV	EVG	G305	28,874	58.4	-	-	-	-
TARA_ERS490204_N000582	TOV	EVG	G305	28,798	62.1	-	-	-	-
TARA_ERS490204_N000616	TOV	EVG	G305	27,888	58.6	-	-	-	-
TARA_ERS488613_N000318	TOV	EVG	G306	33,483	60.8	-	-	-	-
TARA_ERS490026_N000182	TOV	EVG	G307	33,836	40	-	-	-	-
TARA_ERS489943_N000494	TOV	EVG	G307	33,848	40	-	-	-	-
TARA_ERS488929_N000254	TOV	EVG	G307	34,671	42.7	-	-	-	-
TARA_ERS492160_N000509	TOV	EVG	G307	31,720	41.2	-	-	-	-
TARA_ERS489084_N000260	TOV	EVG	G307	35,589	39.3	-	-	-	-
TARA_ERS489943_N000537	TOV	EVG	G307	32,138	38.5	-	-	-	-
TARA_ERS488448_N000661	TOV	EVG	G307	30,137	40.9	-	-	-	-
OBV_N00155	OBV	EVG	G308	31,018	43.6	-	-	-	-
TARA_ERS492160_N000545	TOV	EVG	G309	30,375	44.7	-	-	-	-
TARA_ERS488892_N000303	TOV	EVG	G310	31,480	45.4	-	-	-	-
TARA_ERS490494_N000033	TOV	EVG	G311	70,126	47.6	-	-	-	-
TARA_ERS488701_N000045	TOV	EVG	G311	74,428	52.8	-	-	-	-
TARA_ERS490346_N000131	TOV	EVG	G312	70,111	50.1	-	-	-	-
TARA_ERS490494_N000038	TOV	EVG	G313	67,356	43.9	-	-	-	-
TARA_ERS488589_N000118	TOV	EVG	G314	49,772	39.4	-	-	-	-
TARA_ERS488701_N000123	TOV	EVG	G314	49,772	39.4	-	-	-	-
TARA_ERS490053_N000175	TOV	EVG	G315	50,455	39.7	-	-	-	-
TARA_ERS490120_N000294	TOV	EVG	G315	50,584	39.7	-	-	-	-
TARA_ERS490053_N000308	TOV	EVG	G316	38,072	40.6	-	-	-	-
TARA_ERS490120_N000476	TOV	EVG	G316	38,081	40.6	-	-	-	-
TARA_ERS489285_N000382	TOV	EVG	G317	34,776	49.9	-	-	-	-
TARA_ERS492160_N000440	TOV	EVG	G317	34,957	49.8	-	-	-	-
TARA_ERS490494_N000278	TOV	EVG	G317	35,647	46.6	-	-	-	-
TARA_ERS488589_N000259	TOV	EVG	G317	35,889	57.6	-	-	-	-
TARA_ERS490026_N000122	TOV	EVG	G318	39,123	54.7	-	-	-	-
LDNP01000004	cryoconite	EVG	G319	38,898	37.4	-	-	-	-
TARA_ERS492160_N000427	TOV	EVG	G320	35,470	38.1	-	-	-	-
TARA_ERS490494_N000240	TOV	EVG	G320	37,582	43	-	-	-	-
TARA_ERS490320_N000147	TOV	EVG	G321	35,719	42.4	-	-	-	-
TARA_ERS490285_N000417	TOV	EVG	G322	36,335	46.8	-	-	-	-
TARA_ERS489148_N000210	TOV	EVG	G322	40,290	44.2	-	-	-	-
TARA_ERS488673_N000360	TOV	EVG	G322	40,078	43	-	-	-	-
TARA_ERS490180_N000357	TOV	EVG	G322	38,287	46.7	-	-	-	-
TARA_ERS488518_N000439	TOV	EVG	G322	38,964	59	-	-	-	-
TARA_ERS489285_N000328	TOV	EVG	G323	37,929	58.3	-	-	-	-
LDNN01000003	cryoconite	EVG	G324	80,578	60.3	-	-	-	-
TARA_ERS488929_N000059	TOV	EVG	G325	60,336	43.4	-	-	-	-
TARA_ERS488589_N000084	TOV	EVG	G326	53,574	56.9	-	-	-	-
TARA_ERS490026_N000035	TOV	EVG	G326	54,173	53.3	-	-	-	-
TARA_ERS489603_N000192	TOV	EVG	G327	35,238	60.5	-	-	-	-
TARA_ERS490557_N000312	TOV	EVG	G328	39,195	57.8	-	-	-	-
TARA_ERS490610_N000471	TOV	EVG	G328	39,247	56.9	-	-	-	-

TARA_ERS490610_N000500	TOV	EVG	G328	38,469	59.4	-	-	-	-
TARA_ERS488558_N000741	TOV	EVG	G329	37,261	54.1	-	-	-	-
TARA_ERS492198_N000239	TOV	EVG	G330	36,628	49.1	-	-	-	-
TARA_ERS489084_N000206	TOV	EVG	G331	38,435	55.3	-	-	-	-
TARA_ERS490557_N000379	TOV	EVG	G332	35,469	51.6	-	-	-	-
TARA_ERS489113_N000332	TOV	EVG	G333	32,065	47.9	-	-	-	-
TARA_ERS490285_N000466	TOV	EVG	G334	34,049	52.9	-	-	-	-
TARA_ERS492160_N000399	TOV	EVG	G335	36,997	50.3	-	-	-	-
TARA_ERS492198_N000232	TOV	EVG	G335	37,032	50.3	-	-	-	-
TARA_ERS488929_N000194	TOV	EVG	G335	38,586	50.7	-	-	-	-
TARA_ERS489084_N000203	TOV	EVG	G335	38,586	50.7	-	-	-	-
TARA_ERS492198_N000179	TOV	EVG	G335	41,081	47	-	-	-	-
TARA_ERS489148_N000176	TOV	EVG	G336	42,895	49.2	-	-	-	-
TARA_ERS488701_N000222	TOV	EVG	G337	37,785	50.7	-	-	-	-
TARA_ERS489148_N000211	TOV	EVG	G337	40,252	47.5	-	-	-	-
TARA_ERS490557_N000211	TOV	EVG	G338	48,925	51.4	-	-	-	-
TARA_ERS490610_N000314	TOV	EVG	G338	48,925	51.4	-	-	-	-
TARA_ERS488589_N000182	TOV	EVG	G339	42,481	50.7	-	-	-	-
TARA_ERS488701_N000186	TOV	EVG	G340	40,290	54.6	-	-	-	-
TARA_ERS488701_N000192	TOV	EVG	G341	39,514	39.3	-	-	-	-
LDNO01000006	cryoconite	EVG	G342	43,293	50	-	-	-	-
TARA_ERS488929_N000127	TOV	EVG	G343	45,532	57.4	-	-	-	-
TARA_ERS490053_N000286	TOV	EVG	G344	39,789	44.7	-	-	-	-
TARA_ERS490120_N000436	TOV	EVG	G344	39,789	44.7	-	-	-	-
TARA_ERS490120_N000531	TOV	EVG	G345	34,952	38.1	-	-	-	-
TARA_ERS490204_N000380	TOV	EVG	G345	34,952	38	-	-	-	-
TARA_ERS490494_N000209	TOV	EVG	G346	39,250	50.5	-	-	-	-
AP013361	uvMED	EVG	G347	43,957	53	uvMED-CGR-U-MedDCM-OCT-S34-C1 (G10)	-	-	-
TARA_ERS492198_N000125	TOV	EVG	G347	48,011	51.8	-	-	-	-
TARA_ERS492198_N000038	TOV	EVG	G348	75,141	49.2	-	-	-	-
TARA_ERS489285_N001716	TOV	EVG	G349	12,182	35	-	-	-	-
NC_005964	RefSeq	RVG	G350	18,855	24.8	Mycoplasma phage phiMFV1	Viruses; unclassified phages.	280702	Bacteria; Tenericutes; Mollicutes; Mycoplasmatales; Mycoplasmataceae
NC_001942	RefSeq	RVG	G351	15,644	29	Mycoplasma phage MAV1	Viruses; dsDNA viruses, no RNA stage; unclassified dsDNA phages.	75590	Bacteria; Tenericutes; Mollicutes; Mycoplasmatales; Mycoplasmataceae; Mycoplasma
TARA_ERS490610_N000465	TOV	EVG	G352	39,474	38	-	-	-	-
TARA_ERS490557_N000299	TOV	EVG	G352	40,311	38.1	-	-	-	-
TARA_ERS490494_N000185	TOV	EVG	G353	40,893	35.8	-	-	-	-
TARA_ERS489943_N000345	TOV	EVG	G354	43,106	36.4	-	-	-	-
TARA_ERS490320_N000155	TOV	EVG	G355	34,987	37.1	-	-	-	-
TARA_ERS490494_N000043	TOV	EVG	G356	63,763	33.5	-	-	-	-
TARA_ERS488701_N000184	TOV	EVG	G357	40,973	41.7	-	-	-	-
TARA_ERS490026_N000105	TOV	EVG	G357	41,065	41.6	-	-	-	-
TARA_ERS488701_N000185	TOV	EVG	G358	40,865	45.9	-	-	-	-
TARA_ERS488589_N000216	TOV	EVG	G359	38,771	39.3	-	-	-	-
TARA_ERS490053_N000281	TOV	EVG	G360	40,075	39	-	-	-	-
TARA_ERS489943_N000435	TOV	EVG	G361	37,638	49	-	-	-	-
TARA_ERS490494_N000175	TOV	EVG	G362	41,798	44.4	-	-	-	-
TARA_ERS492198_N000353	TOV	EVG	G363	30,874	60.1	-	-	-	-
TARA_ERS488448_N000605	TOV	EVG	G363	31,275	53.1	-	-	-	-
TARA_ERS488518_N000626	TOV	EVG	G363	31,683	51.6	-	-	-	-
TARA_ERS478007_N000249	TOV	EVG	G363	31,683	51.5	-	-	-	-
TARA_ERS488892_N000238	TOV	EVG	G364	34,562	56.7	-	-	-	-
TARA_ERS489084_N000290	TOV	EVG	G364	34,562	56.7	-	-	-	-
TARA_ERS490494_N000308	TOV	EVG	G364	34,438	51.2	-	-	-	-
TARA_ERS490320_N000260	TOV	EVG	G365	27,972	43.9	-	-	-	-
TARA_ERS489084_N000420	TOV	EVG	G365	29,534	45.1	-	-	-	-
TARA_ERS490610_N000518	TOV	EVG	G366	37,979	51.1	-	-	-	-
TARA_ERS490494_N000082	TOV	EVG	G367	53,458	48.3	-	-	-	-
TARA_ERS489603_N000060	TOV	EVG	G367	54,249	50.1	-	-	-	-
TARA_ERS490494_N000062	TOV	EVG	G367	57,557	39.1	-	-	-	-
TARA_ERS478052_N000165	TOV	EVG	G367	51,737	53.5	-	-	-	-
TARA_ERS489084_N000091	TOV	EVG	G367	49,729	52.7	-	-	-	-
TARA_ERS489603_N000081	TOV	EVG	G368	49,913	56.5	-	-	-	-
TARA_ERS488589_N000149	TOV	EVG	G369	44,883	54.8	-	-	-	-
TARA_ERS488448_N000263	TOV	EVG	G370	45,211	55.8	-	-	-	-
TARA_ERS490346_N000301	TOV	EVG	G371	45,866	48.6	-	-	-	-
TARA_ERS490494_N000133	TOV	EVG	G371	46,209	48.6	-	-	-	-
TARA_ERS489059_N000109	TOV	EVG	G372	42,350	50.6	-	-	-	-
TARA_ERS492160_N000323	TOV	EVG	G372	42,357	54.1	-	-	-	-
TARA_ERS488836_N000113	TOV	EVG	G373	42,463	56.7	-	-	-	-
TARA_ERS492198_N000159	TOV	EVG	G374	43,309	56	-	-	-	-
TARA_ERS488701_N000119	TOV	EVG	G375	50,828	58	-	-	-	-
TARA_ERS489084_N000117	TOV	EVG	G376	45,939	54.2	-	-	-	-
TARA_ERS490026_N000057	TOV	EVG	G376	47,561	46.2	-	-	-	-
TARA_ERS490610_N000186	TOV	EVG	G377	63,944	49.7	-	-	-	-
TARA_ERS489084_N000251	TOV	EVG	G378	36,134	39.2	-	-	-	-

TARA_ERS488813_N000185	TOV	EVG	G379	39,335	53.3	-	-	-	-
TARA_ERS488613_N000132	TOV	EVG	G380	58,243	45	-	-	-	-
TARA_ERS490610_N000218	TOV	EVG	G380	60,050	45.4	-	-	-	-
TARA_ERS489943_N000223	TOV	EVG	G380	55,710	44.7	-	-	-	-
TARA_ERS490610_N000247	TOV	EVG	G380	56,346	48.8	-	-	-	-
TARA_ERS489285_N000140	TOV	EVG	G381	58,844	52.5	-	-	-	-
TARA_ERS490204_N000228	TOV	EVG	G382	43,858	54.2	-	-	-	-
TARA_ERS488518_N000337	TOV	EVG	G382	44,636	54.1	-	-	-	-
NC_012635	RefSeq	RVG	G383	168,394	35.3	Enterobacteria phage RB51	Viruses; dsDNA viruses, no RNA stage; Caudovirales; Myoviridae; Tevenvirinae; T4likevirus.	10693	Bacteria; Proteobacteria; Gammaproteobacteria; Enterobacteriales; Enterobacteriaceae; Escherichia
NC_027979	RefSeq	RVG	G383	168,401	35.3	Enterobacteria phage RB68	Viruses; dsDNA viruses, no RNA stage; Caudovirales; Myoviridae; Tevenvirinae; T4likevirus.	36339	Bacteria; Proteobacteria; Gammaproteobacteria; Enterobacteriales; Enterobacteriaceae; Escherichia
NC_019505	RefSeq	RVG	G383	166,452	35.4	Escherichia phage wV7	Viruses; dsDNA viruses, no RNA stage; Caudovirales; Myoviridae; Tevenvirinae; T4likevirus.	1054480	Bacteria; Proteobacteria; Gammaproteobacteria; Enterobacteriales; Enterobacteriaceae; Escherichia
NC_027983	RefSeq	RVG	G383	167,435	35.3	Enterobacteria phage AR1	Viruses; dsDNA viruses, no RNA stage; Caudovirales; Myoviridae; Tevenvirinae; T4likevirus.	66711	Bacteria; Proteobacteria; Gammaproteobacteria; Enterobacteriales; Enterobacteriaceae; Escherichia
NC_025448	RefSeq	RVG	G383	165,179	35.5	Enterobacteria phage RB27	Viruses; dsDNA viruses, no RNA stage; Caudovirales; Myoviridae; Tevenvirinae; T4likevirus.	69609	Bacteria; Proteobacteria; Gammaproteobacteria; Enterobacteriales; Enterobacteriaceae; Escherichia
NC_027349	RefSeq	RVG	G383	166,977	35.5	Escherichia phage HY01	Viruses; dsDNA viruses, no RNA stage; Caudovirales; Myoviridae.	1434323	Bacteria; Proteobacteria; Gammaproteobacteria; Enterobacteriales; Enterobacteriaceae; Escherichia
NC_025829	RefSeq	RVG	G383	164,999	35.5	Shigella phage pSs-1	Viruses; dsDNA viruses, no RNA stage; Caudovirales; Myoviridae.	1551641	Bacteria; Proteobacteria; Gammaproteobacteria; Enterobacteriales; Enterobacteriaceae; Shigella
NC_024125	RefSeq	RVG	G383	168,470	35.3	Escherichia phage e11/2	Viruses; dsDNA viruses, no RNA stage; Caudovirales; Myoviridae; Tevenvirinae; T4likevirus.	1495285	Bacteria; Proteobacteria; Gammaproteobacteria; Enterobacteriales; Enterobacteriaceae; Escherichia
NC_015457	RefSeq	RVG	G383	165,919	35.6	Shigella phage Shfl2	Viruses; dsDNA viruses, no RNA stage; Caudovirales; Myoviridae; Tevenvirinae; T4likevirus.	1002725	Bacteria; Proteobacteria; Gammaproteobacteria; Enterobacteriales; Enterobacteriaceae; Shigella
NC_028927	RefSeq	RVG	G383	167,298	35.5	Escherichia phage slur02	Viruses; dsDNA viruses, no RNA stage; Caudovirales; Myoviridae; Tevenvirinae; T4likevirus.	1720494	-
NC_028780	RefSeq	RVG	G383	167,124	35.4	Escherichia phage slur07	Viruses; dsDNA viruses, no RNA stage; Caudovirales; Myoviridae; Tevenvirinae; T4likevirus.	1720500	-
NC_019503	RefSeq	RVG	G383	166,499	35.7	Enterobacteria phage ime09	Viruses; dsDNA viruses, no RNA stage; Caudovirales; Myoviridae; Tevenvirinae; T4likevirus.	1054834	Bacteria; Proteobacteria; Gammaproteobacteria; Enterobacteriales; Enterobacteriaceae; Escherichia
NC_028448	RefSeq	RVG	G383	167,467	35.4	Escherichia phage slur14	Viruses; dsDNA viruses, no RNA stage; Caudovirales; Myoviridae; Tevenvirinae; T4likevirus.	1720504	-
NC_027353	RefSeq	RVG	G383	167,063	35.4	Yersinia phage phiD1	Viruses; dsDNA viruses, no RNA stage; Caudovirales; Myoviridae.	1206560	Bacteria; Proteobacteria; Gammaproteobacteria; Enterobacteriales; Enterobacteriaceae; Yersinia; Yersinia pseudotuberculosis complex
KM606999	EBI	RVG	G383	168,401	35.4	Enterobacteria phage RB10	Viruses; dsDNA viruses, no RNA stage; Caudovirales; Myoviridae; Tevenvirinae; T4likevirus.	69608	-
KM606997	EBI	RVG	G383	168,395	35.4	Enterobacteria phage RB7	Viruses; dsDNA viruses, no RNA stage; Caudovirales; Myoviridae; Tevenvirinae; T4likevirus.	697291	-
KM606998	EBI	RVG	G383	168,395	35.4	Enterobacteria phage RB9	Viruses; dsDNA viruses, no RNA stage; Caudovirales; Myoviridae; Tevenvirinae; T4likevirus.	69612	-
KM606995	EBI	RVG	G383	168,394	35.4	Enterobacteria phage RB5	Viruses; dsDNA viruses, no RNA stage; Caudovirales; Myoviridae; Tevenvirinae; T4likevirus.	1112578	-
NC_025419	RefSeq	RVG	G383	168,402	35.4	Enterobacteria phage RB3	Viruses; dsDNA viruses, no RNA stage; Caudovirales; Myoviridae; Tevenvirinae; T4likevirus.	31533	Bacteria; Proteobacteria; Gammaproteobacteria; Enterobacteriales; Enterobacteriaceae; Escherichia
KM606996	EBI	RVG	G383	168,394	35.4	Enterobacteria phage RB6	Viruses; dsDNA viruses, no RNA stage; Caudovirales; Myoviridae; Tevenvirinae; T4likevirus.	69610	-
NC_012638	RefSeq	RVG	G383	165,429	35.3	Enterobacteria phage RB14	Viruses; dsDNA viruses, no RNA stage; Caudovirales; Myoviridae; Tevenvirinae; T4likevirus.	197310	Bacteria; Proteobacteria; Gammaproteobacteria
KM607001	EBI	RVG	G383	166,007	35.3	Enterobacteria phage RB33	Viruses; dsDNA viruses, no RNA stage; Caudovirales; Myoviridae; Tevenvirinae; T4likevirus.	134822	-
NC_008515	RefSeq	RVG	G383	165,890	35.3	Enterobacteria phage RB32	Viruses; dsDNA viruses, no RNA stage; Caudovirales; Myoviridae; Tevenvirinae; T4likevirus.	45406	Bacteria; Proteobacteria; Gammaproteobacteria; Enterobacteriales; Enterobacteriaceae; Escherichia
NC_025449	RefSeq	RVG	G383	166,783	35.4	Escherichia phage ECML-134	Viruses; dsDNA viruses, no RNA stage; Caudovirales; Myoviridae; Tevenvirinae; T4likevirus.	1204522	Bacteria; Proteobacteria; Gammaproteobacteria; Enterobacteriales; Enterobacteriaceae; Escherichia
NC_027404	RefSeq	RVG	G383	167,785	35.3	Yersinia phage PST	Viruses; dsDNA viruses, no RNA stage; Caudovirales; Myoviridae; Tevenvirinae; T4likevirus.	1351740	Bacteria; Proteobacteria; Gammaproteobacteria; Enterobacteriales; Enterobacteriaceae; Yersinia; Yersinia pseudotuberculosis complex
NC_019399	RefSeq	RVG	G383	167,396	35.2	Enterobacteria phage vB_EcoM_ACG-C40	Viruses; dsDNA viruses, no RNA stage; Caudovirales; Myoviridae; Tevenvirinae; T4likevirus.	1141141	Bacteria; Proteobacteria; Gammaproteobacteria; Enterobacteriales; Enterobacteriaceae; Escherichia
KM607002	EBI	RVG	G383	168,896	35.3	Enterobacteria phage RB55	Viruses; dsDNA viruses, no RNA stage; Caudovirales; Myoviridae; Tevenvirinae; T4likevirus.	697289	-
NC_000866	RefSeq	RVG	G383	168,903	35.3	Enterobacteria phage T4	Viruses; dsDNA viruses, no RNA stage; Caudovirales; Myoviridae; Tevenvirinae; T4likevirus.	10665	Bacteria; Proteobacteria; Gammaproteobacteria; Enterobacteriales; Enterobacteriaceae; Escherichia
HM137666	EBI	RVG	G383	168,920	35.3	Enterobacteria phage T4T	Viruses; dsDNA viruses, no RNA stage; Caudovirales; Myoviridae; Tevenvirinae; T4likevirus.	857277	-
KM607003	EBI	RVG	G383	168,966	35.3	Enterobacteria phage RB59	Viruses; dsDNA viruses, no RNA stage; Caudovirales; Myoviridae; Tevenvirinae; T4likevirus.	697290	-
KP869104	EBI	RVG	G383	160,570	37.6	Escherichia coli O157 typing phage 6	Viruses; dsDNA viruses, no RNA stage; Caudovirales; Myoviridae.	1508681	-
KP869101	EBI	RVG	G383	168,733	37.6	Escherichia coli O157 typing phage 3	Viruses; dsDNA viruses, no RNA stage; Caudovirales; Myoviridae.	1508678	-
NC_029091	RefSeq	RVG	G383	168,771	37.7	Escherichia phage APCEc01	Viruses; dsDNA viruses, no RNA stage; Caudovirales; Myoviridae.	1655305	-
NC_024124	RefSeq	RVG	G383	169,148	37.6	Escherichia phage vB_EcoM_JS09	Viruses; dsDNA viruses, no RNA stage; Caudovirales; Myoviridae; Tevenvirinae; T4likevirus.	1430444	Bacteria; Proteobacteria; Gammaproteobacteria; Enterobacteriales; Enterobacteriaceae; Escherichia

NC_018855	RefSeq	RVG	G383	169,158	37.6	Enterobacteria phage HX01	Viruses; dsDNA viruses, no RNA stage; Caudovirales; Myoviridae; Tevenvirinae; T4likevirus.	1237364	Bacteria; Proteobacteria; Gammaproteobacteria; Enterobacteriales; Enterobacteriaceae; Escherichia
NC_025437	RefSeq	RVG	G383	169,062	37.6	Shigella phage Shf125875	Viruses; dsDNA viruses, no RNA stage; Caudovirales; Myoviridae.	1541825	Bacteria; Proteobacteria; Gammaproteobacteria; Enterobacteriales; Enterobacteriaceae; Shigella
NC_024794	RefSeq	RVG	G383	167,318	37.7	Escherichia phage vB_EcoM_PhAPEC2	Viruses; dsDNA viruses, no RNA stage; Caudovirales; Myoviridae; Tevenvirinae; T4likevirus.	1391224	Bacteria; Proteobacteria; Gammaproteobacteria; Enterobacteriales; Enterobacteriaceae; Escherichia
NC_004928	RefSeq	RVG	G383	167,560	37.7	Enterobacteria phage RB69	Viruses; dsDNA viruses, no RNA stage; Caudovirales; Myoviridae; Tevenvirinae; T4likevirus.	12353	Bacteria; Proteobacteria; Gammaproteobacteria; Enterobacteriales; Enterobacteriaceae; Escherichia
NC_019500	RefSeq	RVG	G383	168,066	39.5	Enterobacteria phage Bp7	Viruses; dsDNA viruses, no RNA stage; Caudovirales; Myoviridae; Tevenvirinae; T4likevirus.	1052121	Bacteria; Proteobacteria; Gammaproteobacteria; Enterobacteriales; Enterobacteriaceae; Escherichia
NC_028847	RefSeq	RVG	G383	170,527	39.6	Enterobacteria phage QL01	Viruses; dsDNA viruses, no RNA stage; Caudovirales; Myoviridae; Tevenvirinae; T4likevirus.	1673871	-
NC_012741	RefSeq	RVG	G383	171,451	39.5	Enterobacteria phage JS10	Viruses; dsDNA viruses, no RNA stage; Caudovirales; Myoviridae; Tevenvirinae; T4likevirus.	576790	Bacteria; Proteobacteria; Gammaproteobacteria; Enterobacteriales; Enterobacteriaceae; Escherichia
NC_010105	RefSeq	RVG	G383	170,523	39.5	Enterobacteria phage JS98	Viruses; dsDNA viruses, no RNA stage; Caudovirales; Myoviridae; Tevenvirinae; T4likevirus.	293178	Bacteria; Proteobacteria; Gammaproteobacteria; Enterobacteriales; Enterobacteriaceae; Escherichia
NC_014260	RefSeq	RVG	G383	172,253	39.6	Enterobacteria phage IME08	Viruses; dsDNA viruses, no RNA stage; Caudovirales; Myoviridae; Tevenvirinae; T4likevirus.	697227	Bacteria; Proteobacteria; Gammaproteobacteria; Enterobacteriales; Enterobacteriaceae; Escherichia
NC_028881	RefSeq	RVG	G383	170,473	39.3	Enterobacteria phage vB_EcoM_VR5	Viruses; dsDNA viruses, no RNA stage; Caudovirales; Myoviridae; Tevenvirinae; T4likevirus.	1567026	-
NC_028925	RefSeq	RVG	G383	170,822	40.4	Enterobacteria phage vB_EcoM_VR25	Viruses; dsDNA viruses, no RNA stage; Caudovirales; Myoviridae; Tevenvirinae; T4likevirus.	1567028	-
NC_014792	RefSeq	RVG	G383	169,285	40.3	Enterobacteria phage vB_EcoM-VR7	Viruses; dsDNA viruses, no RNA stage; Caudovirales; Myoviridae; Tevenvirinae; T4likevirus.	700939	Bacteria; Proteobacteria; Gammaproteobacteria; Enterobacteriales; Enterobacteriaceae; Escherichia
NC_028957	RefSeq	RVG	G383	171,541	40.2	Enterobacteria phage vB_EcoM_VR26	Viruses; dsDNA viruses, no RNA stage; Caudovirales; Myoviridae; Tevenvirinae; T4likevirus.	1567029	-
NC_014595	RefSeq	RVG	G383	170,605	40.4	Shigella phage SP18	Viruses; dsDNA viruses, no RNA stage; Caudovirales; Myoviridae; Tevenvirinae; T4likevirus.	645664	Bacteria; Proteobacteria; Gammaproteobacteria; Enterobacteriales; Enterobacteriaceae; Shigella
NC_028894	RefSeq	RVG	G383	170,336	40.4	Enterobacteria phage vB_EcoM_VR20	Viruses; dsDNA viruses, no RNA stage; Caudovirales; Myoviridae; Tevenvirinae; T4likevirus.	1567027	-
NC_028940	RefSeq	RVG	G383	170,286	34.8	Pectobacterium bacteriophage PM2	Viruses; dsDNA viruses, no RNA stage; Caudovirales; Myoviridae.	1429794	-
NC_028683	RefSeq	RVG	G383	177,643	40.6	Edwardsiella phage PEi20	Viruses; dsDNA viruses, no RNA stage; Caudovirales; Myoviridae.	1608310	-
NC_020416	RefSeq	RVG	G383	160,221	36.9	Salmonella phage S16	Viruses; dsDNA viruses, no RNA stage; Caudovirales; Myoviridae; Tevenvirinae; T4likevirus.	1087482	Bacteria; Proteobacteria; Gammaproteobacteria; Enterobacteriales; Enterobacteriaceae; Salmonella
NC_026607	RefSeq	RVG	G383	159,914	36.9	Salmonella phage STP4-a	Viruses; dsDNA viruses, no RNA stage; Caudovirales; Myoviridae; Tevenvirinae; T4likevirus.	1445860	Bacteria; Proteobacteria; Gammaproteobacteria; Enterobacteriales; Enterobacteriaceae; Salmonella; Salmonella enterica
NC_027344	RefSeq	RVG	G383	158,099	36.9	Salmonella phage STML-198	Viruses; dsDNA viruses, no RNA stage; Caudovirales; Myoviridae.	1204531	Bacteria; Proteobacteria; Gammaproteobacteria; Enterobacteriales; Enterobacteriaceae
NC_028857	RefSeq	RVG	G383	172,733	38.8	Citrobacter phage Merlin	Viruses; dsDNA viruses, no RNA stage; Caudovirales; Myoviridae.	1675602	-
NC_027331	RefSeq	RVG	G383	170,341	38.9	Citrobacter phage Moon	Viruses; dsDNA viruses, no RNA stage; Caudovirales; Myoviridae.	1540095	Bacteria; Proteobacteria; Gammaproteobacteria; Enterobacteriales; Enterobacteriaceae; Citrobacter
NC_014662	RefSeq	RVG	G383	165,540	39.9	Enterobacteria phage CC31	Viruses; dsDNA viruses, no RNA stage; Caudovirales; Myoviridae; Tevenvirinae; T4likevirus.	709484	Bacteria; Proteobacteria; Gammaproteobacteria; Enterobacteriales; Enterobacteriaceae; Escherichia
NC_023561	RefSeq	RVG	G383	173,276	39.8	Enterobacter phage PG7	Viruses; dsDNA viruses, no RNA stage; Caudovirales; Myoviridae.	1455074	Bacteria; Proteobacteria; Gammaproteobacteria; Enterobacteriales; Enterobacteriaceae; Enterobacter; Enterobacter cloacae complex
NC_028686	RefSeq	RVG	G383	166,313	39.6	Klebsiella phage JD18	Viruses; dsDNA viruses, no RNA stage; Caudovirales; Myoviridae.	1698360	-
NC_019909	RefSeq	RVG	G383	168,809	34.5	Yersinia phage phiR1-RT	Viruses; dsDNA viruses, no RNA stage; Caudovirales; Myoviridae.	1206558	Bacteria; Proteobacteria; Gammaproteobacteria; Enterobacteriales; Enterobacteriaceae; Yersinia
NC_028820	RefSeq	RVG	G383	162,101	34.6	Yersinia phage vB_YenM_TG1	Viruses; dsDNA viruses, no RNA stage; Caudovirales; Myoviridae.	1589265	-
NC_028762	RefSeq	RVG	G383	161,989	31.1	Proteus phage vB_PmiM_Pm5461	Viruses; dsDNA viruses, no RNA stage; Caudovirales; Myoviridae; Tevenvirinae; T4likevirus.	1636250	-
NC_024121	RefSeq	RVG	G383	167,266	41.7	Serratia phage PS2	Viruses; dsDNA viruses, no RNA stage; Caudovirales; Myoviridae; Tevenvirinae; T4likevirus.	1481112	Bacteria; Proteobacteria; Gammaproteobacteria; Enterobacteriales; Enterobacteriaceae; Serratia
NC_014663	RefSeq	RVG	G383	169,947	40	Acinetobacter phage Acj9	Viruses; dsDNA viruses, no RNA stage; Caudovirales; Myoviridae; Tevenvirinae; T4likevirus.	760939	Bacteria; Proteobacteria; Gammaproteobacteria; Pseudomonadales; Moraxellaceae; Acinetobacter
NC_014661	RefSeq	RVG	G383	164,093	39	Acinetobacter phage Acj61	Viruses; dsDNA viruses, no RNA stage; Caudovirales; Myoviridae; Tevenvirinae; T4likevirus.	760732	Bacteria; Proteobacteria; Gammaproteobacteria; Pseudomonadales; Moraxellaceae; Acinetobacter
NC_018087	RefSeq	RVG	G383	166,687	34.4	Acinetobacter phage ZZ1	Viruses; dsDNA viruses, no RNA stage; Caudovirales; Myoviridae; Tevenvirinae; T4likevirus.	1049283	Bacteria; Proteobacteria; Gammaproteobacteria; Pseudomonadales; Moraxellaceae; Acinetobacter; Acinetobacter calcoaceticus/baumannii complex
NC_015250	RefSeq	RVG	G383	159,801	39.7	Acinetobacter phage 133	Viruses; dsDNA viruses, no RNA stage; Caudovirales; Myoviridae; Tevenvirinae; T4likevirus.	279006	Bacteria; Proteobacteria; Gammaproteobacteria; Pseudomonadales; Moraxellaceae; Acinetobacter
NC_014660	RefSeq	RVG	G383	167,716	36.4	Acinetobacter phage Ac42	Viruses; dsDNA viruses, no RNA stage; Caudovirales; Myoviridae; Tevenvirinae; T4likevirus.	762660	Bacteria; Proteobacteria; Gammaproteobacteria; Pseudomonadales; Moraxellaceae
NC_029013	RefSeq	RVG	G383	177,688	43.2	Citrobacter phage IME-CF2	Viruses; dsDNA viruses, no RNA stage; Caudovirales; Myoviridae; Tevenvirinae; T4likevirus.	1673887	-
NC_025414	RefSeq	RVG	G383	178,171	43.1	Citrobacter phage Miller	Viruses; dsDNA viruses, no RNA stage; Caudovirales; Myoviridae; Tevenvirinae; T4likevirus.	1527524	Bacteria; Proteobacteria; Gammaproteobacteria; Enterobacteriales; Enterobacteriaceae; Citrobacter
NC_007023	RefSeq	RVG	G383	180,500	43.2	Enterobacteria phage RB43	Viruses; dsDNA viruses, no RNA stage; Caudovirales; Myoviridae; Tevenvirinae; T4likevirus.	115991	Bacteria; Proteobacteria; Gammaproteobacteria; Enterobacteriales
NC_014467	RefSeq	RVG	G383	176,788	43.5	Enterobacteria phage RB16	Viruses; dsDNA viruses, no RNA stage; Caudovirales; Myoviridae; Tevenvirinae; T4likevirus.	329381	Bacteria; Proteobacteria; Gammaproteobacteria; Enterobacteriales; Enterobacteriaceae; Escherichia
NC_021344	RefSeq	RVG	G383	176,227	43.5	Escherichia phage Lw1	Viruses; dsDNA viruses, no RNA stage; Caudovirales; Myoviridae; Tevenvirinae; T4likevirus.	1307804	Bacteria; Proteobacteria; Gammaproteobacteria; Enterobacteriales; Enterobacteriaceae; Escherichia
NC_019398	RefSeq	RVG	G383	178,193	44.5	Cronobacter phage vB_CsaM_GAP161	Viruses; dsDNA viruses, no RNA stage; Caudovirales; Myoviridae; Tevenvirinae; T4likevirus.	1141138	Bacteria; Proteobacteria; Gammaproteobacteria; Enterobacteriales; Enterobacteriaceae; Cronobacter

NC_028755	RefSeq	RVG	G383	178,182	44.9	Citrobacter phage Margaery	Viruses; dsDNA viruses, no RNA stage; Caudovirales; Myoviridae; Tevenvirinae; T4likevirus.	1701810	-
NC_020080	RefSeq	RVG	G383	174,413	41.8	Klebsiella phage KP27	Viruses; dsDNA viruses, no RNA stage; Caudovirales; Myoviridae; Tevenvirinae; T4likevirus.	1129147	Bacteria; Proteobacteria; Gammaproteobacteria; Enterobacteriales; Enterobacteriaceae; Klebsiella
NC_028750	RefSeq	RVG	G383	176,081	41.8	Klebsiella phage Matisse	Viruses; dsDNA viruses, no RNA stage; Caudovirales; Myoviridae.	1675607	-
NC_014036	RefSeq	RVG	G383	174,436	41.8	Klebsiella phage KP15	Viruses; dsDNA viruses, no RNA stage; Caudovirales; Myoviridae; Tevenvirinae; T4likevirus.	707757	Bacteria; Proteobacteria; Gammaproteobacteria; Enterobacteriales; Enterobacteriaceae; Klebsiella
NC_009821	RefSeq	RVG	G383	164,270	40.5	Enterobacteria phage Phi1	Viruses; dsDNA viruses, no RNA stage; Caudovirales; Myoviridae; Tevenvirinae; T4likevirus.	448384	Bacteria; Proteobacteria; Gammaproteobacteria; Enterobacteriales; Enterobacteriaceae; Escherichia
NC_025425	RefSeq	RVG	G383	163,424	40.5	Enterobacteria phage GEC-3S	Viruses; dsDNA viruses, no RNA stage; Caudovirales; Myoviridae; Tevenvirinae; T4likevirus.	1222338	Bacteria; Proteobacteria; Gammaproteobacteria; Enterobacteriales; Enterobacteriaceae; Escherichia
NC_005066	RefSeq	RVG	G383	164,018	40.4	Enterobacteria phage RB49	Viruses; dsDNA viruses, no RNA stage; Caudovirales; Myoviridae; Tevenvirinae; T4likevirus.	50948	Bacteria; Proteobacteria; Gammaproteobacteria; Enterobacteriales; Enterobacteriaceae; Escherichia
NC_012740	RefSeq	RVG	G383	166,418	40.5	Enterobacteria phage JSE	Viruses; dsDNA viruses, no RNA stage; Caudovirales; Myoviridae; Tevenvirinae; T4likevirus.	576789	Bacteria; Proteobacteria; Gammaproteobacteria; Enterobacteriales; Enterobacteriaceae; Escherichia
NC_014635	RefSeq	RVG	G383	163,875	41.3	Aeromonas phage phiAS4	Viruses; dsDNA viruses, no RNA stage; Caudovirales; Myoviridae; Tevenvirinae; T4likevirus.	879628	Bacteria; Proteobacteria; Gammaproteobacteria; Aeromonadales; Aeromonadaceae; Aeromonas
NC_029000	RefSeq	RVG	G383	162,327	41.2	Stenotrophomonas phage IME13	Viruses; dsDNA viruses, no RNA stage; Caudovirales; Myoviridae; Tevenvirinae; T4likevirus.	1211280	-
NC_008208	RefSeq	RVG	G383	161,475	41	Aeromonas phage 25	Viruses; dsDNA viruses, no RNA stage; Caudovirales; Myoviridae; Tevenvirinae; T4likevirus.	233894	Bacteria; Proteobacteria; Gammaproteobacteria; Aeromonadales; Aeromonadaceae; Aeromonas
NC_020879	RefSeq	RVG	G383	161,978	41.3	Aeromonas phage Aes012	Viruses; dsDNA viruses, no RNA stage; Caudovirales; Myoviridae; Tevenvirinae; T4likevirus.	1198014	Bacteria; Proteobacteria; Gammaproteobacteria; Aeromonadales; Aeromonadaceae; Aeromonas
NC_019543	RefSeq	RVG	G383	160,646	41.2	Aeromonas phage Aes508	Viruses; dsDNA viruses, no RNA stage; Caudovirales; Myoviridae; Tevenvirinae; T4likevirus.	1198013	Bacteria; Proteobacteria; Gammaproteobacteria; Aeromonadales; Aeromonadaceae; Aeromonas
NC_005135	RefSeq	RVG	G383	173,591	43.9	Aeromonas phage 44RR2.8t	Viruses; dsDNA viruses, no RNA stage; Caudovirales; Myoviridae; Tevenvirinae; T4likevirus.	115987	Bacteria; Proteobacteria; Gammaproteobacteria; Aeromonadales; Aeromonadaceae; Aeromonas
NC_007022	RefSeq	RVG	G383	172,963	43.9	Aeromonas phage 31	Viruses; dsDNA viruses, no RNA stage; Caudovirales; Myoviridae; Tevenvirinae; T4likevirus.	321023	Bacteria; Proteobacteria; Gammaproteobacteria; Aeromonadales; Aeromonadaceae; Aeromonas
NC_005260	RefSeq	RVG	G383	233,234	42.8	Aeromonas phage Aeh1	Viruses; dsDNA viruses, no RNA stage; Caudovirales; Myoviridae; Tevenvirinae; T4likevirus.	227470	Bacteria; Proteobacteria; Gammaproteobacteria; Aeromonadales; Aeromonadaceae; Aeromonas
NC_023688	RefSeq	RVG	G383	222,006	42	Aeromonas phage PX29	Viruses; dsDNA viruses, no RNA stage; Caudovirales; Myoviridae; Tevenvirinae; T4likevirus.	926067	Bacteria; Proteobacteria; Gammaproteobacteria; Aeromonadales; Aeromonadaceae; Aeromonas
NC_014636	RefSeq	RVG	G383	225,268	43	Aeromonas phage phiAS5	Viruses; dsDNA viruses, no RNA stage; Caudovirales; Myoviridae; Tevenvirinae; T4likevirus.	879630	Bacteria; Proteobacteria; Gammaproteobacteria; Aeromonadales; Aeromonadaceae; Aeromonas
NC_028773	RefSeq	RVG	G383	182,145	40.2	Cronobacter phage S13	Viruses; dsDNA viruses, no RNA stage; Caudovirales; Myoviridae.	1327935	-
NC_015251	RefSeq	RVG	G383	235,229	37.2	Aeromonas phage 65	Viruses; dsDNA viruses, no RNA stage; Caudovirales; Myoviridae; Tevenvirinae; T4likevirus.	260149	Bacteria; Proteobacteria; Gammaproteobacteria; Aeromonadales; Aeromonadaceae; Aeromonas
NC_019538	RefSeq	RVG	G383	231,743	38.8	Aeromonas phage CC2	Viruses; dsDNA viruses, no RNA stage; Caudovirales; Myoviridae; Tevenvirinae; T4likevirus.	1204516	Bacteria; Proteobacteria; Gammaproteobacteria; Aeromonadales; Aeromonadaceae; Aeromonas
JN849462	EBI	RVG	G384	246,421	42.6	Vibriophage phi-pp2	Viruses; dsDNA viruses, no RNA stage; Caudovirales; Myoviridae; Tevenvirinae; Schizot4likevirus.	1204514	-
NC_005083	RefSeq	RVG	G384	244,834	42.6	Vibrio phage KVP40	Viruses; dsDNA viruses, no RNA stage; Caudovirales; Myoviridae; Tevenvirinae; Schizot4likevirus.	75320	Bacteria; Proteobacteria; Gammaproteobacteria; Vibrionales; Vibrionaceae; Vibrio; Vibrio harveyi group
NC_028829	RefSeq	RVG	G384	248,088	41.2	Vibrio phage ValKK3	Viruses; dsDNA viruses, no RNA stage; Caudovirales.	1610855	-
NC_023568	RefSeq	RVG	G384	246,964	41.3	Vibrio phage VH7D	Viruses; dsDNA viruses, no RNA stage; Caudovirales; Myoviridae; Tevenvirinae; Schizot4likevirus; unclassified Schizot4likevirus.	1262539	Bacteria; Proteobacteria; Gammaproteobacteria; Vibrionales; Vibrionaceae; Vibrio; Vibrio harveyi group
NC_021529	RefSeq	RVG	G384	247,511	41.3	Vibrio phage nt-1	Viruses; dsDNA viruses, no RNA stage; Caudovirales; Myoviridae; Tevenvirinae; Schizot4likevirus.	115992	Bacteria; Proteobacteria; Gammaproteobacteria; Vibrionales; Vibrionaceae; Vibrio; Vibrio harveyi group
NC_021073	RefSeq	RVG	G385	107,218	41	Vibrio phage henriette 12B8	Viruses; dsDNA viruses, no RNA stage; unclassified dsDNA phages.	573174	Bacteria; Proteobacteria; Gammaproteobacteria; Vibrionales; Vibrionaceae; Vibrio
KJ019026	EBI	RVG	G386	171,282	39.4	Synechococcus phage ACG-2014a	Viruses; dsDNA viruses, no RNA stage; Caudovirales; Myoviridae; Tevenvirinae; T4likevirus.	1493507	-
KJ019158	EBI	RVG	G386	171,292	39.4	Synechococcus phage ACG-2014a	Viruses; dsDNA viruses, no RNA stage; Caudovirales; Myoviridae; Tevenvirinae; T4likevirus.	1493507	-
KJ019137	EBI	RVG	G386	171,289	39.4	Synechococcus phage ACG-2014a	Viruses; dsDNA viruses, no RNA stage; Caudovirales; Myoviridae; Tevenvirinae; T4likevirus.	1493507	-
KJ019157	EBI	RVG	G386	171,292	39.4	Synechococcus phage ACG-2014a	Viruses; dsDNA viruses, no RNA stage; Caudovirales; Myoviridae; Tevenvirinae; T4likevirus.	1493507	-
KJ019122	EBI	RVG	G386	171,285	39.4	Synechococcus phage ACG-2014a	Viruses; dsDNA viruses, no RNA stage; Caudovirales; Myoviridae; Tevenvirinae; T4likevirus.	1493507	-
KJ019030	EBI	RVG	G386	171,279	39.4	Synechococcus phage ACG-2014a	Viruses; dsDNA viruses, no RNA stage; Caudovirales; Myoviridae; Tevenvirinae; T4likevirus.	1493507	-
KJ019076	EBI	RVG	G386	172,372	39.4	Synechococcus phage ACG-2014a	Viruses; dsDNA viruses, no RNA stage; Caudovirales; Myoviridae; Tevenvirinae; T4likevirus.	1493507	-
KJ019138	EBI	RVG	G386	171,295	39.4	Synechococcus phage ACG-2014a	Viruses; dsDNA viruses, no RNA stage; Caudovirales; Myoviridae; Tevenvirinae; T4likevirus.	1493507	-
KJ019039	EBI	RVG	G386	171,296	39.4	Synechococcus phage ACG-2014a	Viruses; dsDNA viruses, no RNA stage; Caudovirales; Myoviridae; Tevenvirinae; T4likevirus.	1493507	-
KJ019088	EBI	RVG	G386	171,276	39.4	Synechococcus phage ACG-2014a	Viruses; dsDNA viruses, no RNA stage; Caudovirales; Myoviridae; Tevenvirinae; T4likevirus.	1493507	-
KJ019055	EBI	RVG	G386	171,294	39.4	Synechococcus phage ACG-2014a	Viruses; dsDNA viruses, no RNA stage; Caudovirales; Myoviridae; Tevenvirinae; T4likevirus.	1493507	-
KJ019163	EBI	RVG	G386	171,179	39.4	Synechococcus phage ACG-2014a	Viruses; dsDNA viruses, no RNA stage; Caudovirales; Myoviridae; Tevenvirinae; T4likevirus.	1493507	-

KJ019153	EBI	RVG	G386	171,166	39.4	Synechococcus phage ACG-2014a	Viruses; dsDNA viruses, no RNA stage; Caudovirales; Myoviridae; Tevenvirinae; T4likevirus.	1493507	-
KJ019087	EBI	RVG	G386	171,238	39.4	Synechococcus phage ACG-2014a	Viruses; dsDNA viruses, no RNA stage; Caudovirales; Myoviridae; Tevenvirinae; T4likevirus.	1493507	-
KJ019116	EBI	RVG	G386	170,403	39.4	Synechococcus phage ACG-2014a	Viruses; dsDNA viruses, no RNA stage; Caudovirales; Myoviridae; Tevenvirinae; T4likevirus.	1493507	-
KJ019065	EBI	RVG	G386	171,179	39.4	Synechococcus phage ACG-2014a	Viruses; dsDNA viruses, no RNA stage; Caudovirales; Myoviridae; Tevenvirinae; T4likevirus.	1493507	-
KJ019084	EBI	RVG	G386	171,183	39.4	Synechococcus phage ACG-2014a	Viruses; dsDNA viruses, no RNA stage; Caudovirales; Myoviridae; Tevenvirinae; T4likevirus.	1493507	-
KJ019068	EBI	RVG	G386	171,231	39.4	Synechococcus phage ACG-2014a	Viruses; dsDNA viruses, no RNA stage; Caudovirales; Myoviridae; Tevenvirinae; T4likevirus.	1493507	-
KJ019033	EBI	RVG	G386	171,035	39.4	Synechococcus phage ACG-2014a	Viruses; dsDNA viruses, no RNA stage; Caudovirales; Myoviridae; Tevenvirinae; T4likevirus.	1493507	-
KJ019067	EBI	RVG	G386	171,192	39.4	Synechococcus phage ACG-2014a	Viruses; dsDNA viruses, no RNA stage; Caudovirales; Myoviridae; Tevenvirinae; T4likevirus.	1493507	-
KJ019114	EBI	RVG	G386	171,192	39.4	Synechococcus phage ACG-2014a	Viruses; dsDNA viruses, no RNA stage; Caudovirales; Myoviridae; Tevenvirinae; T4likevirus.	1493507	-
KJ019081	EBI	RVG	G386	171,192	39.4	Synechococcus phage ACG-2014a	Viruses; dsDNA viruses, no RNA stage; Caudovirales; Myoviridae; Tevenvirinae; T4likevirus.	1493507	-
KJ019135	EBI	RVG	G386	171,274	39.4	Synechococcus phage ACG-2014a	Viruses; dsDNA viruses, no RNA stage; Caudovirales; Myoviridae; Tevenvirinae; T4likevirus.	1493507	-
NC_023584	RefSeq	RVG	G386	170,438	39.4	Synechococcus phage S-MbCM100	Viruses; dsDNA viruses, no RNA stage; Caudovirales; Myoviridae; Tevenvirinae; T4likevirus.	1340812	Bacteria; Cyanobacteria; Oscillatoriophycideae; Chroococcales; Synechococcus
KJ019038	EBI	RVG	G386	171,073	39.4	Synechococcus phage ACG-2014a	Viruses; dsDNA viruses, no RNA stage; Caudovirales; Myoviridae; Tevenvirinae; T4likevirus.	1493507	-
NC_027130	RefSeq	RVG	G386	172,688	39.1	Synechococcus phage ACG-2014b	Viruses; dsDNA viruses, no RNA stage; Caudovirales; Myoviridae; Tevenvirinae; T4likevirus.	1493508	Bacteria; Cyanobacteria; Oscillatoriophycideae; Chroococcales; Synechococcus
NC_026924	RefSeq	RVG	G386	174,885	39.3	Synechococcus phage ACG-2014g	Viruses; dsDNA viruses, no RNA stage; Caudovirales; Myoviridae; Tevenvirinae; T4likevirus.	1493512	Bacteria; Cyanobacteria; Oscillatoriophycideae; Chroococcales; Synechococcus
NC_021530	RefSeq	RVG	G386	171,407	39.3	Synechococcus phage S-CAM8	Viruses; dsDNA viruses, no RNA stage; Caudovirales; Myoviridae.	754038	Bacteria; Cyanobacteria; Oscillatoriophycideae; Chroococcales; Synechococcus
HQ317290	EBI	RVG	G386	175,430	42.2	Synechococcus phage S-RIM2 R21_2007	Viruses; dsDNA viruses, no RNA stage; Caudovirales; Myoviridae.	869661	-
HQ317291	EBI	RVG	G386	175,419	42.2	Synechococcus phage S-RIM2 R9_2006	Viruses; dsDNA viruses, no RNA stage; Caudovirales; Myoviridae.	869663	-
NC_020859	RefSeq	RVG	G386	175,430	42.2	Synechococcus phage S-RIM2 R1_1999	Viruses; dsDNA viruses, no RNA stage; Caudovirales; Myoviridae.	869662	Bacteria; Cyanobacteria; Oscillatoriophycideae; Chroococcales; Synechococcus
NC_015285	RefSeq	RVG	G386	174,285	39.6	Prochlorococcus phage Syn33	Viruses; dsDNA viruses, no RNA stage; Caudovirales.	444878	Bacteria; Cyanobacteria; Oscillatoriophycideae; Chroococcales; Synechococcus
KF156339	EBI	RVG	G386	176,044	39.1	Synechococcus phage S-MbCM25	Viruses; dsDNA viruses, no RNA stage; Caudovirales; Myoviridae; Tevenvirinae; T4likevirus.	1340811	-
NC_019444	RefSeq	RVG	G386	176,043	39.1	Synechococcus phage ACG-2014c	Viruses; dsDNA viruses, no RNA stage; Caudovirales; Myoviridae; Tevenvirinae; T4likevirus.	1079998	Bacteria; Cyanobacteria; Oscillatoriophycideae; Chroococcales; Synechococcus
HQ317385	EBI	RVG	G386	168,327	40.6	Synechococcus phage S-RIM8 A.HR5	Viruses; dsDNA viruses, no RNA stage; Caudovirales; Myoviridae.	869726	-
NC_020486	RefSeq	RVG	G386	171,211	40.6	Synechococcus phage S-RIM8 A.HR1	Viruses; dsDNA viruses, no RNA stage; Caudovirales; Myoviridae.	869724	Bacteria; Cyanobacteria; Oscillatoriophycideae; Chroococcales; Synechococcus
JF974289	EBI	RVG	G386	171,211	40.6	Synechococcus phage S-RIM8 A.HR3	Viruses; dsDNA viruses, no RNA stage; Caudovirales; Myoviridae.	869725	-
NC_021072	RefSeq	RVG	G386	178,807	39.9	Cyanophage Syn30	Viruses; dsDNA viruses, no RNA stage; unclassified dsDNA phages.	536474	Bacteria; Cyanobacteria; Oscillatoriophycideae; Chroococcales; Synechococcus
NC_015282	RefSeq	RVG	G386	174,079	41.1	Synechococcus phage S-SM1	Viruses; dsDNA viruses, no RNA stage; Caudovirales; Myoviridae.	444859	Bacteria; Cyanobacteria; Oscillatoriophycideae; Chroococcales; Synechococcus
NC_021536	RefSeq	RVG	G386	171,797	40.6	Synechococcus phage S-IOM18	Viruses; dsDNA viruses, no RNA stage; Caudovirales; Myoviridae.	754039	Bacteria; Cyanobacteria; Oscillatoriophycideae; Chroococcales; Synechococcus
NC_015286	RefSeq	RVG	G386	175,230	41.3	Synechococcus phage Syn19	Viruses; dsDNA viruses, no RNA stage; Caudovirales; Myoviridae.	445684	Bacteria; Cyanobacteria; Oscillatoriophycideae; Chroococcales; Synechococcus
NC_015289	RefSeq	RVG	G386	176,184	40	Synechococcus phage S-SSM5	Viruses; dsDNA viruses, no RNA stage; Caudovirales; Myoviridae.	445685	Bacteria; Cyanobacteria; Oscillatoriophycideae; Chroococcales; Synechococcus
NC_015283	RefSeq	RVG	G386	176,428	37.6	Prochlorococcus phage P-RSM4	Viruses; dsDNA viruses, no RNA stage; Caudovirales; Myoviridae.	444862	Bacteria; Cyanobacteria; Prochlorales; Prochlorococcaceae; Prochlorococcus
NC_008296	RefSeq	RVG	G386	177,300	40.6	Synechococcus phage syn9	Viruses; dsDNA viruses, no RNA stage; Caudovirales; Myoviridae; Tevenvirinae; T4likevirus.	382359	Bacteria; Cyanobacteria; Oscillatoriophycideae; Chroococcales; Synechococcus
NC_021559	RefSeq	RVG	G386	179,063	36.7	Prochlorococcus phage P-SSM3	Viruses; dsDNA viruses, no RNA stage; Caudovirales; Myoviridae.	536453	Bacteria; Cyanobacteria; Prochlorales; Prochlorococcaceae; Prochlorococcus
NC_006884	RefSeq	RVG	G386	178,249	36.7	Prochlorococcus phage P-SSM4	Viruses; dsDNA viruses, no RNA stage; Caudovirales; Myoviridae; Tevenvirinae; T4likevirus.	268747	Bacteria; Cyanobacteria; Prochlorales; Prochlorococcaceae; Prochlorococcus
NC_021071	RefSeq	RVG	G386	177,211	40.2	Cyanophage P-RSM1	Viruses; dsDNA viruses, no RNA stage; Caudovirales; Myoviridae.	536444	Bacteria; Cyanobacteria; Prochlorales; Prochlorococcaceae; Prochlorococcus
NC_019443	RefSeq	RVG	G386	172,879	39.8	Synechococcus phage metaG-MbCM1	Viruses; dsDNA viruses, no RNA stage; Caudovirales; Myoviridae; Tevenvirinae; T4likevirus.	1079999	Bacteria; Cyanobacteria; Oscillatoriophycideae; Chroococcales
NC_015290	RefSeq	RVG	G386	182,180	37.1	Prochlorococcus phage P-SSM7	Viruses; dsDNA viruses, no RNA stage; Caudovirales; Myoviridae.	445688	Bacteria; Cyanobacteria; Prochlorales; Prochlorococcaceae; Prochlorococcus
NC_020875	RefSeq	RVG	G386	182,801	39.4	Synechococcus phage S-SSM4	Viruses; dsDNA viruses, no RNA stage; Caudovirales; Myoviridae.	536466	Bacteria; Cyanobacteria; Oscillatoriophycideae; Chroococcales; Synechococcus
NC_026923	RefSeq	RVG	G386	179,110	40.3	Synechococcus phage ACG-2014d	Viruses; dsDNA viruses, no RNA stage; Caudovirales; Myoviridae; Tevenvirinae; T4likevirus.	1493509	Bacteria; Cyanobacteria; Oscillatoriophycideae; Chroococcales; Synechococcus
NC_015281	RefSeq	RVG	G386	179,563	41.1	Synechococcus phage S-ShM2	Viruses; dsDNA viruses, no RNA stage; Caudovirales; Myoviridae.	445683	Bacteria; Cyanobacteria; Oscillatoriophycideae; Chroococcales; Synechococcus
NC_020855	RefSeq	RVG	G386	192,497	39.3	Cyanophage P-RSM6	Viruses; dsDNA viruses, no RNA stage; Caudovirales; Myoviridae.	929832	Bacteria; Cyanobacteria; Prochlorales; Prochlorococcaceae; Prochlorococcus
OBV_N00003	OBV	EVG	G386	187,437	46.2	-	-	-	-
NC_028663	RefSeq	RVG	G386	188,632	40.7	Cyanophage P-TIM40	Viruses; dsDNA viruses, no RNA stage; Caudovirales; Myoviridae.	1589733	-
HQ633061	CAMERA	RVG	G386	180,892	39.6	Synechococcus phage S-CBM2	Viruses; dsDNA viruses, no RNA stage; Caudovirales; Myoviridae.	351708	-
NC_020845	RefSeq	RVG	G386	180,977	37.8	Prochlorococcus phage MED4-213	Viruses; dsDNA viruses, no RNA stage; Caudovirales; Myoviridae.	889956	Bacteria; Cyanobacteria; Prochlorales; Prochlorococcaceae
NC_015280	RefSeq	RVG	G386	181,044	37.8	Prochlorococcus phage P-HM1	Viruses; dsDNA viruses, no RNA stage; Caudovirales; Myoviridae.	445700	Bacteria; Cyanobacteria; Prochlorales; Prochlorococcaceae; Prochlorococcus; Prochlorococcus marinus
NC_015284	RefSeq	RVG	G386	183,806	38.1	Prochlorococcus phage P-HM2	Viruses; dsDNA viruses, no RNA stage; Caudovirales; Myoviridae.	445696	Bacteria; Cyanobacteria; Prochlorales; Prochlorococcaceae; Prochlorococcus; Prochlorococcus marinus
NC_027132	RefSeq	RVG	G386	190,768	39	Synechococcus phage ACG-2014i	Viruses; dsDNA viruses, no RNA stage; Caudovirales; Myoviridae; Tevenvirinae; T4likevirus.	1493513	Bacteria; Cyanobacteria; Oscillatoriophycideae; Chroococcales; Synechococcus
NC_026928	RefSeq	RVG	G386	189,418	38.9	Synechococcus phage ACG-2014e	Viruses; dsDNA viruses, no RNA stage; Caudovirales; Myoviridae; Tevenvirinae; T4likevirus.	1493510	Bacteria; Cyanobacteria; Oscillatoriophycideae; Chroococcales; Synechococcus

NC_026926	RefSeq	RVG	G386	192,108	38.6	Synechococcus phage ACG-2014j	Viruses; dsDNA viruses, no RNA stage; Caudovirales; Myoviridae; Tevenvirinae; T4likevirus.	1493514	Bacteria; Cyanobacteria; Oscillatoriophycideae; Chroococcales; Synechococcus
NC_023587	RefSeq	RVG	G386	189,311	40.5	Synechococcus phage ACG-2014h	Viruses; dsDNA viruses, no RNA stage; Caudovirales; Myoviridae; Tevenvirinae; T4likevirus.	1340810	Bacteria; Cyanobacteria; Oscillatoriophycideae; Chroococcales; Synechococcus
NC_013085	RefSeq	RVG	G386	194,454	41.1	Synechococcus phage S-RSM4	Viruses; dsDNA viruses, no RNA stage; Caudovirales; Myoviridae; Tevenvirinae; T4likevirus.	555387	Bacteria; Cyanobacteria; Oscillatoriophycideae; Chroococcales; Synechococcus
NC_006820	RefSeq	RVG	G386	196,280	37.8	Synechococcus phage S-PM2	Viruses; dsDNA viruses, no RNA stage; Caudovirales; Myoviridae; Tevenvirinae; T4likevirus.	238854	Bacteria; Cyanobacteria; Oscillatoriophycideae; Chroococcales; Synechococcus
NC_015288	RefSeq	RVG	G386	191,195	40.6	Prochlorococcus phage Syn1	Viruses; dsDNA viruses, no RNA stage; Caudovirales.	444861	Bacteria; Cyanobacteria; Oscillatoriophycideae; Chroococcales; Synechococcus
NC_020837	RefSeq	RVG	G386	198,013	43	Synechococcus phage S-CAM1	Viruses; dsDNA viruses, no RNA stage; Caudovirales; Myoviridae.	754037	Bacteria; Cyanobacteria; Oscillatoriophycideae; Chroococcales; Synechococcus
NC_020851	RefSeq	RVG	G386	208,007	36	Synechococcus phage S-SKS1	Viruses; dsDNA viruses, no RNA stage; Caudovirales; Siphoviridae.	754042	Bacteria; Cyanobacteria; Oscillatoriophycideae; Chroococcales; Synechococcus
NC_006883	RefSeq	RVG	G386	252,401	35.5	Prochlorococcus phage P-SSM2	Viruses; dsDNA viruses, no RNA stage; Caudovirales; Myoviridae; Tevenvirinae; T4likevirus.	268746	Bacteria; Cyanobacteria; Prochlorales; Prochlorococcaceae; Prochlorococcus
OBV_N00002	OBV	EVG	G386	191,793	41.8	-	-	-	-
NC_015279	RefSeq	RVG	G386	190,789	40.4	Synechococcus phage S-SM2	Viruses; dsDNA viruses, no RNA stage; Caudovirales; Myoviridae.	444860	Bacteria; Cyanobacteria; Oscillatoriophycideae; Chroococcales; Synechococcus
NC_028955	RefSeq	RVG	G386	197,361	34.3	Prochlorococcus phage P-TIM68	Viruses; dsDNA viruses, no RNA stage; Caudovirales; Myoviridae.	1542477	-
NC_015287	RefSeq	RVG	G386	232,878	39.1	Synechococcus phage S-SSM7	Viruses; dsDNA viruses, no RNA stage; Caudovirales; Myoviridae.	445686	Bacteria; Cyanobacteria; Oscillatoriophycideae; Chroococcales; Synechococcus
NC_026927	RefSeq	RVG	G386	228,143	41.6	Synechococcus phage ACG-2014f	Viruses; dsDNA viruses, no RNA stage; Caudovirales; Myoviridae; Tevenvirinae; T4likevirus.	1493511	Bacteria; Cyanobacteria; Oscillatoriophycideae; Chroococcales; Synechococcus
NC_015569	RefSeq	RVG	G387	178,563	39.7	Synechococcus phage S-CRM01	Viruses; dsDNA viruses, no RNA stage; Caudovirales; Myoviridae.	1026955	Bacteria; Cyanobacteria; Oscillatoriophycideae; Chroococcales; Synechococcus
NC_020484	RefSeq	RVG	G388	147,284	33.4	Pelagibacter phage HTVC008M	Viruses; dsDNA viruses, no RNA stage; Caudovirales; Myoviridae.	1283076	Bacteria; Proteobacteria; Alphaproteobacteria; Pelagibacterales; Pelagibacteraceae; Candidatus Pelagibacter
TARA_ERS489603_N000003	TOV	EVG	G388	136,515	32.9	-	-	-	-
TARA_ERS490452_N000022	TOV	EVG	G388	142,921	41.5	-	-	-	-
TARA_ERS488499_N000029	TOV	EVG	G388	143,743	32.9	-	-	-	-
TARA_ERS488448_N000015	TOV	EVG	G388	143,922	32.9	-	-	-	-
TARA_ERS478007_N000001	TOV	EVG	G388	143,922	32.9	-	-	-	-
TARA_ERS488448_N000021	TOV	EVG	G389	124,678	38	-	-	-	-
TARA_ERS478052_N000010	TOV	EVG	G389	166,838	37.4	-	-	-	-
TARA_ERS488813_N000021	TOV	EVG	G390	127,820	32.6	-	-	-	-
TARA_ERS488673_N000052	TOV	EVG	G390	158,573	36.2	-	-	-	-
TARA_ERS490953_N000001	TOV	EVG	G390	207,341	34.7	-	-	-	-
OBV_N00006	OBV	EVG	G390	143,397	39.9	-	-	-	-
OBV_N00005	OBV	EVG	G390	148,347	41.1	-	-	-	-
NC_028945	RefSeq	RVG	G391	206,713	49.1	Sinorhizobium phage phiN3	Viruses; dsDNA viruses, no RNA stage; Caudovirales; Myoviridae; Tevenvirinae; T4likevirus.	1647405	-
NC_027204	RefSeq	RVG	G391	194,701	49	Sinorhizobium phage phiM12	Viruses; dsDNA viruses, no RNA stage; Caudovirales; Myoviridae; Tevenvirinae; T4likevirus.	1357423	Bacteria; Proteobacteria; Alphaproteobacteria; Rhizobiales; Rhizobiaceae; Sinorhizobium
NC_025422	RefSeq	RVG	G392	155,997	37.8	Caulobacter phage Cr30	Viruses; dsDNA viruses, no RNA stage; Caudovirales; Myoviridae.	1357714	Bacteria; Proteobacteria; Alphaproteobacteria; Caulobacterales; Caulobacteraceae; Caulobacter
NC_016570	RefSeq	RVG	G393	157,304	44.5	Escherichia phage Cba120	Viruses; dsDNA viruses, no RNA stage; Caudovirales; Myoviridae.	1077152	Bacteria; Proteobacteria; Gammaproteobacteria; Enterobacteriales; Enterobacteriaceae; Escherichia
NC_016073	RefSeq	RVG	G393	157,950	44.5	Salmonella phage SFP10	Viruses; dsDNA viruses, no RNA stage; Caudovirales; Myoviridae.	1080800	Bacteria; Proteobacteria; Gammaproteobacteria; Enterobacteriales; Enterobacteriaceae; Escherichia
NC_019452	RefSeq	RVG	G393	156,628	44.5	Escherichia phage PhaxI	Viruses; dsDNA viruses, no RNA stage; Caudovirales; Myoviridae.	926589	Bacteria; Proteobacteria; Gammaproteobacteria; Enterobacteriales; Enterobacteriaceae; Escherichia
NC_024122	RefSeq	RVG	G393	162,910	44.4	Salmonella phage vB_SalM_SJ3	Viruses; dsDNA viruses, no RNA stage; Caudovirales; Myoviridae.	1446492	Bacteria; Proteobacteria; Gammaproteobacteria; Enterobacteriales; Enterobacteriaceae
NC_019530	RefSeq	RVG	G393	157,785	44.7	Salmonella phage PhiSH19	Viruses; dsDNA viruses, no RNA stage; Caudovirales; Myoviridae.	1108865	Bacteria; Proteobacteria; Gammaproteobacteria; Enterobacteriales; Enterobacteriaceae; Salmonella; Salmonella enterica
NC_029042	RefSeq	RVG	G393	156,833	44.6	Salmonella phage 38	Viruses; dsDNA viruses, no RNA stage; Caudovirales; Myoviridae.	1654891	-
NC_027119	RefSeq	RVG	G393	157,498	44.6	Salmonella phage Det7	Viruses; dsDNA viruses, no RNA stage; Caudovirales; Myoviridae.	454798	Bacteria; Proteobacteria; Gammaproteobacteria; Enterobacteriales; Enterobacteriaceae; Salmonella; Salmonella enterica
NC_015296	RefSeq	RVG	G393	157,061	45.2	Salmonella phage ViI	Viruses; dsDNA viruses, no RNA stage; Caudovirales; Myoviridae.	867696	Bacteria; Proteobacteria; Gammaproteobacteria; Enterobacteriales; Enterobacteriaceae; Salmonella; Salmonella enterica
NC_025446	RefSeq	RVG	G393	157,308	45	Escherichia phage ECML-4	Viruses; dsDNA viruses, no RNA stage; Caudovirales; Myoviridae.	1204523	Bacteria; Proteobacteria; Gammaproteobacteria; Enterobacteriales; Enterobacteriaceae; Escherichia
NC_023856	RefSeq	RVG	G393	152,460	44.9	Salmonella phage vB_SalM_SJ2	Viruses; dsDNA viruses, no RNA stage; Caudovirales; Myoviridae.	1458849	Bacteria; Proteobacteria; Gammaproteobacteria; Enterobacteriales; Enterobacteriaceae; Salmonella; Salmonella enterica
NC_022772	RefSeq	RVG	G393	156,338	45.6	Salmonella phage Marshall	Viruses; dsDNA viruses, no RNA stage; Caudovirales; Myoviridae.	1406794	Bacteria; Proteobacteria; Gammaproteobacteria; Enterobacteriales; Enterobacteriaceae; Salmonella; Salmonella enterica
NC_022768	RefSeq	RVG	G393	154,701	45.6	Salmonella phage Maynard	Viruses; dsDNA viruses, no RNA stage; Caudovirales; Myoviridae.	1406795	Bacteria; Proteobacteria; Gammaproteobacteria; Enterobacteriales; Enterobacteriaceae; Salmonella; Salmonella enterica
NC_019910	RefSeq	RVG	G393	159,624	50.2	Salmonella phage SKML-39	Viruses; dsDNA viruses, no RNA stage; Caudovirales; Myoviridae.	1204528	Bacteria; Proteobacteria; Gammaproteobacteria; Enterobacteriales; Enterobacteriaceae
NC_013693	RefSeq	RVG	G393	158,006	50.4	Shigella phage Ag3	Viruses; dsDNA viruses, no RNA stage; Caudovirales; Myoviridae.	637730	Bacteria; Proteobacteria; Gammaproteobacteria; Enterobacteriales; Enterobacteriaceae; Shigella
NC_019925	RefSeq	RVG	G393	152,427	49.3	Dickeya phage Limestone	Viruses; dsDNA viruses, no RNA stage; Caudovirales; Myoviridae.	1091052	Bacteria; Proteobacteria; Gammaproteobacteria; Enterobacteriales; Enterobacteriaceae; Dickeya
NC_025452	RefSeq	RVG	G393	155,346	49.6	Dickeya phage RC-2014	Viruses; dsDNA viruses, no RNA stage; Caudovirales; Myoviridae.	1477406	Bacteria; Proteobacteria; Gammaproteobacteria; Enterobacteriales; Enterobacteriaceae
NC_022343	RefSeq	RVG	G393	159,991	46.7	Klebsiella phage 0507-KN2-1	Viruses; dsDNA viruses, no RNA stage; Caudovirales; Myoviridae.	1206545	Bacteria; Proteobacteria; Gammaproteobacteria; Enterobacteriales; Enterobacteriaceae; Klebsiella
NC_020083	RefSeq	RVG	G393	157,834	52	Serratia phage phiMAM1	Viruses; dsDNA viruses, no RNA stage; Caudovirales; Myoviridae.	1262513	Bacteria; Proteobacteria; Gammaproteobacteria; Enterobacteriales; Enterobacteriaceae; Serratia
NC_027340	RefSeq	RVG	G393	162,160	50.3	Erwinia phage phiEa2809	Viruses; dsDNA viruses, no RNA stage; Caudovirales; Myoviridae.	1564096	Bacteria; Proteobacteria; Gammaproteobacteria; Enterobacteriales; Enterobacteriaceae; Erwinia

NC_013697	RefSeq	RVG	G394	157,486	56.3	Deftia phage phiW-14	Viruses; dsDNA viruses, no RNA stage; Caudovirales; Myoviridae.	665032	Bacteria; Proteobacteria; Betaproteobacteria; Burkholderiales; Comamonadaceae; Delftia
NC_028676	RefSeq	RVG	G395	149,218	49.8	Sinorhizobium phage phiM9	Viruses; dsDNA viruses, no RNA stage; Caudovirales; Myoviridae.	1636182	-
NC_025429	RefSeq	RVG	G395	156,446	49.9	Rhizobium phage vB_RleM_P10VF	Viruses; dsDNA viruses, no RNA stage; Caudovirales; Myoviridae.	1527770	Bacteria; Proteobacteria; Alphaproteobacteria; Rhizobiales; Rhizobiaceae; Rhizobium
KF148616	EBI	RVG	G396	132,667	26	Campylobacter phage CP8	Viruses; dsDNA viruses, no RNA stage; Caudovirales; Myoviridae; Cp8unalikevirus.	1340809	-
NC_016562	RefSeq	RVG	G396	132,662	26	Campylobacter phage CPX	Viruses; dsDNA viruses, no RNA stage; Caudovirales; Myoviridae; Cp8unalikevirus.	1110702	Bacteria; Proteobacteria; delta/epsilon subdivisions; Epsilonproteobacteria; Campylobacterales; Campylobacteraceae; Campylobacter
FR823450	EBI	RVG	G396	132,454	26	Campylobacter phage CP81	Viruses; dsDNA viruses, no RNA stage; Caudovirales; Myoviridae; Cp8unalikevirus.	990550	-
NC_015464	RefSeq	RVG	G396	135,041	26.2	Campylobacter phage NCTC12673	Viruses; dsDNA viruses, no RNA stage; Caudovirales; Myoviridae; Cp8unalikevirus.	934027	Bacteria; Proteobacteria; delta/epsilon subdivisions; Epsilonproteobacteria; Campylobacterales; Campylobacteraceae; Campylobacter
NC_018861	RefSeq	RVG	G396	133,572	26.1	Campylobacter phage CP30A	Viruses; dsDNA viruses, no RNA stage; Caudovirales; Myoviridae.	1229752	Bacteria; Proteobacteria; delta/epsilon subdivisions; Epsilonproteobacteria; Campylobacterales; Campylobacteraceae; Campylobacter; Campylobacter jejuni
NC_027997	RefSeq	RVG	G397	177,534	27.4	Campylobacter phage CP220	Viruses; dsDNA viruses, no RNA stage; Caudovirales; Myoviridae; Cp220likevirus.	722417	Bacteria; Proteobacteria; delta/epsilon subdivisions; Epsilonproteobacteria; Campylobacterales; Campylobacteraceae; Campylobacter
NC_027996	RefSeq	RVG	G397	175,720	27.3	Campylobacter phage CPt10	Viruses; dsDNA viruses, no RNA stage; Caudovirales; Myoviridae; Cp220likevirus.	722418	Bacteria; Proteobacteria; delta/epsilon subdivisions; Epsilonproteobacteria; Campylobacterales; Campylobacteraceae
NC_019507	RefSeq	RVG	G397	182,833	27.2	Campylobacter phage CP21	Viruses; dsDNA viruses, no RNA stage; Caudovirales; Myoviridae; Cp220likevirus.	1190451	Bacteria; Proteobacteria; delta/epsilon subdivisions; Epsilonproteobacteria; Campylobacterales; Campylobacteraceae; Campylobacter
NC_027989	RefSeq	RVG	G397	53,237	27.3	Campylobacter phage IBB35	Viruses; dsDNA viruses, no RNA stage; Caudovirales; Myoviridae; Cp220likevirus.	1006972	Bacteria; Proteobacteria; delta/epsilon subdivisions; Epsilonproteobacteria; Campylobacterales; Campylobacteraceae; Campylobacter
TARA_ERS488589_N000003	TOV	EVG	G398	179,949	32	-	-	-	-
TARA_ERS490204_N000019	TOV	EVG	G399	211,173	42.9	-	-	-	-
NC_019526	RefSeq	RVG	G400	345,809	31.7	Enterobacteria phage vB_KleM-RaK2	Viruses; dsDNA viruses, no RNA stage; Caudovirales; Myoviridae.	1147094	Bacteria; Proteobacteria; Gammaproteobacteria; Enterobacteriales; Enterobacteriaceae; Klebsiella
NC_027399	RefSeq	RVG	G400	346,602	31.7	Klebsiella phage K64-1	Viruses; dsDNA viruses, no RNA stage; Caudovirales; Myoviridae.	1439894	Bacteria; Proteobacteria; Gammaproteobacteria; Enterobacteriales; Enterobacteriaceae; Klebsiella
NC_027364	RefSeq	RVG	G400	348,113	34.1	Escherichia phage PBECO 4	Viruses; dsDNA viruses, no RNA stage; Caudovirales; Myoviridae.	1273738	Bacteria; Proteobacteria; Gammaproteobacteria; Enterobacteriales; Enterobacteriaceae; Escherichia
NC_025447	RefSeq	RVG	G400	348,532	34.1	Escherichia phage 121Q	Viruses; dsDNA viruses, no RNA stage; Caudovirales; Myoviridae.	1555202	Bacteria; Proteobacteria; Gammaproteobacteria; Enterobacteriales; Enterobacteriaceae; Escherichia
NC_019401	RefSeq	RVG	G400	358,663	35.6	Cronobacter phage vB_CsaM_GAP32	Viruses; dsDNA viruses, no RNA stage; Caudovirales; Myoviridae.	1141136	Bacteria; Proteobacteria; Gammaproteobacteria; Enterobacteriales; Enterobacteriaceae; Cronobacter
NC_018088	RefSeq	RVG	G401	104,936	36.6	Colwellia phage 9A	Viruses; dsDNA viruses, no RNA stage; Caudovirales; Siphoviridae.	765765	Bacteria; Proteobacteria; Gammaproteobacteria; Alteromonadales; Colwelliaceae; Colwellia
NC_019516	RefSeq	RVG	G402	161,440	40.5	Cyanophage S-TIM5	Viruses; dsDNA viruses, no RNA stage; Caudovirales; Myoviridae.	1137745	Bacteria; Cyanobacteria; Oscillatoriophycideae; Chroococcales; Synechococcus
TARA_ERS488499_N000212	TOV	EVG	G403	40,097	36.5	-	-	-	-
TARA_ERS490320_N000001	TOV	EVG	G404	178,331	41.6	-	-	-	-
TARA_ERS478052_N000007	TOV	EVG	G404	180,244	38.4	-	-	-	-
TARA_ERS478052_N000008	TOV	EVG	G404	178,632	37.5	-	-	-	-
TARA_ERS488589_N000002	TOV	EVG	G404	191,499	45.8	-	-	-	-
TARA_ERS490142_N000041	TOV	EVG	G404	177,412	40	-	-	-	-
TARA_ERS488701_N000003	TOV	EVG	G404	177,963	40.7	-	-	-	-
TARA_ERS490346_N000037	TOV	EVG	G405	143,709	33.4	-	-	-	-
TARA_ERS488589_N000011	TOV	EVG	G406	106,054	42.4	-	-	-	-
TARA_ERS488558_N000072	TOV	EVG	G406	145,597	42.1	-	-	-	-
NC_004735	RefSeq	RVG	G407	129,908	42.5	Rhodothermus phage RM378	Viruses; dsDNA viruses, no RNA stage; Caudovirales; Myoviridae.	148943	Bacteria; Bacteroidetes/Chlorobi group; Bacteroidetes; Bacteroidetes Order II. Incertae sedis; Rhodothermaceae; Rhodothermus
TARA_ERS489148_N000004	TOV	EVG	G408	144,656	37.5	-	-	-	-
TARA_ERS490026_N000002	TOV	EVG	G409	149,021	40.9	-	-	-	-
TARA_ERS488929_N000011	TOV	EVG	G410	111,798	33.8	-	-	-	-
KP869110	EBI	RVG	G411	88,632	38.9	Escherichia coli O157 typing phage 12	Viruses; dsDNA viruses, no RNA stage; Caudovirales; Myoviridae.	1508674	-
KP869109	EBI	RVG	G411	88,771	38.9	Escherichia coli O157 typing phage 11	Viruses; dsDNA viruses, no RNA stage; Caudovirales; Myoviridae.	1508673	-
NC_012749	RefSeq	RVG	G411	88,487	38.9	Escherichia phage wV8	Viruses; dsDNA viruses, no RNA stage; Caudovirales; Myoviridae.	576791	Bacteria; Proteobacteria; Gammaproteobacteria; Enterobacteriales; Enterobacteriaceae; Escherichia
KP869100	EBI	RVG	G411	88,531	38.8	Escherichia coli O157 typing phage 1	Viruses; dsDNA viruses, no RNA stage; Caudovirales; Myoviridae.	1508671	-
NC_028872	RefSeq	RVG	G411	86,252	38.9	Escherichia phage HY02	Viruses; dsDNA viruses, no RNA stage; Caudovirales; Myoviridae.	1527531	-
NC_029023	RefSeq	RVG	G411	87,712	38.8	Escherichia phage JH2	Viruses; dsDNA viruses, no RNA stage; Caudovirales; Myoviridae.	1340750	-
NC_027360	RefSeq	RVG	G411	87,603	38.9	Enterobacteriaphage UAB_Phi87	Viruses; dsDNA viruses, no RNA stage; Caudovirales; Myoviridae.	1197935	Bacteria; Proteobacteria; Gammaproteobacteria; Enterobacteriales; Enterobacteriaceae; Salmonella; Salmonella enterica
NC_028825	RefSeq	RVG	G411	87,372	39	Escherichia phage vB_EcoM_AYO145A	Viruses; dsDNA viruses, no RNA stage; Caudovirales; Myoviridae; unclassified FelixO1likevirus.	1636202	-
NC_027337	RefSeq	RVG	G411	88,403	38.9	Escherichia phage vB_EcoM-VpaE1	Viruses; dsDNA viruses, no RNA stage; Caudovirales; Myoviridae.	1555238	Bacteria; Proteobacteria; Gammaproteobacteria; Enterobacteriales; Enterobacteriaceae; Escherichia
NC_027369	RefSeq	RVG	G411	86,231	38.9	Escherichia phage EC6	Viruses; dsDNA viruses, no RNA stage; Caudovirales; Myoviridae.	1229757	Bacteria; Proteobacteria; Gammaproteobacteria; Enterobacteriales; Enterobacteriaceae; Escherichia
JF461087	EBI	RVG	G411	83,331	38.9	Salmonella phage FO1a	Viruses; dsDNA viruses, no RNA stage; Caudovirales; Myoviridae.	1087480	-
NC_005282	RefSeq	RVG	G411	86,155	39	Salmonella phage FelixO1	Viruses; dsDNA viruses, no RNA stage; Caudovirales; Myoviridae.	77775	Bacteria; Proteobacteria; Gammaproteobacteria; Enterobacteriales; Enterobacteriaceae
KP143762	EBI	RVG	G411	87,709	39	Salmonella phage Mushroom	Viruses; dsDNA viruses, no RNA stage; Caudovirales; Myoviridae.	1572716	-
NC_027329	RefSeq	RVG	G411	87,510	38.8	Salmonella phage HB-2014	Viruses; dsDNA viruses, no RNA stage; Caudovirales; Myoviridae.	1567025	Bacteria; Proteobacteria; Gammaproteobacteria; Enterobacteriales; Enterobacteriaceae; Salmonella
NC_028935	RefSeq	RVG	G411	88,698	40.2	Escherichia phage phiSUSP2	Viruses; dsDNA viruses, no RNA stage; Caudovirales; Myoviridae.	1718669	-
NC_028808	RefSeq	RVG	G411	90,743	39.8	Escherichia phage phiSUSP1	Viruses; dsDNA viruses, no RNA stage; Caudovirales; Myoviridae.	1718606	-

NC_028247	RefSeq	RVG	G411	90,000	38.8	Citrobacter phage Michonne	Viruses; dsDNA viruses, no RNA stage; Caudovirales; Myoviridae.	1675603	-
NC_027293	RefSeq	RVG	G411	87,999	39	Citrobacter phage Moogle	Viruses; dsDNA viruses, no RNA stage; Caudovirales; Myoviridae.	1540094	Bacteria; Proteobacteria; Gammaproteobacteria; Enterobacteriales; Enterobacteriaceae; Citrobacter
NC_015292	RefSeq	RVG	G411	84,565	43.8	Erwinia phage phiEa104	Viruses; dsDNA viruses, no RNA stage; Caudovirales; Myoviridae.	925986	Bacteria; Proteobacteria; Gammaproteobacteria; Enterobacteriales; Enterobacteriaceae; Erwinia
NC_011811	RefSeq	RVG	G411	84,576	43.8	Erwinia phage phiEa21-4	Viruses; dsDNA viruses, no RNA stage; Caudovirales; Myoviridae.	557393	Bacteria; Proteobacteria; Gammaproteobacteria; Enterobacteriales; Enterobacteriaceae; Erwinia
HQ728263	EBI	RVG	G411	84,694	43.4	Erwinia phage vB_EamM-M7	Viruses; dsDNA viruses, no RNA stage; Caudovirales; Myoviridae.	1051674	-
KP869103	EBI	RVG	G412	137,973	43.6	Escherichia coli O157 typing phage 5	Viruses; dsDNA viruses, no RNA stage; Caudovirales; Myoviridae.	1508680	-
NC_011041	RefSeq	RVG	G412	137,947	43.6	Escherichia phage rv5	Viruses; dsDNA viruses, no RNA stage; Caudovirales; Myoviridae.	399183	Bacteria; Proteobacteria; Gammaproteobacteria; Enterobacteriales; Enterobacteriaceae; Escherichia
NC_022323	RefSeq	RVG	G412	136,910	43.6	Escherichia phage 2 JES-2013	Viruses; dsDNA viruses, no RNA stage; Caudovirales; Myoviridae.	1327956	Bacteria; Proteobacteria; Gammaproteobacteria; Enterobacteriales; Enterobacteriaceae; Escherichia
NC_028248	RefSeq	RVG	G412	136,896	43.6	Escherichia phage slur16	Viruses; dsDNA viruses, no RNA stage; Caudovirales; Myoviridae.	1720495	-
NC_024134	RefSeq	RVG	G412	139,020	43.6	Escherichia phage vB_EcoM_FFH2	Viruses; dsDNA viruses, no RNA stage; Caudovirales; Myoviridae.	1446490	Bacteria; Proteobacteria; Gammaproteobacteria; Enterobacteriales; Enterobacteriaceae; Escherichia
NC_019517	RefSeq	RVG	G412	136,947	43.7	Enterobacteria phage vB_EcoM-FV3	Viruses; dsDNA viruses, no RNA stage; Caudovirales; Myoviridae.	1131317	Bacteria; Proteobacteria; Gammaproteobacteria; Enterobacteriales; Enterobacteriaceae; Escherichia
NC_019400	RefSeq	RVG	G412	147,940	46.3	Cronobacter phage vB_CsaM_GAP31	Viruses; dsDNA viruses, no RNA stage; Caudovirales; Myoviridae.	1141135	Bacteria; Proteobacteria; Gammaproteobacteria; Enterobacteriales; Enterobacteriaceae; Cronobacter
NC_022968	RefSeq	RVG	G412	148,567	46.3	Enterobacteria phage 4MG	Viruses; dsDNA viruses, no RNA stage; Caudovirales; Myoviridae.	1391428	Bacteria; Proteobacteria; Gammaproteobacteria; Enterobacteriales; Enterobacteriaceae; Escherichia
NC_016071	RefSeq	RVG	G412	145,964	45.6	Salmonella phage PVP-SE1	Viruses; dsDNA viruses, no RNA stage; Caudovirales; Myoviridae.	889338	Bacteria; Proteobacteria; Gammaproteobacteria; Enterobacteriales; Enterobacteriaceae; Salmonella; Salmonella enterica
NC_027351	RefSeq	RVG	G412	147,745	45.3	Salmonella phage SSE-121	Viruses; dsDNA viruses, no RNA stage; Caudovirales; Myoviridae.	1204529	Bacteria; Proteobacteria; Gammaproteobacteria; Enterobacteriales; Enterobacteriaceae
NC_028659	RefSeq	RVG	G412	142,987	44.6	Klebsiella phage vB_KpnM_KB57	Viruses; dsDNA viruses, no RNA stage; Caudovirales; Myoviridae.	1719140	-
NC_025830	RefSeq	RVG	G412	120,938	40	Escherichia phage Av-05	Viruses; dsDNA viruses, no RNA stage; Caudovirales; Myoviridae.	1527519	Bacteria; Proteobacteria; Gammaproteobacteria; Enterobacteriales; Enterobacteriaceae; Escherichia
NC_017974	RefSeq	RVG	G413	149,273	50.9	Cronobacter phage CR3	Viruses; dsDNA viruses, no RNA stage; Caudovirales; Myoviridae.	1162295	Bacteria; Proteobacteria; Gammaproteobacteria; Enterobacteriales; Enterobacteriaceae; Cronobacter
NC_024354	RefSeq	RVG	G413	149,162	50.8	Cronobacter phage CR8	Viruses; dsDNA viruses, no RNA stage; Caudovirales; Myoviridae.	1327934	Bacteria; Proteobacteria; Gammaproteobacteria; Enterobacteriales; Enterobacteriaceae; Cronobacter
NC_028672	RefSeq	RVG	G413	149,732	50.7	Cronobacter phage PBES 02	Viruses; dsDNA viruses, no RNA stage; Caudovirales; Myoviridae.	1684115	-
NC_023717	RefSeq	RVG	G413	151,924	50.6	Cronobacter phage CR9	Viruses; dsDNA viruses, no RNA stage; Caudovirales; Myoviridae.	1162290	Bacteria; Proteobacteria; Gammaproteobacteria; Enterobacteriales; Enterobacteriaceae; Cronobacter
NC_020201	RefSeq	RVG	G413	142,349	50.1	Pectobacterium phage phiTE	Viruses; dsDNA viruses, no RNA stage; Caudovirales; Myoviridae.	1116482	Bacteria; Proteobacteria; Gammaproteobacteria; Enterobacteriales; Enterobacteriaceae; Pectobacterium
NC_029072	RefSeq	RVG	G414	94,766	45.6	Salmonella phage 19	Viruses; dsDNA viruses, no RNA stage; Caudovirales; Myoviridae.	1654883	-
NC_020079	RefSeq	RVG	G415	147,737	39.1	Escherichia phage phAPEC8	Viruses; dsDNA viruses, no RNA stage; Caudovirales; Myoviridae.	1229753	Bacteria; Proteobacteria; Gammaproteobacteria; Enterobacteriales; Enterobacteriaceae; Escherichia
NC_023693	RefSeq	RVG	G415	148,612	37.4	Enterobacteria phage phi92	Viruses; dsDNA viruses, no RNA stage; Caudovirales; Myoviridae.	948870	Bacteria; Proteobacteria; Gammaproteobacteria; Enterobacteriales; Enterobacteriaceae; Escherichia
HQ641350	EBI	RVG	G416	123,097	37.1	Vibrio phage ICP1_2006_B	Viruses; dsDNA viruses, no RNA stage; Caudovirales; Myoviridae.	979530	-
HQ641351	EBI	RVG	G416	123,104	37.1	Vibrio phage ICP1_2006_A	Viruses; dsDNA viruses, no RNA stage; Caudovirales; Myoviridae.	979529	-
HQ641353	EBI	RVG	G416	124,826	37.1	Vibrio phage ICP1_2001_A	Viruses; dsDNA viruses, no RNA stage; Caudovirales; Myoviridae.	979526	-
NC_015157	RefSeq	RVG	G416	125,956	37.1	Vibrio phage ICP1	Viruses; dsDNA viruses, no RNA stage; Caudovirales; Myoviridae.	979525	Bacteria; Proteobacteria; Gammaproteobacteria; Vibrionales; Vibrionaceae; Vibrio
HQ641352	EBI	RVG	G416	129,373	37.1	Vibrio phage ICP1_2005_A	Viruses; dsDNA viruses, no RNA stage; Caudovirales; Myoviridae.	979528	-
HQ641354	EBI	RVG	G416	128,083	37.2	Vibrio phage ICP1_2004_A	Viruses; dsDNA viruses, no RNA stage; Caudovirales; Myoviridae.	979527	-
HQ641348	EBI	RVG	G416	124,497	37.1	Vibrio phage ICP1_2006_D	Viruses; dsDNA viruses, no RNA stage; Caudovirales; Myoviridae.	979532	-
HQ641349	EBI	RVG	G416	124,497	37.1	Vibrio phage ICP1_2006_C	Viruses; dsDNA viruses, no RNA stage; Caudovirales; Myoviridae.	979531	-
NC_021067	RefSeq	RVG	G417	135,982	37.6	Vibrio phage helene 12B3	Viruses; dsDNA viruses, no RNA stage; unclassified dsDNA phages.	573173	Bacteria; Proteobacteria; Gammaproteobacteria; Vibrionales; Vibrionaceae; Vibrio
NC_029067	RefSeq	RVG	G417	138,234	38	Vibrio phage eugene 12A10	Viruses; dsDNA viruses, no RNA stage; unclassified dsDNA phages.	573172	-
NC_020843	RefSeq	RVG	G418	126,434	35.5	Vibrio phage 11895-B1	Viruses; dsDNA viruses, no RNA stage; Caudovirales; Myoviridae.	754075	Bacteria; Proteobacteria; Gammaproteobacteria; Vibrionales; Vibrionaceae; Vibrio
NC_020863	RefSeq	RVG	G419	129,155	35.5	Vibrio phage PWH3a-P1	Viruses; dsDNA viruses, no RNA stage; unclassified dsDNA phages.	754058	Bacteria; Proteobacteria; Gammaproteobacteria; Vibrionales; Vibrionaceae; Vibrio; Vibrio harveyi group
NC_029057	RefSeq	RVG	G419	134,742	35.3	Vibrio phage qdvp001	Viruses; dsDNA viruses, no RNA stage; unclassified dsDNA phages.	1003177	-
NC_025470	RefSeq	RVG	G420	139,004	36.9	Shewanella sp. phage 1/40	Viruses; dsDNA viruses, no RNA stage; Caudovirales; Myoviridae.	1458860	Bacteria; Proteobacteria; Gammaproteobacteria; Alteromonadales; Shewanellaceae; Shewanella
NC_025436	RefSeq	RVG	G420	133,824	36.9	Shewanella sp. phage 1/4	Viruses; dsDNA viruses, no RNA stage; Caudovirales; Myoviridae.	1458859	Bacteria; Proteobacteria; Gammaproteobacteria; Alteromonadales; Shewanellaceae; Shewanella
KF302034	EBI	RVG	G421	129,439	35.7	Pseudoalteromonas phage HM1	Viruses; unclassified phages.	1357705	-
NC_029094	RefSeq	RVG	G421	131,903	37.4	Pseudoalteromonas phage H101	Viruses; dsDNA viruses, no RNA stage; Caudovirales; Myoviridae.	1654919	-
TARA_ERS488613_N000031	TOV	EVG	G422	142,318	34.3	-	-	-	-
TARA_ERS489059_N000018	TOV	EVG	G423	93,537	36.1	-	-	-	-
LN610586	EBI	RVG	G424	93,053	49.3	Pseudomonas phage vB_PaeM_C2-10_Ab10	Viruses; dsDNA viruses, no RNA stage; Caudovirales; Myoviridae.	1548914	-
NC_019918	RefSeq	RVG	G424	92,777	49.3	Pseudomonas phage vB_PaeM_C2-10_Ab1	Viruses; dsDNA viruses, no RNA stage; Caudovirales; Myoviridae.	1231048	Bacteria; Proteobacteria; Gammaproteobacteria; Pseudomonadales; Pseudomonadaceae; Pseudomonas; Pseudomonas aeruginosa group
LN610575	EBI	RVG	G424	93,503	49.2	Pseudomonas phage vB_PaeM_C2-10_Ab08	Viruses; dsDNA viruses, no RNA stage; Caudovirales; Myoviridae.	1548903	-
LN610587	EBI	RVG	G424	93,308	49.3	Pseudomonas phage vB_PaeM_C2-10_Ab15	Viruses; dsDNA viruses, no RNA stage; Caudovirales; Myoviridae.	1548915	-
LN610572	EBI	RVG	G424	93,848	49.4	Pseudomonas phage vB_PaeM_C2-10_Ab02	Viruses; dsDNA viruses, no RNA stage; Caudovirales; Myoviridae.	1548900	-
NC_022986	RefSeq	RVG	G424	93,147	49.3	Pseudomonas phage PAK_P4	Viruses; dsDNA viruses, no RNA stage; Caudovirales; Myoviridae.	1327966	Bacteria; Proteobacteria; Gammaproteobacteria; Pseudomonadales; Pseudomonadaceae; Pseudomonas; Pseudomonas aeruginosa group
NC_022967	RefSeq	RVG	G424	92,495	49.3	Pseudomonas phage PAK_P2	Viruses; dsDNA viruses, no RNA stage; Caudovirales; Myoviridae.	1348912	Bacteria; Proteobacteria; Gammaproteobacteria; Pseudomonadales; Pseudomonadaceae; Pseudomonas; Pseudomonas aeruginosa group

NC_019450	RefSeq	RVG	G424	93,017	49.3	Pseudomonas phage JG004	Viruses; dsDNA viruses, no RNA stage; Caudovirales; Myoviridae.	757342	Bacteria; Proteobacteria; Gammaproteobacteria; Pseudomonadales; Pseudomonadaceae; Pseudomonas; Pseudomonas aeruginosa group
NC_028817	RefSeq	RVG	G424	93,879	49.4	Pseudomonas phage K8	Viruses; dsDNA viruses, no RNA stage; Caudovirales; Myoviridae.	1716041	-
NC_019913	RefSeq	RVG	G424	91,715	49.4	Pseudomonas phage PaP1	Viruses; dsDNA viruses, no RNA stage; Caudovirales; Myoviridae.	685892	Bacteria; Proteobacteria; Gammaproteobacteria; Pseudomonadales; Pseudomonadaceae; Pseudomonas; Pseudomonas aeruginosa group
NC_029083	RefSeq	RVG	G424	93,464	49.5	Pseudomonas phage PaoP5	Viruses; dsDNA viruses, no RNA stage; Caudovirales; Myoviridae.	1716042	-
NC_028652	RefSeq	RVG	G424	94,109	49.4	Pseudomonas phage C11	Viruses; dsDNA viruses, no RNA stage; Caudovirales; Myoviridae.	1735586	-
NC_015294	RefSeq	RVG	G424	93,198	49.5	Pseudomonas phage PAK_P1	Viruses; dsDNA viruses, no RNA stage; Caudovirales; Myoviridae.	743813	Bacteria; Proteobacteria; Gammaproteobacteria; Pseudomonadales; Pseudomonadaceae; Pseudomonas; Pseudomonas aeruginosa group
NC_029065	RefSeq	RVG	G424	98,765	48.5	Pseudomonas phage VCM	Viruses; dsDNA viruses, no RNA stage; Caudovirales; Myoviridae.	1729937	-
NC_023601	RefSeq	RVG	G424	97,906	47.7	Pseudomonas phage phiPsa374	Viruses; dsDNA viruses, no RNA stage; Caudovirales; Myoviridae.	1458843	Bacteria; Proteobacteria; Gammaproteobacteria; Pseudomonadales; Pseudomonadaceae; Pseudomonas; Pseudomonas syringae group; Pseudomonas syringae group genomosp. 1
NC_028882	RefSeq	RVG	G424	84,583	54.7	Pseudomonas phage vB_PaeM_PS24	Viruses; dsDNA viruses, no RNA stage; Caudovirales; Myoviridae.	1542092	-
NC_015272	RefSeq	RVG	G424	88,322	54.8	Pseudomonas phage KPP10	Viruses; dsDNA viruses, no RNA stage; Caudovirales; Myoviridae.	582345	Bacteria; Proteobacteria; Gammaproteobacteria; Pseudomonadales; Pseudomonadaceae; Pseudomonas; Pseudomonas aeruginosa group
NC_022974	RefSeq	RVG	G424	88,255	54.6	Pseudomonas phage CHA_P1	Viruses; dsDNA viruses, no RNA stage; Caudovirales; Myoviridae.	1327965	Bacteria; Proteobacteria; Gammaproteobacteria; Pseudomonadales; Pseudomonadaceae; Pseudomonas; Pseudomonas aeruginosa group
NC_022966	RefSeq	RVG	G424	88,135	54.7	Pseudomonas phage PAK_P5	Viruses; dsDNA viruses, no RNA stage; Caudovirales; Myoviridae.	1327964	Bacteria; Proteobacteria; Gammaproteobacteria; Pseudomonadales; Pseudomonadaceae; Pseudomonas; Pseudomonas aeruginosa group
KC862296	EBI	RVG	G424	88,097	54.8	Pseudomonas phage P3_CHA	Viruses; dsDNA viruses, no RNA stage; Caudovirales; Myoviridae.	981430	-
NC_022970	RefSeq	RVG	G424	88,097	54.8	Pseudomonas phage PAK_P3	Viruses; dsDNA viruses, no RNA stage; Caudovirales; Myoviridae.	981431	Bacteria; Proteobacteria; Gammaproteobacteria; Pseudomonadales; Pseudomonadaceae; Pseudomonas; Pseudomonas aeruginosa group
LN610581	EBI	RVG	G424	86,668	54.6	Pseudomonas phage vB_PaeM_PAO1_Ab04	Viruses; dsDNA viruses, no RNA stage; Caudovirales; Myoviridae.	1548909	-
NC_026587	RefSeq	RVG	G424	86,246	54.7	Pseudomonas phage vB_PaeM_PAO1_Ab03	Viruses; dsDNA viruses, no RNA stage; Caudovirales; Myoviridae.	1548901	Bacteria; Proteobacteria; Gammaproteobacteria; Pseudomonadales; Pseudomonadaceae; Pseudomonas; Pseudomonas aeruginosa group
LN610582	EBI	RVG	G424	84,759	54.6	Pseudomonas phage vB_PaeM_PAO1_Ab06	Viruses; dsDNA viruses, no RNA stage; Caudovirales; Myoviridae.	1548910	-
LN610576	EBI	RVG	G424	83,598	54.6	Pseudomonas phage vB_PaeM_PAO1_Ab17	Viruses; dsDNA viruses, no RNA stage; Caudovirales; Myoviridae.	1548904	-
LN610583	EBI	RVG	G424	85,783	54.5	Pseudomonas phage vB_PaeM_PAO1_Ab11	Viruses; dsDNA viruses, no RNA stage; Caudovirales; Myoviridae.	1548911	-
TARA_ERS489084_N000022	TOV	EVG	G425	86,671	40.4	-	-	-	-
TARA_ERS489113_N000080	TOV	EVG	G425	86,671	40.4	-	-	-	-
TARA_ERS489148_N000020	TOV	EVG	G425	86,671	40.4	-	-	-	-
TARA_ERS489059_N000023	TOV	EVG	G425	86,671	40.4	-	-	-	-
TARA_ERS489285_N000079	TOV	EVG	G426	83,840	41.2	-	-	-	-
TARA_ERS490953_N000011	TOV	EVG	G426	91,726	42.9	-	-	-	-
TARA_ERS490388_N000175	TOV	EVG	G427	72,817	40.2	-	-	-	-
NC_027332	RefSeq	RVG	G428	98,170	37	Acinetobacter phage YMC13/03/R2096	Viruses; dsDNA viruses, no RNA stage; Caudovirales; Myoviridae.	1560342	Bacteria; Proteobacteria; Gammaproteobacteria; Pseudomonadales; Moraxellaceae; Acinetobacter; Acinetobacter calcoaceticus/baumannii complex
NC_025462	RefSeq	RVG	G429	99,730	37.3	Acinetobacter phage vB_AbaM_Acibel004	Viruses; dsDNA viruses, no RNA stage; Caudovirales; Myoviridae.	1481186	Bacteria; Proteobacteria; Gammaproteobacteria; Pseudomonadales; Moraxellaceae; Acinetobacter; Acinetobacter calcoaceticus/baumannii complex
NC_024217	RefSeq	RVG	G430	82,438	41.3	Nitrincola phage 1M3-16	Viruses; dsDNA viruses, no RNA stage; Caudovirales.	1472912	Bacteria; Proteobacteria; Gammaproteobacteria; Oceanospirillales; Oceanospirillaceae
NC_010106	RefSeq	RVG	G431	52,975	44	Enterobacteria phage phiEcoM-GJ1	Viruses; dsDNA viruses, no RNA stage; Caudovirales; Myoviridae.	451705	Bacteria; Proteobacteria; Gammaproteobacteria; Enterobacteriales; Enterobacteriaceae; Escherichia
NC_023865	RefSeq	RVG	G431	55,098	44.9	Pectobacterium phage PM1	Viruses; dsDNA viruses, no RNA stage; Caudovirales; Myoviridae.	1399915	Bacteria; Proteobacteria; Gammaproteobacteria; Enterobacteriales; Enterobacteriaceae; Pectobacterium; Pectobacterium carotovorum
NC_019504	RefSeq	RVG	G431	56,621	44.2	Erwinia phage vB_EamM-Y2	Viruses; dsDNA viruses, no RNA stage; Caudovirales; Myoviridae.	1051676	Bacteria; Proteobacteria; Gammaproteobacteria; Enterobacteriales; Enterobacteriaceae; Erwinia
NC_025459	RefSeq	RVG	G432	53,744	52.8	Aeromonas phage pAh6-C	Viruses; dsDNA viruses, no RNA stage; Caudovirales; Myoviridae.	1505227	Bacteria; Proteobacteria; Gammaproteobacteria; Aeromonadales; Aeromonadaceae; Aeromonas
NC_023594	RefSeq	RVG	G432	54,789	49.4	Shewanella phage Spp001	Viruses; dsDNA viruses, no RNA stage; Caudovirales; Myoviridae.	1445859	Bacteria; Proteobacteria; Gammaproteobacteria; Alteromonadales; Shewanellaceae; Shewanella
NC_017969	RefSeq	RVG	G433	108,853	38.9	Escherichia phage Akfv33	Viruses; dsDNA viruses, no RNA stage; Caudovirales; Siphoviridae; T5likevirus.	1112008	Bacteria; Proteobacteria; Gammaproteobacteria; Enterobacteriales; Enterobacteriaceae; Escherichia
NC_024139	RefSeq	RVG	G433	108,483	39.2	Escherichia phage vB_EcoS_FFH1	Viruses; dsDNA viruses, no RNA stage; Caudovirales; Siphoviridae.	1446489	Bacteria; Proteobacteria; Gammaproteobacteria; Enterobacteriales; Enterobacteriaceae; Escherichia
NC_028840	RefSeq	RVG	G433	111,751	39	Escherichia phage slur09	Viruses; dsDNA viruses, no RNA stage; Caudovirales; Siphoviridae; T5likevirus; unclassified T5likevirus.	1728958	-
NC_015269	RefSeq	RVG	G433	118,351	39.4	Salmonella phage Spc35	Viruses; dsDNA viruses, no RNA stage; Caudovirales; Siphoviridae; T5likevirus.	977927	Bacteria; Proteobacteria; Gammaproteobacteria; Enterobacteriales; Enterobacteriaceae; Salmonella; Salmonella enterica
KM979355	EBI	RVG	G433	108,418	39.7	Enterobacteria phage DT571/2	Viruses; dsDNA viruses, no RNA stage; Caudovirales; Siphoviridae; T5likevirus; unclassified T5likevirus.	1567007	-
NC_027356	RefSeq	RVG	G433	108,065	39.7	Enterobacteria phage DT57C	Viruses; dsDNA viruses, no RNA stage; Caudovirales; Siphoviridae; T5likevirus; unclassified T5likevirus.	1567006	Bacteria; Proteobacteria; Gammaproteobacteria; Enterobacteriales; Enterobacteriaceae; Escherichia
NC_005859	RefSeq	RVG	G433	121,750	39.3	Escherichia phage T5	Viruses; dsDNA viruses, no RNA stage; Caudovirales; Siphoviridae; T5likevirus.	10726	Bacteria; Proteobacteria; Gammaproteobacteria; Enterobacteriales; Enterobacteriaceae; Escherichia
NC_028754	RefSeq	RVG	G433	120,098	38.8	Salmonella phage Shivani	Viruses; dsDNA viruses, no RNA stage; Caudovirales; Siphoviridae.	1572715	-
NC_010583	RefSeq	RVG	G433	111,382	39.9	Escherichia phage Eps7	Viruses; dsDNA viruses, no RNA stage; Caudovirales; Siphoviridae; T5likevirus.	491003	Bacteria; Proteobacteria; Gammaproteobacteria; Enterobacteriales; Enterobacteriaceae; Escherichia
NC_027297	RefSeq	RVG	G433	123,475	40.3	Salmonella phage Stitch	Viruses; dsDNA viruses, no RNA stage; Caudovirales; Siphoviridae; T5likevirus; unclassified T5likevirus.	1540099	Bacteria; Proteobacteria; Gammaproteobacteria; Enterobacteriales; Enterobacteriaceae; Salmonella; Salmonella enterica
NC_019919	RefSeq	RVG	G433	112,795	40.5	Yersinia phage phiR201	Viruses; dsDNA viruses, no RNA stage; Caudovirales; Siphoviridae.	1206557	Bacteria; Proteobacteria; Gammaproteobacteria; Enterobacteriales; Enterobacteriaceae; Yersinia
NC_018837	RefSeq	RVG	G433	122,024	40.6	Pectobacterium phage My1	Viruses; dsDNA viruses, no RNA stage; Caudovirales; Siphoviridae.	1204539	Bacteria; Proteobacteria; Gammaproteobacteria; Enterobacteriales; Enterobacteriaceae; Pectobacterium; Pectobacterium carotovorum

NC_019529	RefSeq	RVG	G433	111,506	39.7	Vibrio phage pVp-1	Viruses; dsDNA viruses, no RNA stage; Caudovirales; Siphoviridae; T5likevirus; unclassified T5likevirus.	1150989	Bacteria; Proteobacteria; Gammaproteobacteria; Vibrionales; Vibrionaceae; Vibrio; Vibrio harveyi group
NC_028895	RefSeq	RVG	G433	116,138	42.8	Vibrio phage phi 3	Viruses; dsDNA viruses, no RNA stage; Caudovirales; Siphoviridae; T5likevirus; unclassified T5likevirus.	1589298	-
TARA_ERS488813_N000032	TOV	EVG	G434	108,824	40	-	-	-	-
NC_016163	RefSeq	RVG	G435	262,391	32.5	Yersinia phage phiR1-37	Viruses; dsDNA viruses, no RNA stage; Caudovirales; Myoviridae.	331278	Bacteria; Proteobacteria; Gammaproteobacteria; Enterobacteriales; Enterobacteriaceae; Yersinia
NC_023718	RefSeq	RVG	G436	26,499	42.7	Pseudomonas phage phiPto-bp6g	Viruses; unclassified phages.	1080237	Bacteria; Proteobacteria; Gammaproteobacteria; Pseudomonadales; Pseudomonadaceae; Pseudomonas; Pseudomonas fluorescens group
NC_029050	RefSeq	RVG	G437	51,896	46.9	Salmonella phage 21	Viruses; dsDNA viruses, no RNA stage; Caudovirales; Myoviridae.	1654884	-
NC_024142	RefSeq	RVG	G438	72,583	42.9	Enterobacteria phage Bp4	Viruses; dsDNA viruses, no RNA stage; Caudovirales; Podoviridae; N4likevirus; unclassified N4likevirus.	1458848	Bacteria; Proteobacteria; Gammaproteobacteria; Enterobacteriales; Enterobacteriaceae; Escherichia
NC_018854	RefSeq	RVG	G438	69,855	42.7	Escherichia phage ECBP1	Viruses; dsDNA viruses, no RNA stage; Caudovirales; Podoviridae; N4likevirus; unclassified N4likevirus.	1604356	Bacteria; Proteobacteria; Gammaproteobacteria; Enterobacteriales; Enterobacteriaceae; Escherichia
KC206276	EBI	RVG	G438	70,912	42.9	Escherichia phage EC1-UPM	Viruses; dsDNA viruses, no RNA stage; Caudovirales; Podoviridae; N4likevirus; unclassified N4likevirus.	1258572	-
NC_023589	RefSeq	RVG	G438	71,629	42.7	Shigella phage pSb-1	Viruses; dsDNA viruses, no RNA stage; Caudovirales; Podoviridae.	1414738	Bacteria; Proteobacteria; Gammaproteobacteria; Enterobacteriales; Enterobacteriaceae; Shigella
NC_024790	RefSeq	RVG	G438	71,778	43.3	Escherichia phage vB_EcoP_PhAPEC7	Viruses; dsDNA viruses, no RNA stage; Caudovirales; Podoviridae; N4likevirus; unclassified N4likevirus.	1391223	Bacteria; Proteobacteria; Gammaproteobacteria; Enterobacteriales; Enterobacteriaceae; Escherichia
NC_024786	RefSeq	RVG	G438	71,248	43.5	Escherichia phage vB_EcoP_PhAPEC5	Viruses; dsDNA viruses, no RNA stage; Caudovirales; Podoviridae; N4likevirus; unclassified N4likevirus.	1395983	Bacteria; Proteobacteria; Gammaproteobacteria; Enterobacteriales; Enterobacteriaceae; Escherichia
NC_015933	RefSeq	RVG	G438	72,917	43.4	Escherichia phage vB_EcoP_G7C	Viruses; dsDNA viruses, no RNA stage; Caudovirales; Podoviridae; N4likevirus; unclassified N4likevirus.	1054461	Bacteria; Proteobacteria; Gammaproteobacteria; Enterobacteriales; Enterobacteriaceae; Escherichia
NC_019423	RefSeq	RVG	G438	72,570	43.1	Enterobacter phage IME11	Viruses; dsDNA viruses, no RNA stage; Caudovirales; Podoviridae; N4likevirus; unclassified N4likevirus.	1239384	Bacteria; Proteobacteria; Gammaproteobacteria; Enterobacteriales; Enterobacteriaceae; Escherichia
NC_008720	RefSeq	RVG	G438	70,153	41.3	Escherichia phage N4	Viruses; dsDNA viruses, no RNA stage; Caudovirales; Podoviridae; N4likevirus.	10752	Bacteria; Proteobacteria; Gammaproteobacteria; Enterobacteriales; Enterobacteriaceae; Escherichia
KF787094	EBI	RVG	G438	73,659	54.3	Achromobacter phage JWDelta	Viruses; dsDNA viruses, no RNA stage; Caudovirales; Podoviridae; N4likevirus; unclassified N4likevirus.	1416008	-
NC_023556	RefSeq	RVG	G438	72,329	54.4	Achromobacter phage JWAlpha	Viruses; dsDNA viruses, no RNA stage; Caudovirales; Podoviridae; N4likevirus; unclassified N4likevirus.	1416009	Bacteria; Proteobacteria; Betaproteobacteria; Burkholderiales; Alcaligenaceae; Achromobacter
NC_028908	RefSeq	RVG	G438	72,409	55.2	Achromobacter phage phiAxp-3	Viruses; dsDNA viruses, no RNA stage; Caudovirales; Podoviridae; N4likevirus; unclassified N4likevirus.	1664247	-
NC_023579	RefSeq	RVG	G438	75,568	47	Erwinia phage Ea9-2	Viruses; dsDNA viruses, no RNA stage; Caudovirales; Podoviridae; N4likevirus; unclassified N4likevirus.	1429767	Bacteria; Proteobacteria; Gammaproteobacteria; Enterobacteriales; Enterobacteriaceae; Erwinia
NC_027348	RefSeq	RVG	G438	73,990	59.9	Delftia phage RG-2014	Viruses; dsDNA viruses, no RNA stage; Caudovirales; Podoviridae.	1563661	Bacteria; Proteobacteria; Betaproteobacteria; Burkholderiales; Comamonadaceae; Delftia
NC_021782	RefSeq	RVG	G438	72,098	39.5	Salmonella phage FSL SP-076	Viruses; dsDNA viruses, no RNA stage; Caudovirales; Podoviridae; N4likevirus; unclassified N4likevirus.	1173762	Bacteria; Proteobacteria; Gammaproteobacteria; Enterobacteriales; Enterobacteriaceae; Salmonella; Salmonella enterica
NC_021772	RefSeq	RVG	G438	72,394	39.6	Salmonella phage FSL SP-058	Viruses; dsDNA viruses, no RNA stage; Caudovirales; Podoviridae; N4likevirus; unclassified N4likevirus.	1173761	Bacteria; Proteobacteria; Gammaproteobacteria; Enterobacteriales; Enterobacteriaceae; Salmonella; Salmonella enterica
NC_027381	RefSeq	RVG	G438	68,365	36	Escherichia phage Pollock	Viruses; dsDNA viruses, no RNA stage; Caudovirales; Podoviridae.	1540097	Bacteria; Proteobacteria; Gammaproteobacteria; Enterobacteriales; Enterobacteriaceae; Escherichia
NC_019485	RefSeq	RVG	G438	59,080	36.9	Enterobacter phage EcP1	Viruses; dsDNA viruses, no RNA stage; Caudovirales; Podoviridae; N4likevirus; unclassified N4likevirus.	942016	Bacteria; Proteobacteria; Gammaproteobacteria; Enterobacteriales; Enterobacteriaceae; Enterobacter; Enterobacter cloacae complex
NC_019514	RefSeq	RVG	G438	74,669	52.1	Erwinia phage vB_EamP-S6	Viruses; dsDNA viruses, no RNA stage; Caudovirales; Podoviridae; N4likevirus; unclassified N4likevirus.	1051675	Bacteria; Proteobacteria; Gammaproteobacteria; Enterobacteriales; Enterobacteriaceae; Erwinia
KJ803031	EBI	RVG	G439	74,806	49.2	Dinoroseobacter phage vBDshPR2C	Viruses; dsDNA viruses, no RNA stage; Caudovirales; Podoviridae; N4likevirus; unclassified N4likevirus.	1498169	-
NC_024367	RefSeq	RVG	G439	75,028	49.3	Dinoroseobacter phage DFL12phi1	Viruses; dsDNA viruses, no RNA stage; Caudovirales; Podoviridae; N4likevirus; unclassified N4likevirus.	1477404	Bacteria; Proteobacteria; Alphaproteobacteria; Rhodobacterales; Rhodobacteraceae; Dinoroseobacter
NC_012697	RefSeq	RVG	G439	74,611	47.9	Silicibacter phage DSS3phi2	Viruses; dsDNA viruses, no RNA stage; Caudovirales; Podoviridae; N4likevirus; unclassified N4likevirus.	490912	Bacteria; Proteobacteria; Alphaproteobacteria; Rhodobacterales; Rhodobacteraceae; Ruegeria
NC_012696	RefSeq	RVG	G439	73,325	47	Sulfitobacter phage EE36phi1	Viruses; dsDNA viruses, no RNA stage; Caudovirales; Podoviridae; N4likevirus; unclassified N4likevirus.	490913	Bacteria; Proteobacteria; Alphaproteobacteria; Rhodobacterales; Rhodobacteraceae; Sulfitobacter
NC_020862	RefSeq	RVG	G439	74,485	43	Sulfitobacter phage phiCB2047-B	Viruses; dsDNA viruses, no RNA stage; Caudovirales; Podoviridae; N4likevirus; unclassified N4likevirus.	754046	Bacteria; Proteobacteria; Alphaproteobacteria; Rhodobacterales; Rhodobacteraceae; Sulfitobacter
KM411958	EBI	RVG	G440	72,646	54.9	Pseudomonas phage RWG	Viruses; dsDNA viruses, no RNA stage; Caudovirales; Podoviridae; N4likevirus; unclassified N4likevirus.	1541890	-
NC_024140	RefSeq	RVG	G440	72,028	54.9	Pseudomonas phage vB_PaeP_C2-10_Ab09	Viruses; dsDNA viruses, no RNA stage; Caudovirales; Podoviridae; N4likevirus; unclassified N4likevirus.	1476391	Bacteria; Proteobacteria; Gammaproteobacteria; Pseudomonadales; Pseudomonadaceae; Pseudomonas; Pseudomonas aeruginosa group
KM411960	EBI	RVG	G440	73,048	54.9	Pseudomonas phage phi176	Viruses; dsDNA viruses, no RNA stage; Caudovirales; Podoviridae; N4likevirus; unclassified N4likevirus.	1541891	-
NC_027345	RefSeq	RVG	G440	73,008	54.9	Pseudomonas phage Pa2	Viruses; dsDNA viruses, no RNA stage; Caudovirales; Podoviridae; N4likevirus; unclassified N4likevirus.	1530400	Bacteria; Proteobacteria; Gammaproteobacteria; Pseudomonadales; Pseudomonadaceae; Pseudomonas; Pseudomonas aeruginosa group
NC_013692	RefSeq	RVG	G440	72,544	55	Pseudomonas phage LIT1	Viruses; dsDNA viruses, no RNA stage; Caudovirales; Podoviridae; N4likevirus; unclassified N4likevirus.	655098	Bacteria; Proteobacteria; Gammaproteobacteria; Pseudomonadales; Pseudomonadaceae; Pseudomonas; Pseudomonas aeruginosa group
NC_028885	RefSeq	RVG	G440	72,378	55	Pseudomonas phage DL64	Viruses; dsDNA viruses, no RNA stage; Caudovirales; Podoviridae.	1640973	-
JX194238	EBI	RVG	G440	72,321	54.8	Pseudomonas phage PA26	Viruses; dsDNA viruses, no RNA stage; Caudovirales; Podoviridae; N4likevirus; unclassified N4likevirus.	1204542	-
NC_029101	RefSeq	RVG	G440	72,192	54.9	Pseudomonas phage YH30	Viruses; dsDNA viruses, no RNA stage; Caudovirales; Podoviridae; N4likevirus; unclassified N4likevirus.	1636189	-

NC_027388	RefSeq	RVG	G440	73,050	54.9	Pseudomonas phage YH6	Viruses; dsDNA viruses, no RNA stage; Caudovirales; Podoviridae; N4likevirus; unclassified N4likevirus.	1566995	Bacteria; Proteobacteria; Gammaproteobacteria; Pseudomonadales; Pseudomonadaceae; Pseudomonas; Pseudomonas aeruginosa group
NC_013691	RefSeq	RVG	G440	74,901	53.2	Pseudomonas phage LUZ7	Viruses; dsDNA viruses, no RNA stage; Caudovirales; Podoviridae; N4likevirus; unclassified N4likevirus.	655097	Bacteria; Proteobacteria; Gammaproteobacteria; Pseudomonadales; Pseudomonadaceae; Pseudomonas; Pseudomonas aeruginosa group
NC_029017	RefSeq	RVG	G440	73,420	53.5	Pseudomonas phage KPP21	Viruses; dsDNA viruses, no RNA stage; Caudovirales; Podoviridae; N4likevirus; unclassified N4likevirus.	1678082	-
KC438283	EBI	RVG	G441	68,964	34.6	Vibrio phage VCO139	Viruses; dsDNA viruses, no RNA stage; Caudovirales; Podoviridae; N4likevirus; unclassified N4likevirus.	1283073	-
NC_021540	RefSeq	RVG	G441	69,278	34.6	Vibrio phage JA-1	Viruses; dsDNA viruses, no RNA stage; Caudovirales; Podoviridae; N4likevirus; unclassified N4likevirus.	1283071	Bacteria; Proteobacteria; Gammaproteobacteria; Vibrionales; Vibrionaceae; Vibrio
NC_028799	RefSeq	RVG	G441	66,708	34.5	Vibrio phage phi 1	Viruses; dsDNA viruses, no RNA stage; Caudovirales; Podoviridae.	1589297	-
NC_020868	RefSeq	RVG	G442	76,718	42.5	Vibrio phage VBP32	Viruses; dsDNA viruses, no RNA stage; Caudovirales; Podoviridae; N4likevirus; unclassified N4likevirus.	754072	Bacteria; Proteobacteria; Gammaproteobacteria; Vibrionales; Vibrionaceae; Vibrio; Vibrio harveyi group
NC_020848	RefSeq	RVG	G442	76,705	42.5	Vibrio phage VBP47	Viruses; dsDNA viruses, no RNA stage; Caudovirales; Podoviridae; N4likevirus; unclassified N4likevirus.	754073	Bacteria; Proteobacteria; Gammaproteobacteria; Vibrionales; Vibrionaceae; Vibrio; Vibrio harveyi group
NC_020849	RefSeq	RVG	G442	76,802	38.7	Pseudoalteromonas phage pYD6-A	Viruses; dsDNA viruses, no RNA stage; Caudovirales; Podoviridae; N4likevirus; unclassified N4likevirus.	754052	Bacteria; Proteobacteria; Gammaproteobacteria; Alteromonadales; Pseudoalteromonadaceae; Pseudoalteromonas
NC_023581	RefSeq	RVG	G443	77,792	37.8	Acinetobacter phage Presley	Viruses; dsDNA viruses, no RNA stage; Caudovirales; Podoviridae; N4likevirus; unclassified N4likevirus.	1406780	Bacteria; Proteobacteria; Gammaproteobacteria; Pseudomonadales; Moraxellaceae; Acinetobacter; Acinetobacter calcoaceticus/baumannii complex
KF005320	EBI	RVG	G444	104,036	43.2	Alteromonas phage vB_AmaP_AD45-P2	Viruses; dsDNA viruses, no RNA stage; Caudovirales; Podoviridae.	1300005	-
NC_021532	RefSeq	RVG	G444	103,910	43.2	Alteromonas phage vB_AmaP_AD45-P1	Viruses; dsDNA viruses, no RNA stage; Caudovirales; Podoviridae.	1300004	Bacteria; Proteobacteria; Gammaproteobacteria; Alteromonadales; Alteromonadaceae; Alteromonas
KF005319	EBI	RVG	G444	100,619	43.2	Alteromonas phage vB_AmaP_AD45-P4	Viruses; dsDNA viruses, no RNA stage; Caudovirales; Podoviridae.	1300007	-
KF005318	EBI	RVG	G444	101,724	43.2	Alteromonas phage vB_AmaP_AD45-P3	Viruses; dsDNA viruses, no RNA stage; Caudovirales; Podoviridae.	1300006	-
NC_004629	RefSeq	RVG	G445	280,334	36.8	Pseudomonas phage phiKZ	Viruses; dsDNA viruses, no RNA stage; Caudovirales; Myoviridae; Phikzlikevirus.	169683	Bacteria; Proteobacteria; Gammaproteobacteria; Pseudomonadales; Pseudomonadaceae; Pseudomonas; Pseudomonas aeruginosa group
NC_028999	RefSeq	RVG	G445	309,208	47.7	Pseudomonas phage PhiPA3	Viruses; dsDNA viruses, no RNA stage; Caudovirales; Myoviridae; Phikzlikevirus; unclassified phiKZ-like viruses.	998086	-
NC_010821	RefSeq	RVG	G445	316,674	45.3	Pseudomonas phage 201phi2-1	Viruses; dsDNA viruses, no RNA stage; Caudovirales; Myoviridae; Phikzlikevirus; unclassified phiKZ-like viruses.	198110	Bacteria; Proteobacteria; Gammaproteobacteria; Pseudomonadales; Pseudomonadaceae; Pseudomonas; Pseudomonas chlororaphis group
NC_028950	RefSeq	RVG	G446	223,932	52.1	Ralstonia phage RSL2	Viruses; dsDNA viruses, no RNA stage; Caudovirales; Myoviridae.	1585840	-
NC_028899	RefSeq	RVG	G446	222,888	52.3	Ralstonia phage RSF1	Viruses; dsDNA viruses, no RNA stage; Caudovirales; Myoviridae.	1689679	-
NC_023557	RefSeq	RVG	G447	271,084	49.9	Erwinia phage Ea35-70	Viruses; dsDNA viruses, no RNA stage; Caudovirales; Myoviridae.	1429768	Bacteria; Proteobacteria; Gammaproteobacteria; Enterobacteriales; Enterobacteriaceae; Erwinia
NC_023610	RefSeq	RVG	G448	218,339	52.3	Erwinia phage PhiEaH1	Viruses; dsDNA viruses, no RNA stage; Caudovirales; Siphoviridae.	1401669	Bacteria; Proteobacteria; Gammaproteobacteria; Enterobacteriales; Enterobacteriaceae; Erwinia
NC_019929	RefSeq	RVG	G449	243,050	51.3	Erwinia phage phiEaH2	Viruses; dsDNA viruses, no RNA stage; Caudovirales; Siphoviridae.	1029988	Bacteria; Proteobacteria; Gammaproteobacteria; Enterobacteriales; Enterobacteriaceae; Erwinia
NC_027402	RefSeq	RVG	G449	240,413	48.5	Salmonella phage SPN3US	Viruses; dsDNA viruses, no RNA stage; Caudovirales; Myoviridae.	1090134	Bacteria; Proteobacteria; Gammaproteobacteria; Enterobacteriales; Enterobacteriaceae; Salmonella; Salmonella enterica
NC_021531	RefSeq	RVG	G449	223,989	50.1	Cronobacter phage CR5	Viruses; dsDNA viruses, no RNA stage; Caudovirales; Myoviridae.	1195085	Bacteria; Proteobacteria; Gammaproteobacteria; Enterobacteriales; Enterobacteriaceae; Cronobacter
JQ340088	EBI	RVG	G450	167,292	35.4	Halocynthia phage JM-2012	Viruses; dsDNA viruses, no RNA stage; Caudovirales; Myoviridae.	1173297	-
NC_016571	RefSeq	RVG	G451	284,757	43.5	Pseudomonas phage OBP	Viruses; dsDNA viruses, no RNA stage; Caudovirales; Myoviridae.	1124849	Bacteria; Proteobacteria; Gammaproteobacteria; Pseudomonadales; Pseudomonadaceae; Pseudomonas; Pseudomonas fluorescens group
NC_007623	RefSeq	RVG	G452	211,215	49.3	Pseudomonas phage EL	Viruses; dsDNA viruses, no RNA stage; Caudovirales; Myoviridae; Phikzlikevirus.	273133	Bacteria; Proteobacteria; Gammaproteobacteria; Pseudomonadales; Pseudomonadaceae; Pseudomonas; Pseudomonas aeruginosa group
TARA_ERS488448_N000032	TOV	EVG	G453	103,286	44.6	-	-	-	-
TARA_ERS490053_N000033	TOV	EVG	G453	103,825	54.8	-	-	-	-
TARA_ERS490053_N000029	TOV	EVG	G453	108,936	39.9	-	-	-	-
TARA_ERS488836_N000019	TOV	EVG	G453	106,654	42.7	-	-	-	-
TARA_ERS489285_N000041	TOV	EVG	G453	107,258	46.5	-	-	-	-
TARA_ERS488929_N000013	TOV	EVG	G453	105,709	44.9	-	-	-	-
TARA_ERS490452_N000042	TOV	EVG	G453	102,889	49.4	-	-	-	-
TARA_ERS488354_N000017	TOV	EVG	G453	106,116	51.6	-	-	-	-
TARA_ERS489285_N000047	TOV	EVG	G453	103,026	45	-	-	-	-
TARA_ERS488701_N000010	TOV	EVG	G453	111,484	45.8	-	-	-	-
TARA_ERS490346_N000059	TOV	EVG	G453	115,336	51.3	-	-	-	-
TARA_ERS490120_N000069	TOV	EVG	G453	117,196	51.2	-	-	-	-
TARA_ERS489285_N000032	TOV	EVG	G453	117,940	50.9	-	-	-	-
TARA_ERS490142_N000090	TOV	EVG	G453	103,564	44.9	-	-	-	-
TARA_ERS489285_N000043	TOV	EVG	G453	105,099	44.2	-	-	-	-
TARA_ERS489285_N000042	TOV	EVG	G453	105,645	48.4	-	-	-	-
TARA_ERS488929_N000012	TOV	EVG	G453	110,289	44.4	-	-	-	-
TARA_ERS490452_N000040	TOV	EVG	G454	103,982	47.6	-	-	-	-
TARA_ERS489113_N000073	TOV	EVG	G455	90,523	34	-	-	-	-
TARA_ERS490204_N000056	TOV	EVG	G455	93,191	33.6	-	-	-	-
TARA_ERS490494_N000007	TOV	EVG	G455	120,961	35.5	-	-	-	-
TARA_ERS490142_N000117	TOV	EVG	G455	84,197	36.2	-	-	-	-
TARA_ERS488813_N000045	TOV	EVG	G455	88,776	33.1	-	-	-	-
TARA_ERS490053_N000040	TOV	EVG	G455	93,958	37.3	-	-	-	-
TARA_ERS490346_N000089	TOV	EVG	G455	92,558	38.6	-	-	-	-
OBV_N00010	OBV	EVG	G455	96,913	39.6	-	-	-	-
TARA_ERS488892_N000014	TOV	EVG	G455	97,488	36.5	-	-	-	-

TARA_ERS490120_N000097	TOV	EVG	G455	97,899	36.6	-	-	-	-
TARA_ERS490494_N000013	TOV	EVG	G456	93,198	34.7	-	-	-	-
TARA_ERS488929_N000018	TOV	EVG	G456	94,063	30.1	-	-	-	-
TARA_ERS489285_N000062	TOV	EVG	G456	91,174	36	-	-	-	-
TARA_ERS490494_N000035	TOV	EVG	G457	68,447	43.8	-	-	-	-
OBV_N00021	OBV	EVG	G457	66,977	43.7	-	-	-	-
TARA_ERS490320_N000018	TOV	EVG	G457	70,055	45.9	-	-	-	-
TARA_ERS490285_N000129	TOV	EVG	G457	70,057	45.9	-	-	-	-
TARA_ERS490452_N000108	TOV	EVG	G457	62,571	41.2	-	-	-	-
TARA_ERS490120_N000153	TOV	EVG	G457	73,216	44.6	-	-	-	-
TARA_ERS488836_N000030	TOV	EVG	G457	74,926	42.7	-	-	-	-
TARA_ERS478007_N000020	TOV	EVG	G457	65,333	50.7	-	-	-	-
TARA_ERS491107_N000077	TOV	EVG	G457	68,117	47.7	-	-	-	-
TARA_ERS490452_N000106	TOV	EVG	G457	64,614	36.6	-	-	-	-
TARA_ERS490053_N000090	TOV	EVG	G457	67,663	43.6	-	-	-	-
TARA_ERS492160_N000171	TOV	EVG	G457	61,352	40.9	-	-	-	-
TARA_ERS492198_N000063	TOV	EVG	G457	61,042	46.7	-	-	-	-
TARA_ERS488929_N000052	TOV	EVG	G457	64,308	39.9	-	-	-	-
TARA_ERS490346_N000180	TOV	EVG	G457	59,656	43.2	-	-	-	-
TARA_ERS488836_N000047	TOV	EVG	G457	60,451	45.3	-	-	-	-
TARA_ERS478007_N000024	TOV	EVG	G457	61,947	46.2	-	-	-	-
TARA_ERS488836_N000039	TOV	EVG	G457	62,847	40.8	-	-	-	-
TARA_ERS489084_N000047	TOV	EVG	G457	60,194	59.6	-	-	-	-
TARA_ERS490204_N000107	TOV	EVG	G457	63,367	47.3	-	-	-	-
TARA_ERS489084_N000038	TOV	EVG	G457	65,382	48.1	-	-	-	-
TARA_ERS488558_N000319	TOV	EVG	G457	66,271	43.1	-	-	-	-
TARA_ERS489084_N000033	TOV	EVG	G457	69,043	42.7	-	-	-	-
TARA_ERS488701_N000035	TOV	EVG	G458	80,519	49.2	-	-	-	-
TARA_ERS489148_N000025	TOV	EVG	G458	80,753	49.3	-	-	-	-
TARA_ERS489285_N000092	TOV	EVG	G458	74,799	48	-	-	-	-
TARA_ERS489285_N000094	TOV	EVG	G458	74,689	51.8	-	-	-	-
TARA_ERS490204_N000419	TOV	EVG	G459	33,764	47.9	-	-	-	-
TARA_ERS488813_N000250	TOV	EVG	G459	34,712	42.2	-	-	-	-
OBV_N00106	OBV	EVG	G459	36,131	51.1	-	-	-	-
TARA_ERS488448_N000460	TOV	EVG	G459	35,721	50.6	-	-	-	-
TARA_ERS490346_N000432	TOV	EVG	G459	36,611	40.9	-	-	-	-
TARA_ERS490204_N000428	TOV	EVG	G459	33,465	55.8	-	-	-	-
TARA_ERS488518_N000199	TOV	EVG	G460	60,124	44	-	-	-	-
TARA_ERS488448_N000098	TOV	EVG	G461	66,538	58.9	-	-	-	-
LDNO01000009	cryoconite	EVG	G462	36,212	49.1	-	-	-	-
LDNN01000002	cryoconite	EVG	G463	82,463	31.1	-	-	-	-
TARA_ERS490142_N000652	TOV	EVG	G464	27,511	34.6	-	-	-	-
TARA_ERS488673_N000574	TOV	EVG	G464	29,261	33.7	-	-	-	-
TARA_ERS490610_N000847	TOV	EVG	G464	28,437	33.9	-	-	-	-
TARA_ERS488892_N000362	TOV	EVG	G464	29,313	35.4	-	-	-	-
TARA_ERS488929_N000353	TOV	EVG	G464	29,437	35.9	-	-	-	-
TARA_ERS490320_N000250	TOV	EVG	G464	28,550	30	-	-	-	-
TARA_ERS490285_N000680	TOV	EVG	G464	28,619	30	-	-	-	-
TARA_ERS489603_N000365	TOV	EVG	G464	26,458	30.9	-	-	-	-
TARA_ERS488929_N000326	TOV	EVG	G464	31,402	29.4	-	-	-	-
TARA_ERS489943_N000539	TOV	EVG	G464	32,061	29.7	-	-	-	-
TARA_ERS489285_N000521	TOV	EVG	G464	28,546	30	-	-	-	-
TARA_ERS488558_N001036	TOV	EVG	G464	28,671	28.5	-	-	-	-
TARA_ERS490346_N000748	TOV	EVG	G464	26,660	27.4	-	-	-	-
TARA_ERS488929_N000431	TOV	EVG	G465	26,417	38	-	-	-	-
TARA_ERS488892_N000437	TOV	EVG	G465	26,527	35.7	-	-	-	-
TARA_ERS489943_N000604	TOV	EVG	G465	29,847	38.8	-	-	-	-
TARA_ERS490026_N000258	TOV	EVG	G465	29,847	38.8	-	-	-	-
TARA_ERS491107_N000346	TOV	EVG	G465	29,329	37.8	-	-	-	-
TARA_ERS489285_N000510	TOV	EVG	G465	28,850	54.2	-	-	-	-
TARA_ERS488892_N000386	TOV	EVG	G465	28,135	44.2	-	-	-	-
TARA_ERS488813_N000390	TOV	EVG	G465	28,138	44.1	-	-	-	-
TARA_ERS490142_N000611	TOV	EVG	G465	28,523	38.2	-	-	-	-
TARA_ERS489285_N000650	TOV	EVG	G466	24,192	55.7	-	-	-	-
TARA_ERS488892_N000336	TOV	EVG	G466	30,217	54.3	-	-	-	-
TARA_ERS478052_N000622	TOV	EVG	G466	25,519	49.4	-	-	-	-
TARA_ERS488929_N000450	TOV	EVG	G466	25,852	49.5	-	-	-	-
TARA_ERS490452_N000458	TOV	EVG	G466	27,549	34.2	-	-	-	-
TARA_ERS488448_N000706	TOV	EVG	G466	29,166	55.7	-	-	-	-
TARA_ERS490180_N000462	TOV	EVG	G466	31,511	51.5	-	-	-	-
TARA_ERS490204_N000584	TOV	EVG	G467	28,748	42.5	-	-	-	-
TARA_ERS489943_N000597	TOV	EVG	G468	30,131	31.7	-	-	-	-
TARA_ERS490026_N000253	TOV	EVG	G468	30,131	31.7	-	-	-	-
TARA_ERS490120_N000684	TOV	EVG	G468	30,186	31.2	-	-	-	-
TARA_ERS488892_N000313	TOV	EVG	G468	31,011	31.6	-	-	-	-

TARA_ERS488929_N000329	TOV	EVG	G468	31,107	31.6	-	-	-	-
TARA_ERS488558_N001047	TOV	EVG	G468	28,442	29	-	-	-	-
TARA_ERS488589_N000412	TOV	EVG	G468	28,442	29	-	-	-	-
TARA_ERS490346_N000593	TOV	EVG	G468	31,154	30.9	-	-	-	-
TARA_ERS490494_N000428	TOV	EVG	G468	29,486	31.8	-	-	-	-
TARA_ERS488892_N000353	TOV	EVG	G468	29,612	31.9	-	-	-	-
TARA_ERS489084_N000419	TOV	EVG	G468	29,535	32.3	-	-	-	-
TARA_ERS489059_N000262	TOV	EVG	G468	26,885	31	-	-	-	-
TARA_ERS478052_N000528	TOV	EVG	G468	28,286	31.2	-	-	-	-
TARA_ERS492160_N000662	TOV	EVG	G468	26,933	33	-	-	-	-
TARA_ERS488836_N000297	TOV	EVG	G468	28,519	32	-	-	-	-
TARA_ERS492160_N000553	TOV	EVG	G468	29,979	34.2	-	-	-	-
TARA_ERS488892_N000383	TOV	EVG	G468	28,309	30.9	-	-	-	-
TARA_ERS490320_N000240	TOV	EVG	G468	29,360	30	-	-	-	-
TARA_ERS490346_N000651	TOV	EVG	G468	29,323	31.3	-	-	-	-
TARA_ERS490120_N000691	TOV	EVG	G468	29,985	33	-	-	-	-
TARA_ERS490320_N000225	TOV	EVG	G468	30,457	32.6	-	-	-	-
TARA_ERS490452_N000430	TOV	EVG	G468	28,723	31.6	-	-	-	-
TARA_ERS490610_N000851	TOV	EVG	G468	28,361	31.3	-	-	-	-
TARA_ERS490320_N000236	TOV	EVG	G468	29,758	29.9	-	-	-	-
TARA_ERS490953_N000263	TOV	EVG	G468	27,132	30.8	-	-	-	-
AP013521	uvMED	EVG	G468	35,858	30.3	uvMED-CGR-U-MedDCM-OCT-S42-C60 (G21)	-	-	-
TARA_ERS488499_N000464	TOV	EVG	G468	26,865	32.3	-	-	-	-
OBV_N00191	OBV	EVG	G468	28,525	33.3	-	-	-	-
TARA_ERS488448_N000760	TOV	EVG	G468	28,147	37	-	-	-	-
TARA_ERS488892_N000395	TOV	EVG	G469	27,741	36.9	-	-	-	-
TARA_ERS478007_N000306	TOV	EVG	G469	29,492	29.7	-	-	-	-
TARA_ERS488813_N000378	TOV	EVG	G469	28,594	33.5	-	-	-	-
TARA_ERS490953_N000223	TOV	EVG	G469	29,024	32	-	-	-	-
TARA_ERS488813_N000350	TOV	EVG	G470	29,877	32.7	-	-	-	-
OBV_N00163	OBV	EVG	G470	30,613	32.5	-	-	-	-
TARA_ERS490610_N000705	TOV	EVG	G470	31,746	32.8	-	-	-	-
TARA_ERS488340_N000757	TOV	EVG	G470	33,405	31.3	-	-	-	-
TARA_ERS488354_N000257	TOV	EVG	G470	33,406	31.3	-	-	-	-
TARA_ERS490610_N000703	TOV	EVG	G470	31,840	31	-	-	-	-
TARA_ERS490346_N000709	TOV	EVG	G470	27,787	32.2	-	-	-	-
TARA_ERS490142_N000518	TOV	EVG	G470	32,633	30.9	-	-	-	-
TARA_ERS489285_N000407	TOV	EVG	G470	33,408	32.3	-	-	-	-
TARA_ERS478007_N000181	TOV	EVG	G470	35,841	31.9	-	-	-	-
TARA_ERS488929_N000303	TOV	EVG	G470	32,639	31.5	-	-	-	-
TARA_ERS490053_N000494	TOV	EVG	G470	30,804	29.8	-	-	-	-
TARA_ERS490120_N000662	TOV	EVG	G470	30,804	29.8	-	-	-	-
TARA_ERS490610_N000637	TOV	EVG	G470	33,485	31.6	-	-	-	-
TARA_ERS490953_N000167	TOV	EVG	G470	34,402	28.9	-	-	-	-
TARA_ERS478007_N000362	TOV	EVG	G470	27,484	38.4	-	-	-	-
TARA_ERS492160_N000620	TOV	EVG	G470	27,975	38.8	-	-	-	-
TARA_ERS490953_N000228	TOV	EVG	G470	28,885	33.7	-	-	-	-
TARA_ERS490180_N000497	TOV	EVG	G470	29,922	31.5	-	-	-	-
TARA_ERS490285_N000504	TOV	EVG	G470	32,825	31.3	-	-	-	-
TARA_ERS488892_N000281	TOV	EVG	G470	32,496	36.5	-	-	-	-
TARA_ERS488813_N000388	TOV	EVG	G470	28,163	37.6	-	-	-	-
TARA_ERS488929_N000319	TOV	EVG	G470	31,568	40.7	-	-	-	-
TARA_ERS488448_N000730	TOV	EVG	G470	28,798	33.7	-	-	-	-
AP013519	uvMED	EVG	G470	30,352	30.3	uvMED-CGR-U-MedDCM-OCT-S31-C58 (G21)	-	-	-
TARA_ERS488892_N000307	TOV	EVG	G470	31,365	30.5	-	-	-	-
TARA_ERS490204_N000479	TOV	EVG	G470	31,778	31.7	-	-	-	-
TARA_ERS478052_N000461	TOV	EVG	G470	30,753	33.8	-	-	-	-
TARA_ERS490204_N000507	TOV	EVG	G470	30,917	33.5	-	-	-	-
TARA_ERS488813_N000314	TOV	EVG	G470	31,277	30.9	-	-	-	-
TARA_ERS488836_N000239	TOV	EVG	G470	31,277	30.9	-	-	-	-
TARA_ERS490120_N000663	TOV	EVG	G470	30,766	28.8	-	-	-	-
TARA_ERS490053_N000495	TOV	EVG	G470	30,769	28.8	-	-	-	-
TARA_ERS490053_N000503	TOV	EVG	G470	30,512	33.2	-	-	-	-
TARA_ERS478052_N000417	TOV	EVG	G470	32,330	32.3	-	-	-	-
TARA_ERS490346_N000682	TOV	EVG	G471	28,537	28.9	-	-	-	-
TARA_ERS488340_N000881	TOV	EVG	G471	29,545	31.6	-	-	-	-
TARA_ERS490452_N000394	TOV	EVG	G471	30,474	31.1	-	-	-	-
TARA_ERS488892_N000242	TOV	EVG	G470	34,347	36	-	-	-	-
TARA_ERS488929_N000261	TOV	EVG	G470	34,347	36	-	-	-	-
AP013514	uvMED	EVG	G470	33,701	37.4	uvMED-CGR-U-MedDCM-OCT-S26-C60 (G21)	-	-	-
TARA_ERS488558_N000869	TOV	EVG	G470	32,842	34.7	-	-	-	-
TARA_ERS490204_N000450	TOV	EVG	G470	32,875	32.5	-	-	-	-
TARA_ERS490346_N000523	TOV	EVG	G470	33,305	31.3	-	-	-	-
TARA_ERS488929_N000231	TOV	EVG	G470	35,722	31.3	-	-	-	-
TARA_ERS488558_N000828	TOV	EVG	G470	34,197	32.6	-	-	-	-

TARA_ERS488518_N000595	TOV	EVG	G470	32,901	36.1	-	-	-	-
AP013515	uvMED	EVG	G470	37,598	32.8	uvMED-CGR-U-MedDCM-OCT-S27-C25 (G21)	-	-	-
TARA_ERS490120_N000566	TOV	EVG	G470	34,047	33.6	-	-	-	-
TARA_ERS490953_N000238	TOV	EVG	G470	28,603	33	-	-	-	-
TARA_ERS490120_N000726	TOV	EVG	G470	28,828	33.4	-	-	-	-
TARA_ERS489943_N000570	TOV	EVG	G472	31,334	31.1	-	-	-	-
TARA_ERS490346_N000483	TOV	EVG	G472	34,600	31.5	-	-	-	-
AP013517	uvMED	EVG	G472	34,524	33.4	uvMED-CGR-U-MedDCM-OCT-S30-C51 (G21)	-	-	-
TARA_ERS490285_N000586	TOV	EVG	G471	30,741	32.8	-	-	-	-
TARA_ERS492198_N000332	TOV	EVG	G471	31,746	31	-	-	-	-
TARA_ERS488813_N000325	TOV	EVG	G471	30,736	34.1	-	-	-	-
AP013516	uvMED	EVG	G471	35,191	36.6	uvMED-CGR-U-MedDCM-OCT-S29-C44 (G21)	-	-	-
OBV_N00119	OBV	EVG	G471	34,805	34.7	-	-	-	-
TARA_ERS490953_N000132	TOV	EVG	G473	38,500	35.3	-	-	-	-
TARA_ERS488340_N000863	TOV	EVG	G474	30,037	53.3	-	-	-	-
NC_018272	RefSeq	RVG	G475	35,652	35.9	Persicivirga phage P12024L	Viruses; dsDNA viruses, no RNA stage; unclassified dsDNA phages.	1168479	Bacteria; Bacteroidetes/Chlorobi group; Bacteroidetes; Flavobacteriia; Flavobacteriales; Flavobacteriaceae
NC_018271	RefSeq	RVG	G475	35,700	35.5	Persicivirga phage P12024S	Viruses; dsDNA viruses, no RNA stage; unclassified dsDNA phages.	1168478	Bacteria; Bacteroidetes/Chlorobi group; Bacteroidetes; Flavobacteriia; Flavobacteriales; Flavobacteriaceae
OBV_N00149	OBV	EVG	G476	31,630	34	-	-	-	-
TARA_ERS490204_N000564	TOV	EVG	G477	29,211	41	-	-	-	-
TARA_ERS488813_N000313	TOV	EVG	G478	31,351	30.5	-	-	-	-
TARA_ERS490204_N000607	TOV	EVG	G479	28,195	36.4	-	-	-	-
TARA_ERS490204_N000986	TOV	EVG	G480	21,472	35	-	-	-	-
TARA_ERS490204_N000774	TOV	EVG	G480	24,680	35.1	-	-	-	-
TARA_ERS488448_N000694	TOV	EVG	G481	29,494	34.4	-	-	-	-
TARA_ERS490610_N000665	TOV	EVG	G482	32,670	37.9	-	-	-	-
TARA_ERS492160_N000492	TOV	EVG	G482	32,671	37.9	-	-	-	-
TARA_ERS492198_N000314	TOV	EVG	G482	32,667	37.9	-	-	-	-
TARA_ERS488448_N000517	TOV	EVG	G482	33,832	34.7	-	-	-	-
TARA_ERS490204_N000364	TOV	EVG	G482	35,435	33.3	-	-	-	-
TARA_ERS488836_N000172	TOV	EVG	G482	35,836	27.8	-	-	-	-
TARA_ERS488448_N000283	TOV	EVG	G483	44,203	36.3	-	-	-	-
OBV_N00135	OBV	EVG	G484	32,884	41	-	-	-	-
TARA_ERS488929_N000242	TOV	EVG	G485	35,110	32.1	-	-	-	-
TARA_ERS488892_N000223	TOV	EVG	G485	35,111	32.1	-	-	-	-
TARA_ERS489943_N000468	TOV	EVG	G485	35,327	32.7	-	-	-	-
TARA_ERS490346_N000494	TOV	EVG	G485	34,217	30.8	-	-	-	-
TARA_ERS488673_N000441	TOV	EVG	G485	34,985	31.2	-	-	-	-
TARA_ERS488892_N000247	TOV	EVG	G485	34,102	34.8	-	-	-	-
TARA_ERS488929_N000266	TOV	EVG	G485	34,102	34.8	-	-	-	-
TARA_ERS488892_N000274	TOV	EVG	G485	33,003	35.7	-	-	-	-
TARA_ERS492198_N000305	TOV	EVG	G485	33,109	33	-	-	-	-
TARA_ERS490610_N000729	TOV	EVG	G486	31,280	34	-	-	-	-
TARA_ERS490346_N000441	TOV	EVG	G487	36,192	30.3	-	-	-	-
TARA_ERS488892_N000171	TOV	EVG	G488	39,742	30.7	-	-	-	-
TARA_ERS488929_N000183	TOV	EVG	G488	39,742	30.8	-	-	-	-
TARA_ERS490204_N000337	TOV	EVG	G488	36,732	34.7	-	-	-	-
AP013520	uvMED	EVG	G489	37,934	35.2	uvMED-CGR-U-MedDCM-OCT-S39-C26 (G21)	-	-	-
TARA_ERS490285_N000146	TOV	EVG	G490	65,197	36.6	-	-	-	-
TARA_ERS490320_N000023	TOV	EVG	G490	65,347	36.6	-	-	-	-
TARA_ERS490142_N000102	TOV	EVG	G491	93,332	32.4	-	-	-	-
TARA_ERS488836_N000162	TOV	EVG	G492	36,428	30.4	-	-	-	-
TARA_ERS490053_N000309	TOV	EVG	G492	38,070	32	-	-	-	-
TARA_ERS490204_N000548	TOV	EVG	G493	29,655	37.1	-	-	-	-
TARA_ERS489113_N000365	TOV	EVG	G493	29,974	35.7	-	-	-	-
TARA_ERS488813_N000345	TOV	EVG	G493	30,006	31.4	-	-	-	-
TARA_ERS490953_N000224	TOV	EVG	G493	29,008	34.1	-	-	-	-
TARA_ERS488340_N000879	TOV	EVG	G493	29,588	39	-	-	-	-
TARA_ERS488813_N000219	TOV	EVG	G493	36,250	31.6	-	-	-	-
TARA_ERS488673_N000250	TOV	EVG	G493	50,564	31.2	-	-	-	-
TARA_ERS488448_N000671	TOV	EVG	G493	30,051	33.5	-	-	-	-
TARA_ERS478007_N000286	TOV	EVG	G493	30,051	33.5	-	-	-	-
TARA_ERS488892_N000246	TOV	EVG	G493	34,102	31.1	-	-	-	-
TARA_ERS488929_N000267	TOV	EVG	G493	34,102	31	-	-	-	-
AP013513	uvMED	EVG	G493	32,413	33.1	uvMED-CGR-C84-MedDCM-OCT-S32-C77 (G21)	-	-	-
AP013512	uvMED	EVG	G493	32,707	32.9	uvMED-CGR-C84-MedDCM-OCT-S29-C65 (G21)	-	-	-
TARA_ERS488448_N000780	TOV	EVG	G493	27,507	35.3	-	-	-	-
TARA_ERS490346_N000577	TOV	EVG	G493	31,410	30.8	-	-	-	-
TARA_ERS488892_N000248	TOV	EVG	G493	34,079	34.7	-	-	-	-
TARA_ERS488929_N000269	TOV	EVG	G493	34,080	34.7	-	-	-	-
TARA_ERS488813_N000257	TOV	EVG	G493	34,080	34.6	-	-	-	-
TARA_ERS490346_N000499	TOV	EVG	G493	34,043	33.8	-	-	-	-
TARA_ERS490180_N000467	TOV	EVG	G493	31,368	32.4	-	-	-	-
TARA_ERS490026_N000227	TOV	EVG	G493	31,368	32.4	-	-	-	-

TARA_ERS490610_N000776	TOV	EVG	G494	29,842	31.2	-	-	-	-
TARA_ERS490142_N000530	TOV	EVG	G494	32,219	32.6	-	-	-	-
TARA_ERS490320_N000210	TOV	EVG	G494	31,461	31.4	-	-	-	-
TARA_ERS489113_N000178	TOV	EVG	G495	48,132	44.8	-	-	-	-
TARA_ERS489059_N000088	TOV	EVG	G495	48,178	44.7	-	-	-	-
TARA_ERS490204_N000198	TOV	EVG	G495	47,512	43.6	-	-	-	-
TARA_ERS490494_N000312	TOV	EVG	G495	34,206	37.6	-	-	-	-
TARA_ERS489113_N000192	TOV	EVG	G495	44,762	36.3	-	-	-	-
TARA_ERS489113_N000167	TOV	EVG	G495	49,653	47.1	-	-	-	-
TARA_ERS492198_N000152	TOV	EVG	G495	44,696	33.9	-	-	-	-
TARA_ERS490142_N000310	TOV	EVG	G495	45,628	46.1	-	-	-	-
TARA_ERS488558_N000916	TOV	EVG	G495	31,634	34.4	-	-	-	-
TARA_ERS488589_N000343	TOV	EVG	G495	31,634	34.3	-	-	-	-
TARA_ERS489084_N000250	TOV	EVG	G495	36,153	32.4	-	-	-	-
TARA_ERS492198_N000110	TOV	EVG	G495	50,387	48.4	-	-	-	-
TARA_ERS488892_N000082	TOV	EVG	G495	52,999	40.2	-	-	-	-
TARA_ERS490204_N000187	TOV	EVG	G495	48,578	48.6	-	-	-	-
TARA_ERS488701_N000314	TOV	EVG	G496	32,771	38.8	-	-	-	-
TARA_ERS488354_N000291	TOV	EVG	G497	31,728	52.2	-	-	-	-
TARA_ERS488589_N000335	TOV	EVG	G497	32,261	53	-	-	-	-
TARA_ERS492198_N000292	TOV	EVG	G497	33,416	53.9	-	-	-	-
TARA_ERS490320_N000054	TOV	EVG	G497	51,328	45	-	-	-	-
TARA_ERS490557_N000530	TOV	EVG	G498	30,430	48.5	-	-	-	-
TARA_ERS488589_N000047	TOV	EVG	G499	62,773	46.2	-	-	-	-
TARA_ERS490610_N000298	TOV	EVG	G500	50,954	48.8	-	-	-	-
TARA_ERS490053_N000408	TOV	EVG	G501	33,779	64.5	-	-	-	-
TARA_ERS490026_N000031	TOV	EVG	G502	56,246	59.1	-	-	-	-
LDNN01000024	cryoconite	EVG	G503	32,761	40.8	-	-	-	-
TARA_ERS490346_N000191	TOV	EVG	G504	58,641	35.6	-	-	-	-
TARA_ERS490142_N000196	TOV	EVG	G504	61,244	36.9	-	-	-	-
TARA_ERS490610_N000223	TOV	EVG	G504	59,402	33.2	-	-	-	-
TARA_ERS492160_N000180	TOV	EVG	G504	60,432	33.1	-	-	-	-
TARA_ERS490142_N000184	TOV	EVG	G504	64,301	33.3	-	-	-	-
TARA_ERS490346_N000156	TOV	EVG	G504	64,343	33.9	-	-	-	-
TARA_ERS488892_N000044	TOV	EVG	G504	63,618	34.1	-	-	-	-
TARA_ERS488813_N000075	TOV	EVG	G504	61,239	34	-	-	-	-
TARA_ERS488836_N000042	TOV	EVG	G504	61,239	34	-	-	-	-
TARA_ERS489113_N000118	TOV	EVG	G504	60,828	34.3	-	-	-	-
TARA_ERS488673_N000208	TOV	EVG	G504	59,918	33.9	-	-	-	-
TARA_ERS489059_N000052	TOV	EVG	G504	61,209	35.2	-	-	-	-
TARA_ERS490953_N000042	TOV	EVG	G504	60,069	37.5	-	-	-	-
TARA_ERS488892_N000051	TOV	EVG	G504	62,322	37.2	-	-	-	-
TARA_ERS492198_N000080	TOV	EVG	G504	57,052	38.7	-	-	-	-
TARA_ERS488558_N000375	TOV	EVG	G504	58,726	35.2	-	-	-	-
TARA_ERS488836_N000044	TOV	EVG	G504	60,815	34.1	-	-	-	-
TARA_ERS492198_N000066	TOV	EVG	G504	60,859	34.4	-	-	-	-
TARA_ERS489943_N000203	TOV	EVG	G504	58,859	33.4	-	-	-	-
TARA_ERS488836_N000045	TOV	EVG	G504	60,752	35.5	-	-	-	-
TARA_ERS490053_N000098	TOV	EVG	G504	64,076	34.4	-	-	-	-
TARA_ERS490120_N000192	TOV	EVG	G504	64,080	34.4	-	-	-	-
TARA_ERS490953_N000029	TOV	EVG	G504	65,280	32.2	-	-	-	-
TARA_ERS488448_N000127	TOV	EVG	G504	59,922	38.7	-	-	-	-
TARA_ERS488892_N000066	TOV	EVG	G504	58,035	44.9	-	-	-	-
TARA_ERS490120_N000202	TOV	EVG	G504	62,339	52.4	-	-	-	-
TARA_ERS488892_N000060	TOV	EVG	G504	59,248	44	-	-	-	-
AAA300J04_N004	SAG	EVG	G505	42,985	37.3	-	-	-	-
TARA_ERS488589_N000065	TOV	EVG	G506	57,546	31.2	-	-	-	-
OBV_N00024	OBV	EVG	G506	64,322	36.3	-	-	-	-
OBV_N00023	OBV	EVG	G506	64,495	32.3	-	-	-	-
OBV_N00025	OBV	EVG	G506	62,085	39.4	-	-	-	-
TARA_ERS488892_N000039	TOV	EVG	G507	65,997	33.5	-	-	-	-
TARA_ERS488929_N000048	TOV	EVG	G507	66,878	33.5	-	-	-	-
TARA_ERS490557_N000120	TOV	EVG	G507	62,902	34.1	-	-	-	-
KC821612	EBI	RVG	G508	72,529	38.1	Cellulophaga phage phi40:1	Viruses; dsDNA viruses, no RNA stage; Caudovirales; Podoviridae.	1327975	-
NC_021796	RefSeq	RVG	G508	72,534	38.1	Cellulophaga phage phi38:1	Viruses; dsDNA viruses, no RNA stage; Caudovirales; Podoviridae.	1327977	Bacteria; Bacteroidetes/Chlorobi group; Bacteroidetes; Flavobacteriia; Flavobacteriales; Flavobacteriaceae; Cellulophaga
TARA_ERS488448_N000174	TOV	EVG	G509	52,615	41.9	-	-	-	-
TARA_ERS490142_N000230	TOV	EVG	G509	56,641	40.7	-	-	-	-
TARA_ERS488589_N000042	TOV	EVG	G509	65,586	45.5	-	-	-	-
TARA_ERS488340_N000320	TOV	EVG	G509	59,913	52	-	-	-	-
TARA_ERS488757_N000031	TOV	EVG	G509	55,713	38.4	-	-	-	-
TARA_ERS489285_N000160	TOV	EVG	G509	55,122	38.2	-	-	-	-
TARA_ERS488737_N000105	TOV	EVG	G509	61,052	49	-	-	-	-
TARA_ERS492160_N000191	TOV	EVG	G509	59,159	51.2	-	-	-	-
TARA_ERS488354_N000081	TOV	EVG	G510	55,181	38.1	-	-	-	-

TARA_ERS488340_N000358	TOV	EVG	G510	55,181	38.2	-	-	-	-
TARA_ERS490053_N000092	TOV	EVG	G511	66,381	32.9	-	-	-	-
TARA_ERS490120_N000180	TOV	EVG	G511	66,381	32.9	-	-	-	-
TARA_ERS489943_N000163	TOV	EVG	G511	66,381	32.9	-	-	-	-
TARA_ERS490452_N000120	TOV	EVG	G512	60,475	32.4	-	-	-	-
TARA_ERS490053_N000111	TOV	EVG	G513	61,562	32.6	-	-	-	-
TARA_ERS490120_N000208	TOV	EVG	G513	61,562	32.6	-	-	-	-
TARA_ERS489285_N000130	TOV	EVG	G514	61,028	44.5	-	-	-	-
TARA_ERS488757_N000013	TOV	EVG	G515	79,822	38.4	-	-	-	-
TARA_ERS490346_N000098	TOV	EVG	G515	86,173	38.9	-	-	-	-
TARA_ERS488737_N000057	TOV	EVG	G515	85,654	33.2	-	-	-	-
TARA_ERS490953_N000012	TOV	EVG	G515	90,349	35.3	-	-	-	-
TARA_ERS488448_N000054	TOV	EVG	G515	80,198	45.8	-	-	-	-
TARA_ERS478007_N000009	TOV	EVG	G515	80,211	45.9	-	-	-	-
TARA_ERS490120_N000114	TOV	EVG	G515	85,766	46.7	-	-	-	-
TARA_ERS489113_N000095	TOV	EVG	G516	77,061	47.7	-	-	-	-
TARA_ERS489059_N000026	TOV	EVG	G516	82,434	34	-	-	-	-
TARA_ERS490204_N000073	TOV	EVG	G517	82,017	48.7	-	-	-	-
TARA_ERS489285_N000060	TOV	EVG	G518	92,954	34	-	-	-	-
LDNP01000001	cryoconite	EVG	G519	85,669	28.1	-	-	-	-
KJ003983	EBI	RVG	G520	99,915	31.9	IAS virus	Viruses; unclassified viruses.	1450749	-
TARA_ERS490285_N000080	TOV	EVG	G521	91,301	33.3	-	-	-	-
TARA_ERS491107_N000042	TOV	EVG	G522	93,286	34.3	-	-	-	-
TARA_ERS490346_N000066	TOV	EVG	G523	112,790	33.2	-	-	-	-
TARA_ERS492160_N000078	TOV	EVG	G523	112,853	33.4	-	-	-	-
TARA_ERS488813_N000030	TOV	EVG	G524	111,519	36.1	-	-	-	-
TARA_ERS488589_N000051	TOV	EVG	G525	62,250	31.7	-	-	-	-
TARA_ERS490026_N000012	TOV	EVG	G525	69,052	33.6	-	-	-	-
TARA_ERS488813_N000081	TOV	EVG	G525	57,631	32.3	-	-	-	-
TARA_ERS488836_N000036	TOV	EVG	G526	65,692	31.2	-	-	-	-
TARA_ERS488929_N000049	TOV	EVG	G526	65,698	31.2	-	-	-	-
TARA_ERS490557_N000097	TOV	EVG	G526	70,863	32.8	-	-	-	-
TARA_ERS490180_N000143	TOV	EVG	G527	70,111	28.8	-	-	-	-
TARA_ERS490142_N000161	TOV	EVG	G527	70,111	28.8	-	-	-	-
TARA_ERS490953_N000025	TOV	EVG	G527	68,932	29.7	-	-	-	-
TARA_ERS488354_N000030	TOV	EVG	G528	77,095	36.3	-	-	-	-
TARA_ERS488340_N000225	TOV	EVG	G528	77,095	36.3	-	-	-	-
NC_021803	RefSeq	RVG	G529	72,369	32.9	Cellulophaga phage phi13:2	Viruses; dsDNA viruses, no RNA stage; Caudovirales; Podoviridae.	1328030	Bacteria; Bacteroidetes/Chlorobi group; Bacteroidetes; Flavobacteriia; Flavobacteriales; Flavobacteriaceae; Cellulophaga
NC_021789	RefSeq	RVG	G529	75,994	32.5	Cellulophaga phage phi19:3	Viruses; dsDNA viruses, no RNA stage; Caudovirales; Podoviridae.	1327971	Bacteria; Bacteroidetes/Chlorobi group; Bacteroidetes; Flavobacteriia; Flavobacteriales; Flavobacteriaceae; Cellulophaga
NC_021794	RefSeq	RVG	G529	71,443	32.9	Cellulophaga phage phi18:3	Viruses; dsDNA viruses, no RNA stage; Caudovirales; Podoviridae.	1327983	Bacteria; Bacteroidetes/Chlorobi group; Bacteroidetes; Flavobacteriia; Flavobacteriales; Flavobacteriaceae; Cellulophaga
NC_021792	RefSeq	RVG	G529	72,961	32.7	Cellulophaga phage phi46:3	Viruses; dsDNA viruses, no RNA stage; Caudovirales; Podoviridae.	1327985	Bacteria; Bacteroidetes/Chlorobi group; Bacteroidetes; Flavobacteriia; Flavobacteriales; Flavobacteriaceae; Cellulophaga
NC_021802	RefSeq	RVG	G530	53,664	31.5	Cellulophaga phage phi10:1	Viruses; dsDNA viruses, no RNA stage; Caudovirales; Siphoviridae.	1327981	Bacteria; Bacteroidetes/Chlorobi group; Bacteroidetes; Flavobacteriia; Flavobacteriales; Flavobacteriaceae; Cellulophaga
NC_021799	RefSeq	RVG	G530	57,447	31.2	Cellulophaga phage phi19:1	Viruses; dsDNA viruses, no RNA stage; Caudovirales; Siphoviridae.	1327970	Bacteria; Bacteroidetes/Chlorobi group; Bacteroidetes; Flavobacteriia; Flavobacteriales; Flavobacteriaceae; Cellulophaga
NC_006356	RefSeq	RVG	G531	36,012	30.6	Flavobacterium phage 11b	Viruses; dsDNA viruses, no RNA stage; Caudovirales; Siphoviridae.	294631	Bacteria; Bacteroidetes/Chlorobi group; Bacteroidetes; Flavobacteriia; Flavobacteriales; Flavobacteriaceae; Flavobacterium
NC_021798	RefSeq	RVG	G532	145,343	32.7	Cellulophaga phage phi17:2	Viruses; dsDNA viruses, no RNA stage; Caudovirales; Podoviridae.	1327972	Bacteria; Bacteroidetes/Chlorobi group; Bacteroidetes; Flavobacteriia; Flavobacteriales; Flavobacteriaceae; Cellulophaga
NC_021788	RefSeq	RVG	G532	145,865	32.7	Cellulophaga phage phi4:1	Viruses; dsDNA viruses, no RNA stage; Caudovirales; Podoviridae.	1328029	Bacteria; Bacteroidetes/Chlorobi group; Bacteroidetes; Flavobacteriia; Flavobacteriales; Flavobacteriaceae; Cellulophaga
TARA_ERS490388_N000065	TOV	EVG	G532	141,288	31.8	-	-	-	-
NC_021806	RefSeq	RVG	G533	100,418	29.6	Cellulophaga phage phi14:2	Viruses; dsDNA viruses, no RNA stage; Caudovirales; Podoviridae.	1327990	Bacteria; Bacteroidetes/Chlorobi group; Bacteroidetes; Flavobacteriia; Flavobacteriales; Flavobacteriaceae; Cellulophaga
NC_028924	RefSeq	RVG	G534	48,689	28.9	Polaribacter phage P12002L	Viruses; dsDNA viruses, no RNA stage; Caudovirales; Siphoviridae.	1647386	-
NC_028763	RefSeq	RVG	G534	49,847	28.9	Polaribacter phage P12002S	Viruses; dsDNA viruses, no RNA stage; Caudovirales; Siphoviridae.	1647387	-
AP013358	uvMED	EVG	G535	33,328	30.5	uvMED-CGR-U-MedDCM-OCT-S27-C45 (G1)	-	-	-
TARA_ERS490204_N000278	TOV	EVG	G536	39,973	35.3	-	-	-	-
TARA_ERS492198_N000180	TOV	EVG	G537	41,032	42	-	-	-	-
NC_019490	RefSeq	RVG	G538	49,329	34.7	Riemerella phage RAP44	Viruses; dsDNA viruses, no RNA stage; Caudovirales; Siphoviridae.	936152	Bacteria; Bacteroidetes/Chlorobi group; Bacteroidetes; Flavobacteriia; Flavobacteriales; Flavobacteriaceae; Riemerella
KC821627	EBI	RVG	G539	38,476	36.6	Cellulophaga phage phi18:2	Viruses; dsDNA viruses, no RNA stage; Caudovirales; Siphoviridae.	1327995	-
NC_021790	RefSeq	RVG	G539	39,189	36.5	Cellulophaga phage phi18:1	Viruses; dsDNA viruses, no RNA stage; Caudovirales; Siphoviridae.	1327982	Bacteria; Bacteroidetes/Chlorobi group; Bacteroidetes; Flavobacteriia; Flavobacteriales; Flavobacteriaceae; Cellulophaga
NC_021795	RefSeq	RVG	G539	38,776	36.5	Cellulophaga phage phi17:1	Viruses; dsDNA viruses, no RNA stage; Caudovirales; Siphoviridae.	1327980	Bacteria; Bacteroidetes/Chlorobi group; Bacteroidetes; Flavobacteriia; Flavobacteriales; Flavobacteriaceae; Cellulophaga
KC821615	EBI	RVG	G539	39,151	36.6	Cellulophaga phage phi12:3	Viruses; dsDNA viruses, no RNA stage; Caudovirales; Siphoviridae.	1327979	-
NC_021791	RefSeq	RVG	G539	39,148	36.6	Cellulophaga phage phi12:1	Viruses; dsDNA viruses, no RNA stage; Caudovirales; Siphoviridae.	1327976	Bacteria; Bacteroidetes/Chlorobi group; Bacteroidetes; Flavobacteriia; Flavobacteriales; Flavobacteriaceae; Cellulophaga

NC_021800	RefSeq	RVG	G540	34,844	38.3	Cellulophaga phage phi46:1	Viruses; dsDNA viruses, no RNA stage; Caudovirales; Siphoviridae.	1327974	Bacteria; Bacteroidetes/Chlorobi group; Bacteroidetes; Flavobacteriia; Flavobacteriales; Flavobacteriaceae; Cellulophaga
TARA_ERS490346_N000551	TOV	EVG	G541	32,322	40.5	-	-	-	-
TARA_ERS490320_N000194	TOV	EVG	G541	32,466	40.5	-	-	-	-
TARA_ERS490204_N000412	TOV	EVG	G541	33,909	40.3	-	-	-	-
AP013511	uvMED	EVG	G541	35,735	41.1	uvMED-CGR-C117A-MedDCM-OCT-S32-C49 (G21)	-	-	-
LDNN01000009	cryoconite	EVG	G542	44,120	33.1	-	-	-	-
TARA_ERS488518_N000387	TOV	EVG	G543	41,945	56.4	-	-	-	-
TARA_ERS488340_N000545	TOV	EVG	G543	41,945	56.4	-	-	-	-
LDNO01000008	cryoconite	EVG	G544	38,581	32.6	-	-	-	-
KC821634	EBI	RVG	G545	54,016	33.5	Cellulophaga phage phi47:1	Viruses; dsDNA viruses, no RNA stage; Caudovirales; Myoviridae.	756281	-
KC821610	EBI	RVG	G545	54,015	33.5	Cellulophaga phage phi3ST:2	Viruses; dsDNA viruses, no RNA stage; Caudovirales; Myoviridae.	1327973	-
KC821629	EBI	RVG	G545	54,012	33.5	Cellulophaga phage phi38:2	Viruses; dsDNA viruses, no RNA stage; Caudovirales; Myoviridae.	1327999	-
NC_020860	RefSeq	RVG	G545	44,557	33.9	Cellulophaga phage phiSM	Viruses; dsDNA viruses, no RNA stage; Caudovirales; Myoviridae.	756280	Bacteria; Bacteroidetes/Chlorobi group; Bacteroidetes; Flavobacteriia; Flavobacteriales; Flavobacteriaceae; Cellulophaga
KC821630	EBI	RVG	G545	54,427	33.4	Cellulophaga phage phi3:1	Viruses; dsDNA viruses, no RNA stage; Caudovirales; Myoviridae.	756279	-
NC_027125	RefSeq	RVG	G545	47,142	30.2	Flavobacterium phage FCL-2	Viruses; dsDNA viruses, no RNA stage; Caudovirales; Myoviridae.	908819	Bacteria; Bacteroidetes/Chlorobi group; Bacteroidetes; Flavobacteriia; Flavobacteriales; Flavobacteriaceae; Flavobacterium
NC_021867	RefSeq	RVG	G546	46,978	31.6	Flavobacterium phage 6H	Viruses; dsDNA viruses, no RNA stage; unclassified dsDNA phages.	1325731	Bacteria; Bacteroidetes/Chlorobi group; Bacteroidetes; Flavobacteriia; Flavobacteriales; Flavobacteriaceae; Flavobacterium
KC821621	EBI	RVG	G547	78,275	30.2	Cellulophaga phage phi19:2	Viruses; dsDNA viruses, no RNA stage; Caudovirales; Siphoviridae.	1327984	-
NC_020842	RefSeq	RVG	G547	79,114	30.2	Cellulophaga phage phiST	Viruses; dsDNA viruses, no RNA stage; Caudovirales; Siphoviridae.	756282	Bacteria; Bacteroidetes/Chlorobi group; Bacteroidetes; Flavobacteriia; Flavobacteriales; Flavobacteriaceae; Cellulophaga
KC821625	EBI	RVG	G547	76,666	30.2	Cellulophaga phage phi13:1	Viruses; dsDNA viruses, no RNA stage; Caudovirales; Siphoviridae.	1327992	-
NC_021804	RefSeq	RVG	G548	28,760	31.3	Cellulophaga phage phi39:1	Viruses; dsDNA viruses, no RNA stage; Caudovirales; Siphoviridae.	1327993	Bacteria; Bacteroidetes/Chlorobi group; Bacteroidetes; Flavobacteriia; Flavobacteriales; Flavobacteriaceae; Cellulophaga
KF751796	EBI	RVG	G549	35,459	28.9	Campylobacter phage CJIE4-4	Viruses; unclassified phages.	1470461	-
KF751795	EBI	RVG	G549	35,417	28.9	Campylobacter phage CJIE4-3	Viruses; unclassified phages.	1470460	-
KF751797	EBI	RVG	G549	36,991	28.7	Campylobacter phage CJIE4-5	Viruses; unclassified phages.	1470462	-
KF751794	EBI	RVG	G549	36,734	28.5	Campylobacter phage CJIE4-2	Viruses; unclassified phages.	1470459	-
KF751793	EBI	RVG	G549	37,940	28.5	Campylobacter phage CJIE4-1	Viruses; unclassified phages.	1470458	-
LDNN01000007	cryoconite	EVG	G550	47,364	46.5	-	-	-	-
TARA_ERS490204_N000416	TOV	EVG	G551	33,798	47.1	-	-	-	-
TARA_ERS489148_N000277	TOV	EVG	G552	36,392	36.2	-	-	-	-
TARA_ERS490053_N000086	TOV	EVG	G553	68,024	39.6	-	-	-	-
TARA_ERS490120_N000175	TOV	EVG	G553	68,024	39.6	-	-	-	-
TARA_ERS488340_N000246	TOV	EVG	G554	72,427	53.2	-	-	-	-
LDNN01000025	cryoconite	EVG	G555	30,365	31.1	-	-	-	-
LDNN01000023	cryoconite	EVG	G555	32,795	31.2	-	-	-	-
LDNN01000005	cryoconite	EVG	G556	55,434	60.4	-	-	-	-
LDNP01000008	cryoconite	EVG	G557	36,683	47.6	-	-	-	-
LDNN01000001	cryoconite	EVG	G558	230,473	46.6	-	-	-	-
NC_016770	RefSeq	RVG	G559	47,159	38.7	Bacteroides phage B124-14	Viruses; dsDNA viruses, no RNA stage; Caudovirales; Siphoviridae.	1105171	Bacteria; Bacteroidetes/Chlorobi group; Bacteroidetes; Bacteroidia; Bacteroidales; Bacteroidaceae; Bacteroides
NC_011222	RefSeq	RVG	G559	44,929	38.6	Bacteroides phage B40-8	Viruses; dsDNA viruses, no RNA stage; Caudovirales; Siphoviridae.	99179	Bacteria; Bacteroidetes/Chlorobi group; Bacteroidetes; Bacteroidia; Bacteroidales; Bacteroidaceae; Bacteroides
NC_018276	RefSeq	RVG	G560	44,988	41.9	Croceibacter phage P2559S	Viruses; dsDNA viruses, no RNA stage; Caudovirales; Siphoviridae.	1176422	Bacteria; Bacteroidetes/Chlorobi group; Bacteroidetes; Flavobacteriia; Flavobacteriales; Flavobacteriaceae; Croceibacter
NC_023614	RefSeq	RVG	G560	43,153	38.9	Croceibacter phage P2559Y	Viruses; dsDNA viruses, no RNA stage; Caudovirales; Siphoviridae.	1327037	Bacteria; Bacteroidetes/Chlorobi group; Bacteroidetes; Flavobacteriia; Flavobacteriales; Flavobacteriaceae; Croceibacter
TARA_ERS491107_N000194	TOV	EVG	G561	42,760	32.6	-	-	-	-
KJ018210	EBI	RVG	G562	42,252	34.3	Flavobacterium sp. phage 1/32	Viruses; dsDNA viruses, no RNA stage; Caudovirales; Myoviridae.	1458858	-
LDNO01000002	cryoconite	EVG	G563	51,661	49.2	-	-	-	-
LDNO01000001	cryoconite	EVG	G564	117,148	31	-	-	-	-
NC_010353	RefSeq	RVG	G565	35,543	39.8	Streptococcus phage 858	Viruses; dsDNA viruses, no RNA stage; Caudovirales; Siphoviridae; Sfi1unalikevirus.	459845	Bacteria; Firmicutes; Bacilli; Lactobacillales; Streptococcaceae; Streptococcus
NC_007019	RefSeq	RVG	G565	34,704	40.2	Streptococcus phage 2972	Viruses; dsDNA viruses, no RNA stage; Caudovirales; Siphoviridae; Sfi1unalikevirus.	306323	Bacteria; Firmicutes; Bacilli; Lactobacillales; Streptococcaceae; Streptococcus
NC_022776	RefSeq	RVG	G565	41,757	39	Streptococcus phage TP-778L	Viruses; dsDNA viruses, no RNA stage; Caudovirales; Siphoviridae.	1385385	Bacteria; Firmicutes; Bacilli; Lactobacillales; Streptococcaceae; Streptococcus
NC_013598	RefSeq	RVG	G565	35,525	39.4	Streptococcus phage Alq132	Viruses; dsDNA viruses, no RNA stage; Caudovirales; Siphoviridae; Sfi1unalikevirus.	682370	Bacteria; Firmicutes; Bacilli; Lactobacillales; Streptococcaceae; Streptococcus
NC_004303	RefSeq	RVG	G565	43,075	38.3	Streptococcus phage O1205	Viruses; dsDNA viruses, no RNA stage; Caudovirales; Siphoviridae; Sfi1unalikevirus.	85154	Bacteria; Firmicutes; Bacilli; Lactobacillales; Streptococcaceae; Streptococcus
NC_002214	RefSeq	RVG	G565	39,807	38.6	Streptococcus phage Sfi11	Viruses; dsDNA viruses, no RNA stage; Caudovirales; Siphoviridae; Sfi1unalikevirus.	78541	Bacteria; Firmicutes; Bacilli; Lactobacillales; Streptococcaceae; Streptococcus
NC_020197	RefSeq	RVG	G565	45,606	38.8	Streptococcus phage TP-J34	Viruses; dsDNA viruses, no RNA stage; Caudovirales; Siphoviridae.	73422	Bacteria; Firmicutes; Bacilli; Lactobacillales; Streptococcaceae; Streptococcus
NC_023503	RefSeq	RVG	G565	48,800	40	Streptococcus phage 20617	Viruses; unclassified phages.	1392231	Bacteria; Firmicutes; Bacilli; Lactobacillales; Streptococcaceae; Streptococcus
NC_012753	RefSeq	RVG	G565	37,184	38	Streptococcus phage 5093	Viruses; dsDNA viruses, no RNA stage; Caudovirales; Siphoviridae.	646413	Bacteria; Firmicutes; Bacilli; Lactobacillales; Streptococcaceae; Streptococcus
NC_000871	RefSeq	RVG	G565	37,370	38.3	Streptococcus phage Sfi19	Viruses; dsDNA viruses, no RNA stage; Caudovirales; Siphoviridae; Sfi21dtunalikevirus.	72638	Bacteria; Firmicutes; Bacilli; Lactobacillales; Streptococcaceae; Streptococcus
NC_000872	RefSeq	RVG	G565	40,739	37.6	Streptococcus phage Sfi21	Viruses; dsDNA viruses, no RNA stage; Caudovirales; Siphoviridae; Sfi21dtunalikevirus.	64186	Bacteria; Firmicutes; Bacilli; Lactobacillales; Streptococcaceae; Streptococcus
NC_002072	RefSeq	RVG	G565	34,815	39.1	Streptococcus phage DT1	Viruses; dsDNA viruses, no RNA stage; Caudovirales; Siphoviridae; Sfi21dtunalikevirus.	90410	Bacteria; Firmicutes; Bacilli; Lactobacillales; Streptococcaceae; Streptococcus

NC_013645	RefSeq	RVG	G565	34,882	39	Streptococcus phage Abc2	Viruses; dsDNA viruses, no RNA stage; Caudovirales; Siphoviridae; Sfi21dtunalikevirus.	633135	Bacteria; Firmicutes; Bacilli; Lactobacillales; Streptococcaceae; Streptococcus
NC_002185	RefSeq	RVG	G565	35,466	38.7	Streptococcus phage 7201	Viruses; dsDNA viruses, no RNA stage; Caudovirales; Siphoviridae; Sfi21dtunalikevirus.	112023	Bacteria; Firmicutes; Bacilli; Lactobacillales; Streptococcaceae; Streptococcus
NC_018285	RefSeq	RVG	G565	40,758	41.2	Streptococcus phage YMC-2011	Viruses; dsDNA viruses, no RNA stage; Caudovirales; Siphoviridae.	1051631	Bacteria; Firmicutes; Bacilli; Lactobacillales; Streptococcaceae; Streptococcus
KC413988	EBI	RVG	G566	38,940	39.9	Streptococcus phage phiST1	Viruses; dsDNA viruses, no RNA stage; Caudovirales; Podoviridae.	1277892	-
NC_019418	RefSeq	RVG	G566	37,282	39.4	Streptococcus phage phiNJ2	Viruses; dsDNA viruses, no RNA stage; Caudovirales; Siphoviridae.	1239381	Bacteria; Firmicutes; Bacilli; Lactobacillales; Streptococcaceae; Streptococcus
NC_028697	RefSeq	RVG	G566	33,900	38.4	Streptococcus phage A25	Viruses; dsDNA viruses, no RNA stage; Caudovirales; Podoviridae.	1701850	-
DQ113772	EBI	RVG	G566	38,893	38.1	Streptococcus phage MM1 1998	Viruses; dsDNA viruses, no RNA stage; Caudovirales; Siphoviridae.	341698	-
NC_003050	RefSeq	RVG	G566	40,248	38.4	Streptococcus phage MM1	Viruses; dsDNA viruses, no RNA stage; Caudovirales; Siphoviridae.	120574	Bacteria; Firmicutes; Bacilli; Lactobacillales; Streptococcaceae; Streptococcus
NC_022791	RefSeq	RVG	G566	37,375	38.3	Streptococcus phage phiBHN167	Viruses; unclassified phages.	1267169	Bacteria; Firmicutes; Bacilli; Lactobacillales; Streptococcaceae; Streptococcus
NC_010945	RefSeq	RVG	G567	39,136	40.5	Streptococcus phage PH15	Viruses; dsDNA viruses, no RNA stage; Caudovirales; Siphoviridae.	537874	Bacteria; Firmicutes; Bacilli; Lactobacillales; Streptococcaceae; Streptococcus
NC_009819	RefSeq	RVG	G568	40,539	39.7	Streptococcus phage P9	Viruses; dsDNA viruses, no RNA stage; Caudovirales; Siphoviridae.	403905	Bacteria; Firmicutes; Bacilli; Lactobacillales; Streptococcaceae; Streptococcus; Streptococcus dysgalactiae group
NC_004589	RefSeq	RVG	G568	40,014	39.7	Streptococcus pyogenes phage 315.6	Viruses; dsDNA viruses, no RNA stage; Caudovirales; Podoviridae.	198543	Bacteria; Firmicutes; Bacilli; Lactobacillales; Streptococcaceae; Streptococcus
NC_004587	RefSeq	RVG	G568	41,796	38.6	Streptococcus pyogenes phage 315.4	Viruses; dsDNA viruses, no RNA stage; Caudovirales; Podoviridae.	198541	Bacteria; Firmicutes; Bacilli; Lactobacillales; Streptococcaceae; Streptococcus
NC_003157	RefSeq	RVG	G568	43,106	38.6	Temperate phage phiNIH1.1	Viruses; dsDNA viruses, no RNA stage; Caudovirales; Siphoviridae.	173707	Bacteria; Firmicutes; Bacilli; Lactobacillales; Streptococcaceae; Streptococcus
NC_009018	RefSeq	RVG	G569	38,528	37.5	Streptococcus phage phi3396	Viruses; dsDNA viruses, no RNA stage; Caudovirales; Siphoviridae.	423476	Bacteria; Firmicutes; Bacilli; Lactobacillales; Streptococcaceae; Streptococcus; Streptococcus dysgalactiae group; Streptococcus dysgalactiae
NC_004584	RefSeq	RVG	G569	39,538	37.8	Streptococcus pyogenes phage 315.1	Viruses; dsDNA viruses, no RNA stage; Caudovirales; Podoviridae.	198538	Bacteria; Firmicutes; Bacilli; Lactobacillales; Streptococcaceae; Streptococcus
JX409894	EBI	RVG	G570	41,660	36.7	Streptococcus phage LYGO9	Viruses; dsDNA viruses, no RNA stage; Caudovirales; Siphoviridae.	1225805	-
JX409895	EBI	RVG	G570	43,028	36.8	Streptococcus phage JX01	Viruses; dsDNA viruses, no RNA stage; Caudovirales; Siphoviridae.	1225806	-
NC_004585	RefSeq	RVG	G570	41,072	38.3	Streptococcus pyogenes phage 315.2	Viruses; dsDNA viruses, no RNA stage; Caudovirales; Podoviridae.	198539	Bacteria; Firmicutes; Bacilli; Lactobacillales; Streptococcaceae; Streptococcus
NC_004588	RefSeq	RVG	G570	38,206	38.1	Streptococcus pyogenes phage 315.5	Viruses; unclassified phages.	198542	Bacteria; Firmicutes; Bacilli; Lactobacillales; Streptococcaceae; Streptococcus
NC_028700	RefSeq	RVG	G570	37,976	38.1	Streptococcus phage T12	Viruses; dsDNA viruses, no RNA stage; Caudovirales; Podoviridae.	35344	-
NC_024370	RefSeq	RVG	G571	39,965	41.1	Streptococcus phage IC1	Viruses; unclassified phages.	1448276	Bacteria; Firmicutes; Bacilli; Lactobacillales; Streptococcaceae; Streptococcus
NC_024361	RefSeq	RVG	G571	38,392	40.8	Streptococcus phage DCC1738	Viruses; unclassified phages.	1448273	Bacteria; Firmicutes; Bacilli; Lactobacillales; Streptococcaceae; Streptococcus
NC_024357	RefSeq	RVG	G571	39,360	40.6	Streptococcus phage K13	Viruses; unclassified phages.	1448274	Bacteria; Firmicutes; Bacilli; Lactobacillales; Streptococcaceae; Streptococcus
KJ417497	EBI	RVG	G571	41,323	39.9	Streptococcus phage Spn1	Viruses; unclassified phages.	1461246	-
KC348602	EBI	RVG	G572	34,367	41.5	Streptococcus phage phi891591	Viruses; dsDNA viruses, no RNA stage; Caudovirales; Podoviridae.	1289599	-
KC348601	EBI	RVG	G572	36,128	41.3	Streptococcus phage phi7917	Viruses; dsDNA viruses, no RNA stage; Caudovirales; Podoviridae.	1289598	-
KC413987	EBI	RVG	G572	35,598	41.6	Streptococcus phage phiSS12	Viruses; dsDNA viruses, no RNA stage; Caudovirales; Podoviridae.	1277891	-
KC348604	EBI	RVG	G572	35,373	41.8	Streptococcus phage phiS10	Viruses; dsDNA viruses, no RNA stage; Caudovirales; Podoviridae.	1289601	-
KC348600	EBI	RVG	G572	38,539	40.9	Streptococcus phage phi5218	Viruses; dsDNA viruses, no RNA stage; Caudovirales; Podoviridae.	1289597	-
KC348599	EBI	RVG	G572	42,703	40.6	Streptococcus phage phi30c	Viruses; dsDNA viruses, no RNA stage; Caudovirales; Podoviridae.	1289596	-
KC348598	EBI	RVG	G572	33,516	41.7	Streptococcus phage phi20c	Viruses; unclassified phages.	1289595	-
NC_008721	RefSeq	RVG	G572	36,019	41.6	Streptococcus phage SMP	Viruses; dsDNA viruses, no RNA stage; Caudovirales; Siphoviridae.	413066	Bacteria; Firmicutes; Bacilli; Lactobacillales; Streptococcaceae; Streptococcus
NC_004586	RefSeq	RVG	G573	34,419	38	Streptococcus pyogenes phage 315.3	Viruses; dsDNA viruses, no RNA stage; Caudovirales; Podoviridae.	198540	Bacteria; Firmicutes; Bacilli; Lactobacillales; Streptococcaceae; Streptococcus
NC_004996	RefSeq	RVG	G573	34,692	39.2	Streptococcus phage SM1	Viruses; dsDNA viruses, no RNA stage; Caudovirales; Siphoviridae.	157924	Bacteria; Firmicutes; Bacilli; Lactobacillales; Streptococcaceae; Streptococcus
NC_005822	RefSeq	RVG	G573	32,172	35.5	Lactococcus phage phiLC3	Viruses; dsDNA viruses, no RNA stage; Caudovirales; Siphoviridae.	12390	Bacteria; Firmicutes; Bacilli; Lactobacillales; Streptococcaceae; Lactococcus; Lactococcus lactis
NC_004302	RefSeq	RVG	G573	33,350	35.5	Lactococcus phage r1t	Viruses; dsDNA viruses, no RNA stage; Caudovirales; Siphoviridae.	43685	Bacteria; Firmicutes; Bacilli; Lactobacillales; Streptococcaceae; Lactococcus; Lactococcus lactis
NC_028666	RefSeq	RVG	G573	36,595	35.3	Streptococcus phage Str-PAP-1	Viruses; dsDNA viruses, no RNA stage; Caudovirales; Podoviridae.	1589270	-
NC_012756	RefSeq	RVG	G574	31,276	39.5	Streptococcus phage PH10	Viruses; dsDNA viruses, no RNA stage; Caudovirales; Siphoviridae.	644007	Bacteria; Firmicutes; Bacilli; Lactobacillales; Streptococcaceae; Streptococcus
NC_027396	RefSeq	RVG	G574	33,756	38.6	Streptococcus phage SpSL1	Viruses; dsDNA viruses, no RNA stage; Caudovirales; Siphoviridae.	1566990	Bacteria; Firmicutes; Bacilli; Lactobacillales; Streptococcaceae; Streptococcus
KC348603	EBI	RVG	G575	49,973	40.8	Streptococcus phage phiD12	Viruses; unclassified phages.	1289600	-
DQ394809	EBI	RVG	G576	34,897	35.8	Lactococcus phage ul36.t1k1	Viruses; dsDNA viruses, no RNA stage; Caudovirales; Siphoviridae.	374529	-
DQ394807	EBI	RVG	G576	35,594	35.7	Lactococcus phage ul36.k1t1	Viruses; dsDNA viruses, no RNA stage; Caudovirales; Siphoviridae.	374526	-
DQ394808	EBI	RVG	G576	35,992	36	Lactococcus phage ul36.t1	Viruses; dsDNA viruses, no RNA stage; Caudovirales; Siphoviridae.	374527	-
DQ394810	EBI	RVG	G576	33,641	36	Lactococcus phage phismq86	Viruses; dsDNA viruses, no RNA stage; Caudovirales; Siphoviridae.	444474	-
NC_004746	RefSeq	RVG	G577	36,596	35.4	Lactococcus phage P335 sensu lato	Viruses; dsDNA viruses, no RNA stage; Caudovirales; Siphoviridae.	354260	Bacteria; Firmicutes; Bacilli; Lactobacillales; Streptococcaceae; Lactococcus
NC_002667	RefSeq	RVG	G577	41,834	35.3	Lactococcus phage bIL286	Viruses; dsDNA viruses, no RNA stage; Caudovirales; Siphoviridae.	151536	Bacteria; Firmicutes; Bacilli; Lactobacillales; Streptococcaceae; Lactococcus; Lactococcus lactis
NC_002796	RefSeq	RVG	G577	40,003	35	Lactococcus phage BK5-T	Viruses; dsDNA viruses, no RNA stage; Caudovirales; Siphoviridae.	31754	Bacteria; Firmicutes; Bacilli; Lactobacillales; Streptococcaceae; Lactococcus
NC_002668	RefSeq	RVG	G577	36,949	35.7	Lactococcus phage bIL309	Viruses; dsDNA viruses, no RNA stage; Caudovirales; Siphoviridae.	151537	Bacteria; Firmicutes; Bacilli; Lactobacillales; Streptococcaceae; Lactococcus; Lactococcus lactis
DQ838728	EBI	RVG	G576	33,613	35.5	Lactococcus phage P335	Viruses; dsDNA viruses, no RNA stage; Caudovirales; Siphoviridae.	201846	-
NC_002703	RefSeq	RVG	G576	38,347	36.2	Lactococcus phage Tuc2009	Viruses; dsDNA viruses, no RNA stage; Caudovirales; Siphoviridae.	35241	Bacteria; Firmicutes; Bacilli; Lactobacillales; Streptococcaceae; Lactococcus
NC_002747	RefSeq	RVG	G576	37,667	35.4	Lactococcus phage TP901-1	Viruses; dsDNA viruses, no RNA stage; Caudovirales; Siphoviridae.	35345	Bacteria; Firmicutes; Bacilli; Lactobacillales; Streptococcaceae; Lactococcus
DQ394806	EBI	RVG	G576	37,131	35.7	Lactococcus phage ul36.k1	Viruses; dsDNA viruses, no RNA stage; Caudovirales; Siphoviridae.	374525	-
NC_004066	RefSeq	RVG	G576	36,798	35.8	Lactococcus phage ul36	Viruses; dsDNA viruses, no RNA stage; Caudovirales; Siphoviridae.	114416	Bacteria; Firmicutes; Bacilli; Lactobacillales; Streptococcaceae; Lactococcus
NC_002666	RefSeq	RVG	G576	35,538	35.2	Lactococcus phage bIL285	Viruses; dsDNA viruses, no RNA stage; Caudovirales; Siphoviridae.	151535	Bacteria; Firmicutes; Bacilli; Lactobacillales; Streptococcaceae; Lactococcus; Lactococcus lactis
JX564242	EBI	RVG	G578	31,106	36.2	Lactococcus phage Q33	Viruses; dsDNA viruses, no RNA stage; Caudovirales; Siphoviridae.	254253	-
NC_021861	RefSeq	RVG	G578	30,910	36	Lactococcus phage BM13	Viruses; dsDNA viruses, no RNA stage; Caudovirales; Siphoviridae.	1229751	Bacteria; Firmicutes; Bacilli; Lactobacillales; Streptococcaceae; Lactococcus
NC_013644	RefSeq	RVG	G578	37,856	37.8	Enterococcus phage phiFL4A	Viruses; dsDNA viruses, no RNA stage; Caudovirales; Siphoviridae.	673839	Bacteria; Firmicutes; Bacilli; Lactobacillales; Enterococcaceae; Enterococcus
NC_002669	RefSeq	RVG	G579	14,957	35.9	Lactococcus phage bIL310	Viruses; dsDNA viruses, no RNA stage; Caudovirales; Siphoviridae.	151538	Bacteria; Firmicutes; Bacilli; Lactobacillales; Streptococcaceae; Lactococcus; Lactococcus lactis
NC_002671	RefSeq	RVG	G580	15,179	33	Lactococcus phage bIL312	Viruses; dsDNA viruses, no RNA stage; Caudovirales; Siphoviridae.	151539	Bacteria; Firmicutes; Bacilli; Lactobacillales; Streptococcaceae; Lactococcus; Lactococcus lactis
NC_002670	RefSeq	RVG	G581	14,510	34.2	Lactococcus phage bIL311	Viruses; dsDNA viruses, no RNA stage; Caudovirales; Siphoviridae.	151534	Bacteria; Firmicutes; Bacilli; Lactobacillales; Streptococcaceae; Lactococcus; Lactococcus lactis

NC_022757	RefSeq	RVG	G582	38,880	44.9	Lactobacillus phage PL-1	Viruses; dsDNA viruses, no RNA stage; Caudovirales; Siphoviridae.	39103	Bacteria; Firmicutes; Bacilli; Lactobacillales; Lactobacillaceae; Lactobacillus; Lactobacillus casei group
NC_022756	RefSeq	RVG	G582	40,931	44.8	Lactobacillus phage J-1	Viruses; dsDNA viruses, no RNA stage; Caudovirales; Siphoviridae.	1414736	Bacteria; Firmicutes; Bacilli; Lactobacillales; Lactobacillaceae; Lactobacillus; Lactobacillus casei group
NC_004112	RefSeq	RVG	G582	43,411	44.9	Lactobacillus phage A2	Viruses; dsDNA viruses, no RNA stage; Caudovirales; Siphoviridae.	51369	Bacteria; Firmicutes; Bacilli; Lactobacillales; Lactobacillaceae; Lactobacillus; Lactobacillus casei group
NC_011104	RefSeq	RVG	G582	39,989	45.5	Lactobacillus phage Lrm1	Viruses; dsDNA viruses, no RNA stage; Caudovirales; Siphoviridae.	496874	Bacteria; Firmicutes; Bacilli; Lactobacillales; Lactobacillaceae; Lactobacillus
NC_028911	RefSeq	RVG	G582	34,176	45	Lactobacillus phage iLp1308	Viruses; dsDNA viruses, no RNA stage; Caudovirales; Siphoviridae.	1739611	-
NC_028888	RefSeq	RVG	G582	39,474	44.8	Lactobacillus phage CL1	Viruses; dsDNA viruses, no RNA stage; Caudovirales; Siphoviridae.	1739607	-
NC_028835	RefSeq	RVG	G582	38,751	44.9	Lactobacillus phage CL2	Viruses; dsDNA viruses, no RNA stage; Caudovirales; Siphoviridae.	1739608	-
NC_028783	RefSeq	RVG	G582	39,399	45.1	Lactobacillus phage iLp84	Viruses; dsDNA viruses, no RNA stage; Caudovirales; Siphoviridae.	1739610	-
NC_007501	RefSeq	RVG	G582	36,466	44.3	Lactobacillus phage Lc-Nu	Viruses; dsDNA viruses, no RNA stage; Caudovirales; Siphoviridae.	146269	Bacteria; Firmicutes; Bacilli; Lactobacillales; Lactobacillaceae; Lactobacillus
NC_005893	RefSeq	RVG	G582	39,166	44.6	Lactobacillus phage phiAT3	Viruses; dsDNA viruses, no RNA stage; Caudovirales; Siphoviridae.	279281	Bacteria; Firmicutes; Bacilli; Lactobacillales; Lactobacillaceae; Lactobacillus; Lactobacillus casei group
NC_019486	RefSeq	RVG	G583	42,606	45	Lactobacillus phage LF1	Viruses; dsDNA viruses, no RNA stage; Caudovirales; Siphoviridae.	947980	Bacteria; Firmicutes; Bacilli; Lactobacillales; Lactobacillaceae; Lactobacillus
NC_027982	RefSeq	RVG	G583	32,847	45.5	Lactobacillus phage phiPYB5	Viruses; dsDNA viruses, no RNA stage; Caudovirales; Siphoviridae.	438780	Bacteria; Firmicutes; Bacilli; Lactobacillales; Lactobacillaceae; Lactobacillus
NC_015270	RefSeq	RVG	G584	16,945	34.6	Enterococcus phage EFRM31	Viruses; dsDNA viruses, no RNA stage; Caudovirales; Siphoviridae.	767806	Bacteria; Firmicutes; Bacilli; Lactobacillales; Enterococcaceae; Enterococcus
NC_023551	RefSeq	RVG	G584	40,692	34.6	Enterococcus phage IME-EF4	Viruses; dsDNA viruses, no RNA stage; Caudovirales; Siphoviridae.	1432658	Bacteria; Firmicutes; Bacilli; Lactobacillales; Enterococcaceae; Enterococcus
NC_023595	RefSeq	RVG	G584	41,687	34.6	Enterococcus phage IME_EF3	Viruses; dsDNA viruses, no RNA stage; Caudovirales; Siphoviridae.	1416012	Bacteria; Firmicutes; Bacilli; Lactobacillales; Enterococcaceae; Enterococcus
NC_012419	RefSeq	RVG	G584	21,115	36.7	Enterococcus phage EFAP-1	Viruses; dsDNA viruses, no RNA stage; Caudovirales; Siphoviridae.	627087	Bacteria; Firmicutes; Bacilli; Lactobacillales; Enterococcaceae; Enterococcus
NC_025465	RefSeq	RVG	G584	40,923	34.6	Enterococcus phage EfaCPT1	Viruses; dsDNA viruses, no RNA stage; Caudovirales; Siphoviridae.	1204540	Bacteria; Firmicutes; Bacilli; Lactobacillales; Enterococcaceae; Enterococcus
NC_028990	RefSeq	RVG	G584	38,886	35	Enterococcus phage vB_IME196	Viruses; dsDNA viruses, no RNA stage; Caudovirales; Siphoviridae.	1747289	-
NC_028826	RefSeq	RVG	G584	42,265	35.5	Enterococcus phage IME-EFm5	Viruses; dsDNA viruses, no RNA stage; Caudovirales; Siphoviridae.	1718158	-
NC_024356	RefSeq	RVG	G584	42,597	35.2	Enterococcus phage IME-EFm1	Viruses; dsDNA viruses, no RNA stage; Caudovirales; Siphoviridae.	1445858	Bacteria; Firmicutes; Bacilli; Lactobacillales; Enterococcaceae; Enterococcus
JX486088	EBI	RVG	G585	80,617	37	Lactobacillus phage ATCC 8014-B2	Viruses; dsDNA viruses, no RNA stage; Caudovirales; Siphoviridae.	1225795	-
NC_026609	RefSeq	RVG	G586	74,806	37.8	Lactobacillus phage Ldl1	Viruses; dsDNA viruses, no RNA stage; Caudovirales; Siphoviridae.	1552735	Bacteria; Firmicutes; Bacilli; Lactobacillales; Lactobacillaceae; Lactobacillus; Lactobacillus delbrueckii
NC_019489	RefSeq	RVG	G587	41,726	40.6	Lactobacillus phage Sha1	Viruses; dsDNA viruses, no RNA stage; Caudovirales; Siphoviridae.	947981	Bacteria; Firmicutes; Bacilli; Lactobacillales; Lactobacillaceae; Lactobacillus
NC_009554	RefSeq	RVG	G588	34,659	47.8	Lactobacillus phage LL-H	Viruses; dsDNA viruses, no RNA stage; Caudovirales; Siphoviridae.	12348	Bacteria; Firmicutes; Bacilli; Lactobacillales; Lactobacillaceae; Lactobacillus; Lactobacillus delbrueckii
NC_022775	RefSeq	RVG	G588	36,969	47.7	Lactobacillus phage phiJB	Viruses; dsDNA viruses, no RNA stage; Caudovirales; Siphoviridae.	1399941	Bacteria; Firmicutes; Bacilli; Lactobacillales; Lactobacillaceae; Lactobacillus
NC_019456	RefSeq	RVG	G589	49,433	44.2	Lactobacillus phage JCL1032	Viruses; dsDNA viruses, no RNA stage; Caudovirales; Siphoviridae.	37105	Bacteria; Firmicutes; Bacilli; Lactobacillales; Lactobacillaceae; Lactobacillus; Lactobacillus delbrueckii
NC_012884	RefSeq	RVG	G590	31,147	39.2	Streptococcus phage M102	Viruses; dsDNA viruses, no RNA stage; Caudovirales; Siphoviridae.	372457	Bacteria; Firmicutes; Bacilli; Lactobacillales; Streptococcaceae; Streptococcus
NC_028984	RefSeq	RVG	G590	30,664	39.6	Streptococcus phage M102AD	Viruses; dsDNA viruses, no RNA stage; Caudovirales; Siphoviridae.	1587907	-
NC_029030	RefSeq	RVG	G590	31,075	39	Streptococcus phage APCM01	Viruses; dsDNA viruses, no RNA stage; Caudovirales; Siphoviridae.	1647391	-
NC_011801	RefSeq	RVG	G591	38,934	37	Lactobacillus phage Lv-1	Viruses; dsDNA viruses, no RNA stage; Caudovirales; Siphoviridae.	578234	Bacteria; Firmicutes; Bacilli; Lactobacillales; Lactobacillaceae; Lactobacillus
NC_000896	RefSeq	RVG	G592	43,785	35.6	Lactobacillus phage phiadh	Viruses; dsDNA viruses, no RNA stage; Caudovirales; Siphoviridae.	12417	Bacteria; Firmicutes; Bacilli; Lactobacillales; Lactobacillaceae; Lactobacillus
NC_025421	RefSeq	RVG	G593	29,616	41.6	Lactobacillus phage Ld3	Viruses; dsDNA viruses, no RNA stage; Caudovirales; Siphoviridae.	1500735	Bacteria; Firmicutes; Bacilli; Lactobacillales; Lactobacillaceae; Lactobacillus
NC_025420	RefSeq	RVG	G593	32,975	41.5	Lactobacillus phage Ld17	Viruses; dsDNA viruses, no RNA stage; Caudovirales; Siphoviridae.	1500733	Bacteria; Firmicutes; Bacilli; Lactobacillales; Lactobacillaceae; Lactobacillus
NC_025415	RefSeq	RVG	G593	32,799	41.7	Lactobacillus phage Ld25A	Viruses; dsDNA viruses, no RNA stage; Caudovirales; Siphoviridae.	1500734	Bacteria; Firmicutes; Bacilli; Lactobacillales; Lactobacillaceae; Lactobacillus
NC_019449	RefSeq	RVG	G593	31,841	42	Lactobacillus phage c5	Viruses; dsDNA viruses, no RNA stage; Caudovirales; Siphoviridae; C5likevirus.	508419	Bacteria; Firmicutes; Bacilli; Lactobacillales; Lactobacillaceae; Lactobacillus; Lactobacillus delbrueckii
NC_022989	RefSeq	RVG	G593	31,080	41.6	Lactobacillus phage LLKu	Viruses; dsDNA viruses, no RNA stage; Caudovirales; Siphoviridae; C5likevirus.	297254	Bacteria; Firmicutes; Bacilli; Lactobacillales; Lactobacillaceae; Lactobacillus; Lactobacillus delbrueckii
NC_022762	RefSeq	RVG	G593	33,996	42	Lactobacillus phage phiLdb	Viruses; dsDNA viruses, no RNA stage; Caudovirales; Siphoviridae.	1399942	Bacteria; Firmicutes; Bacilli; Lactobacillales; Lactobacillaceae; Lactobacillus
GQ478083	EBI	RVG	G594	38,721	34	Enterococcus phage phiFL1C	Viruses; dsDNA viruses, no RNA stage; Caudovirales; Siphoviridae; Phifllikevirus.	673834	-
GQ478082	EBI	RVG	G594	38,989	34	Enterococcus phage phiFL1B	Viruses; dsDNA viruses, no RNA stage; Caudovirales; Siphoviridae; Phifllikevirus.	673833	-
NC_013646	RefSeq	RVG	G594	38,764	34	Enterococcus phage phiFL1A	Viruses; dsDNA viruses, no RNA stage; Caudovirales; Siphoviridae; Phifllikevirus.	673832	Bacteria; Firmicutes; Bacilli; Lactobacillales; Enterococcaceae; Enterococcus
GQ478085	EBI	RVG	G594	36,826	34.6	Enterococcus phage phiFL2B	Viruses; dsDNA viruses, no RNA stage; Caudovirales; Siphoviridae; Phifllikevirus.	673836	-
NC_013643	RefSeq	RVG	G594	36,270	34.6	Enterococcus phage phiFL2A	Viruses; dsDNA viruses, no RNA stage; Caudovirales; Siphoviridae; Phifllikevirus.	673835	Bacteria; Firmicutes; Bacilli; Lactobacillales; Enterococcaceae; Enterococcus
GQ478087	EBI	RVG	G594	40,275	34.5	Enterococcus phage phiFL3B	Viruses; dsDNA viruses, no RNA stage; Caudovirales; Siphoviridae; Phifllikevirus.	673838	-
NC_013648	RefSeq	RVG	G594	39,576	34.5	Enterococcus phage phiFL3A	Viruses; dsDNA viruses, no RNA stage; Caudovirales; Siphoviridae; Phifllikevirus.	673837	Bacteria; Firmicutes; Bacilli; Lactobacillales; Enterococcaceae; Enterococcus
NC_013696	RefSeq	RVG	G595	42,822	34.4	Enterococcus phage phiEf11	Viruses; dsDNA viruses, no RNA stage; Caudovirales; Siphoviridae.	663241	Bacteria; Firmicutes; Bacilli; Lactobacillales; Enterococcaceae; Enterococcus
NC_028671	RefSeq	RVG	G595	41,307	34	Enterococcus phage vB_IME197	Viruses; dsDNA viruses, no RNA stage; Caudovirales; Siphoviridae.	1747326	-
NC_025453	RefSeq	RVG	G595	40,286	35	Enterococcus phage EFC-1	Viruses; dsDNA viruses, no RNA stage; Caudovirales; Siphoviridae.	1486428	Bacteria; Firmicutes; Bacilli; Lactobacillales; Enterococcaceae; Enterococcus
NC_023571	RefSeq	RVG	G596	46,243	38.6	Oenococcus phage phiS11	Viruses; dsDNA viruses, no RNA stage; unclassified dsDNA phages.	1432847	Bacteria; Firmicutes; Bacilli; Lactobacillales; Leuconostocaceae; Oenococcus
NC_023560	RefSeq	RVG	G596	43,454	39	Oenococcus phage phiS13	Viruses; dsDNA viruses, no RNA stage; unclassified dsDNA phages.	1432848	Bacteria; Firmicutes; Bacilli; Lactobacillales; Leuconostocaceae; Oenococcus
NC_023559	RefSeq	RVG	G596	46,145	38.9	Oenococcus phage phi9805	Viruses; dsDNA viruses, no RNA stage; unclassified dsDNA phages.	1435411	Bacteria; Firmicutes; Bacilli; Lactobacillales; Leuconostocaceae; Oenococcus
NC_005355	RefSeq	RVG	G596	40,190	35.1	Lactobacillus prophage Lj965	Viruses; dsDNA viruses, no RNA stage; Caudovirales; Siphoviridae.	139870	Bacteria; Firmicutes; Bacilli; Lactobacillales; Lactobacillaceae; Lactobacillus
NC_015254	RefSeq	RVG	G597	41,518	36.4	Brochothrix phage BL3	Viruses; dsDNA viruses, no RNA stage; Caudovirales; Siphoviridae.	764562	Bacteria; Firmicutes; Bacilli; Bacillales; Listeriaceae; Brochothrix
NC_015252	RefSeq	RVG	G597	36,953	37.3	Brochothrix phage NF5	Viruses; dsDNA viruses, no RNA stage; Caudovirales; Siphoviridae.	764561	Bacteria; Firmicutes; Bacilli; Bacillales; Listeriaceae; Brochothrix
NC_019916	RefSeq	RVG	G598	38,002	47.6	Lactobacillus phage ATCC8014	Viruses; dsDNA viruses, no RNA stage; Caudovirales; Siphoviridae; Phijlunalikevirus.	1225794	Bacteria; Firmicutes; Bacilli; Lactobacillales; Lactobacillaceae; Lactobacillus
NC_016161	RefSeq	RVG	G598	38,013	47.6	Pediococcus phage clP1	Viruses; dsDNA viruses, no RNA stage; Caudovirales; Siphoviridae; Phijlunalikevirus.	1108070	Bacteria; Firmicutes; Bacilli; Lactobacillales; Lactobacillaceae; Pediococcus

NC_006936	RefSeq	RVG	G598	36,674	39.4	Lactobacillus phage phijl1	Viruses; dsDNA viruses, no RNA stage; Caudovirales; Siphoviridae; Phijlunalikevirus.	235201	Bacteria; Firmicutes; Bacilli; Lactobacillales; Lactobacillaceae; Lactobacillus
NC_005354	RefSeq	RVG	G599	38,384	34.7	Lactobacillus prophage Lj928	Viruses; dsDNA viruses, no RNA stage; Caudovirales; Siphoviridae.	139872	Bacteria; Firmicutes; Bacilli; Lactobacillales; Lactobacillaceae; Lactobacillus
NC_029068	RefSeq	RVG	G600	31,703	48	Lactobacillus phage LfeSau	Viruses; dsDNA viruses, no RNA stage; Caudovirales; Siphoviridae.	1567453	-
NC_028929	RefSeq	RVG	G601	40,759	36.9	Listeria phage vB_LmoS_293	Viruses; dsDNA viruses, no RNA stage; Caudovirales; Siphoviridae.	1591073	-
NC_024384	RefSeq	RVG	G601	41,156	36.6	Listeria phage LP-030-3	Viruses; dsDNA viruses, no RNA stage; Caudovirales; Siphoviridae.	1458852	Bacteria; Firmicutes; Bacilli; Bacillales; Listeriaceae; Listeria
NC_009810	RefSeq	RVG	G601	38,867	36.7	Listeria phage A500	Viruses; dsDNA viruses, no RNA stage; Caudovirales; Siphoviridae.	40522	Bacteria; Firmicutes; Bacilli; Bacillales; Listeriaceae; Listeria
NC_003216	RefSeq	RVG	G601	40,834	36.1	Listeria phage A118	Viruses; dsDNA viruses, no RNA stage; Caudovirales; Siphoviridae.	40521	Bacteria; Firmicutes; Bacilli; Bacillales; Listeriaceae; Listeria
NC_009815	RefSeq	RVG	G601	38,124	35.5	Listeria phage A006	Viruses; dsDNA viruses, no RNA stage; Caudovirales; Siphoviridae.	330399	Bacteria; Firmicutes; Bacilli; Bacillales; Listeriaceae; Listeria
NC_028871	RefSeq	RVG	G601	38,392	35.9	Listeria phage vB_LmoS_188	Viruses; dsDNA viruses, no RNA stage; Caudovirales; Siphoviridae.	1591072	-
AJ312240	EBI	RVG	G602	37,618	34.7	Listeria phage PSA	Viruses; dsDNA viruses, no RNA stage; Caudovirales; Siphoviridae.	171618	-
NC_003291	RefSeq	RVG	G602	37,618	34.7	Listeria phage 2389	Viruses; dsDNA viruses, no RNA stage; Caudovirales; Siphoviridae.	181007	Bacteria; Firmicutes; Bacilli; Bacillales; Listeriaceae; Listeria
NC_021539	RefSeq	RVG	G602	38,275	34.8	Listeria phage LP-030-2	Viruses; dsDNA viruses, no RNA stage; Caudovirales; Siphoviridae.	1173743	Bacteria; Firmicutes; Bacilli; Bacillales; Listeriaceae; Listeria
NC_009812	RefSeq	RVG	G602	42,653	35.1	Listeria phage B025	Viruses; dsDNA viruses, no RNA stage; Caudovirales; Siphoviridae.	330396	Bacteria; Firmicutes; Bacilli; Bacillales; Listeriaceae; Listeria
NC_024387	RefSeq	RVG	G602	43,767	35.5	Listeria phage LP-101	Viruses; dsDNA viruses, no RNA stage; Caudovirales; Siphoviridae.	1458856	Bacteria; Firmicutes; Bacilli; Bacillales; Listeriaceae; Listeria
NC_006557	RefSeq	RVG	G603	41,092	41.7	Bacillus phage BCJA1c	Viruses; dsDNA viruses, no RNA stage; Caudovirales; Siphoviridae.	294382	Bacteria; Firmicutes; Bacilli; Bacillales; Sporolactobacillaceae
NC_004305	RefSeq	RVG	G604	42,259	43.1	Lactobacillus phage phig1e	Viruses; dsDNA viruses, no RNA stage; Caudovirales; Siphoviridae.	52979	Bacteria; Firmicutes; Bacilli; Lactobacillales; Lactobacillaceae
NC_007060	RefSeq	RVG	G605	41,902	35.7	Staphylococcus phage 55	Viruses; dsDNA viruses, no RNA stage; Caudovirales; Siphoviridae; Phietalikevirus.	320845	Bacteria; Firmicutes; Bacilli; Bacillales; Staphylococcaceae; Staphylococcus
NC_003288	RefSeq	RVG	G605	43,081	35.4	Staphylococcus phage phiETA	Viruses; dsDNA viruses, no RNA stage; Caudovirales; Siphoviridae; Phietalikevirus.	106284	Bacteria; Firmicutes; Bacilli; Bacillales; Staphylococcaceae; Staphylococcus
NC_023500	RefSeq	RVG	G605	43,301	35.1	Staphylococcus phage StauST398-5	Viruses; dsDNA viruses, no RNA stage; Caudovirales; Siphoviridae.	1308897	Bacteria; Firmicutes; Bacilli; Bacillales; Staphylococcaceae; Staphylococcus
NC_021326	RefSeq	RVG	G605	45,242	34.5	Staphylococcus phage StauST398-1	Viruses; dsDNA viruses, no RNA stage; Caudovirales; Siphoviridae.	1195068	Bacteria; Firmicutes; Bacilli; Bacillales; Staphylococcaceae; Staphylococcus
NC_010147	RefSeq	RVG	G605	43,011	35.6	Staphylococcus phage phiMR11	Viruses; dsDNA viruses, no RNA stage; Caudovirales; Siphoviridae; Phietalikevirus.	379501	Bacteria; Firmicutes; Bacilli; Bacillales; Staphylococcaceae; Staphylococcus
DQ908929	EBI	RVG	G605	42,140	35.6	Staphylococcus phage 80	Viruses; dsDNA viruses, no RNA stage; Caudovirales; Siphoviridae; Phietalikevirus.	405947	-
NC_007062	RefSeq	RVG	G605	41,690	35.5	Staphylococcus phage 52A	Viruses; dsDNA viruses, no RNA stage; Caudovirales; Siphoviridae; Phietalikevirus.	320847	Bacteria; Firmicutes; Bacilli; Bacillales; Staphylococcaceae; Staphylococcus
NC_007061	RefSeq	RVG	G605	42,802	35.4	Staphylococcus phage 29	Viruses; dsDNA viruses, no RNA stage; Caudovirales; Siphoviridae; Phietalikevirus.	320846	Bacteria; Firmicutes; Bacilli; Bacillales; Staphylococcaceae; Staphylococcus
NC_007064	RefSeq	RVG	G605	42,431	35.7	Staphylococcus phage 92	Viruses; dsDNA viruses, no RNA stage; Caudovirales; Siphoviridae; Phietalikevirus.	320849	Bacteria; Firmicutes; Bacilli; Bacillales; Staphylococcaceae; Staphylococcus
NC_007063	RefSeq	RVG	G605	43,231	35.5	Staphylococcus phage 88	Viruses; dsDNA viruses, no RNA stage; Caudovirales; Siphoviridae; Phietalikevirus.	320848	Bacteria; Firmicutes; Bacilli; Bacillales; Staphylococcaceae; Staphylococcus
NC_021863	RefSeq	RVG	G605	42,652	35.4	Staphylococcus phage SA13	Viruses; dsDNA viruses, no RNA stage; Caudovirales; Siphoviridae.	1195086	Bacteria; Firmicutes; Bacilli; Bacillales; Staphylococcaceae; Staphylococcus
NC_028915	RefSeq	RVG	G605	43,228	35.6	Staphylococcus phage B236	Viruses; dsDNA viruses, no RNA stage; Caudovirales; Siphoviridae; Phietalikevirus; unclassified Phietalikevirus.	1636205	-
NC_007059	RefSeq	RVG	G605	43,114	35.2	Staphylococcus phage 71	Viruses; dsDNA viruses, no RNA stage; Caudovirales; Siphoviridae; Phietalikevirus.	320844	Bacteria; Firmicutes; Bacilli; Bacillales; Staphylococcaceae; Staphylococcus
NC_007065	RefSeq	RVG	G605	43,440	36	Staphylococcus phage X2	Viruses; dsDNA viruses, no RNA stage; Caudovirales; Siphoviridae; Phietalikevirus.	320850	Bacteria; Firmicutes; Bacilli; Bacillales; Staphylococcaceae; Staphylococcus
NC_021332	RefSeq	RVG	G605	41,392	35.6	Staphylococcus phage StauST398-3	Viruses; dsDNA viruses, no RNA stage; Caudovirales; Siphoviridae.	1195071	Bacteria; Firmicutes; Bacilli; Bacillales; Staphylococcaceae; Staphylococcus
NC_007057	RefSeq	RVG	G605	43,576	35	Staphylococcus phage 96	Viruses; dsDNA viruses, no RNA stage; Caudovirales; Siphoviridae; Phietalikevirus.	320842	Bacteria; Firmicutes; Bacilli; Bacillales; Staphylococcaceae; Staphylococcus
NC_024391	RefSeq	RVG	G605	41,941	36	Staphylococcus phage DW2	Viruses; dsDNA viruses, no RNA stage; Caudovirales; Siphoviridae.	1464127	Bacteria; Firmicutes; Bacilli; Bacillales; Staphylococcaceae; Staphylococcus
NC_008799	RefSeq	RVG	G605	43,282	34.9	Staphylococcus phage phiETA3	Viruses; dsDNA viruses, no RNA stage; Caudovirales; Siphoviridae; Phietalikevirus.	326037	Bacteria; Firmicutes; Bacilli; Bacillales; Staphylococcaceae; Staphylococcus
NC_028917	RefSeq	RVG	G605	41,931	35.4	Staphylococcus phage 3MRA	Viruses; dsDNA viruses, no RNA stage; Caudovirales; Siphoviridae.	1505026	-
NC_028859	RefSeq	RVG	G605	42,881	34.8	Staphylococcus phage B166	Viruses; dsDNA viruses, no RNA stage; Caudovirales; Siphoviridae; Phietalikevirus; unclassified Phietalikevirus.	1636204	-
KP209285	EBI	RVG	G605	40,365	35.1	Staphylococcus phage phiNM4-gamma4	Viruses; dsDNA viruses, no RNA stage; Caudovirales; Siphoviridae; Phietalikevirus.	1589269	-
NC_028864	RefSeq	RVG	G605	43,189	34.7	Staphylococcus phage phinm4	Viruses; dsDNA viruses, no RNA stage; Caudovirales; Siphoviridae; Phietalikevirus.	387910	-
NC_028669	RefSeq	RVG	G605	43,012	34.7	Staphylococcus phage phiJB	Viruses; dsDNA viruses, no RNA stage; Caudovirales; Siphoviridae; Phietalikevirus; unclassified Phietalikevirus.	1698421	-
NC_007058	RefSeq	RVG	G605	43,155	35.1	Staphylococcus phage ROSA	Viruses; dsDNA viruses, no RNA stage; Caudovirales; Siphoviridae.	320843	Bacteria; Firmicutes; Bacilli; Bacillales; Staphylococcaceae; Staphylococcus
NC_009526	RefSeq	RVG	G605	43,864	34.1	Staphylococcus phage 80alpha	Viruses; dsDNA viruses, no RNA stage; Caudovirales; Siphoviridae; Phietalikevirus.	53369	Bacteria; Firmicutes; Bacilli; Bacillales; Staphylococcaceae; Staphylococcus
NC_007049	RefSeq	RVG	G605	43,883	34.1	Staphylococcus phage 53	Viruses; dsDNA viruses, no RNA stage; Caudovirales; Siphoviridae; Phietalikevirus.	320835	Bacteria; Firmicutes; Bacilli; Bacillales; Staphylococcaceae; Staphylococcus
NC_008583	RefSeq	RVG	G605	43,128	34.2	Staphylococcus phage phiNM	Viruses; dsDNA viruses, no RNA stage; Caudovirales; Siphoviridae.	387905	Bacteria; Firmicutes; Bacilli; Bacillales; Staphylococcaceae; Staphylococcus
NC_028913	RefSeq	RVG	G605	43,145	34.6	Staphylococcus phage phinm2	Viruses; dsDNA viruses, no RNA stage; Caudovirales; Siphoviridae; Phietalikevirus.	387908	-
NC_010808	RefSeq	RVG	G605	44,342	34.3	Staphylococcus phage phiMR25	Viruses; dsDNA viruses, no RNA stage; Caudovirales; Siphoviridae; Phietalikevirus.	487152	Bacteria; Firmicutes; Bacilli; Bacillales; Staphylococcaceae; Staphylococcus
NC_007050	RefSeq	RVG	G605	44,283	34.6	Staphylococcus phage 85	Viruses; dsDNA viruses, no RNA stage; Caudovirales; Siphoviridae; Phietalikevirus.	59506	Bacteria; Firmicutes; Bacilli; Bacillales; Staphylococcaceae; Staphylococcus
NC_008798	RefSeq	RVG	G605	43,265	34.3	Staphylococcus phage phiETA2	Viruses; dsDNA viruses, no RNA stage; Caudovirales; Siphoviridae; Phietalikevirus.	326036	Bacteria; Firmicutes; Bacilli; Bacillales; Staphylococcaceae; Staphylococcus
NC_029010	RefSeq	RVG	G605	40,592	34.2	Staphylococcus phage SA97	Viruses; dsDNA viruses, no RNA stage; Caudovirales; Siphoviridae.	1498171	-
NC_007048	RefSeq	RVG	G605	42,732	34.3	Staphylococcus phage 69	Viruses; dsDNA viruses, no RNA stage; Caudovirales; Siphoviridae; Phietalikevirus.	320834	Bacteria; Firmicutes; Bacilli; Bacillales; Staphylococcaceae; Staphylococcus

NC_004615	RefSeq	RVG	G605	43,604	34.5	Staphylococcus phage 11	Viruses; dsDNA viruses, no RNA stage; Caudovirales; Siphoviridae; Phietalikevirus.	12360	Bacteria; Firmicutes; Bacilli; Bacillales; Staphylococcaceae; Staphylococcus; Staphylococcus aureus
NC_014460	RefSeq	RVG	G605	41,207	34	Staphylococcus phage Sap26	Viruses; dsDNA viruses, no RNA stage; Caudovirales; Siphoviridae; Phietalikevirus.	872294	Bacteria; Firmicutes; Bacilli; Bacillales; Staphylococcaceae; Staphylococcus
JX274647	EBI	RVG	G605	42,902	34.5	Staphylococcus phage SP6	Viruses; dsDNA viruses, no RNA stage; Caudovirales; Siphoviridae.	1211286	-
NC_021801	RefSeq	RVG	G605	42,902	34.5	Staphylococcus phage SA12	Viruses; dsDNA viruses, no RNA stage; Caudovirales; Siphoviridae.	1347760	Bacteria; Firmicutes; Bacilli; Bacillales; Staphylococcaceae; Staphylococcus
JX274646	EBI	RVG	G605	43,305	34.4	Staphylococcus phage SP5	Viruses; dsDNA viruses, no RNA stage; Caudovirales; Siphoviridae.	1211285	-
NC_017968	RefSeq	RVG	G605	43,786	34	Staphylococcus phage TEM123	Viruses; dsDNA viruses, no RNA stage; Caudovirales; Siphoviridae.	1168613	Bacteria; Firmicutes; Bacilli; Bacillales; Staphylococcaceae; Staphylococcus
NC_011614	RefSeq	RVG	G605	42,526	34.9	Staphylococcus phage Ipla88	Viruses; dsDNA viruses, no RNA stage; Caudovirales; Siphoviridae; Phietalikevirus.	575608	Bacteria; Firmicutes; Bacilli; Bacillales; Staphylococcaceae; Staphylococcus
NC_007047	RefSeq	RVG	G605	39,620	34.3	Staphylococcus phage 187	Viruses; dsDNA viruses, no RNA stage; Caudovirales; Siphoviridae; Phietalikevirus.	55511	Bacteria; Firmicutes; Bacilli; Bacillales; Staphylococcaceae; Staphylococcus
NC_008723	RefSeq	RVG	G605	44,041	34.9	Staphylococcus phage PH15	Viruses; dsDNA viruses, no RNA stage; Caudovirales; Siphoviridae; Phietalikevirus.	399185	Bacteria; Firmicutes; Bacilli; Bacillales; Staphylococcaceae; Staphylococcus
NC_018284	RefSeq	RVG	G605	42,123	34.8	Staphylococcus phage Ipla7	Viruses; dsDNA viruses, no RNA stage; Caudovirales; Siphoviridae; Phietalikevirus.	1197953	Bacteria; Firmicutes; Bacilli; Bacillales; Staphylococcaceae; Staphylococcus
NC_008722	RefSeq	RVG	G605	43,420	34.7	Staphylococcus phage CNPH82	Viruses; dsDNA viruses, no RNA stage; Caudovirales; Siphoviridae; Phietalikevirus.	398839	Bacteria; Firmicutes; Bacilli; Bacillales; Staphylococcaceae; Staphylococcus
NC_018281	RefSeq	RVG	G605	43,581	34.7	Staphylococcus phage Ipla5	Viruses; dsDNA viruses, no RNA stage; Caudovirales; Siphoviridae; Phietalikevirus.	1197952	Bacteria; Firmicutes; Bacilli; Bacillales; Staphylococcaceae; Staphylococcus
NC_007056	RefSeq	RVG	G605	45,286	36	Staphylococcus phage EW	Viruses; dsDNA viruses, no RNA stage; Caudovirales; Siphoviridae; Phietalikevirus.	320841	Bacteria; Firmicutes; Bacilli; Bacillales; Staphylococcaceae; Staphylococcus
NC_007055	RefSeq	RVG	G605	43,681	35.1	Staphylococcus phage 37	Viruses; dsDNA viruses, no RNA stage; Caudovirales; Siphoviridae; Phietalikevirus.	320840	Bacteria; Firmicutes; Bacilli; Bacillales; Staphylococcaceae; Staphylococcus
NC_019914	RefSeq	RVG	G605	40,071	33.2	Staphylococcus phage StB27	Viruses; dsDNA viruses, no RNA stage; Caudovirales; Siphoviridae.	1147044	Bacteria; Firmicutes; Bacilli; Bacillales; Staphylococcaceae; Staphylococcus
NC_020490	RefSeq	RVG	G605	44,714	34.2	Staphylococcus phage StB12	Viruses; dsDNA viruses, no RNA stage; Caudovirales; Siphoviridae.	1147042	Bacteria; Firmicutes; Bacilli; Bacillales; Staphylococcaceae; Staphylococcus
AP011956	EBI	RVG	G606	42,481	33.3	Staphylococcus phage phi7247PVL	Viruses; dsDNA viruses, no RNA stage; Caudovirales; Siphoviridae.	874268	-
NC_019921	RefSeq	RVG	G606	42,461	33.3	Staphylococcus phage phi5967PVL	Viruses; dsDNA viruses, no RNA stage; Caudovirales; Siphoviridae.	874267	Bacteria; Firmicutes; Bacilli; Bacillales; Staphylococcaceae; Staphylococcus
NC_025460	RefSeq	RVG	G606	42,600	33.4	Staphylococcus phage phiSa119	Viruses; dsDNA viruses, no RNA stage; Caudovirales; Siphoviridae.	1498220	Bacteria; Firmicutes; Bacilli; Bacillales; Staphylococcaceae; Staphylococcus
NC_005356	RefSeq	RVG	G606	41,708	33.5	Staphylococcus phage 77	Viruses; dsDNA viruses, no RNA stage; Caudovirales; Siphoviridae; 77likevirus.	259901	Bacteria; Firmicutes; Bacilli; Bacillales; Staphylococcaceae; Staphylococcus
NC_013195	RefSeq	RVG	G606	40,761	34	Staphylococcus phage P954	Viruses; dsDNA viruses, no RNA stage; Caudovirales; Siphoviridae.	668618	Bacteria; Firmicutes; Bacilli; Bacillales; Staphylococcaceae; Staphylococcus
NC_008617	RefSeq	RVG	G606	44,061	33	Staphylococcus phage phiNM3	Viruses; dsDNA viruses, no RNA stage; Caudovirales; Siphoviridae.	387909	Bacteria; Firmicutes; Bacilli; Bacillales; Staphylococcaceae; Staphylococcus
NC_028775	RefSeq	RVG	G606	43,098	32.9	Staphylococcus phage 23MRA	Viruses; dsDNA viruses, no RNA stage; Caudovirales; Siphoviridae.	1505027	-
NC_004740	RefSeq	RVG	G606	44,082	32.8	Staphylococcus phage phiN315	Viruses; dsDNA viruses, no RNA stage; Caudovirales; Siphoviridae.	225979	Bacteria; Firmicutes; Bacilli; Bacillales; Staphylococcaceae; Staphylococcus; Staphylococcus aureus
NC_023499	RefSeq	RVG	G606	42,906	33.1	Staphylococcus phage StauST398-4	Viruses; dsDNA viruses, no RNA stage; Caudovirales; Siphoviridae.	1308896	Bacteria; Firmicutes; Bacilli; Bacillales; Staphylococcaceae; Staphylococcus
NC_026016	RefSeq	RVG	G606	43,748	33	Staphylococcus phage phiBU01	Viruses; dsDNA viruses, no RNA stage; Caudovirales; Siphoviridae.	1519999	Bacteria; Firmicutes; Bacilli; Bacillales; Staphylococcaceae; Staphylococcus
NC_012784	RefSeq	RVG	G606	44,492	33.6	Staphylococcus phage phiPVL-CN125	Viruses; dsDNA viruses, no RNA stage; unclassified dsDNA phages.	648017	Bacteria; Firmicutes; Bacilli; Bacillales; Staphylococcaceae; Staphylococcus
NC_008689	RefSeq	RVG	G606	44,857	33.5	Staphylococcus phage Pvl108	Viruses; dsDNA viruses, no RNA stage; Caudovirales; Siphoviridae; 77likevirus.	360398	Bacteria; Firmicutes; Bacilli; Bacillales; Staphylococcaceae; Staphylococcus
NC_002321	RefSeq	RVG	G606	41,401	33.6	Staphylococcus phage PVL	Viruses; dsDNA viruses, no RNA stage; Caudovirales; Siphoviridae.	71366	Bacteria; Firmicutes; Bacilli; Bacillales; Staphylococcaceae; Staphylococcus
NC_009761	RefSeq	RVG	G606	42,232	33.5	Staphylococcus phage tp310-1	Viruses; unclassified phages.	445515	Bacteria; Firmicutes; Bacilli; Bacillales; Staphylococcaceae
NC_004617	RefSeq	RVG	G606	42,722	33.5	Staphylococcus phage 13	Viruses; dsDNA viruses, no RNA stage; Caudovirales; Siphoviridae; 77likevirus.	186153	Bacteria; Firmicutes; Bacilli; Bacillales; Staphylococcaceae; Staphylococcus; Staphylococcus aureus
NC_009763	RefSeq	RVG	G606	42,973	33.5	Staphylococcus phage tp310-3	Viruses; unclassified phages.	445517	Bacteria; Firmicutes; Bacilli; Bacillales; Staphylococcaceae
NC_021773	RefSeq	RVG	G606	43,458	33.3	Staphylococcus phage JS01	Viruses; dsDNA viruses, no RNA stage; Caudovirales; Siphoviridae.	1273712	Bacteria; Firmicutes; Bacilli; Bacillales; Staphylococcaceae; Staphylococcus
NC_002486	RefSeq	RVG	G606	45,636	33.5	Staphylococcus prophage phiPV83	Viruses; dsDNA viruses, no RNA stage; Caudovirales; Siphoviridae.	129009	Bacteria; Firmicutes; Bacilli; Bacillales; Staphylococcaceae; Staphylococcus
NC_007051	RefSeq	RVG	G607	41,318	36.9	Staphylococcus phage 2638A	Viruses; dsDNA viruses, no RNA stage; Caudovirales; Siphoviridae.	320836	Bacteria; Firmicutes; Bacilli; Bacillales; Staphylococcaceae; Staphylococcus
NC_007053	RefSeq	RVG	G607	43,095	33.5	Staphylococcus phage 3A	Viruses; dsDNA viruses, no RNA stage; Caudovirales; Siphoviridae; 3alikevirus.	215167	Bacteria; Firmicutes; Bacilli; Bacillales; Staphylococcaceae; Staphylococcus
NC_009762	RefSeq	RVG	G607	47,785	33.8	Staphylococcus phage tp310-2	Viruses; dsDNA viruses, no RNA stage; Caudovirales; Siphoviridae.	445516	Bacteria; Firmicutes; Bacilli; Bacillales; Staphylococcaceae
NC_004616	RefSeq	RVG	G607	44,970	33.3	Staphylococcus phage Phi12	Viruses; dsDNA viruses, no RNA stage; Caudovirales; Siphoviridae; 3alikevirus.	186152	Bacteria; Firmicutes; Bacilli; Bacillales; Staphylococcaceae; Staphylococcus; Staphylococcus aureus
NC_007054	RefSeq	RVG	G607	44,777	33.5	Staphylococcus phage 47	Viruses; dsDNA viruses, no RNA stage; Caudovirales; Siphoviridae; 3alikevirus.	320839	Bacteria; Firmicutes; Bacilli; Bacillales; Staphylococcaceae; Staphylococcus
NC_022758	RefSeq	RVG	G607	44,459	33.4	Staphylococcus phage YMC/09/04/R1988	Viruses; dsDNA viruses, no RNA stage; Caudovirales; Siphoviridae.	1399949	Bacteria; Firmicutes; Bacilli; Bacillales; Staphylococcaceae; Staphylococcus
JX174275	EBI	RVG	G607	46,048	33.2	Staphylococcus phage LH1	Viruses; dsDNA viruses, no RNA stage; Caudovirales; Siphoviridae.	1296641	-
NC_011344	RefSeq	RVG	G607	47,342	33	Staphylococcus phage phi2958PVL	Viruses; unclassified phages.	430796	Bacteria; Firmicutes; Bacilli; Bacillales; Staphylococcaceae; Staphylococcus
NC_020199	RefSeq	RVG	G607	47,252	33	Staphylococcus phage phi7401PVL	Viruses; dsDNA viruses, no RNA stage; Caudovirales; Siphoviridae.	1165702	Bacteria; Firmicutes; Bacilli; Bacillales; Staphylococcaceae; Staphylococcus
NC_011612	RefSeq	RVG	G607	45,344	33.2	Staphylococcus phage Ipla35	Viruses; dsDNA viruses, no RNA stage; Caudovirales; Siphoviridae; 3alikevirus.	575610	Bacteria; Firmicutes; Bacilli; Bacillales; Staphylococcaceae; Staphylococcus
NC_019513	RefSeq	RVG	G607	45,552	33.4	Staphylococcus phage SMSAP5	Viruses; dsDNA viruses, no RNA stage; Caudovirales; Siphoviridae.	1168612	Bacteria; Firmicutes; Bacilli; Bacillales; Staphylococcaceae; Staphylococcus
NC_021323	RefSeq	RVG	G607	45,572	33.3	Staphylococcus phage StauST398-2	Viruses; dsDNA viruses, no RNA stage; Caudovirales; Siphoviridae.	1277672	Bacteria; Firmicutes; Bacilli; Bacillales; Staphylococcaceae; Staphylococcus
NC_028862	RefSeq	RVG	G607	44,222	33.7	Staphylococcus phage vB_SauS_phi2	Viruses; dsDNA viruses, no RNA stage; Caudovirales; Siphoviridae.	1674930	-
NC_002661	RefSeq	RVG	G607	42,942	33.3	Staphylococcus phage Slt	Viruses; dsDNA viruses, no RNA stage; Caudovirales; Siphoviridae; 3alikevirus.	130478	Bacteria; Firmicutes; Bacilli; Bacillales; Staphylococcaceae; Staphylococcus
NC_007052	RefSeq	RVG	G607	45,861	33.7	Staphylococcus phage 42E	Viruses; dsDNA viruses, no RNA stage; Caudovirales; Siphoviridae; 3alikevirus.	320837	Bacteria; Firmicutes; Bacilli; Bacillales; Staphylococcaceae; Staphylococcus
NC_019915	RefSeq	RVG	G608	40,917	34.1	Staphylococcus phage StB20	Viruses; dsDNA viruses, no RNA stage; Caudovirales; Siphoviridae.	1147043	Bacteria; Firmicutes; Bacilli; Bacillales; Staphylococcaceae; Staphylococcus
NC_028821	RefSeq	RVG	G608	40,670	33.3	Staphylococcus phage StB20-like	Viruses; dsDNA viruses, no RNA stage; Caudovirales; Siphoviridae.	1732064	-
NC_029025	RefSeq	RVG	G608	41,843	34	Staphylococcus phage IME-SA4	Viruses; dsDNA viruses, no RNA stage; Caudovirales; Siphoviridae.	1610872	-
NC_022914	RefSeq	RVG	G608	43,394	34.3	Staphylococcus phage phiRS7	Viruses; dsDNA viruses, no RNA stage; Caudovirales; Siphoviridae.	1403390	Bacteria; Firmicutes; Bacilli; Bacillales; Staphylococcaceae; Staphylococcus
NC_007045	RefSeq	RVG	G609	15,603	31.4	Staphylococcus phage PT1028	Viruses; dsDNA viruses, no RNA stage; unclassified dsDNA phages.	320831	Bacteria; Firmicutes; Bacilli; Bacillales; Staphylococcaceae; Staphylococcus

NC_029116	RefSeq	RVG	G610	33,596	30	Clostridium phage phiCDHM13	Viruses; dsDNA viruses, no RNA stage; Caudovirales; Myoviridae.	1437363	-
NC_029001	RefSeq	RVG	G610	32,000	29.3	Clostridium phage phiCDHM11	Viruses; dsDNA viruses, no RNA stage; Caudovirales; Myoviridae; unclassified PhiCD119likevirus.	1445553	-
LK985321	EBI	RVG	G610	32,651	29.5	Clostridium phage phiCDHM14	Viruses; dsDNA viruses, no RNA stage; Caudovirales; Myoviridae.	1522091	-
NC_028951	RefSeq	RVG	G610	32,846	30.3	Clostridium phage phiCD481-1	Viruses; dsDNA viruses, no RNA stage; Caudovirales; Myoviridae.	1582153	-
NC_028838	RefSeq	RVG	G610	33,274	29.6	Clostridium phage phiCD506	Viruses; dsDNA viruses, no RNA stage; Caudovirales; Myoviridae.	1582155	-
NC_019422	RefSeq	RVG	G610	31,674	30	Clostridium phage phiMMP04	Viruses; dsDNA viruses, no RNA stage; Caudovirales; Myoviridae.	1204535	Bacteria; Firmicutes; Clostridia; Clostridiales; Peptostreptococcaceae; Peptoclostridium
NC_015568	RefSeq	RVG	G610	41,090	30.8	Clostridium phage phiCD38-2	Viruses; dsDNA viruses, no RNA stage; Caudovirales; Siphoviridae.	1032362	Bacteria; Firmicutes; Clostridia; Clostridiales; Peptostreptococcaceae; Peptoclostridium
NC_028905	RefSeq	RVG	G610	41,560	30.9	Clostridium phage phiCD111	Viruses; dsDNA viruses, no RNA stage; Caudovirales; Siphoviridae.	1582150	-
NC_028958	RefSeq	RVG	G610	41,507	30.7	Clostridium phage phiCD146	Viruses; dsDNA viruses, no RNA stage; Caudovirales; Siphoviridae.	1582151	-
NC_015262	RefSeq	RVG	G611	37,664	28.4	Clostridium phage phiCD6356	Viruses; dsDNA viruses, no RNA stage; Caudovirales; Siphoviridae.	864178	Bacteria; Firmicutes; Clostridia; Clostridiales; Peptostreptococcaceae; Peptoclostridium
NC_009231	RefSeq	RVG	G612	56,538	28.7	Clostridium phage phiC2	Viruses; dsDNA viruses, no RNA stage; Caudovirales; Myoviridae.	262071	Bacteria; Firmicutes; Clostridia; Clostridiales; Peptostreptococcaceae; Peptoclostridium
NC_028959	RefSeq	RVG	G612	52,261	28.9	Clostridium phage phiMMP03	Viruses; dsDNA viruses, no RNA stage; Caudovirales; Myoviridae.	1582157	-
NC_028883	RefSeq	RVG	G612	44,461	28.9	Clostridium phage phiMMP01	Viruses; dsDNA viruses, no RNA stage; Caudovirales; Myoviridae.	1582156	-
NC_024144	RefSeq	RVG	G612	54,279	28.4	Clostridium phage CDMH1	Viruses; dsDNA viruses, no RNA stage; Caudovirales; Myoviridae.	1411095	Bacteria; Firmicutes; Clostridia; Clostridiales; Peptostreptococcaceae; Peptoclostridium
NC_007917	RefSeq	RVG	G612	53,325	28.8	Clostridium phage phiCD119	Viruses; dsDNA viruses, no RNA stage; Caudovirales; Myoviridae.	320122	Bacteria; Firmicutes; Clostridia; Clostridiales; Peptostreptococcaceae; Peptoclostridium
NC_028996	RefSeq	RVG	G612	54,295	28.2	Clostridium phage phiCDHM19	Viruses; dsDNA viruses, no RNA stage; Caudovirales; Myoviridae.	1522092	-
NC_019421	RefSeq	RVG	G612	48,396	29.6	Clostridium phage phiMMP02	Viruses; dsDNA viruses, no RNA stage; Caudovirales; Myoviridae.	1204534	Bacteria; Firmicutes; Clostridia; Clostridiales; Peptostreptococcaceae; Peptoclostridium
NC_028764	RefSeq	RVG	G612	49,316	29.4	Clostridium phage phiCD505	Viruses; dsDNA viruses, no RNA stage; Caudovirales; Myoviridae.	1582154	-
NC_011398	RefSeq	RVG	G612	50,930	29.3	Clostridium phage phiCD27	Viruses; dsDNA viruses, no RNA stage; Caudovirales; Myoviridae.	559189	Bacteria; Firmicutes; Clostridia; Clostridiales; Peptostreptococcaceae; Peptoclostridium
LN681534	EBI	RVG	G613	44,129	28.5	Clostridium phage phiCD24-1	Viruses; dsDNA viruses, no RNA stage; Caudovirales; Siphoviridae.	1582149	-
NC_029048	RefSeq	RVG	G614	131,326	26.4	Clostridium phage phiCD211	Viruses; dsDNA viruses, no RNA stage; Caudovirales; Myoviridae.	1582152	-
KC595514	EBI	RVG	G615	54,312	38.1	Brevibacillus phage Jimmer2	Viruses; dsDNA viruses, no RNA stage; Caudovirales; Myoviridae.	1296658	-
NC_029104	RefSeq	RVG	G615	54,312	38.1	Brevibacillus phage Jimmer1	Viruses; dsDNA viruses, no RNA stage; Caudovirales; Myoviridae.	1296659	-
NC_028969	RefSeq	RVG	G615	52,955	38.1	Brevibacillus phage Osiris	Viruses; dsDNA viruses, no RNA stage; Caudovirales; Myoviridae.	1691955	-
NC_029029	RefSeq	RVG	G615	45,552	39.2	Brevibacillus phage Abouo	Viruses; dsDNA viruses, no RNA stage; Caudovirales; Myoviridae.	1296661	-
NC_022980	RefSeq	RVG	G615	45,798	39.1	Brevibacillus phage Davies	Viruses; dsDNA viruses, no RNA stage; Caudovirales; Myoviridae.	1296662	Bacteria; Firmicutes; Bacilli; Bacillales; Paenibacillaceae; Brevibacillus
KM983333	EBI	RVG	G616	47,020	28.4	Clostridium phage phiCTC2A	Viruses; unclassified phages.	1567015	-
KM983330	EBI	RVG	G616	47,020	28.4	Clostridium phage phiCT19406A	Viruses; unclassified phages.	1567009	-
NC_029022	RefSeq	RVG	G616	46,895	28.7	Clostridium phage phiCT9441A	Viruses; unclassified phages.	1567014	-
NC_028991	RefSeq	RVG	G616	42,158	29.5	Clostridium phage phiCT453A	Viruses; unclassified phages.	1567012	-
NC_009737	RefSeq	RVG	G617	35,055	44.8	Bacillus virus 1	Viruses; dsDNA viruses, no RNA stage; Caudovirales.	396034	Bacteria; Firmicutes; Bacilli; Bacillales; Bacillaceae; Bacillus
NC_008376	RefSeq	RVG	G617	34,683	44.4	Geobacillus phage GBSV1	Viruses; dsDNA viruses, no RNA stage; Caudovirales.	365048	Bacteria; Firmicutes; Bacilli; Bacillales; Bacillaceae; Geobacillus
NC_021784	RefSeq	RVG	G617	38,099	44.7	Thermus phage phi OH2	Viruses; dsDNA viruses, no RNA stage; Caudovirales.	1352230	Bacteria; Firmicutes; Bacilli; Bacillales; Bacillaceae; Geobacillus
NC_007924	RefSeq	RVG	G618	38,239	36.9	Lactobacillus phage KC5a	Viruses; dsDNA viruses, no RNA stage; Caudovirales; Myoviridae.	363555	Bacteria; Firmicutes; Bacilli; Lactobacillales; Lactobacillaceae; Lactobacillus
NC_024206	RefSeq	RVG	G618	38,269	37.4	Lactobacillus phage phi jlb1	Viruses; dsDNA viruses, no RNA stage; Caudovirales; Myoviridae.	1416334	-
NC_010179	RefSeq	RVG	G618	40,881	36.4	Lactobacillus prophage Lj771	Viruses; unclassified phages.	139871	Bacteria; Firmicutes; Bacilli; Lactobacillales; Lactobacillaceae; Lactobacillus
NC_019782	RefSeq	RVG	G618	36,566	36.5	Lactobacillus phage phiAQ113	Viruses; dsDNA viruses, no RNA stage; Caudovirales; Myoviridae.	1206110	Bacteria; Firmicutes; Bacilli; Lactobacillales; Lactobacillaceae; Lactobacillus
NC_005294	RefSeq	RVG	G619	42,935	39.6	Streptococcus phage EJ-1	Viruses; dsDNA viruses, no RNA stage; Caudovirales; Myoviridae.	12402	Bacteria; Firmicutes; Bacilli; Lactobacillales; Streptococcaceae; Streptococcus
DQ222851	EBI	RVG	G620	36,615	35.3	Bacillus phage Cherry	Viruses; dsDNA viruses, no RNA stage; Caudovirales; Siphoviridae.	347966	-
NC_007458	RefSeq	RVG	G620	37,253	35.2	Bacillus phage Gamma	Viruses; dsDNA viruses, no RNA stage; Caudovirales; Siphoviridae.	347962	Bacteria; Firmicutes; Bacilli; Bacillales; Bacillaceae; Bacillus; Bacillus cereus group
DQ289556	EBI	RVG	G620	37,373	35.1	Bacillus phage Gamma isolate d'Herelle	Viruses; dsDNA viruses, no RNA stage; Caudovirales; Siphoviridae.	359962	-
NC_007814	RefSeq	RVG	G620	37,974	34.9	Bacillus phage Fah	Viruses; dsDNA viruses, no RNA stage; Caudovirales; Siphoviridae.	345922	Bacteria; Firmicutes; Bacilli; Bacillales; Bacillaceae; Bacillus; Bacillus cereus group
NC_007734	RefSeq	RVG	G620	40,867	35.3	Bacillus phage WBeta	Viruses; dsDNA viruses, no RNA stage; Caudovirales; Siphoviridae; Wbetalikevirus.	359961	Bacteria; Firmicutes; Bacilli; Bacillales; Bacillaceae; Bacillus; Bacillus cereus group
NC_018085	RefSeq	RVG	G620	41,992	35.2	Bacillus phage BtCS33	Viruses; dsDNA viruses, no RNA stage; Caudovirales; Siphoviridae.	1194641	Bacteria; Firmicutes; Bacilli; Bacillales; Bacillaceae; Bacillus; Bacillus cereus group
NC_023599	RefSeq	RVG	G620	38,772	35.5	Bacillus phage phiCM3	Viruses; dsDNA viruses, no RNA stage; Caudovirales; Siphoviridae.	1357713	Bacteria; Firmicutes; Bacilli; Bacillales; Bacillaceae; Bacillus; Bacillus cereus group
NC_019502	RefSeq	RVG	G620	44,401	34.9	Bacillus phage phIS3501	Viruses; dsDNA viruses, no RNA stage; Caudovirales; Siphoviridae.	1124578	Bacteria; Firmicutes; Bacilli; Bacillales; Bacillaceae; Bacillus; Bacillus cereus group
HE614282	EBI	RVG	G621	42,932	35.7	Bacillus phage BceA1	Viruses; dsDNA viruses, no RNA stage; Caudovirales; Siphoviridae.	1118064	-
NC_018277	RefSeq	RVG	G621	42,784	35.6	Staphylococcus phage SpaA1	Viruses; dsDNA viruses, no RNA stage; Caudovirales; Siphoviridae.	1118063	Bacteria; Firmicutes; Bacilli; Bacillales; Staphylococcaceae; Staphylococcus
NC_029008	RefSeq	RVG	G621	41,486	35.9	Bacillus phage phi4J1	Viruses; dsDNA viruses, no RNA stage; Caudovirales; Siphoviridae.	1643326	-
NC_028748	RefSeq	RVG	G621	47,732	35.8	Bacillus phage BMBtpLA	Viruses; dsDNA viruses, no RNA stage; Caudovirales; Siphoviridae.	1445809	-
NC_025424	RefSeq	RVG	G621	45,648	35.5	Bacillus phage Waukesha92	Viruses; dsDNA viruses, no RNA stage; Caudovirales; Siphoviridae.	1510440	Bacteria; Firmicutes; Bacilli; Bacillales; Bacillaceae; Bacillus; Bacillus cereus group
NC_004820	RefSeq	RVG	G622	61,395	37.7	Bacillus phage phBC6A51	Viruses; dsDNA viruses, no RNA stage; Caudovirales; Podoviridae.	225725	Bacteria; Firmicutes; Bacilli; Bacillales; Bacillaceae; Bacillus; Bacillus cereus group
NC_019912	RefSeq	RVG	G623	36,932	37.8	Bacillus phage BMBtp2	Viruses; dsDNA viruses, no RNA stage; Caudovirales; Siphoviridae; Tp2unalikevirus.	1236573	Bacteria; Firmicutes; Bacilli; Bacillales; Bacillaceae; Bacillus; Bacillus cereus group
NC_011645	RefSeq	RVG	G623	37,456	37.8	Bacillus phage TP21-L	Viruses; dsDNA viruses, no RNA stage; Caudovirales; Siphoviridae.	565140	Bacteria; Firmicutes; Bacilli; Bacillales; Bacillaceae; Bacillus; Bacillus cereus group
NC_028886	RefSeq	RVG	G623	38,663	35.9	Bacillus phage phi4B1	Viruses; dsDNA viruses, no RNA stage; Caudovirales; Siphoviridae.	1643324	-
NC_011167	RefSeq	RVG	G624	53,104	36.4	Bacillus phage IEBH	Viruses; dsDNA viruses, no RNA stage; Caudovirales; Siphoviridae; Iebhlikevirus.	552525	Bacteria; Firmicutes; Bacilli; Bacillales; Bacillaceae; Bacillus; Bacillus cereus group
NC_029024	RefSeq	RVG	G624	56,505	36.4	Bacillus phage 250	Viruses; dsDNA viruses, no RNA stage; Caudovirales; Siphoviridae; Iebhlikevirus.	701257	-
NC_017976	RefSeq	RVG	G625	41,164	41.7	Bacillus phage PBC1	Viruses; dsDNA viruses, no RNA stage; Caudovirales; Siphoviridae.	1161901	Bacteria; Firmicutes; Bacilli; Bacillales; Bacillaceae; Bacillus; Bacillus cereus group
KC595516	EBI	RVG	G626	58,573	41.4	Brevibacillus phage Emery	Viruses; dsDNA viruses, no RNA stage; Caudovirales; Myoviridae.	1296660	-
KM983334	EBI	RVG	G627	38,243	29.1	Clostridium phage phiCTC2B	Viruses; unclassified phages.	1567016	-
KM983331	EBI	RVG	G627	38,243	29	Clostridium phage phiCT19406B	Viruses; unclassified phages.	1567010	-
NC_029004	RefSeq	RVG	G627	36,712	29.1	Clostridium phage phiCT453B	Viruses; unclassified phages.	1567013	-
NC_029006	RefSeq	RVG	G627	35,703	29.2	Clostridium phage phiCT19406C	Viruses; unclassified phages.	1567011	-
NC_017978	RefSeq	RVG	G628	33,609	27.5	Clostridium phage PhiS63	Viruses; dsDNA viruses, no RNA stage; Caudovirales; Siphoviridae.	1187894	Bacteria; Firmicutes; Clostridia; Clostridiales; Clostridiaceae; Clostridium
NC_003524	RefSeq	RVG	G629	33,507	28.4	Clostridium phage phi3626	Viruses; dsDNA viruses, no RNA stage; Caudovirales; Siphoviridae.	190478	Bacteria; Firmicutes; Clostridia; Clostridiales; Clostridiaceae; Clostridium
NC_008265	RefSeq	RVG	G630	38,092	28.1	Clostridium phage phiSM101	Viruses; dsDNA viruses, no RNA stage; unclassified dsDNA phages.	396359	Bacteria; Firmicutes; Clostridia; Clostridiales; Clostridiaceae; Clostridium
NC_021325	RefSeq	RVG	G631	39,108	28.1	Clostridium phage vB_CpeS-CP51	Viruses; dsDNA viruses, no RNA stage; Caudovirales; Siphoviridae.	1305708	Bacteria; Firmicutes; Clostridia; Clostridiales; Clostridiaceae; Clostridium
NC_019544	RefSeq	RVG	G632	49,335	46	Deep-sea thermophilic phage D6E	Viruses; dsDNA viruses, no RNA stage; unclassified dsDNA phages.	749413	Bacteria; Firmicutes; Bacilli; Bacillales; Bacillaceae; Geobacillus
NC_009552	RefSeq	RVG	G632	40,863	44.8	Geobacillus virus E2	Viruses; unclassified phages.	447909	Bacteria; Firmicutes; Bacilli; Bacillales; Bacillaceae

NC_004167	RefSeq	RVG	G633	39,325	42.7	Bacillus phage phi105	Viruses; dsDNA viruses, no RNA stage; Caudovirales; Siphoviridae; Lambdalikevirus; unclassified Lambda-like viruses.	10717	Bacteria; Firmicutes; Bacilli; Bacillales; Bacillaceae; Bacillus; Bacillus subtilis group
NC_021558	RefSeq	RVG	G634	37,644	42.4	Paenibacillus phage PG1	Viruses; dsDNA viruses, no RNA stage; Caudovirales; Siphoviridae.	754053	Bacteria; Firmicutes; Bacilli; Bacillales; Paenibacillaceae; Paenibacillus
NC_028830	RefSeq	RVG	G635	34,155	45.6	Lactobacillus phage iA2	Viruses; dsDNA viruses, no RNA stage; Caudovirales; Siphoviridae.	1739609	-
NC_004821	RefSeq	RVG	G636	38,472	34.7	Bacillus phage phBC6A52	Viruses; dsDNA viruses, no RNA stage; Caudovirales; Podoviridae.	225726	Bacteria; Firmicutes; Bacilli; Bacillales; Bacillaceae; Bacillus; Bacillus cereus group
KF669657	EBI	RVG	G637	39,874	40.7	Bacillus phage poppyseed	Viruses; dsDNA viruses, no RNA stage; Caudovirales; Podoviridae.	1406787	-
NC_022764	RefSeq	RVG	G637	39,874	40.7	Bacillus phage Page	Viruses; dsDNA viruses, no RNA stage; Caudovirales; Podoviridae.	1406786	Bacteria; Firmicutes; Bacilli; Bacillales; Bacillaceae; Bacillus
NC_022770	RefSeq	RVG	G637	39,844	40.7	Bacillus phage Pony	Viruses; dsDNA viruses, no RNA stage; Caudovirales; Podoviridae.	1406789	Bacteria; Firmicutes; Bacilli; Bacillales; Bacillaceae; Bacillus
NC_027394	RefSeq	RVG	G637	40,214	40.6	Bacillus phage Pookie	Viruses; dsDNA viruses, no RNA stage; Caudovirales; Podoviridae.	1540093	Bacteria; Firmicutes; Bacilli; Bacillales; Bacillaceae; Bacillus
NC_028782	RefSeq	RVG	G637	40,024	40.6	Bacillus phage Pavlov	Viruses; dsDNA viruses, no RNA stage; Caudovirales; Podoviridae.	1675598	-
NC_028926	RefSeq	RVG	G637	40,000	40.7	Bacillus phage Palmer	Viruses; dsDNA viruses, no RNA stage; Caudovirales; Podoviridae.	1597966	-
NC_027372	RefSeq	RVG	G637	39,639	39.9	Bacillus phage Pascal	Viruses; dsDNA viruses, no RNA stage; Caudovirales; Podoviridae.	1540092	Bacteria; Firmicutes; Bacilli; Bacillales; Bacillaceae; Bacillus
NC_009813	RefSeq	RVG	G638	48,172	36.2	Listeria phage B054	Viruses; dsDNA viruses, no RNA stage; Caudovirales; Siphoviridae.	330397	Bacteria; Firmicutes; Bacilli; Bacillales; Listeriaceae; Listeria
NC_027990	RefSeq	RVG	G639	48,211	45.9	Lactobacillus phage LBR48	Viruses; dsDNA viruses, no RNA stage; Caudovirales; Myoviridae.	755164	Bacteria; Firmicutes; Bacilli; Lactobacillales; Lactobacillaceae; Lactobacillus
NC_029078	RefSeq	RVG	G640	34,997	34	Erysipelothrix phage SE-1	Viruses; dsDNA viruses, no RNA stage; Caudovirales; Siphoviridae.	1675317	-
KC330682	EBI	RVG	G641	49,492	42.3	Bacillus phage Taylor	Viruses; dsDNA viruses, no RNA stage; Caudovirales; Siphoviridae; Andromedalikevirus.	1273744	-
NC_020477	RefSeq	RVG	G641	49,458	42.2	Bacillus phage Eoghan	Viruses; dsDNA viruses, no RNA stage; Caudovirales; Siphoviridae; Andromedalikevirus.	1273741	Bacteria; Firmicutes; Bacilli; Bacillales; Bacillaceae; Bacillus
NC_022773	RefSeq	RVG	G641	50,354	42.2	Bacillus phage Blastoid	Viruses; dsDNA viruses, no RNA stage; Caudovirales; Siphoviridae; Andromedalikevirus.	1406782	Bacteria; Firmicutes; Bacilli; Bacillales; Bacillaceae; Bacillus
NC_020480	RefSeq	RVG	G641	50,161	41.7	Bacillus phage Finn	Viruses; dsDNA viruses, no RNA stage; Caudovirales; Siphoviridae; Andromedalikevirus.	1273742	Bacteria; Firmicutes; Bacilli; Bacillales; Bacillaceae; Bacillus
NC_022765	RefSeq	RVG	G641	49,836	41.5	Bacillus phage Riggi	Viruses; dsDNA viruses, no RNA stage; Caudovirales; Siphoviridae; Andromedalikevirus.	1406788	Bacteria; Firmicutes; Bacilli; Bacillales; Bacillaceae; Bacillus
KC330681	EBI	RVG	G641	49,362	41.9	Bacillus phage Gemini	Viruses; dsDNA viruses, no RNA stage; Caudovirales; Siphoviridae; Andromedalikevirus.	1273743	-
NC_020478	RefSeq	RVG	G641	49,259	41.9	Bacillus phage Andromeda	Viruses; dsDNA viruses, no RNA stage; Caudovirales; Siphoviridae; Andromedalikevirus.	1273739	Bacteria; Firmicutes; Bacilli; Bacillales; Bacillaceae; Bacillus
NC_022766	RefSeq	RVG	G641	49,246	42.1	Bacillus phage Glittering	Viruses; dsDNA viruses, no RNA stage; Caudovirales; Siphoviridae; Andromedalikevirus.	1406784	Bacteria; Firmicutes; Bacilli; Bacillales; Bacillaceae; Bacillus
NC_020479	RefSeq	RVG	G641	49,425	41.8	Bacillus phage Curly	Viruses; dsDNA viruses, no RNA stage; Caudovirales; Siphoviridae; Andromedalikevirus.	1273740	Bacteria; Firmicutes; Bacilli; Bacillales; Bacillaceae; Bacillus
NC_004166	RefSeq	RVG	G642	44,010	43.7	Bacillus phage SPP1	Viruses; dsDNA viruses, no RNA stage; Caudovirales; Siphoviridae; Lambdalikevirus; unclassified Lambda-like viruses.	10724	Bacteria; Firmicutes; Bacilli; Bacillales; Bacillaceae; Bacillus; Bacillus subtilis group
NC_023612	RefSeq	RVG	G643	39,078	43.1	Geobacillus phage GBK2	Viruses; dsDNA viruses, no RNA stage; Caudovirales; Siphoviridae.	1458842	Bacteria; Firmicutes; Bacilli; Bacillales; Bacillaceae; Geobacillus
NC_020883	RefSeq	RVG	G644	50,861	41.3	Bacillus phage PM1	Viruses; dsDNA viruses, no RNA stage; Caudovirales; Siphoviridae.	547228	Bacteria; Firmicutes; Bacilli; Bacillales; Bacillaceae; Bacillus; Bacillus subtilis group; Bacillus subtilis
JF767210	EBI	RVG	G645	39,594	30.2	Clostridium phage phiCP9O	Viruses; dsDNA viruses, no RNA stage; Caudovirales; Siphoviridae.	1042124	-
NC_019496	RefSeq	RVG	G645	39,188	30.3	Clostridium phage phiCP26F	Viruses; dsDNA viruses, no RNA stage; Caudovirales; Siphoviridae.	673376	Bacteria; Firmicutes; Clostridia; Clostridiales; Clostridiaceae; Clostridium
NC_011318	RefSeq	RVG	G645	38,753	30.4	Clostridium phage phiC39-O	Viruses; dsDNA viruses, no RNA stage; Caudovirales; Siphoviridae.	541865	Bacteria; Firmicutes; Clostridia; Clostridiales; Clostridiaceae; Clostridium
NC_019508	RefSeq	RVG	G645	38,309	30.5	Clostridium phage phiCP34O	Viruses; dsDNA viruses, no RNA stage; Caudovirales; Siphoviridae.	1042123	Bacteria; Firmicutes; Clostridia; Clostridiales; Clostridiaceae; Clostridium
NC_019506	RefSeq	RVG	G645	38,329	30.4	Clostridium phage phiCP13O	Viruses; dsDNA viruses, no RNA stage; Caudovirales; Siphoviridae.	1042122	Bacteria; Firmicutes; Clostridia; Clostridiales; Clostridiaceae; Clostridium
NC_011308	RefSeq	RVG	G646	35,638	39.3	Listeria phage P40	Viruses; dsDNA viruses, no RNA stage; Caudovirales; Siphoviridae.	560178	Bacteria; Firmicutes; Bacilli; Bacillales; Listeriaceae
NC_009814	RefSeq	RVG	G646	35,822	40.8	Listeria phage P35	Viruses; dsDNA viruses, no RNA stage; Caudovirales; Siphoviridae.	330398	Bacteria; Firmicutes; Bacilli; Bacillales; Listeriaceae; Listeria
NC_027120	RefSeq	RVG	G647	36,892	51	Lactococcus phage 1358	Viruses; dsDNA viruses, no RNA stage; Caudovirales; Siphoviridae.	741942	Bacteria; Firmicutes; Bacilli; Lactobacillales; Streptococcaceae; Lactococcus
JF731128	EBI	RVG	G648	58,619	40	Enterococcus phage SAP6	Viruses; dsDNA viruses, no RNA stage; Caudovirales; Siphoviridae; Sap6likevirus.	1073766	-
NC_018086	RefSeq	RVG	G648	53,996	40.4	Enterococcus phage BC611	Viruses; dsDNA viruses, no RNA stage; Caudovirales; Siphoviridae; Sap6likevirus.	1173135	Bacteria; Firmicutes; Bacilli; Lactobacillales; Enterococcaceae; Enterococcus
KF192053	EBI	RVG	G648	57,081	40	Enterococcus phage IMEEF1	Viruses; dsDNA viruses, no RNA stage; Caudovirales; Siphoviridae; Sap6likevirus.	1351735	-
NC_029016	RefSeq	RVG	G648	58,000	40	Enterococcus phage vB_IME198	Viruses; dsDNA viruses, no RNA stage; Caudovirales; Siphoviridae.	1747287	-
NC_021868	RefSeq	RVG	G648	58,305	39.9	Streptococcus phage SPQS1	Viruses; dsDNA viruses, no RNA stage; Caudovirales; Siphoviridae; Sap6likevirus.	1208587	Bacteria; Firmicutes; Bacilli; Lactobacillales; Streptococcaceae; Streptococcus
NC_024212	RefSeq	RVG	G648	55,726	40	Enterococcus phage VD13	Viruses; dsDNA viruses, no RNA stage; Caudovirales; Siphoviridae; Sap6likevirus.	1458851	Bacteria; Firmicutes; Bacilli; Lactobacillales; Enterococcaceae; Enterococcus
NC_021787	RefSeq	RVG	G649	64,756	36.6	Listeria phage LP-037	Viruses; dsDNA viruses, no RNA stage; Caudovirales; Siphoviridae.	1173747	Bacteria; Firmicutes; Bacilli; Bacillales; Listeriaceae; Listeria
NC_024375	RefSeq	RVG	G649	67,150	36.3	Listeria phage LP-026	Viruses; dsDNA viruses, no RNA stage; Caudovirales; Siphoviridae.	1173745	Bacteria; Firmicutes; Bacilli; Bacillales; Listeriaceae; Listeria
NC_021785	RefSeq	RVG	G649	65,132	36.3	Listeria phage LP-110	Viruses; dsDNA viruses, no RNA stage; Caudovirales; Siphoviridae.	1173748	Bacteria; Firmicutes; Bacilli; Bacillales; Listeriaceae; Listeria
NC_018831	RefSeq	RVG	G649	67,170	36.5	Listeria phage P70	Viruses; dsDNA viruses, no RNA stage; Caudovirales; Siphoviridae.	1225800	Bacteria; Firmicutes; Bacilli; Bacillales; Listeriaceae
NC_024392	RefSeq	RVG	G649	66,676	36.4	Listeria phage LP-114	Viruses; dsDNA viruses, no RNA stage; Caudovirales; Siphoviridae.	1458857	Bacteria; Firmicutes; Bacilli; Bacillales; Listeriaceae; Listeria
NC_028930	RefSeq	RVG	G650	54,439	48.3	Paenibacillus phage Tripp	Viruses; dsDNA viruses, no RNA stage; Caudovirales; Siphoviridae.	1718161	-
NC_015274	RefSeq	RVG	G651	56,506	40.3	Streptococcus phage Dp-1	Viruses; dsDNA viruses, no RNA stage; Caudovirales; Siphoviridae.	59241	Bacteria; Firmicutes; Bacilli; Lactobacillales; Streptococcaceae; Streptococcus
NC_014457	RefSeq	RVG	G652	59,199	31.3	Clostridium phage phiCTP1	Viruses; dsDNA viruses, no RNA stage; Caudovirales; Siphoviridae.	871584	Bacteria; Firmicutes; Clostridia; Clostridiales; Clostridiaceae; Clostridium
NC_019924	RefSeq	RVG	G653	47,595	35.4	Clostridium phage phi8074-B1	Viruses; dsDNA viruses, no RNA stage; Caudovirales; Siphoviridae.	1147137	Bacteria; Firmicutes; Clostridia; Clostridiales; Clostridiaceae; Clostridium
LDNN01000032	cryoconite	EVG	G654	13,195	56.3	-	-	-	-
NC_018264	RefSeq	RVG	G655	41,938	36.8	Thermoanaerobacterium phage THSA-485A	Viruses; dsDNA viruses, no RNA stage; Caudovirales; Siphoviridae.	1126885	Bacteria; Firmicutes; Clostridia; Thermoanaerobacterales; Thermoanaerobacterales Family III. Incertae Sedis; Thermoanaerobacterium
KP687432	EBI	RVG	G656	140,906	30.3	Staphylococcus phage IME-SA2	Viruses; dsDNA viruses, no RNA stage; Caudovirales; Myoviridae; Spounavirinae; Twortlikevirus.	1610831	-
KP687431	EBI	RVG	G656	140,218	30.3	Staphylococcus phage IME-SA1	Viruses; dsDNA viruses, no RNA stage; Caudovirales; Myoviridae; Spounavirinae; Twortlikevirus.	1610830	-

NC_025417	RefSeq	RVG	G656	140,903	30.3	Staphylococcus phage Team1	Viruses; dsDNA viruses, no RNA stage; Caudovirales; Myoviridae; Spounavirinae; Twortlikevirus.	1262512	Bacteria; Firmicutes; Bacilli; Bacillales; Staphylococcaceae; Staphylococcus
JX875065	EBI	RVG	G656	137,031	30.4	Staphylococcus phage SA5	Viruses; dsDNA viruses, no RNA stage; Caudovirales; Myoviridae; Spounavirinae; Twortlikevirus.	1239385	-
NC_007066	RefSeq	RVG	G656	138,715	30.4	Staphylococcus phage G1	Viruses; dsDNA viruses, no RNA stage; Caudovirales; Myoviridae; Spounavirinae; Twortlikevirus.	292029	Bacteria; Firmicutes; Bacilli; Bacillales; Staphylococcaceae; Staphylococcus
FR852584	EBI	RVG	G656	138,339	30.4	Staphylococcus phage ISP	Viruses; dsDNA viruses, no RNA stage; Caudovirales; Myoviridae; Spounavirinae; Twortlikevirus.	1028375	-
NC_023009	RefSeq	RVG	G656	127,188	30.5	Staphylococcus phage Sb-1	Viruses; dsDNA viruses, no RNA stage; Caudovirales; Myoviridae; Spounavirinae; Spounalikevirus; unclassified SPO1-like viruses.	1007127	Bacteria; Firmicutes; Bacilli; Bacillales; Staphylococcaceae; Staphylococcus
EU418428	EBI	RVG	G656	145,542	30.4	Staphylococcus phage A5W	Viruses; dsDNA viruses, no RNA stage; Caudovirales; Myoviridae; Spounavirinae; Twortlikevirus.	516541	-
NC_029080	RefSeq	RVG	G656	142,096	30.4	Staphylococcus phage 812	Viruses; dsDNA viruses, no RNA stage; Caudovirales; Myoviridae; Spounavirinae; Twortlikevirus; unclassified Twortlikevirus.	307898	-
NC_005880	RefSeq	RVG	G656	148,317	30.4	Staphylococcus phage K	Viruses; dsDNA viruses, no RNA stage; Caudovirales; Myoviridae; Spounavirinae; Twortlikevirus.	221915	Bacteria; Firmicutes; Bacilli; Bacillales; Staphylococcaceae; Staphylococcus
NC_019726	RefSeq	RVG	G656	141,836	30.4	Staphylococcus phage JD007	Viruses; dsDNA viruses, no RNA stage; Caudovirales; Myoviridae; Spounavirinae; Twortlikevirus; unclassified Twortlikevirus.	1239383	Bacteria; Firmicutes; Bacilli; Bacillales; Staphylococcaceae; Staphylococcus
NC_023573	RefSeq	RVG	G656	142,094	30.3	Staphylococcus phage phiSA012	Viruses; dsDNA viruses, no RNA stage; Caudovirales; Myoviridae; Spounavirinae; Twortlikevirus; unclassified Twortlikevirus.	1450142	Bacteria; Firmicutes; Bacilli; Bacillales; Staphylococcaceae; Staphylococcus
NC_022920	RefSeq	RVG	G656	139,738	30.2	Staphylococcus phage S25-3	Viruses; dsDNA viruses, no RNA stage; Caudovirales; Myoviridae; Spounavirinae; Twortlikevirus; unclassified Twortlikevirus.	1041526	Bacteria; Firmicutes; Bacilli; Bacillales; Staphylococcaceae; Staphylococcus
NC_022918	RefSeq	RVG	G656	132,123	30.3	Staphylococcus phage S25-4	Viruses; dsDNA viruses, no RNA stage; Caudovirales; Myoviridae; Spounavirinae; Twortlikevirus; unclassified Twortlikevirus.	1041527	Bacteria; Firmicutes; Bacilli; Bacillales; Staphylococcaceae; Staphylococcus
NC_019448	RefSeq	RVG	G656	139,806	30.2	Staphylococcus phage GH15	Viruses; dsDNA viruses, no RNA stage; Caudovirales; Myoviridae; Spounavirinae; Twortlikevirus; unclassified Twortlikevirus.	760530	Bacteria; Firmicutes; Bacilli; Bacillales; Staphylococcaceae; Staphylococcus
NC_025426	RefSeq	RVG	G656	140,807	30.2	Staphylococcus phage P108	Viruses; dsDNA viruses, no RNA stage; Caudovirales; Myoviridae; Spounavirinae; Twortlikevirus; unclassified Twortlikevirus.	1526408	Bacteria; Firmicutes; Bacilli; Bacillales; Staphylococcaceae; Staphylococcus
NC_028765	RefSeq	RVG	G656	142,348	30.4	Staphylococcus phage phiIPLA-RODI	Viruses; dsDNA viruses, no RNA stage; Caudovirales; Myoviridae.	1572703	-
NC_025416	RefSeq	RVG	G656	141,907	30.4	Staphylococcus phage MCE-2014	Viruses; dsDNA viruses, no RNA stage; Caudovirales; Myoviridae; Spounavirinae; Twortlikevirus; unclassified Twortlikevirus.	1524910	Bacteria; Firmicutes; Bacilli; Bacillales; Staphylococcaceae; Staphylococcus
NC_022090	RefSeq	RVG	G656	134,643	30	Staphylococcus phage vB_SauM_Remus	Viruses; dsDNA viruses, no RNA stage; Caudovirales; Myoviridae; Spounavirinae; Twortlikevirus; unclassified Twortlikevirus.	1235659	Bacteria; Firmicutes; Bacilli; Bacillales; Staphylococcaceae; Staphylococcus
NC_020877	RefSeq	RVG	G656	131,332	30	Staphylococcus phage vB_SauM_Romulus	Viruses; dsDNA viruses, no RNA stage; Caudovirales; Myoviridae; Spounavirinae; Twortlikevirus; unclassified Twortlikevirus.	1235660	Bacteria; Firmicutes; Bacilli; Bacillales; Staphylococcaceae; Staphylococcus
NC_019511	RefSeq	RVG	G656	136,326	30	Staphylococcus phage SA11	Viruses; dsDNA viruses, no RNA stage; Caudovirales.	1204543	Bacteria; Firmicutes; Bacilli; Bacillales; Staphylococcaceae; Staphylococcus
KF021268	EBI	RVG	G656	139,928	27.9	Staphylococcus phage phiIBB-SEP1	Viruses; dsDNA viruses, no RNA stage; Caudovirales; Myoviridae.	1340769	-
NC_028962	RefSeq	RVG	G656	140,961	28	Staphylococcus phage phiIPLA-C1C	Viruses; dsDNA viruses, no RNA stage; Caudovirales; Myoviridae.	1572704	-
NC_007021	RefSeq	RVG	G656	130,706	30.3	Staphylococcus phage Twort	Viruses; dsDNA viruses, no RNA stage; Caudovirales; Myoviridae; Spounavirinae; Twortlikevirus.	55510	Bacteria; Firmicutes; Bacilli; Bacillales; Staphylococcaceae; Staphylococcus
KJ094029	EBI	RVG	G656	135,279	35.9	Listeria phage LP-064	Viruses; dsDNA viruses, no RNA stage; Caudovirales; Myoviridae; Spounavirinae; Twortlikevirus.	1458853	-
NC_021781	RefSeq	RVG	G656	135,281	35.9	Listeria phage LP-125	Viruses; dsDNA viruses, no RNA stage; Caudovirales; Myoviridae; Spounavirinae; Twortlikevirus.	1173766	Bacteria; Firmicutes; Bacilli; Bacillales; Listeriaceae; Listeria
DQ004855	EBI	RVG	G656	131,384	36	Listeria phage P100	Viruses; dsDNA viruses, no RNA stage; Caudovirales; Myoviridae; Spounavirinae; Twortlikevirus.	330395	-
KJ094031	EBI	RVG	G656	135,764	35.9	Listeria phage LP-124	Viruses; dsDNA viruses, no RNA stage; Caudovirales; Myoviridae; Spounavirinae; Twortlikevirus.	1173765	-
NC_024383	RefSeq	RVG	G656	135,831	35.9	Listeria phage LP-083-2	Viruses; dsDNA viruses, no RNA stage; Caudovirales; Myoviridae; Spounavirinae; Twortlikevirus.	1458855	Bacteria; Firmicutes; Bacilli; Bacillales; Listeriaceae; Listeria
NC_024364	RefSeq	RVG	G656	131,952	36	Listeria phage List-36	Viruses; dsDNA viruses, no RNA stage; Caudovirales; Myoviridae.	1486422	Bacteria; Firmicutes; Bacilli; Bacillales; Listeriaceae; Listeria
NC_024787	RefSeq	RVG	G656	132,541	36	Listeria phage LMTA-148	Viruses; dsDNA viruses, no RNA stage; Caudovirales; Myoviridae.	1486413	Bacteria; Firmicutes; Bacilli; Bacillales; Listeriaceae; Listeria
NC_024359	RefSeq	RVG	G656	133,048	36	Listeria phage LP-048	Viruses; dsDNA viruses, no RNA stage; Caudovirales; Myoviridae.	1173764	Bacteria; Firmicutes; Bacilli; Bacillales; Listeriaceae; Listeria
NC_020871	RefSeq	RVG	G656	133,057	35.9	Listeria phage vB_LmoM_AG20	Viruses; dsDNA viruses, no RNA stage; Caudovirales; Myoviridae; Spounavirinae; Spounalikevirus; unclassified SPO1-like viruses.	1168744	Bacteria; Firmicutes; Bacilli; Bacillales; Listeriaceae; Listeria
NC_009811	RefSeq	RVG	G656	137,619	35.9	Listeria phage A511	Viruses; dsDNA viruses, no RNA stage; Caudovirales; Myoviridae; Spounavirinae; Twortlikevirus.	40523	Bacteria; Firmicutes; Bacilli; Bacillales; Listeriaceae; Listeria
NC_025440	RefSeq	RVG	G656	134,369	36	Listeria phage WIL-1	Viruses; dsDNA viruses, no RNA stage; Caudovirales; Myoviridae.	1541821	Bacteria; Firmicutes; Bacilli; Bacillales; Listeriaceae; Listeria
KJ586794	EBI	RVG	G656	138,036	35.9	Listeria phage LMTA-34	Viruses; dsDNA viruses, no RNA stage; Caudovirales; Myoviridae.	1486397	-
NC_024360	RefSeq	RVG	G656	138,036	35.9	Listeria phage LMSP-25	Viruses; dsDNA viruses, no RNA stage; Caudovirales; Myoviridae.	1486421	Bacteria; Firmicutes; Bacilli; Bacillales; Listeriaceae; Listeria
KJ591605	EBI	RVG	G656	136,589	35.8	Listeria phage LMTA-57	Viruses; dsDNA viruses, no RNA stage; Caudovirales; Myoviridae.	1486414	-
KJ586795	EBI	RVG	G656	136,468	35.8	Listeria phage LMTA-94	Viruses; dsDNA viruses, no RNA stage; Caudovirales; Myoviridae.	1486419	-
AB609718	EBI	RVG	G656	142,072	35.7	Enterococcus phage phiEF24C-P2	Viruses; dsDNA viruses, no RNA stage; Caudovirales; Myoviridae; Spounavirinae; unassigned Spounavirinae.	947379	-
NC_009904	RefSeq	RVG	G656	142,072	35.7	Enterococcus phage phiEF24C	Viruses; dsDNA viruses, no RNA stage; Caudovirales; Myoviridae; Spounavirinae; unassigned Spounavirinae.	442493	Bacteria; Firmicutes; Bacilli; Lactobacillales; Enterococcaceae; Enterococcus
NC_027335	RefSeq	RVG	G656	145,518	35.9	Enterococcus phage ECP3	Viruses; dsDNA viruses, no RNA stage; Caudovirales; Myoviridae.	1498168	Bacteria; Firmicutes; Bacilli; Lactobacillales; Enterococcaceae; Enterococcus
NC_029026	RefSeq	RVG	G656	130,952	35.9	Enterococcus phage EFLK1	Viruses; dsDNA viruses, no RNA stage; Caudovirales; Myoviridae.	1640885	-
NC_029009	RefSeq	RVG	G656	147,589	37.2	Enterococcus phage EFDG1	Viruses; dsDNA viruses, no RNA stage; Caudovirales; Myoviridae.	1597976	-
NC_028887	RefSeq	RVG	G657	167,431	37.7	Bacillus phage AvesoBmore	Viruses; dsDNA viruses, no RNA stage; Caudovirales; Myoviridae.	1698451	-
NC_024788	RefSeq	RVG	G657	162,816	37.8	Bacillus phage Riley	Viruses; dsDNA viruses, no RNA stage; Caudovirales; Myoviridae.	1486662	Bacteria; Firmicutes; Bacilli; Bacillales; Bacillaceae; Bacillus; Bacillus cereus group
NC_022088	RefSeq	RVG	G657	163,019	37.8	Bacillus phage Troll	Viruses; dsDNA viruses, no RNA stage; Caudovirales; Myoviridae.	1382932	Bacteria; Firmicutes; Bacilli; Bacillales; Bacillaceae; Bacillus; Bacillus cereus group
NC_022769	RefSeq	RVG	G657	165,238	37.8	Bacillus phage BigBertha	Viruses; dsDNA viruses, no RNA stage; Caudovirales; Myoviridae.	1406781	Bacteria; Firmicutes; Bacilli; Bacillales; Bacillaceae; Bacillus; Bacillus cereus group
JN797796	EBI	RVG	G657	162,598	37.7	Bacillus phage B5S	Viruses; dsDNA viruses, no RNA stage; Caudovirales; Myoviridae.	1126949	-

NC_018863	RefSeq	RVG	G657	162,596	37.7	Bacillus phage B4	Viruses; dsDNA viruses, no RNA stage; Caudovirales; Myoviridae.	1141133	Bacteria; Firmicutes; Bacilli; Bacillales; Bacillaceae; Bacillus; Bacillus cereus group
NC_022763	RefSeq	RVG	G657	164,297	37.6	Bacillus phage Spock	Viruses; dsDNA viruses, no RNA stage; Caudovirales; Myoviridae.	1406791	Bacteria; Firmicutes; Bacilli; Bacillales; Bacillaceae; Bacillus; Bacillus cereus group
NC_029069	RefSeq	RVG	G657	165,031	38.1	Bacillus phage BM5	Viruses; dsDNA viruses, no RNA stage; Caudovirales; Myoviridae.	1755679	-
NC_024216	RefSeq	RVG	G657	160,541	38	Bacillus phage CAM003	Viruses; dsDNA viruses, no RNA stage; Caudovirales; Myoviridae.	1486657	Bacteria; Firmicutes; Bacilli; Bacillales; Bacillaceae; Bacillus; Bacillus cereus group
NC_024205	RefSeq	RVG	G657	159,837	38	Bacillus phage Hoody T	Viruses; dsDNA viruses, no RNA stage; Caudovirales; Myoviridae.	1486660	Bacteria; Firmicutes; Bacilli; Bacillales; Bacillaceae; Bacillus; Bacillus cereus group
NC_024207	RefSeq	RVG	G657	159,656	38.1	Bacillus phage Evoli	Viruses; dsDNA viruses, no RNA stage; Caudovirales; Myoviridae.	1486658	Bacteria; Firmicutes; Bacilli; Bacillales; Bacillaceae; Bacillus; Bacillus cereus group
NC_018856	RefSeq	RVG	G657	153,962	38.1	Bacillus phage Bastille	Viruses; dsDNA viruses, no RNA stage; Caudovirales; Myoviridae; Spounavirinae; Spounalikevirus; unclassified SPO1-like viruses.	57477	Bacteria; Firmicutes; Bacilli; Bacillales; Bacillaceae; Bacillus; Bacillus cereus group
NC_020873	RefSeq	RVG	G657	158,621	40	Bacillus phage vB_BceM-Bc431v3	Viruses; dsDNA viruses, no RNA stage; Caudovirales; Myoviridae; Spounavirinae; Spounalikevirus; unclassified SPO1-like viruses.	1195072	Bacteria; Firmicutes; Bacilli; Bacillales; Bacillaceae; Bacillus; Bacillus cereus group
NC_024137	RefSeq	RVG	G657	152,778	39.8	Bacillus phage Bcp1	Viruses; dsDNA viruses, no RNA stage; Caudovirales; Myoviridae.	584892	Bacteria; Firmicutes; Bacilli; Bacillales; Bacillaceae; Bacillus; Bacillus cereus group
NC_027352	RefSeq	RVG	G657	159,492	39.7	Bacillus phage JBP901	Viruses; dsDNA viruses, no RNA stage; Caudovirales; Myoviridae; Spounavirinae; Spounalikevirus; unclassified SPO1-like viruses.	1498212	Bacteria; Firmicutes; Bacilli; Bacillales; Bacillaceae; Bacillus; Bacillus cereus group
NC_027355	RefSeq	RVG	G657	159,071	39.5	Bacillus phage BCP8-2	Viruses; dsDNA viruses, no RNA stage; Caudovirales; Myoviridae; Spounavirinae; Spounalikevirus; unclassified SPO1-like viruses.	1129192	Bacteria; Firmicutes; Bacilli; Bacillales; Bacillaceae; Bacillus; Bacillus cereus group
JN797798	EBI	RVG	G657	154,371	39.9	Bacillus phage BCU4	Viruses; dsDNA viruses, no RNA stage; Caudovirales; Myoviridae.	1126951	-
NC_018860	RefSeq	RVG	G657	156,176	39.9	Bacillus phage BCP78	Viruses; dsDNA viruses, no RNA stage; Caudovirales; Myoviridae.	1126950	Bacteria; Firmicutes; Bacilli; Bacillales; Bacillaceae; Bacillus; Bacillus cereus group
NC_028890	RefSeq	RVG	G657	162,486	40.1	Bacillus phage TsarBomba	Viruses; dsDNA viruses, no RNA stage; Caudovirales; Myoviridae.	1690456	-
NC_028944	RefSeq	RVG	G657	162,252	38.8	Bacillus phage Eyuki	Viruses; dsDNA viruses, no RNA stage; Caudovirales; Myoviridae.	1690431	-
NC_024211	RefSeq	RVG	G657	158,750	38.8	Bacillus phage Megatron	Viruses; dsDNA viruses, no RNA stage; Caudovirales; Myoviridae.	1486661	Bacteria; Firmicutes; Bacilli; Bacillales; Bacillaceae; Bacillus; Bacillus cereus group
NC_024213	RefSeq	RVG	G657	158,100	38.7	Bacillus phage Hakuna	Viruses; dsDNA viruses, no RNA stage; Caudovirales; Myoviridae.	1486659	Bacteria; Firmicutes; Bacilli; Bacillales; Bacillaceae; Bacillus; Bacillus cereus group
NC_018857	RefSeq	RVG	G657	158,305	38.8	Bacillus phage BPS13	Viruses; dsDNA viruses, no RNA stage; Caudovirales; Myoviridae.	1136731	Bacteria; Firmicutes; Bacilli; Bacillales; Bacillaceae; Bacillus; Bacillus cereus group
NC_023501	RefSeq	RVG	G657	159,590	38.7	Bacillus phage BPS10C	Viruses; dsDNA viruses, no RNA stage; Caudovirales; Myoviridae.	1277886	Bacteria; Firmicutes; Bacilli; Bacillales; Bacillaceae; Bacillus; Bacillus cereus group
NC_016563	RefSeq	RVG	G657	156,897	36.4	Bacillus phage W.Ph.	Viruses; dsDNA viruses, no RNA stage; Caudovirales; Myoviridae.	764595	Bacteria; Firmicutes; Bacilli; Bacillales; Bacillaceae; Bacillus; Bacillus cereus group
KJ010548	EBI	RVG	G657	151,419	41.2	Bacillus phage Bp8p-T	Viruses; dsDNA viruses, no RNA stage; Caudovirales; Myoviridae.	1445811	-
NC_029121	RefSeq	RVG	G657	151,417	41.2	Bacillus phage Bp8p-C	Viruses; dsDNA viruses, no RNA stage; Caudovirales; Myoviridae.	1445810	-
NC_024792	RefSeq	RVG	G657	160,281	41.3	Bacillus phage Bobb	Viruses; dsDNA viruses, no RNA stage; Caudovirales; Myoviridae.	1527469	Bacteria; Firmicutes; Bacilli; Bacillales; Bacillaceae; Bacillus; Bacillus cereus group
NC_020081	RefSeq	RVG	G657	149,844	41	Bacillus phage phiAGATE	Viruses; dsDNA viruses, no RNA stage; Caudovirales; Myoviridae.	1204533	Bacteria; Firmicutes; Bacilli; Bacillales; Bacillaceae; Bacillus
NC_021856	RefSeq	RVG	G657	155,631	42.1	Bacillus phage phiNIT1	Viruses; dsDNA viruses, no RNA stage; Caudovirales.	207656	Bacteria; Firmicutes; Bacilli; Bacillales; Bacillaceae; Bacillus; Bacillus subtilis group
NC_022771	RefSeq	RVG	G657	156,648	42.2	Bacillus phage Grass	Viruses; dsDNA viruses, no RNA stage; Caudovirales.	1406785	Bacteria; Firmicutes; Bacilli; Bacillales; Bacillaceae; Bacillus; Bacillus subtilis group
NC_027374	RefSeq	RVG	G657	161,239	40.1	Bacillus phage Moonbeam	Viruses; dsDNA viruses, no RNA stage; Caudovirales; Myoviridae.	1540091	Bacteria; Firmicutes; Bacilli; Bacillales; Bacillaceae; Bacillus
NC_027366	RefSeq	RVG	G657	164,302	39.5	Bacillus phage Mater	Viruses; dsDNA viruses, no RNA stage; Caudovirales; Myoviridae.	1540090	Bacteria; Firmicutes; Bacilli; Bacillales; Bacillaceae; Bacillus
NC_015253	RefSeq	RVG	G658	127,065	41.4	Brochothrix phage A9	Viruses; dsDNA viruses, no RNA stage; Caudovirales; Myoviridae; Spounavirinae; unclassified Spounavirinae.	857312	Bacteria; Firmicutes; Bacilli; Bacillales; Listeriaceae; Brochothrix
NC_012530	RefSeq	RVG	G659	142,111	37.4	Lactobacillus phage Lb338-1	Viruses; dsDNA viruses, no RNA stage; Caudovirales; Myoviridae.	632112	Bacteria; Firmicutes; Bacilli; Lactobacillales; Lactobacillaceae; Lactobacillus; Lactobacillus casei group
NC_006565	RefSeq	RVG	G660	131,522	37.3	Lactobacillus phage LP65	Viruses; dsDNA viruses, no RNA stage; Caudovirales; Myoviridae; Spounavirinae; unassigned Spounavirinae.	298338	Bacteria; Firmicutes; Bacilli; Lactobacillales; Lactobacillaceae; Lactobacillus
NC_029058	RefSeq	RVG	G661	106,071	38.2	Lactobacillus phage LfeInf	Viruses; dsDNA viruses, no RNA stage; Caudovirales; Myoviridae.	1567484	-
NC_028983	RefSeq	RVG	G662	138,877	40.8	Bacillus phage Shanette	Viruses; dsDNA viruses, no RNA stage; Caudovirales; Myoviridae.	1296656	-
NC_028982	RefSeq	RVG	G662	137,918	40.8	Bacillus phage JL	Viruses; dsDNA viruses, no RNA stage; Caudovirales; Myoviridae.	1296655	-
NC_025423	RefSeq	RVG	G662	138,658	40.9	Bacillus phage CP-51	Viruses; dsDNA viruses, no RNA stage; Caudovirales; Myoviridae; Spounavirinae; Spounalikevirus; unclassified SPO1-like viruses.	1391188	Bacteria; Firmicutes; Bacilli; Bacillales; Bacillaceae; Bacillus; Bacillus cereus group
NC_011421	RefSeq	RVG	G662	132,562	40	Bacillus phage SPO1	Viruses; dsDNA viruses, no RNA stage; Caudovirales; Myoviridae; Spounavirinae; Spounalikevirus.	10685	Bacteria; Firmicutes; Bacilli; Bacillales; Bacillaceae; Bacillus; Bacillus subtilis group
NC_022761	RefSeq	RVG	G662	146,193	40.2	Bacillus phage CampHawk	Viruses; dsDNA viruses, no RNA stage; Caudovirales; Myoviridae.	1406783	Bacteria; Firmicutes; Bacilli; Bacillales; Bacillaceae; Bacillus; Bacillus subtilis group
NC_019487	RefSeq	RVG	G663	143,986	40.5	Bacillus phage SP-10	Viruses; dsDNA viruses, no RNA stage; Caudovirales; Myoviridae.	941058	Bacteria; Firmicutes; Bacilli; Bacillales; Bacillaceae; Bacillus; Bacillus subtilis group
TARA_ERS488518_N000118	TOV	EVG	G664	86,525	48.5	-	-	-	-
TARA_ERS490494_N000005	TOV	EVG	G665	127,416	42.3	-	-	-	-
NC_027341	RefSeq	RVG	G666	130,008	32.4	Lactococcus phage WRP3	Viruses; dsDNA viruses, no RNA stage; Caudovirales; Siphoviridae.	1560313	Bacteria; Firmicutes; Bacilli; Lactobacillales; Streptococcaceae; Lactococcus
NC_023574	RefSeq	RVG	G666	128,546	32.5	Lactococcus phage phiL47	Viruses; dsDNA viruses, no RNA stage; Caudovirales; Siphoviridae.	1412875	Bacteria; Firmicutes; Bacilli; Lactobacillales; Streptococcaceae; Lactococcus; Lactococcus lactis
NC_015263	RefSeq	RVG	G666	114,768	32.7	Lactococcus phage 949	Viruses; dsDNA viruses, no RNA stage; Caudovirales; Siphoviridae.	881953	Bacteria; Firmicutes; Bacilli; Lactobacillales; Streptococcaceae; Lactococcus
NC_023007	RefSeq	RVG	G667	168,876	34.3	Bacillus phage vB_BanS-Tsamsa	Viruses; dsDNA viruses, no RNA stage; Caudovirales; Siphoviridae.	1308863	Bacteria; Firmicutes; Bacilli; Bacillales; Bacillaceae; Bacillus; Bacillus cereus group
NC_028749	RefSeq	RVG	G668	134,270	35.5	Brevibacillus phage Sundance	Viruses; dsDNA viruses, no RNA stage; Caudovirales; Siphoviridae.	1691958	-
NC_029073	RefSeq	RVG	G669	141,298	29.6	Geobacillus virus E3	Viruses; dsDNA viruses, no RNA stage; Caudovirales; Siphoviridae.	1572712	-
NC_007581	RefSeq	RVG	G670	185,683	26.3	Clostridium phage c-st	Viruses; dsDNA viruses, no RNA stage; Caudovirales; Myoviridae.	12336	Bacteria; Firmicutes; Clostridia; Clostridiales; Clostridiaceae; Clostridium
NC_029119	RefSeq	RVG	G671	127,726	30.5	Staphylococcus phage SPbeta-like	Viruses; dsDNA viruses, no RNA stage; Caudovirales; Siphoviridae; Spbetalikevirus; unclassified Spbetalikevirus.	1732063	-
NC_001884	RefSeq	RVG	G672	134,416	34.6	Bacillus phage SPbeta	Viruses; dsDNA viruses, no RNA stage; Caudovirales; Siphoviridae; Spbetalikevirus.	66797	Bacteria; Firmicutes; Bacilli; Bacillales; Bacillaceae; Bacillus; Bacillus subtilis group
NC_023582	RefSeq	RVG	G673	92,417	29.6	Staphylococcus phage vB_SepS_SEP9	Viruses; dsDNA viruses, no RNA stage; Caudovirales; Siphoviridae.	1434319	Bacteria; Firmicutes; Bacilli; Bacillales; Staphylococcaceae; Staphylococcus
NC_024355	RefSeq	RVG	G673	93,794	29.3	Staphylococcus phage 6ec	Viruses; dsDNA viruses, no RNA stage; Caudovirales; Siphoviridae.	1500386	Bacteria; Firmicutes; Bacilli; Bacillales; Staphylococcaceae; Staphylococcus
NC_022774	RefSeq	RVG	G674	80,382	35.2	Bacillus phage Slash	Viruses; dsDNA viruses, no RNA stage; Caudovirales; Siphoviridae.	1406790	Bacteria; Firmicutes; Bacilli; Bacillales; Bacillaceae; Bacillus
NC_022767	RefSeq	RVG	G674	81,656	35.3	Bacillus phage Staley	Viruses; dsDNA viruses, no RNA stage; Caudovirales; Siphoviridae.	1406792	Bacteria; Firmicutes; Bacilli; Bacillales; Bacillaceae; Bacillus
NC_028856	RefSeq	RVG	G674	80,148	35.3	Bacillus phage Stahl	Viruses; dsDNA viruses, no RNA stage; Caudovirales; Siphoviridae.	1610832	-
NC_028777	RefSeq	RVG	G674	80,798	35.5	Bacillus phage Stills	Viruses; dsDNA viruses, no RNA stage; Caudovirales; Siphoviridae.	1610833	-
KC595511	EBI	RVG	G674	82,008	33.9	Bacillus phage Basilisk	Viruses; dsDNA viruses, no RNA stage; Caudovirales; Siphoviridae.	1296654	-
NC_019515	RefSeq	RVG	G675	93,839	38	Bacillus phage BCD7	Viruses; dsDNA viruses, no RNA stage; Caudovirales; Myoviridae.	1136534	Bacteria; Firmicutes; Bacilli; Bacillales; Bacillaceae; Bacillus; Bacillus cereus group
NC_023719	RefSeq	RVG	G676	497,513	29.9	Bacillus phage G	Viruses; dsDNA viruses, no RNA stage; Caudovirales; Myoviridae.	1084719	Bacteria; Firmicutes; Bacilli; Bacillales; Bacillaceae; Bacillus
NC_019521	RefSeq	RVG	G677	219,372	31.3	Sphingomonas phage PAU	Viruses; dsDNA viruses, no RNA stage; Caudovirales; Myoviridae.	1150991	Bacteria; Proteobacteria; Alphaproteobacteria; Sphingomonadales; Sphingomonadaceae; Sphingomonas
NC_009760	RefSeq	RVG	G678	218,948	41.8	Bacillus phage 0305phi8-36	Viruses; dsDNA viruses, no RNA stage; Caudovirales; Myoviridae.	458639	Bacteria; Firmicutes; Bacilli; Bacillales; Bacillaceae; Bacillus; Bacillus cereus group
LDNN01000004	cryoconite	EVG	G679	74,172	46.9	-	-	-	-

NC_028805	RefSeq	RVG	G680	126,341	42.9	Brevibacillus phage Jenst	Viruses; dsDNA viruses, no RNA stage; Caudovirales; Siphoviridae.	1691954	-
NC_015937	RefSeq	RVG	G681	151,483	32.6	Thermus phage TMA	Viruses; dsDNA viruses, no RNA stage; Caudovirales; Myoviridae.	699370	Bacteria; Deinococcus-Thermus; Deinococci; Thermales; Thermaceae; Thermus
NC_008584	RefSeq	RVG	G681	152,372	32.6	Thermus phage phiYS40	Viruses; dsDNA viruses, no RNA stage; Caudovirales; Myoviridae.	407392	Bacteria; Deinococcus-Thermus; Deinococci; Thermales; Thermaceae; Thermus
JQ740814	EBI	RVG	G682	32,275	34.5	Lactococcus Phage ASCC544	Viruses; dsDNA viruses, no RNA stage; Caudovirales; Siphoviridae.	1165161	-
JQ740811	EBI	RVG	G682	32,345	34.5	Lactococcus Phage ASCC527	Viruses; dsDNA viruses, no RNA stage; Caudovirales; Siphoviridae.	1165158	-
JQ740809	EBI	RVG	G682	32,275	34.5	Lactococcus Phage ASCC502	Viruses; dsDNA viruses, no RNA stage; Caudovirales; Siphoviridae.	1165156	-
JQ740798	EBI	RVG	G682	32,276	34.5	Lactococcus Phage ASCC368	Viruses; dsDNA viruses, no RNA stage; Caudovirales; Siphoviridae.	1165145	-
JQ740794	EBI	RVG	G682	32,275	34.5	Lactococcus Phage ASCC337	Viruses; dsDNA viruses, no RNA stage; Caudovirales; Siphoviridae.	1165141	-
JQ740793	EBI	RVG	G682	32,277	34.5	Lactococcus Phage ASCC324	Viruses; dsDNA viruses, no RNA stage; Caudovirales; Siphoviridae.	1165140	-
JQ740806	EBI	RVG	G682	32,242	34.5	Lactococcus Phage ASCC476	Viruses; dsDNA viruses, no RNA stage; Caudovirales; Siphoviridae.	1165153	-
JQ740803	EBI	RVG	G682	32,242	34.5	Lactococcus Phage ASCC460	Viruses; dsDNA viruses, no RNA stage; Caudovirales; Siphoviridae.	1165150	-
JQ740810	EBI	RVG	G682	32,249	34.5	Lactococcus Phage ASCC506	Viruses; dsDNA viruses, no RNA stage; Caudovirales; Siphoviridae.	1165157	-
JQ740801	EBI	RVG	G682	32,180	34.5	Lactococcus Phage ASCC406	Viruses; dsDNA viruses, no RNA stage; Caudovirales; Siphoviridae.	1165148	-
JQ740800	EBI	RVG	G682	32,241	34.5	Lactococcus Phage ASCC397	Viruses; dsDNA viruses, no RNA stage; Caudovirales; Siphoviridae.	1165147	-
JQ740799	EBI	RVG	G682	32,239	34.5	Lactococcus Phage ASCC395	Viruses; dsDNA viruses, no RNA stage; Caudovirales; Siphoviridae.	1165146	-
JQ740788	EBI	RVG	G682	32,582	34.5	Lactococcus Phage ASCC273	Viruses; dsDNA viruses, no RNA stage; Caudovirales; Siphoviridae; Skunalikevirus.	1165135	-
JQ740802	EBI	RVG	G682	32,583	34.5	Lactococcus Phage ASCC454	Viruses; dsDNA viruses, no RNA stage; Caudovirales; Siphoviridae.	1165149	-
JQ740791	EBI	RVG	G682	32,581	34.5	Lactococcus Phage ASCC287	Viruses; dsDNA viruses, no RNA stage; Caudovirales; Siphoviridae.	1165138	-
NC_017688	RefSeq	RVG	G682	32,414	34.1	Lactococcus Phage ASCC191	Viruses; dsDNA viruses, no RNA stage; Caudovirales; Siphoviridae; Skunalikevirus.	1165134	-
FJ848885	EBI	RVG	G682	28,625	35	Lactococcus phage CB20	Viruses; dsDNA viruses, no RNA stage; Caudovirales; Siphoviridae; Skunalikevirus.	665887	-
FJ848884	EBI	RVG	G682	28,643	35.1	Lactococcus phage CB19	Viruses; dsDNA viruses, no RNA stage; Caudovirales; Siphoviridae; Skunalikevirus.	665886	-
NC_013152	RefSeq	RVG	G682	29,459	34.8	Lactococcus phage CB14	Viruses; dsDNA viruses, no RNA stage; Caudovirales; Siphoviridae; Skunalikevirus.	665884	-
NC_013155	RefSeq	RVG	G682	32,182	34.7	Lactococcus phage CB13	Viruses; dsDNA viruses, no RNA stage; Caudovirales; Siphoviridae; Skunalikevirus.	665883	-
KF676640	EBI	RVG	G682	28,452	34.5	Lactococcus phage SK1833	Viruses; dsDNA viruses, no RNA stage; Caudovirales; Siphoviridae; Skunalikevirus.	1414741	-
NC_001835	RefSeq	RVG	G682	28,451	34.5	Lactococcus phage SK1	Viruses; dsDNA viruses, no RNA stage; Caudovirales; Siphoviridae; Skunalikevirus.	31532	Bacteria; Firmicutes; Bacilli; Lactobacillales; Streptococcaceae; Lactococcus
NC_008371	RefSeq	RVG	G682	27,453	34.9	Lactococcus phage jj50	Viruses; dsDNA viruses, no RNA stage; Caudovirales; Siphoviridae; Skunalikevirus.	213775	Bacteria; Firmicutes; Bacilli; Lactobacillales; Streptococcaceae; Lactococcus
KC182544	EBI	RVG	G682	27,302	34.5	Lactococcus phage 936	Viruses; dsDNA viruses, no RNA stage; Caudovirales; Siphoviridae.	39838	-
NC_021854	RefSeq	RVG	G682	28,674	34.4	Lactococcus phage jm3	Viruses; dsDNA viruses, no RNA stage; Caudovirales; Siphoviridae.	1262536	Bacteria; Firmicutes; Bacilli; Lactobacillales; Streptococcaceae; Lactococcus; Lactococcus lactis
KC522412	EBI	RVG	G682	30,692	34.8	Lactococcus phage CaseusJM1	Viruses; dsDNA viruses, no RNA stage; Caudovirales; Siphoviridae.	1289467	-
NC_021855	RefSeq	RVG	G682	32,382	34.2	Lactococcus phage phi7	Viruses; dsDNA viruses, no RNA stage; Caudovirales; Siphoviridae.	1262538	Bacteria; Firmicutes; Bacilli; Lactobacillales; Streptococcaceae; Lactococcus; Lactococcus lactis
NC_028900	RefSeq	RVG	G682	28,144	35	Lactococcus phage SL4	Viruses; dsDNA viruses, no RNA stage; Caudovirales; Siphoviridae; Skunalikevirus.	287412	-
KC182545	EBI	RVG	G682	30,674	34.7	Lactococcus phage fd13	Viruses; dsDNA viruses, no RNA stage; Caudovirales; Siphoviridae.	213782	-
KM091442	EBI	RVG	G682	31,945	34.6	Lactococcus phage phi15	Viruses; dsDNA viruses, no RNA stage; Caudovirales; Siphoviridae.	1527693	-
NC_021860	RefSeq	RVG	G682	31,090	34.3	Lactococcus phage jm2	Viruses; dsDNA viruses, no RNA stage; Caudovirales; Siphoviridae.	1262535	Bacteria; Firmicutes; Bacilli; Lactobacillales; Streptococcaceae; Lactococcus; Lactococcus lactis
JQ740789	EBI	RVG	G682	32,023	34.8	Lactococcus Phage ASCC281	Viruses; dsDNA viruses, no RNA stage; Caudovirales; Siphoviridae; Skunalikevirus.	1165136	-
JQ740796	EBI	RVG	G682	32,052	34.8	Lactococcus Phage ASCC358	Viruses; dsDNA viruses, no RNA stage; Caudovirales; Siphoviridae.	1165143	-
JQ740797	EBI	RVG	G682	32,048	34.8	Lactococcus Phage ASCC365	Viruses; dsDNA viruses, no RNA stage; Caudovirales; Siphoviridae.	1165144	-
JQ740795	EBI	RVG	G682	32,545	34.7	Lactococcus Phage ASCC356	Viruses; dsDNA viruses, no RNA stage; Caudovirales; Siphoviridae.	1165142	-
JQ740792	EBI	RVG	G682	32,546	34.8	Lactococcus Phage ASCC310	Viruses; dsDNA viruses, no RNA stage; Caudovirales; Siphoviridae.	1165139	-
JQ740790	EBI	RVG	G682	33,178	34.7	Lactococcus Phage ASCC284	Viruses; dsDNA viruses, no RNA stage; Caudovirales; Siphoviridae.	1165137	-
JQ740804	EBI	RVG	G682	31,772	34.6	Lactococcus Phage ASCC465	Viruses; dsDNA viruses, no RNA stage; Caudovirales; Siphoviridae; Skunalikevirus.	1165151	-
JQ740812	EBI	RVG	G682	31,773	34.6	Lactococcus Phage ASCC531	Viruses; dsDNA viruses, no RNA stage; Caudovirales; Siphoviridae.	1165159	-
JQ740805	EBI	RVG	G682	31,772	34.6	Lactococcus Phage ASCC473	Viruses; dsDNA viruses, no RNA stage; Caudovirales; Siphoviridae.	1165152	-
JQ740808	EBI	RVG	G682	31,774	34.6	Lactococcus Phage ASCC497	Viruses; dsDNA viruses, no RNA stage; Caudovirales; Siphoviridae.	1165155	-
JQ740807	EBI	RVG	G682	30,876	34.9	Lactococcus Phage ASCC489	Viruses; dsDNA viruses, no RNA stage; Caudovirales; Siphoviridae.	1165154	-
JQ740813	EBI	RVG	G682	32,384	34.7	Lactococcus Phage ASCC532	Viruses; dsDNA viruses, no RNA stage; Caudovirales; Siphoviridae; Skunalikevirus.	1165160	-
KC182550	EBI	RVG	G682	30,961	34.3	Lactococcus lactis phage P475	Viruses; dsDNA viruses, no RNA stage; Caudovirales; Siphoviridae.	213774	-
NC_011046	RefSeq	RVG	G682	29,305	34.7	Lactococcus phage bIBB29	Viruses; dsDNA viruses, no RNA stage; Caudovirales; Siphoviridae; Skunalikevirus.	494269	Bacteria; Firmicutes; Bacilli; Lactobacillales; Streptococcaceae; Lactococcus
KC182549	EBI	RVG	G682	30,778	34.1	Lactococcus lactis phage p272	Viruses; dsDNA viruses, no RNA stage; Caudovirales; Siphoviridae.	213777	-
KC182548	EBI	RVG	G682	30,796	34.1	Lactococcus lactis phage P113G	Viruses; dsDNA viruses, no RNA stage; Caudovirales; Siphoviridae.	213773	-
NC_008363	RefSeq	RVG	G682	28,538	34.7	Lactococcus phage P008	Viruses; dsDNA viruses, no RNA stage; Caudovirales; Siphoviridae; Skunalikevirus.	83129	Bacteria; Firmicutes; Bacilli; Lactobacillales; Streptococcaceae; Lactococcus; Lactococcus lactis
NC_001909	RefSeq	RVG	G682	31,754	34.3	Lactococcus phage bIL170	Viruses; dsDNA viruses, no RNA stage; Caudovirales; Siphoviridae; Skunalikevirus.	63118	Bacteria; Firmicutes; Bacilli; Lactobacillales; Streptococcaceae; Lactococcus
KC182543	EBI	RVG	G682	29,247	35	Lactococcus lactis phage 645	Viruses; dsDNA viruses, no RNA stage; Caudovirales; Siphoviridae.	270182	-
NC_021853	RefSeq	RVG	G682	32,337	34.5	Lactococcus phage 340	Viruses; dsDNA viruses, no RNA stage; Caudovirales; Siphoviridae.	1262533	Bacteria; Firmicutes; Bacilli; Lactobacillales; Streptococcaceae; Lactococcus; Lactococcus lactis

NC_021852	RefSeq	RVG	G682	29,631	35.1	Lactococcus phage P680	Viruses; dsDNA viruses, no RNA stage; Caudovirales; Siphoviridae.	1262537	Bacteria; Firmicutes; Bacilli; Lactobacillales; Streptococcaceae; Lactococcus; Lactococcus lactis
KM091444	EBI	RVG	G682	30,862	34.9	Lactococcus phage phi145	Viruses; dsDNA viruses, no RNA stage; Caudovirales; Siphoviridae.	1527692	-
KM091443	EBI	RVG	G682	31,841	35	Lactococcus phage phi93	Viruses; dsDNA viruses, no RNA stage; Caudovirales; Siphoviridae.	1527694	-
NC_008370	RefSeq	RVG	G682	30,510	33.9	Lactococcus phage 712	Viruses; dsDNA viruses, no RNA stage; Caudovirales; Siphoviridae; Skunalikevirus.	213769	Bacteria; Firmicutes; Bacilli; Lactobacillales; Streptococcaceae; Lactococcus
NC_001629	RefSeq	RVG	G683	22,195	36	Lactococcus phage bIL67	Viruses; dsDNA viruses, no RNA stage; Caudovirales; Siphoviridae; C2likevirus.	36343	Bacteria; Firmicutes; Bacilli; Lactobacillales; Streptococcaceae
NC_001706	RefSeq	RVG	G683	22,172	36.3	Lactococcus phage c2	Viruses; dsDNA viruses, no RNA stage; Caudovirales; Siphoviridae; C2likevirus.	31537	Bacteria; Firmicutes; Bacilli; Lactobacillales; Streptococcaceae; Lactococcus
NC_029118	RefSeq	RVG	G684	24,847	37.9	Lactococcus phage GE1	Viruses; dsDNA viruses, no RNA stage; Caudovirales; Siphoviridae.	1698369	-
NC_008364	RefSeq	RVG	G685	26,537	37.1	Lactococcus phage Q54	Viruses; dsDNA viruses, no RNA stage; Caudovirales; Siphoviridae.	382685	Bacteria; Firmicutes; Bacilli; Lactobacillales; Streptococcaceae; Lactococcus
NC_012663	RefSeq	RVG	G686	60,074	34.4	Lactococcus phage P087	Viruses; dsDNA viruses, no RNA stage; Caudovirales; Siphoviridae.	641487	Bacteria; Firmicutes; Bacilli; Lactobacillales; Streptococcaceae; Lactococcus
NC_027987	RefSeq	RVG	G687	29,508	36.1	Leuconostoc phage 1-A4	Viruses; dsDNA viruses, no RNA stage; Caudovirales; Siphoviridae.	745088	Bacteria; Firmicutes; Bacilli; Lactobacillales; Leuconostocaceae; Leuconostoc
NC_027358	RefSeq	RVG	G687	28,527	36.4	Leuconostoc phage Ln-9	Viruses; dsDNA viruses, no RNA stage; Caudovirales; Siphoviridae.	1536605	Bacteria; Firmicutes; Bacilli; Lactobacillales; Leuconostocaceae; Leuconostoc; Leuconostoc mesenteroides
NC_027377	RefSeq	RVG	G687	28,921	36.1	Leuconostoc phage Ln-8	Viruses; dsDNA viruses, no RNA stage; Caudovirales; Siphoviridae.	1536604	Bacteria; Firmicutes; Bacilli; Lactobacillales; Leuconostocaceae; Leuconostoc; Leuconostoc mesenteroides
NC_024386	RefSeq	RVG	G687	28,427	36.2	Leuconostoc phage phiLN25	Viruses; dsDNA viruses, no RNA stage; Caudovirales; Siphoviridae.	1262518	Bacteria; Firmicutes; Bacilli; Lactobacillales; Leuconostocaceae; Leuconostoc
NC_024389	RefSeq	RVG	G687	28,336	36	Leuconostoc phage phiLNTR2	Viruses; dsDNA viruses, no RNA stage; Caudovirales; Siphoviridae.	1262523	Bacteria; Firmicutes; Bacilli; Lactobacillales; Leuconostocaceae; Leuconostoc
NC_024378	RefSeq	RVG	G687	28,015	36.1	Leuconostoc phage phiLNTR3	Viruses; dsDNA viruses, no RNA stage; Caudovirales; Siphoviridae.	1262521	Bacteria; Firmicutes; Bacilli; Lactobacillales; Leuconostocaceae; Leuconostoc
NC_024388	RefSeq	RVG	G687	28,022	36	Leuconostoc phage phiLN34	Viruses; dsDNA viruses, no RNA stage; Caudovirales; Siphoviridae.	1262519	Bacteria; Firmicutes; Bacilli; Lactobacillales; Leuconostocaceae; Leuconostoc
NC_024390	RefSeq	RVG	G687	26,752	36.8	Leuconostoc phage phiLN03	Viruses; dsDNA viruses, no RNA stage; Caudovirales; Siphoviridae.	1262515	Bacteria; Firmicutes; Bacilli; Lactobacillales; Leuconostocaceae; Leuconostoc
NC_024385	RefSeq	RVG	G687	28,193	36.6	Leuconostoc phage phiLN12	Viruses; dsDNA viruses, no RNA stage; Caudovirales; Siphoviridae.	1262517	Bacteria; Firmicutes; Bacilli; Lactobacillales; Leuconostocaceae; Leuconostoc
NC_020870	RefSeq	RVG	G687	25,865	36.6	Leuconostoc phage phiLN04	Viruses; dsDNA viruses, no RNA stage; Caudovirales; Siphoviridae.	1262516	Bacteria; Firmicutes; Bacilli; Lactobacillales; Leuconostocaceae; Leuconostoc
NC_018273	RefSeq	RVG	G687	26,201	36.4	Leuconostoc phage Lmd1	Viruses; dsDNA viruses, no RNA stage; Caudovirales; Siphoviridae.	1161930	Bacteria; Firmicutes; Bacilli; Lactobacillales; Leuconostocaceae; Leuconostoc; Leuconostoc mesenteroides
NC_024380	RefSeq	RVG	G687	25,740	36.5	Leuconostoc phage phiLN6B	Viruses; dsDNA viruses, no RNA stage; Caudovirales; Siphoviridae.	1262520	Bacteria; Firmicutes; Bacilli; Lactobacillales; Leuconostocaceae; Leuconostoc
NC_020880	RefSeq	RVG	G687	26,769	36.6	Leuconostoc phage P793	Viruses; dsDNA viruses, no RNA stage; Caudovirales; Siphoviridae.	1262522	Bacteria; Firmicutes; Bacilli; Lactobacillales; Leuconostocaceae; Leuconostoc
NC_024215	RefSeq	RVG	G688	54,452	33.5	Lactococcus phage P078	Viruses; dsDNA viruses, no RNA stage; Caudovirales; Siphoviridae.	1476886	Bacteria; Firmicutes; Bacilli; Lactobacillales; Streptococcaceae; Lactococcus
NC_024203	RefSeq	RVG	G688	53,513	33.5	Lactococcus phage P092	Viruses; dsDNA viruses, no RNA stage; Caudovirales; Siphoviridae.	1476887	Bacteria; Firmicutes; Bacilli; Lactobacillales; Streptococcaceae; Lactococcus
NC_024208	RefSeq	RVG	G688	54,173	33.5	Lactococcus phage P118	Viruses; dsDNA viruses, no RNA stage; Caudovirales; Siphoviridae.	1476888	Bacteria; Firmicutes; Bacilli; Lactobacillales; Streptococcaceae; Lactococcus
NC_024214	RefSeq	RVG	G688	55,584	33.3	Lactococcus phage P162	Viruses; dsDNA viruses, no RNA stage; Caudovirales; Siphoviridae.	1476889	Bacteria; Firmicutes; Bacilli; Lactobacillales; Streptococcaceae; Lactococcus
NC_010576	RefSeq	RVG	G688	55,597	33.7	Lactococcus phage 1706	Viruses; dsDNA viruses, no RNA stage; Caudovirales; Siphoviridae.	475178	Bacteria; Firmicutes; Bacilli; Lactobacillales; Streptococcaceae; Lactococcus
NC_023735	RefSeq	RVG	G689	76,797	53.4	Rhodococcus phage ReqiPepy6	Viruses; dsDNA viruses, no RNA stage; Caudovirales; Siphoviridae.	691965	Bacteria; Actinobacteria; Actinobacteria; Corynebacteriales; Nocardiaceae; Rhodococcus
NC_023694	RefSeq	RVG	G689	78,064	53.3	Rhodococcus phage ReqiPoco6	Viruses; dsDNA viruses, no RNA stage; Caudovirales; Siphoviridae.	691964	Bacteria; Actinobacteria; Actinobacteria; Corynebacteriales; Nocardiaceae; Rhodococcus
M11813	EBI	RVG	G690	19,366	39.7	Bacillus phage PZA	Viruses; dsDNA viruses, no RNA stage; Caudovirales; Podoviridae; Picovirinae; Phi29likevirus.	10757	-
NC_011048	RefSeq	RVG	G690	19,282	40	Bacillus phage phi29	Viruses; dsDNA viruses, no RNA stage; Caudovirales; Podoviridae; Picovirinae; Phi29likevirus.	10756	Bacteria; Firmicutes; Bacilli; Bacillales; Bacillaceae; Bacillus; Bacillus subtilis group
EU622808	EBI	RVG	G690	18,753	37.3	Bacillus phage Nf	Viruses; dsDNA viruses, no RNA stage; Caudovirales; Podoviridae; Picovirinae; Phi29likevirus.	10753	-
NC_004165	RefSeq	RVG	G690	18,630	37.7	Bacillus phage B103	Viruses; dsDNA viruses, no RNA stage; Caudovirales; Podoviridae; Picovirinae; Phi29likevirus.	10778	Bacteria; Firmicutes; Bacilli; Bacillales; Bacillaceae; Bacillus; Bacillus subtilis group
NC_028789	RefSeq	RVG	G690	18,609	36.4	Bacillus phage VMY22	Viruses; dsDNA viruses, no RNA stage; Caudovirales; Podoviridae.	1734382	-
NC_002649	RefSeq	RVG	G690	21,129	34.7	Bacillus phage GA-1	Viruses; dsDNA viruses, no RNA stage; Caudovirales; Podoviridae; Picovirinae; Phi29likevirus.	12345	Bacteria; Firmicutes; Bacilli; Bacillales; Bacillaceae; Bacillus; Bacillus subtilis group
NC_021336	RefSeq	RVG	G690	27,190	30.7	Bacillus phage MG-B1	Viruses; dsDNA viruses, no RNA stage; Caudovirales; Podoviridae.	1309583	Bacteria; Firmicutes; Bacilli; Bacillales; Bacillaceae; Bacillus; Bacillus cereus group
NC_009643	RefSeq	RVG	G691	17,171	49.5	Actinomyces phage Av-1	Viruses; dsDNA viruses, no RNA stage; Caudovirales; Podoviridae; Picovirinae; unassigned Picovirinae.	338473	Bacteria; Actinobacteria; Actinobacteria; Actinomycetales; Actinomycetaceae; Actinomyces
KJ617393	EBI	RVG	G692	19,347	38.8	Streptococcus phage SOCP	Viruses; dsDNA viruses, no RNA stage; Caudovirales; Podoviridae; Picovirinae; unassigned Picovirinae.	1498213	-
NC_001825	RefSeq	RVG	G692	19,343	38.8	Streptococcus phage Cp-1	Viruses; dsDNA viruses, no RNA stage; Caudovirales; Podoviridae; Picovirinae; unassigned Picovirinae.	10747	Bacteria; Firmicutes; Bacilli; Lactobacillales; Streptococcaceae; Streptococcus
LK392619	EBI	RVG	G692	19,741	38.5	Streptococcus phage CP-7	Viruses; dsDNA viruses, no RNA stage; Caudovirales; Podoviridae; Picovirinae; unassigned Picovirinae.	10748	-
NC_018083	RefSeq	RVG	G693	17,972	34.5	Clostridium phage phiCPV4	Viruses; dsDNA viruses, no RNA stage; Caudovirales; Podoviridae; Picovirinae; unclassified Picovirinae.	1162305	Bacteria; Firmicutes; Clostridia; Clostridiales; Clostridiaceae; Clostridium
NC_017980	RefSeq	RVG	G693	18,397	34.8	Clostridium phage phiCP7R	Viruses; dsDNA viruses, no RNA stage; Caudovirales; Podoviridae; Picovirinae; unclassified Picovirinae.	1162304	Bacteria; Firmicutes; Clostridia; Clostridiales; Clostridiaceae; Clostridium
NC_018084	RefSeq	RVG	G693	18,078	34.5	Clostridium phage phiZP2	Viruses; dsDNA viruses, no RNA stage; Caudovirales; Podoviridae; Picovirinae; unclassified Picovirinae.	1162306	Bacteria; Firmicutes; Clostridia; Clostridiales; Clostridiaceae; Clostridium
NC_019523	RefSeq	RVG	G693	18,919	27.8	Clostridium phage phi24R	Viruses; dsDNA viruses, no RNA stage; Caudovirales; Podoviridae; Picovirinae; unassigned Picovirinae.	1128071	Bacteria; Firmicutes; Clostridia; Clostridiales; Clostridiaceae; Clostridium
NC_018270	RefSeq	RVG	G694	33,594	43.9	Weissella phage phiYS61	Viruses; dsDNA viruses, no RNA stage; Caudovirales; Podoviridae.	1161906	Bacteria; Firmicutes; Bacilli; Lactobacillales; Leuconostocaceae; Weissella
NC_010363	RefSeq	RVG	G695	18,762	33.7	Lactococcus phage asccphi28	Viruses; dsDNA viruses, no RNA stage; Caudovirales; Podoviridae.	503388	Bacteria; Firmicutes; Bacilli; Lactobacillales; Streptococcaceae; Lactococcus
NC_009817	RefSeq	RVG	G696	79,232	35.1	Lactococcus phage KSY1	Viruses; dsDNA viruses, no RNA stage; Caudovirales; Podoviridae.	388452	Bacteria; Firmicutes; Bacilli; Lactobacillales; Streptococcaceae; Lactococcus
NC_004678	RefSeq	RVG	G697	16,784	29.6	Staphylococcus phage 44AHJD	Viruses; dsDNA viruses, no RNA stage; Caudovirales; Podoviridae; Picovirinae; Ahjdlikevirus.	204086	Bacteria; Firmicutes; Bacilli; Bacillales; Staphylococcaceae; Staphylococcus
NC_004679	RefSeq	RVG	G697	18,227	29.3	Staphylococcus phage P68	Viruses; dsDNA viruses, no RNA stage; Caudovirales; Podoviridae; Picovirinae; Ahjdlikevirus; unclassified AHJD-like viruses.	204090	Bacteria; Firmicutes; Bacilli; Bacillales; Staphylococcaceae; Staphylococcus

NC_007046	RefSeq	RVG	G697	18,199	29.3	Staphylococcus phage 66	Viruses; dsDNA viruses, no RNA stage; Caudovirales; Podoviridae; Picovirinae; Ahjdlikevirus; unclassified AHJD-like viruses.	320832	Bacteria; Firmicutes; Bacilli; Bacillales; Staphylococcaceae; Staphylococcus
AB626963	EBI	RVG	G697	18,186	29.2	Staphylococcus phage S13'	Viruses; dsDNA viruses, no RNA stage; Caudovirales; Podoviridae; Picovirinae; unclassified Picovirinae.	1010615	-
NC_016565	RefSeq	RVG	G697	18,168	28.9	Staphylococcus phage S24-1	Viruses; dsDNA viruses, no RNA stage; Caudovirales; Podoviridae; Picovirinae; unclassified Picovirinae.	1010614	Bacteria; Firmicutes; Bacilli; Bacillales; Staphylococcaceae; Staphylococcus
NC_009875	RefSeq	RVG	G697	17,938	28.9	Staphylococcus phage SAP-2	Viruses; dsDNA viruses, no RNA stage; Caudovirales; Podoviridae; Picovirinae; Ahjdlikevirus; unclassified AHJD-like viruses.	470865	Bacteria; Firmicutes; Bacilli; Bacillales; Staphylococcaceae; Staphylococcus
NC_023550	RefSeq	RVG	G697	17,869	28.9	Staphylococcus phage GRCS	Viruses; dsDNA viruses, no RNA stage; Caudovirales; Podoviridae; Picovirinae; Ahjdlikevirus; unclassified AHJD-like viruses.	1453367	Bacteria; Firmicutes; Bacilli; Bacillales; Staphylococcaceae; Staphylococcus
NC_028693	RefSeq	RVG	G697	18,607	33.2	Enterococcus phage vB_IME195	Viruses; dsDNA viruses, no RNA stage; Caudovirales; Podoviridae.	1747288	-
NC_025467	RefSeq	RVG	G697	17,972	32.8	Enterococcus phage vB_Efae230P-4	Viruses; dsDNA viruses, no RNA stage; Caudovirales; Podoviridae.	1161939	Bacteria; Firmicutes; Bacilli; Lactobacillales; Enterococcaceae; Enterococcus
NC_004814	RefSeq	RVG	G697	16,687	34.6	Streptococcus phage C1	Viruses; dsDNA viruses, no RNA stage; Caudovirales; Podoviridae; Picovirinae; Ahjdlikevirus.	230871	Bacteria; Firmicutes; Bacilli; Lactobacillales; Streptococcaceae; Streptococcus
NC_024149	RefSeq	RVG	G698	18,899	31.1	Lactococcus phage WP-2	Viruses; dsDNA viruses, no RNA stage; Caudovirales; Podoviridae.	1486423	Bacteria; Firmicutes; Bacilli; Lactobacillales; Streptococcaceae; Lactococcus
NC_017732	RefSeq	RVG	G699	30,505	32.7	Enterococcus phage EF62phi	Viruses; unclassified phages.	977801	Bacteria; Firmicutes; Bacilli; Lactobacillales; Enterococcaceae; Enterococcus
NC_022094	RefSeq	RVG	G700	14,319	36.8	Bacillus phage Wip1	Viruses; dsDNA viruses, no RNA stage; Tectiviridae; Tectivirus; unclassified Tectivirus.	663237	Bacteria; Firmicutes; Bacilli; Bacillales; Bacillaceae; Bacillus; Bacillus cereus group
NC_011523	RefSeq	RVG	G700	14,398	38.7	Bacillus phage AP50	Viruses; dsDNA viruses, no RNA stage; Tectiviridae; Tectivirus.	513550	Bacteria; Firmicutes; Bacilli; Bacillales; Bacillaceae; Bacillus; Bacillus cereus group
NC_006945	RefSeq	RVG	G700	14,844	40.1	Bacillus phage GIL16c	Viruses; dsDNA viruses, no RNA stage; Tectiviridae; Tectivirus; unclassified Tectivirus.	307240	Bacteria; Firmicutes; Bacilli; Bacillales; Bacillaceae; Bacillus; Bacillus cereus group
NC_005258	RefSeq	RVG	G700	14,935	39.7	Bacillus phage Bam35c	Viruses; dsDNA viruses, no RNA stage; Tectiviridae; Tectivirus.	236750	Bacteria; Firmicutes; Bacilli; Bacillales; Bacillaceae; Bacillus; Bacillus cereus group
NC_010152	RefSeq	RVG	G701	39,577	36.9	Acidianus filamentous virus 6	Viruses; dsDNA viruses, no RNA stage; Ligamenvirales; Lipothrixviridae; Betalipothrixvirus.	346882	Archaea; Crenarchaeota; Thermoprotei; Sulfolobales; Sulfolobaceae; Acidianus
NC_010155	RefSeq	RVG	G701	40,449	36.9	Acidianus filamentous virus 3	Viruses; dsDNA viruses, no RNA stage; Ligamenvirales; Lipothrixviridae; Betalipothrixvirus.	346881	Archaea; Crenarchaeota; Thermoprotei; Sulfolobales; Sulfolobaceae; Acidianus
NC_010154	RefSeq	RVG	G701	38,179	36.8	Acidianus filamentous virus 8	Viruses; dsDNA viruses, no RNA stage; Ligamenvirales; Lipothrixviridae; Betalipothrixvirus.	346884	Archaea; Crenarchaeota; Thermoprotei; Sulfolobales; Sulfolobaceae; Acidianus
NC_010153	RefSeq	RVG	G701	36,895	38	Acidianus filamentous virus 7	Viruses; dsDNA viruses, no RNA stage; Ligamenvirales; Lipothrixviridae; Betalipothrixvirus.	346883	Archaea; Crenarchaeota; Thermoprotei; Sulfolobales; Sulfolobaceae; Acidianus
NC_010537	RefSeq	RVG	G701	41,172	34.6	Acidianus filamentous virus 9	Viruses; dsDNA viruses, no RNA stage; Ligamenvirales; Lipothrixviridae; Betalipothrixvirus.	512792	Archaea; Crenarchaeota; Thermoprotei; Sulfolobales; Sulfolobaceae
NC_003214	RefSeq	RVG	G701	40,900	33.4	Sulfolobus islandicus filamentous virus	Viruses; dsDNA viruses, no RNA stage; Ligamenvirales; Lipothrixviridae; Betalipothrixvirus.	176106	Archaea; Crenarchaeota; Thermoprotei; Sulfolobales; Sulfolobaceae; Sulfolobus
NC_009884	RefSeq	RVG	G702	31,787	35.7	Acidianus filamentous virus 2	Viruses; dsDNA viruses, no RNA stage; Ligamenvirales; Lipothrixviridae; Deltalipothrixvirus.	300186	Archaea; Crenarchaeota; Thermoprotei; Sulfolobales; Sulfolobaceae; Acidianus
NC_005830	RefSeq	RVG	G703	20,869	36.9	Acidianus filamentous virus 1	Viruses; dsDNA viruses, no RNA stage; Ligamenvirales; Lipothrixviridae; Gammalipothrixvirus.	235266	Archaea; Crenarchaeota; Thermoprotei; Sulfolobales; Sulfolobaceae; Acidianus
AJ748296	EBI	RVG	G704	33,641	25.3	Sulfolobus islandicus rudivirus 1 variant XX	Viruses; dsDNA viruses, no RNA stage; Ligamenvirales; Rudiviridae; Rudivirus.	282066	-
NC_004087	RefSeq	RVG	G704	32,308	25.3	Sulfolobus islandicus rod-shaped virus 1	Viruses; dsDNA viruses, no RNA stage; Ligamenvirales; Rudiviridae; Rudivirus.	157898	Archaea; Crenarchaeota; Thermoprotei; Sulfolobales; Sulfolobaceae; Sulfolobus
NC_004086	RefSeq	RVG	G704	35,450	25.2	Sulfolobus islandicus rod-shaped virus 2	Viruses; dsDNA viruses, no RNA stage; Ligamenvirales; Rudiviridae; Rudivirus.	157899	Archaea; Crenarchaeota; Thermoprotei; Sulfolobales; Sulfolobaceae; Sulfolobus
NC_025375	RefSeq	RVG	G704	28,096	29.3	Stygiolobus rod-shaped virus	Viruses; dsDNA viruses, no RNA stage; Ligamenvirales; Rudiviridae; Rudivirus; unclassified Rudivirus.	537009	Archaea; Crenarchaeota; Thermoprotei; Sulfolobales; Sulfolobaceae
NC_009965	RefSeq	RVG	G704	24,655	39.1	Acidianus rod-shaped virus 1	Viruses; dsDNA viruses, no RNA stage; Ligamenvirales; Rudiviridae; Rudivirus.	309181	Archaea; Crenarchaeota; Thermoprotei; Sulfolobales; Sulfolobaceae; Acidianus
NC_029314	RefSeq	RVG	G704	29,763	32.3	Acidianus rod-shaped virus 2	Viruses; dsDNA viruses, no RNA stage; Ligamenvirales; Rudiviridae; Rudivirus; unclassified Rudivirus.	1732175	-
NC_019413	RefSeq	RVG	G704	27,431	46.6	Sulfolobales Mexican rudivirus 1	Viruses; dsDNA viruses, no RNA stage; Ligamenvirales; Rudiviridae; Rudivirus; unclassified Rudivirus.	1245909	Archaea; Crenarchaeota; Thermoprotei
NC_014099	RefSeq	RVG	G705	16,622	36.7	Sulfolobus turreted icosahedral virus 2	Viruses; dsDNA viruses, no RNA stage; Turriviridae; Alphaturrivirus.	754004	Archaea; Crenarchaeota; Thermoprotei; Sulfolobales; Sulfolobaceae; Sulfolobus
NC_005892	RefSeq	RVG	G705	17,663	36	Sulfolobus turreted icosahedral virus 1	Viruses; dsDNA viruses, no RNA stage; Turriviridae; Alphaturrivirus.	269145	Archaea; Crenarchaeota; Thermoprotei; Sulfolobales; Sulfolobaceae; Sulfolobus
NC_028651	RefSeq	RVG	G706	19,351	38.2	Hydrogenobaculum phage 1	Viruses; dsDNA viruses, no RNA stage; unclassified dsDNA viruses.	1732176	-
NC_029011	RefSeq	RVG	G707	51,711	38.6	Sulfolobus monocaudavirus SMV4	Viruses; dsDNA viruses, no RNA stage; unclassified dsDNA viruses.	1732178	-
NC_023585	RefSeq	RVG	G707	48,775	38.4	Sulfolobus monocaudavirus SMV1	Viruses; dsDNA viruses, no RNA stage; unclassified dsDNA viruses.	1351702	Archaea; Crenarchaeota; Thermoprotei; Sulfolobales; Sulfolobaceae; Sulfolobus
NC_029103	RefSeq	RVG	G707	64,323	38.4	Sulfolobus monocaudavirus SMV3	Viruses; dsDNA viruses, no RNA stage; unclassified dsDNA viruses.	1732177	-
NC_029020	RefSeq	RVG	G707	50,918	43.6	Sulfolobus monocaudavirus SMV2	Viruses; dsDNA viruses, no RNA stage; unclassified dsDNA viruses.	1580591	-
NC_029316	RefSeq	RVG	G708	70,812	36.4	Acidianus tailed spindle virus	Viruses; dsDNA viruses, no RNA stage; Bicaudaviridae; unclassified Bicaudaviridae.	1797140	-
NC_007409	RefSeq	RVG	G709	62,730	41.2	Acidianus two-tailed virus	Viruses; dsDNA viruses, no RNA stage; Bicaudaviridae; Bicaudavirus.	315953	Archaea; Crenarchaeota; Thermoprotei; Sulfolobales; Sulfolobaceae; Acidianus
NC_029027	RefSeq	RVG	G710	40,860	38.3	Sulfolobales virus YNP1	Viruses; dsDNA viruses, no RNA stage; unclassified dsDNA viruses.	1732179	-
NC_028992	RefSeq	RVG	G710	34,783	42.7	Sulfolobales Virus YNP2	Viruses; dsDNA viruses, no RNA stage; unclassified dsDNA viruses.	1732180	-
NC_006268	RefSeq	RVG	G711	75,294	35.2	Sulfolobus virus STSV1	Viruses; dsDNA viruses, no RNA stage; Bicaudaviridae; unclassified Bicaudaviridae.	285013	Archaea; Crenarchaeota; Thermoprotei; Sulfolobales; Sulfolobaceae; Sulfolobus
NC_020077	RefSeq	RVG	G711	76,107	35.1	Sulfolobus virus STSV2	Viruses; dsDNA viruses, no RNA stage; Bicaudaviridae; unclassified Bicaudaviridae.	1123964	Archaea; Crenarchaeota; Thermoprotei; Sulfolobales; Sulfolobaceae
NC_014321	RefSeq	RVG	G712	17,666	52.1	Hyperthermophilic Archaeal Virus 2	Viruses; unclassified archaeal viruses.	762906	Archaea
NC_011217	RefSeq	RVG	G713	15,330	38.5	Sulfolobus spindle-shaped virus 5	Viruses; dsDNA viruses, no RNA stage; Fuselloviridae; Alphafusellovirus.	459291	Archaea; Crenarchaeota; Thermoprotei; Sulfolobales; Sulfolobaceae; Sulfolobus
NC_009986	RefSeq	RVG	G713	15,135	38.5	Sulfolobus spindle-shaped virus 4	Viruses; dsDNA viruses, no RNA stage; Fuselloviridae; Alphafusellovirus.	459290	Archaea; Crenarchaeota; Thermoprotei; Sulfolobales; Sulfolobaceae; Sulfolobus

NC_005265	RefSeq	RVG	G713	14,796	38.5	Sulfolobus spindle-shaped virus 2	Viruses; dsDNA viruses, no RNA stage; Fuselloviridae; Alphafusellovirus.	244590	Archaea; Crenarchaeota; Thermoprotei; Sulfolobales; Sulfolobaceae; Sulfolobus
NC_013588	RefSeq	RVG	G713	17,602	39	Sulfolobus spindle-shaped virus 7	Viruses; dsDNA viruses, no RNA stage; Fuselloviridae; Alphafusellovirus.	693628	Archaea; Crenarchaeota; Thermoprotei; Sulfolobales; Sulfolobaceae; Sulfolobus
NC_005360	RefSeq	RVG	G713	16,473	37.5	Sulfolobus virus Ragged Hills	Viruses; dsDNA viruses, no RNA stage; Fuselloviridae; Alphafusellovirus; unclassified Fusellovirus.	256994	Archaea; Crenarchaeota; Thermoprotei; Sulfolobales; Sulfolobaceae; Sulfolobus
NC_005361	RefSeq	RVG	G713	17,385	38.7	Sulfolobus virus Kamchatka 1	Viruses; dsDNA viruses, no RNA stage; Fuselloviridae; Alphafusellovirus; unclassified Fusellovirus.	248496	Archaea; Crenarchaeota; Thermoprotei; Sulfolobales; Sulfolobaceae; Sulfolobus
NC_001338	RefSeq	RVG	G713	15,465	39.7	Sulfolobus spindle-shaped virus 1	Viruses; dsDNA viruses, no RNA stage; Fuselloviridae; Alphafusellovirus.	244589	Archaea; Crenarchaeota; Thermoprotei; Sulfolobales; Sulfolobaceae; Sulfolobus
NC_013587	RefSeq	RVG	G713	15,684	38.2	Sulfolobus spindle-shaped virus 6	Viruses; dsDNA viruses, no RNA stage; Fuselloviridae; Betafusellovirus.	693627	Archaea; Crenarchaeota; Thermoprotei; Sulfolobales; Sulfolobaceae; Sulfolobus
NC_013585	RefSeq	RVG	G713	24,186	37.7	Acidianus spindle-shaped virus 1	Viruses; dsDNA viruses, no RNA stage; Fuselloviridae; Betafusellovirus.	693629	Archaea; Crenarchaeota; Thermoprotei; Sulfolobales; Sulfolobaceae; Acidianus
NC_020882	RefSeq	RVG	G713	14,847	45.4	Sulfolobales Mexican fusellovirus 1	Viruses; dsDNA viruses, no RNA stage; Fuselloviridae; unclassified Fuselloviridae.	1298531	Archaea; Crenarchaeota; Thermoprotei
NC_028268	RefSeq	RVG	G714	38,049	56.5	Aeropyrum pernix spindle-shaped virus 1	Viruses; unclassified archaeal viruses.	1032473	-
NC_028256	RefSeq	RVG	G715	13,769	55.5	Aeropyrum pernix ovoid virus 1	Viruses; dsDNA viruses, no RNA stage; Guttaviridae; Betaguttavirus.	1032474	-
NC_009452	RefSeq	RVG	G716	23,814	34.6	Acidianus bottle-shaped virus	Viruses; dsDNA viruses, no RNA stage; Ampullaviridae; Ampullavirus.	437444	Archaea; Crenarchaeota; Thermoprotei; Sulfolobales; Sulfolobaceae; Acidianus
NC_028938	RefSeq	RVG	G716	22,613	33.3	Acidianus bottle-shaped virus 2	Viruses; dsDNA viruses, no RNA stage; Ampullaviridae; Ampullavirus; unclassified Ampullavirus.	1732173	-
NC_028787	RefSeq	RVG	G716	28,489	31.7	Acidianus bottle-shaped virus 3	Viruses; dsDNA viruses, no RNA stage; Ampullaviridae; Ampullavirus; unclassified Ampullavirus.	1732174	-
NC_016899	RefSeq	RVG	G717	21,592	49.5	Thermococcus prieurii virus 1	Viruses; unclassified archaeal viruses.	1115696	Archaea; Euryarchaeota; Thermococci; Thermococcales; Thermococcaceae; Thermococcus
NC_009597	RefSeq	RVG	G718	18,098	47.2	Pyrococcus abyssi virus 1	Viruses; dsDNA viruses, no RNA stage; unclassified dsDNA viruses; unclassified archaeal dsDNA viruses.	425386	Archaea; Euryarchaeota; Thermococci; Thermococcales; Thermococcaceae; Pyrococcus
NC_006556	RefSeq	RVG	G719	20,933	49.5	Thermoproteus tenax spherical virus 1	Viruses; dsDNA viruses, no RNA stage; Globuloviridae; Globulovirus.	292639	Archaea; Crenarchaeota; Thermoprotei; Thermoproteales; Thermoproteaceae; Thermoproteus
NC_005872	RefSeq	RVG	G720	28,337	48.4	Pyrobaculum spherical virus	Viruses; dsDNA viruses, no RNA stage; Globuloviridae; Globulovirus.	270161	Archaea; Crenarchaeota; Thermoprotei; Thermoproteales; Thermoproteaceae; Pyrobaculum
NC_014322	RefSeq	RVG	G721	22,743	46.2	Hyperthermophilic Archaeal Virus 1	Viruses; dsDNA viruses, no RNA stage; unclassified dsDNA viruses.	762905	Archaea
LDNO01000010	cryoconite	EVG	G722	10,313	48.6	-	-	-	-
NC_007918	RefSeq	RVG	G723	16,067	40.4	His2 virus	Viruses; dsDNA viruses, no RNA stage; Salterprovirus; unclassified Salterprovirus.	128710	Archaea; Euryarchaeota; Halobacteria; Halobacteriales; Halobacteriaceae; Haloarcula
NC_007914	RefSeq	RVG	G724	14,462	38.9	His 1 virus	Viruses; dsDNA viruses, no RNA stage; Salterprovirus.	128708	Archaea; Euryarchaeota; Halobacteria; Halobacteriales; Halobacteriaceae; Haloarcula
X14855	EBI	RVG	G725	13,669	37	Thermoproteus tenax virus 1	Viruses; dsDNA viruses, no RNA stage; Ligamenvirales; Lipothrixviridae; Alphalipothrixvirus.	10479	-
LDNN01000026	cryoconite	EVG	G726	17,032	33.2	-	-	-	-
TARA_ERS488340_N002912	TOV	EVG	G727	10,669	32.3	-	-	-	-
LDNP01000010	cryoconite	EVG	G728	23,445	37.2	-	-	-	-
TARA_ERS490204_N001564	TOV	EVG	G729	16,193	46.7	-	-	-	-
TARA_ERS490204_N001395	TOV	EVG	G730	17,361	44.9	-	-	-	-
TARA_ERS488448_N003518	TOV	EVG	G731	10,363	45	-	-	-	-
AY848688	EBI	RVG	G732	14,942	48.4	Enterobacteria phage PR772	Viruses; dsDNA viruses, no RNA stage; Tectiviridae; Tectivirus.	261665	-
AY441783	EBI	RVG	G732	14,946	48.3	Enterobacteria phage PR772	Viruses; dsDNA viruses, no RNA stage; Tectiviridae; Tectivirus.	261665	-
AY848687	EBI	RVG	G732	14,939	48.2	Enterobacteria phage PR5	Viruses; dsDNA viruses, no RNA stage; Tectiviridae; Tectivirus.	318596	-
NC_001421	RefSeq	RVG	G732	14,927	48.1	Enterobacteria phage PRD1	Viruses; dsDNA viruses, no RNA stage; Tectiviridae; Tectivirus.	10658	Bacteria; Proteobacteria; Gammaproteobacteria; Enterobacteriales; Enterobacteriaceae; Salmonella; Salmonella enterica
AY848685	EBI	RVG	G732	14,937	48.2	Enterobacteria phage PR3	Viruses; dsDNA viruses, no RNA stage; Tectiviridae; Tectivirus.	318594	-
AY848684	EBI	RVG	G732	14,935	48.3	Enterobacteria phage L17	Viruses; dsDNA viruses, no RNA stage; Tectiviridae; Tectivirus.	318593	-
AY848686	EBI	RVG	G732	14,954	48.3	Enterobacteria phage PR4	Viruses; dsDNA viruses, no RNA stage; Tectiviridae; Tectivirus.	318595	-
TARA_ERS492160_N000767	TOV	EVG	G733	24,300	30.7	-	-	-	-
TARA_ERS488448_N000874	TOV	EVG	G733	25,455	31.1	-	-	-	-
TARA_ERS490204_N000819	TOV	EVG	G733	24,055	26.7	-	-	-	-
TARA_ERS490610_N001030	TOV	EVG	G733	24,803	32.1	-	-	-	-
TARA_ERS490204_N000844	TOV	EVG	G734	23,566	32.3	-	-	-	-
TARA_ERS490204_N000754	TOV	EVG	G734	25,093	28.8	-	-	-	-
TARA_ERS492160_N000763	TOV	EVG	G735	24,356	26.5	-	-	-	-
TARA_ERS490204_N000836	TOV	EVG	G736	23,686	33.3	-	-	-	-
TARA_ERS490204_N000830	TOV	EVG	G736	23,800	34.7	-	-	-	-
TARA_ERS490204_N000847	TOV	EVG	G736	23,475	33.1	-	-	-	-
TARA_ERS488892_N000470	TOV	EVG	G736	25,249	30.9	-	-	-	-
TARA_ERS490452_N000853	TOV	EVG	G737	18,505	32.9	-	-	-	-
TARA_ERS488929_N000790	TOV	EVG	G737	18,698	33.2	-	-	-	-
TARA_ERS488558_N001700	TOV	EVG	G737	19,651	33.7	-	-	-	-
TARA_ERS492160_N001000	TOV	EVG	G738	20,051	31.8	-	-	-	-
TARA_ERS488499_N000768	TOV	EVG	G738	20,126	31.6	-	-	-	-
TARA_ERS490204_N000611	TOV	EVG	G739	28,031	38	-	-	-	-
TARA_ERS488813_N000615	TOV	EVG	G740	22,138	26.6	-	-	-	-
TARA_ERS488836_N000465	TOV	EVG	G740	22,168	26.6	-	-	-	-
TARA_ERS492160_N000856	TOV	EVG	G740	22,565	23.5	-	-	-	-

TARA_ERS490346_N000875	TOV	EVG	G741	23,819	26.5	-	-	-	-
TARA_ERS490346_N000805	TOV	EVG	G742	25,359	33.8	-	-	-	-
TARA_ERS488448_N001103	TOV	EVG	G743	21,679	29.6	-	-	-	-
TARA_ERS488499_N000654	TOV	EVG	G743	21,998	29.5	-	-	-	-
TARA_ERS490285_N001117	TOV	EVG	G744	21,360	29.8	-	-	-	-
TARA_ERS488448_N000946	TOV	EVG	G745	24,018	31.2	-	-	-	-
TARA_ERS488518_N000918	TOV	EVG	G746	25,110	33.4	-	-	-	-
TARA_ERS488518_N001424	TOV	EVG	G747	19,411	37.3	-	-	-	-
TARA_ERS488448_N001253	TOV	EVG	G747	20,057	51.7	-	-	-	-
TARA_ERS489148_N000155	TOV	EVG	G748	44,741	48.3	-	-	-	-
TARA_ERS488701_N000148	TOV	EVG	G749	45,601	51.1	-	-	-	-
NC_007807	RefSeq	RVG	G750	43,365	44.9	Pseudomonas phage 119X	Viruses; dsDNA viruses, no RNA stage; Caudovirales; Podoviridae.	347326	Bacteria; Proteobacteria; Gammaproteobacteria; Pseudomonadales; Pseudomonadaceae; Pseudomonas; Pseudomonas aeruginosa group
NC_005884	RefSeq	RVG	G750	43,783	45.4	Pseudomonas phage PaP2	Viruses; dsDNA viruses, no RNA stage; Caudovirales; Podoviridae.	270673	Bacteria; Proteobacteria; Gammaproteobacteria; Pseudomonadales; Pseudomonadaceae; Pseudomonas; Pseudomonas aeruginosa group
LDNO01000005	cryoconite	EVG	G751	44,342	46.4	-	-	-	-
LDNN01000027	cryoconite	EVG	G752	16,965	39.3	-	-	-	-
FR725450	EBI	RVG	G753	23,750	53	Nitrososphaera phage Pro-Nvie1	Viruses; unclassified phages.	927392	-
TARA_ERS489059_N001507	TOV	EVG	G754	10,502	33.4	-	-	-	-
TARA_ERS488836_N002052	TOV	EVG	G754	10,502	33.5	-	-	-	-
TARA_ERS489113_N002073	TOV	EVG	G754	10,502	33.4	-	-	-	-
TARA_ERS488589_N002486	TOV	EVG	G754	10,499	33.4	-	-	-	-
TARA_ERS488673_N002958	TOV	EVG	G754	10,502	33.5	-	-	-	-
TARA_ERS488892_N002299	TOV	EVG	G754	10,502	33.5	-	-	-	-
TARA_ERS488558_N003782	TOV	EVG	G754	10,502	33.5	-	-	-	-
TARA_ERS488813_N002422	TOV	EVG	G754	10,502	33.5	-	-	-	-
TARA_ERS488929_N002191	TOV	EVG	G754	10,502	33.4	-	-	-	-
TARA_ERS488448_N003460	TOV	EVG	G754	10,477	33.9	-	-	-	-
TARA_ERS488354_N001862	TOV	EVG	G754	10,513	33.9	-	-	-	-
TARA_ERS492198_N002526	TOV	EVG	G754	10,388	34.5	-	-	-	-
TARA_ERS492160_N002717	TOV	EVG	G754	10,424	34.4	-	-	-	-
TARA_ERS492198_N002567	TOV	EVG	G754	10,302	34.2	-	-	-	-
TARA_ERS490494_N002391	TOV	EVG	G754	10,258	34	-	-	-	-
TARA_ERS488673_N002923	TOV	EVG	G754	10,582	34.2	-	-	-	-
TARA_ERS489603_N001973	TOV	EVG	G754	10,258	34.1	-	-	-	-
TARA_ERS488354_N001930	TOV	EVG	G754	10,258	34.1	-	-	-	-
TARA_ERS490557_N003037	TOV	EVG	G754	10,258	34	-	-	-	-
TARA_ERS488589_N002589	TOV	EVG	G754	10,258	34.1	-	-	-	-
TARA_ERS488892_N002402	TOV	EVG	G754	10,258	34	-	-	-	-
TARA_ERS489148_N002866	TOV	EVG	G754	10,258	34	-	-	-	-
TARA_ERS488340_N003062	TOV	EVG	G754	10,258	34	-	-	-	-
TARA_ERS478007_N002613	TOV	EVG	G754	10,287	34	-	-	-	-
TARA_ERS488701_N002551	TOV	EVG	G754	10,258	34.1	-	-	-	-
TARA_ERS490120_N003125	TOV	EVG	G754	10,313	32	-	-	-	-
TARA_ERS488929_N000820	TOV	EVG	G754	18,296	34.6	-	-	-	-
TARA_ERS490320_N001787	TOV	EVG	G754	10,612	34.6	-	-	-	-
TARA_ERS490142_N002444	TOV	EVG	G754	10,613	34.6	-	-	-	-
TARA_ERS490180_N002326	TOV	EVG	G754	10,613	34.6	-	-	-	-
TARA_ERS490285_N003219	TOV	EVG	G754	10,613	34.6	-	-	-	-
TARA_ERS488701_N002242	TOV	EVG	G754	11,001	34.6	-	-	-	-
TARA_ERS490494_N002088	TOV	EVG	G754	11,051	34.6	-	-	-	-
TARA_ERS490557_N002931	TOV	EVG	G754	10,514	34.6	-	-	-	-
TARA_ERS489603_N001877	TOV	EVG	G754	10,514	34.6	-	-	-	-
TARA_ERS488589_N002480	TOV	EVG	G754	10,514	34.5	-	-	-	-
TARA_ERS489113_N002069	TOV	EVG	G754	10,514	34.6	-	-	-	-
TARA_ERS489943_N002703	TOV	EVG	G754	10,514	34.6	-	-	-	-
TARA_ERS489059_N001504	TOV	EVG	G754	10,514	34.6	-	-	-	-
TARA_ERS488558_N003774	TOV	EVG	G754	10,514	34.5	-	-	-	-
TARA_ERS490610_N003428	TOV	EVG	G754	10,514	34.6	-	-	-	-
TARA_ERS490346_N003029	TOV	EVG	G754	10,514	34.5	-	-	-	-
TARA_ERS490452_N002119	TOV	EVG	G754	10,514	34.5	-	-	-	-
TARA_ERS489148_N002763	TOV	EVG	G754	10,514	34.6	-	-	-	-
TARA_ERS489285_N002079	TOV	EVG	G754	10,514	34.6	-	-	-	-
TARA_ERS490053_N002767	TOV	EVG	G754	10,514	34.5	-	-	-	-
TARA_ERS488892_N002292	TOV	EVG	G754	10,514	34.5	-	-	-	-
TARA_ERS488836_N002046	TOV	EVG	G754	10,514	34.6	-	-	-	-
TARA_ERS488929_N002188	TOV	EVG	G754	10,514	34.6	-	-	-	-
TARA_ERS490026_N002075	TOV	EVG	G754	10,514	34.5	-	-	-	-
TARA_ERS488673_N002952	TOV	EVG	G754	10,514	34.5	-	-	-	-
TARA_ERS489084_N002946	TOV	EVG	G754	10,514	34.6	-	-	-	-
TARA_ERS490494_N002289	TOV	EVG	G754	10,514	34.5	-	-	-	-
TARA_ERS490120_N003040	TOV	EVG	G754	10,514	34.6	-	-	-	-
TARA_ERS488813_N002418	TOV	EVG	G754	10,514	34.6	-	-	-	-
TARA_ERS492198_N002523	TOV	EVG	G754	10,410	34.6	-	-	-	-
TARA_ERS490953_N001422	TOV	EVG	G755	10,501	33.5	-	-	-	-

TARA_ERS490180_N002453	TOV	EVG	G756	10,208	33.2	-	-	-	-
TARA_ERS490142_N002570	TOV	EVG	G756	10,232	33.1	-	-	-	-
TARA_ERS489084_N001165	TOV	EVG	G756	17,302	34	-	-	-	-
TARA_ERS490320_N001910	TOV	EVG	G757	10,259	33.8	-	-	-	-
TARA_ERS489113_N000868	TOV	EVG	G757	17,860	34	-	-	-	-
TARA_ERS490120_N001854	TOV	EVG	G758	14,961	35.7	-	-	-	-
TARA_ERS490452_N001909	TOV	EVG	G759	11,230	33.6	-	-	-	-
TARA_ERS492198_N001859	TOV	EVG	G760	12,374	34.1	-	-	-	-
TARA_ERS489943_N001939	TOV	EVG	G761	13,075	33.1	-	-	-	-
TARA_ERS490142_N001144	TOV	EVG	G762	18,091	34.5	-	-	-	-
TARA_ERS489603_N000792	TOV	EVG	G763	17,080	39.5	-	-	-	-
TARA_ERS490204_N000368	TOV	EVG	G764	35,326	41.6	-	-	-	-
TARA_ERS490204_N000310	TOV	EVG	G765	38,200	42.3	-	-	-	-
TARA_ERS478052_N001198	TOV	EVG	G766	16,400	34.8	-	-	-	-
NC_021322	RefSeq	RVG	G767	49,107	56.5	Halovirus HHTV-1	Viruses; dsDNA viruses, no RNA stage; unclassified dsDNA viruses; unclassified archaeal dsDNA viruses; Haloviruses.	1273750	Archaea; Euryarchaeota; Halobacteria; Halobacteriales; Halobacteriaceae; Haloarcula
TARA_ERS490494_N000127	TOV	EVG	G768	46,779	42.3	-	-	-	-
NC_009551	RefSeq	RVG	G769	43,249	46.5	Phormidium phage Pf-WMP3	Viruses; dsDNA viruses, no RNA stage; Caudovirales; Podoviridae.	440250	Bacteria; Cyanobacteria; Oscillatoriophycideae; Oscillatoriales; Phormidium
NC_022751	RefSeq	RVG	G769	42,480	46.4	Cyanophage PP	Viruses; dsDNA viruses, no RNA stage; Caudovirales; Podoviridae.	434346	Bacteria; Cyanobacteria; Oscillatoriophycideae; Oscillatoriales
NC_024358	RefSeq	RVG	G770	41,750	43.4	Anabaena phage A-4L	Viruses; dsDNA viruses, no RNA stage; Caudovirales; Podoviridae.	1357732	Bacteria; Cyanobacteria; Nostocales; Nostocaceae; Anabaena
NC_008367	RefSeq	RVG	G771	40,938	51.8	Phormidium phage Pf-WMP4	Viruses; dsDNA viruses, no RNA stage; Caudovirales; Podoviridae.	400567	Bacteria; Cyanobacteria; Oscillatoriophycideae; Oscillatoriales; Phormidium
TARA_ERS488757_N001223	TOV	EVG	G772	11,267	47.5	-	-	-	-
TARA_ERS488737_N001423	TOV	EVG	G772	11,267	47.6	-	-	-	-
TARA_ERS488813_N002148	TOV	EVG	G772	11,267	47.5	-	-	-	-
TARA_ERS488737_N001499	TOV	EVG	G772	10,963	47.2	-	-	-	-
TARA_ERS488757_N001286	TOV	EVG	G772	10,964	47.2	-	-	-	-
TARA_ERS488836_N001324	TOV	EVG	G772	13,213	45.5	-	-	-	-
TARA_ERS488813_N001103	TOV	EVG	G773	16,106	50.4	-	-	-	-
TARA_ERS488737_N000826	TOV	EVG	G773	16,135	48	-	-	-	-
TARA_ERS488737_N001598	TOV	EVG	G774	10,520	42.7	-	-	-	-
TARA_ERS489148_N002776	TOV	EVG	G775	10,480	40.7	-	-	-	-
TARA_ERS488701_N002442	TOV	EVG	G775	10,480	40.8	-	-	-	-
TARA_ERS488701_N001814	TOV	EVG	G775	12,287	40.3	-	-	-	-
TARA_ERS490494_N001951	TOV	EVG	G775	11,536	39.1	-	-	-	-
TARA_ERS490610_N002523	TOV	EVG	G776	12,866	29.8	-	-	-	-
TARA_ERS490452_N001492	TOV	EVG	G776	13,095	28	-	-	-	-
TARA_ERS490053_N000650	TOV	EVG	G777	25,870	40.6	-	-	-	-
NC_017983	RefSeq	RVG	G778	43,788	57.2	Salisaeta icosahedral phage 1	Viruses; unclassified phages.	1183239	Bacteria; Bacteroidetes/Chlorobi group; Bacteroidetes; Bacteroidetes Order II. Incertae sedis; Rhodothermaceae; Salisaeta
TARA_ERS490053_N003016	TOV	EVG	G779	10,027	34.6	-	-	-	-
LDNN01000030	cryoconite	EVG	G780	14,105	46.9	-	-	-	-
TARA_ERS488813_N000010	TOV	EVG	G781	183,387	54.4	-	-	-	-
AF503408	EBI	RVG	G782	101,660	47.4	Enterobacteria phage P7	Viruses; dsDNA viruses, no RNA stage; Caudovirales; Myoviridae; Punalikevirus.	10682	-
NC_005856	RefSeq	RVG	G782	94,800	47.3	Enterobacteria phage P1	Viruses; dsDNA viruses, no RNA stage; Caudovirales; Myoviridae; Punalikevirus.	10678	Bacteria; Proteobacteria; Gammaproteobacteria; Enterobacteriales; Enterobacteriaceae; Escherichia
TARA_ERS490388_N001342	TOV	EVG	G783	17,445	56.6	-	-	-	-
NC_019512	RefSeq	RVG	G784	26,836	35.5	Helicobacter phage 1961P	Viruses; dsDNA viruses, no RNA stage; Caudovirales; Podoviridae.	1154995	Bacteria; Proteobacteria; delta/epsilon subdivisions; Epsilonproteobacteria; Campylobacterales; Helicobacteraceae; Helicobacter
NC_019928	RefSeq	RVG	G784	26,215	35.8	Helicobacter phage KHP30	Viruses; dsDNA viruses, no RNA stage; Caudovirales; Podoviridae.	1208236	Bacteria; Proteobacteria; delta/epsilon subdivisions; Epsilonproteobacteria; Campylobacterales; Helicobacteraceae; Helicobacter
NC_019931	RefSeq	RVG	G784	26,449	35.9	Helicobacter phage KHP40	Viruses; dsDNA viruses, no RNA stage; Caudovirales; Podoviridae.	1204178	Bacteria; Proteobacteria; delta/epsilon subdivisions; Epsilonproteobacteria; Campylobacterales; Helicobacteraceae; Helicobacter
NC_016568	RefSeq	RVG	G784	24,645	37.3	Helicobacter phage phiHP33	Viruses; dsDNA viruses, no RNA stage; Caudovirales; Siphoviridae.	1069378	Bacteria; Proteobacteria; delta/epsilon subdivisions; Epsilonproteobacteria; Campylobacterales; Helicobacteraceae; Helicobacter
KF114877	EBI	RVG	G785	87,887	34	Leptospira phage vB_LnoZ_CZ214-LE1	Viruses; unclassified phages.	1334243	-
KF114876	EBI	RVG	G785	86,537	39.1	Leptospira phage vB_LalZ_80412-LE1	Viruses; unclassified phages.	1334242	-
KF114880	EBI	RVG	G785	89,607	39.4	Leptospira phage vB_LinZ_10-LE1	Viruses; unclassified phages.	1334246	-
KF114879	EBI	RVG	G785	87,308	39.4	Leptospira phage vB_LbrZ_5399-LE1	Viruses; unclassified phages.	1334245	-
KF114878	EBI	RVG	G786	47,417	34.5	Leptospira phage vb_LkmZ_Bejolso9-LE1	Viruses; unclassified phages.	1334244	-
NC_001447	RefSeq	RVG	G787	11,965	32	Acholeplasma phage L2	Viruses; dsDNA viruses, no RNA stage; Plasmaviridae; Plasmavirus.	46014	Bacteria; Tenericutes; Mollicutes; Acholeplasmatales; Acholeplasmataceae; Acholeplasma
LDNP01000011	cryoconite	EVG	G788	15,770	59.2	-	-	-	-
TARA_ERS490142_N001868	TOV	EVG	G789	12,655	56.5	-	-	-	-
TARA_ERS488737_N000113	TOV	EVG	G790	58,364	33.9	-	-	-	-
LDNN01000029	cryoconite	EVG	G791	15,192	49.1	-	-	-	-
TARA_ERS490388_N000464	TOV	EVG	G792	36,566	52.8	-	-	-	-
TARA_ERS492160_N000154	TOV	EVG	G793	66,851	35.5	-	-	-	-
TARA_ERS488757_N001037	TOV	EVG	G794	12,186	31.2	-	-	-	-
TARA_ERS488589_N001952	TOV	EVG	G794	12,186	31.2	-	-	-	-
TARA_ERS489148_N002107	TOV	EVG	G794	12,186	31.2	-	-	-	-
TARA_ERS488701_N001850	TOV	EVG	G794	12,186	31.2	-	-	-	-
TARA_ERS488836_N001537	TOV	EVG	G794	12,185	31.2	-	-	-	-
TARA_ERS489084_N002225	TOV	EVG	G794	12,186	31.2	-	-	-	-

TARA_ERS488673_N002338	TOV	EVG	G794	12,186	31.2	-	-	-	-
TARA_ERS488813_N001860	TOV	EVG	G794	12,186	31.2	-	-	-	-
TARA_ERS488701_N002405	TOV	EVG	G794	10,538	30.9	-	-	-	-
TARA_ERS478007_N002226	TOV	EVG	G795	11,171	30.7	-	-	-	-
TARA_ERS488499_N002106	TOV	EVG	G795	11,172	30.8	-	-	-	-
TARA_ERS490346_N002301	TOV	EVG	G795	12,517	29.5	-	-	-	-
TARA_ERS492160_N001891	TOV	EVG	G795	13,010	29.4	-	-	-	-
TARA_ERS488673_N003190	TOV	EVG	G796	10,084	34.2	-	-	-	-
TARA_ERS491107_N001068	TOV	EVG	G797	15,093	30	-	-	-	-
TARA_ERS488892_N000371	TOV	EVG	G798	28,826	33.9	-	-	-	-
TARA_ERS488929_N000369	TOV	EVG	G798	28,829	33.9	-	-	-	-
TARA_ERS492160_N000557	TOV	EVG	G798	29,829	33.1	-	-	-	-
LDNN01000011	cryoconite	EVG	G799	42,127	46.2	-	-	-	-
NC_002628	RefSeq	RVG	G800	31,007	45.7	Methanothermobacter phage psiM100	Viruses; dsDNA viruses, no RNA stage; Caudovirales; Siphoviridae.	173824	Archaea; Euryarchaeota; Methanobacteria; Methanobacteriales; Methanobacteriaceae; Methanothermobacter
NC_001902	RefSeq	RVG	G800	26,111	46.3	Methanobacterium phage psiM2	Viruses; dsDNA viruses, no RNA stage; Caudovirales; Siphoviridae.	77048	Archaea; Euryarchaeota; Methanobacteria; Methanobacteriales; Methanobacteriaceae; Methanothermobacter
TARA_ERS489084_N000096	TOV	EVG	G801	49,006	37.5	-	-	-	-
LDNN01000019	cryoconite	EVG	G802	37,016	35.8	-	-	-	-
LDNN01000012	cryoconite	EVG	G803	41,742	41.4	-	-	-	-
LDNP01000002	cryoconite	EVG	G804	42,514	40.2	-	-	-	-
LDNN01000008	cryoconite	EVG	G805	46,397	34.9	-	-	-	-
TARA_ERS488701_N000842	TOV	EVG	G806	18,821	45.7	-	-	-	-
TARA_ERS478052_N000402	TOV	EVG	G807	33,006	50.5	-	-	-	-
TARA_ERS490494_N000237	TOV	EVG	G807	37,682	53.9	-	-	-	-
TARA_ERS488589_N000241	TOV	EVG	G808	37,075	51.5	-	-	-	-
TARA_ERS490494_N000244	TOV	EVG	G808	37,498	48.1	-	-	-	-
TARA_ERS489084_N000141	TOV	EVG	G809	43,554	52.6	-	-	-	-
LDNN01000016	cryoconite	EVG	G810	37,683	42.3	-	-	-	-
LDNN01000014	cryoconite	EVG	G810	38,498	47.3	-	-	-	-
LDNN01000006	cryoconite	EVG	G810	54,228	50.7	-	-	-	-
AP013518	uvMED	EVG	G811	38,921	36.6	uvMED-CGR-U-MedDCM-OCT-S31-C15 (G21)	-	-	-
TARA_ERS488448_N000811	TOV	EVG	G812	26,683	36.6	-	-	-	-
TARA_ERS492198_N000463	TOV	EVG	G812	27,160	36.8	-	-	-	-
LDNN01000021	cryoconite	EVG	G813	35,400	33.7	-	-	-	-
TARA_ERS488518_N000466	TOV	EVG	G814	37,579	33.4	-	-	-	-
TARA_ERS490204_N000311	TOV	EVG	G814	38,191	35.5	-	-	-	-
TARA_ERS490053_N000422	TOV	EVG	G815	33,128	37.8	-	-	-	-
TARA_ERS490120_N000587	TOV	EVG	G815	33,128	37.8	-	-	-	-
TARA_ERS488892_N000284	TOV	EVG	G815	32,467	43.3	-	-	-	-
TARA_ERS490120_N000448	TOV	EVG	G816	39,141	32.6	-	-	-	-
TARA_ERS490026_N000240	TOV	EVG	G817	30,644	33.8	-	-	-	-
NC_029032	RefSeq	RVG	G818	45,532	40	Phormidium phage MIS-PhV1A	Viruses; unclassified phages.	1391455	-
NC_028998	RefSeq	RVG	G818	41,291	40.1	Phormidium phage MIS-PhV1B	Viruses; unclassified phages.	1391456	-
LDNN01000033	cryoconite	EVG	G819	12,701	50.2	-	-	-	-
NC_016166	RefSeq	RVG	G820	74,431	56.8	Gordonia phage GTE7	Viruses; dsDNA viruses, no RNA stage; Caudovirales; Siphoviridae.	1100814	Bacteria; Actinobacteria; Actinobacteria; Corynebacteriales; Gordoniaceae; Gordonia
NC_028673	RefSeq	RVG	G820	73,419	56.6	Gordonia phage GMA7	Viruses; dsDNA viruses, no RNA stage; Caudovirales; Podoviridae.	1647286	-
NC_028966	RefSeq	RVG	G820	76,269	59.3	Tsukamurella phage TIN3	Viruses; dsDNA viruses, no RNA stage; Caudovirales; Siphoviridae.	1636546	-
NC_028865	RefSeq	RVG	G820	76,964	58.9	Tsukamurella phage TIN2	Viruses; dsDNA viruses, no RNA stage; Caudovirales; Siphoviridae.	1636545	-
NC_023706	RefSeq	RVG	G820	75,772	56.7	Rhodococcus phage ReqiDocB7	Viruses; dsDNA viruses, no RNA stage; Caudovirales; Siphoviridae.	691966	Bacteria; Actinobacteria; Actinobacteria; Corynebacteriales; Nocardiaceae; Rhodococcus
NC_029074	RefSeq	RVG	G821	75,987	50.7	Gordonia phage GordTnk2	Viruses; dsDNA viruses, no RNA stage; Caudovirales; Siphoviridae.	1622192	-
NC_029060	RefSeq	RVG	G821	76,276	50.7	Gordonia phage GordDuk1	Viruses; dsDNA viruses, no RNA stage; Caudovirales; Siphoviridae.	1622191	-
NC_028972	RefSeq	RVG	G821	75,167	50.8	Gordonia phage Gmala1	Viruses; dsDNA viruses, no RNA stage; Caudovirales; Siphoviridae.	1622190	-
NC_028668	RefSeq	RVG	G821	77,779	51.3	Gordonia phage GMA3	Viruses; dsDNA viruses, no RNA stage; Caudovirales; Podoviridae.	1647284	-
TARA_ERS490494_N001676	TOV	EVG	G822	12,613	32.6	-	-	-	-
TARA_ERS489113_N001133	TOV	EVG	G823	15,093	28.6	-	-	-	-
TARA_ERS488892_N001208	TOV	EVG	G823	15,232	28.5	-	-	-	-
TARA_ERS489148_N001400	TOV	EVG	G823	15,093	28.5	-	-	-	-
TARA_ERS492160_N001395	TOV	EVG	G823	15,845	28.7	-	-	-	-
TARA_ERS490557_N001753	TOV	EVG	G823	14,697	28.1	-	-	-	-
TARA_ERS490610_N002404	TOV	EVG	G823	13,320	31.8	-	-	-	-
TARA_ERS490557_N001816	TOV	EVG	G823	14,350	28.1	-	-	-	-
TARA_ERS490610_N001975	TOV	EVG	G824	15,375	27.4	-	-	-	-
TARA_ERS490346_N000456	TOV	EVG	G825	35,517	29.9	-	-	-	-
TARA_ERS489084_N000261	TOV	EVG	G825	35,559	30.4	-	-	-	-
NC_019930	RefSeq	RVG	G826	49,219	57.7	Tetrasphaera phage TJE1	Viruses; dsDNA viruses, no RNA stage; Caudovirales.	981335	Bacteria; Actinobacteria; Actinobacteria; Micrococcales; Intrasporangiaceae; Tetrasphaera
TARA_ERS490557_N000827	TOV	EVG	G827	23,287	37.5	-	-	-	-
TARA_ERS490610_N001105	TOV	EVG	G827	23,287	37.5	-	-	-	-
JQ692107	EBI	RVG	G828	76,350	48.8	Vibrio phage SSP002	Viruses; dsDNA viruses, no RNA stage; Caudovirales; Siphoviridae.	1161928	-
NC_019713	RefSeq	RVG	G828	78,751	49.7	Vibrio phage vB_VpaS_MAR10	Viruses; dsDNA viruses, no RNA stage; Caudovirales; Siphoviridae.	1229755	Bacteria; Proteobacteria; Gammaproteobacteria; Vibrionales; Vibrionaceae; Vibrio; Vibrio harveyi group
TARA_ERS492198_N000058	TOV	EVG	G829	63,587	58.3	-	-	-	-

TARA_ERS492198_N000185	TOV	EVG	G830	40,500	58	-	-	-	-
TARA_ERS488558_N000626	TOV	EVG	G831	41,813	60.2	-	-	-	-
TARA_ERS492198_N000169	TOV	EVG	G831	42,137	59.6	-	-	-	-
JF713456	EBI	RVG	G832	81,509	46.9	Vibrio phage 1	Viruses; dsDNA viruses, no RNA stage; Caudovirales; Siphoviridae.	187944	-
NC_016567	RefSeq	RVG	G832	81,184	45	Vibrio phage SIO-2	Viruses; dsDNA viruses, no RNA stage; Caudovirales; Siphoviridae.	700512	Bacteria; Proteobacteria; Gammaproteobacteria; Vibrionales; Vibrionaceae; Vibrio
NC_023502	RefSeq	RVG	G833	109,558	59	Rhizobium phage vB_RleS_L338C	Viruses; dsDNA viruses, no RNA stage; Caudovirales; Siphoviridae.	1414737	Bacteria; Proteobacteria; Alphaproteobacteria; Rhizobiales; Rhizobiaceae; Rhizobium
TARA_ERS492198_N000021	TOV	EVG	G834	91,642	48.9	-	-	-	-
NC_019768	RefSeq	RVG	G835	41,468	49.3	Enterobacteria phage HK106	Viruses; dsDNA viruses, no RNA stage; Caudovirales; Siphoviridae.	432198	Bacteria; Proteobacteria; Gammaproteobacteria; Enterobacteriales; Enterobacteriaceae; Escherichia
NC_019715	RefSeq	RVG	G835	39,578	49.6	Enterobacterial phage mEp234	Viruses; dsDNA viruses, no RNA stage; Caudovirales; Siphoviridae; Lambdalikevirus; unclassified Lambda-like viruses.	1147157	Bacteria; Proteobacteria; Gammaproteobacteria; Enterobacteriales; Enterobacteriaceae; Escherichia
NC_019767	RefSeq	RVG	G835	40,155	49.8	Enterobacteria phage HK544	Viruses; dsDNA viruses, no RNA stage; Caudovirales; Siphoviridae.	432201	Bacteria; Proteobacteria; Gammaproteobacteria; Enterobacteriales; Enterobacteriaceae; Escherichia
NC_019714	RefSeq	RVG	G835	39,026	50.1	Enterobacteria phage HK446	Viruses; dsDNA viruses, no RNA stage; Caudovirales; Siphoviridae; Lambdalikevirus; unclassified Lambda-like viruses.	1147145	Bacteria; Proteobacteria; Gammaproteobacteria; Enterobacteriales; Enterobacteriaceae; Escherichia
NC_002167	RefSeq	RVG	G835	39,732	49.8	Enterobacteria phage HK97	Viruses; dsDNA viruses, no RNA stage; Caudovirales; Siphoviridae; Lambdalikevirus.	37554	Bacteria; Proteobacteria; Gammaproteobacteria; Enterobacteriales; Enterobacteriaceae; Escherichia
NC_019719	RefSeq	RVG	G835	41,528	49.7	Enterobacteria phage HK633	Viruses; dsDNA viruses, no RNA stage; Caudovirales; Siphoviridae; Lambdalikevirus; unclassified Lambda-like viruses.	1147147	Bacteria; Proteobacteria; Gammaproteobacteria; Enterobacteriales; Enterobacteriaceae; Escherichia
NC_016160	RefSeq	RVG	G835	36,661	50.2	Escherichia phage HK75	Viruses; dsDNA viruses, no RNA stage; Caudovirales; Siphoviridae; Lambdalikevirus; unclassified Lambda-like viruses.	906668	Bacteria; Proteobacteria; Gammaproteobacteria; Enterobacteriales; Enterobacteriaceae; Escherichia
NC_019769	RefSeq	RVG	G835	38,964	50.9	Enterobacteria phage HK542	Viruses; dsDNA viruses, no RNA stage; Caudovirales; Siphoviridae.	432200	Bacteria; Proteobacteria; Gammaproteobacteria; Enterobacteriales; Enterobacteriaceae; Escherichia
NC_019709	RefSeq	RVG	G835	41,567	49.3	Enterobacteria phage mEpX1	Viruses; dsDNA viruses, no RNA stage; Caudovirales; Siphoviridae; Lambdalikevirus; unclassified Lambda-like viruses.	1147153	Bacteria; Proteobacteria; Gammaproteobacteria; Enterobacteriales; Enterobacteriaceae; Escherichia
NC_019705	RefSeq	RVG	G835	38,759	50.1	Enterobacteria phage mEpX2	Viruses; dsDNA viruses, no RNA stage; Caudovirales; Siphoviridae; Lambdalikevirus; unclassified Lambda-like viruses.	1147154	Bacteria; Proteobacteria; Gammaproteobacteria; Enterobacteriales; Enterobacteriaceae; Escherichia
NC_002166	RefSeq	RVG	G835	40,751	49.5	Enterobacteria phage HK022	Viruses; dsDNA viruses, no RNA stage; Caudovirales; Siphoviridae; Lambdalikevirus.	10742	Bacteria; Proteobacteria; Gammaproteobacteria; Enterobacteriales; Enterobacteriaceae; Escherichia
NC_019708	RefSeq	RVG	G835	37,595	50	Enterobacteria phage mEp235	Viruses; dsDNA viruses, no RNA stage; Caudovirales; Siphoviridae; Lambdalikevirus; unclassified Lambda-like viruses.	1147150	Bacteria; Proteobacteria; Gammaproteobacteria; Enterobacteriales; Enterobacteriaceae; Escherichia
NC_019710	RefSeq	RVG	G835	40,710	49.9	Enterobacteria phage HK140	Viruses; dsDNA viruses, no RNA stage; Caudovirales; Siphoviridae; Lambdalikevirus; unclassified Lambda-like viruses.	1147143	Bacteria; Proteobacteria; Gammaproteobacteria; Enterobacteriales; Enterobacteriaceae; Escherichia
NC_019934	RefSeq	RVG	G835	39,012	53.1	Cronobacter phage ENT39118	Viruses; dsDNA viruses, no RNA stage; Caudovirales; Siphoviridae.	984175	Bacteria; Proteobacteria; Gammaproteobacteria; Enterobacteriales; Enterobacteriaceae; Cronobacter
NC_019721	RefSeq	RVG	G835	40,029	51.7	Enterobacterial phage mEp390	Viruses; dsDNA viruses, no RNA stage; Caudovirales; Siphoviridae; Lambdalikevirus; unclassified Lambda-like viruses.	1147158	Bacteria; Proteobacteria; Gammaproteobacteria; Enterobacteriales; Enterobacteriaceae; Escherichia
NC_019720	RefSeq	RVG	G835	44,120	51	Enterobacterial phage mEp213	Viruses; dsDNA viruses, no RNA stage; Caudovirales; Siphoviridae; Lambdalikevirus; unclassified Lambda-like viruses.	1147156	Bacteria; Proteobacteria; Gammaproteobacteria; Enterobacteriales; Enterobacteriaceae; Escherichia
NC_019706	RefSeq	RVG	G835	42,780	50.8	Enterobacteria phage mEp043 c-1	Viruses; dsDNA viruses, no RNA stage; Caudovirales; Siphoviridae; Lambdalikevirus; unclassified Lambda-like viruses.	1147149	Bacteria; Proteobacteria; Gammaproteobacteria; Enterobacteriales; Enterobacteriaceae; Escherichia
NC_019723	RefSeq	RVG	G835	47,090	50.1	Enterobacteria phage HK630	Viruses; dsDNA viruses, no RNA stage; Caudovirales; Siphoviridae; Lambdalikevirus; unclassified Lambda-like viruses.	1147146	Bacteria; Proteobacteria; Gammaproteobacteria; Enterobacteriales; Enterobacteriaceae; Escherichia
NC_001416	RefSeq	RVG	G835	48,502	49.9	Enterobacteria phage lambda	Viruses; dsDNA viruses, no RNA stage; Caudovirales; Siphoviridae; Lambdalikevirus.	10710	Bacteria; Proteobacteria; Gammaproteobacteria; Enterobacteriales; Enterobacteriaceae; Escherichia
EU078592	EBI	RVG	G835	42,925	51.9	Enterobacteria phage DE3	Viruses; dsDNA viruses, no RNA stage; Caudovirales; Siphoviridae; Lambdalikevirus.	482822	-
NC_019711	RefSeq	RVG	G835	47,288	49.6	Enterobacteria phage HK629	Viruses; dsDNA viruses, no RNA stage; Caudovirales; Siphoviridae; Lambdalikevirus; unclassified Lambda-like viruses.	1147148	Bacteria; Proteobacteria; Gammaproteobacteria; Enterobacteriales; Enterobacteriaceae; Escherichia
NC_019704	RefSeq	RVG	G835	44,375	51.4	Enterobacteria phage mEp237	Viruses; dsDNA viruses, no RNA stage; Caudovirales; Siphoviridae; Lambdalikevirus; unclassified Lambda-like viruses.	1147151	Bacteria; Proteobacteria; Gammaproteobacteria; Enterobacteriales; Enterobacteriaceae; Escherichia
NC_019717	RefSeq	RVG	G835	45,366	52	Enterobacteria phage HK225	Viruses; dsDNA viruses, no RNA stage; Caudovirales; Siphoviridae; Lambdalikevirus; unclassified Lambda-like viruses.	1147144	Bacteria; Proteobacteria; Gammaproteobacteria; Enterobacteriales; Enterobacteriaceae; Escherichia
NC_021190	RefSeq	RVG	G835	46,150	52.1	Enterobacteria phage phi80	Viruses; dsDNA viruses, no RNA stage; Caudovirales; Siphoviridae; Lambdalikevirus; unclassified Lambda-like viruses.	10713	Bacteria; Proteobacteria; Gammaproteobacteria; Enterobacteriales; Enterobacteriaceae; Escherichia
NC_019716	RefSeq	RVG	G836	44,510	50.9	Enterobacteria phage mEp460	Viruses; dsDNA viruses, no RNA stage; Caudovirales; Siphoviridae; Lambdalikevirus; unclassified Lambda-like viruses.	1147152	Bacteria; Proteobacteria; Gammaproteobacteria; Enterobacteriales; Enterobacteriaceae; Escherichia
NC_009514	RefSeq	RVG	G836	47,021	49.1	Enterobacteria phage cdtI	Viruses; dsDNA viruses, no RNA stage; Caudovirales; Siphoviridae; Lambdalikevirus; unclassified Lambda-like viruses.	414970	Bacteria; Proteobacteria; Gammaproteobacteria; Enterobacteriales; Enterobacteriaceae; Escherichia
KF030445	EBI	RVG	G836	47,343	52.3	Escherichia phage 1720a-02	Viruses; dsDNA viruses, no RNA stage; Caudovirales; Podoviridae.	1115653	-
NC_010393	RefSeq	RVG	G836	45,840	51.1	Phage Gifsy-2	Viruses; unclassified phages.	129862	Bacteria; Proteobacteria; Gammaproteobacteria; Enterobacteriales; Enterobacteriaceae; Salmonella; Salmonella enterica
NC_010392	RefSeq	RVG	G836	48,491	51.1	Phage Gifsy-1	Viruses; unclassified phages.	129861	Bacteria; Proteobacteria; Gammaproteobacteria; Enterobacteriales; Enterobacteriaceae; Salmonella; Salmonella enterica
NC_010391	RefSeq	RVG	G836	42,723	51.6	Salmonella phage Fels-1	Viruses; unclassified phages.	128975	Bacteria; Proteobacteria; Gammaproteobacteria; Enterobacteriales; Enterobacteriaceae; Salmonella; Salmonella enterica
KJ909655	EBI	RVG	G837	61,137	49.7	Escherichia Stx1-converting recombinant phage HUN/2013	Viruses; dsDNA viruses, no RNA stage; Caudovirales; Podoviridae.	1506453	-
NC_004913	RefSeq	RVG	G837	59,866	49.7	Escherichia Stx1 converting phage	Viruses; dsDNA viruses, no RNA stage; Caudovirales; Podoviridae.	194948	Bacteria; Proteobacteria; Gammaproteobacteria; Enterobacteriales; Enterobacteriaceae; Escherichia
NC_000902	RefSeq	RVG	G837	60,942	49.9	Enterobacteria phage VT2-Sakai	Viruses; dsDNA viruses, no RNA stage; Caudovirales; Podoviridae.	97081	Bacteria; Proteobacteria; Gammaproteobacteria; Enterobacteriales; Enterobacteriaceae; Escherichia
NC_004914	RefSeq	RVG	G837	62,706	49.9	Stx2 converting phage II	Viruses; dsDNA viruses, no RNA stage; Caudovirales; Podoviridae.	194949	Bacteria; Proteobacteria; Gammaproteobacteria; Enterobacteriales; Enterobacteriaceae; Escherichia
AP004402	EBI	RVG	G837	61,765	49.4	Stx2 converting phage I	Viruses; dsDNA viruses, no RNA stage; Caudovirales; Podoviridae.	180816	-

NC_000924	RefSeq	RVG	G837	61,670	49.4	Enterobacteria phage 933W	Viruses; dsDNA viruses, no RNA stage; Caudovirales; Podoviridae.	10730	Bacteria; Proteobacteria; Gammaproteobacteria; Enterobacteriales; Enterobacteriaceae; Escherichia
NC_010237	RefSeq	RVG	G837	63,395	49.5	Enterobacteria phage Min27	Viruses; dsDNA viruses, no RNA stage; Caudovirales; Podoviridae.	489779	Bacteria; Proteobacteria; Gammaproteobacteria; Enterobacteriales; Enterobacteriaceae; Escherichia
NC_027984	RefSeq	RVG	G837	57,677	49.7	Stx2 converting phage vB_EcoP_24B	Viruses; dsDNA viruses, no RNA stage; Caudovirales; Podoviridae.	866553	Bacteria; Proteobacteria; Gammaproteobacteria; Enterobacteriales; Enterobacteriaceae; Escherichia
NC_008464	RefSeq	RVG	G837	60,238	49.1	Stx2-converting phage 86	Viruses; dsDNA viruses, no RNA stage; Caudovirales; Podoviridae.	379329	Bacteria; Proteobacteria; Gammaproteobacteria; Enterobacteriales; Enterobacteriaceae; Escherichia
NC_018846	RefSeq	RVG	G837	60,894	50.2	Escherichia phage P13374	Viruses; dsDNA viruses, no RNA stage; Caudovirales; Podoviridae.	1150869	Bacteria; Proteobacteria; Gammaproteobacteria; Enterobacteriales; Enterobacteriaceae; Escherichia
NC_028660	RefSeq	RVG	G837	61,035	50.2	Escherichia phage phi191	Viruses; dsDNA viruses, no RNA stage; Caudovirales; Podoviridae.	1458706	-
NC_019442	RefSeq	RVG	G837	60,523	50.3	Escherichia phage TL-2011c	Viruses; dsDNA viruses, no RNA stage; Caudovirales; Podoviridae.	1124655	Bacteria; Proteobacteria; Gammaproteobacteria; Enterobacteriales; Enterobacteriaceae; Escherichia
NC_028685	RefSeq	RVG	G837	62,851	50.1	Shigella phage Ss-VASD	Viruses; dsDNA viruses, no RNA stage; Caudovirales; Podoviridae.	1675531	-
NC_028656	RefSeq	RVG	G837	65,955	50.1	Enterobacteria phage VT2phi_272	Viruses; dsDNA viruses, no RNA stage; Caudovirales; Podoviridae.	936054	-
NC_028449	RefSeq	RVG	G837	63,569	50.2	Escherichia phage PA2	Viruses; dsDNA viruses, no RNA stage; Caudovirales; Podoviridae.	1660365	-
NC_029120	RefSeq	RVG	G837	60,875	49.1	Shigella phage 75/02 Stx	Viruses; dsDNA viruses, no RNA stage; Caudovirales; Podoviridae.	1416032	-
NC_025434	RefSeq	RVG	G837	62,699	49.3	Shigella phage POCJ13	Viruses; dsDNA viruses, no RNA stage; Caudovirales; Podoviridae.	1498227	Bacteria; Proteobacteria; Gammaproteobacteria; Enterobacteriales; Enterobacteriaceae; Shigella
FM180578	EBI	RVG	G837	57,248	51.1	Enterobacteria phage 2851	Viruses; dsDNA viruses, no RNA stage; Caudovirales; Podoviridae.	254397	-
NC_011357	RefSeq	RVG	G837	62,147	50.9	Stx2-converting phage 1717	Viruses; dsDNA viruses, no RNA stage; Caudovirales; Siphoviridae; Lambdalikevirus; unclassified Lambda-like viruses.	563769	Bacteria; Proteobacteria; Gammaproteobacteria; Enterobacteriales; Enterobacteriaceae; Escherichia
NC_011356	RefSeq	RVG	G837	54,896	51.1	Enterobacteria phage YYZ-2008	Viruses; unclassified phages.	564886	Bacteria; Proteobacteria; Gammaproteobacteria; Enterobacteriales; Enterobacteriaceae; Escherichia
NC_004813	RefSeq	RVG	G837	57,930	50.6	Enterobacteria phage BP-4795	Viruses; dsDNA viruses, no RNA stage; Caudovirales; Siphoviridae; Lambdalikevirus; unclassified Lambda-like viruses.	196242	Bacteria; Proteobacteria; Gammaproteobacteria; Enterobacteriales; Enterobacteriaceae; Escherichia
NC_005857	RefSeq	RVG	G838	51,601	51.5	Klebsiella phage phiKO2	Viruses; dsDNA viruses, no RNA stage; Caudovirales; Siphoviridae.	255431	Bacteria; Proteobacteria; Gammaproteobacteria; Enterobacteriales; Enterobacteriaceae; Klebsiella
NC_001901	RefSeq	RVG	G838	46,375	51.2	Enterobacteria phage N15	Viruses; dsDNA viruses, no RNA stage; Caudovirales; Siphoviridae; N15likevirus.	40631	Bacteria; Proteobacteria; Gammaproteobacteria; Enterobacteriales; Enterobacteriaceae; Escherichia
NC_005069	RefSeq	RVG	G838	46,339	44.6	Yersinia phage PY54	Viruses; dsDNA viruses, no RNA stage; Caudovirales; Siphoviridae.	172667	Bacteria; Proteobacteria; Gammaproteobacteria; Enterobacteriales; Enterobacteriaceae; Yersinia
NC_005284	RefSeq	RVG	G839	54,865	60.7	Burkholderia phage phi1026b	Viruses; dsDNA viruses, no RNA stage; Caudovirales; Siphoviridae; Phie125likevirus.	255131	Bacteria; Proteobacteria; Betaproteobacteria; Burkholderiales; Burkholderiaceae; Burkholderia; pseudomallei group
NC_003309	RefSeq	RVG	G839	53,373	61.2	Burkholderia phage phiE125	Viruses; dsDNA viruses, no RNA stage; Caudovirales; Siphoviridae; Phie125likevirus.	180504	Bacteria; Proteobacteria; Betaproteobacteria; Burkholderiales; Burkholderiaceae; Burkholderia; pseudomallei group
NC_009235	RefSeq	RVG	G839	48,674	60.4	Burkholderia phage phi6442	Viruses; dsDNA viruses, no RNA stage; Caudovirales; Siphoviridae; Phie125likevirus.	431891	Bacteria; Proteobacteria; Betaproteobacteria; Burkholderiales; Burkholderiaceae; Burkholderia; pseudomallei group
NC_013055	RefSeq	RVG	G839	39,896	60.7	Burkholderia phage KS9	Viruses; dsDNA viruses, no RNA stage; Caudovirales; Siphoviridae; Lambdalikevirus; unclassified Lambda-like viruses.	335797	Bacteria; Proteobacteria; Betaproteobacteria; Burkholderiales; Burkholderiaceae; Burkholderia; Burkholderia cepacia complex
NC_007497	RefSeq	RVG	G839	44,856	61.5	Burkholderia phage Bcep176	Viruses; dsDNA viruses, no RNA stage; Caudovirales; Siphoviridae.	348137	Bacteria; Proteobacteria; Betaproteobacteria; Burkholderiales; Burkholderiaceae; Burkholderia; Burkholderia cepacia complex
NC_023734	RefSeq	RVG	G840	35,725	44.5	Psychrobacter phage Psymv2	Viruses; dsDNA viruses, no RNA stage; Caudovirales; Siphoviridae.	1071177	Bacteria; Proteobacteria; Gammaproteobacteria; Pseudomonadales; Moraxellaceae; Psychrobacter
NC_022749	RefSeq	RVG	G841	39,758	50.3	Shigella phage SfIV	Viruses; dsDNA viruses, no RNA stage; Caudovirales; Myoviridae.	1407493	Bacteria; Proteobacteria; Gammaproteobacteria; Enterobacteriales; Enterobacteriaceae; Shigella
NC_021857	RefSeq	RVG	G841	41,475	49.2	Shigella phage SfII	Viruses; dsDNA viruses, no RNA stage; Caudovirales; Myoviridae.	66284	Bacteria; Proteobacteria; Gammaproteobacteria; Enterobacteriales; Enterobacteriaceae; Shigella
NC_003444	RefSeq	RVG	G841	37,074	50.8	Enterobacteria phage SfV	Viruses; dsDNA viruses, no RNA stage; Caudovirales; Myoviridae.	55884	Bacteria; Proteobacteria; Gammaproteobacteria; Enterobacteriales; Enterobacteriaceae; Shigella
NC_027339	RefSeq	RVG	G841	38,389	50.1	Enterobacteria phage SfI	Viruses; dsDNA viruses, no RNA stage; Caudovirales; Myoviridae.	1225789	Bacteria; Proteobacteria; Gammaproteobacteria; Enterobacteriales; Enterobacteriaceae; Shigella
NC_004313	RefSeq	RVG	G841	40,149	51	Salmonella phage ST64B	Viruses; dsDNA viruses, no RNA stage; Caudovirales.	175950	Bacteria; Proteobacteria; Gammaproteobacteria; Enterobacteriales; Enterobacteriaceae; Salmonella; Salmonella enterica
NC_003356	RefSeq	RVG	G841	42,575	49.4	Enterobacteria phage phiP27	Viruses; dsDNA viruses, no RNA stage; Caudovirales; Myoviridae.	103807	Bacteria; Proteobacteria; Gammaproteobacteria; Enterobacteriales; Enterobacteriaceae; Escherichia
NC_019725	RefSeq	RVG	G842	50,552	45.6	Escherichia phage ADB-2	Viruses; dsDNA viruses, no RNA stage; Caudovirales; Siphoviridae; Tunalikevirus; unclassified Tunalikevirus.	1216926	Bacteria; Proteobacteria; Gammaproteobacteria; Enterobacteriales; Enterobacteriaceae; Escherichia
NC_005833	RefSeq	RVG	G842	48,836	45.6	Enterobacteria phage T1	Viruses; dsDNA viruses, no RNA stage; Caudovirales; Siphoviridae; Tunalikevirus.	12355	Bacteria; Proteobacteria; Gammaproteobacteria; Enterobacteriales; Enterobacteriaceae; Escherichia
NC_015456	RefSeq	RVG	G842	50,661	45.4	Shigella phage ShfI1	Viruses; dsDNA viruses, no RNA stage; Caudovirales; Siphoviridae; Tunalikevirus.	1002724	Bacteria; Proteobacteria; Gammaproteobacteria; Enterobacteriales; Enterobacteriaceae; Shigella
NC_026010	RefSeq	RVG	G842	50,109	45.4	Shigella phage pSf-2	Viruses; dsDNA viruses, no RNA stage; Caudovirales; Siphoviridae.	1572702	Bacteria; Proteobacteria; Gammaproteobacteria; Enterobacteriales; Enterobacteriaceae; Shigella
NC_009540	RefSeq	RVG	G842	49,902	42.7	Escherichia phage Tls	Viruses; dsDNA viruses, no RNA stage; Caudovirales; Siphoviridae; Tunalikevirus; unclassified Tunalikevirus.	245685	Bacteria; Proteobacteria; Gammaproteobacteria; Enterobacteriales; Enterobacteriaceae; Escherichia
NC_027350	RefSeq	RVG	G842	49,816	42.8	Citrobacter phage Stevie	Viruses; dsDNA viruses, no RNA stage; Caudovirales; Siphoviridae.	1540096	Bacteria; Proteobacteria; Gammaproteobacteria; Enterobacteriales; Enterobacteriaceae; Citrobacter
NC_029071	RefSeq	RVG	G842	41,085	43.5	Salmonella phage 36	Viruses; dsDNA viruses, no RNA stage; Caudovirales; Siphoviridae.	1654889	-
NC_021331	RefSeq	RVG	G842	51,821	44	Shigella phage pSf-1	Viruses; dsDNA viruses, no RNA stage; Caudovirales; Siphoviridae.	1315956	Bacteria; Proteobacteria; Gammaproteobacteria; Enterobacteriales; Enterobacteriaceae; Shigella
NC_019509	RefSeq	RVG	G842	49,116	50.1	Cronobacter phage ESP2949-1	Viruses; dsDNA viruses, no RNA stage; Caudovirales; Siphoviridae; Tunalikevirus.	1073767	Bacteria; Proteobacteria; Gammaproteobacteria; Enterobacteriales; Enterobacteriaceae; Cronobacter
NC_028786	RefSeq	RVG	G842	49,462	50.6	Klebsiella phage 1513	Viruses; dsDNA viruses, no RNA stage; Caudovirales; Siphoviridae.	1610829	-

NC_028774	RefSeq	RVG	G842	48,754	50.8	Klebsiella phage Sushi	Viruses; dsDNA viruses, no RNA stage; Caudovirales; Siphoviridae.	1675609	-
NC_029099	RefSeq	RVG	G842	49,797	50.7	Klebsiella phage KP36	Viruses; dsDNA viruses, no RNA stage; Caudovirales; Siphoviridae; Tunalikevirus; unclassified Tunalikevirus.	1129191	-
NC_028760	RefSeq	RVG	G842	49,037	50.5	Klebsiella phage KLPN1	Viruses; dsDNA viruses, no RNA stage; Caudovirales; Siphoviridae.	1647408	-
KF771236	EBI	RVG	G842	46,719	43.8	Escherichia phage bV_EcoS_AHP24	Viruses; dsDNA viruses, no RNA stage; Caudovirales; Siphoviridae.	1416027	-
NC_024784	RefSeq	RVG	G842	46,440	43.8	Escherichia phage bV_EcoS_AHS24	Viruses; dsDNA viruses, no RNA stage; Caudovirales; Siphoviridae.	1416030	Bacteria; Proteobacteria; Gammaproteobacteria; Enterobacteriales; Enterobacteriaceae; Escherichia
NC_024793	RefSeq	RVG	G842	46,847	44	Escherichia phage bV_EcoS_AHP42	Viruses; dsDNA viruses, no RNA stage; Caudovirales; Siphoviridae.	1416028	Bacteria; Proteobacteria; Gammaproteobacteria; Enterobacteriales; Enterobacteriaceae; Escherichia
NC_024789	RefSeq	RVG	G842	45,746	43.9	Escherichia phage bV_EcoS_AKS96	Viruses; dsDNA viruses, no RNA stage; Caudovirales; Siphoviridae.	1416031	Bacteria; Proteobacteria; Gammaproteobacteria; Enterobacteriales; Enterobacteriaceae; Escherichia
KC579452	EBI	RVG	G842	47,285	44.3	Enterobacterio phage phiKP26	Viruses; dsDNA viruses, no RNA stage; Caudovirales; Siphoviridae; Tunalikevirus; unclassified Tunalikevirus.	1296653	-
KC333879	EBI	RVG	G842	43,017	44.2	Enterobacteria phage phiJLA23	Viruses; dsDNA viruses, no RNA stage; Caudovirales; Siphoviridae; Tunalikevirus; unclassified Tunalikevirus.	1273706	-
NC_019718	RefSeq	RVG	G842	45,805	44.2	Enterobacteria phage vB_EcoS_Rogue1	Viruses; dsDNA viruses, no RNA stage; Caudovirales; Siphoviridae; Tunalikevirus.	1147155	Bacteria; Proteobacteria; Gammaproteobacteria; Enterobacteriales; Enterobacteriaceae; Escherichia
NC_007291	RefSeq	RVG	G842	46,072	44	Escherichia phage Jk06	Viruses; dsDNA viruses, no RNA stage; Caudovirales; Siphoviridae; Tunalikevirus.	337828	Bacteria; Proteobacteria; Gammaproteobacteria; Enterobacteriales; Enterobacteriaceae; Escherichia
NC_024210	RefSeq	RVG	G842	47,112	44.1	Escherichia phage e4/1c	Viruses; dsDNA viruses, no RNA stage; Caudovirales; Siphoviridae.	1495286	Bacteria; Proteobacteria; Gammaproteobacteria; Enterobacteriales; Enterobacteriaceae; Escherichia
NC_023743	RefSeq	RVG	G842	47,180	44	Escherichia phage EB49	Viruses; dsDNA viruses, no RNA stage; Caudovirales; Siphoviridae; Tunalikevirus.	1048207	Bacteria; Proteobacteria; Gammaproteobacteria; Enterobacteriales; Enterobacteriaceae; Escherichia
NC_019404	RefSeq	RVG	G842	46,054	43.5	Enterobacteria phage vB_EcoS_ACG-M12	Viruses; dsDNA viruses, no RNA stage; Caudovirales; Siphoviridae; Tunalikevirus; unclassified Tunalikevirus.	1141140	Bacteria; Proteobacteria; Gammaproteobacteria; Enterobacteriales; Enterobacteriaceae; Escherichia
NC_007603	RefSeq	RVG	G842	46,219	44.3	Escherichia phage Rtp	Viruses; dsDNA viruses, no RNA stage; Caudovirales; Siphoviridae; Tunalikevirus.	355246	Bacteria; Proteobacteria; Gammaproteobacteria; Enterobacteriales; Enterobacteriaceae; Escherichia
NC_012223	RefSeq	RVG	G843	44,899	54.7	Enterobacteria phage SSL2009a	Viruses; dsDNA viruses, no RNA stage; Caudovirales; Siphoviridae; Hk578likevirus.	624134	Bacteria; Proteobacteria; Gammaproteobacteria; Enterobacteriales; Enterobacteriaceae; Escherichia
NC_019419	RefSeq	RVG	G843	43,457	54.8	Enterobacteria phage JL1	Viruses; dsDNA viruses, no RNA stage; Caudovirales; Siphoviridae; Hk578likevirus.	1245890	Bacteria; Proteobacteria; Gammaproteobacteria; Enterobacteriales; Enterobacteriaceae; Escherichia
NC_027383	RefSeq	RVG	G843	44,613	54.6	Escherichia phage YD-2008.s	Viruses; dsDNA viruses, no RNA stage; Caudovirales; Siphoviridae.	1567004	Bacteria; Proteobacteria; Gammaproteobacteria; Enterobacteriales; Enterobacteriaceae; Escherichia
NC_024783	RefSeq	RVG	G843	44,332	54.5	Enterobacteria phage EK99P-1	Viruses; dsDNA viruses, no RNA stage; Caudovirales; Siphoviridae.	1527514	Bacteria; Proteobacteria; Gammaproteobacteria; Enterobacteriales; Enterobacteriaceae; Escherichia
NC_016566	RefSeq	RVG	G843	44,077	54.4	Shigella phage EP23	Viruses; dsDNA viruses, no RNA stage; Caudovirales; Siphoviridae; Hk578likevirus.	1109721	Bacteria; Proteobacteria; Gammaproteobacteria; Enterobacteriales; Enterobacteriaceae; Shigella
NC_013600	RefSeq	RVG	G843	45,169	54.6	Sodalis phage SO1	Viruses; dsDNA viruses, no RNA stage; Caudovirales; Siphoviridae; Hk578likevirus.	673375	Bacteria; Proteobacteria; Gammaproteobacteria; Enterobacteriales; Enterobacteriaceae; Sodalis
NC_028901	RefSeq	RVG	G843	43,900	54.5	Escherichia phage slur05	Viruses; dsDNA viruses, no RNA stage; Caudovirales; Siphoviridae.	1720498	-
NC_019724	RefSeq	RVG	G843	43,741	54.5	Escherichia phage HK578	Viruses; dsDNA viruses, no RNA stage; Caudovirales; Siphoviridae; Hk578likevirus.	1147142	Bacteria; Proteobacteria; Gammaproteobacteria; Enterobacteriales; Enterobacteriaceae; Escherichia
KF772233	EBI	RVG	G843	43,015	55.4	Edwardsiella phage eiAU	Viruses; dsDNA viruses, no RNA stage; Caudovirales; Myoviridae.	945083	-
NC_023555	RefSeq	RVG	G843	43,017	55.4	Edwardsiella phage eiAU-183	Viruses; dsDNA viruses, no RNA stage; Caudovirales; Myoviridae.	1391659	Bacteria; Proteobacteria; Gammaproteobacteria; Enterobacteriales; Enterobacteriaceae; Edwardsiella
NC_016158	RefSeq	RVG	G844	49,576	52.5	Escherichia phage HK639	Viruses; dsDNA viruses, no RNA stage; Caudovirales; Siphoviridae; Lambdalikevirus; unclassified Lambda-like viruses.	906669	Bacteria; Proteobacteria; Gammaproteobacteria; Enterobacteriales; Enterobacteriaceae; Escherichia
NC_018454	RefSeq	RVG	G845	39,974	53.5	Cronobacter phage phiES15	Viruses; dsDNA viruses, no RNA stage; Caudovirales; Siphoviridae.	1168280	Bacteria; Proteobacteria; Gammaproteobacteria; Enterobacteriales; Enterobacteriaceae; Cronobacter
JX483881	EBI	RVG	G846	115,042	60.3	Rhizobium phage RHEph10	Viruses; unclassified phages.	1220717	-
NC_018843	RefSeq	RVG	G847	103,299	51.1	Salmonella phage SSU5	Viruses; dsDNA viruses, no RNA stage; Caudovirales; Siphoviridae.	1177632	Bacteria; Proteobacteria; Gammaproteobacteria; Enterobacteriales; Enterobacteriaceae; Salmonella; Salmonella enterica
NC_006949	RefSeq	RVG	G848	46,900	48.6	Enterobacteria phage ES18	Viruses; dsDNA viruses, no RNA stage; Caudovirales; Siphoviridae.	101570	Bacteria; Proteobacteria; Gammaproteobacteria; Enterobacteriales; Enterobacteriaceae; Salmonella; Salmonella enterica
NC_018279	RefSeq	RVG	G848	49,116	48.7	Salmonella phage vB_SosS_Oslo	Viruses; dsDNA viruses, no RNA stage; Caudovirales; Siphoviridae.	1168549	Bacteria; Proteobacteria; Gammaproteobacteria; Enterobacteriales; Enterobacteriaceae
NC_019545	RefSeq	RVG	G848	47,355	49.6	Salmonella phage SPN3UB	Viruses; dsDNA viruses, no RNA stage; Caudovirales; Siphoviridae.	1147140	Bacteria; Proteobacteria; Gammaproteobacteria; Enterobacteriales; Enterobacteriaceae; Salmonella; Salmonella enterica
JF314845	EBI	RVG	G848	22,162	50.8	Cronobacter phage ES2	Viruses; unclassified phages.	1073754	-
NC_019927	RefSeq	RVG	G848	47,611	51.6	Cronobacter phage ENT47670	Viruses; dsDNA viruses, no RNA stage; Caudovirales; Myoviridae.	984186	Bacteria; Proteobacteria; Gammaproteobacteria; Enterobacteriales; Enterobacteriaceae; Cronobacter
JX403939	EBI	RVG	G849	49,381	62.2	Pseudomonas phage YMC/01/01/P52_PAE_BP	Viruses; dsDNA viruses, no RNA stage; Caudovirales; Podoviridae.	1191380	-
NC_016762	RefSeq	RVG	G849	49,135	62.1	Pseudomonas phage phi297	Viruses; dsDNA viruses, no RNA stage; Caudovirales; Siphoviridae.	1129145	Bacteria; Proteobacteria; Gammaproteobacteria; Pseudomonadales; Pseudomonadaceae; Pseudomonas; Pseudomonas aeruginosa group
NC_021534	RefSeq	RVG	G850	47,552	47.3	Vibrio phage pYD38-A	Viruses; dsDNA viruses, no RNA stage; unclassified dsDNA phages.	754051	Bacteria; Proteobacteria; Gammaproteobacteria; Vibrionales; Vibrionaceae; Vibrio
NC_010495	RefSeq	RVG	G850	45,051	46.1	Salmonella phage Vi II-E1	Viruses; dsDNA viruses, no RNA stage; Caudovirales; Siphoviridae.	424716	Bacteria; Proteobacteria; Gammaproteobacteria; Enterobacteriales; Enterobacteriaceae; Salmonella
NC_028956	RefSeq	RVG	G851	52,139	41.1	Mannheimia phage vB_MhS_1152AP2	Viruses; dsDNA viruses, no RNA stage; Caudovirales; Siphoviridae; Lambdalikevirus; unclassified Lambda-like viruses.	1572747	-
NC_028853	RefSeq	RVG	G851	50,078	41.3	Mannheimia phage vB_MhS_535AP2	Viruses; dsDNA viruses, no RNA stage; Caudovirales; Siphoviridae; Lambdalikevirus; unclassified Lambda-like viruses.	1572743	-
NC_028743	RefSeq	RVG	G851	48,594	41.3	Mannheimia phage vB_MhS_587AP2	Viruses; dsDNA viruses, no RNA stage; Caudovirales; Siphoviridae; Lambdalikevirus; unclassified Lambda-like viruses.	1572745	-
TARA_ERS490142_N000303	TOV	EVG	G852	46,143	53.4	-	-	-	-
TARA_ERS490026_N000065	TOV	EVG	G852	46,143	53.4	-	-	-	-

TARA_ERS488836_N000087	TOV	EVG	G852	47,407	53	-	-	-	-
TARA_ERS488892_N000124	TOV	EVG	G853	44,810	50.8	-	-	-	-
NC_029046	RefSeq	RVG	G854	50,827	60.7	Sinorhizobium phage phiLM21	Viruses; dsDNA viruses, no RNA stage; Caudovirales; Siphoviridae.	1524882	-
NC_016765	RefSeq	RVG	G855	54,024	57.5	Pseudomonas phage PMG1	Viruses; dsDNA viruses, no RNA stage; Caudovirales; Siphoviridae; D3likevirus.	1129146	Bacteria; Proteobacteria; Gammaproteobacteria; Pseudomonadales; Pseudomonadaceae; Pseudomonas; Pseudomonas aeruginosa group
NC_002484	RefSeq	RVG	G855	56,426	57.8	Pseudomonas phage D3	Viruses; dsDNA viruses, no RNA stage; Caudovirales; Siphoviridae; D3likevirus.	31535	Bacteria; Proteobacteria; Gammaproteobacteria; Pseudomonadales; Pseudomonadaceae; Pseudomonas; Pseudomonas aeruginosa group
NC_011373	RefSeq	RVG	G855	46,872	56.3	Pseudomonas phage PAJU2	Viruses; dsDNA viruses, no RNA stage; Caudovirales; Siphoviridae.	504346	Bacteria; Proteobacteria; Gammaproteobacteria; Pseudomonadales; Pseudomonadaceae; Pseudomonas; Pseudomonas aeruginosa group
NC_028657	RefSeq	RVG	G855	60,919	58.7	Pseudomonas phage YMC11/02/R656	Viruses; dsDNA viruses, no RNA stage; Caudovirales; Siphoviridae.	1755689	-
NC_023575	RefSeq	RVG	G856	45,550	57.1	Pseudomonas phage vB_PaeP_Tr60_Ab31	Viruses; dsDNA viruses, no RNA stage; unclassified dsDNA phages.	1449437	Bacteria; Proteobacteria; Gammaproteobacteria; Pseudomonadales; Pseudomonadaceae; Pseudomonas; Pseudomonas aeruginosa group
NC_029066	RefSeq	RVG	G857	48,666	59.8	Pseudomonas phage PS-1	Viruses; dsDNA viruses, no RNA stage; Caudovirales; Siphoviridae.	1573458	-
NC_019541	RefSeq	RVG	G858	45,364	39.1	Acinetobacter phage Bphi-B1251	Viruses; dsDNA viruses, no RNA stage; Caudovirales; Podoviridae.	1221835	Bacteria; Proteobacteria; Gammaproteobacteria; Pseudomonadales; Moraxellaceae; Acinetobacter; Acinetobacter calcoaceticus/baumannii complex
NC_024365	RefSeq	RVG	G859	51,090	58.6	Pseudomonas phage phiPSA1	Viruses; dsDNA viruses, no RNA stage; Caudovirales; Siphoviridae.	1500757	Bacteria; Proteobacteria; Gammaproteobacteria; Pseudomonadales; Pseudomonadaceae; Pseudomonas; Pseudomonas syringae group; Pseudomonas syringae group genomosp. 1
NC_014900	RefSeq	RVG	G860	40,986	47.1	Salmonella phage ST160	Viruses; dsDNA viruses, no RNA stage; Caudovirales; Podoviridae; P22likevirus.	714583	Bacteria; Proteobacteria; Gammaproteobacteria; Enterobacteriales; Enterobacteriaceae; Salmonella; Salmonella enterica
NC_011802	RefSeq	RVG	G860	41,941	47	Salmonella phage SE1	Viruses; dsDNA viruses, no RNA stage; Caudovirales; Podoviridae; P22likevirus.	329864	Bacteria; Proteobacteria; Gammaproteobacteria; Enterobacteriales; Enterobacteriaceae; Salmonella
NC_005841	RefSeq	RVG	G860	41,391	47.4	Enterobacteria phage ST104	Viruses; dsDNA viruses, no RNA stage; Caudovirales; Podoviridae; P22likevirus.	221029	Bacteria; Proteobacteria; Gammaproteobacteria; Enterobacteriales; Enterobacteriaceae; Salmonella; Salmonella enterica
NC_004348	RefSeq	RVG	G860	40,679	47.5	Salmonella phage ST64T	Viruses; dsDNA viruses, no RNA stage; Caudovirales; Podoviridae; P22likevirus.	173443	Bacteria; Proteobacteria; Gammaproteobacteria; Enterobacteriales; Enterobacteriaceae; Salmonella; Salmonella enterica
NC_017985	RefSeq	RVG	G860	40,128	47.3	Salmonella phage SPN9CC	Viruses; dsDNA viruses, no RNA stage; Caudovirales; Podoviridae.	1127357	Bacteria; Proteobacteria; Gammaproteobacteria; Enterobacteriales; Enterobacteriaceae; Salmonella
AF527608	EBI	RVG	G860	41,724	47.1	Salmonella phage P22-pbi	Viruses; dsDNA viruses, no RNA stage; Caudovirales; Podoviridae; P22likevirus.	200913	-
NC_002371	RefSeq	RVG	G860	41,724	47.1	Enterobacteria phage P22	Viruses; dsDNA viruses, no RNA stage; Caudovirales; Podoviridae; P22likevirus.	10754	Bacteria; Proteobacteria; Gammaproteobacteria; Enterobacteriales; Enterobacteriaceae; Salmonella; Salmonella enterica
NC_018275	RefSeq	RVG	G860	39,783	47.7	Salmonella phage vB_SemP_Emek	Viruses; dsDNA viruses, no RNA stage; Caudovirales; Podoviridae; P22likevirus.	1168548	Bacteria; Proteobacteria; Gammaproteobacteria; Enterobacteriales; Enterobacteriaceae
NC_013059	RefSeq	RVG	G860	40,975	47.4	Salmonella phage g341c	Viruses; dsDNA viruses, no RNA stage; Caudovirales; Podoviridae; P22likevirus.	590739	Bacteria; Proteobacteria; Gammaproteobacteria; Enterobacteriales; Enterobacteriaceae; Salmonella; Salmonella enterica
NC_011976	RefSeq	RVG	G860	43,016	47.3	Salmonella phage epsilon34	Viruses; dsDNA viruses, no RNA stage; Caudovirales; Podoviridae; P22likevirus.	348058	Bacteria; Proteobacteria; Gammaproteobacteria; Enterobacteriales; Enterobacteriaceae; Salmonella; Salmonella enterica
NC_028696	RefSeq	RVG	G860	41,337	47.8	Salmonella phage SEN22	Viruses; dsDNA viruses, no RNA stage; Caudovirales; Podoviridae.	1647458	-
NC_019501	RefSeq	RVG	G860	39,646	47.5	Enterobacteria phage IME10	Viruses; dsDNA viruses, no RNA stage; Caudovirales; Podoviridae.	1090133	Bacteria; Proteobacteria; Gammaproteobacteria; Enterobacteriales; Enterobacteriaceae; Escherichia
NC_005344	RefSeq	RVG	G860	39,043	47.5	Shigella phage Sf6	Viruses; dsDNA viruses, no RNA stage; Caudovirales; Podoviridae; P22likevirus.	10761	Bacteria; Proteobacteria; Gammaproteobacteria; Enterobacteriales; Enterobacteriaceae; Shigella
NC_002730	RefSeq	RVG	G860	38,297	46.7	Salmonella phage HK620	Viruses; dsDNA viruses, no RNA stage; Caudovirales; Podoviridae; P22likevirus.	155148	Bacteria; Proteobacteria; Gammaproteobacteria; Enterobacteriales; Enterobacteriaceae; Escherichia
CP000711	EBI	RVG	G860	40,207	46.9	Enterobacteria phage CUS-3	Viruses; dsDNA viruses, no RNA stage; Caudovirales; Podoviridae; P22likevirus.	539221	-
NC_027398	RefSeq	RVG	G860	38,742	47.4	Enterobacteria phage Sf101	Viruses; dsDNA viruses, no RNA stage; Caudovirales; Podoviridae.	1524881	Bacteria; Proteobacteria; Gammaproteobacteria; Enterobacteriales; Enterobacteriaceae; Shigella
NC_011551	RefSeq	RVG	G861	39,867	42.9	Bacteriophage APSE-2	Viruses; dsDNA viruses, no RNA stage; Caudovirales; Podoviridae.	340054	Bacteria; Proteobacteria; Gammaproteobacteria; Enterobacteriales; Enterobacteriaceae; Candidatus Hamiltonella
NC_000935	RefSeq	RVG	G861	36,524	43.9	Endosymbiont phage APSE-1	Viruses; dsDNA viruses, no RNA stage; Caudovirales; Podoviridae.	67571	Bacteria
NC_007902	RefSeq	RVG	G861	52,162	50.8	Sodalis phage phiSG1	Viruses; unclassified phages.	373126	Bacteria; Proteobacteria; Gammaproteobacteria; Enterobacteriales; Enterobacteriaceae; Sodalis
KF591601	EBI	RVG	G862	41,510	59.2	Enterobacteria phage IME_EC2	Viruses; unclassified phages.	1414766	-
TARA_ERS490346_N000352	TOV	EVG	G863	41,270	57	-	-	-	-
TARA_ERS490452_N000243	TOV	EVG	G863	41,270	57	-	-	-	-
TARA_ERS490388_N000395	TOV	EVG	G863	41,270	57	-	-	-	-
NC_022915	RefSeq	RVG	G864	40,471	59.6	Ralstonia phage RSK1	Viruses; dsDNA viruses, no RNA stage; Caudovirales; Podoviridae.	1417599	Bacteria; Proteobacteria; Betaproteobacteria; Burkholderiales; Burkholderiaceae; Ralstonia
TARA_ERS488929_N000256	TOV	EVG	G865	34,532	48.8	-	-	-	-
TARA_ERS490204_N000338	TOV	EVG	G865	36,712	43.7	-	-	-	-
TARA_ERS491107_N000216	TOV	EVG	G866	39,946	45	-	-	-	-
NC_023605	RefSeq	RVG	G867	41,529	43.7	Vibrio phage PVA1	Viruses; dsDNA viruses, no RNA stage; Caudovirales; Podoviridae.	1461743	Bacteria; Proteobacteria; Gammaproteobacteria; Vibrionales; Vibrionaceae; Vibrio; Vibrio harveyi group
AP013360	uvMED	EVG	G868	39,847	38.2	uvMED-CGR-U-MedDCM-OCT-S40-C16 (G1)	-	-	-
NC_011703	RefSeq	RVG	G869	66,235	55.6	Pseudomonas phage 14-1	Viruses; dsDNA viruses, no RNA stage; Caudovirales; Myoviridae.	581037	Bacteria; Proteobacteria; Gammaproteobacteria; Pseudomonadales; Pseudomonadaceae; Pseudomonas; Pseudomonas aeruginosa group
NC_028971	RefSeq	RVG	G869	66,111	55.7	Pseudomonas phage DL68	Viruses; dsDNA viruses, no RNA stage; Caudovirales; Myoviridae.	1640974	-
NC_017674	RefSeq	RVG	G869	66,275	55.6	Pseudomonas phage JG024	Viruses; dsDNA viruses, no RNA stage; Caudovirales; Myoviridae.	749447	Bacteria; Proteobacteria; Gammaproteobacteria; Pseudomonadales; Pseudomonadaceae; Pseudomonas; Pseudomonas aeruginosa group
NC_011756	RefSeq	RVG	G869	66,390	55.6	Pseudomonas phage SN	Viruses; dsDNA viruses, no RNA stage; Caudovirales; Myoviridae.	582382	Bacteria; Proteobacteria; Gammaproteobacteria; Pseudomonadales; Pseudomonadaceae; Pseudomonas; Pseudomonas aeruginosa group
NC_028939	RefSeq	RVG	G869	68,871	55.2	Pseudomonas phage vB_Pae_PS44	Viruses; dsDNA viruses, no RNA stage; Caudovirales; Myoviridae.	1542090	-

NC_011165	RefSeq	RVG	G869	64,427	55.5	Pseudomonas phage LBL3	Viruses; dsDNA viruses, no RNA stage; Caudovirales; Myoviridae.	549445	Bacteria; Proteobacteria; Gammaproteobacteria; Pseudomonadales; Pseudomonadaceae; Pseudomonas; Pseudomonas aeruginosa group
LN610588	EBI	RVG	G869	66,326	55.6	Pseudomonas phage vB_PaeM_PAO1_Ab29	Viruses; dsDNA viruses, no RNA stage; Caudovirales; Myoviridae.	1548916	-
NC_026586	RefSeq	RVG	G869	66,299	55.7	Pseudomonas phage vB_PaeM_PAO1_Ab27	Viruses; dsDNA viruses, no RNA stage; Caudovirales; Myoviridae.	1548907	Bacteria; Proteobacteria; Gammaproteobacteria; Pseudomonadales; Pseudomonadaceae; Pseudomonas; Pseudomonas aeruginosa group
NC_019935	RefSeq	RVG	G869	64,144	55.6	Pseudomonas phage KPP12	Viruses; dsDNA viruses, no RNA stage; Caudovirales; Myoviridae.	763998	Bacteria; Proteobacteria; Gammaproteobacteria; Pseudomonadales; Pseudomonadaceae; Pseudomonas; Pseudomonas aeruginosa group
NC_019451	RefSeq	RVG	G869	66,116	55.5	Pseudomonas phage NH-4	Viruses; dsDNA viruses, no RNA stage; Caudovirales; Myoviridae.	1158680	Bacteria; Proteobacteria; Gammaproteobacteria; Pseudomonadales; Pseudomonadaceae; Pseudomonas; Pseudomonas aeruginosa group
NC_011166	RefSeq	RVG	G869	66,530	55.6	Pseudomonas phage LMA2	Viruses; dsDNA viruses, no RNA stage; Caudovirales; Myoviridae.	549446	Bacteria; Proteobacteria; Gammaproteobacteria; Pseudomonadales; Pseudomonadaceae; Pseudomonas; Pseudomonas aeruginosa group
KF981875	EBI	RVG	G869	65,729	55	Pseudomonas phage SPM-1	Viruses; dsDNA viruses, no RNA stage; Caudovirales; Myoviridae.	1453336	-
NC_007810	RefSeq	RVG	G869	66,015	54.9	Pseudomonas phage F8	Viruses; dsDNA viruses, no RNA stage; Caudovirales; Myoviridae.	347329	Bacteria; Proteobacteria; Gammaproteobacteria; Pseudomonadales; Pseudomonadaceae; Pseudomonas; Pseudomonas aeruginosa group
JQ067083	EBI	RVG	G869	66,450	55.1	Pseudomonas phage PaMx13	Viruses; dsDNA viruses, no RNA stage; Caudovirales; Myoviridae.	1175653	-
NC_011810	RefSeq	RVG	G869	65,764	54.9	Pseudomonas phage PB1	Viruses; dsDNA viruses, no RNA stage; Caudovirales; Myoviridae.	538398	Bacteria; Proteobacteria; Gammaproteobacteria; Pseudomonadales; Pseudomonadaceae; Pseudomonas; Pseudomonas aeruginosa group
NC_028745	RefSeq	RVG	G869	66,103	54.9	Pseudomonas phage DL60	Viruses; dsDNA viruses, no RNA stage; Caudovirales; Myoviridae.	1640970	-
NC_026600	RefSeq	RVG	G869	66,181	54.9	Pseudomonas phage vB_PaeM_C1-14_Ab28	Viruses; dsDNA viruses, no RNA stage; Caudovirales; Myoviridae.	1548917	Bacteria; Proteobacteria; Gammaproteobacteria; Pseudomonadales; Pseudomonadaceae; Pseudomonas; Pseudomonas aeruginosa group
NC_025441	RefSeq	RVG	G869	66,854	46.2	Escherichia phage ECML-117	Viruses; dsDNA viruses, no RNA stage; Caudovirales; Myoviridae.	1204521	Bacteria; Proteobacteria; Gammaproteobacteria; Enterobacteriales; Enterobacteriaceae; Escherichia
NC_009015	RefSeq	RVG	G869	72,415	55.9	Burkholderia phage BcepF1	Viruses; dsDNA viruses, no RNA stage; Caudovirales; Myoviridae.	417280	Bacteria; Proteobacteria; Betaproteobacteria; Burkholderiales; Burkholderiaceae; Burkholderia; Burkholderia cepacia complex
NC_005886	RefSeq	RVG	G870	47,399	54.5	Burkholderia phage BcepB1A	Viruses; dsDNA viruses, no RNA stage; Caudovirales; Myoviridae.	279530	Bacteria; Proteobacteria; Betaproteobacteria; Burkholderiales; Burkholderiaceae; Burkholderia; Burkholderia cepacia complex
NC_025458	RefSeq	RVG	G871	43,510	42.7	Shewanella sp. phage 1/41	Viruses; dsDNA viruses, no RNA stage; Caudovirales; Myoviridae.	1458861	Bacteria; Proteobacteria; Gammaproteobacteria; Alteromonadales; Shewanellaceae; Shewanella
NC_028855	RefSeq	RVG	G872	44,846	37.6	Acinetobacter phage YMC11/12/R2315	Viruses; dsDNA viruses, no RNA stage; Caudovirales; Myoviridae.	1628720	-
NC_024785	RefSeq	RVG	G872	44,844	37.6	Acinetobacter phage YMC-13-01-C62	Viruses; dsDNA viruses, no RNA stage; Caudovirales; Myoviridae.	1505225	Bacteria; Proteobacteria; Gammaproteobacteria; Pseudomonadales; Moraxellaceae; Acinetobacter; Acinetobacter calcoaceticus/baumannii complex
JX976549	EBI	RVG	G872	43,665	37.5	Acinetobacter phage IME-AB2	Viruses; dsDNA viruses, no RNA stage; Caudovirales; Myoviridae.	1243183	-
HM368260	EBI	RVG	G872	45,159	37.7	Acinetobacter phage AB1	Viruses; dsDNA viruses, no RNA stage; Caudovirales; Myoviridae.	889876	-
NC_017984	RefSeq	RVG	G872	46,387	37.7	Acinetobacter bacteriophage AP22	Viruses; dsDNA viruses, no RNA stage; Caudovirales; Myoviridae.	1187128	Bacteria; Proteobacteria; Gammaproteobacteria; Pseudomonadales; Moraxellaceae; Acinetobacter; Acinetobacter calcoaceticus/baumannii complex
NC_028995	RefSeq	RVG	G872	43,216	38.5	Acinetobacter phage phiAC-1	Viruses; dsDNA viruses, no RNA stage; Caudovirales; Myoviridae.	1229760	-
NC_026611	RefSeq	RVG	G873	43,129	51.3	Edwardsiella phage GF-2	Viruses; dsDNA viruses, no RNA stage; Caudovirales; Myoviridae.	1537091	Bacteria; Proteobacteria; Gammaproteobacteria; Enterobacteriales; Enterobacteriaceae; Edwardsiella
NC_005263	RefSeq	RVG	G874	48,177	63.6	Burkholderia phage Bcep1	Viruses; dsDNA viruses, no RNA stage; Caudovirales; Myoviridae.	244310	Bacteria; Proteobacteria; Betaproteobacteria; Burkholderiales; Burkholderiaceae; Burkholderia; Burkholderia cepacia complex
NC_009604	RefSeq	RVG	G874	47,382	63.6	Burkholderia phage BcepNY3	Viruses; dsDNA viruses, no RNA stage; Caudovirales; Myoviridae.	446807	Bacteria; Proteobacteria; Betaproteobacteria; Burkholderiales; Burkholderiaceae; Burkholderia; Burkholderia cepacia complex
NC_004333	RefSeq	RVG	G874	48,247	63.3	Burkholderia phage Bcep781	Viruses; dsDNA viruses, no RNA stage; Caudovirales; Myoviridae.	209052	Bacteria; Proteobacteria; Betaproteobacteria; Burkholderiales; Burkholderiaceae; Burkholderia; Burkholderia cepacia complex
NC_005342	RefSeq	RVG	G874	48,024	63.4	Burkholderia phage Bcep43	Viruses; dsDNA viruses, no RNA stage; Caudovirales; Myoviridae.	260373	Bacteria; Proteobacteria; Betaproteobacteria; Burkholderiales; Burkholderiaceae; Burkholderia; Burkholderia cepacia complex
NC_007710	RefSeq	RVG	G874	46,643	60.9	Xanthomonas phage OP2	Viruses; dsDNA viruses, no RNA stage; Caudovirales; Myoviridae.	331627	Bacteria; Proteobacteria; Gammaproteobacteria; Xanthomonadales; Xanthomonadaceae; Xanthomonas
NC_019527	RefSeq	RVG	G875	43,551	55.4	Aeromonas phage vB_AsaM-56	Viruses; dsDNA viruses, no RNA stage; Caudovirales; Myoviridae.	1127514	Bacteria; Proteobacteria; Gammaproteobacteria; Aeromonadales; Aeromonadaceae; Aeromonas
TARA_ERS488354_N000110	TOV	EVG	G876	48,784	45.4	-	-	-	-
TARA_ERS488340_N000434	TOV	EVG	G876	48,784	45.4	-	-	-	-
TARA_ERS478007_N000054	TOV	EVG	G876	48,784	45.4	-	-	-	-
NC_019518	RefSeq	RVG	G876	44,485	45.6	Vibrio phage vB_VchM-138	Viruses; dsDNA viruses, no RNA stage; Caudovirales; Myoviridae.	1127518	Bacteria; Proteobacteria; Gammaproteobacteria; Vibrionales; Vibrionaceae; Vibrio
NC_019457	RefSeq	RVG	G876	44,492	45.4	Vibrio phage CP-T1	Viruses; dsDNA viruses, no RNA stage; Caudovirales; Myoviridae.	10689	Bacteria; Proteobacteria; Gammaproteobacteria; Vibrionales; Vibrionaceae; Vibrio
KJ021043	EBI	RVG	G876	35,560	48.8	Klebsiella phage Kpn112	Viruses; dsDNA viruses, no RNA stage; Caudovirales; Myoviridae.	1481110	-
NC_020204	RefSeq	RVG	G876	48,814	48.5	Klebsiella phage JD001	Viruses; dsDNA viruses, no RNA stage; Caudovirales; Myoviridae.	1236000	Bacteria; Proteobacteria; Gammaproteobacteria; Enterobacteriales; Enterobacteriaceae; Klebsiella
NC_020082	RefSeq	RVG	G876	42,746	53.1	Edwardsiella phage MSW-3	Viruses; dsDNA viruses, no RNA stage; Caudovirales; Myoviridae.	1264700	Bacteria; Proteobacteria; Gammaproteobacteria; Enterobacteriales; Enterobacteriaceae; Edwardsiella
NC_021342	RefSeq	RVG	G876	43,378	52.6	Edwardsiella phage PEi21	Viruses; dsDNA viruses, no RNA stage; Caudovirales; Myoviridae.	1325372	Bacteria; Proteobacteria; Gammaproteobacteria; Enterobacteriales; Enterobacteriaceae; Edwardsiella
NC_011142	RefSeq	RVG	G876	47,453	46.1	Iodobacteriophage phiPLPE	Viruses; dsDNA viruses, no RNA stage; Caudovirales; Myoviridae.	551895	Bacteria; Proteobacteria; Betaproteobacteria; Neisseriales; Chromobacteriaceae; Iodobacter
NC_019522	RefSeq	RVG	G877	48,454	50.2	Pectobacterium phage ZF40	Viruses; dsDNA viruses, no RNA stage; Caudovirales; Myoviridae.	1127516	Bacteria; Proteobacteria; Gammaproteobacteria; Enterobacteriales; Enterobacteriaceae; Pectobacterium
TARA_ERS488499_N000141	TOV	EVG	G878	50,377	43.7	-	-	-	-
TARA_ERS490204_N000138	TOV	EVG	G879	55,517	57.4	-	-	-	-
OBV_N00080	OBV	EVG	G880	40,816	50.7	-	-	-	-
NC_019519	RefSeq	RVG	G881	69,391	52.4	Agrobacterium phage 7-7-1	Viruses; dsDNA viruses, no RNA stage; Caudovirales; Myoviridae.	1161931	Bacteria; Proteobacteria; Alphaproteobacteria; Rhizobiales; Rhizobiaceae; Agrobacterium
NC_023566	RefSeq	RVG	G882	56,024	47.9	Rhizobium phage vB_RglS_P106B	Viruses; dsDNA viruses, no RNA stage; Caudovirales; Siphoviridae.	1458697	Bacteria; Proteobacteria; Alphaproteobacteria; Rhizobiales; Rhizobiaceae; Rhizobium
NC_025463	RefSeq	RVG	G883	49,640	39.8	Shewanella sp. phage 1/44	Viruses; dsDNA viruses, no RNA stage; Caudovirales; Myoviridae.	1458862	Bacteria; Proteobacteria; Gammaproteobacteria; Alteromonadales; Shewanellaceae; Shewanella

NC_025443	RefSeq	RVG	G884	52,869	42.9	Salmonella phage 9NA	Viruses; dsDNA viruses, no RNA stage; Caudovirales; Siphoviridae.	1113547	Bacteria; Proteobacteria; Gammaproteobacteria; Enterobacteriales; Enterobacteriaceae; Salmonella; Salmonella enterica
NC_004827	RefSeq	RVG	G885	43,033	42.5	Haemophilus phage Aaphi23	Viruses; dsDNA viruses, no RNA stage; Caudovirales; Myoviridae.	230158	Bacteria; Proteobacteria; Gammaproteobacteria; Pasteurellales; Pasteurellaceae; Aggregatibacter
NC_013597	RefSeq	RVG	G885	43,970	42.4	Aggregatibacter phage S1249	Viruses; dsDNA viruses, no RNA stage; Caudovirales; Myoviridae.	683735	Bacteria; Proteobacteria; Gammaproteobacteria; Pasteurellales; Pasteurellaceae; Aggregatibacter
NC_020841	RefSeq	RVG	G886	40,803	44.1	Psychrobacter phage pOW20-A	Viruses; dsDNA viruses, no RNA stage; Caudovirales; Myoviridae.	754048	Bacteria; Proteobacteria; Gammaproteobacteria; Pseudomonadales; Moraxellaceae; Psychrobacter
NC_028699	RefSeq	RVG	G887	40,740	49.9	Salmonella phage SEN34	Viruses; dsDNA viruses, no RNA stage; Caudovirales; Podoviridae.	1647463	-
NC_015295	RefSeq	RVG	G888	47,279	47.3	Erwinia phage phiEt88	Viruses; dsDNA viruses, no RNA stage; Caudovirales; Myoviridae.	925984	Bacteria; Proteobacteria; Gammaproteobacteria; Enterobacteriales; Enterobacteriaceae; Erwinia
NC_017981	RefSeq	RVG	G888	49,981	52.4	Xanthomonas phage vB_XveM_DIBBI	Viruses; dsDNA viruses, no RNA stage; Caudovirales; Myoviridae.	1129194	Bacteria; Proteobacteria; Gammaproteobacteria; Xanthomonadales; Xanthomonadaceae; Xanthomonas
LDNO01000007	cryoconite	EVG	G889	42,540	52.3	-	-	-	-
NC_020850	RefSeq	RVG	G890	38,374	42.3	Vibrio phage VBM1	Viruses; dsDNA viruses, no RNA stage; unclassified dsDNA phages.	754074	Bacteria; Proteobacteria; Gammaproteobacteria; Vibrionales; Vibrionaceae; Vibrio; Vibrio harveyi group
NC_007806	RefSeq	RVG	G891	42,999	53.6	Pseudomonas phage 73	Viruses; dsDNA viruses, no RNA stage; Caudovirales; Siphoviridae.	347325	Bacteria; Proteobacteria; Gammaproteobacteria; Pseudomonadales; Pseudomonadaceae; Pseudomonas; Pseudomonas aeruginosa group
NC_029019	RefSeq	RVG	G891	42,593	53.7	Stenotrophomonas phage vB_SmaS-DLP_2	Viruses; dsDNA viruses, no RNA stage; Caudovirales; Siphoviridae.	1642663	-
NC_017864	RefSeq	RVG	G891	42,844	53.8	Pseudomonas phage vB_Pae-Kakheti25	Viruses; dsDNA viruses, no RNA stage; Caudovirales; Siphoviridae.	1141526	Bacteria; Proteobacteria; Gammaproteobacteria; Pseudomonadales; Pseudomonadaceae; Pseudomonas; Pseudomonas aeruginosa group
NC_024381	RefSeq	RVG	G891	43,056	53.4	Pseudomonas phage vB_PaeS_SCH_Ab26	Viruses; dsDNA viruses, no RNA stage; Caudovirales; Siphoviridae.	1476390	Bacteria; Proteobacteria; Gammaproteobacteria; Pseudomonadales; Pseudomonadaceae; Pseudomonas; Pseudomonas aeruginosa group
NC_028879	RefSeq	RVG	G891	43,225	54.6	Pseudomonas phage PaMx42	Viruses; dsDNA viruses, no RNA stage; Caudovirales; Siphoviridae.	1175662	-
NC_018278	RefSeq	RVG	G891	42,832	54.6	Burkholderia phage KL1	Viruses; dsDNA viruses, no RNA stage; Caudovirales; Siphoviridae.	1132026	Bacteria; Proteobacteria; Betaproteobacteria; Burkholderiales; Burkholderiaceae; Burkholderia; Burkholderia cepacia complex
NC_029097	RefSeq	RVG	G891	44,496	55.1	Rhodobacter phage RcTitan	Viruses; dsDNA viruses, no RNA stage; Caudovirales.	1662330	-
NC_023590	RefSeq	RVG	G891	43,050	45.5	Acinetobacter phage IME_AB3	Viruses; dsDNA viruses, no RNA stage; Caudovirales.	1458669	Bacteria; Proteobacteria; Gammaproteobacteria; Pseudomonadales; Moraxellaceae; Acinetobacter; Acinetobacter calcoaceticus/baumannii complex
NC_026608	RefSeq	RVG	G891	42,093	56.4	Paracoccus phage vB_PmaS_IMEP1	Viruses; dsDNA viruses, no RNA stage; Caudovirales; Siphoviridae.	1561065	Bacteria; Proteobacteria; Alphaproteobacteria; Rhodobacterales; Rhodobacteraceae; Paracoccus
NC_029033	RefSeq	RVG	G891	45,045	56	Achromobacter phage phiAxp-1	Viruses; dsDNA viruses, no RNA stage; Caudovirales; Siphoviridae.	1610509	-
NC_029028	RefSeq	RVG	G892	60,754	43.2	Enterobacteria phage JenP1	Viruses; dsDNA viruses, no RNA stage; Caudovirales; Siphoviridae.	1610837	-
NC_028997	RefSeq	RVG	G892	59,802	43.2	Enterobacteria phage JenP2	Viruses; dsDNA viruses, no RNA stage; Caudovirales; Siphoviridae.	1610838	-
NC_029021	RefSeq	RVG	G892	60,747	43.9	Enterobacteria phage JenK1	Viruses; dsDNA viruses, no RNA stage; Caudovirales; Siphoviridae.	1610836	-
NC_024146	RefSeq	RVG	G892	56,702	43.9	Enterobacteria phage 9g	Viruses; dsDNA viruses, no RNA stage; Caudovirales; Siphoviridae.	1468411	Bacteria; Proteobacteria; Gammaproteobacteria; Enterobacteriales; Enterobacteriaceae; Escherichia
NC_028831	RefSeq	RVG	G892	61,235	44.4	Escherichia phage slur01	Viruses; dsDNA viruses, no RNA stage; Caudovirales; Siphoviridae.	1720493	-
NC_027378	RefSeq	RVG	G892	56,776	44.6	Escherichia phage Seurat	Viruses; dsDNA viruses, no RNA stage; Caudovirales; Siphoviridae.	1540098	Bacteria; Proteobacteria; Gammaproteobacteria; Enterobacteriales; Enterobacteriaceae; Escherichia
NC_028776	RefSeq	RVG	G892	59,670	44.7	Enterobacteria phage CAjan	Viruses; dsDNA viruses, no RNA stage; Caudovirales; Siphoviridae.	1610828	-
JQ067084	EBI	RVG	G892	57,620	58.5	Pseudomonas phage PaMx25	Viruses; dsDNA viruses, no RNA stage; Caudovirales; Siphoviridae.	1175654	-
NC_026610	RefSeq	RVG	G893	56,637	51.3	Vibrio phage VpKK5	Viruses; dsDNA viruses, no RNA stage; Caudovirales; Siphoviridae.	1538804	Bacteria; Proteobacteria; Gammaproteobacteria; Vibrionales; Vibrionaceae; Vibrio; Vibrio harveyi group
NC_028834	RefSeq	RVG	G894	48,216	54.9	Achromobacter phage 83-24	Viruses; dsDNA viruses, no RNA stage; Caudovirales; Siphoviridae.	1589747	-
NC_028768	RefSeq	RVG	G894	49,714	55.4	Achromobacter phage JWX	Viruses; dsDNA viruses, no RNA stage; Caudovirales; Siphoviridae.	1589746	-
NC_009447	RefSeq	RVG	G895	52,414	56.3	Burkholderia phage BcepGomr	Viruses; dsDNA viruses, no RNA stage; Caudovirales; Siphoviridae.	437329	Bacteria; Proteobacteria; Betaproteobacteria; Burkholderiales; Burkholderiaceae; Burkholderia; Burkholderia cepacia complex
JX483877	EBI	RVG	G896	50,426	56.4	Rhizobium phage RHEph05	Viruses; dsDNA viruses, no RNA stage; Caudovirales; Myoviridae.	1220605	-
NC_027296	RefSeq	RVG	G896	53,721	56.4	Rhizobium phage RHEph06	Viruses; dsDNA viruses, no RNA stage; Caudovirales; Myoviridae.	1220714	Bacteria; Proteobacteria; Alphaproteobacteria; Rhizobiales; Rhizobiaceae; Rhizobium
JX483876	EBI	RVG	G896	53,018	56.4	Rhizobium phage RHEph04	Viruses; dsDNA viruses, no RNA stage; Caudovirales; Myoviridae.	1220604	-
LN610585	EBI	RVG	G897	57,745	63.5	Pseudomonas phage vB_PaeS_PAO1_Ab20	Viruses; dsDNA viruses, no RNA stage; Caudovirales; Siphoviridae.	1548913	-
NC_026594	RefSeq	RVG	G897	56,537	63.5	Pseudomonas phage vB_PaeS_PAO1_Ab18	Viruses; dsDNA viruses, no RNA stage; Caudovirales; Siphoviridae.	1548905	Bacteria; Proteobacteria; Gammaproteobacteria; Pseudomonadales; Pseudomonadaceae; Pseudomonas; Pseudomonas aeruginosa group
LN610584	EBI	RVG	G897	58,139	63.3	Pseudomonas phage vB_PaeS_PAO1_Ab19	Viruses; dsDNA viruses, no RNA stage; Caudovirales; Siphoviridae.	1548912	-
NC_028770	RefSeq	RVG	G897	59,878	64.5	Pseudomonas phage PaMx11	Viruses; dsDNA viruses, no RNA stage; Caudovirales; Siphoviridae.	1175657	-
NC_010116	RefSeq	RVG	G897	58,663	64.3	Pseudomonas phage YuA	Viruses; dsDNA viruses, no RNA stage; Caudovirales; Siphoviridae; Yualikevirus.	462590	Bacteria; Proteobacteria; Gammaproteobacteria; Pseudomonadales; Pseudomonadaceae; Pseudomonas; Pseudomonas aeruginosa group
NC_018282	RefSeq	RVG	G897	61,167	64.3	Pseudomonas phage MP1412	Viruses; dsDNA viruses, no RNA stage; Caudovirales; Siphoviridae.	1204517	Bacteria; Proteobacteria; Gammaproteobacteria; Pseudomonadales; Pseudomonadaceae; Pseudomonas; Pseudomonas aeruginosa group
NC_007809	RefSeq	RVG	G897	59,446	64.5	Pseudomonas phage M6	Viruses; dsDNA viruses, no RNA stage; Caudovirales; Siphoviridae; Yualikevirus.	347328	Bacteria; Proteobacteria; Gammaproteobacteria; Pseudomonadales; Pseudomonadaceae; Pseudomonas; Pseudomonas aeruginosa group
NC_028980	RefSeq	RVG	G897	62,181	64.2	Pseudomonas phage PAE1	Viruses; dsDNA viruses, no RNA stage; Caudovirales; Siphoviridae.	1718273	-
NC_028809	RefSeq	RVG	G897	58,637	68.4	Pseudomonas phage PaMx74	Viruses; dsDNA viruses, no RNA stage; Caudovirales; Siphoviridae.	1175663	-
NC_028931	RefSeq	RVG	G897	55,108	66.4	Pseudomonas phage PaMx28	Viruses; dsDNA viruses, no RNA stage; Caudovirales; Siphoviridae.	1175659	-
NC_006938	RefSeq	RVG	G898	63,649	62.1	Phage phiJL001	Viruses; dsDNA viruses, no RNA stage; Caudovirales; Siphoviridae; Yualikevirus.	279383	Bacteria; Proteobacteria; Alphaproteobacteria
NC_015466	RefSeq	RVG	G899	62,668	57.9	Roseobacter phage RDJL Phi 1	Viruses; dsDNA viruses, no RNA stage; Caudovirales; Siphoviridae.	562742	Bacteria; Proteobacteria; Alphaproteobacteria; Rhodobacterales; Rhodobacteraceae; Roseobacter
TARA_ERS490953_N000037	TOV	EVG	G900	61,463	55.6	-	-	-	-
TARA_ERS488354_N000169	TOV	EVG	G901	40,072	57.7	-	-	-	-
TARA_ERS489148_N000171	TOV	EVG	G902	43,135	50.6	-	-	-	-
TARA_ERS490953_N000118	TOV	EVG	G903	39,894	42.6	-	-	-	-

NC_024204	RefSeq	RVG	G904	42,764	49.8	Salmonella phage vB_SenS-Ent3	Viruses; dsDNA viruses, no RNA stage; Caudovirales; Siphoviridae.	1465613	Bacteria; Proteobacteria; Gammaproteobacteria; Enterobacteriales; Enterobacteriaceae; Salmonella; Salmonella enterica
NC_019539	RefSeq	RVG	G904	42,391	49.8	Salmonella phage Ent1	Viruses; dsDNA viruses, no RNA stage; Caudovirales; Siphoviridae; Jerseylikevirus.	1163482	Bacteria; Proteobacteria; Gammaproteobacteria; Enterobacteriales; Enterobacteriaceae; Salmonella; Salmonella enterica
NC_023608	RefSeq	RVG	G904	42,093	49.9	Salmonella phage vB_SenS-Ent2	Viruses; dsDNA viruses, no RNA stage; Caudovirales; Siphoviridae.	1465618	Bacteria; Proteobacteria; Gammaproteobacteria; Enterobacteriales; Enterobacteriaceae; Salmonella; Salmonella enterica
NC_028698	RefSeq	RVG	G904	41,868	49.8	Salmonella phage f18SE	Viruses; dsDNA viruses, no RNA stage; Caudovirales; Siphoviridae.	1611545	-
NC_021317	RefSeq	RVG	G904	21,248	50.1	Salmonella phage L13	Viruses; dsDNA viruses, no RNA stage; Caudovirales; Siphoviridae.	1325963	Bacteria; Proteobacteria; Gammaproteobacteria; Enterobacteriales; Enterobacteriaceae; Salmonella; Salmonella enterica
JX202565	EBI	RVG	G904	42,633	49.8	Salmonella phage wksl3	Viruses; dsDNA viruses, no RNA stage; Caudovirales; Siphoviridae; Jerseylikevirus.	1204541	-
NC_006940	RefSeq	RVG	G904	40,793	50.1	Salmonella phage SS3e	Viruses; dsDNA viruses, no RNA stage; Caudovirales; Siphoviridae; Jerseylikevirus.	293644	Bacteria; Proteobacteria; Gammaproteobacteria; Enterobacteriales; Enterobacteriaceae; Salmonella; Salmonella enterica
NC_016763	RefSeq	RVG	G904	43,221	49.6	Salmonella phage SE2	Viruses; dsDNA viruses, no RNA stage; Caudovirales; Siphoviridae; Jerseylikevirus.	1115478	Bacteria; Proteobacteria; Gammaproteobacteria; Enterobacteriales; Enterobacteriaceae; Salmonella; Salmonella enterica
NC_022754	RefSeq	RVG	G904	42,749	49.9	Salmonella phage SETP7	Viruses; dsDNA viruses, no RNA stage; Caudovirales; Siphoviridae.	424947	Bacteria; Proteobacteria; Gammaproteobacteria; Enterobacteriales; Enterobacteriaceae; Salmonella; Salmonella enterica
NC_022752	RefSeq	RVG	G904	42,665	49.8	Salmonella phage SETP13	Viruses; dsDNA viruses, no RNA stage; Caudovirales; Siphoviridae.	424949	Bacteria; Proteobacteria; Gammaproteobacteria; Enterobacteriales; Enterobacteriaceae; Salmonella; Salmonella enterica
JX297445	EBI	RVG	G904	41,546	49.9	Salmonella phage vB_SenS_AG11	Viruses; dsDNA viruses, no RNA stage; Caudovirales; Siphoviridae.	1211279	-
NC_009232	RefSeq	RVG	G904	42,572	49.9	Salmonella phage SETP3	Viruses; dsDNA viruses, no RNA stage; Caudovirales; Siphoviridae; Jerseylikevirus.	424944	Bacteria; Proteobacteria; Gammaproteobacteria; Enterobacteriales; Enterobacteriaceae; Salmonella; Salmonella enterica
NC_021777	RefSeq	RVG	G904	43,447	50	Salmonella phage Jersey	Viruses; dsDNA viruses, no RNA stage; Caudovirales; Siphoviridae; Jerseylikevirus.	1340534	Bacteria; Proteobacteria; Gammaproteobacteria; Enterobacteriales; Enterobacteriaceae; Salmonella; Salmonella enterica
NC_026017	RefSeq	RVG	G904	41,880	50.5	Salmonella phage LSPA1	Viruses; dsDNA viruses, no RNA stage; Caudovirales; Siphoviridae.	1540823	Bacteria; Proteobacteria; Gammaproteobacteria; Enterobacteriales; Enterobacteriaceae; Salmonella; Salmonella enterica
GU196281	EBI	RVG	G904	43,461	51.2	Escherichia phage K1-ind(3)	Viruses; dsDNA viruses, no RNA stage; Caudovirales; Siphoviridae.	698490	-
GU196280	EBI	RVG	G904	42,765	51.3	Escherichia phage K1-ind(2)	Viruses; dsDNA viruses, no RNA stage; Caudovirales; Siphoviridae.	698489	-
GU196279	EBI	RVG	G904	42,292	51.3	Escherichia phage K1-ind(1)	Viruses; dsDNA viruses, no RNA stage; Caudovirales; Siphoviridae.	698488	-
NC_027994	RefSeq	RVG	G904	41,632	51.2	Escherichia phage K1-dep(1)	Viruses; dsDNA viruses, no RNA stage; Caudovirales; Siphoviridae.	698487	Bacteria; Proteobacteria; Gammaproteobacteria; Enterobacteriales; Enterobacteriaceae; Escherichia
NC_027993	RefSeq	RVG	G904	43,587	51.1	Escherichia phage K1-dep(4)	Viruses; dsDNA viruses, no RNA stage; Caudovirales; Siphoviridae.	698486	Bacteria; Proteobacteria; Gammaproteobacteria; Enterobacteriales; Enterobacteriaceae; Escherichia
NC_021775	RefSeq	RVG	G904	42,215	51.1	Salmonella phage FSL SP-031	Viruses; dsDNA viruses, no RNA stage; Caudovirales; Siphoviridae.	1173749	Bacteria; Proteobacteria; Gammaproteobacteria; Enterobacteriales; Enterobacteriaceae; Salmonella; Salmonella enterica
NC_028695	RefSeq	RVG	G904	40,491	52	Enterobacter phage phiEap-2	Viruses; dsDNA viruses, no RNA stage; Caudovirales; Podoviridae.	1701257	-
NC_021563	RefSeq	RVG	G904	42,724	49.9	Serratia phage Eta	Viruses; dsDNA viruses, no RNA stage; Caudovirales; Siphoviridae.	1282995	Bacteria; Proteobacteria; Gammaproteobacteria; Enterobacteriales; Enterobacteriaceae; Serratia
NC_025466	RefSeq	RVG	G904	40,161	42	Shewanella sp. phage 3/49	Viruses; dsDNA viruses, no RNA stage; Caudovirales; Myoviridae.	1458863	Bacteria; Proteobacteria; Gammaproteobacteria; Alteromonadales; Shewanellaceae; Shewanella
TARA_ERS478007_N000261	TOV	EVG	G905	31,063	45.9	-	-	-	-
TARA_ERS489059_N000153	TOV	EVG	G905	35,806	43.6	-	-	-	-
AP013359	uvMED	EVG	G906	37,910	39	uvMED-CGR-U-MedDCM-OCT-S28-C23 (G1)	-	-	-
TARA_ERS488340_N000606	TOV	EVG	G907	38,955	41.2	-	-	-	-
NC_007024	RefSeq	RVG	G908	55,770	51.9	Xanthomonas phage Xp15	Viruses; dsDNA viruses, no RNA stage; Caudovirales.	322855	Bacteria; Proteobacteria; Gammaproteobacteria; Xanthomonadales; Xanthomonadaceae; Xanthomonas
NC_028819	RefSeq	RVG	G909	43,190	41.2	Pseudoalteromonas Phage H103	Viruses; dsDNA viruses, no RNA stage; Caudovirales.	1636200	-
TARA_ERS491107_N000181	TOV	EVG	G909	43,834	39.8	-	-	-	-
KC542353	EBI	RVG	G909	39,940	40.2	Pseudoalteromonas phage TW1	Viruses; dsDNA viruses, no RNA stage; Caudovirales; Siphoviridae.	1366055	-
NC_020846	RefSeq	RVG	G910	46,917	43.9	Vibrio phage pYD21-A	Viruses; dsDNA viruses, no RNA stage; unclassified dsDNA phages.	754049	Bacteria; Proteobacteria; Gammaproteobacteria; Vibrionales; Vibrionaceae; Vibrio
NC_025471	RefSeq	RVG	G911	36,844	51.3	Idiomarinaceae phage Phi1M2-2	Viruses; dsDNA viruses, no RNA stage; Caudovirales; Siphoviridae.	1527515	Bacteria; Proteobacteria; Gammaproteobacteria; Alteromonadales; Idiomarinaceae
TARA_ERS488737_N000195	TOV	EVG	G912	42,981	46.7	-	-	-	-
NC_021561	RefSeq	RVG	G913	37,324	43.1	Vibrio phage pYD38-B	Viruses; dsDNA viruses, no RNA stage; Caudovirales; Siphoviridae.	929835	Bacteria; Proteobacteria; Gammaproteobacteria; Vibrionales; Vibrionaceae; Vibrio
NC_006953	RefSeq	RVG	G913	37,966	44	Listonella phage phiHSIC	Viruses; dsDNA viruses, no RNA stage; Caudovirales; Siphoviridae.	310539	Bacteria; Proteobacteria; Gammaproteobacteria; Vibrionales; Vibrionaceae; Vibrio
HQ632860	CAMERA	RVG	G913	40,557	42.3	Vibrio phage jenny 12G5	Viruses; dsDNA viruses, no RNA stage; Caudovirales; Siphoviridae.	573176	-
KF302032	EBI	RVG	G914	37,730	40.4	Pseudoalteromonas phage HS5	Viruses; dsDNA viruses, no RNA stage; Caudovirales; Podoviridae.	1357709	-
KF302033	EBI	RVG	G914	37,211	40.5	Pseudoalteromonas phage HS1	Viruses; dsDNA viruses, no RNA stage; Caudovirales; Podoviridae.	1357707	-
NC_015293	RefSeq	RVG	G914	30,651	40.8	Pseudoalteromonas phage H105/1	Viruses; dsDNA viruses, no RNA stage; Caudovirales; Siphoviridae.	877240	Bacteria; Proteobacteria; Gammaproteobacteria; Alteromonadales; Pseudoalteromonadaceae; Pseudoalteromonas
KF302036	EBI	RVG	G915	38,208	40.2	Pseudoalteromonas phage HS2	Viruses; unclassified phages.	1357708	-
TARA_ERS490120_N000648	TOV	EVG	G916	31,281	47.1	-	-	-	-
TARA_ERS490204_N000491	TOV	EVG	G916	31,281	47.1	-	-	-	-
TARA_ERS488836_N000160	TOV	EVG	G917	36,547	52.7	-	-	-	-
TARA_ERS488448_N000430	TOV	EVG	G918	36,559	39.4	-	-	-	-
TARA_ERS490953_N000110	TOV	EVG	G919	40,731	53.8	-	-	-	-
NC_020844	RefSeq	RVG	G920	40,695	56.4	Salicola phage CGphi29	Viruses; dsDNA viruses, no RNA stage; unclassified dsDNA phages.	754067	Bacteria; Proteobacteria; Gammaproteobacteria; Oceanospirillales; Halomonadaceae
NC_025439	RefSeq	RVG	G921	34,773	52	Idiomarinaceae phage 1N2-2	Viruses; dsDNA viruses, no RNA stage; unclassified dsDNA phages.	1536592	Bacteria; Proteobacteria; Gammaproteobacteria; Alteromonadales; Idiomarinaceae
NC_018269	RefSeq	RVG	G922	31,766	46.6	Marinomonas phage P12026	Viruses; dsDNA viruses, no RNA stage; unclassified dsDNA phages.	1176423	Bacteria; Proteobacteria; Gammaproteobacteria; Oceanospirillales; Oceanospirillaceae
NC_029100	RefSeq	RVG	G923	33,399	40.3	Pseudoalteromonas phage Pq0	Viruses; dsDNA viruses, no RNA stage; Caudovirales; Siphoviridae.	1667322	-
TARA_ERS490204_N000142	TOV	EVG	G924	54,429	45.2	-	-	-	-
TARA_ERS490452_N000144	TOV	EVG	G924	54,429	45.2	-	-	-	-
TARA_ERS490388_N000266	TOV	EVG	G924	54,429	45.2	-	-	-	-
TARA_ERS490346_N000226	TOV	EVG	G924	54,429	45.2	-	-	-	-
TARA_ERS488448_N000126	TOV	EVG	G925	59,933	44.2	-	-	-	-

TARA_ERS488499_N000100	TOV	EVG	G925	59,933	44.2	-	-	-	-
TARA_ERS488518_N000200	TOV	EVG	G925	59,933	44.2	-	-	-	-
TARA_ERS488499_N000162	TOV	EVG	G926	44,867	52.2	-	-	-	-
TARA_ERS488354_N000133	TOV	EVG	G927	45,055	63.4	-	-	-	-
TARA_ERS488340_N000474	TOV	EVG	G927	46,018	63.3	-	-	-	-
TARA_ERS490610_N000446	TOV	EVG	G928	40,192	61.9	-	-	-	-
LDNN01000010	cryoconite	EVG	G929	42,610	42	-	-	-	-
NC_011103	RefSeq	RVG	G930	60,195	59	Rhizobium phage 16-3	Viruses; dsDNA viruses, no RNA stage; Caudovirales; Siphoviridae.	10704	Bacteria; Proteobacteria; Alphaproteobacteria; Rhizobiales; Rhizobiaceae; Sinorhizobium
TARA_ERS490053_N000498	TOV	EVG	G931	30,664	48.1	-	-	-	-
TARA_ERS490120_N000670	TOV	EVG	G931	30,664	48.1	-	-	-	-
TARA_ERS490053_N000492	TOV	EVG	G931	30,877	45.6	-	-	-	-
TARA_ERS490120_N000661	TOV	EVG	G931	30,877	45.6	-	-	-	-
TARA_ERS488836_N000053	TOV	EVG	G932	58,038	44	-	-	-	-
TARA_ERS488892_N000267	TOV	EVG	G933	33,196	37.9	-	-	-	-
TARA_ERS490452_N000347	TOV	EVG	G933	33,196	37.9	-	-	-	-
LDNP01000007	cryoconite	EVG	G934	36,900	57.8	-	-	-	-
NC_029061	RefSeq	RVG	G935	36,315	56.7	Rhodoferax phage P26218	Viruses; dsDNA viruses, no RNA stage; Caudovirales; Podoviridae.	1636270	-
NC_020488	RefSeq	RVG	G936	38,682	49.1	Vibrio phage VvAW1	Viruses; dsDNA viruses, no RNA stage; Caudovirales; Podoviridae.	1168281	Bacteria; Proteobacteria; Gammaproteobacteria; Vibrionales; Vibrionaceae; Vibrio
NC_009990	RefSeq	RVG	G936	37,313	40.9	Thalassomonas phage BA3	Viruses; dsDNA viruses, no RNA stage; Caudovirales; Podoviridae.	469660	Bacteria; Proteobacteria; Gammaproteobacteria; Alteromonadales; Colwelliaceae; Thalassotalea
LDNN01000022	cryoconite	EVG	G937	34,340	36.4	-	-	-	-
TARA_ERS489603_N000261	TOV	EVG	G938	32,040	41.1	-	-	-	-
TARA_ERS490320_N000192	TOV	EVG	G938	32,478	39.8	-	-	-	-
TARA_ERS490452_N000337	TOV	EVG	G939	33,496	52.9	-	-	-	-
TARA_ERS490557_N000584	TOV	EVG	G940	29,015	45.7	-	-	-	-
TARA_ERS488448_N000529	TOV	EVG	G941	33,590	54.5	-	-	-	-
LDNP01000009	cryoconite	EVG	G942	34,925	60.2	-	-	-	-
LDNP01000003	cryoconite	EVG	G943	40,814	60.8	-	-	-	-
NC_019917	RefSeq	RVG	G944	62,952	65.5	Burkholderia phage BcepMigl	Viruses; dsDNA viruses, no RNA stage; Caudovirales; Podoviridae.	1195073	Bacteria; Proteobacteria; Betaproteobacteria; Burkholderiales; Burkholderiaceae; Burkholderia; Burkholderia cepacia complex
NC_005262	RefSeq	RVG	G944	63,882	65.3	Burkholderia phage Bcep22	Viruses; dsDNA viruses, no RNA stage; Caudovirales; Podoviridae.	242527	Bacteria; Proteobacteria; Betaproteobacteria; Burkholderiales; Burkholderiaceae
NC_012743	RefSeq	RVG	G944	62,715	66.2	Burkholderia phage BcepIL02	Viruses; dsDNA viruses, no RNA stage; Caudovirales; Podoviridae.	644524	Bacteria; Proteobacteria; Betaproteobacteria; Burkholderiales; Burkholderiaceae; Burkholderia; Burkholderia cepacia complex
NC_018452	RefSeq	RVG	G944	61,847	66.2	Burkholderia phage DC1	Viruses; dsDNA viruses, no RNA stage; Caudovirales; Podoviridae.	1136535	Bacteria; Proteobacteria; Betaproteobacteria; Burkholderiales; Burkholderiaceae; Burkholderia; Burkholderia cepacia complex
NC_016767	RefSeq	RVG	G945	60,714	62.8	Erwinia phage PEp14	Viruses; dsDNA viruses, no RNA stage; Caudovirales; Podoviridae.	1131315	Bacteria; Proteobacteria; Gammaproteobacteria; Enterobacteriales; Enterobacteriaceae; Erwinia
NC_003324	RefSeq	RVG	G945	57,416	61.5	Sinorhizobium phage PBC5	Viruses; dsDNA viruses, no RNA stage; Caudovirales.	179237	Bacteria; Proteobacteria; Alphaproteobacteria; Rhizobiales; Rhizobiaceae; Sinorhizobium
KC262634	EBI	RVG	G946	65,270	62.6	Pseudomonas phage H66	Viruses; dsDNA viruses, no RNA stage; Caudovirales; Podoviridae.	1273707	-
NC_006552	RefSeq	RVG	G946	65,195	63.2	Pseudomonas phage F116	Viruses; dsDNA viruses, no RNA stage; Caudovirales; Podoviridae.	280701	Bacteria; Proteobacteria; Gammaproteobacteria; Pseudomonadales; Pseudomonadaceae; Pseudomonas; Pseudomonas aeruginosa group
NC_021068	RefSeq	RVG	G947	57,611	39	Vibrio phage douglas 12A4	Viruses; dsDNA viruses, no RNA stage; unclassified dsDNA phages.	573171	Bacteria; Proteobacteria; Gammaproteobacteria; Vibrionales; Vibrionaceae; Aliivibrio
NC_023569	RefSeq	RVG	G948	77,837	46	Vibrio phage SHOU24	Viruses; dsDNA viruses, no RNA stage; Caudovirales; Siphoviridae.	1414739	Bacteria; Proteobacteria; Gammaproteobacteria; Vibrionales; Vibrionaceae; Vibrio; Vibrio harveyi group
LDNP01000006	cryoconite	EVG	G949	37,582	33.9	-	-	-	-
LDNN01000018	cryoconite	EVG	G949	37,329	32.7	-	-	-	-
LDNP01000005	cryoconite	EVG	G949	38,190	36.1	-	-	-	-
LDNN01000028	cryoconite	EVG	G950	15,551	36.9	-	-	-	-
AP013537	uvMED	EVG	G951	34,933	55	uvMED-CGR-C59A-MedDCM-OCT-S34-C49 (G6)	-	-	-
AP013536	uvMED	EVG	G951	34,130	54.1	uvMED-CGR-C59-MedDCM-OCT-S39-C65 (G6)	-	-	-
TARA_ERS490452_N000338	TOV	EVG	G952	33,447	36.2	-	-	-	-
TARA_ERS488340_N000739	TOV	EVG	G952	33,811	51.1	-	-	-	-
TARA_ERS488354_N000243	TOV	EVG	G953	34,316	49.8	-	-	-	-
TARA_ERS488813_N000300	TOV	EVG	G954	32,051	30.4	-	-	-	-
LDNN01000020	cryoconite	EVG	G955	35,860	48.4	-	-	-	-
TARA_ERS488448_N000355	TOV	EVG	G956	40,115	51.5	-	-	-	-
LDNN01000017	cryoconite	EVG	G957	37,647	51.9	-	-	-	-
TARA_ERS491107_N000225	TOV	EVG	G958	38,971	52	-	-	-	-
TARA_ERS491107_N000198	TOV	EVG	G958	41,977	43.3	-	-	-	-
NC_003085	RefSeq	RVG	G959	49,534	67.7	Myxococcus phage Mx8	Viruses; dsDNA viruses, no RNA stage; Caudovirales; Podoviridae.	49964	Bacteria; Proteobacteria; delta/epsilon subdivisions; Deltaproteobacteria; Myxococcales; Cystobacterineae; Myxococcaceae; Myxococcus
TARA_ERS490204_N000118	TOV	EVG	G960	59,571	43.1	-	-	-	-
KC556898	EBI	RVG	G961	38,253	48.2	Brucella phage Wb	Viruses; dsDNA viruses, no RNA stage; Caudovirales; Podoviridae.	1277893	-
KC556894	EBI	RVG	G961	41,142	48.2	Brucella phage Fz	Viruses; dsDNA viruses, no RNA stage; Caudovirales; Podoviridae.	1308870	-
KC556896	EBI	RVG	G961	38,253	48.2	Brucella phage S708	Viruses; dsDNA viruses, no RNA stage; Caudovirales; Podoviridae.	1308871	-
KC556895	EBI	RVG	G961	38,253	48.2	Brucella phage R/C	Viruses; dsDNA viruses, no RNA stage; Caudovirales; Podoviridae.	1277895	-
KC556893	EBI	RVG	G961	38,253	48.2	Brucella phage Bk	Viruses; dsDNA viruses, no RNA stage; Caudovirales; Podoviridae.	1308869	-
HG428758	EBI	RVG	G961	41,143	48.2	Brucella phage F1	Viruses; dsDNA viruses, no RNA stage; Caudovirales; Podoviridae.	1401446	-
NC_019446	RefSeq	RVG	G961	41,148	48.2	Brucella phage Tb	Viruses; dsDNA viruses, no RNA stage; Caudovirales; Podoviridae.	1133292	Bacteria; Proteobacteria; Alphaproteobacteria; Rhizobiales; Brucellaceae; Brucella
NC_019447	RefSeq	RVG	G961	38,253	48.2	Brucella phage Pr	Viruses; dsDNA viruses, no RNA stage; Caudovirales; Podoviridae.	1133293	Bacteria; Proteobacteria; Alphaproteobacteria; Rhizobiales; Brucellaceae; Brucella
TARA_ERS489084_N000174	TOV	EVG	G961	40,225	62	-	-	-	-
TARA_ERS488813_N000187	TOV	EVG	G962	39,192	60	-	-	-	-

NC_020858	RefSeq	RVG	G963	40,929	58.8	Sulfitobacter phage pCB2047-A	Viruses; dsDNA viruses, no RNA stage; unclassified dsDNA phages.	754045	Bacteria; Proteobacteria; Alphaproteobacteria; Rhodobacterales; Rhodobacteraceae; Sulfitobacter
NC_020856	RefSeq	RVG	G963	40,931	59	Sulfitobacter phage pCB2047-C	Viruses; dsDNA viruses, no RNA stage; unclassified dsDNA phages.	754043	Bacteria; Proteobacteria; Alphaproteobacteria; Rhodobacterales; Rhodobacteraceae; Sulfitobacter
NC_027299	RefSeq	RVG	G963	42,092	58.5	Sulfitobacter phage NYA-2014a	Viruses; dsDNA viruses, no RNA stage; unclassified dsDNA phages.	1526550	Bacteria; Proteobacteria; Alphaproteobacteria; Rhodobacterales; Rhodobacteraceae; Sulfitobacter
TARA_ERS488673_N000365	TOV	EVG	G964	39,890	58.5	-	-	-	-
TARA_ERS490953_N000119	TOV	EVG	G964	39,890	58.5	-	-	-	-
TARA_ERS489084_N000065	TOV	EVG	G965	55,765	58.4	-	-	-	-
TARA_ERS490320_N000035	TOV	EVG	G965	57,172	58.4	-	-	-	-
AB757801	EBI	RVG	G966	41,684	48.3	Edwardsiella phage IW-1	Viruses; dsDNA viruses, no RNA stage; Caudovirales; Podoviridae.	1244857	-
NC_019420	RefSeq	RVG	G966	41,549	48.3	Edwardsiella phage KF-1	Viruses; dsDNA viruses, no RNA stage; Caudovirales; Podoviridae.	1244856	Bacteria; Proteobacteria; Gammaproteobacteria; Enterobacteriales; Enterobacteriaceae; Edwardsiella
NC_021776	RefSeq	RVG	G966	42,313	44.7	Vibrio phage VPMS1	Viruses; dsDNA viruses, no RNA stage; Caudovirales; Podoviridae.	1233488	Bacteria; Proteobacteria; Gammaproteobacteria; Vibrionales; Vibrionaceae; Vibrio; Vibrio harveyi group
AB746912	EBI	RVG	G967	37,159	40.6	Nitratiruptor phage NrS-1	Viruses; dsDNA viruses, no RNA stage; Caudovirales; Siphoviridae.	1230469	-
NC_019525	RefSeq	RVG	G968	45,354	44.3	Bdellovibrio phage phi1422	Viruses; dsDNA viruses, no RNA stage; Caudovirales; Myoviridae.	1127515	Bacteria; Proteobacteria; delta/epsilon subdivisions; Deltaproteobacteria; Bdellovibrionales; Bdellovibrionaceae; Bdellovibrio
NC_015721	RefSeq	RVG	G969	23,931	50.3	Bdellovibrio phage phi1402	Viruses; dsDNA viruses, no RNA stage; Caudovirales; Myoviridae.	1035662	Bacteria; Proteobacteria; delta/epsilon subdivisions; Deltaproteobacteria; Bdellovibrionales; Bdellovibrionaceae; Bdellovibrio
JN859129	EBI	RVG	G970	55,363	61.8	Mycobacterium phage DotProduct	Viruses; dsDNA viruses, no RNA stage; Caudovirales; Siphoviridae; Che8likevirus.	1097752	-
NC_022056	RefSeq	RVG	G970	57,155	61.8	Mycobacterium phage Hamulus	Viruses; dsDNA viruses, no RNA stage; Caudovirales; Siphoviridae.	1340824	Bacteria; Actinobacteria; Actinobacteria; Corynebacteriales; Mycobacteriaceae; Mycobacterium
NC_009820	RefSeq	RVG	G970	58,692	61.7	Mycobacterium phage Tweety	Viruses; dsDNA viruses, no RNA stage; Caudovirales; Siphoviridae; Che8likevirus.	439809	Bacteria; Actinobacteria; Actinobacteria; Corynebacteriales; Mycobacteriaceae; Mycobacterium
NC_021301	RefSeq	RVG	G970	56,279	61.5	Mycobacterium phage SiSi	Viruses; dsDNA viruses, no RNA stage; Caudovirales; Siphoviridae.	1327777	Bacteria; Actinobacteria; Actinobacteria; Corynebacteriales; Mycobacteriaceae; Mycobacterium
NC_023746	RefSeq	RVG	G970	57,387	61.4	Mycobacterium phage GUmbie	Viruses; dsDNA viruses, no RNA stage; Caudovirales; Siphoviridae; Che8likevirus.	1071505	Bacteria; Actinobacteria; Actinobacteria; Corynebacteriales; Mycobacteriaceae; Mycobacterium
NC_014901	RefSeq	RVG	G970	59,230	61.8	Mycobacterium phage Wee	Viruses; dsDNA viruses, no RNA stage; Caudovirales; Siphoviridae; Che8likevirus.	938131	Bacteria; Actinobacteria; Actinobacteria; Corynebacteriales; Mycobacteriaceae; Mycobacterium
NC_025433	RefSeq	RVG	G970	58,550	61.9	Mycobacterium phage Taj	Viruses; dsDNA viruses, no RNA stage; Caudovirales; Siphoviridae; Che8likevirus.	1204515	Bacteria; Actinobacteria; Actinobacteria; Corynebacteriales; Mycobacteriaceae; Mycobacterium
NC_023728	RefSeq	RVG	G970	56,461	61.6	Mycobacterium phage DeadP	Viruses; dsDNA viruses, no RNA stage; Caudovirales; Siphoviridae; Che8likevirus.	1089120	Bacteria; Actinobacteria; Actinobacteria; Corynebacteriales; Mycobacteriaceae; Mycobacterium
NC_013936	RefSeq	RVG	G970	52,141	61.5	Mycobacterium phage Ardmore	Viruses; dsDNA viruses, no RNA stage; Caudovirales; Siphoviridae; Che8likevirus.	685506	Bacteria; Actinobacteria; Actinobacteria; Corynebacteriales; Mycobacteriaceae; Mycobacterium
NC_011288	RefSeq	RVG	G970	58,471	61.8	Mycobacterium phage Fruitloop	Viruses; dsDNA viruses, no RNA stage; Caudovirales; Siphoviridae; Che8likevirus.	563119	Bacteria; Actinobacteria; Actinobacteria; Corynebacteriales; Mycobacteriaceae; Mycobacterium
JF937093	EBI	RVG	G970	58,899	61.9	Mycobacterium phage DLane	Viruses; dsDNA viruses, no RNA stage; Caudovirales; Siphoviridae; Che8likevirus.	1034101	-
NC_028910	RefSeq	RVG	G970	55,617	61.7	Mycobacterium phage XFactor	Viruses; dsDNA viruses, no RNA stage; Caudovirales; Siphoviridae.	1698715	-
NC_028842	RefSeq	RVG	G970	57,328	61.5	Mycobacterium phage CaptainTrips	Viruses; dsDNA viruses, no RNA stage; Caudovirales; Siphoviridae.	1556289	-
NC_028937	RefSeq	RVG	G970	58,338	62	Mycobacterium phage Ovechkin	Viruses; dsDNA viruses, no RNA stage; Caudovirales; Siphoviridae.	1673889	-
NC_028961	RefSeq	RVG	G970	55,094	61.6	Mycobacterium phage PopTart	Viruses; dsDNA viruses, no RNA stage; Caudovirales; Siphoviridae.	1698712	-
NC_023699	RefSeq	RVG	G970	59,016	61.9	Mycobacterium phage SG4	Viruses; dsDNA viruses, no RNA stage; Caudovirales; Siphoviridae; Che8likevirus.	1089132	Bacteria; Actinobacteria; Actinobacteria; Corynebacteriales; Mycobacteriaceae; Mycobacterium
JQ300538	EBI	RVG	G970	61,164	61.7	Mycobacterium phage Spartacus	Viruses; dsDNA viruses, no RNA stage; Caudovirales; Siphoviridae; Che8likevirus.	1147141	-
JN020142	EBI	RVG	G970	57,701	61.3	Mycobacterium phage Mutaforma13	Viruses; dsDNA viruses, no RNA stage; Caudovirales; Siphoviridae; Che8likevirus.	1036614	-
NC_026595	RefSeq	RVG	G970	54,986	61.4	Mycobacterium phage Hades	Viruses; dsDNA viruses, no RNA stage; Caudovirales; Siphoviridae.	1527511	Bacteria; Actinobacteria; Actinobacteria; Corynebacteriales; Mycobacteriaceae; Mycobacterium
NC_008196	RefSeq	RVG	G970	56,852	61.5	Mycobacterium phage Llij	Viruses; dsDNA viruses, no RNA stage; Caudovirales; Siphoviridae; Che8likevirus.	373408	Bacteria; Actinobacteria; Actinobacteria; Corynebacteriales; Mycobacteriaceae; Mycobacterium
NC_028844	RefSeq	RVG	G970	57,293	61.3	Mycobacterium phage Phatniss	Viruses; dsDNA viruses, no RNA stage; Caudovirales; Siphoviridae.	1698356	-
NC_008205	RefSeq	RVG	G970	56,692	61.4	Mycobacterium phage PMC	Viruses; dsDNA viruses, no RNA stage; Caudovirales; Siphoviridae; Che8likevirus.	373412	Bacteria; Actinobacteria; Actinobacteria; Corynebacteriales; Mycobacteriaceae; Mycobacterium
NC_028654	RefSeq	RVG	G970	56,275	61.2	Mycobacterium phage Sparkdehlily	Viruses; dsDNA viruses, no RNA stage; Caudovirales; Siphoviridae.	1739966	-
JF937098	EBI	RVG	G970	55,600	61.2	Mycobacterium phage Ibhubesi	Viruses; dsDNA viruses, no RNA stage; Caudovirales; Siphoviridae; Che8likevirus.	1034107	-
JX411620	EBI	RVG	G970	58,866	61.4	Mycobacterium phage Dorothy	Viruses; dsDNA viruses, no RNA stage; Caudovirales; Siphoviridae; Che8likevirus.	1225861	-
NC_026596	RefSeq	RVG	G970	57,052	61.4	Mycobacterium phage Inventum	Viruses; dsDNA viruses, no RNA stage; Caudovirales; Siphoviridae.	1527580	Bacteria; Actinobacteria; Actinobacteria; Corynebacteriales; Mycobacteriaceae; Mycobacterium
NC_022068	RefSeq	RVG	G970	58,043	61.6	Mycobacterium phage Daenerys	Viruses; dsDNA viruses, no RNA stage; Caudovirales; Siphoviridae.	1340822	Bacteria; Actinobacteria; Actinobacteria; Corynebacteriales; Mycobacteriaceae; Mycobacterium
NC_011287	RefSeq	RVG	G970	58,554	61.3	Mycobacterium phage Pacc40	Viruses; dsDNA viruses, no RNA stage; Caudovirales; Siphoviridae; Che8likevirus.	563122	Bacteria; Actinobacteria; Actinobacteria; Corynebacteriales; Mycobacteriaceae; Mycobacterium
NC_028946	RefSeq	RVG	G970	59,652	61.8	Mycobacterium phage Dante	Viruses; dsDNA viruses, no RNA stage; Caudovirales; Siphoviridae.	1698357	-
JN020143	EBI	RVG	G970	59,794	61.4	Mycobacterium phage ShiLan	Viruses; dsDNA viruses, no RNA stage; Caudovirales; Siphoviridae; Che8likevirus.	1036616	-
NC_028678	RefSeq	RVG	G970	56,669	61.2	Mycobacterium phage Cabrinians	Viruses; dsDNA viruses, no RNA stage; Caudovirales; Siphoviridae.	1739967	-

NC_023580	RefSeq	RVG	G970	57,775	61.3	Mycobacterium phage Saal	Viruses; dsDNA viruses, no RNA stage; Caudovirales; Siphoviridae.	1445728	Bacteria; Actinobacteria; Actinobacteria; Corynebacteriales; Mycobacteriaceae; Mycobacterium
NC_004680	RefSeq	RVG	G970	59,471	61.3	Mycobacterium phage Che8	Viruses; dsDNA viruses, no RNA stage; Caudovirales; Siphoviridae; Che8likevirus.	205868	Bacteria; Actinobacteria; Actinobacteria; Corynebacteriales; Mycobacteriaceae; Mycobacterium
NC_028923	RefSeq	RVG	G970	58,472	61.1	Mycobacterium phage Llama	Viruses; dsDNA viruses, no RNA stage; Caudovirales; Siphoviridae.	1541823	-
NC_026585	RefSeq	RVG	G970	60,727	61.3	Mycobacterium phage Estave1	Viruses; dsDNA viruses, no RNA stage; Caudovirales; Siphoviridae.	1536603	Bacteria; Actinobacteria; Actinobacteria; Corynebacteriales; Mycobacteriaceae; Mycobacterium
JN020141	EBI	RVG	G970	59,314	61.7	Mycobacterium phage Shauna1	Viruses; dsDNA viruses, no RNA stage; Caudovirales; Siphoviridae; Che8likevirus.	1036615	-
NC_028823	RefSeq	RVG	G970	58,985	61.4	Mycobacterium phage Bipolar	Viruses; dsDNA viruses, no RNA stage; Caudovirales; Siphoviridae.	1551711	-
NC_028792	RefSeq	RVG	G970	56,826	61.4	Mycobacterium phage Kimberlium	Viruses; dsDNA viruses, no RNA stage; Caudovirales; Siphoviridae.	1662284	-
NC_023721	RefSeq	RVG	G970	54,411	61.2	Mycobacterium phage Drago	Viruses; dsDNA viruses, no RNA stage; Caudovirales; Siphoviridae; Che8likevirus.	1074303	Bacteria; Actinobacteria; Actinobacteria; Corynebacteriales; Mycobacteriaceae; Mycobacterium
NC_011054	RefSeq	RVG	G970	58,037	61.1	Mycobacterium phage Boomer	Viruses; dsDNA viruses, no RNA stage; Caudovirales; Siphoviridae; Che8likevirus.	546182	Bacteria; Actinobacteria; Actinobacteria; Corynebacteriales; Mycobacteriaceae; Mycobacterium
NC_021538	RefSeq	RVG	G970	59,626	61.2	Mycobacterium phage Job42	Viruses; dsDNA viruses, no RNA stage; Caudovirales; Siphoviridae.	1327771	Bacteria; Actinobacteria; Actinobacteria; Corynebacteriales; Mycobacteriaceae; Mycobacterium
NC_026593	RefSeq	RVG	G970	59,419	61.1	Mycobacterium phage BuzzLyseyear	Viruses; dsDNA viruses, no RNA stage; Caudovirales; Siphoviridae.	1536598	Bacteria; Actinobacteria; Actinobacteria; Corynebacteriales; Mycobacteriaceae; Mycobacterium
NC_011289	RefSeq	RVG	G970	58,578	61.2	Mycobacterium phage Ramsey	Viruses; dsDNA viruses, no RNA stage; Caudovirales; Siphoviridae; Che8likevirus.	563123	Bacteria; Actinobacteria; Actinobacteria; Corynebacteriales; Mycobacteriaceae; Mycobacterium
NC_028813	RefSeq	RVG	G970	57,766	61.8	Mycobacterium phage Seagreen	Viruses; dsDNA viruses, no RNA stage; Caudovirales; Siphoviridae.	1698713	-
NC_028889	RefSeq	RVG	G970	59,416	61.7	Mycobacterium phage Florinda	Viruses; dsDNA viruses, no RNA stage; Caudovirales; Siphoviridae.	1675549	-
NC_028751	RefSeq	RVG	G970	58,671	61.7	Mycobacterium phage Quico	Viruses; dsDNA viruses, no RNA stage; Caudovirales; Siphoviridae.	1675555	-
JF937102	EBI	RVG	G970	57,278	61.1	Mycobacterium phage Mozy	Viruses; dsDNA viruses, no RNA stage; Caudovirales; Siphoviridae; Che8likevirus.	1034112	-
NC_022055	RefSeq	RVG	G970	59,179	61.7	Mycobacterium phage Bobi	Viruses; dsDNA viruses, no RNA stage; Caudovirales; Siphoviridae.	1340708	Bacteria; Actinobacteria; Actinobacteria; Corynebacteriales; Mycobacteriaceae; Mycobacterium
NC_021334	RefSeq	RVG	G970	53,359	61	Mycobacterium phage WIVsmall	Viruses; dsDNA viruses, no RNA stage; Caudovirales; Siphoviridae.	1327036	Bacteria; Actinobacteria; Actinobacteria; Corynebacteriales; Mycobacteriaceae; Mycobacterium
NC_022060	RefSeq	RVG	G970	54,314	61.5	Mycobacterium phage Velveteen	Viruses; dsDNA viruses, no RNA stage; Caudovirales; Siphoviridae.	1340821	Bacteria; Actinobacteria; Actinobacteria; Corynebacteriales; Mycobacteriaceae; Mycobacterium
NC_026604	RefSeq	RVG	G970	53,636	61.5	Mycobacterium phage Cerasum	Viruses; dsDNA viruses, no RNA stage; Caudovirales; Siphoviridae.	1527463	Bacteria; Actinobacteria; Actinobacteria; Corynebacteriales; Mycobacteriaceae; Mycobacterium
NC_026588	RefSeq	RVG	G970	60,285	62.4	Mycobacterium phage Squirty	Viruses; dsDNA viruses, no RNA stage; Caudovirales; Siphoviridae.	1527512	Bacteria; Actinobacteria; Actinobacteria; Corynebacteriales; Mycobacteriaceae; Mycobacterium
NC_022069	RefSeq	RVG	G970	55,213	61.1	Mycobacterium phage Jabbawokkie	Viruses; dsDNA viruses, no RNA stage; Caudovirales; Siphoviridae.	1340820	Bacteria; Actinobacteria; Actinobacteria; Corynebacteriales; Mycobacteriaceae; Mycobacterium
NC_023698	RefSeq	RVG	G970	54,470	61	Mycobacterium phage Avani	Viruses; dsDNA viruses, no RNA stage; Caudovirales; Siphoviridae; Che8likevirus.	1168594	Bacteria; Actinobacteria; Actinobacteria; Corynebacteriales; Mycobacteriaceae; Mycobacterium
NC_004686	RefSeq	RVG	G970	56,276	60.9	Mycobacterium phage Che9d	Viruses; dsDNA viruses, no RNA stage; Caudovirales; Siphoviridae; Che8likevirus.	205876	Bacteria; Actinobacteria; Actinobacteria; Corynebacteriales; Mycobacteriaceae; Mycobacterium
KJ829260	EBI	RVG	G971	70,214	65.3	Mycobacterium phage YungJamal	Viruses; dsDNA viruses, no RNA stage; Caudovirales; Siphoviridae.	1505226	-
NC_004685	RefSeq	RVG	G971	69,777	65.4	Mycobacterium phage Corndog	Viruses; dsDNA viruses, no RNA stage; Caudovirales; Siphoviridae; Corndoglikevirus.	205875	Bacteria; Actinobacteria; Actinobacteria; Corynebacteriales; Mycobacteriaceae; Mycobacterium
NC_022325	RefSeq	RVG	G971	69,815	65.4	Mycobacterium phage Dylan	Viruses; dsDNA viruses, no RNA stage; Caudovirales; Siphoviridae.	1340831	Bacteria; Actinobacteria; Actinobacteria; Corynebacteriales; Mycobacteriaceae; Mycobacterium
NC_023712	RefSeq	RVG	G971	71,341	65.5	Mycobacterium phage Firecracker	Viruses; dsDNA viruses, no RNA stage; Caudovirales; Siphoviridae; Corndoglikevirus.	1089135	Bacteria; Actinobacteria; Actinobacteria; Corynebacteriales; Mycobacteriaceae; Mycobacterium
NC_022057	RefSeq	RVG	G971	72,108	65.4	Mycobacterium phage Catdawg	Viruses; dsDNA viruses, no RNA stage; Caudovirales; Siphoviridae.	1340819	Bacteria; Actinobacteria; Actinobacteria; Corynebacteriales; Mycobacteriaceae; Mycobacterium
JN572061	EBI	RVG	G972	45,580	67.3	Mycobacterium phage Jebeks	Viruses; dsDNA viruses, no RNA stage; Caudovirales; Siphoviridae; Bignuzlikevirus.	1076631	-
NC_026605	RefSeq	RVG	G972	46,869	67.1	Mycobacterium phage Malithi	Viruses; dsDNA viruses, no RNA stage; Caudovirales; Siphoviridae.	1567472	Bacteria; Actinobacteria; Actinobacteria; Corynebacteriales; Mycobacteriaceae; Mycobacterium
NC_028747	RefSeq	RVG	G972	47,618	67	Mycobacterium phage Brusacoram	Viruses; dsDNA viruses, no RNA stage; Caudovirales; Siphoviridae.	1698358	-
NC_021302	RefSeq	RVG	G972	47,109	67.3	Mycobacterium phage Fishburne	Viruses; dsDNA viruses, no RNA stage; Caudovirales; Siphoviridae.	1327765	Bacteria; Actinobacteria; Actinobacteria; Corynebacteriales; Mycobacteriaceae; Mycobacterium
NC_023552	RefSeq	RVG	G972	47,162	67.2	Mycobacterium phage Donovan	Viruses; dsDNA viruses, no RNA stage; Caudovirales; Siphoviridae.	1429793	Bacteria; Actinobacteria; Actinobacteria; Corynebacteriales; Mycobacteriaceae; Mycobacterium
NC_028796	RefSeq	RVG	G972	49,203	66.7	Mycobacterium phage Phayonce	Viruses; dsDNA viruses, no RNA stage; Caudovirales; Siphoviridae.	1647302	-
NC_023692	RefSeq	RVG	G972	48,984	66.7	Mycobacterium phage BigNuz	Viruses; dsDNA viruses, no RNA stage; Caudovirales; Siphoviridae; Bignuzlikevirus.	1074309	Bacteria; Actinobacteria; Actinobacteria; Corynebacteriales; Mycobacteriaceae; Mycobacterium
HM152765	EBI	RVG	G972	47,287	66.8	Mycobacterium phage island3	Viruses; dsDNA viruses, no RNA stage; Caudovirales; Siphoviridae; Che9clikevirus.	861048	-
NC_011291	RefSeq	RVG	G972	47,057	66.8	Mycobacterium phage Brujita	Viruses; dsDNA viruses, no RNA stage; Caudovirales; Siphoviridae; Che9clikevirus.	561996	Bacteria; Actinobacteria; Actinobacteria; Corynebacteriales; Mycobacteriaceae; Mycobacterium
NC_023697	RefSeq	RVG	G972	48,420	67.1	Mycobacterium phage Babsiella	Viruses; dsDNA viruses, no RNA stage; Caudovirales; Siphoviridae; Che9clikevirus.	1089115	Bacteria; Actinobacteria; Actinobacteria; Corynebacteriales; Mycobacteriaceae; Mycobacterium
NC_004683	RefSeq	RVG	G972	57,050	65.4	Mycobacterium phage Che9c	Viruses; dsDNA viruses, no RNA stage; Caudovirales; Siphoviridae; Che9clikevirus.	205872	Bacteria; Actinobacteria; Actinobacteria; Corynebacteriales; Mycobacteriaceae; Mycobacterium
NC_026589	RefSeq	RVG	G972	55,832	65.6	Mycobacterium phage Sbash	Viruses; dsDNA viruses, no RNA stage; Caudovirales; Siphoviridae.	1567475	Bacteria; Actinobacteria; Actinobacteria; Corynebacteriales; Mycobacteriaceae; Mycobacterium

NC_021061	RefSeq	RVG	G972	41,491	65.8	Mycobacterium phage Butters	Viruses; dsDNA viruses, no RNA stage; Caudovirales; Siphoviridae.	1296646	Bacteria; Actinobacteria; Actinobacteria; Corynebacteriales; Mycobacteriaceae; Mycobacterium
NC_023572	RefSeq	RVG	G972	42,240	66	Mycobacterium phage MichelleMyBell	Viruses; dsDNA viruses, no RNA stage; Caudovirales; Siphoviridae.	1445726	Bacteria; Actinobacteria; Actinobacteria; Corynebacteriales; Mycobacteriaceae; Mycobacterium
NC_023730	RefSeq	RVG	G972	42,594	66.1	Mycobacterium phage Redi	Viruses; dsDNA viruses, no RNA stage; Caudovirales; Siphoviridae; Charlielikevirus.	1079894	Bacteria; Actinobacteria; Actinobacteria; Corynebacteriales; Mycobacteriaceae; Mycobacterium
NC_028779	RefSeq	RVG	G972	43,680	66.2	Mycobacterium phage Carcharodon	Viruses; dsDNA viruses, no RNA stage; Caudovirales; Siphoviridae.	1555233	-
NC_023729	RefSeq	RVG	G972	43,036	66.3	Mycobacterium phage Charlie	Viruses; dsDNA viruses, no RNA stage; Caudovirales; Siphoviridae; Charlielikevirus.	1056830	Bacteria; Actinobacteria; Actinobacteria; Corynebacteriales; Mycobacteriaceae; Mycobacterium
NC_021309	RefSeq	RVG	G973	74,711	63.1	Mycobacterium phage Phrux	Viruses; dsDNA viruses, no RNA stage; Caudovirales; Siphoviridae.	1327774	Bacteria; Actinobacteria; Actinobacteria; Corynebacteriales; Mycobacteriaceae; Mycobacterium
NC_022330	RefSeq	RVG	G973	76,586	63	Mycobacterium phage Quink	Viruses; dsDNA viruses, no RNA stage; Caudovirales; Siphoviridae.	1354514	Bacteria; Actinobacteria; Actinobacteria; Corynebacteriales; Mycobacteriaceae; Mycobacterium
JN391441	EBI	RVG	G973	74,675	63	Mycobacterium phage Elph10	Viruses; dsDNA viruses, no RNA stage; Caudovirales; Siphoviridae.	1070989	-
NC_004681	RefSeq	RVG	G973	75,931	63.1	Mycobacterium phage Cjw1	Viruses; dsDNA viruses, no RNA stage; Caudovirales; Siphoviridae; Cjwunalikevirus.	205869	Bacteria; Actinobacteria; Actinobacteria; Corynebacteriales; Mycobacteriaceae; Mycobacterium
GQ303265	EBI	RVG	G973	74,491	63	Mycobacterium phage Pumpkin	Viruses; dsDNA viruses, no RNA stage; Caudovirales; Siphoviridae; Cjwunalikevirus.	663559	-
NC_021305	RefSeq	RVG	G973	76,179	62.9	Mycobacterium phage Murphy	Viruses; dsDNA viruses, no RNA stage; Caudovirales; Siphoviridae.	1327939	Bacteria; Actinobacteria; Actinobacteria; Corynebacteriales; Mycobacteriaceae; Mycobacterium
NC_023689	RefSeq	RVG	G973	76,260	63	Mycobacterium phage Lilac	Viruses; dsDNA viruses, no RNA stage; Caudovirales; Siphoviridae.	1070990	Bacteria; Actinobacteria; Actinobacteria; Corynebacteriales; Mycobacteriaceae; Mycobacterium
NC_021306	RefSeq	RVG	G973	75,736	63	Mycobacterium phage Dumbo	Viruses; dsDNA viruses, no RNA stage; Caudovirales; Siphoviridae.	1327764	Bacteria; Actinobacteria; Actinobacteria; Corynebacteriales; Mycobacteriaceae; Mycobacterium
NC_008194	RefSeq	RVG	G973	74,483	62.9	Mycobacterium phage 244	Viruses; dsDNA viruses, no RNA stage; Caudovirales; Siphoviridae; Cjwunalikevirus.	373403	Bacteria; Actinobacteria; Actinobacteria; Corynebacteriales; Mycobacteriaceae; Mycobacterium
NC_022059	RefSeq	RVG	G973	77,367	63	Mycobacterium phage DrDrey	Viruses; dsDNA viruses, no RNA stage; Caudovirales; Siphoviridae.	1357669	Bacteria; Actinobacteria; Actinobacteria; Corynebacteriales; Mycobacteriaceae; Mycobacterium
KF493883	EBI	RVG	G973	74,533	63.1	Mycobacterium phage Mosby	Viruses; dsDNA viruses, no RNA stage; Caudovirales; Siphoviridae.	1429913	-
NC_022065	RefSeq	RVG	G973	74,533	63.1	Mycobacterium phage Contagion	Viruses; dsDNA viruses, no RNA stage; Caudovirales; Siphoviridae.	1340833	Bacteria; Actinobacteria; Actinobacteria; Corynebacteriales; Mycobacteriaceae; Mycobacterium
NC_022988	RefSeq	RVG	G973	74,210	63	Mycobacterium phage Bruin	Viruses; dsDNA viruses, no RNA stage; Caudovirales; Siphoviridae.	1391179	Bacteria; Actinobacteria; Actinobacteria; Corynebacteriales; Mycobacteriaceae; Mycobacterium
NC_022976	RefSeq	RVG	G973	75,894	63.1	Mycobacterium phage Nala	Viruses; dsDNA viruses, no RNA stage; Caudovirales; Siphoviridae.	1391180	Bacteria; Actinobacteria; Actinobacteria; Corynebacteriales; Mycobacteriaceae; Mycobacterium
NC_021311	RefSeq	RVG	G973	76,479	62.9	Mycobacterium phage Phaux	Viruses; dsDNA viruses, no RNA stage; Caudovirales; Siphoviridae.	1327937	Bacteria; Actinobacteria; Actinobacteria; Corynebacteriales; Mycobacteriaceae; Mycobacterium
NC_022969	RefSeq	RVG	G973	76,217	63	Mycobacterium phage PhatBacter	Viruses; dsDNA viruses, no RNA stage; Caudovirales; Siphoviridae.	1391181	Bacteria; Actinobacteria; Actinobacteria; Corynebacteriales; Mycobacteriaceae; Mycobacterium
NC_022981	RefSeq	RVG	G973	76,323	63.1	Mycobacterium phage HufflyPuff	Viruses; dsDNA viruses, no RNA stage; Caudovirales; Siphoviridae.	1430411	Bacteria; Actinobacteria; Actinobacteria; Corynebacteriales; Mycobacteriaceae; Mycobacterium
NC_011055	RefSeq	RVG	G973	76,312	62.8	Mycobacterium phage Porky	Viruses; dsDNA viruses, no RNA stage; Caudovirales; Siphoviridae; Cjwunalikevirus.	546185	Bacteria; Actinobacteria; Actinobacteria; Corynebacteriales; Mycobacteriaceae; Mycobacterium
JF937096	EBI	RVG	G973	76,049	63	Mycobacterium phage Henry	Viruses; dsDNA viruses, no RNA stage; Caudovirales; Siphoviridae; Cjwunalikevirus.	1034105	-
NC_029093	RefSeq	RVG	G973	75,796	63	Mycobacterium phage Mindy	Viruses; dsDNA viruses, no RNA stage; Caudovirales; Siphoviridae.	1647311	-
NC_011056	RefSeq	RVG	G973	75,811	62.9	Mycobacterium phage Kostya	Viruses; dsDNA viruses, no RNA stage; Caudovirales; Siphoviridae; Cjwunalikevirus.	546183	Bacteria; Actinobacteria; Actinobacteria; Corynebacteriales; Mycobacteriaceae; Mycobacterium
NC_028785	RefSeq	RVG	G973	72,790	63.1	Mycobacterium phage NelitzaMV	Viruses; dsDNA viruses, no RNA stage; Caudovirales; Siphoviridae.	1679530	-
KF188414	EBI	RVG	G973	76,131	63	Mycobacterium phage ABCat	Viruses; dsDNA viruses, no RNA stage; Caudovirales; Siphoviridae; Cjwunalikevirus; unclassified Cjwunalikevirus.	1358124	-
NC_029079	RefSeq	RVG	G973	75,339	63	Mycobacterium phage Dusk	Viruses; dsDNA viruses, no RNA stage; Caudovirales; Siphoviridae; Cjwunalikevirus; unclassified Cjwunalikevirus.	1679524	-
NC_028906	RefSeq	RVG	G973	75,933	63	Mycobacterium phage Toto	Viruses; dsDNA viruses, no RNA stage; Caudovirales; Siphoviridae; Cjwunalikevirus.	1035485	-
JN006062	EBI	RVG	G973	75,706	62.9	Mycobacterium phage Rakim	Viruses; dsDNA viruses, no RNA stage; Caudovirales; Siphoviridae.	1035483	-
JF937106	EBI	RVG	G973	75,973	62.9	Mycobacterium phage SirDuracell	Viruses; dsDNA viruses, no RNA stage; Caudovirales; Siphoviridae; Cjwunalikevirus.	1034116	-
JF937091	EBI	RVG	G973	74,997	62.9	Mycobacterium phage Bask21	Viruses; dsDNA viruses, no RNA stage; Caudovirales; Siphoviridae; Cjwunalikevirus.	1034100	-
JN412590	EBI	RVG	G973	76,174	62.9	Mycobacterium phage Eureka	Viruses; dsDNA viruses, no RNA stage; Caudovirales; Siphoviridae; Cjwunalikevirus.	1074306	-
NC_022085	RefSeq	RVG	G973	76,483	62.8	Mycobacterium phage Goku	Viruses; dsDNA viruses, no RNA stage; Caudovirales; Siphoviridae.	1383057	Bacteria; Actinobacteria; Actinobacteria; Corynebacteriales; Mycobacteriaceae; Mycobacterium
JF957059	EBI	RVG	G974	109,270	60.8	Mycobacterium phage Optimus	Viruses; dsDNA viruses, no RNA stage; Caudovirales; Siphoviridae; Omegalikevirus.	1032893	-
JF937090	EBI	RVG	G974	111,688	60.7	Mycobacterium phage Baka	Viruses; dsDNA viruses, no RNA stage; Caudovirales; Siphoviridae; Omegalikevirus.	1034099	-
NC_022067	RefSeq	RVG	G974	109,960	60.8	Mycobacterium phage Wanda	Viruses; dsDNA viruses, no RNA stage; Caudovirales; Siphoviridae.	1340713	Bacteria; Actinobacteria; Actinobacteria; Corynebacteriales; Mycobacteriaceae; Mycobacterium
NC_026584	RefSeq	RVG	G974	109,871	60.7	Mycobacterium phage Minerva	Viruses; dsDNA viruses, no RNA stage; Caudovirales; Siphoviridae.	1527513	Bacteria; Actinobacteria; Actinobacteria; Corynebacteriales; Mycobacteriaceae; Mycobacterium
NC_022066	RefSeq	RVG	G974	108,297	60.9	Mycobacterium phage Redno2	Viruses; dsDNA viruses, no RNA stage; Caudovirales; Siphoviridae.	1340709	Bacteria; Actinobacteria; Actinobacteria; Corynebacteriales; Mycobacteriaceae; Mycobacterium

NC_023738	RefSeq	RVG	G974	106,327	60.8	Mycobacterium phage Thibault	Viruses; dsDNA viruses, no RNA stage; Caudovirales; Siphoviridae; Omegalikevirus.	1052673	Bacteria; Actinobacteria; Actinobacteria; Corynebacteriales; Mycobacteriaceae; Mycobacterium
NC_028876	RefSeq	RVG	G974	109,800	61	Mycobacterium phage Ariel	Viruses; dsDNA viruses, no RNA stage; Caudovirales; Siphoviridae.	1541824	-
NC_023690	RefSeq	RVG	G974	110,569	60.9	Mycobacterium phage Courthouse	Viruses; dsDNA viruses, no RNA stage; Caudovirales; Siphoviridae; Omegalikevirus.	1089119	Bacteria; Actinobacteria; Actinobacteria; Corynebacteriales; Mycobacteriaceae; Mycobacterium
NC_028953	RefSeq	RVG	G974	110,764	61.2	Mycobacterium phage MiaZeal	Viruses; dsDNA viruses, no RNA stage; Caudovirales; Siphoviridae.	1567005	-
JF937101	EBI	RVG	G974	109,086	61.3	Mycobacterium phage LittleE	Viruses; dsDNA viruses, no RNA stage; Caudovirales; Siphoviridae; Omegalikevirus.	1034111	-
NC_004688	RefSeq	RVG	G974	110,865	61.4	Mycobacterium phage Omega	Viruses; dsDNA viruses, no RNA stage; Caudovirales; Siphoviridae; Omegalikevirus.	205879	Bacteria; Actinobacteria; Actinobacteria; Corynebacteriales; Mycobacteriaceae; Mycobacterium
NC_026590	RefSeq	RVG	G975	90,460	56.8	Mycobacterium phage Gaia	Viruses; dsDNA viruses, no RNA stage; Caudovirales; Siphoviridae.	1486472	Bacteria; Actinobacteria; Actinobacteria; Corynebacteriales; Mycobacteriaceae; Mycobacterium
KJ567045	EBI	RVG	G976	60,310	66.6	Mycobacterium phage Emerson	Viruses; dsDNA viruses, no RNA stage; Caudovirales; Siphoviridae.	1486470	-
NC_023747	RefSeq	RVG	G976	59,672	66.6	Mycobacterium phage BarrelRoll	Viruses; dsDNA viruses, no RNA stage; Caudovirales; Siphoviridae.	1084722	Bacteria; Actinobacteria; Actinobacteria; Corynebacteriales; Mycobacteriaceae; Mycobacterium
JN185608	EBI	RVG	G976	59,749	66.6	Mycobacterium phage jaws	Viruses; dsDNA viruses, no RNA stage; Caudovirales; Siphoviridae; Tm4likevirus.	1051143	-
NC_014458	RefSeq	RVG	G976	59,598	66.4	Mycobacterium phage Angelica	Viruses; dsDNA viruses, no RNA stage; Caudovirales; Siphoviridae; Tm4likevirus.	861044	Bacteria; Actinobacteria; Actinobacteria; Corynebacteriales; Mycobacteriaceae; Mycobacterium
NC_014459	RefSeq	RVG	G976	59,798	66.9	Mycobacterium phage CrimD	Viruses; dsDNA viruses, no RNA stage; Caudovirales; Siphoviridae; Tm4likevirus.	861045	Bacteria; Actinobacteria; Actinobacteria; Corynebacteriales; Mycobacteriaceae; Mycobacterium
NC_028978	RefSeq	RVG	G976	60,609	66.7	Mycobacterium phage Murucutumbu	Viruses; dsDNA viruses, no RNA stage; Caudovirales; Siphoviridae.	1560286	-
NC_028936	RefSeq	RVG	G976	59,052	67.2	Mycobacterium phage Enkosi	Viruses; dsDNA viruses, no RNA stage; Caudovirales; Siphoviridae.	1698709	-
NC_023498	RefSeq	RVG	G976	62,466	68.4	Mycobacterium phage Validus	Viruses; dsDNA viruses, no RNA stage; Caudovirales; Siphoviridae.	1414747	Bacteria; Actinobacteria; Actinobacteria; Corynebacteriales; Mycobacteriaceae; Mycobacterium
JF937104	EBI	RVG	G976	61,147	67.3	Mycobacterium phage Pixie	Viruses; dsDNA viruses, no RNA stage; Caudovirales; Siphoviridae; Tm4likevirus.	1034114	-
NC_028846	RefSeq	RVG	G976	61,081	67.3	Mycobacterium phage ShedlockHolmes	Viruses; dsDNA viruses, no RNA stage; Caudovirales; Siphoviridae.	1647313	-
NC_026603	RefSeq	RVG	G976	61,251	67.3	Mycobacterium phage Keshu	Viruses; dsDNA viruses, no RNA stage; Caudovirales; Siphoviridae.	1567471	Bacteria; Actinobacteria; Actinobacteria; Corynebacteriales; Mycobacteriaceae; Mycobacterium
JX042579	EBI	RVG	G976	61,567	67.3	Mycobacterium phage macncheese	Viruses; dsDNA viruses, no RNA stage; Caudovirales; Siphoviridae; Tm4likevirus.	1195070	-
NC_003387	RefSeq	RVG	G976	52,797	68.1	Mycobacterium phage TM4	Viruses; dsDNA viruses, no RNA stage; Caudovirales; Siphoviridae; Tm4likevirus.	88870	Bacteria; Actinobacteria; Actinobacteria; Corynebacteriales; Mycobacteriaceae; Mycobacterium
NC_028759	RefSeq	RVG	G976	58,065	68.2	Mycobacterium phage Mufasa	Viruses; dsDNA viruses, no RNA stage; Caudovirales; Siphoviridae.	1718600	-
NC_026598	RefSeq	RVG	G976	58,211	68.3	Mycobacterium phage Milly	Viruses; dsDNA viruses, no RNA stage; Caudovirales; Siphoviridae.	1567473	Bacteria; Actinobacteria; Actinobacteria; Corynebacteriales; Mycobacteriaceae; Mycobacterium
NC_024147	RefSeq	RVG	G976	57,315	68.5	Mycobacterium phage ZoeJ	Viruses; dsDNA viruses, no RNA stage; Caudovirales; Siphoviridae.	1486427	Bacteria; Actinobacteria; Actinobacteria; Corynebacteriales; Mycobacteriaceae; Mycobacterium
NC_028979	RefSeq	RVG	G976	57,253	68	Mycobacterium phage Cheetobro	Viruses; dsDNA viruses, no RNA stage; Caudovirales; Siphoviridae.	1506716	-
NC_027365	RefSeq	RVG	G976	58,076	68	Mycobacterium phage fionn	Viruses; dsDNA viruses, no RNA stage; Caudovirales; Siphoviridae; Tm4likevirus.	1097753	Bacteria; Actinobacteria; Actinobacteria; Corynebacteriales; Mycobacteriaceae; Mycobacterium
NC_028947	RefSeq	RVG	G976	62,738	65.7	Mycobacterium phage Kratio	Viruses; dsDNA viruses, no RNA stage; Caudovirales; Siphoviridae.	1606763	-
NC_023724	RefSeq	RVG	G976	62,991	65.3	Mycobacterium phage Larva	Viruses; dsDNA viruses, no RNA stage; Caudovirales; Siphoviridae; Tm4likevirus.	1056831	Bacteria; Actinobacteria; Actinobacteria; Corynebacteriales; Mycobacteriaceae; Mycobacterium
NC_024366	RefSeq	RVG	G976	62,661	64.9	Mycobacterium phage OkiRoe	Viruses; dsDNA viruses, no RNA stage; Caudovirales; Siphoviridae.	1486473	Bacteria; Actinobacteria; Actinobacteria; Corynebacteriales; Mycobacteriaceae; Mycobacterium
NC_028832	RefSeq	RVG	G976	61,511	64	Mycobacterium phage Omnicron	Viruses; dsDNA viruses, no RNA stage; Caudovirales; Siphoviridae.	1541819	-
EU568876	EBI	RVG	G976	41,901	66.6	Mycobacterium phage BPs	Viruses; dsDNA viruses, no RNA stage; Caudovirales; Siphoviridae.	515982	-
GQ303261	EBI	RVG	G976	41,901	66.6	Mycobacterium phage Hope	Viruses; dsDNA viruses, no RNA stage; Caudovirales; Siphoviridae.	663555	-
JN699002	EBI	RVG	G976	41,901	66.6	Mycobacterium phage Avrafan	Viruses; dsDNA viruses, no RNA stage; Caudovirales; Siphoviridae.	1089114	-
KM923970	EBI	RVG	G976	41,903	66.6	Mycobacterium phage Gomashi	Viruses; dsDNA viruses, no RNA stage; Caudovirales; Siphoviridae.	1606762	-
NC_012788	RefSeq	RVG	G976	41,441	66.7	Mycobacterium phage Angel	Viruses; dsDNA viruses, no RNA stage; Caudovirales; Siphoviridae.	649688	Bacteria; Actinobacteria; Actinobacteria; Corynebacteriales; Mycobacteriaceae; Mycobacterium
NC_008202	RefSeq	RVG	G976	42,289	66.7	Mycobacterium phage Halo	Viruses; dsDNA viruses, no RNA stage; Caudovirales; Siphoviridae; Halolikevirus.	373407	Bacteria; Actinobacteria; Actinobacteria
KJ725374	EBI	RVG	G976	40,086	66.8	Mycobacterium phage Guo1	Viruses; dsDNA viruses, no RNA stage; Caudovirales; Siphoviridae.	1498190	-
NC_021556	RefSeq	RVG	G976	39,981	66.9	Mycobacterium phage Leo	Viruses; dsDNA viruses, no RNA stage; Caudovirales; Siphoviridae.	1327952	Bacteria; Actinobacteria; Actinobacteria; Corynebacteriales; Mycobacteriaceae; Mycobacterium; Mycobacterium tuberculosis complex
KC787107	EBI	RVG	G976	39,318	66.8	Mycobacterium phage Bo4	Viruses; dsDNA viruses, no RNA stage; Caudovirales; Siphoviridae.	1327949	-
KC787108	EBI	RVG	G976	39,520	66.8	Mycobacterium phage DNAIII	Viruses; dsDNA viruses, no RNA stage; Caudovirales; Siphoviridae.	1327950	-
NC_023705	RefSeq	RVG	G976	41,650	66.8	Mycobacterium phage Liefie	Viruses; dsDNA viruses, no RNA stage; Caudovirales; Siphoviridae; Halolikevirus.	1074304	Bacteria; Actinobacteria; Actinobacteria; Corynebacteriales; Mycobacteriaceae; Mycobacterium
NC_028922	RefSeq	RVG	G976	45,161	68.8	Mycobacterium phage Cambiare	Viruses; dsDNA viruses, no RNA stage; Caudovirales; Siphoviridae.	1647305	-
NC_028861	RefSeq	RVG	G976	44,576	68.5	Mycobacterium phage FlagStaff	Viruses; dsDNA viruses, no RNA stage; Caudovirales; Siphoviridae.	1647304	-
NC_028791	RefSeq	RVG	G976	44,492	68.6	Mycobacterium phage MOOREtheMARYer	Viruses; dsDNA viruses, no RNA stage; Caudovirales; Siphoviridae.	1647309	-
NC_023604	RefSeq	RVG	G976	44,306	68.1	Mycobacterium phage Jolie2	Viruses; dsDNA viruses, no RNA stage; Caudovirales; Siphoviridae.	1458831	Bacteria; Actinobacteria; Actinobacteria; Corynebacteriales; Mycobacteriaceae; Mycobacterium
KM591905	EBI	RVG	G977	42,596	66.1	Mycobacterium phage RonRayGun	Viruses; dsDNA viruses, no RNA stage; Caudovirales; Siphoviridae.	1555234	-
NC_024135	RefSeq	RVG	G977	42,392	66.2	Mycobacterium phage Bernal13	Viruses; dsDNA viruses, no RNA stage; Caudovirales; Siphoviridae.	1486424	Bacteria; Actinobacteria; Actinobacteria; Corynebacteriales; Mycobacteriaceae; Mycobacterium
NC_026597	RefSeq	RVG	G978	63,334	65.2	Mycobacterium phage Sparky	Viruses; dsDNA viruses, no RNA stage; Caudovirales; Siphoviridae.	1527493	Bacteria; Actinobacteria; Actinobacteria; Corynebacteriales; Mycobacteriaceae; Mycobacterium

NC_023744	RefSeq	RVG	G979	60,588	68.4	Mycobacterium phage DS6A	Viruses; dsDNA viruses, no RNA stage; Caudovirales; Siphoviridae.	45764	Bacteria; Actinobacteria; Actinobacteria; Corynebacteriales; Mycobacteriaceae; Mycobacterium; Mycobacterium tuberculosis complex
NC_022054	RefSeq	RVG	G980	48,228	58.8	Mycobacterium phage Muddy	Viruses; dsDNA viruses, no RNA stage; Caudovirales; Siphoviridae.	1340829	Bacteria; Actinobacteria; Actinobacteria; Corynebacteriales; Mycobacteriaceae; Mycobacterium
NC_021063	RefSeq	RVG	G980	47,724	58.6	Mycobacterium phage vB_MapS_FF47	Viruses; dsDNA viruses, no RNA stage; Caudovirales; Siphoviridae.	1305710	Bacteria; Actinobacteria; Actinobacteria; Corynebacteriales; Mycobacteriaceae; Mycobacterium; Mycobacterium avium complex (MAC); Mycobacterium avium
NC_015720	RefSeq	RVG	G980	45,530	60.3	Gordonia phage GTE2	Viruses; dsDNA viruses, no RNA stage; Caudovirales; Siphoviridae.	981323	Bacteria; Actinobacteria; Actinobacteria; Corynebacteriales; Nocardiaceae; Nocardia
KJ538722	EBI	RVG	G981	53,746	67.4	Mycobacterium phage HH92	Viruses; dsDNA viruses, no RNA stage; Caudovirales; Siphoviridae.	1471543	-
NC_009993	RefSeq	RVG	G981	53,746	67.4	Mycobacterium phage Giles	Viruses; dsDNA viruses, no RNA stage; Caudovirales; Siphoviridae.	480808	Bacteria; Actinobacteria; Actinobacteria; Corynebacteriales; Mycobacteriaceae; Mycobacterium
NC_023602	RefSeq	RVG	G981	50,781	65.7	Mycobacterium phage 32HC	Viruses; dsDNA viruses, no RNA stage; Caudovirales; Siphoviridae.	1445729	Bacteria; Actinobacteria; Actinobacteria; Corynebacteriales; Mycobacteriaceae; Mycobacterium
NC_016652	RefSeq	RVG	G982	49,330	65.4	Rhodococcus phage REQ2	Viruses; unclassified phages.	1109713	Bacteria; Actinobacteria; Actinobacteria; Corynebacteriales; Nocardiaceae; Rhodococcus
NC_009603	RefSeq	RVG	G983	46,365	68.3	Microbacterium phage Min1	Viruses; dsDNA viruses, no RNA stage; Caudovirales; Siphoviridae.	446529	Bacteria; Actinobacteria; Actinobacteria; Micrococcales; Microbacteriaceae; Microbacterium
NC_022972	RefSeq	RVG	G984	37,372	62.7	Arthrobacter phage vB_ArS-ArV2	Viruses; dsDNA viruses, no RNA stage; Caudovirales; Siphoviridae.	1414742	Bacteria; Actinobacteria; Actinobacteria; Micrococcales; Micrococcaceae; Arthrobacter
GU247134	EBI	RVG	G985	68,432	66.5	Mycobacterium phage Scoot17C	Viruses; dsDNA viruses, no RNA stage; Caudovirales; Siphoviridae.	701455	-
NC_008197	RefSeq	RVG	G985	68,427	66.5	Mycobacterium phage Orion	Viruses; dsDNA viruses, no RNA stage; Caudovirales; Siphoviridae.	373409	Bacteria; Actinobacteria; Actinobacteria; Corynebacteriales; Mycobacteriaceae; Mycobacterium
FJ174694	EBI	RVG	G985	68,450	66.5	Mycobacterium phage Chah	Viruses; dsDNA viruses, no RNA stage; Caudovirales; Siphoviridae.	563124	-
JN699009	EBI	RVG	G985	68,992	66.5	Mycobacterium phage ThreeOh3D2	Viruses; dsDNA viruses, no RNA stage; Caudovirales; Siphoviridae.	1089133	-
NC_028682	RefSeq	RVG	G985	69,030	66.5	Mycobacterium phage Badfish	Viruses; dsDNA viruses, no RNA stage; Caudovirales; Siphoviridae.	1458710	-
KP027209	EBI	RVG	G985	68,311	66.5	Mycobacterium phage Sigman	Viruses; dsDNA viruses, no RNA stage; Caudovirales; Siphoviridae.	1567476	-
NC_023740	RefSeq	RVG	G985	68,311	66.5	Mycobacterium phage JacAttac	Viruses; dsDNA viruses, no RNA stage; Caudovirales; Siphoviridae.	1089136	Bacteria; Actinobacteria; Actinobacteria; Corynebacteriales; Mycobacteriaceae; Mycobacterium
JN006063	EBI	RVG	G985	68,804	66.5	Mycobacterium phage Serendipity	Viruses; dsDNA viruses, no RNA stage; Caudovirales; Siphoviridae.	1035484	-
JN698990	EBI	RVG	G985	68,839	66.5	Mycobacterium phage IsaacEli	Viruses; dsDNA viruses, no RNA stage; Caudovirales; Siphoviridae.	1089124	-
NC_028907	RefSeq	RVG	G985	68,839	66.5	Mycobacterium phage Kikipoo	Viruses; dsDNA viruses, no RNA stage; Caudovirales; Siphoviridae.	1089127	-
JF937097	EBI	RVG	G985	68,675	66.4	Mycobacterium phage Hertubise	Viruses; dsDNA viruses, no RNA stage; Caudovirales; Siphoviridae.	1034106	-
JX649097	EBI	RVG	G985	68,992	66.5	Mycobacterium phage Piglet	Viruses; dsDNA viruses, no RNA stage; Caudovirales; Siphoviridae.	1235693	-
JX649100	EBI	RVG	G985	68,910	66.5	Mycobacterium phage Alex	Viruses; dsDNA viruses, no RNA stage; Caudovirales; Siphoviridae.	1235690	-
JX649096	EBI	RVG	G985	68,884	66.5	Mycobacterium phage Serpentine	Viruses; dsDNA viruses, no RNA stage; Caudovirales; Siphoviridae.	1235694	-
KJ194583	EBI	RVG	G985	68,607	66.5	Mycobacterium phage Numberten	Viruses; dsDNA viruses, no RNA stage; Caudovirales; Siphoviridae.	1458713	-
NC_028674	RefSeq	RVG	G985	68,355	66.5	Mycobacterium phage ShiVal	Viruses; dsDNA viruses, no RNA stage; Caudovirales; Siphoviridae.	1296647	-
KJ567044	EBI	RVG	G985	68,428	66.5	Mycobacterium phage EmpTee	Viruses; dsDNA viruses, no RNA stage; Caudovirales; Siphoviridae.	1486471	-
NC_028689	RefSeq	RVG	G985	67,774	66.5	Mycobacterium phage Colbert	Viruses; dsDNA viruses, no RNA stage; Caudovirales; Siphoviridae.	663553	-
JX649098	EBI	RVG	G985	69,321	66.6	Mycobacterium phage Nacho	Viruses; dsDNA viruses, no RNA stage; Caudovirales; Siphoviridae.	1235692	-
NC_023563	RefSeq	RVG	G985	68,262	66.6	Mycobacterium phage Suffolk	Viruses; dsDNA viruses, no RNA stage; Caudovirales; Siphoviridae.	1414748	Bacteria; Actinobacteria; Actinobacteria; Corynebacteriales; Mycobacteriaceae; Mycobacterium
NC_021310	RefSeq	RVG	G985	68,598	66.5	Mycobacterium phage Newman	Viruses; dsDNA viruses, no RNA stage; Caudovirales; Siphoviridae.	1327766	Bacteria; Actinobacteria; Actinobacteria; Corynebacteriales; Mycobacteriaceae; Mycobacterium
NC_028690	RefSeq	RVG	G985	68,472	66.5	Mycobacterium phage Eremos	Viruses; dsDNA viruses, no RNA stage; Caudovirales; Siphoviridae.	1527494	-
NC_028681	RefSeq	RVG	G985	68,367	66.6	Mycobacterium phage Pops	Viruses; dsDNA viruses, no RNA stage; Caudovirales; Siphoviridae.	1675554	-
NC_028691	RefSeq	RVG	G985	68,227	66.4	Mycobacterium phage Apizium	Viruses; dsDNA viruses, no RNA stage; Caudovirales; Siphoviridae.	1673886	-
NC_024363	RefSeq	RVG	G985	68,807	66.4	Mycobacterium phage Manad	Viruses; dsDNA viruses, no RNA stage; Caudovirales; Siphoviridae.	1486403	Bacteria; Actinobacteria; Actinobacteria; Corynebacteriales; Mycobacteriaceae; Mycobacterium
KP027208	EBI	RVG	G985	68,328	66.5	Mycobacterium phage Pipsqueak	Viruses; dsDNA viruses, no RNA stage; Caudovirales; Siphoviridae.	1567474	-
NC_023727	RefSeq	RVG	G985	68,494	66.5	Mycobacterium phage Vista	Viruses; dsDNA viruses, no RNA stage; Caudovirales; Siphoviridae.	1089108	Bacteria; Actinobacteria; Actinobacteria; Corynebacteriales; Mycobacteriaceae; Mycobacterium
NC_027985	RefSeq	RVG	G985	68,016	66.5	Mycobacterium phage UncleHowie	Viruses; dsDNA viruses, no RNA stage; Caudovirales; Siphoviridae.	663560	Bacteria; Actinobacteria; Actinobacteria; Corynebacteriales; Mycobacteriaceae; Mycobacterium
JN638752	EBI	RVG	G985	68,600	66.4	Mycobacterium phage Murdoc	Viruses; dsDNA viruses, no RNA stage; Caudovirales; Siphoviridae.	1084720	-
JF957056	EBI	RVG	G985	68,839	66.5	Mycobacterium phage Thora	Viruses; dsDNA viruses, no RNA stage; Caudovirales; Siphoviridae.	1032894	-
KM408320	EBI	RVG	G985	68,576	66.4	Mycobacterium phage Lasso	Viruses; dsDNA viruses, no RNA stage; Caudovirales; Siphoviridae.	1541886	-
GQ303264	EBI	RVG	G985	68,323	66.4	Mycobacterium phage Puhltonio	Viruses; dsDNA viruses, no RNA stage; Caudovirales; Siphoviridae.	663558	-
JF937095	EBI	RVG	G985	68,193	66.5	Mycobacterium phage Harvey	Viruses; dsDNA viruses, no RNA stage; Caudovirales; Siphoviridae.	1034104	-
JN638753	EBI	RVG	G985	68,307	66.4	Mycobacterium phage Morgushi	Viruses; dsDNA viruses, no RNA stage; Caudovirales; Siphoviridae.	1084721	-
NC_028801	RefSeq	RVG	G985	68,346	66.5	Mycobacterium phage Vortex	Viruses; dsDNA viruses, no RNA stage; Caudovirales; Siphoviridae.	1034149	-
JN699010	EBI	RVG	G985	68,133	66.5	Mycobacterium phage TallGRassMM	Viruses; dsDNA viruses, no RNA stage; Caudovirales; Siphoviridae.	1089107	-
JF937109	EBI	RVG	G985	68,719	66.5	Mycobacterium phage Yoshand	Viruses; dsDNA viruses, no RNA stage; Caudovirales; Siphoviridae.	1034119	-
KP027197	EBI	RVG	G985	68,716	66.5	Mycobacterium phage FluffyNinja	Viruses; dsDNA viruses, no RNA stage; Caudovirales; Siphoviridae.	1567469	-
NC_028658	RefSeq	RVG	G985	68,735	66.5	Mycobacterium phage Swish	Viruses; dsDNA viruses, no RNA stage; Caudovirales; Siphoviridae.	1458715	-
GU247133	EBI	RVG	G985	68,569	66.5	Mycobacterium phage Fang	Viruses; dsDNA viruses, no RNA stage; Caudovirales; Siphoviridae.	701454	-
NC_028942	RefSeq	RVG	G985	68,293	66.5	Mycobacterium phage Phipps	Viruses; dsDNA viruses, no RNA stage; Caudovirales; Siphoviridae.	1034143	-
KC661274	EBI	RVG	G985	67,702	66.5	Mycobacterium phage SDcharge11	Viruses; dsDNA viruses, no RNA stage; Caudovirales; Siphoviridae.	1327775	-
KJ538723	EBI	RVG	G985	68,043	66.5	Mycobacterium phage KingVeveve	Viruses; dsDNA viruses, no RNA stage; Caudovirales; Siphoviridae.	1471544	-
NC_025438	RefSeq	RVG	G985	67,744	66.6	Mycobacterium phage Soto	Viruses; dsDNA viruses, no RNA stage; Caudovirales; Siphoviridae.	1458725	Bacteria; Actinobacteria; Actinobacteria; Corynebacteriales; Mycobacteriaceae; Mycobacterium
JX649099	EBI	RVG	G985	68,004	66.5	Mycobacterium phage Gyarad	Viruses; dsDNA viruses, no RNA stage; Caudovirales; Siphoviridae.	1235691	-
KM347890	EBI	RVG	G985	68,873	66.4	Mycobacterium phage Vivaldi	Viruses; dsDNA viruses, no RNA stage; Caudovirales; Siphoviridae.	1536599	-
KM363597	EBI	RVG	G985	69,271	66.3	Mycobacterium phage Zonia	Viruses; dsDNA viruses, no RNA stage; Caudovirales; Siphoviridae.	1541820	-
NC_023711	RefSeq	RVG	G985	68,720	66.4	Mycobacterium phage Oline	Viruses; dsDNA viruses, no RNA stage; Caudovirales; Siphoviridae.	1052671	Bacteria; Actinobacteria; Actinobacteria; Corynebacteriales; Mycobacteriaceae; Mycobacterium

NC_028803	RefSeq	RVG	G985	69,118	66.3	Mycobacterium phage OSmaximus	Viruses; dsDNA viruses, no RNA stage; Caudovirales; Siphoviridae.	1035482	-
KF279413	EBI	RVG	G985	69,306	68.9	Mycobacterium phage Bane2	Viruses; dsDNA viruses, no RNA stage; Caudovirales; Siphoviridae.	1354509	-
NC_022331	RefSeq	RVG	G985	69,309	68.9	Mycobacterium phage Bane1	Viruses; dsDNA viruses, no RNA stage; Caudovirales; Siphoviridae.	1354508	Bacteria; Actinobacteria; Actinobacteria; Corynebacteriales; Mycobacteriaceae; Mycobacterium
NC_028968	RefSeq	RVG	G985	71,214	68.9	Mycobacterium phage BrownCNA	Viruses; dsDNA viruses, no RNA stage; Caudovirales; Siphoviridae.	1698252	-
NC_023554	RefSeq	RVG	G985	70,841	68.8	Mycobacterium phage JAMaL	Viruses; dsDNA viruses, no RNA stage; Caudovirales; Siphoviridae.	1429905	Bacteria; Actinobacteria; Actinobacteria; Corynebacteriales; Mycobacteriaceae; Mycobacterium
NC_022328	RefSeq	RVG	G985	70,236	68.8	Mycobacterium phage Adawi	Viruses; dsDNA viruses, no RNA stage; Caudovirales; Siphoviridae.	1354507	Bacteria; Actinobacteria; Actinobacteria; Corynebacteriales; Mycobacteriaceae; Mycobacterium
NC_008195	RefSeq	RVG	G985	70,654	69.1	Mycobacterium phage Cooper	Viruses; dsDNA viruses, no RNA stage; Caudovirales; Siphoviridae; Pgonelikevirus.	373406	Bacteria; Actinobacteria; Actinobacteria; Corynebacteriales; Mycobacteriaceae; Mycobacterium
NC_011044	RefSeq	RVG	G985	69,904	68.3	Mycobacterium phage Nigel	Viruses; dsDNA viruses, no RNA stage; Caudovirales; Siphoviridae; Pgonelikevirus.	543152	Bacteria; Actinobacteria; Actinobacteria; Corynebacteriales; Mycobacteriaceae; Mycobacterium
NC_023741	RefSeq	RVG	G985	69,641	68.6	Mycobacterium phage Stinger	Viruses; dsDNA viruses, no RNA stage; Caudovirales; Siphoviridae; Pgonelikevirus.	1089137	Bacteria; Actinobacteria; Actinobacteria; Corynebacteriales; Mycobacteriaceae; Mycobacterium
NC_028934	RefSeq	RVG	G985	72,139	68.9	Mycobacterium phage Vincenzo	Viruses; dsDNA viruses, no RNA stage; Caudovirales; Siphoviridae.	1647301	-
KF024727	EBI	RVG	G985	70,120	68.3	Mycobacterium phage Reprobate	Viruses; dsDNA viruses, no RNA stage; Caudovirales; Siphoviridae.	1340828	-
NC_022063	RefSeq	RVG	G985	70,115	68.3	Mycobacterium phage Phelemich	Viruses; dsDNA viruses, no RNA stage; Caudovirales; Siphoviridae.	1383055	Bacteria; Actinobacteria; Actinobacteria; Corynebacteriales; Mycobacteriaceae; Mycobacterium
NC_028742	RefSeq	RVG	G985	70,270	67.6	Mycobacterium phage Baee	Viruses; dsDNA viruses, no RNA stage; Caudovirales; Siphoviridae.	1647306	-
NC_023701	RefSeq	RVG	G985	69,864	68.4	Mycobacterium phage Acadian	Viruses; dsDNA viruses, no RNA stage; Caudovirales; Siphoviridae; Pgonelikevirus.	1089109	Bacteria; Actinobacteria; Actinobacteria; Corynebacteriales; Mycobacteriaceae; Mycobacterium
NC_024145	RefSeq	RVG	G985	70,667	69.9	Mycobacterium phage Hosp	Viruses; dsDNA viruses, no RNA stage; Caudovirales; Siphoviridae.	1463811	Bacteria; Actinobacteria; Actinobacteria; Corynebacteriales; Mycobacteriaceae; Mycobacterium
NC_023603	RefSeq	RVG	G985	71,565	70	Mycobacterium phage 39HC	Viruses; dsDNA viruses, no RNA stage; Caudovirales; Siphoviridae.	1463809	Bacteria; Actinobacteria; Actinobacteria; Corynebacteriales; Mycobacteriaceae; Mycobacterium
NC_023600	RefSeq	RVG	G985	71,058	69.9	Mycobacterium phage Jolie1	Viruses; dsDNA viruses, no RNA stage; Caudovirales; Siphoviridae.	1463812	Bacteria; Actinobacteria; Actinobacteria; Corynebacteriales; Mycobacteriaceae; Mycobacterium
NC_022061	RefSeq	RVG	G985	70,838	70	Mycobacterium phage KayaCho	Viruses; dsDNA viruses, no RNA stage; Caudovirales; Siphoviridae.	1340830	Bacteria; Actinobacteria; Actinobacteria; Corynebacteriales; Mycobacteriaceae; Mycobacterium
JN699018	EBI	RVG	G985	68,633	67.5	Mycobacterium phage Kamiyu	Viruses; dsDNA viruses, no RNA stage; Caudovirales; Siphoviridae.	1089126	-
NC_011057	RefSeq	RVG	G985	68,090	67.6	Mycobacterium phage Phaedrus	Viruses; dsDNA viruses, no RNA stage; Caudovirales; Siphoviridae.	546184	Bacteria; Actinobacteria; Actinobacteria; Corynebacteriales; Mycobacteriaceae; Mycobacterium
NC_012027	RefSeq	RVG	G985	69,378	67.5	Mycobacterium phage Phlyer	Viruses; dsDNA viruses, no RNA stage; Caudovirales; Siphoviridae.	591487	Bacteria; Actinobacteria; Actinobacteria; Corynebacteriales; Mycobacteriaceae; Mycobacterium
JN699003	EBI	RVG	G985	69,409	67.5	Mycobacterium phage Athena	Viruses; dsDNA viruses, no RNA stage; Caudovirales; Siphoviridae; Pgonelikevirus.	1089113	-
KJ194584	EBI	RVG	G985	68,628	67.5	Mycobacterium phage Heathcliff	Viruses; dsDNA viruses, no RNA stage; Caudovirales; Siphoviridae.	1458712	-
NC_023742	RefSeq	RVG	G985	68,711	67.5	Mycobacterium phage Akoma	Viruses; dsDNA viruses, no RNA stage; Caudovirales; Siphoviridae.	1089110	Bacteria; Actinobacteria; Actinobacteria; Corynebacteriales; Mycobacteriaceae; Mycobacterium
KP027207	EBI	RVG	G985	69,450	67.5	Mycobacterium phage Chandler	Viruses; dsDNA viruses, no RNA stage; Caudovirales; Siphoviridae.	1567465	-
KJ194581	EBI	RVG	G985	68,662	67.5	Mycobacterium phage Audrey	Viruses; dsDNA viruses, no RNA stage; Caudovirales; Siphoviridae.	1458709	-
NC_023686	RefSeq	RVG	G985	67,949	67.5	Mycobacterium phage Gadjet	Viruses; dsDNA viruses, no RNA stage; Caudovirales; Siphoviridae; Pgonelikevirus.	1089122	Bacteria; Actinobacteria; Actinobacteria; Corynebacteriales; Mycobacteriaceae; Mycobacterium
NC_022983	RefSeq	RVG	G985	68,196	67.4	Mycobacterium phage Bernardo	Viruses; dsDNA viruses, no RNA stage; Caudovirales; Siphoviridae.	1429903	Bacteria; Actinobacteria; Actinobacteria; Corynebacteriales; Mycobacteriaceae; Mycobacterium
NC_008199	RefSeq	RVG	G985	69,059	67.3	Mycobacterium phage Pipefish	Viruses; dsDNA viruses, no RNA stage; Caudovirales; Siphoviridae; Pgonelikevirus.	373413	Bacteria; Actinobacteria; Actinobacteria; Corynebacteriales; Mycobacteriaceae; Mycobacterium
JN698991	EBI	RVG	G985	67,451	69	Mycobacterium phage Hedgerow	Viruses; dsDNA viruses, no RNA stage; Caudovirales; Siphoviridae.	1089123	-
NC_004684	RefSeq	RVG	G985	67,480	69	Mycobacterium phage Rosebush	Viruses; dsDNA viruses, no RNA stage; Caudovirales; Siphoviridae; Pgonelikevirus.	205874	Bacteria; Actinobacteria; Actinobacteria; Corynebacteriales; Mycobacteriaceae; Mycobacterium
JN618996	EBI	RVG	G985	67,169	68.9	Mycobacterium phage Arbiter	Viruses; dsDNA viruses, no RNA stage; Caudovirales; Siphoviridae.	1079896	-
KF024722	EBI	RVG	G985	67,324	69	Mycobacterium phage TA17A	Viruses; dsDNA viruses, no RNA stage; Caudovirales; Siphoviridae; Pgonelikevirus.	1131248	-
JN699004	EBI	RVG	G985	67,436	69	Mycobacterium phage Ares	Viruses; dsDNA viruses, no RNA stage; Caudovirales; Siphoviridae.	1089112	-
NC_008204	RefSeq	RVG	G985	67,188	69	Mycobacterium phage Qyrzula	Viruses; dsDNA viruses, no RNA stage; Caudovirales; Siphoviridae.	373414	Bacteria; Actinobacteria; Actinobacteria; Corynebacteriales; Mycobacteriaceae; Mycobacterium
NC_015210	RefSeq	RVG	G985	61,440	69.6	Tsukamurella phage TPA2	Viruses; dsDNA viruses, no RNA stage; Caudovirales; Siphoviridae.	981330	Bacteria; Actinobacteria; Actinobacteria; Corynebacteriales; Tsukamurellaceae; Tsukamurella
TARA_ERS490120_N000185	TOV	EVG	G985	65,386	67.6	-	-	-	-
TARA_ERS490610_N000182	TOV	EVG	G985	65,386	67.6	-	-	-	-
NC_023703	RefSeq	RVG	G985	64,613	66	Mycobacterium phage Dori	Viruses; dsDNA viruses, no RNA stage; Caudovirales; Siphoviridae.	1089121	Bacteria; Actinobacteria; Actinobacteria; Corynebacteriales; Mycobacteriaceae; Mycobacterium
NC_023722	RefSeq	RVG	G986	59,231	67.1	Rhodococcus phage ReqiPine5	Viruses; dsDNA viruses, no RNA stage; Caudovirales; Siphoviridae.	691963	Bacteria; Actinobacteria; Actinobacteria; Corynebacteriales; Nocardiaceae; Rhodococcus
JN699625	EBI	RVG	G987	155,299	64.7	Mycobacterium phage Wally	Viruses; dsDNA viruses, no RNA stage; Caudovirales; Myoviridae; I3likevirus; unclassified I3-like viruses.	1088867	-
NC_011272	RefSeq	RVG	G987	153,894	64.7	Mycobacterium phage Rizal	Viruses; dsDNA viruses, no RNA stage; Caudovirales; Myoviridae; I3likevirus; unclassified I3-like viruses.	546806	Bacteria; Actinobacteria; Actinobacteria; Corynebacteriales; Mycobacteriaceae; Mycobacterium
KJ595575	EBI	RVG	G987	155,476	64.7	Mycobacterium phage Willis	Viruses; dsDNA viruses, no RNA stage; Caudovirales; Siphoviridae.	1486404	-
NC_008207	RefSeq	RVG	G987	153,766	64.7	Mycobacterium phage Catera	Viruses; dsDNA viruses, no RNA stage; Caudovirales; Myoviridae; I3likevirus; unclassified I3-like viruses.	373404	Bacteria; Actinobacteria; Actinobacteria; Corynebacteriales; Mycobacteriaceae; Mycobacterium
JQ911768	EBI	RVG	G987	154,466	64.8	Mycobacterium phage Ava3	Viruses; dsDNA viruses, no RNA stage; Caudovirales; Myoviridae.	1176426	-

NC_023737	RefSeq	RVG	G987	155,586	64.7	Mycobacterium phage Pleione	Viruses; dsDNA viruses, no RNA stage; Caudovirales; Myoviridae; I3likevirus; unclassified I3-like viruses.	1079895	Bacteria; Actinobacteria; Actinobacteria; Corynebacteriales; Mycobacteriaceae; Mycobacterium
NC_021346	RefSeq	RVG	G987	157,482	64.6	Mycobacterium phage Gizmo	Viruses; dsDNA viruses, no RNA stage; Caudovirales; Myoviridae; I3likevirus; unclassified I3-like viruses.	1327936	Bacteria; Actinobacteria; Actinobacteria; Corynebacteriales; Mycobacteriaceae; Mycobacterium
KF024734	EBI	RVG	G987	155,714	64.7	Mycobacterium phage Shrimp	Viruses; dsDNA viruses, no RNA stage; Caudovirales; Myoviridae.	1340835	-
NC_021348	RefSeq	RVG	G987	156,558	64.7	Mycobacterium phage ArcherS7	Viruses; dsDNA viruses, no RNA stage; Caudovirales; Myoviridae; I3likevirus; unclassified I3-like viruses.	1327938	Bacteria; Actinobacteria; Actinobacteria; Corynebacteriales; Mycobacteriaceae; Mycobacterium
NC_029044	RefSeq	RVG	G987	154,434	64.8	Mycobacterium phage Breeniome	Viruses; dsDNA viruses, no RNA stage; Caudovirales; Myoviridae.	1340712	-
NC_011270	RefSeq	RVG	G987	154,906	64.8	Mycobacterium phage Spud	Viruses; dsDNA viruses, no RNA stage; Caudovirales; Myoviridae; I3likevirus; unclassified I3-like viruses.	546808	Bacteria; Actinobacteria; Actinobacteria; Corynebacteriales; Mycobacteriaceae; Mycobacterium
NC_011269	RefSeq	RVG	G987	154,017	64.8	Mycobacterium phage ScottMcG	Viruses; dsDNA viruses, no RNA stage; Caudovirales; Myoviridae; I3likevirus; unclassified I3-like viruses.	546807	Bacteria; Actinobacteria; Actinobacteria; Corynebacteriales; Mycobacteriaceae; Mycobacterium
NC_023733	RefSeq	RVG	G987	154,573	64.8	Mycobacterium phage MoMoMixon	Viruses; dsDNA viruses, no RNA stage; Caudovirales; Myoviridae; I3likevirus; unclassified I3-like viruses.	1088865	Bacteria; Actinobacteria; Actinobacteria; Corynebacteriales; Mycobacteriaceae; Mycobacterium
JN699013	EBI	RVG	G987	156,758	64.8	Mycobacterium phage Pio	Viruses; dsDNA viruses, no RNA stage; Caudovirales; Myoviridae; I3likevirus; unclassified I3-like viruses.	1089131	-
NC_004687	RefSeq	RVG	G987	156,102	64.8	Mycobacterium phage Bxz1	Viruses; dsDNA viruses, no RNA stage; Caudovirales; Myoviridae; I3likevirus; unclassified I3-like viruses.	205877	Bacteria; Actinobacteria; Actinobacteria; Corynebacteriales; Mycobacteriaceae; Mycobacterium
NC_021349	RefSeq	RVG	G987	154,872	64.7	Mycobacterium phage Astraea	Viruses; dsDNA viruses, no RNA stage; Caudovirales; Myoviridae; I3likevirus; unclassified I3-like viruses.	1327762	Bacteria; Actinobacteria; Actinobacteria; Corynebacteriales; Mycobacteriaceae; Mycobacterium
NC_023725	RefSeq	RVG	G987	156,646	64.7	Mycobacterium phage Nappy	Viruses; dsDNA viruses, no RNA stage; Caudovirales; Myoviridae; I3likevirus; unclassified I3-like viruses.	1088866	Bacteria; Actinobacteria; Actinobacteria; Corynebacteriales; Mycobacteriaceae; Mycobacterium
GQ303262	EBI	RVG	G987	154,349	64.7	Mycobacterium phage LRRHood	Viruses; dsDNA viruses, no RNA stage; Caudovirales; Myoviridae; I3likevirus; unclassified I3-like viruses.	663556	-
NC_011271	RefSeq	RVG	G987	155,372	64.7	Mycobacterium phage Cali	Viruses; dsDNA viruses, no RNA stage; Caudovirales; Myoviridae; I3likevirus; unclassified I3-like viruses.	546803	Bacteria; Actinobacteria; Actinobacteria; Corynebacteriales; Mycobacteriaceae; Mycobacterium
NC_013650	RefSeq	RVG	G987	155,445	64.6	Mycobacterium phage ET08	Viruses; dsDNA viruses, no RNA stage; Caudovirales; Myoviridae; I3likevirus; unclassified I3-like viruses.	663554	Bacteria; Actinobacteria; Actinobacteria; Corynebacteriales; Mycobacteriaceae; Mycobacterium
JN204348	EBI	RVG	G987	155,286	64.8	Mycobacterium phage Sebata	Viruses; dsDNA viruses, no RNA stage; Caudovirales; Myoviridae; I3likevirus; unclassified I3-like viruses.	1052672	-
NC_023714	RefSeq	RVG	G987	153,882	64.8	Mycobacterium phage LinStu	Viruses; dsDNA viruses, no RNA stage; Caudovirales; Myoviridae; I3likevirus; unclassified I3-like viruses.	1074307	Bacteria; Actinobacteria; Actinobacteria; Corynebacteriales; Mycobacteriaceae; Mycobacterium
NC_023696	RefSeq	RVG	G987	157,568	64.7	Mycobacterium phage Dandelion	Viruses; dsDNA viruses, no RNA stage; Caudovirales; Myoviridae; I3likevirus; unclassified I3-like viruses.	1074305	Bacteria; Actinobacteria; Actinobacteria; Corynebacteriales; Mycobacteriaceae; Mycobacterium
NC_028986	RefSeq	RVG	G987	153,401	64.7	Mycobacterium phage Alice	Viruses; dsDNA viruses, no RNA stage; Caudovirales; Myoviridae.	1034128	-
NC_028869	RefSeq	RVG	G987	153,714	64.7	Mycobacterium phage HyRo	Viruses; dsDNA viruses, no RNA stage; Caudovirales; Myoviridae.	1698710	-
NC_011273	RefSeq	RVG	G987	164,602	65.4	Mycobacterium phage Myrna	Viruses; dsDNA viruses, no RNA stage; Caudovirales; Myoviridae.	546805	Bacteria; Actinobacteria; Actinobacteria; Corynebacteriales; Mycobacteriaceae; Mycobacterium
NC_021347	RefSeq	RVG	G987	142,563	67.5	Rhodococcus phage E3	Viruses; dsDNA viruses, no RNA stage; Caudovirales; Myoviridae.	1007869	Bacteria; Actinobacteria; Actinobacteria; Corynebacteriales; Nocardiaceae; Rhodococcus
NC_007967	RefSeq	RVG	G988	38,194	71.2	Streptomyces phage mu1/6	Viruses; dsDNA viruses, no RNA stage; Caudovirales; Siphoviridae.	370623	Bacteria; Actinobacteria; Actinobacteria; Streptomycetales; Streptomycetaceae; Streptomyces
NC_028974	RefSeq	RVG	G989	56,528	69.2	Streptomyces phage YDN12	Viruses; dsDNA viruses, no RNA stage; Caudovirales; Siphoviridae.	1636183	-
NC_028818	RefSeq	RVG	G989	57,168	69.2	Streptomyces phage TP1604	Viruses; dsDNA viruses, no RNA stage; Caudovirales; Siphoviridae.	1636184	-
NC_029005	RefSeq	RVG	G989	56,445	68.3	Streptomyces phage phiSAJS1	Viruses; dsDNA viruses, no RNA stage; Caudovirales; Siphoviridae.	1755682	-
NC_005885	RefSeq	RVG	G990	58,638	70.4	Actinoplanes phage phiAsp2	Viruses; dsDNA viruses, no RNA stage; Caudovirales; Siphoviridae.	279303	Bacteria; Actinobacteria; Actinobacteria; Micromonosporales; Micromonosporaceae; Actinoplanes
NC_016569	RefSeq	RVG	G991	46,140	67.5	Nocardia phage NBR1	Viruses; dsDNA viruses, no RNA stage; Caudovirales; Siphoviridae.	1109711	Bacteria; Actinobacteria; Actinobacteria; Corynebacteriales; Nocardiaceae; Nocardia
NC_020205	RefSeq	RVG	G992	42,963	67	Xanthomonas citri phage CP2	Viruses; dsDNA viruses, no RNA stage; Caudovirales; Podoviridae.	1188795	Bacteria; Proteobacteria; Gammaproteobacteria; Xanthomonadales; Xanthomonadaceae; Xanthomonas; Xanthomonas citri group
NC_018848	RefSeq	RVG	G993	37,612	72.7	Streptomyces phage SV1	Viruses; dsDNA viruses, no RNA stage; Caudovirales; Siphoviridae.	1204525	Bacteria; Actinobacteria; Actinobacteria; Streptomycetales; Streptomycetaceae; Streptomyces
NC_016654	RefSeq	RVG	G994	39,474	65.9	Rhodococcus phage REQ3	Viruses; unclassified phages.	1109714	Bacteria; Actinobacteria; Actinobacteria; Corynebacteriales; Nocardiaceae; Rhodococcus
NC_019410	RefSeq	RVG	G995	221,828	66.2	Caulobacter phage karma	Viruses; dsDNA viruses, no RNA stage; Caudovirales; Siphoviridae; Phicbklikevirus.	1211641	Bacteria; Proteobacteria; Alphaproteobacteria; Caulobacterales; Caulobacteraceae; Caulobacter
NC_019407	RefSeq	RVG	G995	218,929	66.1	Caulobacter phage magneto	Viruses; dsDNA viruses, no RNA stage; Caudovirales; Siphoviridae; Phicbklikevirus.	1211642	Bacteria; Proteobacteria; Alphaproteobacteria; Caulobacterales; Caulobacteraceae; Caulobacter
NC_019405	RefSeq	RVG	G995	215,710	66.2	Caulobacter phage phiCbK	Viruses; dsDNA viruses, no RNA stage; Caudovirales; Siphoviridae; Phicbklikevirus.	1204537	Bacteria; Proteobacteria; Alphaproteobacteria; Caulobacterales; Caulobacteraceae; Caulobacter
NC_019411	RefSeq	RVG	G995	219,216	66.1	Caulobacter phage swift	Viruses; dsDNA viruses, no RNA stage; Caudovirales; Siphoviridae; Phicbklikevirus.	1211644	Bacteria; Proteobacteria; Alphaproteobacteria; Caulobacterales; Caulobacteraceae; Caulobacter
NC_019408	RefSeq	RVG	G995	223,720	66.1	Caulobacter phage rogue	Viruses; dsDNA viruses, no RNA stage; Caudovirales; Siphoviridae; Phicbklikevirus.	1211643	Bacteria; Proteobacteria; Alphaproteobacteria; Caulobacterales; Caulobacteraceae; Caulobacter
NC_019406	RefSeq	RVG	G995	279,967	62.2	Caulobacter phage CcrColossus	Viruses; dsDNA viruses, no RNA stage; Caudovirales; Siphoviridae.	1211640	Bacteria; Proteobacteria; Alphaproteobacteria; Caulobacterales; Caulobacteraceae; Caulobacter
NC_028954	RefSeq	RVG	G996	36,065	65.4	Rhodobacter phage RcRhea	Viruses; dsDNA viruses, no RNA stage; Caudovirales.	1662332	-
NC_020998	RefSeq	RVG	G997	28,072	67.6	Haloarcula hispanica virus PH1	Viruses; Virus families not assigned to an order; Sphaerolipoviridae; Alphasphaerolipovirus.	1282967	Archaea; Euryarchaeota; Halobacteria; Halobacteriales; Halobacteriaceae; Haloarcula
NC_007217	RefSeq	RVG	G997	30,889	68.4	Haloarcula hispanica virus SH1	Viruses; Virus families not assigned to an order; Sphaerolipoviridae; Alphasphaerolipovirus.	326574	Archaea; Euryarchaeota; Halobacteria; Halobacteriales; Halobacteriaceae; Haloarcula
NC_016989	RefSeq	RVG	G997	30,578	66.5	Haloarcula hispanica icosahedral virus 2	Viruses; Virus families not assigned to an order; Sphaerolipoviridae; Alphasphaerolipovirus.	1154689	Archaea; Euryarchaeota; Halobacteria; Halobacteriales; Halobacteriaceae; Haloarcula

NC_013197	RefSeq	RVG	G998	17,036	68	Thermus thermophilus bacteriophage P23-77	Viruses; Virus families not assigned to an order; Sphaerolipoviridae; Gammasphaerolipovirus.		1714272	Bacteria; Deinococcus-Thermus; Deinococci; Thermales; Thermaceae; Thermus
NC_004462	RefSeq	RVG	G998	19,604	65.9	Thermus thermophilus phage IN93	Viruses; Virus families not assigned to an order; Sphaerolipoviridae; Gammasphaerolipovirus.		1714273	Bacteria; Deinococcus-Thermus; Deinococci; Thermales; Thermaceae; Thermus
NC_016655	RefSeq	RVG	G999	51,342	66.3	Rhodococcus phage REQ1	Viruses; dsDNA viruses, no RNA stage; Caudovirales; Siphoviridae.		1109712	Bacteria; Actinobacteria; Actinobacteria; Corynebacteriales; Nocardiaceae; Rhodococcus
NC_028952	RefSeq	RVG	G1000	60,934	69.1	Streptomyces phage SF3	Viruses; dsDNA viruses, no RNA stage; Caudovirales; Siphoviridae.		1690818	-
NC_028807	RefSeq	RVG	G1000	43,150	69.1	Streptomyces phage SF1	Viruses; dsDNA viruses, no RNA stage; Caudovirales; Siphoviridae.		1690817	-
NC_005345	RefSeq	RVG	G1000	49,220	71.1	Streptomyces phage VWB	Viruses; dsDNA viruses, no RNA stage; Caudovirales; Siphoviridae; Lambdalikevirus; unclassified Lambda-like viruses.		10702	Bacteria; Actinobacteria; Actinobacteria; Streptomycetales; Streptomycetaceae; Streptomyces
NC_024138	RefSeq	RVG	G1001	65,243	63.4	Mycobacterium phage MosMoris	Viruses; dsDNA viruses, no RNA stage; Caudovirales; Siphoviridae.		1471542	Bacteria; Actinobacteria; Actinobacteria; Corynebacteriales; Mycobacteriaceae; Mycobacterium
JX006077	EBI	RVG	G1002	94,870	65.9	Saccharomonospora phage PIS 136	Viruses; dsDNA viruses, no RNA stage; Caudovirales; Siphoviridae.		182851	-
NC_028665	RefSeq	RVG	G1003	56,982	67.8	Gordonia phage GTE6	Viruses; dsDNA viruses, no RNA stage; Caudovirales; Podoviridae.		1647474	-
TARA_ERS490346_N000146	TOV	EVG	G1004	66,263	63.6	-	-	-		-
NC_016651	RefSeq	RVG	G1005	14,270	68.4	Rhodococcus phage RRH1	Viruses; dsDNA viruses, no RNA stage; Caudovirales; Siphoviridae.		1109717	Bacteria; Actinobacteria; Actinobacteria; Corynebacteriales; Nocardiaceae; Rhodococcus
NC_028692	RefSeq	RVG	G1006	15,524	60.2	Arthrobacter phage Decurro	Viruses; dsDNA viruses, no RNA stage; Caudovirales; Siphoviridae.		1698361	-
JN561150	EBI	RVG	G1007	51,367	63.9	Mycobacterium phage TiroTheta9	Viruses; dsDNA viruses, no RNA stage; Caudovirales; Siphoviridae; L5likevirus.		1074829	-
JQ809701	EBI	RVG	G1007	51,370	63.9	Mycobacterium phage Flux	Viruses; dsDNA viruses, no RNA stage; Caudovirales; Siphoviridae; L5likevirus.		1168593	-
JX307703	EBI	RVG	G1007	51,377	63.9	Mycobacterium phage Sabertooth	Viruses; dsDNA viruses, no RNA stage; Caudovirales; Siphoviridae; L5likevirus.		1211284	-
KC661271	EBI	RVG	G1007	51,373	63.9	Mycobacterium phage Dhanush	Viruses; dsDNA viruses, no RNA stage; Caudovirales; Siphoviridae; L5likevirus.		1327769	-
KP057620	EBI	RVG	G1007	51,370	63.9	Mycobacterium phage HamSlice	Viruses; dsDNA viruses, no RNA stage; Caudovirales; Siphoviridae.		1567483	-
KP027198	EBI	RVG	G1007	51,376	64	Mycobacterium phage Gadost	Viruses; dsDNA viruses, no RNA stage; Caudovirales; Siphoviridae.		1567470	-
JN243856	EBI	RVG	G1007	51,368	63.9	Mycobacterium phage MeeZee	Viruses; dsDNA viruses, no RNA stage; Caudovirales; Siphoviridae; L5likevirus.		1056832	-
NC_013694	RefSeq	RVG	G1007	51,376	63.9	Mycobacterium phage Peaches	Viruses; dsDNA viruses, no RNA stage; Caudovirales; Siphoviridae; L5likevirus.		663557	Bacteria; Actinobacteria; Actinobacteria; Corynebacteriales; Mycobacteriaceae; Mycobacterium
JQ896627	EBI	RVG	G1007	51,440	63.9	Mycobacterium phage ICleared	Viruses; dsDNA viruses, no RNA stage; Caudovirales; Siphoviridae.		1176425	-
NC_024141	RefSeq	RVG	G1007	51,378	63.9	Mycobacterium phage Kampy	Viruses; dsDNA viruses, no RNA stage; Caudovirales; Siphoviridae.		1486425	Bacteria; Actinobacteria; Actinobacteria; Corynebacteriales; Mycobacteriaceae; Mycobacterium
NC_023562	RefSeq	RVG	G1007	51,236	63.9	Mycobacterium phage BellusTerra	Viruses; dsDNA viruses, no RNA stage; Caudovirales; Siphoviridae.		1429791	Bacteria; Actinobacteria; Actinobacteria; Corynebacteriales; Mycobacteriaceae; Mycobacterium
HM152766	EBI	RVG	G1007	51,436	63.9	Mycobacterium phage Eagle	Viruses; dsDNA viruses, no RNA stage; Caudovirales; Siphoviridae.		861046	-
KF841476	EBI	RVG	G1007	51,369	63.9	Mycobacterium phage Melvin	Viruses; dsDNA viruses, no RNA stage; Caudovirales; Siphoviridae.		1429792	-
KF024733	EBI	RVG	G1007	51,384	63.9	Mycobacterium phage Medusa	Viruses; dsDNA viruses, no RNA stage; Caudovirales; Siphoviridae.		1340834	-
NC_028687	RefSeq	RVG	G1007	51,637	64	Mycobacterium phage Iracema64	Viruses; dsDNA viruses, no RNA stage; Caudovirales; Siphoviridae.		1755681	-
NC_023565	RefSeq	RVG	G1007	51,250	63.9	Mycobacterium phage Nyxis	Viruses; dsDNA viruses, no RNA stage; Caudovirales; Siphoviridae.		1445714	Bacteria; Actinobacteria; Actinobacteria; Corynebacteriales; Mycobacteriaceae; Mycobacterium
JX307702	EBI	RVG	G1007	51,500	64.1	Mycobacterium phage Arturo	Viruses; dsDNA viruses, no RNA stage; Caudovirales; Siphoviridae; L5likevirus.		1211281	-
JN699015	EBI	RVG	G1007	51,813	63.9	Mycobacterium phage LHTSCC	Viruses; dsDNA viruses, no RNA stage; Caudovirales; Siphoviridae; L5likevirus.		1089129	-
NC_023577	RefSeq	RVG	G1007	51,797	64	Mycobacterium phage Obama12	Viruses; dsDNA viruses, no RNA stage; Caudovirales; Siphoviridae; L5likevirus.		1445715	Bacteria; Actinobacteria; Actinobacteria; Corynebacteriales; Mycobacteriaceae; Mycobacterium
NC_023709	RefSeq	RVG	G1007	51,308	63.7	Mycobacterium phage Wile	Viruses; dsDNA viruses, no RNA stage; Caudovirales; Siphoviridae.		1056833	Bacteria; Actinobacteria; Actinobacteria; Corynebacteriales; Mycobacteriaceae; Mycobacterium
JQ512844	EBI	RVG	G1007	51,094	65	Mycobacterium phage Twister	Viruses; dsDNA viruses, no RNA stage; Caudovirales; Siphoviridae; L5likevirus.		1161933	-
JX411619	EBI	RVG	G1007	51,235	65.1	Mycobacterium phage Rebeuca	Viruses; dsDNA viruses, no RNA stage; Caudovirales; Siphoviridae; L5likevirus.		1225862	-
JX307704	EBI	RVG	G1007	50,645	65.1	Mycobacterium phage Goose	Viruses; dsDNA viruses, no RNA stage; Caudovirales; Siphoviridae; L5likevirus.		1211282	-
NC_023609	RefSeq	RVG	G1007	46,739	65.3	Mycobacterium phage RhynO	Viruses; dsDNA viruses, no RNA stage; Caudovirales; Siphoviridae.		1458846	Bacteria; Actinobacteria; Actinobacteria; Corynebacteriales; Mycobacteriaceae; Mycobacterium
NC_021307	RefSeq	RVG	G1007	49,894	64.4	Mycobacterium phage Severus	Viruses; dsDNA viruses, no RNA stage; Caudovirales; Siphoviridae.		1327776	Bacteria; Actinobacteria; Actinobacteria; Corynebacteriales; Mycobacteriaceae; Mycobacterium
NC_026591	RefSeq	RVG	G1007	44,716	64.5	Mycobacterium phage Trike	Viruses; dsDNA viruses, no RNA stage; Caudovirales; Siphoviridae.		1527536	Bacteria; Actinobacteria; Actinobacteria; Corynebacteriales; Mycobacteriaceae; Mycobacterium
HM755814	EBI	RVG	G1007	48,491	64	Mycobacterium phage Wonder	Viruses; dsDNA viruses, no RNA stage; Caudovirales; Siphoviridae; L5likevirus; unclassified L5likevirus.		927988	-
NC_028798	RefSeq	RVG	G1007	50,882	64	Mycobacterium phage MarQuardt	Viruses; dsDNA viruses, no RNA stage; Caudovirales; Siphoviridae; L5likevirus; unclassified L5likevirus.		1527516	-
NC_028757	RefSeq	RVG	G1007	50,768	64	Mycobacterium phage Tiffany	Viruses; dsDNA viruses, no RNA stage; Caudovirales; Siphoviridae; L5likevirus; unclassified L5likevirus.		1527535	-
KP027202	EBI	RVG	G1007	50,877	64	Mycobacterium phage Taurus	Viruses; dsDNA viruses, no RNA stage; Caudovirales; Siphoviridae; L5likevirus; unclassified L5likevirus.		1567477	-
NC_029018	RefSeq	RVG	G1007	50,856	64	Mycobacterium phage Anubis	Viruses; dsDNA viruses, no RNA stage; Caudovirales; Siphoviridae; L5likevirus; unclassified L5likevirus.		1354511	-
KM233455	EBI	RVG	G1007	49,190	64.2	Mycobacterium phage Farber	Viruses; dsDNA viruses, no RNA stage; Caudovirales; Siphoviridae.		1527517	-

NC_021535	RefSeq	RVG	G1007	50,679	64	Mycobacterium phage Jobu08	Viruses; dsDNA viruses, no RNA stage; Caudovirales; Siphoviridae.	1327772	Bacteria; Actinobacteria; Actinobacteria; Corynebacteriales; Mycobacteriaceae; Mycobacterium
KP017311	EBI	RVG	G1007	50,989	64.1	Mycobacterium phage Spike509	Viruses; dsDNA viruses, no RNA stage; Caudovirales; Siphoviridae.	1567452	-
KP017310	EBI	RVG	G1007	49,267	64.2	Mycobacterium phage Phoxy	Viruses; dsDNA viruses, no RNA stage; Caudovirales; Siphoviridae.	1567451	-
KC661272	EBI	RVG	G1007	50,891	64.2	Mycobacterium phage Methuselah	Viruses; dsDNA viruses, no RNA stage; Caudovirales; Siphoviridae; L5likevirus.	1327779	-
NC_004682	RefSeq	RVG	G1007	50,913	64.2	Mycobacterium phage Bxz2	Viruses; dsDNA viruses, no RNA stage; Caudovirales; Siphoviridae; L5likevirus.	205870	Bacteria; Actinobacteria; Actinobacteria; Corynebacteriales; Mycobacteriaceae; Mycobacterium
NC_021533	RefSeq	RVG	G1007	45,942	64.2	Mycobacterium phage BTCU-1	Viruses; dsDNA viruses, no RNA stage; Caudovirales; Siphoviridae.	1262532	Bacteria; Actinobacteria; Actinobacteria; Corynebacteriales; Mycobacteriaceae; Mycobacterium
NC_024148	RefSeq	RVG	G1007	50,101	63.8	Mycobacterium phage Phantastic	Viruses; dsDNA viruses, no RNA stage; Caudovirales; Siphoviridae.	1486426	Bacteria; Actinobacteria; Actinobacteria; Corynebacteriales; Mycobacteriaceae; Mycobacterium
JF957058	EBI	RVG	G1007	50,364	64	Mycobacterium phage HelDan	Viruses; dsDNA viruses, no RNA stage; Caudovirales; Siphoviridae; L5likevirus.	1032892	-
KM592966	EBI	RVG	G1007	50,066	64	Mycobacterium phage QuinnKiro	Viruses; dsDNA viruses, no RNA stage; Caudovirales; Siphoviridae.	1551642	-
JF957060	EBI	RVG	G1007	53,278	63.1	Mycobacterium phage Timshel	Viruses; dsDNA viruses, no RNA stage; Caudovirales; Siphoviridae; L5likevirus.	1032895	-
NC_021308	RefSeq	RVG	G1007	52,617	62.8	Mycobacterium phage HINdeR	Viruses; dsDNA viruses, no RNA stage; Caudovirales; Siphoviridae.	1327770	Bacteria; Actinobacteria; Actinobacteria; Corynebacteriales; Mycobacteriaceae; Mycobacterium
NC_028914	RefSeq	RVG	G1007	52,927	63.4	Mycobacterium phage Sheen	Viruses; dsDNA viruses, no RNA stage; Caudovirales; Siphoviridae.	1589274	-
KM463009	EBI	RVG	G1007	53,395	63.5	Mycobacterium phage Power	Viruses; dsDNA viruses, no RNA stage; Caudovirales; Siphoviridae.	1552733	-
NC_029043	RefSeq	RVG	G1007	53,183	63.5	Mycobacterium phage SweetiePie	Viruses; dsDNA viruses, no RNA stage; Caudovirales; Siphoviridae.	1555235	-
NC_023707	RefSeq	RVG	G1007	53,169	63.3	Mycobacterium phage Turbido	Viruses; dsDNA viruses, no RNA stage; Caudovirales; Siphoviridae; L5likevirus.	1071504	Bacteria; Actinobacteria; Actinobacteria; Corynebacteriales; Mycobacteriaceae; Mycobacterium
NC_023553	RefSeq	RVG	G1007	53,159	63.7	Mycobacterium phage Echild	Viruses; dsDNA viruses, no RNA stage; Caudovirales; Siphoviridae.	1437839	Bacteria; Actinobacteria; Actinobacteria; Corynebacteriales; Mycobacteriaceae; Mycobacterium
NC_020876	RefSeq	RVG	G1007	53,028	63.4	Mycobacterium phage First	Viruses; dsDNA viruses, no RNA stage; Caudovirales; Siphoviridae.	1245814	Bacteria; Actinobacteria; Actinobacteria; Corynebacteriales; Mycobacteriaceae; Mycobacterium
NC_023597	RefSeq	RVG	G1007	53,124	63.4	Mycobacterium phage 20ES	Viruses; dsDNA viruses, no RNA stage; Caudovirales; Siphoviridae.	1458726	Bacteria; Actinobacteria; Actinobacteria; Corynebacteriales; Mycobacteriaceae; Mycobacterium
NC_028744	RefSeq	RVG	G1007	53,141	63.5	Mycobacterium phage LadyBird	Viruses; dsDNA viruses, no RNA stage; Caudovirales; Siphoviridae.	1718166	-
NC_023606	RefSeq	RVG	G1007	52,963	63.2	Mycobacterium phage CRB1	Viruses; dsDNA viruses, no RNA stage; Caudovirales; Siphoviridae.	1458841	Bacteria; Actinobacteria; Actinobacteria; Corynebacteriales; Mycobacteriaceae; Mycobacterium
NC_028852	RefSeq	RVG	G1007	53,042	63.5	Mycobacterium phage Equemioh13	Viruses; dsDNA viruses, no RNA stage; Caudovirales; Siphoviridae.	1555201	-
NC_028794	RefSeq	RVG	G1007	52,647	63.4	Mycobacterium phage Piro94	Viruses; dsDNA viruses, no RNA stage; Caudovirales; Siphoviridae.	1527520	-
NC_028877	RefSeq	RVG	G1007	52,967	63.5	Mycobacterium phage Larenn	Viruses; dsDNA viruses, no RNA stage; Caudovirales; Siphoviridae.	1560285	-
NC_022087	RefSeq	RVG	G1007	53,253	64.4	Mycobacterium phage AnnaL29	Viruses; dsDNA viruses, no RNA stage; Caudovirales; Siphoviridae.	1076630	Bacteria; Actinobacteria; Actinobacteria; Corynebacteriales; Mycobacteriaceae; Mycobacterium
NC_025444	RefSeq	RVG	G1007	53,332	64.5	Mycobacterium phage RedRock	Viruses; dsDNA viruses, no RNA stage; Caudovirales; Siphoviridae; L5likevirus.	711470	Bacteria; Actinobacteria; Actinobacteria; Corynebacteriales; Mycobacteriaceae; Mycobacterium
NC_023731	RefSeq	RVG	G1007	53,526	64.5	Mycobacterium phage Trixie	Viruses; dsDNA viruses, no RNA stage; Caudovirales; Siphoviridae; L5likevirus.	1071503	Bacteria; Actinobacteria; Actinobacteria; Corynebacteriales; Mycobacteriaceae; Mycobacterium
NC_023607	RefSeq	RVG	G1007	53,396	63.3	Mycobacterium phage 40AC	Viruses; dsDNA viruses, no RNA stage; Caudovirales; Siphoviridae.	1458717	Bacteria; Actinobacteria; Actinobacteria; Corynebacteriales; Mycobacteriaceae; Mycobacterium
NC_011023	RefSeq	RVG	G1007	52,892	63.3	Mycobacterium phage Pukovnik	Viruses; dsDNA viruses, no RNA stage; Caudovirales; Siphoviridae; L5likevirus.	540068	Bacteria; Actinobacteria; Actinobacteria; Corynebacteriales; Mycobacteriaceae; Mycobacterium
NC_021318	RefSeq	RVG	G1007	51,214	63.6	Mycobacterium phage Chy5	Viruses; dsDNA viruses, no RNA stage; Caudovirales; Siphoviridae.	1327948	Bacteria; Actinobacteria; Actinobacteria; Corynebacteriales; Mycobacteriaceae; Mycobacterium; Mycobacterium tuberculosis complex
NC_021338	RefSeq	RVG	G1007	46,639	63.7	Mycobacterium phage Chy4	Viruses; dsDNA viruses, no RNA stage; Caudovirales; Siphoviridae.	1327947	Bacteria; Actinobacteria; Actinobacteria; Corynebacteriales; Mycobacteriaceae; Mycobacterium; Mycobacterium tuberculosis complex
NC_001900	RefSeq	RVG	G1007	49,136	63.5	Mycobacterium phage D29	Viruses; dsDNA viruses, no RNA stage; Caudovirales; Siphoviridae; L5likevirus.	28369	Bacteria; Actinobacteria; Actinobacteria; Corynebacteriales; Mycobacteriaceae; Mycobacterium; Mycobacterium tuberculosis complex
NC_017973	RefSeq	RVG	G1007	52,474	62.4	Mycobacterium phage SWU1	Viruses; dsDNA viruses, no RNA stage; Caudovirales; Siphoviridae; L5likevirus.	1175504	Bacteria; Actinobacteria; Actinobacteria; Corynebacteriales; Mycobacteriaceae; Mycobacterium
NC_001335	RefSeq	RVG	G1007	52,297	62.3	Mycobacterium phage L5	Viruses; dsDNA viruses, no RNA stage; Caudovirales; Siphoviridae; L5likevirus.	31757	Bacteria; Actinobacteria; Actinobacteria; Corynebacteriales; Mycobacteriaceae
NC_028932	RefSeq	RVG	G1007	52,088	62.6	Mycobacterium phage Serenity	Viruses; dsDNA viruses, no RNA stage; Caudovirales; Siphoviridae.	1701853	-
NC_022058	RefSeq	RVG	G1007	52,519	62.6	Mycobacterium phage Adzzy	Viruses; dsDNA viruses, no RNA stage; Caudovirales; Siphoviridae.	1383059	Bacteria; Actinobacteria; Actinobacteria; Corynebacteriales; Mycobacteriaceae; Mycobacterium
NC_008203	RefSeq	RVG	G1007	52,047	62.9	Mycobacterium phage Che12	Viruses; dsDNA viruses, no RNA stage; Caudovirales; Siphoviridae; L5likevirus.	373405	Bacteria; Actinobacteria; Actinobacteria; Corynebacteriales; Mycobacteriaceae; Mycobacterium
KF017927	EBI	RVG	G1007	52,807	62.3	Mycobacterium phage Odin	Viruses; dsDNA viruses, no RNA stage; Caudovirales; Siphoviridae.	1340711	-
JN699019	EBI	RVG	G1007	48,963	61.6	Mycobacterium phage Jeffabunny	Viruses; dsDNA viruses, no RNA stage; Caudovirales; Siphoviridae; L5likevirus.	1089125	-
NC_022965	RefSeq	RVG	G1007	52,873	61.4	Mycobacterium phage CloudWang3	Viruses; dsDNA viruses, no RNA stage; Caudovirales; Siphoviridae.	1391430	Bacteria; Actinobacteria; Actinobacteria; Corynebacteriales; Mycobacteriaceae; Mycobacterium
NC_022985	RefSeq	RVG	G1007	52,122	61.5	Mycobacterium phage Zaka	Viruses; dsDNA viruses, no RNA stage; Caudovirales; Siphoviridae.	1391433	Bacteria; Actinobacteria; Actinobacteria; Corynebacteriales; Mycobacteriaceae; Mycobacterium
NC_022977	RefSeq	RVG	G1007	52,344	61.4	Mycobacterium phage Artemis2UCLA	Viruses; dsDNA viruses, no RNA stage; Caudovirales; Siphoviridae.	1391429	Bacteria; Actinobacteria; Actinobacteria; Corynebacteriales; Mycobacteriaceae; Mycobacterium
JF937094	EBI	RVG	G1007	51,889	61.3	Mycobacterium phage Hammer	Viruses; dsDNA viruses, no RNA stage; Caudovirales; Siphoviridae; L5likevirus.	1034103	-
NC_023713	RefSeq	RVG	G1007	52,288	61.4	Mycobacterium phage Blue7	Viruses; dsDNA viruses, no RNA stage; Caudovirales; Siphoviridae.	1089117	Bacteria; Actinobacteria; Actinobacteria; Corynebacteriales; Mycobacteriaceae; Mycobacterium

JF937092	EBI	RVG	G1007	51,547	61.5	Mycobacterium phage DaVinci	Viruses; dsDNA viruses, no RNA stage; Caudovirales; Siphoviridae.	1034102	-
NC_029077	RefSeq	RVG	G1007	52,155	61.5	Mycobacterium phage VohminGhazi	Viruses; dsDNA viruses, no RNA stage; Caudovirales; Siphoviridae.	1542912	-
JN049605	EBI	RVG	G1007	51,702	61.5	Mycobacterium phage EricB	Viruses; dsDNA viruses, no RNA stage; Caudovirales; Siphoviridae; L5likevirus.	1041406	-
NC_023564	RefSeq	RVG	G1007	52,974	61.4	Mycobacterium phage EagleEye	Viruses; dsDNA viruses, no RNA stage; Caudovirales; Siphoviridae.	1429759	Bacteria; Actinobacteria; Actinobacteria; Corynebacteriales; Mycobacteriaceae; Mycobacterium
NC_028965	RefSeq	RVG	G1007	53,219	62.6	Mycobacterium phage Pioneer	Viruses; dsDNA viruses, no RNA stage; Caudovirales; Siphoviridae.	1698417	-
NC_023716	RefSeq	RVG	G1007	53,177	62.5	Mycobacterium phage Alma	Viruses; dsDNA viruses, no RNA stage; Caudovirales; Siphoviridae; L5likevirus.	1089111	Bacteria; Actinobacteria; Actinobacteria; Corynebacteriales; Mycobacteriaceae; Mycobacterium
NC_028849	RefSeq	RVG	G1007	53,387	62.1	Mycobacterium phage Luchador	Viruses; dsDNA viruses, no RNA stage; Caudovirales; Siphoviridae.	1647300	-
NC_028662	RefSeq	RVG	G1007	50,418	60.1	Mycobacterium phage Phlei	Viruses; dsDNA viruses, no RNA stage; Caudovirales; Podoviridae.	1690684	-
NC_022086	RefSeq	RVG	G1007	50,690	60.9	Mycobacterium phage LittleCherry	Viruses; dsDNA viruses, no RNA stage; Caudovirales; Siphoviridae.	1340818	Bacteria; Actinobacteria; Actinobacteria; Corynebacteriales; Mycobacteriaceae; Mycobacterium
NC_028912	RefSeq	RVG	G1007	49,717	61	Mycobacterium phage Swirley	Viruses; dsDNA viruses, no RNA stage; Caudovirales; Siphoviridae.	1527534	-
JN408459	EBI	RVG	G1007	50,965	60.9	Mycobacterium phage Cuco	Viruses; dsDNA viruses, no RNA stage; Caudovirales; Siphoviridae; L5likevirus.	1071502	-
NC_028960	RefSeq	RVG	G1007	50,999	60.8	Mycobacterium phage Theia	Viruses; dsDNA viruses, no RNA stage; Caudovirales; Siphoviridae.	1718172	-
NC_022984	RefSeq	RVG	G1007	51,319	60.8	Mycobacterium phage Jovo	Viruses; dsDNA viruses, no RNA stage; Caudovirales; Siphoviridae.	1429912	Bacteria; Actinobacteria; Actinobacteria; Corynebacteriales; Mycobacteriaceae; Mycobacterium
NC_022973	RefSeq	RVG	G1007	50,755	60.6	Mycobacterium phage Conspiracy	Viruses; dsDNA viruses, no RNA stage; Caudovirales; Siphoviridae.	1391431	Bacteria; Actinobacteria; Actinobacteria; Corynebacteriales; Mycobacteriaceae; Mycobacterium
JQ684677	EBI	RVG	G1007	50,332	60.7	Mycobacterium phage Tiger	Viruses; dsDNA viruses, no RNA stage; Caudovirales; Siphoviridae; L5likevirus.	1161934	-
JN083853	EBI	RVG	G1007	51,241	60	Mycobacterium phage Airmid	Viruses; dsDNA viruses, no RNA stage; Caudovirales; Siphoviridae.	1041808	-
JX042578	EBI	RVG	G1007	51,505	59.8	Mycobacteriophage ElTiger69	Viruses; dsDNA viruses, no RNA stage; Caudovirales.	1195069	-
JN083852	EBI	RVG	G1007	51,083	59.8	Mycobacterium phage Benedict	Viruses; dsDNA viruses, no RNA stage; Caudovirales; Siphoviridae; L5likevirus.	1041807	-
NC_028897	RefSeq	RVG	G1007	49,421	59.8	Mycobacterium phage Chadwick	Viruses; dsDNA viruses, no RNA stage; Caudovirales; Siphoviridae.	1698366	-
NC_028802	RefSeq	RVG	G1007	49,158	60	Mycobacterium phage UnionJack	Viruses; dsDNA viruses, no RNA stage; Caudovirales; Siphoviridae.	1673876	-
JX015524	EBI	RVG	G1007	52,494	61.4	Mycobacterium phage Astro	Viruses; dsDNA viruses, no RNA stage; Caudovirales; Siphoviridae; L5likevirus; unclassified L5likevirus.	1195075	-
NC_028860	RefSeq	RVG	G1007	52,392	61.4	Mycobacterium phage Smeadley	Viruses; dsDNA viruses, no RNA stage; Caudovirales; Siphoviridae; L5likevirus; unclassified L5likevirus.	1673873	-
JN831654	EBI	RVG	G1007	49,228	61.2	Mycobacterium phage Saintus	Viruses; dsDNA viruses, no RNA stage; Caudovirales; Siphoviridae; L5likevirus.	1097754	-
NC_022753	RefSeq	RVG	G1007	52,282	61.2	Mycobacterium phage Fredward	Viruses; dsDNA viruses, no RNA stage; Caudovirales; Siphoviridae.	1354510	Bacteria; Actinobacteria; Actinobacteria; Corynebacteriales; Mycobacteriaceae; Mycobacterium
NC_016653	RefSeq	RVG	G1007	46,586	58.6	Rhodococcus phage RER2	Viruses; dsDNA viruses, no RNA stage; Caudovirales; Siphoviridae; L5likevirus.	1109715	Bacteria; Actinobacteria; Actinobacteria; Corynebacteriales; Nocardiaceae; Rhodococcus
NC_028677	RefSeq	RVG	G1007	46,596	58.5	Rhodococcus phage CosmicSans	Viruses; dsDNA viruses, no RNA stage; Caudovirales; Siphoviridae.	1701851	-
NC_016650	RefSeq	RVG	G1007	48,072	62.7	Rhodococcus phage RGL3	Viruses; dsDNA viruses, no RNA stage; Caudovirales; Siphoviridae; L5likevirus.	1109716	Bacteria; Actinobacteria; Actinobacteria; Corynebacteriales; Nocardiaceae; Rhodococcus
NC_028784	RefSeq	RVG	G1007	51,409	63.9	Mycobacterium phage Tasp14	Viruses; dsDNA viruses, no RNA stage; Caudovirales; Siphoviridae.	1698420	-
NC_024143	RefSeq	RVG	G1007	53,255	63.7	Mycobacterium phage Lamina13	Viruses; dsDNA viruses, no RNA stage; Caudovirales; Siphoviridae.	1468169	Bacteria; Actinobacteria; Actinobacteria; Corynebacteriales; Mycobacteriaceae; Mycobacterium
NC_011267	RefSeq	RVG	G1007	49,487	63.8	Mycobacterium phage Solon	Viruses; dsDNA viruses, no RNA stage; Caudovirales; Siphoviridae; L5likevirus.	555603	Bacteria; Actinobacteria; Actinobacteria; Corynebacteriales; Mycobacteriaceae; Mycobacterium
NC_022062	RefSeq	RVG	G1007	52,102	63.6	Mycobacterium phage Trouble	Viruses; dsDNA viruses, no RNA stage; Caudovirales; Siphoviridae.	1340825	Bacteria; Actinobacteria; Actinobacteria; Corynebacteriales; Mycobacteriaceae; Mycobacterium
NC_028928	RefSeq	RVG	G1007	53,455	63.7	Mycobacterium phage Nerujay	Viruses; dsDNA viruses, no RNA stage; Caudovirales; Siphoviridae.	1647308	-
KP027203	EBI	RVG	G1007	53,008	63.9	Mycobacterium phage Treddle	Viruses; dsDNA viruses, no RNA stage; Caudovirales; Siphoviridae.	1567479	-
NC_029059	RefSeq	RVG	G1007	51,492	64	Mycobacterium phage Edtherson	Viruses; dsDNA viruses, no RNA stage; Caudovirales; Siphoviridae.	1567468	-
JF937100	EBI	RVG	G1007	50,486	63.8	Mycobacterium phage Lesedi	Viruses; dsDNA viruses, no RNA stage; Caudovirales; Siphoviridae; L5likevirus.	1034110	-
NC_026583	RefSeq	RVG	G1007	49,577	63.6	Mycobacterium phage Alvin	Viruses; dsDNA viruses, no RNA stage; Caudovirales; Siphoviridae.	1567466	Bacteria; Actinobacteria; Actinobacteria; Corynebacteriales; Mycobacteriaceae; Mycobacterium
KF279416	EBI	RVG	G1007	53,693	63.7	Mycobacterium phage SargentShorty9	Viruses; dsDNA viruses, no RNA stage; Caudovirales; Siphoviridae.	1354513	-
NC_022329	RefSeq	RVG	G1007	53,636	63.7	Mycobacterium phage PhrostyMug	Viruses; dsDNA viruses, no RNA stage; Caudovirales; Siphoviridae.	1354512	Bacteria; Actinobacteria; Actinobacteria; Corynebacteriales; Mycobacteriaceae; Mycobacterium
JQ698665	EBI	RVG	G1007	53,972	63.5	Mycobacterium phage Nepal	Viruses; dsDNA viruses, no RNA stage; Caudovirales; Siphoviridae; L5likevirus.	1194642	-
NC_023723	RefSeq	RVG	G1007	53,684	63.6	Mycobacterium phage Aeneas	Viruses; dsDNA viruses, no RNA stage; Caudovirales; Siphoviridae.	1168595	Bacteria; Actinobacteria; Actinobacteria; Corynebacteriales; Mycobacteriaceae; Mycobacterium
NC_023720	RefSeq	RVG	G1007	53,142	63.7	Mycobacterium phage Perseus	Viruses; dsDNA viruses, no RNA stage; Caudovirales; Siphoviridae; L5likevirus.	1076136	Bacteria; Actinobacteria; Actinobacteria; Corynebacteriales; Mycobacteriaceae; Mycobacterium
NC_028920	RefSeq	RVG	G1007	52,530	63.8	Mycobacterium phage Abrogate	Viruses; dsDNA viruses, no RNA stage; Caudovirales; Siphoviridae.	1551710	-
NC_023710	RefSeq	RVG	G1007	50,844	64	Mycobacterium phage RidgeCB	Viruses; dsDNA viruses, no RNA stage; Caudovirales; Siphoviridae; L5likevirus.	1071506	Bacteria; Actinobacteria; Actinobacteria; Corynebacteriales; Mycobacteriaceae; Mycobacterium
JF937099	EBI	RVG	G1007	52,169	63.6	Mycobacterium phage JC27	Viruses; dsDNA viruses, no RNA stage; Caudovirales; Siphoviridae; L5likevirus.	1034108	-
JX307705	EBI	RVG	G1007	49,186	64	Mycobacterium phage Marcell	Viruses; dsDNA viruses, no RNA stage; Caudovirales; Siphoviridae; L5likevirus.	1211283	-
NC_021324	RefSeq	RVG	G1007	53,369	63.6	Mycobacterium phage CASbig	Viruses; dsDNA viruses, no RNA stage; Caudovirales; Siphoviridae.	1327035	Bacteria; Actinobacteria; Actinobacteria; Corynebacteriales; Mycobacteriaceae; Mycobacterium

NC_022070	RefSeq	RVG	G1007	53,588	63.3	Mycobacterium phage Wheeler	Viruses; dsDNA viruses, no RNA stage; Caudovirales; Siphoviridae.	1383054	Bacteria; Actinobacteria; Actinobacteria; Corynebacteriales; Mycobacteriaceae; Mycobacterium
NC_011021	RefSeq	RVG	G1007	51,478	63.4	Mycobacterium phage Lockley	Viruses; dsDNA viruses, no RNA stage; Caudovirales; Siphoviridae; L5likevirus.	540067	Bacteria; Actinobacteria; Actinobacteria; Corynebacteriales; Mycobacteriaceae; Mycobacterium
NC_011022	RefSeq	RVG	G1007	51,621	63.4	Mycobacterium phage DD5	Viruses; dsDNA viruses, no RNA stage; Caudovirales; Siphoviridae.	540064	Bacteria; Actinobacteria; Actinobacteria; Corynebacteriales; Mycobacteriaceae; Mycobacterium
NC_023748	RefSeq	RVG	G1007	53,137	63.6	Mycobacterium phage SkiPole	Viruses; dsDNA viruses, no RNA stage; Caudovirales; Siphoviridae; L5likevirus.	701456	Bacteria; Actinobacteria; Actinobacteria; Corynebacteriales; Mycobacteriaceae; Mycobacterium
JF957057	EBI	RVG	G1007	53,171	63.4	Mycobacterium phage BBPiebs31	Viruses; dsDNA viruses, no RNA stage; Caudovirales; Siphoviridae; L5likevirus.	1032891	-
NC_024368	RefSeq	RVG	G1007	50,610	63.5	Mycobacterium phage Pinto	Viruses; dsDNA viruses, no RNA stage; Caudovirales; Siphoviridae.	1498204	Bacteria; Actinobacteria; Actinobacteria; Corynebacteriales; Mycobacteriaceae; Mycobacterium
NC_009878	RefSeq	RVG	G1007	52,250	63.2	Mycobacterium phage Bethlehem	Viruses; dsDNA viruses, no RNA stage; Caudovirales; Siphoviridae; L5likevirus.	260121	Bacteria; Actinobacteria; Actinobacteria; Corynebacteriales; Mycobacteriaceae; Mycobacterium
NC_028810	RefSeq	RVG	G1007	52,357	63.9	Mycobacterium phage Rufus	Viruses; dsDNA viruses, no RNA stage; Caudovirales; Siphoviridae.	1698441	-
NC_028941	RefSeq	RVG	G1007	51,161	63.7	Mycobacterium phage Turj99	Viruses; dsDNA viruses, no RNA stage; Caudovirales; Siphoviridae.	1701849	-
KP027204	EBI	RVG	G1007	53,058	63.7	Mycobacterium phage Thor	Viruses; dsDNA viruses, no RNA stage; Caudovirales; Siphoviridae.	1567478	-
NC_022324	RefSeq	RVG	G1007	53,701	63.8	Mycobacterium phage Sarfire	Viruses; dsDNA viruses, no RNA stage; Caudovirales; Siphoviridae.	1340827	Bacteria; Actinobacteria; Actinobacteria; Corynebacteriales; Mycobacteriaceae; Mycobacterium
JF937103	EBI	RVG	G1007	51,426	63.6	Mycobacterium phage Museum	Viruses; dsDNA viruses, no RNA stage; Caudovirales; Siphoviridae; L5likevirus.	1034113	-
NC_021297	RefSeq	RVG	G1007	52,057	63.6	Mycobacterium phage PattyP	Viruses; dsDNA viruses, no RNA stage; Caudovirales; Siphoviridae.	1327773	Bacteria; Actinobacteria; Actinobacteria; Corynebacteriales; Mycobacteriaceae; Mycobacterium
JN020140	EBI	RVG	G1007	50,988	63.8	Mycobacterium phage MrGordo	Viruses; dsDNA viruses, no RNA stage; Caudovirales; Siphoviridae; L5likevirus.	1036613	-
NC_023695	RefSeq	RVG	G1007	52,481	63.8	Mycobacterium phage Violet	Viruses; dsDNA viruses, no RNA stage; Caudovirales; Siphoviridae; L5likevirus.	1086800	Bacteria; Actinobacteria; Actinobacteria; Corynebacteriales; Mycobacteriaceae; Mycobacterium
NC_022975	RefSeq	RVG	G1007	52,390	63.8	Mycobacterium phage HanShotFirst	Viruses; dsDNA viruses, no RNA stage; Caudovirales; Siphoviridae.	1429904	Bacteria; Actinobacteria; Actinobacteria; Corynebacteriales; Mycobacteriaceae; Mycobacterium
NC_028815	RefSeq	RVG	G1007	51,355	63.8	Mycobacterium phage Nhonho	Viruses; dsDNA viruses, no RNA stage; Caudovirales; Siphoviridae.	1675553	-
NC_028804	RefSeq	RVG	G1007	52,643	63.4	Mycobacterium phage Barriga	Viruses; dsDNA viruses, no RNA stage; Caudovirales; Siphoviridae.	1675548	-
JF937108	EBI	RVG	G1007	52,298	63.8	Mycobacterium phage Switzer	Viruses; dsDNA viruses, no RNA stage; Caudovirales; Siphoviridae; L5likevirus.	1034118	-
NC_009877	RefSeq	RVG	G1007	51,277	63.7	Mycobacterium phage U2	Viruses; dsDNA viruses, no RNA stage; Caudovirales; Siphoviridae; L5likevirus.	260120	Bacteria; Actinobacteria; Actinobacteria; Corynebacteriales; Mycobacteriaceae; Mycobacterium
JF937110	EBI	RVG	G1007	51,381	63.6	Mycobacterium phage KSSJEB	Viruses; dsDNA viruses, no RNA stage; Caudovirales; Siphoviridae; L5likevirus.	1034109	-
NC_024136	RefSeq	RVG	G1007	51,781	63.7	Mycobacterium phage Seabiscuit	Viruses; dsDNA viruses, no RNA stage; Caudovirales; Siphoviridae.	1458714	Bacteria; Actinobacteria; Actinobacteria; Corynebacteriales; Mycobacteriaceae; Mycobacterium
NC_011020	RefSeq	RVG	G1007	50,968	63.7	Mycobacterium phage Jasper	Viruses; dsDNA viruses, no RNA stage; Caudovirales; Siphoviridae; L5likevirus.	540065	Bacteria; Actinobacteria; Actinobacteria; Corynebacteriales; Mycobacteriaceae; Mycobacterium
NC_023708	RefSeq	RVG	G1007	51,083	63.9	Mycobacterium phage Dreamboat	Viruses; dsDNA viruses, no RNA stage; Caudovirales; Siphoviridae.	1086749	Bacteria; Actinobacteria; Actinobacteria; Corynebacteriales; Mycobacteriaceae; Mycobacterium
NC_023726	RefSeq	RVG	G1007	53,597	63.7	Mycobacterium phage Euphoria	Viruses; dsDNA viruses, no RNA stage; Caudovirales; Siphoviridae; L5likevirus.	1051140	Bacteria; Actinobacteria; Actinobacteria; Corynebacteriales; Mycobacteriaceae; Mycobacterium
NC_023687	RefSeq	RVG	G1007	53,003	63.6	Mycobacterium phage Bruns	Viruses; dsDNA viruses, no RNA stage; Caudovirales; Siphoviridae; L5likevirus.	1089118	Bacteria; Actinobacteria; Actinobacteria; Corynebacteriales; Mycobacteriaceae; Mycobacterium
NC_028874	RefSeq	RVG	G1007	50,614	63.5	Mycobacterium phage Pari	Viruses; dsDNA viruses, no RNA stage; Caudovirales; Siphoviridae.	1718171	-
NC_011019	RefSeq	RVG	G1007	53,572	63.6	Mycobacterium phage KBG	Viruses; dsDNA viruses, no RNA stage; Caudovirales; Siphoviridae; L5likevirus.	540066	Bacteria; Actinobacteria; Actinobacteria; Corynebacteriales; Mycobacteriaceae; Mycobacterium
NC_023704	RefSeq	RVG	G1007	51,421	63.8	Mycobacterium phage Doom	Viruses; dsDNA viruses, no RNA stage; Caudovirales; Siphoviridae; L5likevirus.	1051138	Bacteria; Actinobacteria; Actinobacteria; Corynebacteriales; Mycobacteriaceae; Mycobacterium
NC_022979	RefSeq	RVG	G1007	52,823	63.5	Mycobacterium phage Graduation	Viruses; dsDNA viruses, no RNA stage; Caudovirales; Siphoviridae.	1391432	Bacteria; Actinobacteria; Actinobacteria; Corynebacteriales; Mycobacteriaceae; Mycobacterium
NC_023862	RefSeq	RVG	G1007	52,136	63.6	Mycobacterium phage Alsfro	Viruses; dsDNA viruses, no RNA stage; Caudovirales; Siphoviridae.	1458724	Bacteria; Actinobacteria; Actinobacteria; Corynebacteriales; Mycobacteriaceae; Mycobacterium
NC_028828	RefSeq	RVG	G1007	52,055	63.6	Mycobacterium phage TheloniousMonk	Viruses; dsDNA viruses, no RNA stage; Caudovirales; Siphoviridae.	1701845	-
NC_028680	RefSeq	RVG	G1007	50,515	64	Mycobacterium phage Pepe	Viruses; dsDNA viruses, no RNA stage; Caudovirales; Siphoviridae.	1735466	-
NC_002656	RefSeq	RVG	G1007	50,550	63.6	Mycobacterium phage Bxb1	Viruses; dsDNA viruses, no RNA stage; Caudovirales; Siphoviridae; L5likevirus.	148603	Bacteria; Actinobacteria; Actinobacteria; Corynebacteriales; Mycobacteriaceae; Mycobacterium
NC_023739	RefSeq	RVG	G1007	51,821	63.4	Mycobacterium phage BillKnuckles	Viruses; dsDNA viruses, no RNA stage; Caudovirales; Siphoviridae; L5likevirus.	1089116	Bacteria; Actinobacteria; Actinobacteria; Corynebacteriales; Mycobacteriaceae; Mycobacterium
NC_023702	RefSeq	RVG	G1007	52,379	63.8	Mycobacterium phage Kugel	Viruses; dsDNA viruses, no RNA stage; Caudovirales; Siphoviridae; L5likevirus.	1089128	Bacteria; Actinobacteria; Actinobacteria; Corynebacteriales; Mycobacteriaceae; Mycobacterium
NC_021304	RefSeq	RVG	G1008	51,552	65.7	Streptomyces phage Sujidade	Viruses; dsDNA viruses, no RNA stage; Caudovirales; Siphoviridae.	1327759	Bacteria; Actinobacteria; Actinobacteria; Streptomycetales; Streptomycetaceae; Streptomyces
NC_021298	RefSeq	RVG	G1008	51,252	65.8	Streptomyces phage Lika	Viruses; dsDNA viruses, no RNA stage; Caudovirales; Siphoviridae.	1327758	Bacteria; Actinobacteria; Actinobacteria; Streptomycetales; Streptomycetaceae; Streptomyces
NC_021339	RefSeq	RVG	G1008	51,077	65.7	Streptomyces phage Zemlya	Viruses; dsDNA viruses, no RNA stage; Caudovirales; Siphoviridae.	1327760	Bacteria; Actinobacteria; Actinobacteria; Streptomycetales; Streptomycetaceae; Streptomyces
NC_028827	RefSeq	RVG	G1008	50,165	65.7	Streptomyces phage Lannister	Viruses; dsDNA viruses, no RNA stage; Caudovirales; Siphoviridae.	1674927	-
NC_028976	RefSeq	RVG	G1008	50,113	65.9	Streptomyces phage Izzy	Viruses; dsDNA viruses, no RNA stage; Caudovirales; Siphoviridae.	1674926	-
NC_028892	RefSeq	RVG	G1008	49,949	66.2	Streptomyces phage Caliburn	Viruses; dsDNA viruses, no RNA stage; Caudovirales; Siphoviridae.	1690425	-
JX889246	EBI	RVG	G1008	50,348	65.6	Streptomyces phage phiCAM	Viruses; dsDNA viruses, no RNA stage; Caudovirales; Siphoviridae.	1239386	-

NC_028904	RefSeq	RVG	G1008	49,452	65.6	Streptomyces phage Amela	Viruses; dsDNA viruses, no RNA stage; Caudovirales; Siphoviridae.	1673877	-
JX262376	EBI	RVG	G1008	51,160	67	Streptomyces phage phiELB20	Viruses; dsDNA viruses, no RNA stage; Caudovirales; Siphoviridae.	1211278	-
NC_019414	RefSeq	RVG	G1008	51,071	67	Streptomyces phage R4	Viruses; dsDNA viruses, no RNA stage; Caudovirales; Siphoviridae.	10732	Bacteria; Actinobacteria; Actinobacteria; Streptomycetales; Streptomycetaceae; Streptomyces
NC_018836	RefSeq	RVG	G1008	50,255	67.8	Streptomyces phage phiHau3	Viruses; dsDNA viruses, no RNA stage; Caudovirales; Siphoviridae.	1204524	Bacteria; Actinobacteria; Actinobacteria; Streptomycetales; Streptomycetaceae; Streptomyces
NC_004664	RefSeq	RVG	G1009	41,831	62.8	Streptomyces phage phiBT1	Viruses; dsDNA viruses, no RNA stage; Caudovirales; Siphoviridae; Phic3unalikevirus.	225588	Bacteria; Actinobacteria; Actinobacteria; Streptomycetales; Streptomycetaceae; Streptomyces
NC_001978	RefSeq	RVG	G1009	41,491	63.6	Streptomyces phage phiC31	Viruses; dsDNA viruses, no RNA stage; Caudovirales; Siphoviridae; Phic3unalikevirus.	10719	Bacteria; Actinobacteria; Actinobacteria; Streptomycetales; Streptomycetaceae; Streptomyces
NC_018853	RefSeq	RVG	G1009	40,474	64.6	Streptomyce phage TG1	Viruses; dsDNA viruses, no RNA stage; Caudovirales; Siphoviridae.	1204526	Bacteria; Actinobacteria; Actinobacteria; Streptomycetales; Streptomycetaceae; Streptomyces
NC_014229	RefSeq	RVG	G1010	37,068	66.3	Streptomyces phage phiSASD1	Viruses; dsDNA viruses, no RNA stage; Caudovirales; Siphoviridae; Phic3unalikevirus; unclassified Phic3unalikevirus.	747763	Bacteria; Actinobacteria; Actinobacteria; Streptomycetales; Streptomycetaceae; Streptomyces
NC_026606	RefSeq	RVG	G1011	71,200	61.6	Arthrobacter phage vB_ArtM-ArV1	Viruses; dsDNA viruses, no RNA stage; Caudovirales; Myoviridae.	1566993	Bacteria; Actinobacteria; Actinobacteria; Micrococcales; Micrococcaceae; Arthrobacter
KC661276	EBI	RVG	G1012	76,653	58.9	Mycobacterium phage Winky	Viruses; dsDNA viruses, no RNA stage; Caudovirales; Siphoviridae; Bronlikevirus.	1327778	-
NC_015584	RefSeq	RVG	G1012	75,960	58.9	Mycobacterium phage Faith1	Viruses; dsDNA viruses, no RNA stage; Caudovirales; Siphoviridae; Bronlikevirus.	1034125	Bacteria; Actinobacteria; Actinobacteria; Corynebacteriales; Mycobacteriaceae; Mycobacterium
NC_022071	RefSeq	RVG	G1012	76,129	58.9	Mycobacterium phage Crossroads	Viruses; dsDNA viruses, no RNA stage; Caudovirales; Siphoviridae.	1340836	Bacteria; Actinobacteria; Actinobacteria; Corynebacteriales; Mycobacteriaceae; Mycobacterium
NC_023732	RefSeq	RVG	G1012	69,279	58.9	Mycobacterium phage Rumpelstiltskin	Viruses; dsDNA viruses, no RNA stage; Caudovirales; Siphoviridae; Bronlikevirus.	1086751	Bacteria; Actinobacteria; Actinobacteria; Corynebacteriales; Mycobacteriaceae; Mycobacterium
NC_028878	RefSeq	RVG	G1012	76,271	58.7	Mycobacterium phage Archie	Viruses; dsDNA viruses, no RNA stage; Caudovirales; Siphoviridae.	1718599	-
JN600672	EBI	RVG	G1012	74,155	58.7	Mycobacterium phage Fezzik	Viruses; dsDNA viruses, no RNA stage; Caudovirales; Siphoviridae.	1131256	-
NC_014461	RefSeq	RVG	G1012	73,453	58.8	Mycobacterium phage bron	Viruses; dsDNA viruses, no RNA stage; Caudovirales; Siphoviridae; Bronlikevirus.	861047	Bacteria; Actinobacteria; Actinobacteria; Corynebacteriales; Mycobacteriaceae; Mycobacterium
NC_022052	RefSeq	RVG	G1012	76,050	59.3	Mycobacterium phage Whirlwind	Viruses; dsDNA viruses, no RNA stage; Caudovirales; Siphoviridae.	1340826	Bacteria; Actinobacteria; Actinobacteria; Corynebacteriales; Mycobacteriaceae; Mycobacterium
NC_028843	RefSeq	RVG	G1012	75,816	59.3	Mycobacterium phage Lolly9	Viruses; dsDNA viruses, no RNA stage; Caudovirales; Siphoviridae.	1698711	-
NC_028778	RefSeq	RVG	G1012	75,626	59.3	Mycobacterium phage Snenia	Viruses; dsDNA viruses, no RNA stage; Caudovirales; Siphoviridae.	1698714	-
JN699628	EBI	RVG	G1013	80,228	61.6	Mycobacterium phage Bongo	Viruses; dsDNA viruses, no RNA stage; Caudovirales; Siphoviridae; Reylikevirus.	1088864	-
NC_021299	RefSeq	RVG	G1013	80,955	61.5	Mycobacterium phage PegLeg	Viruses; dsDNA viruses, no RNA stage; Caudovirales; Siphoviridae.	1325953	Bacteria; Actinobacteria; Actinobacteria; Corynebacteriales; Mycobacteriaceae; Mycobacterium
JF937105	EBI	RVG	G1013	83,724	60.9	Mycobacterium phage Rey	Viruses; dsDNA viruses, no RNA stage; Caudovirales; Siphoviridae; Reylikevirus.	1034115	-
KP027195	EBI	RVG	G1014	78,229	56.8	Mycobacterium phage Cosmo	Viruses; dsDNA viruses, no RNA stage; Caudovirales; Siphoviridae.	1567467	-
NC_008206	RefSeq	RVG	G1014	78,296	56.9	Mycobacterium phage Wildcat	Viruses; dsDNA viruses, no RNA stage; Caudovirales; Siphoviridae.	373415	Bacteria; Actinobacteria; Actinobacteria
NC_029075	RefSeq	RVG	G1015	81,541	60.1	Achromobacter phage JWF	Viruses; dsDNA viruses, no RNA stage; Caudovirales; Siphoviridae.	1589748	-
NC_010355	RefSeq	RVG	G1016	62,337	63.9	Azospirillum phage Cd	Viruses; dsDNA viruses, no RNA stage; Caudovirales; Siphoviridae.	467481	Bacteria; Proteobacteria; Alphaproteobacteria; Rhodospirillales; Rhodospirillaceae; Azospirillum
NC_021560	RefSeq	RVG	G1017	53,102	57.2	Rhizobium phage RR1-A	Viruses; dsDNA viruses, no RNA stage; Caudovirales; Myoviridae.	929833	Bacteria; Proteobacteria; Alphaproteobacteria; Rhizobiales; Rhizobiaceae; Agrobacterium; Agrobacterium tumefaciens complex
NC_025427	RefSeq	RVG	G1017	54,506	61.9	Rhizobium phage vB_RleM_PPF1	Viruses; dsDNA viruses, no RNA stage; Caudovirales; Myoviridae.	1498228	Bacteria; Proteobacteria; Alphaproteobacteria; Rhizobiales; Rhizobiaceae; Rhizobium
NC_027334	RefSeq	RVG	G1018	47,800	66.7	Aurantimonas phage AmM-1	Viruses; dsDNA viruses, no RNA stage; Caudovirales.	1503929	Bacteria; Proteobacteria; Alphaproteobacteria; Rhizobiales; Aurantimonadaceae; Aurantimonas
NC_020489	RefSeq	RVG	G1019	40,489	65.1	Rhodobacter phage RcapNL	Viruses; dsDNA viruses, no RNA stage; unclassified dsDNA phages.	1131316	Bacteria; Proteobacteria; Alphaproteobacteria; Rhodobacterales; Rhodobacteraceae; Rhodobacter
KF981876	EBI	RVG	G1020	95,702	62.7	Mycobacterium phage ADLER F1725	Viruses; dsDNA viruses, no RNA stage; Caudovirales; Siphoviridae.	1453337	-
NC_023591	RefSeq	RVG	G1020	95,705	62.7	Mycobacterium phage Adler	Viruses; dsDNA viruses, no RNA stage; Caudovirales; Siphoviridae.	1327959	Bacteria; Actinobacteria; Actinobacteria; Corynebacteriales; Mycobacteriaceae; Mycobacterium; Mycobacterium chelonae group; Mycobacterium abscessus subgroup; Mycobacterium abscessus
TARA_ERS488448_N000368	TOV	EVG	G1021	39,468	64.3	-	-	-	-
EU676000	EBI	RVG	G1022	64,511	59.7	Mycobacterium phage Adjutor	Viruses; dsDNA viruses, no RNA stage; Caudovirales; Siphoviridae; Pbiunalikevirus.	528321	-
NC_008198	RefSeq	RVG	G1022	64,494	59.7	Mycobacterium phage PBI1	Viruses; dsDNA viruses, no RNA stage; Caudovirales; Siphoviridae; Pbiunalikevirus.	373410	Bacteria; Actinobacteria; Actinobacteria; Corynebacteriales; Mycobacteriaceae; Mycobacterium
FJ168660	EBI	RVG	G1022	64,562	59.7	Mycobacterium phage Butterscotch	Viruses; dsDNA viruses, no RNA stage; Caudovirales; Siphoviridae; Pbiunalikevirus.	561997	-
NC_011285	RefSeq	RVG	G1022	64,618	59.6	Mycobacterium phage Troll4	Viruses; dsDNA viruses, no RNA stage; Caudovirales; Siphoviridae.	561999	Bacteria; Actinobacteria; Actinobacteria; Corynebacteriales; Mycobacteriaceae; Mycobacterium
NC_008200	RefSeq	RVG	G1022	64,787	59.7	Mycobacterium phage PLot	Viruses; dsDNA viruses, no RNA stage; Caudovirales; Siphoviridae.	373411	Bacteria; Actinobacteria; Actinobacteria; Corynebacteriales; Mycobacteriaceae; Mycobacterium
JF937107	EBI	RVG	G1022	64,791	59.6	Mycobacterium phage SirHarley	Viruses; dsDNA viruses, no RNA stage; Caudovirales; Siphoviridae.	1034117	-
NC_011290	RefSeq	RVG	G1022	64,807	59.6	Mycobacterium phage Gumball	Viruses; dsDNA viruses, no RNA stage; Caudovirales; Siphoviridae.	561998	Bacteria; Actinobacteria; Actinobacteria; Corynebacteriales; Mycobacteriaceae; Mycobacterium
JN699014	EBI	RVG	G1022	65,108	59.7	Mycobacterium phage Nova	Viruses; dsDNA viruses, no RNA stage; Caudovirales; Siphoviridae.	1089130	-
NC_024209	RefSeq	RVG	G1022	67,383	57	Mycobacterium phage Hawkeye	Viruses; dsDNA viruses, no RNA stage; Caudovirales; Siphoviridae.	1458711	Bacteria; Actinobacteria; Actinobacteria; Corynebacteriales; Mycobacteriaceae; Mycobacterium
NC_024371	RefSeq	RVG	G1023	68,386	57.6	Mycobacterium phage Damien	Viruses; dsDNA viruses, no RNA stage; Caudovirales; Siphoviridae.	1486469	Bacteria; Actinobacteria; Actinobacteria; Corynebacteriales; Mycobacteriaceae; Mycobacterium
NC_023578	RefSeq	RVG	G1023	69,099	57.5	Mycobacterium phage Oaker	Viruses; dsDNA viruses, no RNA stage; Caudovirales; Siphoviridae.	1445727	Bacteria; Actinobacteria; Actinobacteria; Corynebacteriales; Mycobacteriaceae; Mycobacterium

NC_011292	RefSeq	RVG	G1023	68,952	57.3	Mycobacterium phage Konstantine	Viruses; dsDNA viruses, no RNA stage; Caudovirales; Siphoviridae; Barnyardlikevirus.	563121	Bacteria; Actinobacteria; Actinobacteria; Corynebacteriales; Mycobacteriaceae; Mycobacterium
NC_011039	RefSeq	RVG	G1023	70,110	56.3	Mycobacterium phage Predator	Viruses; dsDNA viruses, no RNA stage; Caudovirales; Siphoviridae; Barnyardlikevirus.	543153	Bacteria; Actinobacteria; Actinobacteria; Corynebacteriales; Mycobacteriaceae; Mycobacterium
NC_004689	RefSeq	RVG	G1023	70,797	57.3	Mycobacterium phage Barnyard	Viruses; dsDNA viruses, no RNA stage; Caudovirales; Siphoviridae; Barnyardlikevirus.	205880	Bacteria; Actinobacteria; Actinobacteria; Corynebacteriales; Mycobacteriaceae; Mycobacterium
NC_023691	RefSeq	RVG	G1023	70,506	50.3	Mycobacterium phage Patience	Viruses; dsDNA viruses, no RNA stage; Caudovirales; Siphoviridae; Barnyardlikevirus.	1074308	Bacteria; Actinobacteria; Actinobacteria; Corynebacteriales; Mycobacteriaceae; Mycobacterium
NC_022053	RefSeq	RVG	G1024	70,657	56	Mycobacterium phage Papyrus	Viruses; dsDNA viruses, no RNA stage; Caudovirales; Siphoviridae.	1383056	Bacteria; Actinobacteria; Actinobacteria; Corynebacteriales; Mycobacteriaceae; Mycobacterium
NC_016434	RefSeq	RVG	G1025	65,839	65.1	Gordonia phage GTE5	Viruses; dsDNA viruses, no RNA stage; Caudovirales; Siphoviridae.	319522	Bacteria; Actinobacteria; Actinobacteria; Corynebacteriales; Gordoniaceae; Gordonia
NC_016435	RefSeq	RVG	G1025	65,766	65.5	Gordonia phage GRU1	Viruses; dsDNA viruses, no RNA stage; Caudovirales; Siphoviridae.	1109710	Bacteria; Actinobacteria; Actinobacteria; Corynebacteriales; Gordoniaceae; Gordonia
NC_028653	RefSeq	RVG	G1025	67,617	66	Gordonia phage GTE8	Viruses; dsDNA viruses, no RNA stage; Caudovirales; Podoviridae.	1647475	-
NC_009816	RefSeq	RVG	G1026	70,579	50.9	Corynebacterium phage P1201	Viruses; dsDNA viruses, no RNA stage; Caudovirales; Siphoviridae.	384848	Bacteria; Actinobacteria; Actinobacteria; Corynebacteriales; Corynebacteriaceae; Corynebacterium
KP790011	EBI	RVG	G1027	43,505	62.8	Gordonia phage Gsput1	Viruses; unclassified phages.	1622193	-
NC_009799	RefSeq	RVG	G1028	42,972	56.2	Corynebacterium phage BFK20	Viruses; dsDNA viruses, no RNA stage; Caudovirales; Siphoviridae.	28358	Bacteria; Actinobacteria; Actinobacteria; Corynebacteriales; Corynebacteriaceae; Corynebacterium
JN811560	EBI	RVG	G1029	37,840	64.1	Pseudomonas phage JBD26	Viruses; dsDNA viruses, no RNA stage; Caudovirales; Siphoviridae.	1093672	-
NC_005178	RefSeq	RVG	G1029	37,611	64.3	Pseudomonas phage D3112	Viruses; dsDNA viruses, no RNA stage; Caudovirales; Siphoviridae; D3112likevirus.	10708	Bacteria; Proteobacteria; Gammaproteobacteria; Pseudomonadales; Pseudomonadaceae; Pseudomonas; Pseudomonas aeruginosa group
NC_027384	RefSeq	RVG	G1029	37,359	64.2	Pseudomonas phage H70	Viruses; dsDNA viruses, no RNA stage; Caudovirales; Siphoviridae.	1536596	Bacteria; Proteobacteria; Gammaproteobacteria; Pseudomonadales; Pseudomonadaceae; Pseudomonas; Pseudomonas aeruginosa group
NC_027298	RefSeq	RVG	G1029	36,814	64.4	Pseudomonas phage LPB1	Viruses; dsDNA viruses, no RNA stage; Caudovirales; Siphoviridae; D3112likevirus.	1091556	Bacteria; Proteobacteria; Gammaproteobacteria; Pseudomonadales; Pseudomonadaceae; Pseudomonas; Pseudomonas aeruginosa group
JQ067085	EBI	RVG	G1029	36,570	64.2	Pseudomonas phage PaMx73	Viruses; dsDNA viruses, no RNA stage; Caudovirales; Siphoviridae; D3112likevirus.	1175655	-
NC_011613	RefSeq	RVG	G1029	36,632	64.3	Pseudomonas phage MP29	Viruses; dsDNA viruses, no RNA stage; Caudovirales; Siphoviridae; D3112likevirus.	505291	Bacteria; Proteobacteria; Gammaproteobacteria; Pseudomonadales; Pseudomonadaceae; Pseudomonas; Pseudomonas aeruginosa group
NC_023700	RefSeq	RVG	G1029	34,553	64.8	Pseudomonas phage PA1phi	Viruses; dsDNA viruses, no RNA stage; Caudovirales; Siphoviridae; D3112likevirus.	937835	Bacteria; Proteobacteria; Gammaproteobacteria; Pseudomonadales; Pseudomonadaceae; Pseudomonas; Pseudomonas aeruginosa group
NC_020202	RefSeq	RVG	G1029	37,740	64.3	Pseudomonas phage JBD5	Viruses; dsDNA viruses, no RNA stage; Caudovirales; Siphoviridae.	1223261	Bacteria; Proteobacteria; Gammaproteobacteria; Pseudomonadales; Pseudomonadaceae; Pseudomonas; Pseudomonas aeruginosa group
NC_024330	RefSeq	RVG	G1029	37,380	64.2	Pseudomonas phage JD024	Viruses; dsDNA viruses, no RNA stage; Caudovirales; Siphoviridae.	1481224	Bacteria; Proteobacteria; Gammaproteobacteria; Pseudomonadales; Pseudomonadaceae; Pseudomonas; Pseudomonas aeruginosa group
NC_024782	RefSeq	RVG	G1029	36,838	64.1	Pseudomonas phage MP48	Viruses; dsDNA viruses, no RNA stage; Caudovirales; Siphoviridae; Lambdalikevirus; unclassified Lambda-like viruses.	1391190	Bacteria; Proteobacteria; Gammaproteobacteria; Pseudomonadales; Pseudomonadaceae; Pseudomonas; Pseudomonas aeruginosa group
NC_020198	RefSeq	RVG	G1029	36,947	64.3	Pseudomonas phage JBD30	Viruses; dsDNA viruses, no RNA stage; Caudovirales; Siphoviridae.	1223260	Bacteria; Proteobacteria; Gammaproteobacteria; Pseudomonadales; Pseudomonadaceae; Pseudomonas; Pseudomonas aeruginosa group
NC_018274	RefSeq	RVG	G1029	36,847	64.2	Pseudomonas phage MP42	Viruses; dsDNA viruses, no RNA stage; Caudovirales; Siphoviridae; Lambdalikevirus; unclassified Lambda-like viruses.	1161903	Bacteria; Proteobacteria; Gammaproteobacteria; Pseudomonadales; Pseudomonadaceae; Pseudomonas; Pseudomonas aeruginosa group
NC_008717	RefSeq	RVG	G1029	36,415	64.3	Pseudomonas phage DMS3	Viruses; dsDNA viruses, no RNA stage; Caudovirales; Siphoviridae; D3112likevirus.	389469	Bacteria; Proteobacteria; Gammaproteobacteria; Pseudomonadales; Pseudomonadaceae; Pseudomonas; Pseudomonas aeruginosa group
NC_020203	RefSeq	RVG	G1029	37,095	64.2	Pseudomonas phage JBD24	Viruses; dsDNA viruses, no RNA stage; Caudovirales; Siphoviridae.	1223259	Bacteria; Proteobacteria; Gammaproteobacteria; Pseudomonadales; Pseudomonadaceae; Pseudomonas; Pseudomonas aeruginosa group
NC_026601	RefSeq	RVG	G1029	37,238	64.1	Pseudomonas phage vB_PaeS_PAO1_Ab30	Viruses; dsDNA viruses, no RNA stage; Caudovirales; Siphoviridae.	1548918	Bacteria; Proteobacteria; Gammaproteobacteria; Pseudomonadales; Pseudomonadaceae; Pseudomonas; Pseudomonas aeruginosa group
NC_020200	RefSeq	RVG	G1029	36,429	64	Pseudomonas phage JBD88a	Viruses; dsDNA viruses, no RNA stage; Caudovirales; Siphoviridae.	1223262	Bacteria; Proteobacteria; Gammaproteobacteria; Pseudomonadales; Pseudomonadaceae; Pseudomonas; Pseudomonas aeruginosa group
NC_009818	RefSeq	RVG	G1029	36,409	64.2	Pseudomonas phage MP22	Viruses; dsDNA viruses, no RNA stage; Caudovirales; Siphoviridae; D3112likevirus.	397353	Bacteria; Proteobacteria; Gammaproteobacteria; Pseudomonadales; Pseudomonadaceae; Pseudomonas; Pseudomonas aeruginosa group
JN808773	EBI	RVG	G1029	37,374	64.1	Pseudomonas phage FHA0480	Viruses; dsDNA viruses, no RNA stage; Caudovirales; Siphoviridae; D3112likevirus.	1093100	-
NC_011611	RefSeq	RVG	G1029	36,885	64.5	Pseudomonas phage MP38	Viruses; dsDNA viruses, no RNA stage; Caudovirales; Siphoviridae; D3112likevirus.	505292	Bacteria; Proteobacteria; Gammaproteobacteria; Pseudomonadales; Pseudomonadaceae; Pseudomonas; Pseudomonas aeruginosa group
NC_028667	RefSeq	RVG	G1029	39,593	63.1	Pseudomonas phage vB_PaeS_PM105	Viruses; dsDNA viruses, no RNA stage; Caudovirales; Siphoviridae.	1743016	-
NC_027986	RefSeq	RVG	G1029	39,014	63.4	Pseudomonas phage JBD18	Viruses; dsDNA viruses, no RNA stage; Caudovirales; Siphoviridae.	1225791	Bacteria; Proteobacteria; Gammaproteobacteria; Pseudomonadales; Pseudomonadaceae
NC_006548	RefSeq	RVG	G1029	38,439	63.2	Pseudomonas phage B3	Viruses; dsDNA viruses, no RNA stage; Caudovirales; Siphoviridae.	151599	Bacteria; Proteobacteria; Gammaproteobacteria; Pseudomonadales; Pseudomonadaceae; Pseudomonas; Pseudomonas aeruginosa group
NC_027992	RefSeq	RVG	G1029	39,552	62.5	Pseudomonas phage JBD25	Viruses; dsDNA viruses, no RNA stage; Caudovirales; Siphoviridae.	1225792	Bacteria; Proteobacteria; Gammaproteobacteria; Pseudomonadales; Pseudomonadaceae
NC_029107	RefSeq	RVG	G1030	41,941	65.1	Ralstonia phage RS138	Viruses; unclassified phages.	1483485	-
NC_005882	RefSeq	RVG	G1031	36,748	62.9	Burkholderia phage BcepMu	Viruses; dsDNA viruses, no RNA stage; Caudovirales; Myoviridae.	264729	Bacteria; Proteobacteria; Betaproteobacteria; Burkholderiales; Burkholderiaceae; Burkholderia; Burkholderia cepacia complex
NC_009237	RefSeq	RVG	G1031	37,446	63.1	Burkholderia phage phiE255	Viruses; dsDNA viruses, no RNA stage; Caudovirales; Myoviridae.	431894	Bacteria; Proteobacteria; Betaproteobacteria; Burkholderiales; Burkholderiaceae; Burkholderia; pseudomallei group
NC_019455	RefSeq	RVG	G1032	37,151	41.9	Haemophilus phage SuMu	Viruses; dsDNA viruses, no RNA stage; Caudovirales; Myoviridae; Mulikevirus; unclassified Mu-like viruses.	483266	Bacteria; Proteobacteria; Gammaproteobacteria; Pasteurellales; Pasteurellaceae; Haemophilus
NC_028766	RefSeq	RVG	G1032	33,755	43.1	Mannheimia phage vB_MhM_3927AP2	Viruses; dsDNA viruses, no RNA stage; Caudovirales; Myoviridae; Mulikevirus; unclassified Mu-like viruses.	1572750	-
NC_011216	RefSeq	RVG	G1033	37,635	62.9	Burkholderia phage KS10	Viruses; dsDNA viruses, no RNA stage; Caudovirales; Myoviridae.	557289	Bacteria; Proteobacteria; Betaproteobacteria; Burkholderiales; Burkholderiaceae; Burkholderia; Burkholderia cepacia complex

NC_013594	RefSeq	RVG	G1034	37,235	51.8	Escherichia phage D108	Viruses; dsDNA viruses, no RNA stage; Caudovirales; Myoviridae; Mulikevirus; unclassified Mu-like viruses.	665033	Bacteria; Proteobacteria; Gammaproteobacteria; Enterobacteriales; Enterobacteriaceae; Escherichia
NC_000929	RefSeq	RVG	G1034	36,717	52.1	Enterobacteria phage Mu	Viruses; dsDNA viruses, no RNA stage; Caudovirales; Myoviridae; Mulikevirus.	10677	Bacteria; Proteobacteria; Gammaproteobacteria; Enterobacteriales; Enterobacteriaceae; Escherichia
NC_027382	RefSeq	RVG	G1034	37,146	51.9	Enterobacteria phage SfMu	Viruses; dsDNA viruses, no RNA stage; Caudovirales; Myoviridae; Mulikevirus; unclassified Mu-like viruses.	1567022	Bacteria; Proteobacteria; Gammaproteobacteria; Enterobacteriales; Enterobacteriaceae; Shigella
NC_021070	RefSeq	RVG	G1034	33,277	45.8	Vibrio phage martha 12B12	Viruses; dsDNA viruses, no RNA stage; unclassified dsDNA phages.	573175	Bacteria; Proteobacteria; Gammaproteobacteria; Vibrionales; Vibrionaceae; Vibrio
KF302035	EBI	RVG	G1035	35,330	44.9	Pseudoalteromonas phage HS6	Viruses; unclassified phages.	1357710	-
NC_021557	RefSeq	RVG	G1036	37,378	58.3	Rhizobium phage RR1-B	Viruses; dsDNA viruses, no RNA stage; Caudovirales; Myoviridae.	929834	Bacteria; Proteobacteria; Alphaproteobacteria; Rhizobiales; Rhizobiaceae; Agrobacterium; Agrobacterium tumefaciens complex
NC_016165	RefSeq	RVG	G1036	39,283	64.9	Rhodobacter phage RcapMu	Viruses; dsDNA viruses, no RNA stage; Caudovirales; Siphoviridae.	1105286	Bacteria; Proteobacteria; Alphaproteobacteria; Rhodobacterales; Rhodobacteraceae; Rhodobacter
NC_020866	RefSeq	RVG	G1037	40,231	62.1	Rhodovulum phage RS1	Viruses; dsDNA viruses, no RNA stage; unclassified dsDNA phages.	754056	Bacteria; Proteobacteria; Alphaproteobacteria; Rhodobacterales; Rhodobacteraceae; Rhodovulum
NC_020839	RefSeq	RVG	G1037	39,573	62.3	Rhodobacter phage RC1	Viruses; dsDNA viruses, no RNA stage; Caudovirales; Siphoviridae.	754055	Bacteria; Proteobacteria; Alphaproteobacteria; Rhodobacterales; Rhodobacteraceae; Rhodobacter
KC139515	EBI	RVG	G1038	59,245	56.5	Salmonella phage FSL SP-124	Viruses; dsDNA viruses, no RNA stage; Caudovirales; Siphoviridae; Chilikevirus.	1173758	-
NC_021780	RefSeq	RVG	G1038	59,454	56.4	Salmonella phage FSLSP088	Viruses; dsDNA viruses, no RNA stage; Caudovirales; Siphoviridae; Chilikevirus.	1173757	Bacteria; Proteobacteria; Gammaproteobacteria; Enterobacteriales; Enterobacteriaceae; Salmonella; Salmonella enterica
NC_029045	RefSeq	RVG	G1038	60,216	56.5	Salmonella phage 37	Viruses; dsDNA viruses, no RNA stage; Caudovirales; Siphoviridae.	1654890	-
NC_019417	RefSeq	RVG	G1038	59,203	56.5	Salmonella phage SPN19	Viruses; dsDNA viruses, no RNA stage; Caudovirales; Siphoviridae; Chilikevirus.	1141134	Bacteria; Proteobacteria; Gammaproteobacteria; Enterobacteriales; Enterobacteriaceae; Salmonella
NC_021783	RefSeq	RVG	G1038	59,254	56.3	Salmonella phage iEPS5	Viruses; dsDNA viruses, no RNA stage; Caudovirales; Siphoviridae; Chilikevirus.	1005056	Bacteria; Proteobacteria; Gammaproteobacteria; Enterobacteriales; Enterobacteriaceae; Salmonella; Salmonella enterica
NC_025442	RefSeq	RVG	G1038	59,578	56.5	Salmonella phage Chi	Viruses; dsDNA viruses, no RNA stage; Caudovirales; Siphoviridae; Chilikevirus.	1541887	Bacteria; Proteobacteria; Gammaproteobacteria; Enterobacteriales; Enterobacteriaceae; Salmonella
KC139514	EBI	RVG	G1038	59,815	56.6	Salmonella phage FSL SP-039	Viruses; dsDNA viruses, no RNA stage; Caudovirales; Siphoviridae; Chilikevirus.	1173755	-
NC_021779	RefSeq	RVG	G1038	59,746	56.6	Salmonella phage FSLSP030	Viruses; dsDNA viruses, no RNA stage; Caudovirales; Siphoviridae; Chilikevirus.	1173754	Bacteria; Proteobacteria; Gammaproteobacteria; Enterobacteriales; Enterobacteriaceae; Salmonella; Salmonella enterica
NC_027991	RefSeq	RVG	G1038	147,303	45.8	Staphylococcus phage SA1	Viruses; dsDNA viruses, no RNA stage; Caudovirales; Myoviridae.	694060	Bacteria; Firmicutes; Bacilli; Bacillales; Staphylococcaceae; Staphylococcus
NC_019524	RefSeq	RVG	G1038	60,364	51.1	Enterobacter phage Enc34	Viruses; dsDNA viruses, no RNA stage; Caudovirales; Siphoviridae.	1150990	Bacteria; Proteobacteria; Gammaproteobacteria; Enterobacteriales; Enterobacteriaceae; Enterobacter; Enterobacter cloacae complex
NC_018832	RefSeq	RVG	G1038	58,104	49.5	Providencia phage Redjac	Viruses; dsDNA viruses, no RNA stage; Caudovirales.	1235559	Bacteria; Proteobacteria; Gammaproteobacteria; Enterobacteriales; Enterobacteriaceae; Providencia
NC_028812	RefSeq	RVG	G1038	58,546	46.9	Proteus phage pPM_01	Viruses; dsDNA viruses, no RNA stage; Caudovirales; Siphoviridae.	1567485	-
NC_005091	RefSeq	RVG	G1039	57,455	60.6	Burkholderia phage BcepNazgul	Viruses; dsDNA viruses, no RNA stage; Caudovirales; Siphoviridae.	242861	Bacteria; Proteobacteria; Betaproteobacteria; Burkholderiales; Burkholderiaceae; Burkholderia; Burkholderia cepacia complex
NC_018283	RefSeq	RVG	G1039	58,065	61.3	Burkholderia phage AH2	Viruses; dsDNA viruses, no RNA stage; Caudovirales; Siphoviridae.	1133022	Bacteria; Proteobacteria; Betaproteobacteria; Burkholderiales; Burkholderiaceae; Burkholderia; Burkholderia cepacia complex
NC_029106	RefSeq	RVG	G1040	62,220	60.1	Achromobacter phage phiAxp-2	Viruses; dsDNA viruses, no RNA stage; Caudovirales; Siphoviridae.	1664246	-
KF626665	EBI	RVG	G1041	56,147	62.4	Xylella phage Sano	Viruses; dsDNA viruses, no RNA stage; Caudovirales; Siphoviridae.	1415148	-
KF626668	EBI	RVG	G1041	55,601	63	Xylella phage Salvo	Viruses; dsDNA viruses, no RNA stage; Caudovirales; Siphoviridae.	1415147	-
NC_020853	RefSeq	RVG	G1042	56,958	55	Loktanella phage pCB2051-A	Viruses; dsDNA viruses, no RNA stage; unclassified dsDNA phages.	754044	Bacteria; Proteobacteria; Alphaproteobacteria; Rhodobacterales; Rhodobacteraceae; Loktanella
NC_025428	RefSeq	RVG	G1043	59,601	64.1	Ruegeria phage DSS3-P1	Viruses; dsDNA viruses, no RNA stage; Caudovirales; Siphoviridae.	1555208	Bacteria; Proteobacteria; Alphaproteobacteria; Rhodobacterales; Rhodobacteraceae; Ruegeria
AY526908	EBI	RVG	G1044	42,663	65.4	Bordetella phage BMP-1	Viruses; dsDNA viruses, no RNA stage; Caudovirales; Podoviridae.	263374	-
NC_005357	RefSeq	RVG	G1044	42,493	65.4	Bordetella phage BPP-1	Viruses; dsDNA viruses, no RNA stage; Caudovirales; Podoviridae.	194699	Bacteria; Proteobacteria; Betaproteobacteria; Burkholderiales; Alcaligenaceae; Bordetella
AY526909	EBI	RVG	G1044	42,638	65.4	Bordetella phage BIP-1	Viruses; dsDNA viruses, no RNA stage; Caudovirales; Podoviridae.	263375	-
NC_005887	RefSeq	RVG	G1045	42,415	65.2	Burkholderia phage BcepC6B	Viruses; dsDNA viruses, no RNA stage; Caudovirales; Podoviridae.	279280	Bacteria; Proteobacteria; Betaproteobacteria; Burkholderiales; Burkholderiaceae; Burkholderia; Burkholderia cepacia complex
NC_013599	RefSeq	RVG	G1046	36,674	56.8	Xylella phage Xfas53	Viruses; dsDNA viruses, no RNA stage; Caudovirales; Podoviridae.	670252	Bacteria; Proteobacteria; Gammaproteobacteria; Xanthomonadales; Xanthomonadaceae; Xylella
JF773396	EBI	RVG	G1047	38,552	39.3	Liberibacter phage FP2	Viruses; dsDNA viruses, no RNA stage; Caudovirales; Podoviridae.	1072683	-
NC_019550	RefSeq	RVG	G1047	38,997	39.4	Liberibacter phage SC2	Viruses; dsDNA viruses, no RNA stage; Caudovirales; Podoviridae.	941970	Bacteria; Proteobacteria; Alphaproteobacteria; Rhizobiales; Rhizobiaceae; Candidatus Liberibacter
NC_019549	RefSeq	RVG	G1047	40,048	41.2	Liberibacter phage SC1	Viruses; dsDNA viruses, no RNA stage; Caudovirales; Podoviridae.	941969	Bacteria; Proteobacteria; Alphaproteobacteria; Rhizobiales; Rhizobiaceae; Candidatus Liberibacter
TARA_ERS490120_N000412	TOV	EVG	G1048	40,893	60.3	-	-	-	-
TARA_ERS488613_N000232	TOV	EVG	G1048	41,317	60.2	-	-	-	-
TARA_ERS489943_N000302	TOV	EVG	G1049	46,866	52.6	-	-	-	-
NC_004927	RefSeq	RVG	G1050	75,898	55.8	Halovirus HF1	Viruses; dsDNA viruses, no RNA stage; unclassified dsDNA viruses; unclassified archaeal dsDNA viruses; Haloviruses.	222645	Archaea; Euryarchaeota; Halobacteria; Haloferacales; Haloferacaceae; Haloferax
NC_003345	RefSeq	RVG	G1050	77,670	55.8	Halorubrum phage HF2	Viruses; dsDNA viruses, no RNA stage; unclassified dsDNA viruses; unclassified archaeal dsDNA viruses; Haloviruses.	33771	Archaea; Euryarchaeota; Halobacteria; Haloferacales; Haloferacaceae; Halorubrum
NC_021320	RefSeq	RVG	G1050	76,134	56.4	Halovirus HRTV-5	Viruses; dsDNA viruses, no RNA stage; unclassified dsDNA viruses; unclassified archaeal dsDNA viruses; Haloviruses.	1273753	Archaea; Euryarchaeota; Halobacteria; Haloferacales; Haloferacaceae; Halorubrum
NC_021321	RefSeq	RVG	G1050	74,519	57.1	Halovirus HRTV-8	Viruses; dsDNA viruses, no RNA stage; unclassified dsDNA viruses; unclassified archaeal dsDNA viruses; Haloviruses.	1273755	Archaea; Euryarchaeota; Halobacteria; Haloferacales; Haloferacaceae; Halorubrum
NC_021335	RefSeq	RVG	G1050	69,048	59.6	Halovirus HRTV-7	Viruses; dsDNA viruses, no RNA stage; unclassified dsDNA viruses; unclassified archaeal dsDNA viruses; Haloviruses.	1273754	Archaea; Euryarchaeota; Halobacteria; Haloferacales; Haloferacaceae; Halorubrum

NC_020159	RefSeq	RVG	G1050	68,527	60.3	Halovirus HSTV-2	Viruses; dsDNA viruses, no RNA stage; unclassified dsDNA viruses; unclassified archaeal dsDNA viruses; Haloviruses.	1262527	Archaea; Euryarchaeota; Halobacteria; Haloferacales; Haloferacaceae; Halorubrum
NC_021328	RefSeq	RVG	G1051	143,855	50.4	Halovirus HGTV-1	Viruses; dsDNA viruses, no RNA stage; unclassified dsDNA viruses; unclassified archaeal dsDNA viruses; Haloviruses.	1273749	Archaea; Euryarchaeota; Halobacteria; Haloferacales; Haloferacaceae; Halogranum
NC_021327	RefSeq	RVG	G1052	102,105	57.6	Halovirus HCTV-5	Viruses; dsDNA viruses, no RNA stage; unclassified dsDNA viruses; unclassified archaeal dsDNA viruses; Haloviruses.	1273748	Archaea; Euryarchaeota; Halobacteria; Halobacteriales; Halobacteriaceae; Haloarcula
NC_020158	RefSeq	RVG	G1052	102,319	58.3	Halovirus HVTV-1	Viruses; dsDNA viruses, no RNA stage; unclassified dsDNA viruses; unclassified archaeal dsDNA viruses; Haloviruses.	1262528	Archaea; Euryarchaeota; Halobacteria; Halobacteriales; Halobacteriaceae; Haloarcula
NC_021330	RefSeq	RVG	G1052	103,257	57	Halovirus HCTV-1	Viruses; dsDNA viruses, no RNA stage; unclassified dsDNA viruses; unclassified archaeal dsDNA viruses; Haloviruses.	1273746	Archaea; Euryarchaeota; Halobacteria; Halobacteriales; Halobacteriaceae; Haloarcula
NC_021340	RefSeq	RVG	G1053	52,643	66.6	Halovirus HHTV-2	Viruses; dsDNA viruses, no RNA stage; unclassified dsDNA viruses; unclassified archaeal dsDNA viruses; Haloviruses.	1273751	Archaea; Euryarchaeota; Halobacteria; Halobacteriales; Halobacteriaceae; Haloarcula
NC_021319	RefSeq	RVG	G1053	54,291	68.1	Halovirus HCTV-2	Viruses; dsDNA viruses, no RNA stage; unclassified dsDNA viruses; unclassified archaeal dsDNA viruses; Haloviruses.	1273747	Archaea; Euryarchaeota; Halobacteria; Halobacteriales; Halobacteriaceae; Haloarcula
NC_021537	RefSeq	RVG	G1054	39,784	65.1	Halorubrum phage CGphi46	Viruses; dsDNA viruses, no RNA stage; unclassified dsDNA phages.	754066	Archaea; Euryarchaeota; Halobacteria; Haloferacales; Haloferacaceae
NC_008695	RefSeq	RVG	G1054	42,271	64.9	Archaeal BJ1 virus	Viruses; dsDNA viruses, no RNA stage; Caudovirales; Siphoviridae.	416419	Archaea; Euryarchaeota; Halobacteria; Haloferacales; Haloferacaceae
NC_004084	RefSeq	RVG	G1055	58,498	61.9	Natrialba phage PhiCh1	Viruses; dsDNA viruses, no RNA stage; Caudovirales; Myoviridae.	114777	Archaea; Euryarchaeota; Halobacteria; Natrialbales; Natrialbaceae; Natrialba
NC_021471	RefSeq	RVG	G1056	32,189	60.3	Halovirus HSTV-1	Viruses; dsDNA viruses, no RNA stage; unclassified dsDNA viruses; unclassified archaeal dsDNA viruses; Haloviruses.	1262530	Archaea; Euryarchaeota; Halobacteria; Halobacteriales; Halobacteriaceae; Haloarcula
NC_021329	RefSeq	RVG	G1057	35,722	59.5	Halovirus HRTV-4	Viruses; dsDNA viruses, no RNA stage; unclassified dsDNA viruses; unclassified archaeal dsDNA viruses; Haloviruses.	1273752	Archaea; Euryarchaeota; Halobacteria; Haloferacales; Haloferacaceae; Halorubrum
NC_025213	RefSeq	RVG	G1058	10,021	49.1	Halovirus VNH-1	Viruses; unclassified viruses.	1500510	Archaea; Nanohaloarchaeota; Nanohaloarchaea
JF974305	CAMERA	RVG	G1059	82,365	53.3	Halorubrum phage GNf2	Viruses; dsDNA viruses, no RNA stage; unclassified dsDNA phages.	889959	-
NC_013698	RefSeq	RVG	G1060	58,652	57	Clavibacter phage CMP1	Viruses; dsDNA viruses, no RNA stage; Caudovirales; Siphoviridae.	686439	Bacteria; Actinobacteria; Actinobacteria; Micrococcales; Microbacteriaceae; Clavibacter; Clavibacter michiganensis
NC_023549	RefSeq	RVG	G1061	56,789	62.1	Clavibacter phage CN1A	Viruses; dsDNA viruses, no RNA stage; Caudovirales; Siphoviridae.	1406793	Bacteria; Actinobacteria; Actinobacteria; Micrococcales; Microbacteriaceae; Clavibacter
TARA_ERS489113_N000128	TOV	EVG	G1062	58,398	56.2	-	-	-	-
NC_023859	RefSeq	RVG	G1063	59,254	62.8	Microbacterium phage vB_MoxS-ISF9	Viruses; dsDNA viruses, no RNA stage; Caudovirales; Siphoviridae.	1458670	Bacteria; Actinobacteria; Actinobacteria; Micrococcales; Microbacteriaceae; Microbacterium
TARA_ERS478052_N000200	TOV	EVG	G1064	47,218	62	-	-	-	-
NC_004745	RefSeq	RVG	G1065	30,728	52.1	Yersinia phage L-413C	Viruses; dsDNA viruses, no RNA stage; Caudovirales; Myoviridae; Peduovirinae; P2likevirus.	227940	Bacteria; Proteobacteria; Gammaproteobacteria; Enterobacteriales; Enterobacteriaceae; Yersinia; Yersinia pseudotuberculosis complex
NC_005056	RefSeq	RVG	G1065	32,684	51.7	Enterobacteria phage WPhi	Viruses; dsDNA viruses, no RNA stage; Caudovirales; Myoviridae; Peduovirinae; P2likevirus.	103216	-
NC_022750	RefSeq	RVG	G1065	33,628	51.9	Enterobacteria phage fiAA91-ss	Viruses; dsDNA viruses, no RNA stage; Caudovirales; Myoviridae; Peduovirinae; P2likevirus.	1357825	Bacteria; Proteobacteria; Gammaproteobacteria; Enterobacteriales; Enterobacteriaceae; Escherichia
NC_028943	RefSeq	RVG	G1065	29,237	53	Escherichia phage pro483	Viruses; dsDNA viruses, no RNA stage; Caudovirales; Myoviridae; Peduovirinae; P2likevirus.	1649240	-
NC_028896	RefSeq	RVG	G1065	32,675	50.7	Escherichia phage pro147	Viruses; dsDNA viruses, no RNA stage; Caudovirales; Myoviridae; Peduovirinae; P2likevirus.	1649239	-
NC_001895	RefSeq	RVG	G1065	33,593	50.2	Enterobacteria phage P2	Viruses; dsDNA viruses, no RNA stage; Caudovirales; Myoviridae; Peduovirinae; P2likevirus.	10679	Bacteria; Proteobacteria; Gammaproteobacteria; Enterobacteriales; Enterobacteriaceae; Escherichia
NC_005340	RefSeq	RVG	G1065	30,636	52.8	Enterobacteria phage PsP3	Viruses; dsDNA viruses, no RNA stage; Caudovirales; Myoviridae; Peduovirinae; P2likevirus.	12407	Bacteria; Proteobacteria; Gammaproteobacteria; Enterobacteriales; Enterobacteriaceae
NC_029003	RefSeq	RVG	G1065	29,732	53	Salmonella phage SEN1	Viruses; dsDNA viruses, no RNA stage; Caudovirales; Myoviridae; Peduovirinae; P2likevirus.	1647455	-
NC_001317	RefSeq	RVG	G1065	30,624	53.1	Enterobacteria phage 186	Viruses; dsDNA viruses, no RNA stage; Caudovirales; Myoviridae; Peduovirinae; P2likevirus.	29252	-
NC_021774	RefSeq	RVG	G1065	29,742	52.8	Salmonella phage FSL SP-004	Viruses; dsDNA viruses, no RNA stage; Caudovirales; Myoviridae; Peduovirinae; P2likevirus.	1173769	Bacteria; Proteobacteria; Gammaproteobacteria; Enterobacteriales; Enterobacteriaceae; Salmonella; Salmonella enterica
NC_019932	RefSeq	RVG	G1065	29,564	55.8	Erwinia phage ENT90	Viruses; dsDNA viruses, no RNA stage; Caudovirales; Myoviridae.	947843	Bacteria; Proteobacteria; Gammaproteobacteria; Enterobacteriales; Enterobacteriaceae; Erwinia
NC_019488	RefSeq	RVG	G1065	34,117	51	Salmonella phage RE-2010	Viruses; dsDNA viruses, no RNA stage; Caudovirales; Myoviridae.	929814	Bacteria; Proteobacteria; Gammaproteobacteria; Enterobacteriales; Enterobacteriaceae; Salmonella; Salmonella enterica
NC_010463	RefSeq	RVG	G1065	33,693	52.5	Salmonella phage Fels-2	Viruses; dsDNA viruses, no RNA stage; Caudovirales; Myoviridae; Peduovirinae; P2likevirus.	194701	Bacteria; Proteobacteria; Gammaproteobacteria; Enterobacteriales; Enterobacteriaceae; Salmonella; Salmonella enterica
NC_029015	RefSeq	RVG	G1065	33,507	53.4	Salmonella phage SEN4	Viruses; dsDNA viruses, no RNA stage; Caudovirales; Myoviridae; Peduovirinae; P2likevirus.	1647465	-
NC_028701	RefSeq	RVG	G1065	33,508	53.4	Salmonella phage SEN5	Viruses; dsDNA viruses, no RNA stage; Caudovirales; Myoviridae.	1647466	-
NC_026014	RefSeq	RVG	G1065	35,814	52.9	Enterobacteria phage P88	Viruses; dsDNA viruses, no RNA stage; Caudovirales; Myoviridae.	1567486	Bacteria; Proteobacteria; Gammaproteobacteria; Enterobacteriales; Enterobacteriaceae; Escherichia
JX681814	EBI	RVG	G1065	37,637	64.8	Burkholderia phage phiX216	Viruses; dsDNA viruses, no RNA stage; Caudovirales; Myoviridae; Peduovirinae; P2likevirus.	1235712	-
CP008753	EBI	RVG	G1065	37,631	64.8	Burkholderia phage BEK	Viruses; dsDNA viruses, no RNA stage; Caudovirales; Myoviridae; Peduovirinae; P2likevirus.	1514988	-
NC_007145	RefSeq	RVG	G1065	37,639	64.8	Burkholderia phage phi52237	Viruses; dsDNA viruses, no RNA stage; Caudovirales; Myoviridae; Peduovirinae; P2likevirus.	332032	Bacteria; Proteobacteria; Betaproteobacteria; Burkholderiales; Burkholderiaceae; Burkholderia; pseudomallei group
NC_009234	RefSeq	RVG	G1065	35,741	65.4	Burkholderia phage phiE202	Viruses; dsDNA viruses, no RNA stage; Caudovirales; Myoviridae; Peduovirinae; P2likevirus.	431893	Bacteria; Proteobacteria; Betaproteobacteria; Burkholderiales; Burkholderiaceae; Burkholderia; pseudomallei group
NC_009236	RefSeq	RVG	G1065	36,690	64.6	Burkholderia phage phiE12-2	Viruses; dsDNA viruses, no RNA stage; Caudovirales; Myoviridae; Peduovirinae; P2likevirus.	431892	Bacteria; Proteobacteria; Betaproteobacteria; Burkholderiales; Burkholderiaceae; Burkholderia; pseudomallei group
NC_015266	RefSeq	RVG	G1065	40,555	63.2	Burkholderia phage KL3	Viruses; dsDNA viruses, no RNA stage; Caudovirales; Myoviridae; Peduovirinae; P2likevirus.	910474	Bacteria; Proteobacteria; Betaproteobacteria; Burkholderiales; Burkholderiaceae; Burkholderia; Burkholderia cepacia complex

NC_015273	RefSeq	RVG	G1065	32,317	62.3	Burkholderia phage KS14	Viruses; dsDNA viruses, no RNA stage; Caudovirales; Myoviridae; Peduovirinae; P2likevirus.	910475	Bacteria; Proteobacteria; Betaproteobacteria; Burkholderiales; Burkholderiaceae; Burkholderia; Burkholderia cepacia complex
NC_015265	RefSeq	RVG	G1065	37,236	63.7	Burkholderia phage KS5	Viruses; dsDNA viruses, no RNA stage; Caudovirales; Myoviridae; Peduovirinae; P2likevirus.	910473	Bacteria; Proteobacteria; Betaproteobacteria; Burkholderiales; Burkholderiaceae; Burkholderia; Burkholderia cepacia complex
NC_009382	RefSeq	RVG	G1065	38,760	65.3	Ralstonia phage RSA1	Viruses; dsDNA viruses, no RNA stage; Caudovirales; Myoviridae; Peduovirinae; P2likevirus.	406340	Bacteria; Proteobacteria; Betaproteobacteria; Burkholderiales; Burkholderiaceae; Ralstonia
NC_025115	RefSeq	RVG	G1065	40,002	64.8	Ralstonia phage RSY1	Viruses; dsDNA viruses, no RNA stage; Caudovirales; Myoviridae; Peduovirinae; P2likevirus.	1530085	Bacteria; Proteobacteria; Betaproteobacteria; Burkholderiales; Burkholderiaceae; Ralstonia
NC_021343	RefSeq	RVG	G1065	35,430	62.5	Burkholderia phage ST79	Viruses; dsDNA viruses, no RNA stage; Caudovirales; Myoviridae.	1282994	Bacteria; Proteobacteria; Betaproteobacteria; Burkholderiales; Burkholderiaceae; Burkholderia; pseudomallei group
NC_003278	RefSeq	RVG	G1065	35,580	62.6	Pseudomonas phage phiCTX	Viruses; dsDNA viruses, no RNA stage; Caudovirales; Myoviridae; Peduovirinae; P2likevirus.	35343	Bacteria; Proteobacteria; Gammaproteobacteria; Pseudomonadales; Pseudomonadaceae; Pseudomonas; Pseudomonas aeruginosa group
NC_023588	RefSeq	RVG	G1065	33,525	65	Stenotrophomonas phage Smp131	Viruses; dsDNA viruses, no RNA stage; Caudovirales; Myoviridae; Peduovirinae; P2likevirus.	1168563	Bacteria; Proteobacteria; Gammaproteobacteria; Xanthomonadales; Xanthomonadaceae; Stenotrophomonas
JN255163	EBI	RVG	G1065	34,719	41.6	Mannheimia phage vB_MhM_1152AP	Viruses; dsDNA viruses, no RNA stage; Caudovirales; Myoviridae; Peduovirinae; P2likevirus.	1182515	-
DQ426905	EBI	RVG	G1065	34,600	41.6	Mannheimia phage phiMhaA1-BAA410	Viruses; dsDNA viruses, no RNA stage; Caudovirales; Myoviridae; Peduovirinae; P2likevirus.	376821	-
DQ426904	EBI	RVG	G1065	34,525	41.6	Mannheimia phage phiMhaA1-PHL101	Viruses; dsDNA viruses, no RNA stage; Caudovirales; Myoviridae; Peduovirinae; P2likevirus.	376820	-
NC_008201	RefSeq	RVG	G1065	34,525	41.6	Mannheimia phage phiMHaA1	Viruses; dsDNA viruses, no RNA stage; Caudovirales; Myoviridae; Peduovirinae; P2likevirus.	390978	Bacteria; Proteobacteria; Gammaproteobacteria; Pasteurellales; Pasteurellaceae; Mannheimia
NC_028898	RefSeq	RVG	G1065	35,764	42.1	Mannheimia phage vB_MhM_587AP1	Viruses; dsDNA viruses, no RNA stage; Caudovirales; Myoviridae; Peduovirinae; P2likevirus.	1572744	-
NC_003313	RefSeq	RVG	G1066	33,106	48.9	Vibrio phage K139	Viruses; dsDNA viruses, no RNA stage; Caudovirales; Myoviridae; Peduovirinae; Hpunalikevirus.	70734	Bacteria; Proteobacteria; Gammaproteobacteria; Vibrionales; Vibrionaceae; Vibrio
NC_022747	RefSeq	RVG	G1066	34,145	48.8	Vibrio phage VPUSM 8	Viruses; dsDNA viruses, no RNA stage; Caudovirales; Myoviridae.	1341101	Bacteria; Proteobacteria; Gammaproteobacteria; Vibrionales; Vibrionaceae; Vibrio
AB374228	EBI	RVG	G1066	33,507	48.8	Vibrio phage kappa	Viruses; dsDNA viruses, no RNA stage; Caudovirales; Myoviridae; Peduovirinae; Hpunalikevirus.	493906	-
JF974301	CAMERA	RVG	G1066	81,013	44.2	Vibrio phage VD1	Viruses; dsDNA viruses, no RNA stage; unclassified dsDNA phages.	754057	-
NC_027368	RefSeq	RVG	G1066	33,828	48.2	Vibrio phage PV94	Viruses; dsDNA viruses, no RNA stage; Caudovirales; Myoviridae.	1451050	Bacteria; Proteobacteria; Gammaproteobacteria; Vibrionales; Vibrionaceae; Vibrio
HQ110083	EBI	RVG	G1066	28,765	55.2	Cronobacter phage ESSI-2	Viruses; unclassified phages.	947842	-
NC_009542	RefSeq	RVG	G1066	33,985	61.7	Aeromonas phage phiO18P	Viruses; dsDNA viruses, no RNA stage; Caudovirales; Myoviridae; Peduovirinae; Hpunalikevirus.	393598	Bacteria; Proteobacteria; Gammaproteobacteria; Aeromonadales; Aeromonadaceae; Aeromonas
NC_001697	RefSeq	RVG	G1066	32,355	40	Haemophilus phage HP1	Viruses; dsDNA viruses, no RNA stage; Caudovirales; Myoviridae; Peduovirinae; Hpunalikevirus.	10690	Bacteria; Proteobacteria; Gammaproteobacteria; Pasteurellales; Pasteurellaceae; Haemophilus
NC_003315	RefSeq	RVG	G1066	31,508	39.9	Haemophilus phage HP2	Viruses; dsDNA viruses, no RNA stage; Caudovirales; Myoviridae; Peduovirinae; Hpunalikevirus.	157239	Bacteria; Proteobacteria; Gammaproteobacteria; Pasteurellales; Pasteurellaceae; Haemophilus
NC_008193	RefSeq	RVG	G1066	30,505	42.1	Pasteurella phage F108	Viruses; dsDNA viruses, no RNA stage; Caudovirales; Myoviridae; Peduovirinae; Hpunalikevirus.	342564	Bacteria; Proteobacteria; Gammaproteobacteria; Pasteurellales; Pasteurellaceae; Pasteurella
NC_000867	RefSeq	RVG	G1067	10,079	42.2	Pseudoalteromonas phage PM2	Viruses; dsDNA viruses, no RNA stage; Corticoviridae; Corticovirus.	10661	Bacteria; Proteobacteria; Gammaproteobacteria; Alteromonadales; Pseudoalteromonadaceae
NC_004456	RefSeq	RVG	G1068	43,198	50.6	Vibrio phage VHML	Viruses; dsDNA viruses, no RNA stage; Caudovirales; Myoviridae; VHML-like phages.	207597	Bacteria; Proteobacteria; Gammaproteobacteria; Vibrionales; Vibrionaceae; Vibrio; Vibrio harveyi group
NC_027981	RefSeq	RVG	G1068	42,612	50.9	Vibrio phage VP58.5	Viruses; dsDNA viruses, no RNA stage; Caudovirales; Myoviridae; VHML-like phages.	631719	Bacteria; Proteobacteria; Gammaproteobacteria; Vibrionales; Vibrionaceae; Vibrio; Vibrio harveyi group
NC_019722	RefSeq	RVG	G1068	41,351	51.3	Vibrio phage vB_VpaM_MAR	Viruses; dsDNA viruses, no RNA stage; Caudovirales; Myoviridae.	1229754	Bacteria; Proteobacteria; Gammaproteobacteria; Vibrionales; Vibrionaceae; Vibrio; Vibrio harveyi group
NC_010342	RefSeq	RVG	G1069	39,245	59	Halomonas phage phiHAP-1	Viruses; dsDNA viruses, no RNA stage; Caudovirales; Myoviridae.	499235	Bacteria; Proteobacteria; Gammaproteobacteria; Oceanospirillales; Halomonadaceae; Halomonas
NC_009016	RefSeq	RVG	G1069	38,197	57	Vibrio phage VP882	Viruses; dsDNA viruses, no RNA stage; Caudovirales; Myoviridae.	261726	Bacteria; Proteobacteria; Gammaproteobacteria; Vibrionales; Vibrionaceae; Vibrio; Vibrio harveyi group
NC_027995	RefSeq	RVG	G1070	41,666	53.4	Escherichia phage vB_EcoM_ECO1230-10	Viruses; dsDNA viruses, no RNA stage; Caudovirales; Myoviridae.	669875	Bacteria; Proteobacteria; Gammaproteobacteria; Enterobacteriales; Enterobacteriaceae; Escherichia
NC_025430	RefSeq	RVG	G1070	42,351	53.3	Escherichia phage vB_EcoM-ep3	Viruses; dsDNA viruses, no RNA stage; Caudovirales; Myoviridae.	1541883	Bacteria; Proteobacteria; Gammaproteobacteria; Enterobacteriales; Enterobacteriaceae; Escherichia
NC_023006	RefSeq	RVG	G1070	43,564	61.1	Pseudomonas phage PPpW-3	Viruses; dsDNA viruses, no RNA stage; Caudovirales; Myoviridae.	1279082	Bacteria; Proteobacteria; Gammaproteobacteria; Pseudomonadales; Pseudomonadaceae; Pseudomonas; Pseudomonas putida group
JX507079	EBI	RVG	G1071	59,363	65.5	Acidithiobacillus phage AcaML1	Viruses; dsDNA viruses, no RNA stage; Caudovirales; Myoviridae.	1229761	-
TARA_ERS478052_N000430	TOV	EVG	G1072	32,064	32.8	-	-	-	-
TARA_ERS490142_N000511	TOV	EVG	G1072	32,857	39.3	-	-	-	-
TARA_ERS489148_N000845	TOV	EVG	G1073	20,291	45.9	-	-	-	-
NC_016164	RefSeq	RVG	G1074	30,332	58.7	Synechococcus phage S-CBS1	Viruses; dsDNA viruses, no RNA stage; Caudovirales; Siphoviridae.	909297	Bacteria; Cyanobacteria; Oscillatoriophycideae; Chroococcales; Synechococcus
NC_015465	RefSeq	RVG	G1074	33,004	60.7	Synechococcus phage S-CBS3	Viruses; dsDNA viruses, no RNA stage; Caudovirales; Siphoviridae.	753085	Bacteria; Cyanobacteria; Oscillatoriophycideae; Chroococcales; Synechococcus
KP296794	EBI	RVG	G1075	37,971	41.8	Bacteriophage Redbud	Viruses; dsDNA viruses, no RNA stage; Caudovirales; Siphoviridae.	1589753	-
NC_029084	RefSeq	RVG	G1075	37,990	41.8	Bacteriophage Rani	Viruses; dsDNA viruses, no RNA stage; Caudovirales; Siphoviridae.	1589752	-
NC_028758	RefSeq	RVG	G1075	35,644	41.8	Paenibacillus phage HB10c2	Viruses; dsDNA viruses, no RNA stage; Caudovirales; Siphoviridae.	1589749	-
NC_028854	RefSeq	RVG	G1075	43,724	41.6	Bacteriophage Sitara	Viruses; dsDNA viruses, no RNA stage; Caudovirales; Siphoviridae.	1589755	-
KP296795	EBI	RVG	G1075	41,152	41.5	Bacteriophage Shelly	Viruses; dsDNA viruses, no RNA stage; Caudovirales; Siphoviridae.	1589754	-
NC_028837	RefSeq	RVG	G1075	41,149	41.5	Paenibacillus phage Xenia	Viruses; dsDNA viruses, no RNA stage; Caudovirales; Siphoviridae.	1636263	-
NC_028788	RefSeq	RVG	G1075	37,246	42.1	Bacteriophage Diva	Viruses; dsDNA viruses, no RNA stage; Caudovirales; Siphoviridae.	1589750	-
NC_028851	RefSeq	RVG	G1075	37,995	41.9	Paenibacillus phage Fern	Viruses; dsDNA viruses, no RNA stage; Caudovirales; Siphoviridae.	1636255	-
NC_021865	RefSeq	RVG	G1075	41,294	40.9	Paenibacillus phage phiIBB_Pl23	Viruses; dsDNA viruses, no RNA stage; Caudovirales.	1337877	Bacteria; Firmicutes; Bacilli; Bacillales; Paenibacillaceae; Paenibacillus
NC_028746	RefSeq	RVG	G1075	44,249	40.2	Paenibacillus phage Harrison	Viruses; dsDNA viruses, no RNA stage; Caudovirales; Siphoviridae.	1636257	-

NC_028767	RefSeq	RVG	G1075	45,653	43.6	Paenibacillus phage Vegas	Viruses; dsDNA viruses, no RNA stage; Caudovirales; Siphoviridae.	1636261	-
NC_028841	RefSeq	RVG	G1075	44,952	42.7	Bacteriophage Lily	Viruses; dsDNA viruses, no RNA stage; Caudovirales; Siphoviridae.	1589751	-
KJ545483	EBI	RVG	G1076	41,476	46.1	Vibrio phage phi 2	Viruses; dsDNA viruses, no RNA stage; Caudovirales; Myoviridae.	1476902	-
NC_024369	RefSeq	RVG	G1076	41,569	46.1	Vibrio phage X29	Viruses; dsDNA viruses, no RNA stage; Caudovirales; Myoviridae.	1500713	Bacteria; Proteobacteria; Gammaproteobacteria; Vibrionales; Vibrionaceae; Vibrio
KR131710	EBI	RVG	G1077	39,921	27	Fusobacterium phage Funu1	Viruses; unclassified phages.	1640977	-
TARA_ERS490346_N000374	TOV	EVG	G1078	39,448	58.3	-	-	-	-
TARA_ERS488499_N000223	TOV	EVG	G1078	39,448	58.3	-	-	-	-
TARA_ERS488701_N000194	TOV	EVG	G1078	39,448	58.3	-	-	-	-
NC_007805	RefSeq	RVG	G1079	39,199	62.1	Pseudomonas phage F10	Viruses; dsDNA viruses, no RNA stage; Caudovirales; Siphoviridae.	347324	Bacteria; Proteobacteria; Gammaproteobacteria; Pseudomonadales; Pseudomonadaceae; Pseudomonas; Pseudomonas aeruginosa group
X51522	EBI	RVG	G1080	11,624	49.5	Enterobacteria phage P4	Viruses; dsDNA viruses, no RNA stage; Caudovirales; Myoviridae; Peduovirinae; P2likevirus.	10680	-
NC_011589	RefSeq	RVG	G1081	40,287	63.7	Stenotrophomonas phage S1	Viruses; dsDNA viruses, no RNA stage; Caudovirales; Siphoviridae.	573591	Bacteria; Proteobacteria; Gammaproteobacteria; Xanthomonadales; Xanthomonadaceae; Stenotrophomonas
KJ578792	EBI	RVG	G1082	29,442	54.2	Propionibacterium phage PHL308M00	Viruses; dsDNA viruses, no RNA stage; Caudovirales; Siphoviridae.	1500832	-
NC_027294	RefSeq	RVG	G1082	29,438	54.2	Propionibacterium phage PHL150M00	Viruses; dsDNA viruses, no RNA stage; Caudovirales; Siphoviridae.	1500822	Bacteria; Actinobacteria; Actinobacteria; Propionibacteriales; Propionibacteriaceae; Propionibacterium
NC_018842	RefSeq	RVG	G1082	29,348	54.4	Propionibacterium phage P1.1	Viruses; dsDNA viruses, no RNA stage; Caudovirales; Siphoviridae.	1229792	Bacteria; Actinobacteria; Actinobacteria; Propionibacteriales; Propionibacteriaceae; Propionibacterium
NC_022337	RefSeq	RVG	G1082	29,467	53.9	Propionibacterium phage PHL071N05	Viruses; dsDNA viruses, no RNA stage; Caudovirales; Siphoviridae.	1235650	Bacteria; Actinobacteria; Actinobacteria; Propionibacteriales; Propionibacteriaceae; Propionibacterium
NC_027357	RefSeq	RVG	G1082	29,496	54	Propionibacterium phage PHL025M00	Viruses; dsDNA viruses, no RNA stage; Caudovirales; Siphoviridae.	1500799	Bacteria; Actinobacteria; Actinobacteria; Propionibacteriales; Propionibacteriaceae; Propionibacterium
NC_018852	RefSeq	RVG	G1082	29,506	53.8	Propionibacterium phage P100D	Viruses; dsDNA viruses, no RNA stage; Caudovirales; Siphoviridae.	1229789	Bacteria; Actinobacteria; Actinobacteria; Propionibacteriales; Propionibacteriaceae; Propionibacterium
NC_018845	RefSeq	RVG	G1082	29,371	54	Propionibacterium phage P104A	Viruses; dsDNA viruses, no RNA stage; Caudovirales; Siphoviridae.	1229787	Bacteria; Actinobacteria; Actinobacteria; Propionibacteriales; Propionibacteriaceae; Propionibacterium
NC_027627	RefSeq	RVG	G1082	29,440	53.9	Propionibacterium phage Solid	Viruses; dsDNA viruses, no RNA stage; Caudovirales; Siphoviridae.	1655021	Bacteria; Actinobacteria; Actinobacteria; Propionibacteriales; Propionibacteriaceae; Propionibacterium
NC_027626	RefSeq	RVG	G1082	29,347	54	Propionibacterium phage Procrass1	Viruses; dsDNA viruses, no RNA stage; Caudovirales; Siphoviridae.	1655019	Bacteria; Actinobacteria; Actinobacteria; Propionibacteriales; Propionibacteriaceae; Propionibacterium
NC_015454	RefSeq	RVG	G1082	29,074	54.1	Propionibacterium phage PAD20	Viruses; dsDNA viruses, no RNA stage; Caudovirales; Siphoviridae.	504501	Bacteria; Actinobacteria; Actinobacteria; Propionibacteriales; Propionibacteriaceae; Propionibacterium
NC_022338	RefSeq	RVG	G1082	29,514	54	Propionibacterium phage PHL060L00	Viruses; dsDNA viruses, no RNA stage; Caudovirales; Siphoviridae.	1235647	Bacteria; Actinobacteria; Actinobacteria; Propionibacteriales; Propionibacteriaceae; Propionibacterium
NC_018841	RefSeq	RVG	G1082	29,574	54.1	Propionibacterium phage P101A	Viruses; dsDNA viruses, no RNA stage; Caudovirales; Siphoviridae.	1229786	Bacteria; Actinobacteria; Actinobacteria; Propionibacteriales; Propionibacteriaceae; Propionibacterium
NC_028967	RefSeq	RVG	G1082	29,605	54	Propionibacterium phage PAC1	Viruses; dsDNA viruses, no RNA stage; Caudovirales; Siphoviridae.	1690805	-
NC_027367	RefSeq	RVG	G1082	29,003	54.3	Propionibacterium phage PHL132N00	Viruses; dsDNA viruses, no RNA stage; Caudovirales; Siphoviridae.	1500820	Bacteria; Actinobacteria; Actinobacteria; Propionibacteriales; Propionibacteriaceae; Propionibacterium
KJ578766	EBI	RVG	G1082	29,386	54.3	Propionibacterium phage PHL067M09	Viruses; dsDNA viruses, no RNA stage; Caudovirales; Siphoviridae.	1500806	-
KJ578765	EBI	RVG	G1082	29,377	54.3	Propionibacterium phage PHL067M01	Viruses; dsDNA viruses, no RNA stage; Caudovirales; Siphoviridae.	1500805	-
NC_022335	RefSeq	RVG	G1082	29,377	54.3	Propionibacterium phage PHL067M10	Viruses; dsDNA viruses, no RNA stage; Caudovirales; Siphoviridae.	1235649	Bacteria; Actinobacteria; Actinobacteria; Propionibacteriales; Propionibacteriaceae; Propionibacterium
NC_027389	RefSeq	RVG	G1082	29,494	53.9	Propionibacterium phage PHL141N00	Viruses; dsDNA viruses, no RNA stage; Caudovirales; Siphoviridae.	1500821	Bacteria; Actinobacteria; Actinobacteria; Propionibacteriales; Propionibacteriaceae; Propionibacterium
NC_027370	RefSeq	RVG	G1082	29,428	53.9	Propionibacterium phage PHL179M00	Viruses; dsDNA viruses, no RNA stage; Caudovirales; Siphoviridae.	1500828	Bacteria; Actinobacteria; Actinobacteria; Propionibacteriales; Propionibacteriaceae; Propionibacterium
NC_018834	RefSeq	RVG	G1082	29,214	54.1	Propionibacterium phage P9.1	Viruses; dsDNA viruses, no RNA stage; Caudovirales; Siphoviridae.	1229782	Bacteria; Actinobacteria; Actinobacteria; Propionibacteriales; Propionibacteriaceae; Propionibacterium
NC_027401	RefSeq	RVG	G1082	29,751	54	Propionibacterium phage PHL095N00	Viruses; dsDNA viruses, no RNA stage; Caudovirales; Siphoviridae.	1500814	Bacteria; Actinobacteria; Actinobacteria; Propionibacteriales; Propionibacteriaceae; Propionibacterium
NC_018840	RefSeq	RVG	G1082	29,612	54.1	Propionibacterium phage P100_1	Viruses; dsDNA viruses, no RNA stage; Caudovirales; Siphoviridae.	1229791	Bacteria; Actinobacteria; Actinobacteria; Propionibacteriales; Propionibacteriaceae; Propionibacterium
NC_027336	RefSeq	RVG	G1082	29,503	53.9	Propionibacterium phage PHL009M11	Viruses; dsDNA viruses, no RNA stage; Caudovirales; Siphoviridae.	1500798	Bacteria; Actinobacteria; Actinobacteria; Propionibacteriales; Propionibacteriaceae; Propionibacterium
NC_015453	RefSeq	RVG	G1082	29,017	54	Propionibacterium phage PAS50	Viruses; dsDNA viruses, no RNA stage; Caudovirales; Siphoviridae.	504553	Bacteria; Actinobacteria; Actinobacteria; Propionibacteriales; Propionibacteriaceae; Propionibacterium
NC_009541	RefSeq	RVG	G1082	29,739	54	Propionibacterium phage PA6	Viruses; dsDNA viruses, no RNA stage; Caudovirales; Siphoviridae.	376758	Bacteria; Actinobacteria; Actinobacteria; Propionibacteriales; Propionibacteriaceae; Propionibacterium
KJ578789	EBI	RVG	G1082	29,264	54.2	Propionibacterium phage PHL194M00	Viruses; dsDNA viruses, no RNA stage; Caudovirales; Siphoviridae.	1500829	-
NC_027400	RefSeq	RVG	G1082	29,264	54.2	Propionibacterium phage PHL055N00	Viruses; dsDNA viruses, no RNA stage; Caudovirales; Siphoviridae.	1500802	Bacteria; Actinobacteria; Actinobacteria; Propionibacteriales; Propionibacteriaceae; Propionibacterium
KJ578786	EBI	RVG	G1082	29,264	54.2	Propionibacterium phage PHL163M00	Viruses; dsDNA viruses, no RNA stage; Caudovirales; Siphoviridae.	1500826	-
KJ578778	EBI	RVG	G1082	29,255	54.2	Propionibacterium phage PHL117M00	Viruses; dsDNA viruses, no RNA stage; Caudovirales; Siphoviridae.	1500818	-
NC_027385	RefSeq	RVG	G1082	29,261	53.9	Propionibacterium phage PHL092M00	Viruses; dsDNA viruses, no RNA stage; Caudovirales; Siphoviridae.	1500813	Bacteria; Actinobacteria; Actinobacteria; Propionibacteriales; Propionibacteriaceae; Propionibacterium
NC_028694	RefSeq	RVG	G1082	29,407	53.9	Propionibacterium phage PA1-14	Viruses; dsDNA viruses, no RNA stage; Caudovirales; Siphoviridae.	1747271	-
NC_022342	RefSeq	RVG	G1082	29,140	54.3	Propionibacterium phage PHL111M01	Viruses; dsDNA viruses, no RNA stage; Caudovirales; Siphoviridae.	1235653	Bacteria; Actinobacteria; Actinobacteria; Propionibacteriales; Propionibacteriaceae; Propionibacterium
NC_022341	RefSeq	RVG	G1082	29,200	54.1	Propionibacterium phage PHL113M01	Viruses; dsDNA viruses, no RNA stage; Caudovirales; Siphoviridae.	1235655	Bacteria; Actinobacteria; Actinobacteria; Propionibacteriales; Propionibacteriaceae; Propionibacterium
KJ578769	EBI	RVG	G1082	29,491	54.4	Propionibacterium phage PHL082M02	Viruses; dsDNA viruses, no RNA stage; Caudovirales; Siphoviridae.	1500809	-

NC_027359	RefSeq	RVG	G1082	29,491	54.4	Propionibacterium phage PHL082M00	Viruses; dsDNA viruses, no RNA stage; Caudovirales; Siphoviridae.	1500808	Bacteria; Actinobacteria; Actinobacteria; Propionibacteriales; Propionibacteriaceae; Propionibacterium
KJ578771	EBI	RVG	G1082	29,491	54.4	Propionibacterium phage PHL082M04	Viruses; dsDNA viruses, no RNA stage; Caudovirales; Siphoviridae.	1500811	-
KJ578770	EBI	RVG	G1082	29,491	54.4	Propionibacterium phage PHL082M03	Viruses; dsDNA viruses, no RNA stage; Caudovirales; Siphoviridae.	1500810	-
NC_027620	RefSeq	RVG	G1082	29,726	54.4	Propionibacterium phage MrAK	Viruses; dsDNA viruses, no RNA stage; Caudovirales; Siphoviridae.	1655016	Bacteria; Actinobacteria; Actinobacteria; Propionibacteriales; Propionibacteriaceae; Propionibacterium
NC_027624	RefSeq	RVG	G1082	29,594	54.6	Propionibacterium phage SKKY	Viruses; dsDNA viruses, no RNA stage; Caudovirales; Siphoviridae.	1655020	Bacteria; Actinobacteria; Actinobacteria; Propionibacteriales; Propionibacteriaceae; Propionibacterium
NC_018849	RefSeq	RVG	G1082	29,202	54.2	Propionibacterium phage P105	Viruses; dsDNA viruses, no RNA stage; Caudovirales; Siphoviridae.	1229788	Bacteria; Actinobacteria; Actinobacteria; Propionibacteriales; Propionibacteriaceae; Propionibacterium
NC_027622	RefSeq	RVG	G1082	29,330	53.8	Propionibacterium phage Stormborn	Viruses; dsDNA viruses, no RNA stage; Caudovirales; Siphoviridae.	1655022	Bacteria; Actinobacteria; Actinobacteria; Propionibacteriales; Propionibacteriaceae; Propionibacterium
KJ578777	EBI	RVG	G1082	29,396	54	Propionibacterium phage PHL116M10	Viruses; dsDNA viruses, no RNA stage; Caudovirales; Siphoviridae.	1500817	-
NC_027362	RefSeq	RVG	G1082	29,394	54	Propionibacterium phage PHL116M00	Viruses; dsDNA viruses, no RNA stage; Caudovirales; Siphoviridae.	1500816	Bacteria; Actinobacteria; Actinobacteria; Propionibacteriales; Propionibacteriaceae; Propionibacterium
NC_018839	RefSeq	RVG	G1082	29,729	54.1	Propionibacterium phage P14.4	Viruses; dsDNA viruses, no RNA stage; Caudovirales; Siphoviridae.	1229784	Bacteria; Actinobacteria; Actinobacteria; Propionibacteriales; Propionibacteriaceae; Propionibacterium
NC_027629	RefSeq	RVG	G1082	28,876	54.7	Propionibacterium phage Attacne	Viruses; dsDNA viruses, no RNA stage; Caudovirales; Siphoviridae.	1655012	Bacteria; Actinobacteria; Actinobacteria; Propionibacteriales; Propionibacteriaceae; Propionibacterium
NC_027621	RefSeq	RVG	G1082	29,463	54.4	Propionibacterium phage Wizzo	Viruses; dsDNA viruses, no RNA stage; Caudovirales; Siphoviridae.	1655023	Bacteria; Actinobacteria; Actinobacteria; Propionibacteriales; Propionibacteriaceae; Propionibacterium
NC_018851	RefSeq	RVG	G1082	29,516	54	Propionibacterium phage ATCC29399B_C	Viruses; dsDNA viruses, no RNA stage; Caudovirales; Siphoviridae.	1229794	Bacteria; Actinobacteria; Actinobacteria; Propionibacteriales; Propionibacteriaceae; Propionibacterium
NC_018847	RefSeq	RVG	G1082	29,516	54	Propionibacterium phage ATCC29399B_T	Viruses; dsDNA viruses, no RNA stage; Caudovirales; Siphoviridae.	1229793	Bacteria; Actinobacteria; Actinobacteria; Propionibacteriales; Propionibacteriaceae; Propionibacterium
NC_022334	RefSeq	RVG	G1082	29,266	54.5	Propionibacterium phage PHL112N00	Viruses; dsDNA viruses, no RNA stage; Caudovirales; Siphoviridae.	1235654	Bacteria; Actinobacteria; Actinobacteria; Propionibacteriales; Propionibacteriaceae; Propionibacterium
NC_027391	RefSeq	RVG	G1082	29,412	54.3	Propionibacterium phage PHL041M10	Viruses; dsDNA viruses, no RNA stage; Caudovirales; Siphoviridae.	1500801	Bacteria; Actinobacteria; Actinobacteria; Propionibacteriales; Propionibacteriaceae; Propionibacterium
JX570708	EBI	RVG	G1082	29,453	53.8	Propionibacterium phage PHL115M02	Viruses; dsDNA viruses, no RNA stage; Caudovirales; Siphoviridae.	1235657	-
JX570707	EBI	RVG	G1082	29,451	53.8	Propionibacterium phage PHL085M01	Viruses; dsDNA viruses, no RNA stage; Caudovirales; Siphoviridae.	1235652	-
NC_027361	RefSeq	RVG	G1082	29,454	53.8	Propionibacterium phage PHL085N00	Viruses; dsDNA viruses, no RNA stage; Caudovirales; Siphoviridae.	1500812	Bacteria; Actinobacteria; Actinobacteria; Propionibacteriales; Propionibacteriaceae; Propionibacterium
JX570706	EBI	RVG	G1082	29,443	53.8	Propionibacterium phage PHL037M02	Viruses; dsDNA viruses, no RNA stage; Caudovirales; Siphoviridae.	1235646	-
NC_027346	RefSeq	RVG	G1082	29,327	53.7	Propionibacterium phage PHL171M01	Viruses; dsDNA viruses, no RNA stage; Caudovirales; Siphoviridae.	1500827	Bacteria; Actinobacteria; Actinobacteria; Propionibacteriales; Propionibacteriaceae; Propionibacterium
NC_027630	RefSeq	RVG	G1082	29,506	53.9	Propionibacterium phage Ouroboros	Viruses; dsDNA viruses, no RNA stage; Caudovirales; Siphoviridae.	1655017	Bacteria; Actinobacteria; Actinobacteria; Propionibacteriales; Propionibacteriaceae; Propionibacterium
NC_027333	RefSeq	RVG	G1082	29,421	54	Propionibacterium phage PHL070N00	Viruses; dsDNA viruses, no RNA stage; Caudovirales; Siphoviridae.	1500807	Bacteria; Actinobacteria; Actinobacteria; Propionibacteriales; Propionibacteriaceae; Propionibacterium
NC_018838	RefSeq	RVG	G1082	29,505	53.8	Propionibacterium phage P100_A	Viruses; dsDNA viruses, no RNA stage; Caudovirales; Siphoviridae.	1229790	Bacteria; Actinobacteria; Actinobacteria; Propionibacteriales; Propionibacteriaceae; Propionibacterium
NC_027628	RefSeq	RVG	G1082	29,517	53.9	Propionibacterium phage Lauchelly	Viruses; dsDNA viruses, no RNA stage; Caudovirales; Siphoviridae.	1655015	Bacteria; Actinobacteria; Actinobacteria; Propionibacteriales; Propionibacteriaceae; Propionibacterium
KJ578775	EBI	RVG	G1082	29,464	54.2	Propionibacterium phage PHL114N00	Viruses; dsDNA viruses, no RNA stage; Caudovirales; Siphoviridae.	1500815	-
NC_022340	RefSeq	RVG	G1082	29,464	54.2	Propionibacterium phage PHL114L00	Viruses; dsDNA viruses, no RNA stage; Caudovirales; Siphoviridae.	1235656	Bacteria; Actinobacteria; Actinobacteria; Propionibacteriales; Propionibacteriaceae; Propionibacterium
NC_027295	RefSeq	RVG	G1082	29,806	54	Propionibacterium phage PHL199M00	Viruses; dsDNA viruses, no RNA stage; Caudovirales; Siphoviridae.	1500830	Bacteria; Actinobacteria; Actinobacteria; Propionibacteriales; Propionibacteriaceae; Propionibacterium
KJ578779	EBI	RVG	G1082	29,422	53.9	Propionibacterium phage PHL117M01	Viruses; dsDNA viruses, no RNA stage; Caudovirales; Siphoviridae.	1500819	-
NC_027373	RefSeq	RVG	G1082	29,423	53.9	Propionibacterium phage PHL030N00	Viruses; dsDNA viruses, no RNA stage; Caudovirales; Siphoviridae.	1500800	Bacteria; Actinobacteria; Actinobacteria; Propionibacteriales; Propionibacteriaceae; Propionibacterium
KJ578764	EBI	RVG	G1082	29,407	53.9	Propionibacterium phage PHL064M02	Viruses; dsDNA viruses, no RNA stage; Caudovirales; Siphoviridae.	1500804	-
KJ578763	EBI	RVG	G1082	29,424	53.9	Propionibacterium phage PHL064M01	Viruses; dsDNA viruses, no RNA stage; Caudovirales; Siphoviridae.	1500803	-
NC_027354	RefSeq	RVG	G1082	29,323	54.4	Propionibacterium phage PHL301M00	Viruses; dsDNA viruses, no RNA stage; Caudovirales; Siphoviridae.	1500831	Bacteria; Actinobacteria; Actinobacteria; Propionibacteriales; Propionibacteriaceae; Propionibacterium
NC_027623	RefSeq	RVG	G1082	29,328	54.1	Propionibacterium phage Pirate	Viruses; dsDNA viruses, no RNA stage; Caudovirales; Siphoviridae.	1655018	Bacteria; Actinobacteria; Actinobacteria; Propionibacteriales; Propionibacteriaceae; Propionibacterium
NC_027386	RefSeq	RVG	G1082	29,247	54.2	Propionibacterium phage PHL152M00	Viruses; dsDNA viruses, no RNA stage; Caudovirales; Siphoviridae.	1500825	Bacteria; Actinobacteria; Actinobacteria; Propionibacteriales; Propionibacteriaceae; Propionibacterium
NC_027625	RefSeq	RVG	G1082	29,461	54.5	Propionibacterium phage Kubed	Viruses; dsDNA viruses, no RNA stage; Caudovirales; Siphoviridae.	1655014	Bacteria; Actinobacteria; Actinobacteria; Propionibacteriales; Propionibacteriaceae; Propionibacterium
KJ578783	EBI	RVG	G1082	29,511	54	Propionibacterium phage PHL151M00	Viruses; dsDNA viruses, no RNA stage; Caudovirales; Siphoviridae.	1500823	-
JX570711	EBI	RVG	G1082	29,512	54	Propionibacterium phage PHL066M04	Viruses; dsDNA viruses, no RNA stage; Caudovirales; Siphoviridae.	1235648	-
KJ578784	EBI	RVG	G1082	29,511	54	Propionibacterium phage PHL151N00	Viruses; dsDNA viruses, no RNA stage; Caudovirales; Siphoviridae.	1500824	-
NC_022336	RefSeq	RVG	G1082	29,511	54	Propionibacterium phage PHL010M04	Viruses; dsDNA viruses, no RNA stage; Caudovirales; Siphoviridae.	1235645	Bacteria; Actinobacteria; Actinobacteria; Propionibacteriales; Propionibacteriaceae; Propionibacterium
JX570703	EBI	RVG	G1082	29,503	54	Propionibacterium phage PHL073M02	Viruses; dsDNA viruses, no RNA stage; Caudovirales; Siphoviridae.	1235651	-
NC_027371	RefSeq	RVG	G1082	29,741	54.4	Propionibacterium phage Pacnes 2012-15	Viruses; dsDNA viruses, no RNA stage; Caudovirales; Siphoviridae.	1498188	Bacteria; Actinobacteria; Actinobacteria; Propionibacteriales; Propionibacteriaceae; Propionibacterium
NC_002515	RefSeq	RVG	G1083	11,660	26.8	Mycoplasma phage P1	Viruses; dsDNA viruses, no RNA stage; Caudovirales; Podoviridae; Picovirinae; unassigned Picovirinae.	35238	Bacteria; Tenericutes; Mollicutes; Mycoplasmatales; Mycoplasmataceae; Mycoplasma
NC_017972	RefSeq	RVG	G1084	280,538	50.9	Pseudomonas phage Lu11	Viruses; dsDNA viruses, no RNA stage; Caudovirales; Myoviridae.	1161927	Bacteria; Proteobacteria; Gammaproteobacteria; Pseudomonadales; Pseudomonadaceae; Pseudomonas; Pseudomonas putida group

NC_022096	RefSeq	RVG	G1085	258,139	55.8	Pseudomonas phage PaBG	Viruses; dsDNA viruses, no RNA stage; Caudovirales; Myoviridae.	1335230	Bacteria; Proteobacteria; Gammaproteobacteria; Pseudomonadales; Pseudomonadaceae; Pseudomonas; Pseudomonas aeruginosa group
NC_010811	RefSeq	RVG	G1086	231,255	58	Ralstonia phage RSL1	Viruses; dsDNA viruses, no RNA stage; Caudovirales; Myoviridae.	482058	Bacteria; Proteobacteria; Betaproteobacteria; Burkholderiales; Burkholderiaceae; Ralstonia
TARA_ERS488518_N001377	TOV	EVG	G1087	19,817	58.4	-	-	-	-
TARA_ERS488340_N001411	TOV	EVG	G1087	19,817	58.3	-	-	-	-
TARA_ERS488448_N001289	TOV	EVG	G1087	19,817	58.3	-	-	-	-
TARA_ERS488499_N000791	TOV	EVG	G1087	19,817	58.3	-	-	-	-

Table S1C. Host prediction of 29 EVGs by genome-wise sequence similarity (S_G).

EVG	source of EVG	closest RVG name	similarity (S_G)	prediction precision	predicted host group	closest RVG ID	closest RVG taxonomy
TARA_ERS488558_N000636	TOV	Prochlorococcus phage P-RSP2	0.9008	>95%	Cyanobacteria	HQ332139	Viruses; dsDNA viruses, no RNA stage; unclassified dsDNA phages.
AP013542	uvMED	Pelagibacter phage HTVC019P	0.6488	>95%	*Pelagibacter*	NC_020483	Viruses; dsDNA viruses, no RNA stage; Caudovirales; Podoviridae.
AP013538	uvMED	Cyanophage KBS-P-1A	0.6324	>95%	Cyanobacteria	NC_020865	Viruses; dsDNA viruses, no RNA stage; unclassified dsDNA phages.
OBV_N00003	OBV	Cyanophage P-RSM6	0.495	>95%	Cyanobacteria	NC_020855	Viruses; dsDNA viruses, no RNA stage; Caudovirales; Myoviridae.
TARA_ERS491107_N000181	TOV	Pseudoalteromonas Phage H103	0.483	>95%	*Pseudoalteromonas*	NC_028819	Viruses; dsDNA viruses, no RNA stage; Caudovirales.
AP013543	uvMED	Pelagibacter phage HTVC011P	0.4445	>95%	*Pelagibacter*	NC_020482	Viruses; dsDNA viruses, no RNA stage; Caudovirales; Podoviridae.
TARA_ERS488737_N000081	TOV	Synechococcus phage S-EIVl	0.3832	>90%	Cyanobacteria	KJ410740	Viruses; unclassified phages.
AP013539	uvMED	Cyanophage KBS-P-1A	0.3823	>90%	Cyanobacteria	NC_020865	Viruses; dsDNA viruses, no RNA stage; unclassified dsDNA phages.
TARA_ERS490026_N000066	TOV	Synechococcus phage S-RIP1	0.382	>90%	Cyanobacteria	NC_020867	Viruses; dsDNA viruses, no RNA stage; Caudovirales; Podoviridae.
TARA_ERS488929_N000037	TOV	Synechococcus phage S-EIVl	0.379	>90%	Cyanobacteria	KJ410740	Viruses; unclassified phages.
AP013504	uvMED	Prochlorococcus phage MED4-184	0.373	>90%	Cyanobacteria	NC_020847	Viruses; dsDNA viruses, no RNA stage; Caudovirales; Myoviridae.
AP013502	uvMED	Prochlorococcus phage MED4-184	0.3707	>90%	Cyanobacteria	NC_020847	Viruses; dsDNA viruses, no RNA stage; Caudovirales; Myoviridae.
TARA_ERS488448_N000369	TOV	Pelagibacter phage HTVC019P	0.3704	>90%	*Pelagibacter*	NC_020483	Viruses; dsDNA viruses, no RNA stage; Caudovirales; Podoviridae.
AP013545	uvMED	Pelagibacter phage HTVC019P	0.3606	>90%	*Pelagibacter*	NC_020483	Viruses; dsDNA viruses, no RNA stage; Caudovirales; Podoviridae.
TARA_ERS478052_N000257	TOV	Pelagibacter phage HTVC019P	0.3566	>90%	*Pelagibacter*	NC_020483	Viruses; dsDNA viruses, no RNA stage; Caudovirales; Podoviridae.
TARA_ERS488518_N000405	TOV	Pelagibacter phage HTVC019P	0.356	>90%	*Pelagibacter*	NC_020483	Viruses; dsDNA viruses, no RNA stage; Caudovirales; Podoviridae.
TARA_ERS489943_N000335	TOV	Synechococcus phage S-CBP3	0.3532	>90%	Cyanobacteria	NC_025461	Viruses; dsDNA viruses, no RNA stage; unclassified dsDNA phages.
AP013387	uvMED	Pelagibacter phage HTVC010P	0.3531	>90%	*Pelagibacter*	NC_020481	Viruses; dsDNA viruses, no RNA stage; Caudovirales; Podoviridae.
TARA_ERS490053_N000074	TOV	Synechococcus phage S-CBS4	0.3494	>90%	Cyanobacteria	NC_016766	Viruses; dsDNA viruses, no RNA stage; Caudovirales; Siphoviridae.
AP013541	uvMED	Pelagibacter phage HTVC019P	0.3449	>90%	*Pelagibacter*	NC_020483	Viruses; dsDNA viruses, no RNA stage; Caudovirales; Podoviridae.
OBV_N00073	OBV	Pelagibacter phage HTVC019P	0.3428	>90%	*Pelagibacter*	NC_020483	Viruses; dsDNA viruses, no RNA stage; Caudovirales; Podoviridae.
AP013506	uvMED	Prochlorococcus phage MED4-184	0.3394	>90%	Cyanobacteria	NC_020847	Viruses; dsDNA viruses, no RNA stage; Caudovirales; Myoviridae.
AP013503	uvMED	Prochlorococcus phage MED4-184	0.3392	>90%	Cyanobacteria	NC_020847	Viruses; dsDNA viruses, no RNA stage; Caudovirales; Myoviridae.
AP013505	uvMED	Prochlorococcus phage MED4-184	0.3342	>90%	Cyanobacteria	NC_020847	Viruses; dsDNA viruses, no RNA stage; Caudovirales; Myoviridae.
TARA_ERS478007_N000062	TOV	Synechococcus phage S-RIP2	0.3195	>90%	Cyanobacteria	NC_020838	Viruses; dsDNA viruses, no RNA stage; Caudovirales; Podoviridae.
AP013508	uvMED	Cyanophage MED4-117	0.3094	>90%	Cyanobacteria	NC_020857	Viruses; dsDNA viruses, no RNA stage; unclassified dsDNA phages.
AP013386	uvMED	Pelagibacter phage HTVC010P	0.3055	>90%	*Pelagibacter*	NC_020481	Viruses; dsDNA viruses, no RNA stage; Caudovirales; Podoviridae.
TARA_ERS490120_N000335	TOV	Synechococcus phage S-CBP42	0.2986	>90%	Cyanobacteria	NC_029031	Viruses; dsDNA viruses, no RNA stage; Caudovirales; Podoviridae.
TARA_ERS490142_N000309	TOV	Synechococcus phage Syn5	0.2972	>90%	Cyanobacteria	NC_009531	Viruses; dsDNA viruses, no RNA stage; Caudovirales; Podoviridae; Autographivirinae; unassigned Autographivirinae.

Table S1D. Viral structural protein detected in 58 putative archaeal EVGs.

genome ID	clade	genus-level gOTU	#hit to structural motif	E-values of HMMER search against Pfam (best hit for each gene)								
				Phage portal protein (PF04860.8)	Phage-related minor tail protein (PF10145.5)	Caudovirus prohead serine protease (PF04586.13)	Phage tail assembly chaperone protein (PF16778.1)	Putative phage serine protease XkdF (PF14550.2)	Phage capsid family (PF05065.9)	Putative phage tail protein (PF13550.2)	Bacteriophage T4, Gp8 (PF09215.6)	Bacteriophage HK97-gp10, putative tail-component (PF04883.8)
TARA_ERS488448_N000032	clade 1	G453	2	gene85 (2e-10)	-	-	-	gene80 (4.7e-10)	-	-	-	-
TARA_ERS490053_N000033	clade 1	G453	2	gene14 (1.3e-10)	-	-	-	gene19 (4.4e-10)	-	-	-	-
TARA_ERS490053_N000029	clade 1	G453	3	gene7 (1.9e-12)	-	-	-	gene2 (4.3e-11)	gene123 (6.4e-05)	-	-	-
TARA_ERS488836_N000019	clade 1	G453	2	gene84 (4.9e-10)	-	gene79 (2.1e-10)	-	-	-	-	-	-
TARA_ERS489285_N000041	clade 1	G453	2	gene92 (1.1e-09)	-	gene97 (2.3e-10)	-	-	-	-	-	-
TARA_ERS488929_N000013	clade 1	G453	3	gene27 (3e-10)	-	gene22 (3.8e-09)	-	-	gene21 (6.9e-05)	-	-	-
TARA_ERS490452_N000042	clade 1	G453	2	gene25 (7.1e-12)	-	gene30 (3.4e-11)	-	-	-	-	-	-
TARA_ERS488354_N000017	clade 1	G453	2	gene26 (1.7e-10)	-	gene21 (9.4e-11)	-	-	-	-	-	-
TARA_ERS489285_N000047	clade 1	G453	2	gene82 (2e-11)	-	gene77 (2e-10)	-	-	-	-	-	-
TARA_ERS488701_N000010	clade 1	G453	2	gene77 (4.4e-10)	-	gene72 (7.9e-10)	-	-	-	-	-	-
TARA_ERS490346_N000059	clade 1	G453	3	gene69 (2.8e-09)	-	gene74 (9.4e-10)	gene2 (1.9e-08)	-	-	-	-	-
TARA_ERS490120_N000069	clade 1	G453	3	gene74 (2.7e-09)	-	gene79 (7.2e-10)	gene1 (3.6e-09)	-	-	-	-	-
TARA_ERS489285_N000032	clade 1	G453	4	gene58 (1.1e-07)	-	gene63 (3.1e-10)	gene85 (5.9e-10)	-	-	gene78 (6.5e-05)	-	-
TARA_ERS490142_N000090	clade 1	G453	2	gene43 (3.2e-10)	-	gene38 (1.2e-09)	-	-	-	-	-	-
TARA_ERS489285_N000043	clade 1	G453	2	gene94 (1.1e-10)	-	gene89 (7.7e-10)	-	-	-	-	-	-
TARA_ERS489285_N000042	clade 1	G453	2	gene24 (3.7e-09)	-	gene29 (1.5e-10)	-	-	-	-	-	-
TARA_ERS488929_N000012	clade 1	G453	2	gene17 (6.4e-10)	-	gene12 (3.3e-10)	-	-	-	-	-	-
TARA_ERS490452_N000040	clade 1	G454	2	gene29 (2.3e-15)	-	gene37 (2.1e-12)	-	-	-	-	-	-
TARA_ERS489113_N000073	clade 2	G455	3	gene125 (1.9e-13)	-	gene1 (2e-08)	-	gene132 (5.2e-06)	-	-	-	-
TARA_ERS490204_N000056	clade 2	G455	3	gene49 (8e-14)	-	gene42 (1.6e-10)	-	-	-	-	gene20 (1.8e-05)	-
TARA_ERS490494_N000007	clade 2	G455	2	gene145 (1.2e-12)	-	gene136 (2.7e-09)	-	-	-	-	-	-
TARA_ERS490142_N000117	clade 2	G455	2	gene111 (1.6e-10)	-	-	-	gene102 (1.1e-10)	-	-	-	-
TARA_ERS488813_N000045	clade 2	G455	2	gene45 (4.3e-12)	-	gene38 (2.2e-09)	-	-	-	-	-	-
TARA_ERS490053_N000040	clade 2	G455	2	gene69 (5.4e-14)	-	gene77 (2.4e-11)	-	-	-	-	-	-
TARA_ERS490346_N000089	clade 2	G455	3	gene66 (2.7e-11)	-	gene55 (9.8e-10)	gene33 (2e-14)	-	-	-	-	-
OBV_N00010	clade 2	G455	3	gene18 (2.4e-13)	-	-	gene60 (3e-09)	gene31 (7.4e-10)	-	-	-	-
TARA_ERS488892_N000014	clade 2	G455	2	gene26 (1.9e-11)	-	gene15 (6.8e-11)	-	-	-	-	-	-
TARA_ERS490120_N000097	clade 2	G455	3	gene114 (3.5e-11)	-	-	gene82 (5.8e-13)	gene105 (2.3e-10)	-	-	-	-
TARA_ERS490494_N000013	clade 2	G456	2	gene57 (2.9e-09)	-	gene65 (5.8e-13)	-	-	-	-	-	-
TARA_ERS488929_N000018	clade 2	G456	2	gene2 (4.1e-09)	-	gene8 (2.6e-11)	-	-	-	-	-	-
TARA_ERS489285_N000062	clade 2	G456	2	gene84 (2e-10)	-	gene91 (2.5e-12)	-	-	-	-	-	-
TARA_ERS490494_N000035	clade 3	G457	2	gene41 (2.3e-10)	gene14 (9e-16)	-	-	-	-	-	-	-
OBV_N00021	clade 3	G457	3	gene76 (1.1e-09)	gene52 (2.9e-18)	-	gene68 (9.6e-06)	-	-	-	-	-
TARA_ERS490320_N000018	clade 3	G457	2	gene53 (6.3e-08)	gene28 (7.5e-16)	-	-	-	-	-	-	-
TARA_ERS490285_N000129	clade 3	G457	2	gene27 (6.3e-08)	gene52 (7.8e-16)	-	-	-	-	-	-	-
TARA_ERS490452_N000108	clade 3	G457	2	gene26 (1.7e-07)	gene5 (1.8e-14)	-	-	-	-	-	-	-
TARA_ERS490120_N000153	clade 3	G457	2	gene76 (5.9e-12)	gene53 (6.5e-17)	-	-	-	-	-	-	-
TARA_ERS488836_N000030	clade 3	G457	3	gene17 (1.9e-11)	gene39 (9.9e-16)	-	gene20 (2.8e-06)	-	-	-	-	-
TARA_ERS478007_N000020	clade 3	G457	2	gene18 (3.7e-06)	gene76 (6.3e-17)	-	-	-	-	-	-	-
TARA_ERS491107_N000077	clade 3	G457	1	-	gene22 (3.2e-14)	-	-	-	-	-	-	-
TARA_ERS490452_N000106	clade 3	G457	2	gene4 (5.7e-09)	gene23 (1.8e-15)	-	-	-	-	-	-	-
TARA_ERS490053_N000090	clade 3	G457	2	gene14 (2.5e-08)	gene60 (4.7e-14)	-	-	-	-	-	-	-
TARA_ERS492160_N000171	clade 3	G457	2	gene51 (3.9e-07)	gene30 (4.2e-16)	-	-	-	-	-	-	-
TARA_ERS492198_N000063	clade 3	G457	2	gene65 (1.4e-06)	gene13 (3e-13)	-	-	-	-	-	-	-
TARA_ERS488929_N000052	clade 3	G457	2	gene9 (2.5e-11)	gene26 (6.6e-16)	-	-	-	-	-	-	-
TARA_ERS490346_N000180	clade 3	G457	2	gene5 (9.5e-10)	gene25 (1.8e-16)	-	-	-	-	-	-	-
TARA_ERS488836_N000047	clade 3	G457	2	gene22 (5.7e-08)	gene2 (5.9e-13)	-	-	-	-	-	-	-
TARA_ERS478007_N000024	clade 3	G457	2	gene60 (1.9e-07)	gene9 (6.5e-15)	-	-	-	-	-	-	-
TARA_ERS488836_N000039	clade 3	G457	1	-	gene61 (1.2e-19)	-	-	-	-	-	-	-

TARA_ERS489084_N000047	clade 3	G457	2	gene15 (4.5e-09)	gene34 (5.3e-10)	-	-	-	-	-	-	-
TARA_ERS490204_N000107	clade 3	G457	2	gene32 (4.3e-13)	gene15 (4.6e-19)	-	-	-	-	-	-	-
TARA_ERS489084_N000038	clade 3	G457	2	gene27 (6.9e-07)	gene48 (3e-15)	-	-	-	-	-	-	-
TARA_ERS488558_N000319	clade 3	G457	2	gene41 (1.4e-05)	gene62 (2.9e-13)	-	-	-	-	-	-	-
TARA_ERS489084_N000033	clade 3	G457	2	gene30 (1.5e-07)	gene12 (7.5e-12)	-	-	-	-	-	-	-
TARA_ERS488701_N000035	clade 4	G458	5	gene10 (9.5e-33)	gene24 (8e-26)	gene13 (1.8e-07)	gene57 (1.1e-09)	-	gene14 (1.9e-12)	-	-	-
TARA_ERS489148_N000025	clade 4	G458	4	gene44 (7.1e-33)	gene30 (8e-26)	gene41 (2.6e-07)	-	-	gene40 (3.3e-12)	-	-	-
TARA_ERS489285_N000092	clade 4	G458	4	gene42 (7.8e-35)	gene10 (6.7e-30)	-	-	-	gene36 (1.7e-11)	-	-	gene41 (5.7e-06)
TARA_ERS489285_N000094	clade 4	G458	3	gene2 (2.2e-33)	gene66 (6.2e-34)	-	-	-	gene74 (2.3e-16)	-	-	-

Table S1E. Detected Fe-S cluster assembly genes and Fe-S related genes in nine T4-like EVGs.

OBV_N00005	Query gene	Pfam ID	Pfam Accession	Evalue of HMMER to Pfam	Pfam Definition	TIGRFAMs ID	Evalue of HMMER to TIGRFAMs	TIGRFAMs name/function	COG ID	COG symbol	Evalue of RPS-BLAST to COG	COG name	COG function	COG function name
Fe-S related	OBV_N00005_112	SPASM	PF13186.2	5.20E-07	Iron-sulfur cluster-binding domain	TIGR04317	8.20E-12	W_rSAM_matur: tungsten cofactor oxidoreducase radical SAM maturase	COG0535	COG0535	1.00E-05	Radical SAM superfamily enzyme, MoaA/NifB/PqqE/SkfB family	R	General function prediction only
	OBV_N00005_148	PDDEXK_1	PF12705.3	3.30E-06	PD-(D/E)XK nuclease superfamily	TIGR00372	1.50E-08	cas4: CRISPR-associated protein Cas4	-	-	-	-	-	-
Fe-S assembly	OBV_N00005_155	Fe-S_biosyn	PF01521.16	5.00E-07	Iron-sulphur cluster biosynthesis	TIGR01997	2.10E-13	sufA_proteo: FeS assembly scaffold SufA	COG0316	sufA	8.00E-12	Fe-S cluster assembly iron-binding protein IscA	O	Posttranslational modification, protein turnover, chaperones
	OBV_N00005_156	NifU_N	PF01592.12	1.50E-44	NifU-like N terminal domain	TIGR01999	6.50E-50	iscU: FeS cluster assembly scaffold IscU	COG0822	IscU	9.00E-40	NifU homolog involved in Fe-S cluster formation	O	Posttranslational modification, protein turnover, chaperones

TARA_ERS490953_N000001	Query gene	Pfam ID	Pfam Accession	Evalue of HMMER to Pfam	Pfam Definition	TIGRFAMs ID	Evalue of HMMER to TIGRFAMs	TIGRFAMs name/function	COG ID	COG symbol	Evalue of RPS-BLAST to COG	COG name	COG function	COG function name
Fe-S related	TARA_ERS490953_N000001_33	Glutaredoxin	PF00462.20	1.90E-14	Glutaredoxin	TIGR02181	6.10E-14	GRX_bact: glutaredoxin 3	COG0695	GrxC	3.00E-13	Glutaredoxin	O	Posttranslational modification, protein
	TARA_ERS490953_N000001_51	CLP_protease	PF00574.19	6.10E-27	Clp protease	TIGR00493	7.40E-18	clpP: ATP-dependent Clp endopeptidase, proteolytic subunit ClpP	COG0740	ClpP	1.00E-23	ATP-dependent protease ClpP, protease subunit	O	Posttranslational modification, protein turnover, chaperones
Fe-S assembly	TARA_ERS490953_N000001_64	Fe-S_biosyn	PF01521.16	3.40E-09	Iron-sulphur cluster biosynthesis	TIGR00049	1.50E-20	TIGR00049: iron-sulfur cluster assembly accessory protein	COG0316	sufA	3.00E-23	Fe-S cluster assembly iron-binding protein IscA	O	Posttranslational modification, protein turnover, chaperones
Fe-S related	TARA_ERS490953_N000001_123	-	-	-	-	TIGR04085	9.70E-10	rSAM_more_4Fe4S: radical SAM additional 4Fe4S-binding SPASM domain	COG0535	COG0535	4.00E-06	Radical SAM superfamily enzyme, MoaA/NifB/PqqE/SkfB family	R	General function prediction only
	TARA_ERS490953_N000001_126	Radical_SAM	PF04055.17	1.90E-05	Radical SAM superfamily	-	-	-	-	-	-	-	-	-
	TARA_ERS490953_N000001_128	Radical_SAM	PF04055.17	2.30E-06	Radical SAM superfamily	TIGR04013	3.60E-06	B12_SAM_MJ_1487: B12-binding domain/radical SAM domain protein, MJ_1487 family	COG1032	COG1032	3.00E-09	Radical SAM superfamily enzyme YgiQ, UPF0313 family	R	General function prediction only
	TARA_ERS490953_N000001_131	-	-	-	-	TIGR03942	8.30E-09	sulfatase_rSAM: anaerobic sulfatase maturase	-	-	-	-	-	-
	TARA_ERS490953_N000001_132	Radical_SAM	PF04055.17	9.80E-06	Radical SAM superfamily	TIGR02495	4.00E-06	NrdG2: anaerobic ribonucleoside-triphosphate reductase activating protein	COG0535	COG0535	1.00E-05	Radical SAM superfamily enzyme, MoaA/NifB/PqqE/SkfB family	R	General function prediction only

TARA_ERS488673_N000052	Query gene	Pfam ID	Pfam Accession	Evalue of HMMER to Pfam	Pfam Definition	TIGRFAMs ID	Evalue of HMMER to TIGRFAMs	TIGRFAMs name/function	COG ID	COG symbol	Evalue of RPS-BLAST to COG	COG name	COG function	COG function name
Fe-S related	TARA_ERS488673_N000052_38	PDDEXK_1	PF12705.3	1.10E-06	PD-(D/E)XK nuclease superfamily	TIGR00372	4.40E-09	cas4: CRISPR-associated protein Cas4	-	-	-	-	-	-
	TARA_ERS488673_N000052_80	Radical_SAM	PF04055.17	1.20E-11	Radical SAM superfamily	TIGR02668	6.00E-19	moaA_archaeal: probable molybdenum cofactor biosynthesis protein A	COG2896	MoaA	9.00E-10	Molybdenum cofactor biosynthesis enzyme MoaA	H	Coenzyme transport and metabolism
Fe-S assembly	TARA_ERS488673_N000052_127	Fe-S_biosyn	PF01521.16	1.10E-10	Iron-sulphur cluster biosynthesis	TIGR00049	2.90E-19	TIGR00049: iron-sulfur cluster assembly accessory protein	COG0316	sufA	2.00E-26	Fe-S cluster assembly iron-binding protein IscA	O	Posttranslational modification, protein turnover, chaperones
Fe-S related	TARA_ERS488673_N000052_151	-	-	-	-	TIGR04085	7.80E-08	rSAM_more_4Fe4S: radical SAM additional 4Fe4S-binding SPASM domain	COG0535	COG0535	3.00E-05	Radical SAM superfamily enzyme, MoaA/NifB/PqqE/SkfB family	R	General function prediction only
	TARA_ERS488673_N000052_152	-	-	-	-	TIGR03942	4.60E-06	sulfatase_rSAM: anaerobic sulfatase maturase	-	-	-	-	-	-

TARA_ERS488813_N000021	Query gene	Pfam ID	Pfam Accession	Evalue of HMMER to Pfam	Pfam Definition	TIGRFAMs ID	Evalue of HMMER to TIGRFAMs	TIGRFAMs name/function	COG ID	COG symbol	Evalue of RPS-BLAST to COG	COG name	COG function	COG function name
Fe-S related	TARA_ERS488813_N000021_23	-	-	-	-	-	-	-	COG0535	COG0535	8.00E-06	Radical SAM superfamily enzyme, MoaA/NifB/PqqE/SkfB family	R	General function prediction only
	TARA_ERS488813_N000021_28	Glutaredoxin	PF00462.20	6.00E-13	Glutaredoxin	TIGR02181	6.60E-17	GRX_bact: glutaredoxin 3	COG0695	GrxC	2.00E-16	Glutaredoxin	O	Posttranslational modification, protein turnover, chaperones
	TARA_ERS488813_N000021_60	SPASM	PF13186.2	1.00E-12	Iron-sulfur cluster-binding domain	TIGR04317	6.50E-15	W_rSAM_matur: tungsten cofactor oxidoreducase radical SAM maturase	COG0731	COG0731	2.00E-06	Wyosine [tRNA(Phe)-imidazoG37] synthetase, radical SAM superfamily	J	Translation, ribosomal structure and biogenesis
	TARA_ERS488813_N000021_74	Radical_SAM	PF04055.17	1.50E-20	Radical SAM superfamily	TIGR03471	2.80E-36	HpnJ: hopanoid biosynthesis associated radical SAM protein HpnJ	COG1032	COG1032	3.00E-36	Radical SAM superfamily enzyme YgiQ, UPF0313 family	R	General function prediction only
	TARA_ERS488813_N000021_75	Radical_SAM	PF04055.17	2.10E-11	Radical SAM superfamily	TIGR04317	5.00E-25	W_rSAM_matur: tungsten cofactor oxidoreducase radical SAM maturase	COG0535	COG0535	4.00E-10	Radical SAM superfamily enzyme, MoaA/NifB/PqqE/SkfB family	R	General function prediction only
	TARA_ERS488813_N000021_76	Radical_SAM	PF04055.17	8.90E-08	Radical SAM superfamily	TIGR04317	7.20E-05	W_rSAM_matur: tungsten cofactor oxidoreducase radical SAM maturase	-	-	-	-	-	-
	TARA_ERS488813_N000021_77	SPASM	PF13186.2	2.90E-05	Iron-sulfur cluster-binding domain	TIGR04317	4.50E-10	W_rSAM_matur: tungsten cofactor oxidoreducase radical SAM maturase	-	-	-	-	-	-
Fe-S assembly	TARA_ERS488813_N000021_145	Fe-S_biosyn	PF01521.16	4.40E-15	Iron-sulphur cluster biosynthesis	TIGR00049	1.00E-24	TIGR00049: iron-sulfur cluster assembly accessory protein	COG0316	sufA	3.00E-28	Fe-S cluster assembly iron-binding protein IscA	O	Posttranslational modification, protein turnover, chaperones

TARA_ERS478052_N000010	Query gene	Pfam ID	Pfam Accession	Evalue of HMMER to Pfam	Pfam Definition	TIGRFAMs ID	Evalue of HMMER to TIGRFAMs	TIGRFAMs name/function	COG ID	COG symbol	Evalue of RPS-BLAST to COG	COG name	COG function	COG function name
Fe-S related	TARA_ERS478052_N000010_40	Fer4_14	PF13394.2	2.50E-08	4Fe-4S single cluster domain	TIGR03963	6.00E-06	rSAM_QueE_Clost: putative 7-cyano-7-deazaguanosine (preQ0) biosynthesis protein QueE	COG0602	NrdG	2.00E-11	Organic radical activating enzyme	R	General function prediction only
	TARA_ERS478052_N000010_73	Radical_SAM	PF04055.17	2.20E-07	Radical SAM superfamily	TIGR02666	2.20E-15	moaA: molybdenum cofactor biosynthesis protein A	COG1964	COG1964	2.00E-13	Uncharacterized Fe-S cluster-containing enzyme, radical SAM	R	General function prediction only
Fe-S assembly	TARA_ERS478052_N000010_112	Fe-S_biosyn	PF01521.16	1.00E-10	Iron-sulphur cluster biosynthesis	TIGR00049	1.10E-21	TIGR00049: iron-sulfur cluster assembly accessory protein	COG0316	sufA	2.00E-26	Fe-S cluster assembly iron-binding protein IscA	O	Posttranslational modification, protein turnover, chaperones
Fe-S related	TARA_ERS478052_N000010_126	-	-	-	-	TIGR00372	2.40E-08	cas4: CRISPR-associated protein Cas4	-	-	-	-	-	-
	TARA_ERS478052_N000010_130	Glutaredoxin	PF00462.20	4.50E-17	Glutaredoxin	TIGR02181	5.30E-21	GRX_bact: glutaredoxin 3	COG0695	GrxC	1.00E-19	Glutaredoxin	O	Posttranslational modification, protein turnover, chaperones
	TARA_ERS478052_N000010_132	Radical_SAM	PF04055.17	5.30E-08	Radical SAM superfamily	TIGR02666	2.10E-09	moaA: molybdenum cofactor biosynthesis protein A	COG0641	AslB	1.00E-05	Sulfatase maturation enzyme AslB, radical SAM superfamily	O	Posttranslational modification, protein turnover, chaperones
	TARA_ERS478052_N000010_133	Radical_SAM	PF04055.17	6.20E-06	Radical SAM superfamily	TIGR02666	1.60E-06	moaA: molybdenum cofactor biosynthesis protein A	COG0535	COG0535	3.00E-09	Radical SAM superfamily enzyme, MoaA/NifB/PqqE/SkfB family	R	General function prediction only
	TARA_ERS478052_N000010_134	Fer4_12	PF13353.2	2.60E-08	4Fe-4S single cluster domain	TIGR02666	1.00E-09	moaA: molybdenum cofactor biosynthesis protein A	COG0641	AslB	9.00E-07	Sulfatase maturation enzyme AslB, radical SAM superfamily	O	Posttranslational modification, protein turnover, chaperones
	TARA_ERS478052_N000010_135	SPASM	PF13186.2	3.10E-08	Iron-sulfur cluster-binding domain	TIGR04317	5.80E-05	W_rSAM_matur: tungsten cofactor oxidoreducase radical SAM maturase	-	-	-	-	-	-
	TARA_ERS478052_N000010_136	Radical_SAM	PF04055.17	7.10E-08	Radical SAM superfamily	TIGR04317	6.00E-11	W_rSAM_matur: tungsten cofactor oxidoreducase radical SAM maturase	COG0641	AslB	2.00E-06	Sulfatase maturation enzyme AslB, radical SAM superfamily	O	Posttranslational modification, protein turnover, chaperones
	TARA_ERS478052_N000010_137	Radical_SAM	PF04055.17	9.20E-07	Radical SAM superfamily	TIGR04317	1.60E-08	W_rSAM_matur: tungsten cofactor oxidoreducase radical SAM maturase	COG0535	COG0535	6.00E-07	Radical SAM superfamily enzyme, MoaA/NifB/PqqE/SkfB family	R	General function prediction only
	TARA_ERS478052_N000010_138	Radical_SAM	PF04055.17	1.40E-06	Radical SAM superfamily	TIGR02666	3.90E-10	moaA: molybdenum cofactor biosynthesis protein A	COG2896	MoaA	1.00E-07	Molybdenum cofactor biosynthesis enzyme MoaA	H	Coenzyme transport and metabolism
	TARA_ERS478052_N000010_144	Radical_SAM	PF04055.17	1.00E-12	Radical SAM superfamily	TIGR04479	8.70E-21	bcpD_PhpK_rSAM: radical SAM P-methyltransferase, PhpK family	COG1032	COG1032	4.00E-24	Radical SAM superfamily enzyme YgiQ, UPF0313 family	R	General function prediction only
	TARA_ERS478052_N000010_162	DUF59	PF01883.15	1.30E-16	Domain of unknown function DUF59	TIGR02945	4.40E-31	SUF_assoc: FeS assembly SUF system protein	COG2151	PaaD	3.00E-20	Metal-sulfur cluster biosynthetic enzyme	O	Posttranslational modification, protein turnover, chaperones
	TARA_ERS478052_N000010_174	Radical_SAM	PF04055.17	2.00E-14	Radical SAM superfamily	TIGR04479	2.00E-29	bcpD_PhpK_rSAM: radical SAM P-methyltransferase, PhpK family	COG1032	COG1032	8.00E-23	Radical SAM superfamily enzyme YgiQ, UPF0313 family	R	General function prediction only
	TARA_ERS478052_N000010_175	Radical_SAM	PF04055.17	1.70E-07	Radical SAM superfamily	TIGR04013	2.80E-10	B12_SAM_MJ_1487: B12-binding domain/radical SAM domain protein, MJ_1487 family	COG1032	COG1032	3.00E-10	Radical SAM superfamily enzyme YgiQ, UPF0313 family	R	General function prediction only

TARA_ERS478007_N000001	Query gene	Pfam ID	Pfam Accession	Evalue of HMMER to Pfam	Pfam Definition	TIGRFAMs ID	Evalue of HMMER to TIGRFAMs	TIGRFAMs name/function	COG ID	COG symbol	Evalue of RPS-BLAST to COG	COG name	COG function	COG function name
Fe-S related	TARA_ERS478007_N000001_53	PDDEXK_1	PF12705.3	6.10E-06	PD-(D/E)XK nuclease superfamily	TIGR00372	4.20E-09	cas4: CRISPR-associated protein Cas4	-	-	-	-	-	-
	TARA_ERS478007_N000001_66	-	-	-	-	TIGR02181	2.30E-05	GRX_bact: glutaredoxin 3	-	-	-	-	-	-
	TARA_ERS478007_N000001_79	CLP_protease	PF00574.19	2.10E-09	Clp protease	-	-	-	COG0740	ClpP	2.00E-06	ATP-dependent protease ClpP, protease subunit	O	Posttranslational modification, protein turnover, chaperones
Fe-S assembly	TARA_ERS478007_N000001_123	Fe-S_biosyn	PF01521.16	1.50E-19	Iron-sulphur cluster biosynthesis	TIGR00049	2.40E-37	TIGR00049: iron-sulfur cluster assembly accessory protein	COG0316	sufA	5.00E-48	Fe-S cluster assembly iron-binding protein IscA	O	Posttranslational modification, protein turnover, chaperones
	TARA_ERS478007_N000001_124	NifU_N	PF01592.12	1.50E-55	NifU-like N terminal domain	TIGR01999	3.00E-70	iscU: FeS cluster assembly scaffold IscU	COG0822	IscU	7.00E-48	NifU homolog involved in Fe-S cluster formation	O	Posttranslational modification, protein turnover, chaperones

TARA_ERS488499_N000029	Query gene	Pfam ID	Pfam Accession	Evalue of HMMER to Pfam	Pfam Definition	TIGRFAMs ID	Evalue of HMMER to TIGRFAMs	TIGRFAMs name/function	COG ID	COG symbol	Evalue of RPS-BLAST to COG	COG name	COG function	COG function name
Fe-S related	TARA_ERS488499_N000029_7	CLP_protease	PF00574.19	2.10E-09	Clp protease	-	-	-	COG0740	ClpP	0.000002	ATP-dependent protease ClpP, protease subunit	O	Posttranslational modification, protein turnover, chaperones
	TARA_ERS488499_N000029_20	-	-	-	-	TIGR02181	2.30E-05	GRX_bact: glutaredoxin 3	-	-	-	-	-	-
	TARA_ERS488499_N000029_33	PDDEXK_1	PF12705.3	6.10E-06	PD-(D/E)XK nuclease superfamily	TIGR00372	4.2E-09	cas4: CRISPR-associated protein Cas4	-	-	-	-	-	-
Fe-S assembly	TARA_ERS488499_N000029_121	NifU_N	PF01592.12	1.50E-55	NifU-like N terminal domain	TIGR01999	3.00E-70	iscU: FeS cluster assembly scaffold IscU	COG0822	IscU	7.00E-48	NifU homolog involved in Fe-S cluster formation	O	Posttranslational modification, protein turnover, chaperones
	TARA_ERS488499_N000029_122	Fe-S_biosyn	PF01521.16	1.50E-19	Iron-sulphur cluster biosynthesis	TIGR00049	2.40E-37	TIGR00049: iron-sulfur cluster assembly accessory protein	COG0316	sufA	5.00E-48	Fe-S cluster assembly iron-binding protein IscA	O	Posttranslational modification, protein turnover, chaperones

TARA_ERS488448_N000015	Query gene	Pfam ID	Pfam Accession	Evalue of HMMER to Pfam	Pfam Definition	TIGRFAMs ID	Evalue of HMMER to TIGRFAMs	TIGRFAMs name/function	COG ID	COG symbol	Evalue of RPS-BLAST to COG	COG name	COG function	COG function name
Fe-S assembly	TARA_ERS488448_N000015_37	NifU_N	PF01592.12	1.50E-55	NifU-like N terminal domain	TIGR01999	3.00E-70	iscU: FeS cluster assembly scaffold IscU	COG0822	IscU	7.00E-48	NifU homolog involved in Fe-S cluster formation	O	Posttranslational modification, protein turnover, chaperones
	TARA_ERS488448_N000015_38	Fe-S_biosyn	PF01521.16	1.50E-19	Iron-sulphur cluster biosynthesis	TIGR00049	2.40E-37	TIGR00049: iron-sulfur cluster assembly accessory protein	COG0316	sufA	5.00E-48	Fe-S cluster assembly iron-binding protein IscA	O	Posttranslational modification, protein turnover, chaperones
Fe-S related	TARA_ERS488448_N000015_82	CLP_protease	PF00574.19	2.10E-09	Clp protease	-	-	-	COG0740	ClpP	2.00E-06	ATP-dependent protease ClpP, protease subunit	O	Posttranslational modification, protein turnover, chaperones
	TARA_ERS488448_N000015_95	-	-	-	-	TIGR02181	2.30E-05	GRX_bact: glutaredoxin 3	-	-	-	-	-	-
	TARA_ERS488448_N000015_108	PDDEXK_1	PF12705.3	6.10E-06	PD-(D/E)XK nuclease superfamily	TIGR00372	4.20E-09	cas4: CRISPR-associated protein Cas4	-	-	-	-	-	-

TARA_ERS490452_N000022	Query gene	Pfam ID	Pfam Accession	Evalue of HMMER to Pfam	Pfam Definition	TIGRFAMs ID	Evalue of HMMER to TIGRFAMs	TIGRFAMs name/function	COG ID	COG symbol	Evalue of RPS-BLAST to COG	COG name	COG function	COG function name
**Fe-S related**	TARA_ERS490452_N000022_53	DUF59	PF01883.15	8.50E-18	Domain of unknown function DUF59	TIGR02945	7.60E-19	SUF_assoc: FeS assembly SUF system protein	COG2151	PaaD	1.00E-16	Metal-sulfur cluster biosynthetic enzyme	O	Posttranslational modification, protein turnover, chaperones
**Fe-S assembly**	TARA_ERS490452_N000022_129	Fe-S_biosyn	PF01521.16	1.30E-13	Iron-sulphur cluster biosynthesis	TIGR00049	3.90E-24	TIGR00049: iron-sulfur cluster assembly accessory protein	COG0316	sufA	3.00E-31	Fe-S cluster assembly iron-binding protein IscA	O	Posttranslational modification, protein turnover, chaperones
**Fe-S related**	TARA_ERS490452_N000022_145	PDDEXK_1	PF12705.3	9.20E-07	PD-(D/E)XK nuclease superfamily	TIGR00372	2.60E-05	cas4: CRISPR-associated protein Cas4	-	-	-	-	-	-

Table S1F. Photosynthetic genes detected in the set of EVGs/RVGs.

genome ID	source	type	genus-level gOTU	length	%G+C	name	# photosynthetic genes detected by PSI-BLAST: ho1	pcyA	pebS	cpeT	psbA	psbD	petE	petF	ptoX	hli	nblA	taxonomy
NC_020865	RefSeq	RVG	G14	45,730	47.4	Cyanophage KBS-P-1A	0	0	0	0	1	0	0	0	0	1	0	Viruses; dsDNA viruses, no RNA stage; unclassified dsDNA phages.
NC_020838	RefSeq	RVG	G14	45,728	47.3	Synechococcus phage S-RIP2	0	0	0	0	1	0	0	0	0	1	0	Viruses; dsDNA viruses, no RNA stage; Caudovirales; Podoviridae.
AP013538	uvMED	EVG	G14	44,705	45.3	uvMED-CGR-U-MedDCM-OCT-S45-C4 (G7)	0	0	0	0	1	0	0	0	0	1	0	-
NC_020867	RefSeq	RVG	G14	44,892	42.9	Synechococcus phage S-RIP1	0	0	0	0	1	0	0	0	0	1	0	Viruses; dsDNA viruses, no RNA stage; Caudovirales; Podoviridae.
AP013539	uvMED	EVG	G14	43,444	46.7	uvMED-CGR-U-MedDCM-OCT-S28-C3 (G7)	0	0	0	0	0	0	0	0	0	0	0	-
TARA_ERS490026_N000066	TOV	EVG	G14	46,040	44.1	-	0	0	0	0	1	0	0	0	0	1	0	-
TARA_ERS489943_N000335	TOV	EVG	G14	43,667	42.9	-	0	0	0	0	1	0	0	0	0	1	0	-
NC_025464	RefSeq	RVG	G14	44,147	44.4	Synechococcus phage S-CBP4	0	0	0	0	1	0	0	0	0	1	0	Viruses; dsDNA viruses, no RNA stage; Caudovirales; Podoviridae.
NC_025461	RefSeq	RVG	G14	45,871	46.9	Synechococcus phage S-CBP3	0	0	0	0	1	0	0	0	0	1	0	Viruses; dsDNA viruses, no RNA stage; unclassified dsDNA phages.
NC_025456	RefSeq	RVG	G14	46,547	47.6	Synechococcus phage S-CBP1	0	0	0	0	1	0	0	0	0	1	0	Viruses; dsDNA viruses, no RNA stage; Caudovirales; Podoviridae; Autographivirinae; unclassified Autographivirinae.
TARA_ERS478007_N000062	TOV	EVG	G14	47,397	45	-	0	0	0	0	0	0	0	0	0	0	0	-
HQ634152	EBI	RVG	G14	47,039	39.2	Prochlorococcus phage P-SSP6	0	0	0	0	0	0	0	0	0	0	0	Viruses; dsDNA viruses, no RNA stage; Caudovirales; Podoviridae; Autographivirinae; unclassified Autographivirinae.
NC_016657	RefSeq	RVG	G14	47,055	39.2	Cyanophage 9515-10a	0	0	0	0	1	0	0	0	0	1	0	Viruses; dsDNA viruses, no RNA stage; Caudovirales; Podoviridae; Autographivirinae; unclassified Autographivirinae.
NC_020835	RefSeq	RVG	G14	47,325	39.2	Prochlorococcus phage P-SSP10	0	0	0	0	1	0	0	0	0	1	0	Viruses; dsDNA viruses, no RNA stage; Caudovirales; Podoviridae.
NC_016659	RefSeq	RVG	G14	47,536	39.9	Cyanophage NATL2A-133	0	0	0	0	1	0	0	0	0	1	0	Viruses; dsDNA viruses, no RNA stage; Caudovirales; Podoviridae; Autographivirinae; unclassified Autographivirinae.
JF974297	CAMERA	RVG	G14	32,402	42.9	Cyanophage KBS-S-1A	0	0	0	0	0	0	0	0	0	0	0	Viruses; dsDNA viruses, no RNA stage; unclassified dsDNA phages.
NC_020874	RefSeq	RVG	G14	46,198	37.9	Prochlorococcus phage P-SSP3	0	0	0	0	1	0	0	0	0	1	0	Viruses; dsDNA viruses, no RNA stage; unclassified dsDNA phages.
NC_016656	RefSeq	RVG	G14	45,890	37.9	Cyanophage P-SSP2	0	0	0	0	1	0	0	0	0	1	0	Viruses; dsDNA viruses, no RNA stage; Caudovirales.
NC_006882	RefSeq	RVG	G14	45,176	38.8	Prochlorococcus phage P-SSP7	0	0	0	0	1	0	0	0	0	1	0	Viruses; dsDNA viruses, no RNA stage; Caudovirales; Podoviridae; Autographivirinae; unassigned Autographivirinae.
NC_020878	RefSeq	RVG	G14	44,945	39.6	Prochlorococcus phage P-GSP1	0	0	0	0	1	0	0	0	0	1	0	Viruses; dsDNA viruses, no RNA stage; unclassified dsDNA phages.
NC_016658	RefSeq	RVG	G14	47,741	38.7	Cyanophage NATL1A-7	0	0	0	0	1	0	0	0	0	1	0	Viruses; dsDNA viruses, no RNA stage; Caudovirales; Podoviridae; Autographivirinae; unclassified Autographivirinae.
NC_020872	RefSeq	RVG	G14	46,997	40.5	Cyanophage SS120-1	0	0	0	0	0	0	0	0	0	0	0	Viruses; dsDNA viruses, no RNA stage; Caudovirales.
NC_009531	RefSeq	RVG	G14	46,214	55	Synechococcus phage Syn5	0	0	0	0	0	0	0	0	0	0	0	Viruses; dsDNA viruses, no RNA stage; Caudovirales; Podoviridae; Autographivirinae; unassigned Autographivirinae.
TARA_ERS490142_N000309	TOV	EVG	G14	45,651	45.6	-	0	0	0	0	0	0	0	0	0	0	0	-
TARA_ERS490120_N000335	TOV	EVG	G14	47,070	44.7	-	0	0	0	0	0	0	0	0	0	0	0	-
NC_029031	RefSeq	RVG	G14	45,218	54.6	Synechococcus phage S-CBP42	0	0	0	0	0	0	0	0	0	1	0	Viruses; dsDNA viruses, no RNA stage; Caudovirales; Podoviridae.
NC_003390	RefSeq	RVG	G14	46,675	53.3	Synechococcus phage P60	0	0	0	0	0	0	0	0	0	0	0	Viruses; dsDNA viruses, no RNA stage; Caudovirales; Podoviridae; Autographivirinae; unassigned Autographivirinae.
NC_025455	RefSeq	RVG	G14	46,237	55	Synechococcus phage S-CBP2	0	0	0	0	0	0	0	0	0	0	0	Viruses; dsDNA viruses, no RNA stage; unclassified dsDNA phages.
HQ332139	CAMERA	RVG	G15	42,257	34	Prochlorococcus phage P-RSP2	0	0	0	0	0	0	0	0	0	0	0	Viruses; dsDNA viruses, no RNA stage; unclassified dsDNA phages.
TARA_ERS488558_N000636	TOV	EVG	G15	41,574	34.1	-	0	0	0	0	0	0	0	0	0	1	0	-
AP013531	uvMED	EVG	G234	42,230	48.8	uvMED-CGR-C17C-MedDCM-OCT-S44-C5 (G4)	0	0	0	0	0	0	0	0	0	0	0	-
AP013530	uvMED	EVG	G234	42,200	48.8	uvMED-CGR-C17C-MedDCM-OCT-S23-C4 (G4)	0	0	0	0	0	0	0	0	0	0	0	-
AP013534	uvMED	EVG	G234	41,065	47.2	uvMED-CGR-U-MedDCM-OCT-S40-C11 (G4)	0	0	0	0	0	0	0	0	0	0	0	-
TARA_ERS490557_N000308	TOV	EVG	G234	39,640	42.3	-	0	0	0	0	0	0	0	0	0	0	0	-
TARA_ERS489285_N000262	TOV	EVG	G234	43,161	50.3	-	0	0	0	0	0	0	0	0	0	0	0	-
AP013533	uvMED	EVG	G234	37,887	49.7	uvMED-CGR-U-MedDCM-OCT-S38-C34 (G4)	0	0	0	0	0	0	0	0	0	0	0	-
AP013529	uvMED	EVG	G234	40,572	50.6	uvMED-CGR-U-MedDCM-OCT-S40-C13 (G3)	0	0	0	0	0	0	0	0	0	0	0	-
AP013532	uvMED	EVG	G234	38,241	51.5	uvMED-CGR-U-MedDCM-OCT-S28-C22 (G4)	0	0	0	0	0	0	0	0	0	0	0	-
TARA_ERS488929_N000265	TOV	EVG	G234	34,121	46.8	-	0	0	0	0	0	0	0	0	0	0	0	-
AP013506	uvMED	EVG	G234	38,411	38	uvMED-CGR-C14A-MedDCM-OCT-S42-C35 (G2)	0	0	0	0	0	0	0	0	0	0	0	-
AP013503	uvMED	EVG	G234	38,569	37.9	uvMED-CGR-C14A-MedDCM-OCT-S29-C22 (G2)	0	0	0	0	0	0	0	0	0	0	0	-
AP013505	uvMED	EVG	G234	38,511	37.9	uvMED-CGR-C14A-MedDCM-OCT-S38-C26 (G2)	0	0	0	0	0	0	0	0	0	0	0	-
AP013504	uvMED	EVG	G234	38,514	38	uvMED-CGR-C14A-MedDCM-OCT-S34-C29 (G2)	0	0	0	0	0	0	0	0	0	0	0	-
AP013502	uvMED	EVG	G234	38,514	37.9	uvMED-CGR-C14A-MedDCM-OCT-S23-C10 (G2)	0	0	0	0	0	0	0	0	0	0	0	-
NC_020857	RefSeq	RVG	G234	38,834	37.2	Cyanophage MED4-117	0	0	0	0	0	0	0	0	0	0	0	Viruses; dsDNA viruses, no RNA stage; unclassified dsDNA phages.
NC_020847	RefSeq	RVG	G234	38,327	37.2	Prochlorococcus phage MED4-184	0	0	0	0	0	0	0	0	0	0	0	Viruses; dsDNA viruses, no RNA stage; Caudovirales; Myoviridae.
AP013508	uvMED	EVG	G234	34,694	36.6	uvMED-CGR-C15J-MedDCM-OCT-S37-C55 (G2)	0	0	0	0	0	0	0	0	0	0	0	-
AP013509	uvMED	EVG	G234	38,614	36.1	uvMED-CGR-U-MedDCM-OCT-S35-C26 (G2)	0	0	0	0	0	0	0	0	0	0	0	-
NC_020854	RefSeq	RVG	G234	40,658	49.1	Cyanophage KBS-S-2A	0	0	0	0	0	0	0	0	0	0	0	Viruses; dsDNA viruses, no RNA stage; unclassified dsDNA phages.
NC_016766	RefSeq	RVG	G237	69,420	50.8	Synechococcus phage S-CBS4	0	0	0	0	0	0	0	0	0	0	0	Viruses; dsDNA viruses, no RNA stage; Caudovirales; Siphoviridae.
TARA_ERS490053_N000074	TOV	EVG	G237	71,674	50.3	-	0	0	0	0	0	0	0	0	0	0	0	-
TARA_ERS488929_N000031	TOV	EVG	G238	79,216	40.6	-	0	0	0	0	0	0	0	0	0	0	0	-
TARA_ERS489943_N000131	TOV	EVG	G238	80,215	39.5	-	0	0	0	0	0	0	0	0	0	0	0	-
TARA_ERS490610_N000155	TOV	EVG	G238	74,738	38.6	-	0	0	0	0	0	0	0	0	0	0	0	-
TARA_ERS490452_N000077	TOV	EVG	G238	78,068	38.8	-	0	0	0	0	0	0	0	0	0	1	0	-
TARA_ERS488737_N000081	TOV	EVG	G238	73,663	40.5	-	0	0	0	0	0	0	0	0	1	0	0	-
TARA_ERS488929_N000037	TOV	EVG	G238	73,931	40.5	-	0	0	0	0	0	0	0	0	1	0	0	-
KJ410740	EBI	RVG	G238	79,178	45.9	Synechococcus phage S-EIVl	0	0	0	0	0	0	0	0	0	0	0	Viruses; unclassified phages.
TARA_ERS489084_N000023	TOV	EVG	G241	84,534	41.8	-	0	0	0	0	1	0	0	0	0	1	0	-
NC_015463	RefSeq	RVG	G242	72,332	54.5	Synechococcus phage S-CBS2	0	0	0	0	0	0	0	0	0	1	0	Viruses; dsDNA viruses, no RNA stage; Caudovirales; Siphoviridae.
OBV_N00020	OBV	EVG	G242	67,112	45.2	-	0	0	0	0	1	0	0	0	0	1	0	-
NC_013021	RefSeq	RVG	G243	107,530	52.3	Cyanophage PSS2	0	0	0	0	0	0	0	0	0	0	0	Viruses; dsDNA viruses, no RNA stage; Caudovirales; Siphoviridae.

NC_016564	RefSeq	RVG	G277	95,299	41.4	Planktothrix phage PaV-LD	0	0	0	0	0	0	0	0	0	0	1	Viruses; dsDNA viruses, no RNA stage; Caudovirales; Podoviridae.
NC_008562	RefSeq	RVG	G278	162,109	46	Microcystis aeruginosa phage Ma-LMM01	0	0	0	0	0	0	0	0	0	0	1	Viruses; dsDNA viruses, no RNA stage; Caudovirales; Myoviridae.
NC_029002	RefSeq	RVG	G278	169,223	46	Microcystis phage MaMV-DC	0	0	0	0	0	0	0	0	0	0	1	Viruses; dsDNA viruses, no RNA stage; Caudovirales; Myoviridae.
KJ019026	EBI	RVG	G386	171,282	39.4	Synechococcus phage ACG-2014a	0	0	0	1	1	1	1	1	0	2	0	Viruses; dsDNA viruses, no RNA stage; Caudovirales; Myoviridae; Tevenvirinae; T4likevirus.
KJ019158	EBI	RVG	G386	171,292	39.4	Synechococcus phage ACG-2014a	0	0	0	1	1	1	1	1	0	2	0	Viruses; dsDNA viruses, no RNA stage; Caudovirales; Myoviridae; Tevenvirinae; T4likevirus.
KJ019137	EBI	RVG	G386	171,289	39.4	Synechococcus phage ACG-2014a	0	0	0	1	1	1	1	1	0	2	0	Viruses; dsDNA viruses, no RNA stage; Caudovirales; Myoviridae; Tevenvirinae; T4likevirus.
KJ019157	EBI	RVG	G386	171,292	39.4	Synechococcus phage ACG-2014a	0	0	0	1	1	1	1	1	0	2	0	Viruses; dsDNA viruses, no RNA stage; Caudovirales; Myoviridae; Tevenvirinae; T4likevirus.
KJ019122	EBI	RVG	G386	171,285	39.4	Synechococcus phage ACG-2014a	0	0	0	1	1	1	1	1	0	2	0	Viruses; dsDNA viruses, no RNA stage; Caudovirales; Myoviridae; Tevenvirinae; T4likevirus.
KJ019030	EBI	RVG	G386	171,279	39.4	Synechococcus phage ACG-2014a	0	0	0	1	1	1	1	1	0	2	0	Viruses; dsDNA viruses, no RNA stage; Caudovirales; Myoviridae; Tevenvirinae; T4likevirus.
KJ019076	EBI	RVG	G386	172,372	39.4	Synechococcus phage ACG-2014a	0	0	0	1	1	1	1	1	0	2	0	Viruses; dsDNA viruses, no RNA stage; Caudovirales; Myoviridae; Tevenvirinae; T4likevirus.
KJ019138	EBI	RVG	G386	171,295	39.4	Synechococcus phage ACG-2014a	0	0	0	1	1	1	1	1	0	2	0	Viruses; dsDNA viruses, no RNA stage; Caudovirales; Myoviridae; Tevenvirinae; T4likevirus.
KJ019039	EBI	RVG	G386	171,296	39.4	Synechococcus phage ACG-2014a	0	0	0	1	1	1	1	1	0	2	0	Viruses; dsDNA viruses, no RNA stage; Caudovirales; Myoviridae; Tevenvirinae; T4likevirus.
KJ019088	EBI	RVG	G386	171,276	39.4	Synechococcus phage ACG-2014a	0	0	0	1	1	1	1	1	0	2	0	Viruses; dsDNA viruses, no RNA stage; Caudovirales; Myoviridae; Tevenvirinae; T4likevirus.
KJ019055	EBI	RVG	G386	171,294	39.4	Synechococcus phage ACG-2014a	0	0	0	1	1	1	1	1	0	2	0	Viruses; dsDNA viruses, no RNA stage; Caudovirales; Myoviridae; Tevenvirinae; T4likevirus.
KJ019163	EBI	RVG	G386	171,179	39.4	Synechococcus phage ACG-2014a	0	0	0	1	1	1	1	1	0	2	0	Viruses; dsDNA viruses, no RNA stage; Caudovirales; Myoviridae; Tevenvirinae; T4likevirus.
KJ019153	EBI	RVG	G386	171,166	39.4	Synechococcus phage ACG-2014a	0	0	0	1	1	1	1	1	0	2	0	Viruses; dsDNA viruses, no RNA stage; Caudovirales; Myoviridae; Tevenvirinae; T4likevirus.
KJ019087	EBI	RVG	G386	171,238	39.4	Synechococcus phage ACG-2014a	0	0	0	1	1	1	1	1	0	2	0	Viruses; dsDNA viruses, no RNA stage; Caudovirales; Myoviridae; Tevenvirinae; T4likevirus.
KJ019116	EBI	RVG	G386	170,403	39.4	Synechococcus phage ACG-2014a	0	0	0	1	1	1	1	1	0	2	0	Viruses; dsDNA viruses, no RNA stage; Caudovirales; Myoviridae; Tevenvirinae; T4likevirus.
KJ019065	EBI	RVG	G386	171,179	39.4	Synechococcus phage ACG-2014a	0	0	0	1	1	1	1	1	0	2	0	Viruses; dsDNA viruses, no RNA stage; Caudovirales; Myoviridae; Tevenvirinae; T4likevirus.
KJ019084	EBI	RVG	G386	171,183	39.4	Synechococcus phage ACG-2014a	0	0	0	1	1	1	1	1	0	2	0	Viruses; dsDNA viruses, no RNA stage; Caudovirales; Myoviridae; Tevenvirinae; T4likevirus.
KJ019068	EBI	RVG	G386	171,231	39.4	Synechococcus phage ACG-2014a	0	0	0	1	1	1	1	1	0	2	0	Viruses; dsDNA viruses, no RNA stage; Caudovirales; Myoviridae; Tevenvirinae; T4likevirus.
KJ019033	EBI	RVG	G386	171,035	39.4	Synechococcus phage ACG-2014a	0	0	0	1	1	1	1	1	0	2	0	Viruses; dsDNA viruses, no RNA stage; Caudovirales; Myoviridae; Tevenvirinae; T4likevirus.
KJ019067	EBI	RVG	G386	171,192	39.4	Synechococcus phage ACG-2014a	0	0	0	1	1	1	1	1	0	2	0	Viruses; dsDNA viruses, no RNA stage; Caudovirales; Myoviridae; Tevenvirinae; T4likevirus.
KJ019114	EBI	RVG	G386	171,192	39.4	Synechococcus phage ACG-2014a	0	0	0	1	1	1	1	1	0	2	0	Viruses; dsDNA viruses, no RNA stage; Caudovirales; Myoviridae; Tevenvirinae; T4likevirus.
KJ019081	EBI	RVG	G386	171,192	39.4	Synechococcus phage ACG-2014a	0	0	0	1	1	1	1	1	0	2	0	Viruses; dsDNA viruses, no RNA stage; Caudovirales; Myoviridae; Tevenvirinae; T4likevirus.
KJ019135	EBI	RVG	G386	171,274	39.4	Synechococcus phage ACG-2014a	0	0	0	1	1	1	1	1	0	2	0	Viruses; dsDNA viruses, no RNA stage; Caudovirales; Myoviridae; Tevenvirinae; T4likevirus.
NC_023584	RefSeq	RVG	G386	170,438	39.4	Synechococcus phage S-MbCM100	0	0	0	1	1	1	1	1	0	2	0	Viruses; dsDNA viruses, no RNA stage; Caudovirales; Myoviridae; Tevenvirinae; T4likevirus.
KJ019038	EBI	RVG	G386	171,073	39.4	Synechococcus phage ACG-2014a	0	0	0	1	1	1	1	1	0	2	0	Viruses; dsDNA viruses, no RNA stage; Caudovirales; Myoviridae; Tevenvirinae; T4likevirus.
NC_027130	RefSeq	RVG	G386	172,688	39.1	Synechococcus phage ACG-2014b	0	0	0	1	1	1	1	1	0	2	0	Viruses; dsDNA viruses, no RNA stage; Caudovirales; Myoviridae; Tevenvirinae; T4likevirus.
NC_026924	RefSeq	RVG	G386	174,885	39.3	Synechococcus phage ACG-2014g	0	0	0	1	1	1	1	1	0	2	0	Viruses; dsDNA viruses, no RNA stage; Caudovirales; Myoviridae; Tevenvirinae; T4likevirus.
NC_021530	RefSeq	RVG	G386	171,407	39.3	Synechococcus phage S-CAM8	0	0	0	1	1	1	1	1	0	1	0	Viruses; dsDNA viruses, no RNA stage; Caudovirales; Myoviridae.
HQ317290	EBI	RVG	G386	175,430	42.2	Synechococcus phage S-RIM2 R21_2007	0	0	0	1	1	1	1	0	0	1	0	Viruses; dsDNA viruses, no RNA stage; Caudovirales; Myoviridae.
HQ317291	EBI	RVG	G386	175,419	42.2	Synechococcus phage S-RIM2 R9_2006	0	0	0	1	1	1	1	0	0	1	0	Viruses; dsDNA viruses, no RNA stage; Caudovirales; Myoviridae.
NC_020859	RefSeq	RVG	G386	175,430	42.2	Synechococcus phage S-RIM2 R1_1999	0	0	0	1	1	1	1	0	0	1	0	Viruses; dsDNA viruses, no RNA stage; Caudovirales; Myoviridae.
NC_015285	RefSeq	RVG	G386	174,285	39.6	Prochlorococcus phage Syn33	0	0	0	1	1	1	1	1	0	2	0	Viruses; dsDNA viruses, no RNA stage; Caudovirales.
KF156339	EBI	RVG	G386	176,044	39.1	Synechococcus phage S-MbCM25	0	0	0	1	1	1	1	1	0	2	0	Viruses; dsDNA viruses, no RNA stage; Caudovirales; Myoviridae; Tevenvirinae; T4likevirus.
NC_019444	RefSeq	RVG	G386	176,043	39.1	Synechococcus phage ACG-2014c	0	0	0	1	1	1	1	1	0	2	0	Viruses; dsDNA viruses, no RNA stage; Caudovirales; Myoviridae; Tevenvirinae; T4likevirus.
HQ317385	EBI	RVG	G386	168,327	40.6	Synechococcus phage S-RIM8 A.HR5	0	0	0	1	1	1	1	1	0	2	0	Viruses; dsDNA viruses, no RNA stage; Caudovirales; Myoviridae.
NC_020486	RefSeq	RVG	G386	171,211	40.6	Synechococcus phage S-RIM8 A.HR1	0	0	0	1	1	1	1	1	0	2	0	Viruses; dsDNA viruses, no RNA stage; Caudovirales; Myoviridae.
JF974289	EBI	RVG	G386	171,211	40.6	Synechococcus phage S-RIM8 A.HR3	0	0	0	1	1	1	1	1	0	2	0	Viruses; dsDNA viruses, no RNA stage; Caudovirales; Myoviridae.
NC_021072	RefSeq	RVG	G386	178,807	39.9	Cyanophage Syn30	0	0	0	1	1	1	1	0	1	1	0	Viruses; dsDNA viruses, no RNA stage; unclassified dsDNA phages.
NC_015282	RefSeq	RVG	G386	174,079	41.1	Synechococcus phage S-SM1	0	0	0	1	1	1	1	0	1	2	0	Viruses; dsDNA viruses, no RNA stage; Caudovirales; Myoviridae.
NC_021536	RefSeq	RVG	G386	171,797	40.6	Synechococcus phage S-IOM18	0	0	0	1	1	1	1	1	1	1	0	Viruses; dsDNA viruses, no RNA stage; Caudovirales; Myoviridae.
NC_015286	RefSeq	RVG	G386	175,230	41.3	Synechococcus phage Syn19	0	0	0	1	1	1	1	0	1	2	0	Viruses; dsDNA viruses, no RNA stage; Caudovirales; Myoviridae.
NC_015289	RefSeq	RVG	G386	176,184	40	Synechococcus phage S-SSM5	0	0	0	1	1	1	1	0	1	2	0	Viruses; dsDNA viruses, no RNA stage; Caudovirales; Myoviridae.
NC_015283	RefSeq	RVG	G386	176,428	37.6	Prochlorococcus phage P-RSM4	0	1	0	1	1	0	0	0	1	4	0	Viruses; dsDNA viruses, no RNA stage; Caudovirales; Myoviridae.
NC_008296	RefSeq	RVG	G386	177,300	40.6	Synechococcus phage syn9	0	0	0	1	1	1	1	0	1	2	0	Viruses; dsDNA viruses, no RNA stage; Caudovirales; Myoviridae; Tevenvirinae; T4likevirus.
NC_021559	RefSeq	RVG	G386	179,063	36.7	Prochlorococcus phage P-SSM3	0	1	0	1	1	1	0	0	1	2	0	Viruses; dsDNA viruses, no RNA stage; Caudovirales; Myoviridae.
NC_006884	RefSeq	RVG	G386	178,249	36.7	Prochlorococcus phage P-SSM4	0	1	0	1	1	1	0	0	1	4	0	Viruses; dsDNA viruses, no RNA stage; Caudovirales; Myoviridae; Tevenvirinae; T4likevirus.
NC_021071	RefSeq	RVG	G386	177,211	40.2	Cyanophage P-RSM1	0	1	0	1	1	0	1	0	1	2	0	Viruses; dsDNA viruses, no RNA stage; Caudovirales; Myoviridae.
NC_019443	RefSeq	RVG	G386	172,879	39.8	Synechococcus phage metaG-MbCM1	0	1	0	1	1	0	0	0	1	3	0	Viruses; dsDNA viruses, no RNA stage; Caudovirales; Myoviridae; Tevenvirinae; T4likevirus.
NC_015290	RefSeq	RVG	G386	182,180	37.1	Prochlorococcus phage P-SSM7	0	1	0	1	1	0	0	1	0	5	0	Viruses; dsDNA viruses, no RNA stage; Caudovirales; Myoviridae.
NC_020875	RefSeq	RVG	G386	182,801	39.4	Synechococcus phage S-SSM4	0	1	0	1	1	0	1	0	1	2	0	Viruses; dsDNA viruses, no RNA stage; Caudovirales; Myoviridae.
NC_026923	RefSeq	RVG	G386	179,110	40.3	Synechococcus phage ACG-2014d	0	0	0	1	1	1	1	1	0	2	0	Viruses; dsDNA viruses, no RNA stage; Caudovirales; Myoviridae; Tevenvirinae; T4likevirus.
NC_015281	RefSeq	RVG	G386	179,563	41.1	Synechococcus phage S-ShM2	0	0	0	1	1	1	1	0	0	1	0	Viruses; dsDNA viruses, no RNA stage; Caudovirales; Myoviridae.
NC_020855	RefSeq	RVG	G386	192,497	39.3	Cyanophage P-RSM6	1	1	1	1	1	0	1	0	0	2	0	Viruses; dsDNA viruses, no RNA stage; Caudovirales; Myoviridae.
OBV_N00003	OBV	EVG	G386	187,437	46.2	-	0	0	0	1	1	1	1	0	1	2	0	-
NC_028663	RefSeq	RVG	G386	188,632	40.7	Cyanophage P-TIM40	0	1	0	1	1	0	0	0	1	4	0	Viruses; dsDNA viruses, no RNA stage; Caudovirales; Myoviridae.
HQ633061	CAMERA	RVG	G386	180,892	39.6	Synechococcus phage S-CBM2	0	0	0	0	1	0	0	1	0	1	0	Viruses; dsDNA viruses, no RNA stage; Caudovirales; Myoviridae.
NC_020845	RefSeq	RVG	G386	180,977	37.8	Prochlorococcus phage MED4-213	1	0	1	1	1	1	0	0	1	1	0	Viruses; dsDNA viruses, no RNA stage; Caudovirales; Myoviridae.
NC_015280	RefSeq	RVG	G386	181,044	37.8	Prochlorococcus phage P-HM1	1	0	1	1	1	1	0	0	1	4	0	Viruses; dsDNA viruses, no RNA stage; Caudovirales; Myoviridae.
NC_015284	RefSeq	RVG	G386	183,806	38.1	Prochlorococcus phage P-HM2	1	0	1	1	1	1	0	0	0	4	0	Viruses; dsDNA viruses, no RNA stage; Caudovirales; Myoviridae.
NC_027132	RefSeq	RVG	G386	190,768	39	Synechococcus phage ACG-2014i	0	0	0	1	1	1	1	1	0	2	0	Viruses; dsDNA viruses, no RNA stage; Caudovirales; Myoviridae; Tevenvirinae; T4likevirus.
NC_026928	RefSeq	RVG	G386	189,418	38.9	Synechococcus phage ACG-2014e	0	0	0	1	1	1	1	1	0	2	0	Viruses; dsDNA viruses, no RNA stage; Caudovirales; Myoviridae; Tevenvirinae; T4likevirus.
NC_026926	RefSeq	RVG	G386	192,108	38.6	Synechococcus phage ACG-2014j	0	0	0	1	1	1	1	1	0	2	0	Viruses; dsDNA viruses, no RNA stage; Caudovirales; Myoviridae; Tevenvirinae; T4likevirus.
NC_023587	RefSeq	RVG	G386	189,311	40.5	Synechococcus phage ACG-2014h	0	0	0	1	1	1	1	1	0	2	0	Viruses; dsDNA viruses, no RNA stage; Caudovirales; Myoviridae; Tevenvirinae; T4likevirus.
NC_013085	RefSeq	RVG	G386	194,454	41.1	Synechococcus phage S-RSM4	0	0	0	1	1	1	1	1	1	2	0	Viruses; dsDNA viruses, no RNA stage; Caudovirales; Myoviridae; Tevenvirinae; T4likevirus.
NC_006820	RefSeq	RVG	G386	196,280	37.8	Synechococcus phage S-PM2	0	0	0	1	1	1	0	0	0	2	0	Viruses; dsDNA viruses, no RNA stage; Caudovirales; Myoviridae; Tevenvirinae; T4likevirus.

NC_015288	RefSeq	RVG	G386	191,195	40.6	Prochlorococcus phage Syn1	0	0	0	1	1	1	1	0	0	2	0	Viruses; dsDNA viruses, no RNA stage; Caudovirales.
NC_020837	RefSeq	RVG	G386	198,013	43	Synechococcus phage S-CAM1	0	0	0	1	1	1	1	1	1	1	0	Viruses; dsDNA viruses, no RNA stage; Caudovirales; Myoviridae.
NC_020851	RefSeq	RVG	G386	208,007	36	Synechococcus phage S-SKS1	0	0	0	0	1	1	0	1	0	1	0	Viruses; dsDNA viruses, no RNA stage; Caudovirales; Siphoviridae.
NC_006883	RefSeq	RVG	G386	252,401	35.5	Prochlorococcus phage P-SSM2	1	0	1	0	1	0	1	1	0	6	0	Viruses; dsDNA viruses, no RNA stage; Caudovirales; Myoviridae; Tevenvirinae; T4likevirus.
OBV_N00002	OBV	EVG	G386	191,793	41.8	-	0	0	0	0	1	1	1	0	1	2	0	-
NC_015279	RefSeq	RVG	G386	190,789	40.4	Synechococcus phage S-SM2	0	0	0	0	1	1	1	1	1	1	0	Viruses; dsDNA viruses, no RNA stage; Caudovirales; Myoviridae.
NC_028955	RefSeq	RVG	G386	197,361	34.3	Prochlorococcus phage P-TIM68	0	0	0	0	1	1	0	0	0	4	0	Viruses; dsDNA viruses, no RNA stage; Caudovirales; Myoviridae.
NC_015287	RefSeq	RVG	G386	232,878	39.1	Synechococcus phage S-SSM7	2	0	1	0	1	0	1	0	1	2	0	Viruses; dsDNA viruses, no RNA stage; Caudovirales; Myoviridae.
NC_026927	RefSeq	RVG	G386	228,143	41.6	Synechococcus phage ACG-2014f	0	0	0	1	1	1	1	1	0	2	0	Viruses; dsDNA viruses, no RNA stage; Caudovirales; Myoviridae; Tevenvirinae; T4likevirus.
NC_015569	RefSeq	RVG	G387	178,563	39.7	Synechococcus phage S-CRM01	0	0	0	0	1	0	0	0	0	1	0	Viruses; dsDNA viruses, no RNA stage; Caudovirales; Myoviridae.
NC_019516	RefSeq	RVG	G402	161,440	40.5	Cyanophage S-TIM5	0	0	0	0	1	1	1	0	1	2	0	Viruses; dsDNA viruses, no RNA stage; Caudovirales; Myoviridae.
NC_009551	RefSeq	RVG	G769	43,249	46.5	Phormidium phage Pf-WMP3	0	0	0	0	0	0	0	0	0	0	0	Viruses; dsDNA viruses, no RNA stage; Caudovirales; Podoviridae.
NC_022751	RefSeq	RVG	G769	42,480	46.4	Cyanophage PP	0	0	0	0	0	0	0	0	0	0	0	Viruses; dsDNA viruses, no RNA stage; Caudovirales; Podoviridae.
NC_024358	RefSeq	RVG	G770	41,750	43.4	Anabaena phage A-4L	0	0	0	0	0	0	0	0	0	0	0	Viruses; dsDNA viruses, no RNA stage; Caudovirales; Podoviridae.
NC_008367	RefSeq	RVG	G771	40,938	51.8	Phormidium phage Pf-WMP4	0	0	0	0	0	0	0	0	0	0	0	Viruses; dsDNA viruses, no RNA stage; Caudovirales; Podoviridae.
NC_029032	RefSeq	RVG	G818	45,532	40	Phormidium phage MIS-PhV1A	0	0	0	0	0	0	0	0	0	0	1	Viruses; unclassified phages.
NC_028998	RefSeq	RVG	G818	41,291	40.1	Phormidium phage MIS-PhV1B	0	0	0	0	0	0	0	0	0	0	1	Viruses; unclassified phages.
NC_016164	RefSeq	RVG	G1074	30,332	58.7	Synechococcus phage S-CBS1	0	0	0	0	0	0	0	0	0	0	0	Viruses; dsDNA viruses, no RNA stage; Caudovirales; Siphoviridae.
NC_015465	RefSeq	RVG	G1074	33,004	60.7	Synechococcus phage S-CBS3	0	0	0	0	0	0	0	0	0	0	0	Viruses; dsDNA viruses, no RNA stage; Caudovirales; Siphoviridae.

Table S1G. Gene annotation table of an a*ceBA* encoding TOV-EVG (TARA_ERS478052_N000008).

					HHsearch against Pfam (best hit)			HMMER against Pfam (best hit)			RPS-BLAST against COG (best hit)			
gene_id	start	stop	strand	amino acid length	accession	evalue	definition	ID	evalue	description	ID	symbol	evalue	name
TARA_ERS478052_N000008_1	3	98	-	32	-	-	-	-	-	-	-	-	-	-
TARA_ERS478052_N000008_2	103	291	-	62	-	-	-	-	-	-	-	-	-	-
TARA_ERS478052_N000008_3	291	524	-	77	-	-	-	-	-	-	-	-	-	-
TARA_ERS478052_N000008_4	569	700	-	43	-	-	-	-	-	-	-	-	-	-
TARA_ERS478052_N000008_5	750	1055	-	101	-	-	-	-	-	-	-	-	-	-
TARA_ERS478052_N000008_6	1131	1295	-	54	-	-	-	-	-	-	-	-	-	-
TARA_ERS478052_N000008_7	1313	1510	-	65	-	-	-	-	-	-	-	-	-	-
TARA_ERS478052_N000008_8	1507	1782	-	91	-	-	-	-	-	-	-	-	-	-
TARA_ERS478052_N000008_9	1787	2086	-	99	-	-	-	-	-	-	-	-	-	-
TARA_ERS478052_N000008_10	2319	2585	-	88	-	-	-	-	-	-	-	-	-	-
TARA_ERS478052_N000008_11	2585	2854	-	89	-	-	-	-	-	-	-	-	-	-
TARA_ERS478052_N000008_12	2854	3102	-	82	-	-	-	-	-	-	-	-	-	-
TARA_ERS478052_N000008_13	3102	4316	-	404	PF13203.3	8.60E-31	Putative metallopeptidase domain	-	-	-	COG3864	COG3864	1.00E-18	Predicted metal-dependent peptidase
TARA_ERS478052_N000008_14	4361	4576	-	71	-	-	-	-	-	-	-	-	-	-
TARA_ERS478052_N000008_15	4573	5736	-	387	PF07726.8	1.30E-12	ATPase family associated with various cellular activities (AAA)	-	-	-	COG0714	COG0714	2.00E-06	MoxR-like ATPase
TARA_ERS478052_N000008_16	5819	6406	-	195	-	-	-	-	-	-	-	-	-	-
TARA_ERS478052_N000008_17	6516	6662	-	48	-	-	-	-	-	-	-	-	-	-
TARA_ERS478052_N000008_18	6721	6909	-	62	-	-	-	-	-	-	-	-	-	-
TARA_ERS478052_N000008_19	7014	7355	-	113	-	-	-	-	-	-	-	-	-	-
TARA_ERS478052_N000008_20	7360	7890	-	176	PF06871.8	6.30E-14	TraH_2	-	-	-	COG0741	MltE	2.00E-08	Soluble lytic murein transglycosylase and related regulatory proteins (some contain LysM/invasin domains)
TARA_ERS478052_N000008_21	7903	8214	-	103	-	-	-	-	-	-	-	-	-	-
TARA_ERS478052_N000008_22	8273	10192	-	639	PF00362.15	2.50E-10	Integrin, beta chain	-	-	-	-	-	-	-
TARA_ERS478052_N000008_23	10238	10819	-	193	PF06941.9	5.60E-15	5' nucleotidase, deoxy (Pyrimidine), cytosolic type C protein (NT5C)	-	-	-	-	-	-	-
TARA_ERS478052_N000008_24	10816	11163	-	115	-	-	-	-	-	-	-	-	-	-
TARA_ERS478052_N000008_25	11196	11645	-	149	PF06189.9	8.80E-16	5'-nucleotidase	TIGR01689	4.10E-05	EcbF-BcbF: capsule biosynthesis phosphatase	-	-	-	-
TARA_ERS478052_N000008_26	11652	12110	-	152	-	-	-	-	-	-	-	-	-	-
TARA_ERS478052_N000008_27	12149	12511	-	120	-	-	-	-	-	-	-	-	-	-
TARA_ERS478052_N000008_28	12528	12863	-	111	-	-	-	-	-	-	-	-	-	-
TARA_ERS478052_N000008_29	12866	13441	-	191	-	-	-	-	-	-	-	-	-	-
TARA_ERS478052_N000008_30	13451	14617	-	388	-	-	-	-	-	-	-	-	-	-
TARA_ERS478052_N000008_31	14629	16980	-	783	-	-	-	-	-	-	-	-	-	-
TARA_ERS478052_N000008_32	16987	17178	-	63	-	-	-	-	-	-	-	-	-	-
TARA_ERS478052_N000008_33	17178	17816	-	212	-	-	-	-	-	-	-	-	-	-
TARA_ERS478052_N000008_34	17821	19728	-	635	-	-	-	-	-	-	-	-	-	-
TARA_ERS478052_N000008_35	19890	20522	-	210	-	-	-	-	-	-	-	-	-	-
TARA_ERS478052_N000008_36	20519	20932	-	137	-	-	-	-	-	-	-	-	-	-
TARA_ERS478052_N000008_37	20941	21450	-	169	-	-	-	-	-	-	-	-	-	-
TARA_ERS478052_N000008_38	21452	21844	-	130	-	-	-	-	-	-	-	-	-	-
TARA_ERS478052_N000008_39	21844	22167	-	107	-	-	-	-	-	-	-	-	-	-
TARA_ERS478052_N000008_40	22164	23126	-	320	PF06414.9	1.10E-20	Zeta toxin	-	-	-	-	-	-	-

TARA_ERS478052_N000008_41	23449	23850	-	133	PF00011.18	5.70E-12	Hsp20/alpha crystallin family	-	-	-	COG0071	IbpA	1.00E-15	Molecular chaperone IbpA, HSP20 family
TARA_ERS478052_N000008_42	23907	24140	-	77	-	-	-	-	-	-	-	-	-	-
TARA_ERS478052_N000008_43	24154	24546	-	130	-	-	-	-	-	-	-	-	-	-
TARA_ERS478052_N000008_44	24543	25217	-	224	-	-	-	-	-	-	-	-	-	-
TARA_ERS478052_N000008_45	25261	25542	-	93	-	-	-	-	-	-	-	-	-	-
TARA_ERS478052_N000008_46	25565	27136	-	523	PF00118.21	3.30E-56	TCP-1/cpn60 chaperonin family	TIGR02348	1.20E-165	GroEL: chaperonin GroL	COG0459	GroL	1.00E-107	Chaperonin GroEL (HSP60 family)
TARA_ERS478052_N000008_47	27173	27694	-	173	-	-	-	-	-	-	-	-	-	-
TARA_ERS478052_N000008_48	27791	28543	-	250	PF01510.22	2.50E-21	N-acetylmuramoyl-L-alanine amidase	-	-	-	COG3023	ampD	3.00E-10	N-acetyl-anhydromuramyl-L-alanine amidase AmpD
TARA_ERS478052_N000008_49	28560	29141	-	193	PF00574.20	1.00E-23	Clp protease	TIGR00493	4.00E-22	clpP: ATP-dependent Clp endopeptidase, proteolytic subunit ClpP	COG0740	ClpP	2.00E-23	ATP-dependent protease ClpP, protease subunit
TARA_ERS478052_N000008_50	29161	29508	-	115	-	-	-	-	-	-	-	-	-	-
TARA_ERS478052_N000008_51	29505	29894	-	129	-	-	-	-	-	-	-	-	-	-
TARA_ERS478052_N000008_52	30034	30864	-	276	-	-	-	-	-	-	-	-	-	-
TARA_ERS478052_N000008_53	30935	31177	-	80	-	-	-	TIGR02479	1.30E-07	FliA_WhiG: RNA polymerase sigma factor, FliA/WhiG family	COG1191	FliA	1.00E-05	DNA-directed RNA polymerase specialized sigma subunit
TARA_ERS478052_N000008_54	31174	31494	-	106	-	-	-	-	-	-	-	-	-	-
TARA_ERS478052_N000008_55	31494	31829	-	111	PF05869.8	3.50E-24	DNA N-6-adenine-methyltransferase (Dam)	TIGR01712	5.70E-13	phage_N6A_met: phage N-6-adenine-methyltransferase	-	-	-	-
TARA_ERS478052_N000008_56	31954	32157	-	67	-	-	-	-	-	-	-	-	-	-
TARA_ERS478052_N000008_57	32219	32719	-	166	PF00692.16	8.90E-33	dUTPase	TIGR00576	2.30E-26	dut: dUTP diphosphatase	COG0756	Dut	7.00E-27	dUTPase
TARA_ERS478052_N000008_58	32720	33034	-	104	-	-	-	-	-	-	-	-	-	-
TARA_ERS478052_N000008_59	33035	33253	-	72	-	-	-	-	-	-	-	-	-	-
TARA_ERS478052_N000008_60	33263	36655	-	1130	PF07733.9	2.00E-102	Bacterial DNA polymerase III alpha subunit	TIGR00594	4.00E-175	polc: DNA polymerase III, alpha subunit	COG0587	DnaE	6.00E-173	DNA polymerase III, alpha subunit
TARA_ERS478052_N000008_61	36696	36938	-	80	-	-	-	-	-	-	-	-	-	-
TARA_ERS478052_N000008_62	37007	37429	+	140	-	-	-	-	-	-	-	-	-	-
TARA_ERS478052_N000008_63	37746	39839	+	697	-	-	-	-	-	-	-	-	-	-
TARA_ERS478052_N000008_64	39912	40121	-	69	-	-	-	-	-	-	-	-	-	-
TARA_ERS478052_N000008_65	40118	40429	-	103	-	-	-	-	-	-	-	-	-	-
TARA_ERS478052_N000008_66	40431	40853	-	140	-	-	-	-	-	-	-	-	-	-
TARA_ERS478052_N000008_67	40856	41737	-	293	-	-	-	-	-	-	-	-	-	-
TARA_ERS478052_N000008_68	41787	42716	-	309	-	-	-	-	-	-	-	-	-	-
TARA_ERS478052_N000008_69	42765	43076	-	103	-	-	-	-	-	-	-	-	-	-
TARA_ERS478052_N000008_70	43382	44020	-	212	PF02511.12	6.90E-35	Thymidylate synthase complementing protein	TIGR02170	3.70E-45	thyX: thymidylate synthase, flavin-dependent	COG1351	THY1	2.00E-33	Thymidylate synthase ThyX
TARA_ERS478052_N000008_71	44073	44765	-	230	-	-	-	-	-	-	-	-	-	-
TARA_ERS478052_N000008_72	44778	45101	-	107	-	-	-	-	-	-	-	-	-	-
TARA_ERS478052_N000008_73	45130	46992	-	620	PF02867.12	5.90E-66	Ribonucleotide reductase, barrel domain	TIGR02505	7.10E-82	RTPR: ribonucleoside-triphosphate reductase, adenosylcobalamin-dependent	COG0209	NrdA	3.00E-05	Ribonucleotide reductase alpha subunit
TARA_ERS478052_N000008_74	46992	48158	-	388	PF03796.12	1.30E-33	DnaB-like helicase C terminal domain	TIGR00665	2.30E-15	DnaB: replicative DNA helicase	COG0305	DnaB	1.00E-07	Replicative DNA helicase
TARA_ERS478052_N000008_75	48210	48368	-	52	-	-	-	-	-	-	-	-	-	-
TARA_ERS478052_N000008_76	48454	49419	-	321	PF02739.13	2.60E-22	5'-3' exonuclease, N-terminal resolvase-like domain	TIGR00593	7.80E-32	pola: DNA polymerase I	COG0258	Exo	3.00E-29	5'-3' exonuclease
TARA_ERS478052_N000008_77	49421	50233	-	270	PF03332.10	9.20E-15	Eukaryotic phosphomannomutase	TIGR01484	6.80E-16	HAD-SF-IIB: HAD hydrolase, family IIB	COG0561	Cof	8.00E-13	Hydroxymethylpyrimidine pyrophosphatase and other HAD family phosphatases
TARA_ERS478052_N000008_78	50248	51432	-	394	PF00154.18	4.00E-27	recA bacterial DNA recombination protein	TIGR02012	2.50E-39	tigrfam_recA: protein RecA	COG0468	RecA	3.00E-33	RecA/RadA recombinase
TARA_ERS478052_N000008_79	51464	52195	-	243	PF08804.7	2.00E-26	gp32 DNA binding protein like	-	-	-	-	-	-	-

TARA_ERS478052_N000008_80	52247	53077	-	276	-	-	-	-	-	-	-	-	-	-
TARA_ERS478052_N000008_81	53077	53385	-	102	-	-	-	-	-	-	COG4997	COG4997	7.00E-17	Predicted house-cleaning noncanonical NTP pyrophosphatase, all-alpha NTP-PPase (MazG) superfamily
TARA_ERS478052_N000008_82	53382	54584	-	400	PF00476.17	1.00E-55	DNA polymerase family A	TIGR00593	2.40E-06	pola: DNA polymerase I	COG0749	PolA	9.00E-07	DNA polymerase I - 3'-5' exonuclease and polymerase domains
TARA_ERS478052_N000008_83	54617	55747	-	376	-	-	-	-	-	-	-	-	-	-
TARA_ERS478052_N000008_84	55747	58302	-	851	-	-	-	-	-	-	-	-	-	-
TARA_ERS478052_N000008_85	58304	58642	-	112	-	-	-	-	-	-	-	-	-	-
TARA_ERS478052_N000008_86	58639	59430	-	263	-	-	-	-	-	-	-	-	-	-
TARA_ERS478052_N000008_87	59481	61883	-	800	-	-	-	-	-	-	-	-	-	-
TARA_ERS478052_N000008_88	61892	62191	-	99	-	-	-	-	-	-	-	-	-	-
TARA_ERS478052_N000008_89	62232	62414	-	60	-	-	-	-	-	-	-	-	-	-
TARA_ERS478052_N000008_90	62431	63261	-	276	PF12322.5	1.30E-21	T4 bacteriophage base plate protein	-	-	-	-	-	-	-
TARA_ERS478052_N000008_91	63290	63826	-	178	PF06841.9	3.50E-18	T4-like virus tail tube protein gp19	-	-	-	-	-	-	-
TARA_ERS478052_N000008_92	63865	66846	-	993	PF10758.6	1.10E-27	Protein of unknown function (DUF2586)	-	-	-	COG3497	COG3497	3.00E-07	Phage tail sheath protein FI
TARA_ERS478052_N000008_93	66932	67828	-	298	-	-	-	-	-	-	-	-	-	-
TARA_ERS478052_N000008_94	67831	68322	-	163	PF11649.5	1.20E-55	Virus neck protein	-	-	-	-	-	-	-
TARA_ERS478052_N000008_95	68328	69638	-	436	-	-	-	-	-	-	-	-	-	-
TARA_ERS478052_N000008_96	69748	71496	-	582	PF07068.8	2.00E-105	Major capsid protein Gp23	-	-	-	-	-	-	-
TARA_ERS478052_N000008_97	71520	72722	-	400	-	-	-	-	-	-	-	-	-	-
TARA_ERS478052_N000008_98	72810	73217	-	135	-	-	-	-	-	-	-	-	-	-
TARA_ERS478052_N000008_99	73217	73639	-	140	-	-	-	-	-	-	-	-	-	-
TARA_ERS478052_N000008_100	73639	74052	-	137	-	-	-	-	-	-	-	-	-	-
TARA_ERS478052_N000008_101	74079	74276	-	65	PF01165.17	1.30E-12	Ribosomal protein S21	TIGR00030	8.30E-10	S21p: ribosomal protein bS21	-	-	-	-
TARA_ERS478052_N000008_102	74276	74698	-	140	-	-	-	-	-	-	-	-	-	-
TARA_ERS478052_N000008_103	74695	75264	-	189	PF03420.10	4.50E-54	Prohead core protein serine protease	-	-	-	-	-	-	-
TARA_ERS478052_N000008_104	75293	75640	-	115	-	-	-	-	-	-	-	-	-	-
TARA_ERS478052_N000008_105	75738	76028	-	96	PF07098.8	2.90E-16	Protein of unknown function (DUF1360)	-	-	-	-	-	-	-
TARA_ERS478052_N000008_106	76028	76504	-	158	-	-	-	-	-	-	-	-	-	-
TARA_ERS478052_N000008_107	76575	77408	-	277	-	-	-	-	-	-	-	-	-	-
TARA_ERS478052_N000008_108	77408	79396	-	662	PF07230.8	1.00E-99	Bacteriophage T4-like capsid assembly protein (Gp20)	-	-	-	-	-	-	-
TARA_ERS478052_N000008_109	79399	80988	-	529	PF03354.12	3.20E-32	Phage Terminase	-	-	-	COG4373	COG4373	7.00E-08	Mu-like prophage FluMu protein gp28
TARA_ERS478052_N000008_110	80994	81584	-	196	-	-	-	-	-	-	-	-	-	-
TARA_ERS478052_N000008_111	81588	82163	-	191	-	-	-	-	-	-	-	-	-	-
TARA_ERS478052_N000008_112	82167	82499	-	110	-	-	-	-	-	-	-	-	-	-
TARA_ERS478052_N000008_113	82496	82846	-	116	-	-	-	-	-	-	-	-	-	-
TARA_ERS478052_N000008_114	82914	83309	-	131	-	-	-	-	-	-	-	-	-	-
TARA_ERS478052_N000008_115	83306	84058	-	250	-	-	-	-	-	-	-	-	-	-
TARA_ERS478052_N000008_116	84074	84544	-	156	-	-	-	-	-	-	-	-	-	-
TARA_ERS478052_N000008_117	84558	85043	-	161	-	-	-	-	-	-	-	-	-	-
TARA_ERS478052_N000008_118	85043	85402	-	119	-	-	-	-	-	-	-	-	-	-
TARA_ERS478052_N000008_119	85473	86045	-	190	-	-	-	-	-	-	-	-	-	-
TARA_ERS478052_N000008_120	86047	86232	-	61	-	-	-	-	-	-	-	-	-	-
TARA_ERS478052_N000008_121	86229	86459	-	76	-	-	-	-	-	-	-	-	-	-
TARA_ERS478052_N000008_122	86496	89612	-	1038	PF02463.16	3.00E-22	RecF/RecN/SMC N terminal domain	TIGR00619	9.30E-13	sbcd: exonuclease SbcCD, D subunit	COG0420	SbcD	8.00E-26	DNA repair exonuclease SbcCD nuclease subunit
TARA_ERS478052_N000008_123	89658	90056	-	132	-	-	-	-	-	-	-	-	-	-

TARA_ERS478052_N000008_124	90171	90977	-	268	PF13362.3	1.40E-13	Toprim domain	TIGR01391	2.00E-14	dnaG: DNA primase	COG0358	DnaG	2.00E-12	DNA primase (bacterial type)
TARA_ERS478052_N000008_125	91058	91588	-	176	PF02075.14	9.40E-27	Crossover junction endodeoxyribonuclease RuvC	-	-	-	-	-	-	-
TARA_ERS478052_N000008_126	91585	91935	-	116	-	-	-	-	-	-	-	-	-	-
TARA_ERS478052_N000008_127	91960	93828	-	622	PF02973.13	8.80E-13	Sialidase, N-terminal domain	-	-	-	-	-	-	-
TARA_ERS478052_N000008_128	93852	94964	-	370	-	-	-	-	-	-	-	-	-	-
TARA_ERS478052_N000008_129	94975	95964	-	329	-	-	-	-	-	-	-	-	-	-
TARA_ERS478052_N000008_130	96007	99318	-	1103	-	-	-	-	-	-	-	-	-	-
TARA_ERS478052_N000008_131	99322	102537	-	1071	-	-	-	-	-	-	-	-	-	-
TARA_ERS478052_N000008_132	102537	103628	-	363	-	-	-	-	-	-	-	-	-	-
TARA_ERS478052_N000008_133	103625	113149	-	3174	-	-	-	-	-	-	-	-	-	-
TARA_ERS478052_N000008_134	113154	114686	-	510	-	-	-	-	-	-	-	-	-	-
TARA_ERS478052_N000008_135	114686	116488	-	600	PF04865.11	1.30E-17	Baseplate J-like protein	TIGR02243	9.60E-09	TIGR02243: putative baseplate assembly protein	-	-	-	-
TARA_ERS478052_N000008_136	116512	116901	-	129	PF10934.5	1.20E-18	Protein of unknown function (DUF2634)	-	-	-	COG3628	COG3628	9.00E-09	Phage baseplate assembly protein W
TARA_ERS478052_N000008_137	116946	124973	-	2675	-	-	-	-	-	-	-	-	-	-
TARA_ERS478052_N000008_138	124975	126051	-	358	-	-	-	-	-	-	-	-	-	-
TARA_ERS478052_N000008_139	126064	127602	-	512	-	-	-	-	-	-	-	-	-	-
TARA_ERS478052_N000008_140	127602	127895	-	97	-	-	-	-	-	-	-	-	-	-
TARA_ERS478052_N000008_141	127892	128431	-	179	-	-	-	-	-	-	-	-	-	-
TARA_ERS478052_N000008_142	128547	128891	-	114	-	-	-	-	-	-	-	-	-	-
TARA_ERS478052_N000008_143	128905	129177	-	90	PF06676.8	7.10E-13	Protein of unknown function (DUF1178)	TIGR02605	1.10E-06	CxxC_CxxC_SSSS: putative regulatory protein, FmdB family	-	-	-	-
TARA_ERS478052_N000008_144	129177	129365	-	62	-	-	-	-	-	-	-	-	-	-
TARA_ERS478052_N000008_145	129368	130333	-	321	PF16363.2	5.50E-43	GDP-mannose 4,6 dehydratase	TIGR01472	1.00E-136	gmd: GDP-mannose 4,6-dehydratase	COG1089	Gmd	2.00E-179	GDP-D-mannose dehydratase
TARA_ERS478052_N000008_146	130330	131256	-	308	PF16363.2	1.20E-38	GDP-mannose 4,6 dehydratase	TIGR01181	1.50E-24	dTDP_gluc_dehyt: dTDP-glucose 4,6-dehydratase	COG0451	WcaG	2.00E-46	Nucleoside-diphosphate-sugar epimerase
TARA_ERS478052_N000008_147	131253	132230	-	325	PF08013.8	6.70E-19	Tagatose 6 phosphate kinase	-	-	-	-	-	-	-
TARA_ERS478052_N000008_148	132256	133719	-	487	-	-	-	TIGR03992	3.90E-06	Arch_glmU: UDP-N-acetylglucosamine diphosphorylase/glucosamine-1-phosphate N-acetyltransferase	-	-	-	-
TARA_ERS478052_N000008_149	133796	134158	-	120	PF01050.15	1.60E-17	Mannose-6-phosphate isomerase	TIGR01479	2.60E-34	GMP_PMI: mannose-1-phosphate guanylyltransferase/mannose-6-phosphate isomerase	COG0662	{ManC}	6.00E-32	Mannose-6-phosphate isomerase, cupin superfamily
TARA_ERS478052_N000008_150	134196	134846	-	216	-	-	-	-	-	-	-	-	-	-
TARA_ERS478052_N000008_151	134869	135234	-	121	PF06189.9	5.20E-13	5'-nucleotidase	TIGR00099	3.10E-08	Cof-subfamily: Cof-like hydrolase	COG0561	Cof	3.00E-06	Hydroxymethylpyrimidine pyrophosphatase and other HAD family phosphatases
TARA_ERS478052_N000008_152	135362	136444	-	360	PF12897.4	2.60E-23	Alanine-glyoxylate amino-transferase	TIGR01141	1.50E-57	hisC: histidinol-phosphate transaminase	COG0079	HisC	7.00E-57	Histidinol-phosphate/aromatic aminotransferase or cobyric acid decarboxylase
TARA_ERS478052_N000008_153	136467	137294	-	275	PF05721.10	1.70E-12	Phytanoyl-CoA dioxygenase (PhyH)	TIGR02408	1.20E-05	ectoine_ThpD: ectoine hydroxylase	COG5285	COG5285	3.00E-11	Ectoine hydroxylase-related dioxygenase, phytanoyl-CoA dioxygenase (PhyH) family
TARA_ERS478052_N000008_154	137295	138083	-	262	-	-	-	-	-	-	-	-	-	-
TARA_ERS478052_N000008_155	138085	138804	-	239	PF13489.3	1.70E-12	Methyltransferase domain	-	-	-	COG4627	COG4627	4.00E-09	Predicted SAM-dependent methyltransferase
TARA_ERS478052_N000008_156	138806	139471	-	221	PF05721.10	8.50E-11	Phytanoyl-CoA dioxygenase (PhyH)	-	-	-	-	-	-	-
TARA_ERS478052_N000008_157	139553	140224	-	223	PF02348.16	2.70E-23	Cytidylyltransferase	TIGR03584	1.10E-36	PseF: pseudaminic acid cytidylyltransferase	COG1083	NeuA	1.00E-44	CMP-N-acetylneuraminic acid synthetase

TARA_ERS478052_N000008_158	140224	140994	-	256	PF00793.17	5.60E-20	DAHP synthetase I family	TIGR01362	4.80E-75	KDO8P_synth: 3-deoxy-8-phosphooctulonate synthase	COG2877	KdsA	3.00E-82	3-deoxy-D-manno-octulosonic acid (KDO) 8-phosphate synthase
TARA_ERS478052_N000008_159	140991	141740	-	249	PF00701.19	3.60E-18	Dihydrodipicolinate synthetase family	TIGR00674	5.80E-06	dapA: 4-hydroxy-tetrahydrodipicolinate synthase	COG0329	DapA	1.00E-06	Dihydrodipicolinate synthase/N-acetylneuraminate lyase
TARA_ERS478052_N000008_160	141836	142174	-	112	PF06189.9	7.90E-16	5'-nucleotidase	TIGR01689	4.40E-06	EcbF-BcbF: capsule biosynthesis phosphatase	-	-	-	-
TARA_ERS478052_N000008_161	142171	142833	-	220	PF00777.15	6.50E-13	Glycosyltransferase family 29 (sialyltransferase)	-	-	-	-	-	-	-
TARA_ERS478052_N000008_162	142838	144181	-	447	PF13522.3	2.90E-17	Glutamine amidotransferase domain	TIGR01536	6.10E-34	asn_synth_AEB: asparagine synthase (glutamine-hydrolyzing)	COG0367	AsnB	1.00E-32	Asparagine synthetase B (glutamine-hydrolyzing)
TARA_ERS478052_N000008_163	144213	144905	-	230	PF03016.12	9.40E-15	Exostosin family	-	-	-	-	-	-	-
TARA_ERS478052_N000008_164	145006	145845	-	279	-	-	-	-	-	-	-	-	-	-
TARA_ERS478052_N000008_165	145842	146456	-	204	PF05575.8	1.30E-19	Vibrio cholerae RfbT protein	TIGR01444	5.80E-13	fkbM_fam: methyltransferase, FkbM family	-	-	-	-
TARA_ERS478052_N000008_166	146468	147322	-	284	-	-	-	-	-	-	-	-	-	-
TARA_ERS478052_N000008_167	147381	148208	-	275	-	-	-	-	-	-	-	-	-	-
TARA_ERS478052_N000008_168	148205	150223	-	672	PF10111.6	6.10E-18	Glycosyltransferase like family 2	TIGR03111	8.60E-06	glyc2_xrt_Gpos1: putative glycosyltransferase, exosortase G-associated	COG0463	WcaA	2.00E-10	Glycosyltransferase involved in cell wall bisynthesis
TARA_ERS478052_N000008_169	150220	151587	-	455	PF05693.10	1.60E-21	Glycogen synthase	TIGR03999	3.90E-10	thiol_BshA: N-acetyl-alpha-D-glucosaminyl L-malate synthase BshA	COG0438	RfaG*	4.00E-15	Glycosyltransferase involved in cell wall bisynthesis
TARA_ERS478052_N000008_170	151596	152363	-	255	PF12705.4	8.90E-24	PD-(D/E)XK nuclease superfamily	-	-	-	-	-	-	-
TARA_ERS478052_N000008_171	152366	152701	-	111	-	-	-	-	-	-	-	-	-	-
TARA_ERS478052_N000008_172	152704	153387	-	227	-	-	-	-	-	-	-	-	-	-
TARA_ERS478052_N000008_173	153442	154764	-	440	PF02562.13	2.30E-25	PhoH-like protein	-	-	-	COG1875	COG1875	8.00E-156	Predicted ribonuclease YlaK, contains NYN-type RNase and PhoH-family ATPase domains
TARA_ERS478052_N000008_174	154863	155723	+	286	PF13365.3	2.20E-15	Trypsin-like peptidase domain	-	-	-	-	-	-	-
TARA_ERS478052_N000008_175	155730	156284	+	184	-	-	-	-	-	-	-	-	-	-
TARA_ERS478052_N000008_176	156281	156490	-	69	-	-	-	-	-	-	-	-	-	-
TARA_ERS478052_N000008_177	156541	156855	-	104	-	-	-	-	-	-	-	-	-	-
TARA_ERS478052_N000008_178	157316	157570	-	84	-	-	-	-	-	-	-	-	-	-
TARA_ERS478052_N000008_179	157644	157946	-	100	-	-	-	-	-	-	-	-	-	-
TARA_ERS478052_N000008_180	157963	158844	-	293	-	-	-	-	-	-	-	-	-	-
TARA_ERS478052_N000008_181	158870	159127	-	85	-	-	-	-	-	-	-	-	-	-
TARA_ERS478052_N000008_182	159137	159328	-	63	-	-	-	-	-	-	-	-	-	-
TARA_ERS478052_N000008_183	159332	161089	-	585	-	-	-	TIGR02869	7.60E-12	spore_SleB: spore cortex-lytic enzyme	COG3409	COG3409	4.00E-10	Peptidoglycan-binding (PGRP) domain of peptidoglycan hydrolases
TARA_ERS478052_N000008_184	161110	161415	-	101	-	-	-	-	-	-	-	-	-	-
TARA_ERS478052_N000008_185	161417	161617	-	66	-	-	-	-	-	-	-	-	-	-
TARA_ERS478052_N000008_186	161654	161884	-	76	-	-	-	-	-	-	-	-	-	-
TARA_ERS478052_N000008_187	161881	162330	-	149	-	-	-	-	-	-	-	-	-	-
TARA_ERS478052_N000008_188	162420	163439	-	339	PF02973.13	3.40E-12	Sialidase, N-terminal domain	-	-	-	-	-	-	-
TARA_ERS478052_N000008_189	163484	163792	-	102	-	-	-	-	-	-	-	-	-	-
TARA_ERS478052_N000008_190	163805	164377	-	190	PF16473.2	1.90E-20	3' exoribonuclease, RNase T-like	TIGR01405	4.20E-11	polC_Gram_pos: DNA polymerase III, alpha subunit, Gram-positive type	COG0847	DnaQ	2.00E-14	DNA polymerase III, epsilon subunit or related 3'-5' exonuclease
TARA_ERS478052_N000008_191	164409	165320	-	303	PF03851.11	1.30E-17	UV-endonuclease UvdE	TIGR00629	4.80E-64	uvde: UV damage endonuclease UvdE	COG4294	Uve	9.00E-60	UV DNA damage repair endonuclease
TARA_ERS478052_N000008_192	165360	165863	-	167	PF05766.9	3.60E-11	Bacteriophage Lambda NinG protein	TIGR02646	9.50E-08	TIGR02646: TIGR02646 family protein	COG1403	McrA	1.00E-15	5-methylcytosine-specific restriction endonuclease McrA
TARA_ERS478052_N000008_193	165941	166093	-	50	-	-	-	-	-	-	-	-	-	-
TARA_ERS478052_N000008_194	166103	166753	-	216	-	-	-	-	-	-	-	-	-	-
TARA_ERS478052_N000008_195	167296	168531	-	411	PF00463.18	4.30E-73	Isocitrate lyase family	TIGR01346	3.40E-178	isocit_lyase: isocitrate lyase	COG2224	AceA	0	Isocitrate lyase
TARA_ERS478052_N000008_196	168547	170055	-	502	PF01274.19	2.40E-76	Malate synthase	TIGR01344	2.70E-215	malate_syn_A: malate synthase A	COG2225	AceB	0	Malate synthase

TARA_ERS478052_N000008_197	170067	170462	-	131	-	-	-	-	-	-	-	-	-	-
TARA_ERS478052_N000008_198	170491	170724	-	77	-	-	-	-	-	-	-	-	-	-
TARA_ERS478052_N000008_199	170730	170969	-	79	-	-	-	-	-	-	-	-	-	-
TARA_ERS478052_N000008_200	170972	171202	-	76	-	-	-	-	-	-	-	-	-	-
TARA_ERS478052_N000008_201	171234	171455	-	73	-	-	-	-	-	-	-	-	-	-
TARA_ERS478052_N000008_202	171672	171878	-	68	-	-	-	-	-	-	-	-	-	-
TARA_ERS478052_N000008_203	171880	172077	-	65	-	-	-	-	-	-	-	-	-	-
TARA_ERS478052_N000008_204	172294	172449	-	51	-	-	-	-	-	-	-	-	-	-
TARA_ERS478052_N000008_205	172493	172744	-	83	-	-	-	-	-	-	-	-	-	-
TARA_ERS478052_N000008_206	172946	173251	-	101	-	-	-	-	-	-	-	-	-	-
TARA_ERS478052_N000008_207	173448	173672	-	74	-	-	-	-	-	-	-	-	-	-
TARA_ERS478052_N000008_208	174094	174273	-	59	-	-	-	-	-	-	-	-	-	-
TARA_ERS478052_N000008_209	174613	174834	-	73	-	-	-	-	-	-	-	-	-	-
TARA_ERS478052_N000008_210	175150	175413	-	87	-	-	-	-	-	-	-	-	-	-
TARA_ERS478052_N000008_211	175518	175805	-	95	-	-	-	-	-	-	-	-	-	-
TARA_ERS478052_N000008_212	175869	176135	-	88	-	-	-	-	-	-	-	-	-	-
TARA_ERS478052_N000008_213	176223	176402	-	59	-	-	-	-	-	-	-	-	-	-
TARA_ERS478052_N000008_214	177297	177425	-	42	-	-	-	-	-	-	-	-	-	-
TARA_ERS478052_N000008_215	177615	177731	-	38	-	-	-	-	-	-	-	-	-	-
TARA_ERS478052_N000008_216	177728	178069	-	113	-	-	-	-	-	-	-	-	-	-
TARA_ERS478052_N000008_217	178070	178315	-	81	-	-	-	-	-	-	-	-	-	-
TARA_ERS478052_N000008_218	178315	178494	-	59	-	-	-	-	-	-	-	-	-	-
TARA_ERS478052_N000008_219	178499	178630	-	43	-	-	-	-	-	-	-	-	-	-

* RfaG is a former name of waaG (Heinrichs *et al*., 1998; pmid:9791168)

Table S1H. PCR primer pairs for validation of OBV-EVG assemblies.

OBV-EVG	Region	Forward	Reverse
OBV_N00005	1	5'-TTGCCACCTTCTCTACGAGG-3'	5'-CGTCCTATCGATGCCTCCAA-3'
OBV_N00005	2	5'-TACGTTCTGCTTCCTCTCCC-3'	5'-TGGGGAAGTGGTATCTCAAGT-3'
OBV_N00005	3	5'-GTGAGGCCTCTTTGTTGTGG-3'	5'-TGCGTATACCCATTGTCCGT-3'
OBV_N00005	4	5'-TGTAGTCAAGACCTTCAGCGT-3'	5'-GGATGTGGTGAAAGTGTGACT-3'
OBV_N00020	1	5'-TCTTTGGATCTACGTGGGCA-3'	5'-GTTCGAGGAAGCGGAACATC-3'
OBV_N00020	2	5'-GTAGCGGTCGTTTGAACTGG-3'	5'-CTGAATCGACATCAGCAGCC-3'
OBV_N00020	3	5'-TAATAACCCTGTCGCTGCCT-3'	5'-ACTTGCGGTTCTGTAAGATTTG-3'
OBV_N00021	1	5'-TGGATTGTCGTCGCCAGTAG-3'	5'-ACCTTTCGCACCTCTCATCA-3'
OBV_N00021	2	5'-ACAGCAGAAACAAGAGTGGC-3'	5'-TTGTCAAACGCCGTAGATGC-3'
OBV_N00021	3	5'-AGAGTGGTTGAAGTGAGCGA-3'	5'-TGGACTTCTCAGCCCATTGA-3'
OBV_N00021	4	5'-TGAAGTATCACGGTGGCGTA-3'	5'-CGCTGTTGAGGTGTTGGAAT-3'
OBV_N00021	5	5'-ACTCACCCCTGAACTGTAGC-3'	5'-GCACAAGAGGCTATGGACGA-3'
OBV_N00021	6	5'-GGCATGATACCACTTCGCTC-3'	5'-CGGCAACTTCGGTACATCAG-3'
OBV_N00021	7	5'-ACCAAGTACCTTCCCACACT-3'	5'-TCGCGTTTTCCATACCACAG-3'
OBV_N00021	8	5'-CACCGTCACTACCGTCACTA-3'	5'-AACAGGCGCTACAGGTTCC-3'
OBV_N00023	1	5'-AGCTTAGCGTTGTTTCTAGTTCC-3'	5'-CGGTGAGTCTACCTACGCTA-3'
OBV_N00023	2	5'-AGAAGTCGCAATACCAAACAAG-3'	5'-TGCCTGAAACATTGACTGAACT-3'
OBV_N00023	3	5'-ACGCTGCTGAGTCACCTTTT-3'	5'-GAAGTGCAGGAAGTAGAAGAAGA-3'
OBV_N00023	4	5'-GACTTTCAGGCAAATAGCTAAAGAA-3'	5'-TTAATAAGCGACTCGCAGCC-3'
OBV_N00023	5	5'-ACATACCAACCACAGAGACCA-3'	5'-ACAATCTAACAAACCTGCGACT-3'
OBV_N00023	6	5'-TGGGAATAAATGGCTTGGGG-3'	5'-GGATCGCCAGACAAATACCA-3'

Table S1I. Seed sequences of photosynthetic genes for PSI-BLAST.

Gene	Protein ID	Species
ho1	YP_214522.1	*Prochlorococcus* phage P-SSM2
pcyA	YP_004323259.1	*Prochlorococcus* phage P-RSM4
pebS	YP_214290.1	*Prochlorococcus* phage P-SSM2
cpeT	YP_004323159.1	*Prochlorococcus* phage P-RSM4
psbA	YP_003097255.1	*Synechococcus* phage S-RSM4
psbD	YP_003097254.1	*Synechococcus* phage S-RSM4
petE	YP_003097354.1	*Synechococcus* phage S-RSM4
petF	YP_003097261.1	*Synechococcus* phage S-RSM4
ptoX	YP_003097353.1	*Synechococcus* phage S-RSM4
hli	YP_214503.1, YP_214504.1, YP_214505.1, YP_214506.1, YP_214507.1, YP_214510.1	*Prochlorococcus* phage P-SSM2
nblA	YP_851019.1	*Microcystis* phage Ma-LMM01
nblA	YP_004957295.1	*Planktothrix* phage PaV-LD
