# Supplementary material for: Environmental Viral Genomes Shed New Light on Virus-Host Interactions in the Ocean
Source: mSphere. 2017 Mar 1;2(2):e00359-16. doi: 10.1128/mSphere.00359-16 (PMC5332604; doi:10.1128/mSphere.00359-16)
Supplement: TEXT S1 [file sph002172244s9.docx]

**New method for discriminating between viral and prokaryotic contigs**

We developed a new method to discriminate between viral and prokaryotic contigs based on BLASTp sequence similarity searches with viral and prokaryotic protein sequences. We prepared the following four protein sequence datasets: prokaryotic simulation dataset (*PS*), prokaryotic evaluation (i.e., reference) dataset (*PE*), viral simulation dataset (*VS*), and viral evaluation (i.e., reference) dataset (*VE*). The *PE* protein sequences were compiled from 796 genomes. Each genome represents a distinct prokaryotic genus from the KEGG GENES database (July 2015), and we discarded those derived from prophage regions predicted by PHAST ([1](#_ENREF_1)). We also used *PE* as *PS*. The *VS* was constructed using RefSeq Viral (release 68; January 2015) by discarding redundant genomes identified by a single linkage clustering (*S*_G_ threshold = 0.8). We constructed *VE* from the phage orthologous groups ([2](#_ENREF_2)). Each phage orthologous group contains a viral quotient (*VQ*) representing the virus specificity, which was used to evaluate virus-likeness in our simulation study. We then calculated prokaryote-likeness (*L*_P_; 0 ≤ *L*_P_ ≤ 1) and virus-likeness (*L*_V_; 0 ≤ *L*_V_ ≤ 1) for the simulated contigs that were randomly extracted from *PS* (10,000 simulated contigs) and *VS* (10,000 simulated contigs). The number of proteins included in a contig was fixed to *N.* This simulation was conducted for every *N* from 1 to 100. The *L*_P_ was calculated based on the proportion of protein-coding genes that matched at least 10 proteins in *PE* during BLASTp analysis (E-value < 1e-3, excluding self-hits). The *L*_V_ was calculated as the sum of the *VQ* for the top BLASTp matches in *VE* (E-value < 1e-3, excluding self-hits) divided by *N*. The simulated contigs of *PS* and *VS* were well separated on *L*_P_–*L*_V_ plots (data not shown).

OBV and TOV contigs were discriminated using these simulated *L*_P_–*L*_V_ plots. For a given contig (*L*_P_ = *p*, *L*_V_ = *v*, and *N* = *n*), we referred to the *L*_P_–*L*_V_ plot of the 10,000 simulated *PS* contigs of *n* proteins (exception: 100 proteins when *n* > 100). We calculated the *p*-value under the null hypothesis that the contig is prokaryotic by dividing the number of simulated *PS* contigs in the area of *L*_P_ ≤ *p* and *L*_V_ ≥ *v* (i.e., less prokaryote-like and more virus-like than a given contig) by the total number of simulated *PS* contigs (i.e., 10,000). Based on the *p*-value, a false discovery rate for multiple tests was calculated using the Benjamini–Hochberg correction ([3](#_ENREF_3)). We tested 934 OBV contigs and 1,618 TOV circular contigs with this method. Twenty-three OBV contigs (all non-circular) and 59 TOV circular contigs were classified as prokaryotic (false discovery rate < 0.005).

**References**

1. **Zhou Y, Liang Y, Lynch KH, Dennis JJ, Wishart DS.** 2011. PHAST: a fast phage search tool. Nucleic Acids Res **39:**W347-352.

2. **Kristensen DM, Waller AS, Yamada T, Bork P, Mushegian AR, Koonin EV.** 2013. Orthologous gene clusters and taxon signature genes for viruses of prokaryotes. Journal of bacteriology **195:**941-950.

3. **Benjamini Y, Hochberg Y.** 1995. Controlling the false discovery rate: a practical and powerful approach to multiple testing. Journal of the Royal Statistical Society Series B (Methodological)**:**289-300.
